# Synthesis and screening of a library of Lewis^x^ deoxyfluoro-analogues reveals differential recognition by glycan-binding partners

**DOI:** 10.1038/s41467-024-51081-7

**Published:** 2024-09-13

**Authors:** Kristian Hollingsworth, Antonio Di Maio, Sarah-Jane Richards, Jean-Baptiste Vendeville, David E. Wheatley, Claire E. Council, Tessa Keenan, Hélène Ledru, Harriet Chidwick, Kun Huang, Fabio Parmeggiani, Andrea Marchesi, Wengang Chai, Ryan McBerney, Tomasz P. Kamiński, Matthew R. Balmforth, Alexandra Tamasanu, James D. Finnigan, Carl Young, Stuart L. Warriner, Michael E. Webb, Martin A. Fascione, Sabine Flitsch, M. Carmen Galan, Ten Feizi, Matthew I. Gibson, Yan Liu, W. Bruce Turnbull, Bruno Linclau

**Affiliations:** 1 School of Chemistry and Astbury Centre for Structural Molecular Biology, University of Leeds, Leeds, UK; 2 Glycosciences Laboratory, Department of Metabolism, Digestion and Reproduction, Imperial College London, London, UK; 3 Department of Chemistry, University of Warwick, Coventry, UK; 4 Manchester Institute of Biotechnology (MIB), Department of Chemistry, University of Manchester, Manchester, UK; 5 School of Chemistry, University of Southampton, Highfield, Southampton, UK; 6 Department of Chemistry, University of York, Heslington, York UK; 7 School of Chemistry, Cantock’s Close, University of Bristol, Bristol, UK; 8 Prozomix Limited, Haltwhistle Industrial Estate, Haltwhistle, Northumberland UK; 9 Division of Biomedical Sciences, Warwick Medical School, University of Warwick, Coventry, UK; 10 Department of Organic and Macromolecular Chemistry, Ghent University, Ghent, Belgium

**Keywords:** Carbohydrate chemistry, Biocatalysis, Carbohydrates, Glycobiology

## Abstract

Glycan-mediated interactions play a crucial role in biology and medicine, influencing signalling, immune responses, and disease pathogenesis. However, the use of glycans in biosensing and diagnostics is limited by cross-reactivity, as certain glycan motifs can be recognised by multiple biologically distinct protein receptors. To address this specificity challenge, we report the enzymatic synthesis of a 150-member library of site-specifically fluorinated Lewis^x^ analogues (‘*glycofluoroforms*’) using naturally occurring enzymes and fluorinated monosaccharides. Subsequent incorporation of a subset of these glycans into nanoparticles or a microarray revealed a striking spectrum of distinct binding intensities across different proteins that recognise Lewis^x^. Notably, we show that for two proteins with unique binding sites for Lewis^x^, glycofluoroforms exhibited enhanced binding to one protein, whilst reduced binding to the other, with selectivity governed by fluorination patterns. We finally showcase the potential diagnostic utility of this approach in glycofluoroform-mediated bacterial toxin detection by lateral flow.

## Abstract (web summary)

Distinguishing between different proteins that each bind to the same type of glycan is challenging. Here, the authors demonstrate that an enzymatically synthesised library of Lewis^x^ ‘glycofluoroforms’ that feature site-specific fluorination can discriminate closely related proteins.

## Introduction

**Fig. 1:**
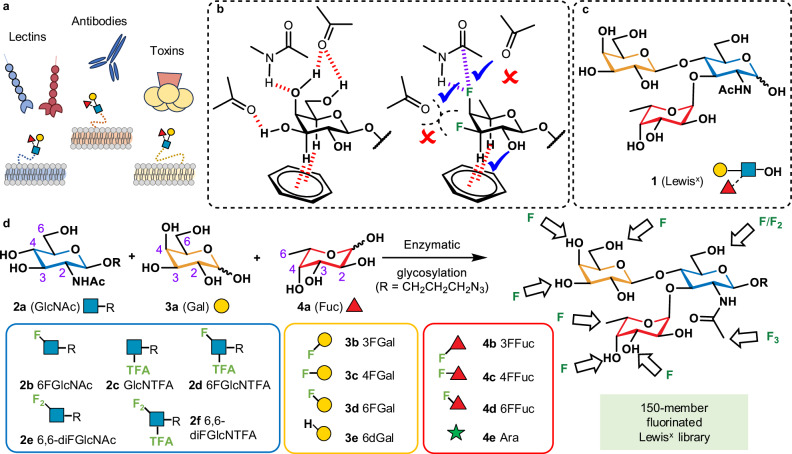
Aim of this work. **a** A glycan motif such as Lewis^x^ on surface of different cell types is bound by many different proteins, such as antibodies, lectins, and bacterial toxins. **b** Illustration of potentially favourable and unfavourable interactions upon deoxyfluorination/deoxygenation. **c** Structure of Lewis^x^. d) Depiction of chemo-enzymatic synthesis of Lewis^x^ trisaccharide and a panel of deoxyfluoro and deoxy monosaccharide building blocks **2b–f**, **3b–e**, **4b–e** used to construct a 150-member library of fluorinated Lewis^x^ analogues. Monosaccharide symbols are in accord with the updated Symbol Nomenclature for Glycans (SNFG) convention^81^. The position of each fluorine substitution is indicated by the bond angle, and anomeric configuration is shown as solid (beta) or dashed (alpha) lines, both following the Oxford Nomenclature System^82^. GlcNAc *N*-acetyl glucosamine, Gal galactose, Fuc fucose, Ara arabinose, TFA trifluoroacetyl.

Cell surface carbohydrates (glycans) play many important roles in health and disease^1^, and significant advances have been made in understanding these processes through studies of the recognition of glycan sequences by glycan-binding proteins^2^. As the same glycan motifs are sometimes recognised by multiple biologically distinct protein partners (Fig. 1a), Nature deploys local glycan concentrations to generate selectivity, not only by varying 3D presentation and density^3–6^, but also spatial-segregation: e.g., the human bronchial and alveolar epithelia interact with and are colonised by different influenza viruses^7^. Biosensing or diagnostics using glycan sequences as targets is limited by glycan cross-reactivities, for example using sialic acids or heparan sulfates as attachment sites for COVID, which have lower selectivity than antibody-based systems^8,9^. This raises the challenge of how one can develop small molecule probes for diagnostics or therapeutics that can distinguish between the interactions of different proteins with the same glycan.

The contributions of individual non-covalent interactions of proteins are routinely studied using site-directed mutagenesis. The nearest equivalent for carbohydrates is the controlled replacement of individual OH-groups with hydrogen or fluorine to reveal which OH-groups are important for binding^10,11^. Whereas deoxygenation is almost always detrimental to binding, deoxy-fluorination (-OH to -F) can potentially provide a more diverse range of effects (Fig. 1b)^12^ as fluorine is a hydrogen-bond acceptor surrogate^13,14^. Furthermore, fluorination enhances CH–π interactions involving adjacent C-H bonds^15–17^, modifies lipophilicity^18–20^, and can facilitate attractive multipolar interactions^21,22^. OH to F replacement has a minimal steric effect and does not significantly alter monosaccharide conformation, as evidenced by emerging glycan 3D structures^23,24^. Fluorination can also lead to unfavourable interaction effects, such as loss of hydrogen bonding, or mismatched dipoles or partial charges. Hence, glycan fluorination has the potential to be a powerful strategy for introducing selectivity and differentiation between proteins that recognise the same glycan^20,25^.

Lewis^x^ (Fig. 1c) is a glycan motif that is a ligand for recognition in numerous contexts with many different proteins. It is the trisaccharide capping sequence of developmentally regulated stage-specific embryonic antigen, SSEA-1^26^, and the L5 antigen involved in early neural development^27^ The Lewis^x^ motif is also expressed on tumour cells^28,29^ and occurs among human milk oligosaccharides^30^ where it has been shown to protect against toxins and pathogens involved in childhood diseases^31^. Lewis^x^ is also expressed on the surface of pathogens such as gastric cancer-causing *Helicobacter pylori*^32^, and *Schistosoma mansoni*^33^, the causative agent of the life threatening parasitic disease schistosomiasis, affecting over 200 million people worldwide.

Given the biological importance of Lewis^x^, it would be desirable, for diagnostic and therapeutic purposes, to have chemical probes that could distinguish between each of the diverse proteins with which this glycan can interact. We envisioned that fingerprint profiles of protein binding could be generated using a library of site-specific deoxyfluorinated analogues or ‘glycofluoroforms’. However, the generation of such a library constitutes a formidable synthetic challenge.

**Fig. 2:**
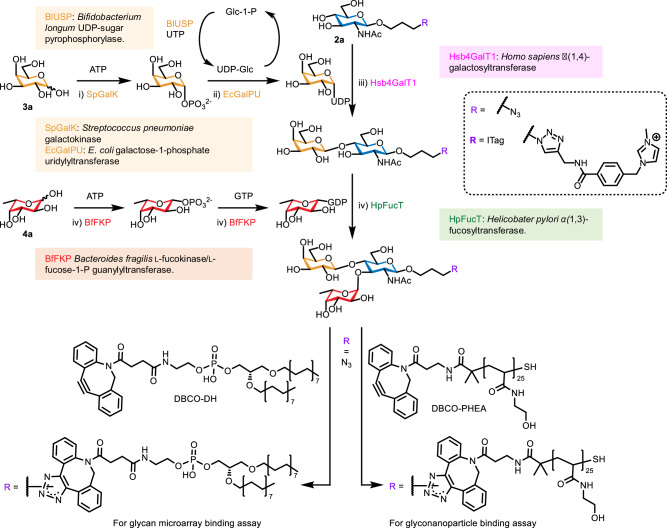
General enzymatic synthesis of Lewis^x^ glycofluoroforms. Mass spectrometry-based screening was performed using ITag derivatives before scaling up the synthesis of the azidopropyl glycans for preparation of neoglycolipid (NGL) and polyhydroxyethylacrylamide (PHEA) derivatives for use in binding assays. UDP Uridine diphosphate, ATP Adenosine 5’-triphosphate, GTP Guanosine 5’-triphosphate, DBCO-DH dibenzocycloctyne-functionalised DHPE, DHPE 1,2-Dihexadecyl-*sn*-glycero-3-phosphoethanolamine.

Here we report the synthesis of a 150-member library of Lewis^x^ ‘glycofluoroforms’ with site-specific fluorination on each monosaccharide component, by employing a diversity-orientated enzymatic assembly process (Fig. 1d), featuring a wide substrate scope. We investigate a 24-member subset of this library, for unique fluorination patterns that may be discerned by Lewis^x^ binding proteins. These include antibodies and closely related glycan-binding proteins and bacterial toxins. Our design strategy involves adding azido-propyl handles to the glycans so that they can be converted into positively charged imidazolium-based tags (ITags) for reaction screening. These handles also enable facile conversion of a 24-member subset library into lipid-linked glycan probes (neoglycolipids, NGLs), or into glyconanoparticles (Fig. 2) for probing protein binding both in solid-phase high-throughput glycan microarray screening analyses^34^ and in solution-phase nanoparticle-based interaction studies^4^.

## Results

### Enzymatic synthesis

The glycan library was constructed using a panel of chemically synthesised monosaccharide derivatives based on glucosamine **2a**, galactose **3a** and fucose **4a**. *N*-Acetyl/*N*-trifluoroacetyl glucosamine derivatives included mono-and di-deoxyfluorination at C-6 (Fig. 1), and galactose and fucose derivatives were monodeoxyfluorinated at positions 3, 4 or 6. d-Fucose **3e** was also included as 6-deoxy-d-galactose, and d-arabinose **4e** as a de-methylated l-fucose analogue.

**Fig. 3:**
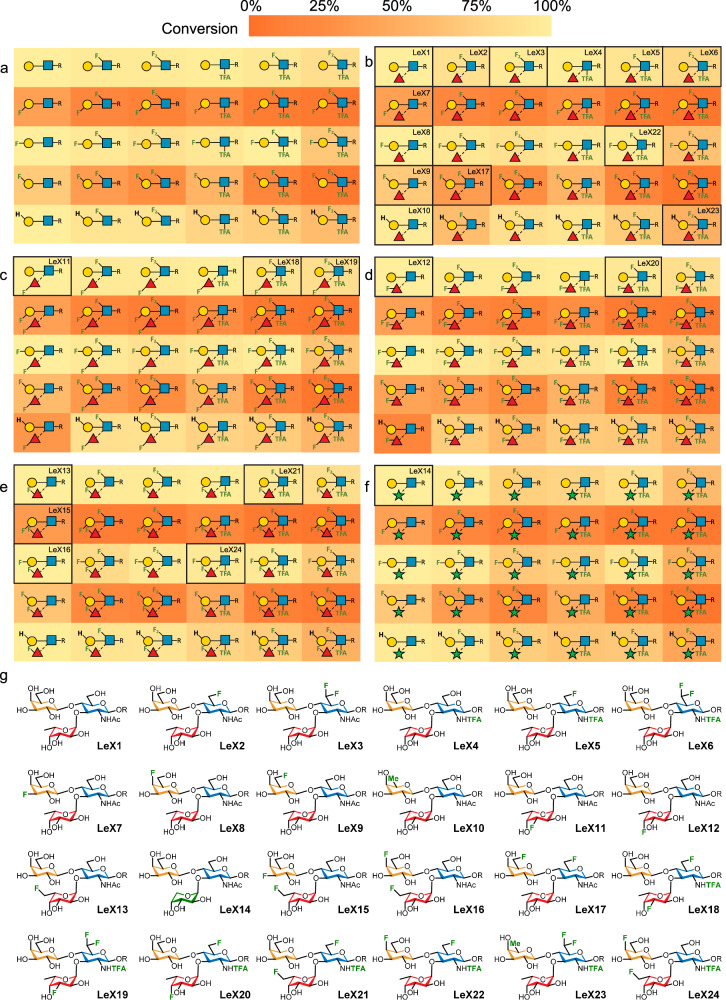
Enzymatic synthesis of Lewis^x^ analogues. The matrix depicts ESMS-derived conversion efficiencies of GlcNAc derivatives bearing ITags to (**a**) LacNAc analogues, and (**b**–**f**) Lewis^x^ analogues containing fucose, 3FFuc, 4FFuc, 6FFuc, and Ara, respectively (See Supplementary Table 1 for numerical data). **g** A sub-library of 24 Lewis^x^ analogues (R = azidopropyl), synthesised on a multi-milligram scale for binding assays, is also indicated in (**b**–**f**) with black boxes. The source data for Fig. 3 are provided in the supporting information file (Supplementary Figs. 18–197).

The fluorinated/deoxy UDP-Gal analogues were synthesised using a one-pot multienzyme system (Fig. 2)^35^, with each galactose analogue (**3a**-**e**) first converted to the corresponding sugar-1-phosphate using *Streptococcus pneumoniae* galactokinase (SpGalK)^36,37^, before transfer of UMP from UDP-Glc by *E. coli* galactose-1-phosphate uridylyltransferase (EcGalPU) and in-situ regeneration of UDP by *Bifidobacterium longum* UDP-sugar pyrophosphorylase (BlUSP) (Fig. 2)^38^. Crude preparations of each UDP derivative were used in subsequent glycosylation reactions. To aid assembly of the LacNAc disaccharide core, an electrospray-mass spectrometry (ESMS) screening protocol was established to investigate the substrate scope for enzymatic galactosylation of GlcNAc library **2a-f** with UDP-Gal library UDP-**3a-e** using *Homo sapiens* β(1,4)-galactosyltransferase (Hsb4GalT1), which showed high tolerance of fluorination at the GlcNAc 6-position and trifluorination of the *N*-acetyl group, and could be easily expressed in *E. coli* as a fusion protein with maltose-binding protein. Conversion of the azidopropyl aglycon to a positively charged imidazolium-based tag (ITag, Fig. 2)^39^ enabled semi-quantitative MS-analysis by ensuring that both the starting material and product were fully ionised and thus detectable by ESMS with comparable efficiency, allowing an estimation of their relative conversion^40,41^. Formation of all thirty LacNAc analogues (Fig. 3a) was thus confirmed. All six GlcNAc derivatives were accepted as substrates by the Hsb4GalT1 enzyme, with high conversion when using the natural UDP-Gal donor; all other UDP-Gal analogues also accepted but with significant differences in conversion based on the position of fluorination/deoxygenation. Specifically, UDP-Gal, UDP-4FGal and UDP-6dGal were all substrates for HsB4GalT1 with non-optimised conversions generally over 60%, whilst UDP-3FGal and UDP-6FGal were poorer substrates with most conversions below 40%, which can be partially rationalised by crystal structures of β(1,4)-galactosyltransferase bound to UDP-Gal (Supplementary Fig. 1)^42^.

The library of thirty LacNAc acceptors was then glycosylated with fucoses **4a-e**, to give a total of 150 possible Lewis^x^ glycofluoroforms (Fig. 3b–f). A one-pot, two-step glycosylation reaction in which the crude product mixture from galactosylation of **2a**–**f** was directly fucosylated using 1 molar equivalent of an α-(1,3)-fucosyltransferase (HpFucT) from *H. pylori*^43^ and in-situ generated GDP-fucose donors^44^. Fucosylation proved to be much more efficient than galactosylation, with the >80% relative conversion compared to **LeX1**, for fucosylation in 147 out of 149 reactions. Increased fluorination generally led to lower conversion although LacNAc analogues containing 6,6-diFGlcNTFA showed full comparative conversion in several cases. It was satisfying to note that the promiscuity of HpFucT^44^ facilitated turnover of our broad range of modified LacNAc acceptors. The crystal structure of HpFucT bound to GDP-Fuc shows that C-6 is surface exposed and therefore modifications at this position would be less likely to affect interactions between enzyme and substrate (Supplementary Fig. 2)^45^. In contrast, both the O-3/O-4 positions of GDP-Fuc form potential H-bonds to the enzyme so interruption of their ability to act as hydrogen bond donors would be expected to disrupt binding to the enzyme however both 3FFuc-GDP and 4FFuc-GDP were successfully turned over. The two notable exceptions to this observation were addition of 3FFuc and 4FFuc to 6dGal-β(1,4)-GlcNAcOR. Of the 150 Lewis^x^ glycans identified by ESMS, 140 glycofluoroforms are novel compounds including, to the best of our knowledge, the first examples of fluorination to more than one monosaccharide in a Lewis^x^ analogue.

### Preparative scale synthesis and glycofluoroform characterisation

In order to obtain high-quality binding data, it was decided to use purified and fully characterised analogues instead of crude enzyme-synthesis reaction mixtures. Hence, a set of 24 compounds (Fig. 3g) was selected for preparative-scale synthesis, which included all single fluoro/deoxy monosaccharides employed in the screen and examples with fluorination in two and three of the monosaccharide residues. Following optimisation, synthetically useful conversion efficiencies were achieved, including examples for which screening had shown relatively poor conversion (**LeX15,** **LeX17**, and **LeX23** which showed 30–50% conversion in the screen), demonstrating the scalability of our approach. Nuclear magnetic resonance (NMR) spectroscopic analysis of the scaled-up library of 24 trisaccharides confirmed that all of these had the correct Lewis^x^ structure, with all analogues adopting the well-defined ‘closed conformation’ when in aqueous medium. This was deduced from the increased chemical shift of fucose H-5, which is known to result from a non-conventional CH-O hydrogen bond between fucose H-5 and the ring oxygen of the galactosyl residue (Supplementary Fig. 5)^46^. Another consequence of this ‘closed conformation’ is the proximity of the fucose H-6 and galactose H-2 positions, which is believed to provide hydrophobic stabilisation to the closed conformation of Lewis^x^ ^46,47^. NMR analysis of the 24 glycans revealed a distinct increase, averaging 0.12 ppm, of the galactose H-2 chemical shift for those Lewis^x^ analogues that contain a 6-fluorofucosyl residue, when compared to analogues with a normal fucosyl residue (Supplementary Fig. 6). It is postulated that this change results from an interaction of Fuc F-6 with Gal H-2, which is taken as further evidence of the preservation of the Lewis^x^ 3D-conformation in aqueous medium upon fluorination.

### Glycan microarray construction and screening

**Fig. 4:**
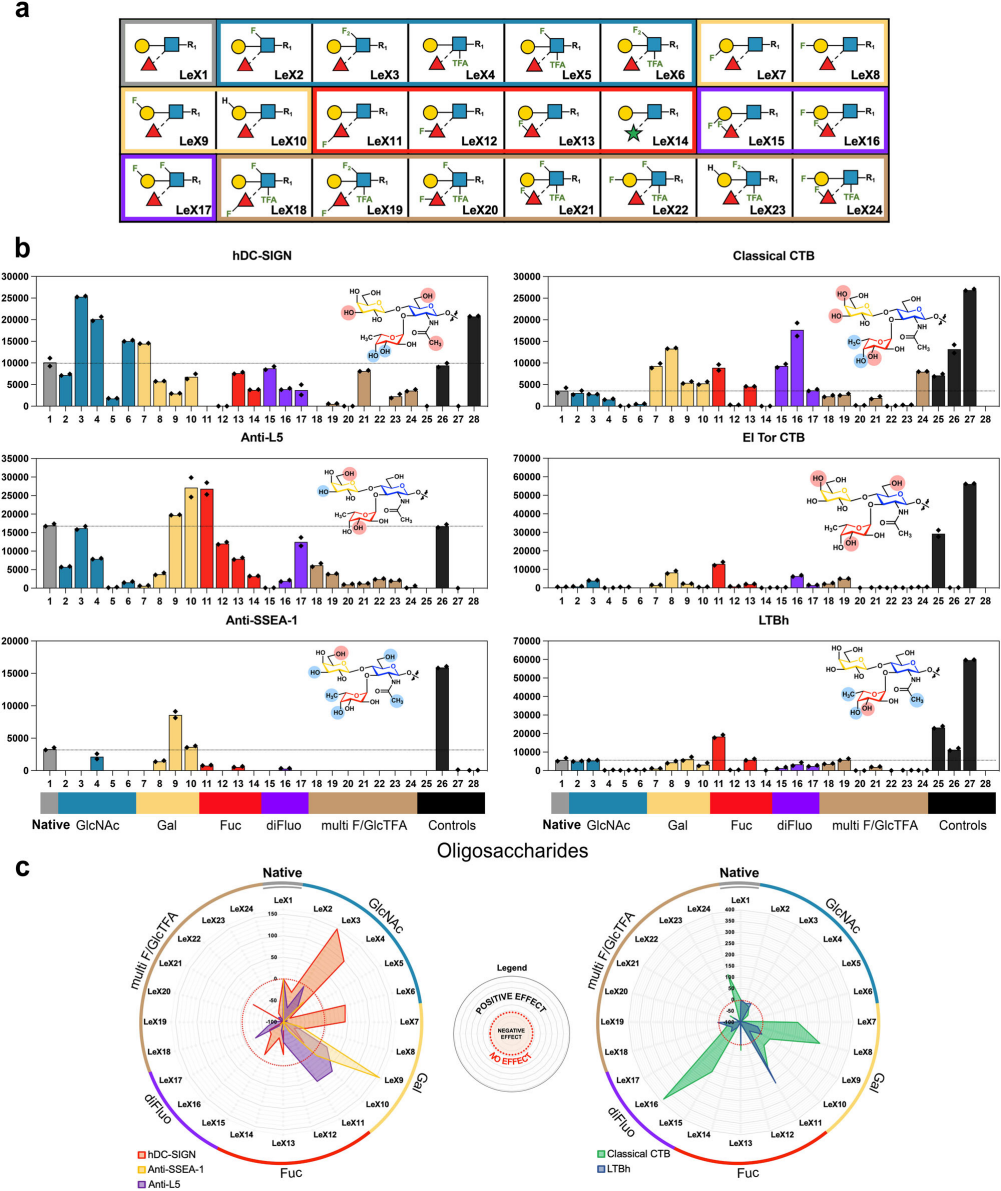
Microarray analyses of 24 Lewis^x^ NGL probes. **a** Symbolic forms of the 24 Lewis^x^ analogues; **b** Binding signals with the six glycan-binding proteins presented as histogram charts. Positions 1 to 24 are NGLs corresponding to **LeX1** to **LeX24** shown in (**a**). The four control probes are at positions 25 to 28 (Supplementary Table 2). Numerical scores are average fluorescence intensities of the duplicate spots, with the two individual values displayed. The results shown are representative of at least three experiments. The differing effects of fluorination and other modifications at different sites of the Lewis^x^ trisaccharide are summarised using colour shading: red, strong enhancement; blue, abolished binding. **c** Spider charts showing distinctive binding modes of Lewis^x^ glycofluoroforms observed in microarray analyses. The signal intensities of the unnatural Lewis^x^ probes (**LeX2** to **LeX24**) are normalised against **LeX1** and the difference is presented as percentage value (%) in the spider charts. Positive and negative values mean enhanced and diminished binding, respectively, compared to that of **LeX1**. Zero (red dotted circle line) means the same binding intensity as **LeX1**. DC-SIGN Dendritic cell-specific intracellular adhesion molecule-3-grabbing nonintegrin, CTB *Vibrio cholerae* toxin B-Subunit, LTBh *Escherichia coli* heat-labile toxin. The raw fluorescence intensities of the quantified microarray data are provided as Source data file (excel file).

The 24 Lewis^x^ trisaccharide analogues (**LeX1** to **LeX24**) (Figs. 3g/4a) were converted into NGL probes to allow their non-covalent immobilisation on nitrocellulose-coated microarray slides. The clustered and flexible presentation of glycans in the NGL-based microarray system is advantageous in revealing binding by a diverse range of glycan binding systems, including endogenous carbohydrate binding receptors, adhesins of microbes, virus particles or their adhesins^34,48^. We used a new procedure for NGL preparation that takes advantage of the efficiency of the strain-promoted azide-alkyne cycloaddition (SPAAC, Fig. 2)^49^. The Lewis^x^ analogues were conjugated to a new lipid reagent dibenzocycloctyne-functionalised DHPE (DBCO-DH) to generate the desired DBCO-DH-NGL probes in quantitative yields. A microarray was then constructed with the 24 Lewis^x^-based DBCO-DH-NGL probes which were prepared, quantified and arrayed in parallel (Fig. 4). The unmodified Lewis^x^ trisaccharide (**LeX1**) served as the the control for the modified glycan probes including those with modification of the GlcNAc residue only (**LeX2**–**LeX6**); modification of Gal only (**LeX7**–**LeX12**), mono-fluorination of Fuc (**LeX13** and **LeX14**); di-fluorination on two monosaccharide residues (**LeX15**–**LeX17**); multi-fluorinated structures with modification on two or more sugar residues (**LeX18**–**LeX24**). Four conventional NGLs derived from natural oligosaccharides were included as reference compounds to show that the the glycan-binding proteins analysed had the predcited binding activities. These were lacto-*N*-neotetraose (LNnT, position 25), its fucosylated analogue Lewis^x^ pentasaccharide lacto-*N*-fucopentaose III (LNFP III, 26), pentasaccharide of GM1 ganglioside (27), and high-mannose *N*-glycan Man9GN2 (28) (Supplementary Table 2). The microarray was probed with nine glycan-binding proteins (Supplementary Table 3): three recombinant Fc-tagged C-type immune lectin receptors, three anti-Lewis^x^ monoclonal antibodies, and three closely related bacterial toxins. The results of microarray analyses are shown in Fig. 4, Supplementary Figs. 7, 8, and in the Source Data file.

Monoclonal anti-Lewis^x^ antibodies: With high specificity for Lewis^x^ among the histo-blood group carbohydrate determinants, anti-Lewis^x^ antibodies are widely used as research tools for identifying Lewis^x^ expressed on different organisms, tissues, and cells. The monoclonal antibodies anti-SSEA-1^26^ and anti-L5^27^ together with a commercial antibody BG-7 were analysed in the microarray, and all gave binding to native Lewis^x^ probes **LeX1** and the LNFPIII standard. Distinct binding patterns were observed with the three antibodies: anti-SSEA-1 showed the most restricted and anti-L5 the broadest binding profiles (Fig. 4c, Supplementary Fig. 10). Earlier studies have shown that anti-SSEA-1 recognises Lewis^x^ epitope on a longer carbohydrate backbone compared to anti-L5^50^, and this is corroborated by the weaker binding detected to the **LeX1** than LNFPIII in the present study. A clear enhancement (almost 3-fold) of the binding of anti-SSEA-1 was observed with the 6F-Gal **LeX9** compared to the native **LeX1**. The **LeX9** was also the most potent ligand for BG-7 among all the Le^x^ analogues. Interestingly, anti-L5 showed preferential binding to 6H-Gal (**LeX10**) compared to 6F-Gal (**LeX9**). The 3F-Gal, as in **LeX7**, abolished binding by all antibodies, indicating that the 3-OH group is likely a key hydrogen bond donor in the interactions of these antibodies. Most of the fluorine modifications on GlcNAc resulted in a reduction or abolition of antibody binding, except for C-6 di-fluorination as in **LeX3**, which was well tolerated by anti-L5 but not at all by anti-SSEA-1 and BG-7 (Fig. 4c, Supplementary Figs. 8–10). The GlcNAc N-TFA modification alone was tolerated by all of the antibodies, whereas little binding was detected when this modification occurred in combination with other fluorination in the Lewis^x^ trisaccharide. Modifications on Fuc had differential effects on antibody binding: **LeX11** with 3F-Fuc was strongly bound by anti-L5 but only moderately bound by BG-7 and not at all by anti-SSEA-1. The **LeX14** which has arabinose instead of Fuc in the trisaccharide structure gave no binding with anti-SSEA-1 and BG-7 and very weak binding with anti-L5, highlighting the importance of methyl group at the Fuc C-5 position for binding by the two antibodies.

C-type immune lectins (CLRs): Dendritic cell-specific intracellular adhesion molecule-3-grabbing nonintegrin (DC-SIGN), its closely related protein DC-SIGNR and the Langerhans cell CLR Langerin were chosen for the microarray study. Whilst DC-SIGN is known to bind both to mannose- and fucose-terminating glycan sequences including Lewis^x^ ^51–54^, the lack of Lewis^x^ binding by the other two CLRs has been widely reported^55–57^; and thus DC-SIGNR and Langerin mainly served for comparison with DC-SIGN. As predicted, all the three CLRs gave strong binding signals with the *N*-glycan standard Man9GN2 (position 28) (Fig. 4b, Supplementary Fig. 10), and only human DC-SIGN (hDC-SIGN) showed significant binding to the unmodified Lewis^x^ probes **LeX1** and the LNFPIII standard (position 26). For DC-SIGN, 17 out of 23 unnatural Lewis^x^ probes showed binding, among these there was superior binding to four probes compared to the unmodified **LeX1**. The positive influence on hDC-SIGN binding was mainly the fluorination on the GlcNAc residue: di-fluorination at C-6 position (**LeX3**) followed by the *N*-trifluoroacetyl (N-TFA) modification (**LeX4**), and to a lesser extent a combination of these two modifications as in **LeX6**. Fluorination at C-3 of Gal (as in **LeX7**) also resulted in an enhanced binding by hDC-SIGN, in contrast to the 4F-, 6F- and 6dH-Gal modifications which elicited a negative effect to various degrees. It is striking that 3F- and 4F-modification of the Fuc residue completely abolished hDC-SIGN binding; this is in full agreement with the essential roles of the OH groups at these two positions of the α1-3-linked Fuc in forming coordination bonds with Ca^2+^ ^55,58^. With hDC-SIGNR and rhesus CLR Langerin, only very weak binding was detected to selected fluorinated Lewis^x^ probes (Supplementary Fig. 7) suggesting only a subtle gain in affinity effect through fluorination.

Bacterial Toxins*:* The glycan-binding subunits of three structurally related bacterial toxins were also investigated: *Vibrio cholerae* toxin B-Subunit (CTB) of the O1 classical biotype (Classical CTB) and El Tor biotype (El Tor CTB), along with the enterotoxigenic *Escherichia coli* heat-labile toxin (LTBh). Cholera toxin, secreted by *V. cholerae*, is the major virulence factor in cholera infection, a life-threatening diarrhoeal disease. The cholera toxins and *E. coli* heat-labile toxin, which share 80% sequence identity, all bind strongly to GM1 ganglioside^59,60^. However recent studies have identified a secondary carbohydrate binding site that also recognises histo-blood group antigens, such as Lewis^x^^61–65^, and is important for cellular uptake and toxicity in epithelial cells in the small intestine^66,67^. As anticipated, all three toxins gave strong binding to the GM1 ganglioside standard (position 27, Fig. 4b) and the Gal-terminating tetrasaccharide LNnT NGL (position 25)^68^. Binding to the unmodified Lewis^x^ trisaccharide **LeX1** and LNFPIII standard was observed with classical CTB and LTBh, but not with El Tor CTB which was confirmed by isothermal titration calorimetry (Supplementary Fig. 11). With the library of modified Lewis^x^ trisaccharide NGLs, unique binding profiles were observed with each toxin sample. Classical CTB showed dramatically enhanced binding to several fluorinated Lewis^x^ probes that have modifications on Gal and Fuc. The most significant ‘enhancer modifications’ are 3-F and 4-F on Gal and 3-F on Fuc. **LeX16** having both 4F-Gal and 6F-Fuc modifications was found to be the strongest ligand with almost four-fold increase of binding intensity compared to that of **LeX1**. In contrast, the N-TFA modification, 4F-Fuc and replacing Fuc with arabinose resulted in a substantial decrease in CTB binding. The observed changes in binding strength are consistent with structural information for the Classical CTB-Lewis^x^ interaction (see supplementary discussion)^65^. With LTBh, most of the modifications had little effect or resulted in diminished binding; an exception was 3F-Fuc-modified **LeX11** which showed marked signal enhancement. Although the unmodified Lewis^x^ was not bound by the El Tor CTB, weak but detectable binding was observed to a few fluorinated probes including 4F-Gal (**LeX8**) and 3F-Fuc (**LeX11**).

### Gold nanoparticle-linked assay

**Fig. 5:**
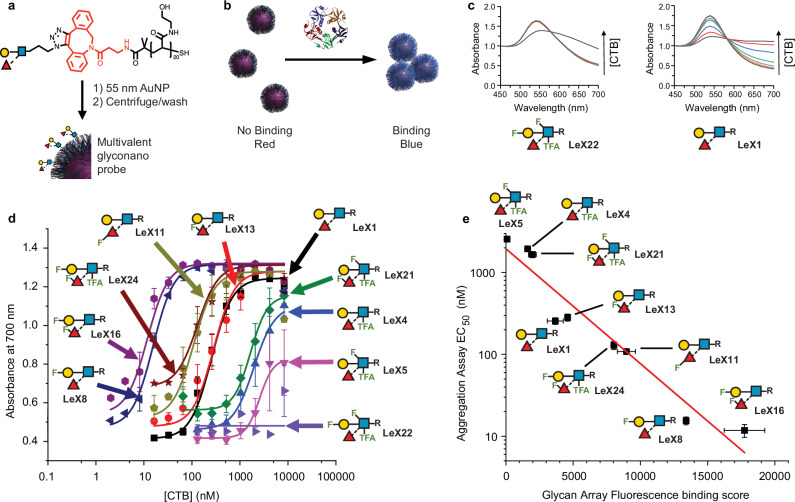
Glyconanoparticle-based sensing of CTB. **a** Polymer-tethered glyconanoparticles; **b** Principle of detection due to gold nanoparticle red-blue shift upon aggregation with CTB; **c** UV-Visible spectra for gold nanoparticles with native Lewis^x^ (**LeX1**) and fluorinated **LeX22**; **d** Dose-dependent response of library of Lewis^x^ glyconanoparticles to CTB. Data is presented as mean normalised Abs700 from UV-Visible spectroscopy ± standard deviation of 3 replicates. Control experiments are shown in Supplementary Fig. 17a–c. **e** Correlation of glycan array and glyconanoparticle binding data **LeX22**, which had a glycan array binding score of 55 ± 53, is omitted from the correlation graph as binding was too weak to be quantified in the nanoparticle assay. Pearson correlation analysis: r(7) = −0.96, *p* = 0.000041 (two-sided). Error bars on the x-axis correspond to values of duplicate measurements, and error bars on the *y*-axis correspond to the fitting uncertainty reported for EC_50_ values from the data shown in Fig. 5d. The gold nanoparticle binding data generated in this study have been deposited in the University of Manchester Figshare database (https://figshare.manchester.ac.uk).

A colorimetric gold nanoparticle-linked assay was developed to provide a comparative validation for a subset of the microarray results, and to demonstrate that the discriminatory power of the fluorinated glycans is independent of biosensor architecture. Gold nanoparticles can be deployed in solution-based assays, where a target protein aggregates particles, leading to coupling of their surface plasmon resonance bands, and a macroscopic red-blue colour shift^69,70^. A subset of 10 azido-propyl Lewis^x^ analogues were conjugated onto poly(hydroxyethyl acrylamide) (PHEA)^71^ by SPAAC (Fig. 2), which was then loaded onto 55 nm gold nanoparticles^25^. Glyconanoparticle aggregation on addition of the classical biotype *V. cholerae* toxin CTB was monitored by UV-Visible spectroscopy (Fig. 5b/c). Figure 5d shows complete dose-dependent binding responses of the Lewis^x^ glycofluoroforms versus CTB. **LeX8** and **LeX16** show a 23-fold decrease in EC50 compared to the native Lewis^x^ glycan **LeX1**. **Lex11** and **LeX24** show a 5-fold improvement in binding strength, while with **LeX13** the binding was similar to that with **LeX1**. The results correlated very well with the microarray assay results (Fig. 5e).

## Discussion

Overall, our microarray and nanoparticle binding studies reveal that different proteins with a Lewis^x^ binding site have distinctive recognition modes for this important trisaccharide, which is illustrated by the contrasting binding preferences and tolerances of monoclonal Lewis^x^ antibodies, DC-SIGN, and bacterial toxins (Fig. 4c, Supplementary Fig. 10). These Lewis^x^-binding proteins have different responses to the site-specific deoxy/fluorine modifications, as summarised for the 11 individual site modifications in Supplementary Table 4. Importantly, deoxygenations/deoxyfluorinations are able to either enhance or reduce affinities, as exemplified in classical CTB and hDC-SIGN binding studies. The minimal binding of fluorinated Lewis^x^ analogues to DC-SIGNR, which is closely related to DC-SIGN but does not bind to Lewis^x^, indicates that enhancements in binding strength did not arise from non-specific interactions. The high levels of selectivity that can be achieved by introducing fluorine at multiple sites on the oligosaccharide is illustrated by **LeX11** (fluorination at the fucose 3-position), which showed enhanced binding to the classical CTB bacterial toxin but was not tolerated by hDC-SIGN, and vice versa by **LeX6** (fluorination at two GlcNAc positions) which showed enhanced binding to hDC-SIGN, but a much reduced binding to classical CTB. The difluorinated **LeX16** (4F-Gal and 6F-Fuc modification) which was identified as the most potent and unique ligand for the classical CTB, but was only very weakly bound by the *E. coli* LTB. Notably **LeX16** showed significantly reduced binding by hDC-SIGN and the anti-Lewis^x^ antibodies compared to native **LeX1**. These fluorination patterns represent a promising framework for design of specific diagnostic or therapeutic agents, for example against pathogenic bacteria that do not interfere with endogenous immune lectins.

As a proof of concept towards the application of glycofluoroforms for diagnostic purposes, a lateral flow experiment using a low-cost glyco-assay was developed (Supplementary Figs. 271, 272). We demonstrated the discrimination between AuNP functionalised with a low-binding glycan (**LeX4**) vs those coated with a high-binding glycan (**LeX16**) for the detection of CTB. This unequivocally shows that the microarray and aggregation results can be translated into diagnostic tools.

Here we have demonstrated a practical and expedient diversity-oriented enzymatic strategy devised to generate a structurally diverse library of mono- and polyfluorinated Lewis^x^ derivatives, termed ‘glycofluoroforms’. We have prepared a 24 member library of isolated and fully characterised glycans out of a total of 150 glycans that were generated and identified by mass spectrometry using a clickable ITag for reaction monitoring. Only wild-type forms of the enzymes were employed, and they showed high tolerance for nucleotide-activated monofluorinated sugar donors and their glycosylation of fluorinated acceptors, with all possible 150 Lewis^x^ glycans generated from six GlcNAc acceptors, five Gal, and five Fuc donors. A third of the Lewis^x^ derivatives were formed with conversions of at least 89%, half had conversions over 70% and almost two thirds had conversions over 40%. A total of 24 glycans, chosen to achieve structural diversity, were successfully upscaled and characterised, even for those cases that showed low (30–50%) conversion in the screening experiments.

Distinctive glycofluoroform fingerprint binding profiles for nine glycan-binding proteins were obtained by screening of a NGL-based microarray using the 24 analogues prepared in multi-milligram scale. A gold nanoparticle-based assay was successfully used to validate the relative binding affinities of a subset (10 glycans) of the microarray results. A number of examples of increased binding strength of Lewis^x^ glycofluoroforms compared to the native glycan were also identified, notably between monoclonal Lewis^x^ antibodies (3 examples) and bacterial toxins (3 examples) investigated, with different preferred fluorination patterns for different proteins. Thus, using wild-type enzymes, this study has established that the facile construction of extensive libraries of a given glycan structure with defined fluorination patterns across multiple monosaccharides, using wild-type enzymes, is a powerful approach to investigating protein-glycan binding specificities. This validated ability of glycan fluorination patterning to deliver significant changes in protein binding whilst maintaining glycan conformation, heralds great promise for the development of diagnostic kits or new anti-adhesion therapies to combat microbial infections. We anticipate that our preliminary data demonstrating application of the glycofluoroform approach for lateral flow detection of a bacterial toxin will accelerate the development of glycan-based diagnostics, and therapeutics.

## Methods

### Synthesis of the Lewis^x^ trisaccharides

Materials and methods: 3-Deoxy-3-fluorogalactose, 4-deoxy-4-fluorogalactose, 6-deoxy-6-fluorogalactose, 6-deoxygalactose, UDP-galactose, fucose, arabinose, ATP, GTP and UTP were received as in-kind support from Biosynth Carbosynth. GlcNAc acceptors **2a**-**2f**^25^, 3FFuc **4b**^72^, 4FFuc **4c**^58^, 6FFuc **4d**^73^ were prepared by literature methods. ^1^H NMR spectra were recorded at 500 MHz on a Bruker AV4 NEO 11.75 T (500 MHz ^1^H) NMR spectrometer. ^13^C NMR spectra were recorded at 125 MHz on a Bruker AV4 NEO 11.75 T (500 MHz ^1^H) NMR spectrometer. ^19^F NMR spectra were recorded at 376.5 MHz on a Bruker AV3HD 9.4 T (400 MHz ^1^H) NMR spectrometer. Chemical shifts are given in parts per million downfield from tetramethylsilane. The following abbreviations are used in ^1^H NMR analysis: s = singlet, d = doublet, t = triplet, q = quartet, m = multiplet, dd = double doublet, dt = double triplet, td = triple doublet, ddd = double double doublet. Accurate Mass spectra were acquired on a Bruker Impact II QqTOF spectrometer equipped with a VIPHESI source using electrospray ionisation. Samples were introduced using an HTC PAL autosampler and Bruker Elute Pump. A 50:50 MeOH:water mix (0.1% formic acid) was used as the eluant. Samples passed through a Bruker Diode array uv-detector before entering the mass spectrometer. Calibration was performed by infusion of 5 mM sodium formate solution at the end of each acquisition. Silica chromatography was performed using a Biotage Isolera 4 with Ecopack basic D17 silica columns. Fractions were analysed by TLC using an DCE:MeOH:AcOH:H_2_O (50:30:25:10) resolving solvent and visualised with 0.2% orcinol and 5% sulfuric acid in MeOH with charring. Size exclusion was performed using an AKTA Prime with a 10 × 300 mm Tricorn column packed with Bio-rad Biogel P2. This method was run at 0.1 mL/min in water collecting 0.5 mL fractions with detection TLC. A 26 × 1000 mm Tricorn column packed with Bio-rad biogel P2 was used for more difficult separations and was run at 0.5 mL / min collecting 2 mL fractions. HPLC was performed on an Agilent 1260 Mass Directed Preparative HPLC using a Kinetex 5 um EVO C18 100 Å LC column 250 ×21.2 mm.

### Enzymes

BlUSP: *Bifidobacterium longum* UDP-sugar pyrophosphorylase (Uniprot code—C2GXC3)

SpGalK: *Streptoccocus pneumoniae* galactokinase (Uniprot code—B1I864)

EcGalPU: *Escherichia coli* galactose-1-phosphate uridylyltransferase (Uniprot code—P07902)

BfFKP: *Bacteroides fragilis* l-fucokinase/l-fucose-1-P guanylyltransferase (Uniprot code—Q58T34)

HpFucT: *Helicobacter pylori* α(1,3/4)-fucosyltransferase (Uniprot code—O30511)

Hsb4GalT1. *Homo sapiens* β(1,4)-galactosyltransferase (Uniprot code—P15291).

HpFucT^43^, BfFKP^74^, spGalK^36^, EcGalPU^35^ and BlUSP^38^ were obtained as in-kind support from Prozomix. They were expressed in *E. coli* BL21 as His-tagged proteins, and stored as ammonium sulfate precipitates following purification by nickel affinity chromatography.

Hsb4GalT1 did not express in a soluble form when expression was attempted using the same approach employed for the other enzymes. Instead it was expressed as follows: a synthetic gene coding for residues 61-398 of human B4GalT1, codon-optimised for expression in *E. coli*, and with an *N*-terminal TEV protease recognition sequence (ENLYFQ/G), was cloned between the BamHI and HindIII sites of a modified version of pDB.His.MBP^75^, in which the nucleotides encoding the original TEV sequence and the multiple cloning site prior to the BamHI site had been deleted. Shuffle T7 Express *E. coli* pRARE2 (New England Biolabs) were transformed with the resulting plasmid. An overnight culture of the strain in LB media was diluted 400-fold into autoinduction media (Formedium) supplemented with kanamycin (50 mg/L), the culture was grown at 37 °C for 2 h, before transfer to 18 °C for 44 h. Cells were harvested at 10000 g for 10 min, resuspended in lysis buffer (100 mM Tris-HCl, pH 7.5, 100 mM NaCl, 40 mM imidazole, 2 units/mL DNase, 1 Complete EDTA-free protease inhibitor mixture tablet) and lysed using a Constant Systems cell disrupter (20 kpsi). Cell lysates were cleared by centrifugation at 30,000 g for 45 min before purification by nickel affinity chromatography, washing with 50 mM imidazole before elution with imidazole (500 mM). Protein was dialysed into 100 mM Tris-HCl, 100 mM NaCl and stored at 4 °C either as a solution (ca. 150 μM) or an ammonium sulfate precipitate (3.2 M NH_4_SO_4_).

### Enzymatic synthesis: general procedures

#### General procedure for the synthesis of the I-Tagged GlcNAc acceptors

ITagged GlcNAc acceptors were synthesised in the following way: Azido GlcNAc (10 mM), Alkyne ITag (10 mM), sodium ascorbate (15 mM) and copper (II) sulfate (5 mM) in H_2_O were incubated at 37 °C for 10 min before LCMS showed the reaction to be complete. The ITagged acceptors were used crude in the HRMS screening assay.

#### HRMS screening assay general procedure

GlcNAc acceptor **2a**-**2f** (R = ITag) (0.2 mM), UDP-donor derived from **3a**-**3e** (1 mM), MnCl_2_ (10 mM), Tris (pH 8.0, 50 mM), BSA (1 mg/mL), HsB4GalT1 (30 μM) in a total volume of 60 μl was incubated at 37 °C for 16 h. The samples for LacNAc conversion were then frozen until analysis by Electrospray Mass Spectrometry (ESMS). Crude LacNAc derivatives (Fig. 2b; R = ITag) (0.1 mM), Tris (pH 8.0, 100 mM), MgCl_2_ (10 mM), ATP (4 mM), GTP (2 mM), monosaccharide **4a**-**4e** (2 mM), HpFucT (0.59 mg/mL), BfFKP (0.78 mg/mL) in a total volume of 10 μl was incubated for 16 h at 37 °C. The samples were then frozen. The samples were analysed by HRMS through direct injection of the crude reaction mixture. The percentage conversion for each reaction featuring ITag-label substrates was calculated automatically by comparison of the integration of the ITag-labelled starting material and product ESMS peaks using Bruker DataAnalysis 4.3. A replicate of n = 1 was used for this study.

#### In situ one-pot synthesis of sugar nucleotides

Fluorinated/deoxy UDP-Gal analogues were synthesised using a one-pot multienzyme system. Briefly, the Gal analogue (8 mM), ATP (10 mM), UTP (10 mM), MgCl_2_ (10 mM), Tris buffer (50 mM, pH 8.0), SpGalK (300 μg/mL), BlUSP (75 μg/mL), EcGalPU (32.5 μg/mL) and pyrophosphatase (50 U/mL) was incubated at 37 °C overnight as described previously (Fig. 2)^35^. GDP-fucose donors were generated in situ from monosaccharides **4a**-**4e** by a bifunctional l-fucokinase/l-fucose-1-P guanylyltransferase from *Bacteroides fragilis* (BfFKP)^44^.

#### One pot, preparative scale fluorinated Lewis^x^ glycan synthesis

Reactions were performed following the one-pot, two-step enzymatic synthesis protocol, on azidopropyl GlcNAc analogues **2a-2f**. In general, the concentration of the acceptors was increased from the 0.1–0.2 mM that was used in the screening experiments to 10–20 mM in the scale up (variations in enzyme and substrate concentrations depending on the efficiency of reaction are detailed in the SI). The molar equivalents of enzyme were also reduced by a factor of 10 compared to the galactosylation screening. GlcNAc derivative **2a**-**2f** (R = azidopropyl) (10 mM), UDP-Gal derived from **3a**-**3e** (11 mM), MnCl_2_ (10 mM), BSA (1 mg/mL), HsB4GalT1 (30 μM) in Tris (100 mM, pH 8.0) in a total volume of 3.25 mL were incubated overnight at 37 °C. The fucosylation reaction components were added to achieve the following final concentrations: Tris, pH 7.0 (200 mM), MgCl_2_ (10 mM), ATP (16 mM), GTP (8 mM), Fuc derivative **4a**-**4e** (8 mM), HpFucT (8 μM) and BfFKP (5 μM) in a total volume of 8.12 mL, and the mixture was incubated overnight at 37 °C. The reaction mixture was passed through a 10 kDa molecular weight cut off spin concentrator to remove proteins and the filtrate was concentrated to dryness. The product was purified by silica gel chromatography (20:80 → 50:50 MeOH:EtOAc), followed by size exclusion chromatography using a Biogel P2 column equilibrated with ammonium formate (20 mM) in water.

### Glycan microarrays

#### Materials

1,2-Dihexadecyl-*sn*-glycero-3-phosphoethanolamine (DHPE) was purchased from Fluka (Dorset, UK). Dibenzocyclooctyne-*N*-hydroxysuccinimidyl ester (DBCO-NHS) was purchased from Conju-Probe, LLC (California, USA). *N,N*-Diisopropylethylamine (DIPEA), high performance thin layer chromatography (HPTLC) plates (60 F254) were from MERCK. Solvents, with HPLC grade purity, were purchased from Fisher scientific (Massachusetts, USA). H_2_O was purified with a Milli‐Q purification system from Millipore (18.3 MΩ‐cm). Sep-Pak silica cartridges (500 mg) were from Waters (Milford, US).

#### Synthesis of the new lipid reagent

Dibenzocyclooctyne-*N*-1,2-dihexadecyl-*sn*-glycero-3-phosphoethanolamine (DBCO-DH): The dibenzocyclooctyne (DBCO) functionality was incorporated into the amino-phospholipid DHPE via amide coupling of DBCO-NHS and DHPE (Supplementary Fig. 1). DBCO-NHS (8.0 mg, 20 μmol) with DIPEA (7 μL, 40 μmol) were dissolved in CHCl_3_ (1 mL) and added dropwise to a solution of DHPE (6.6 mg, 10 μmol) in CHCl_3_:MeOH 2:1 (3 mL). The reaction mixture was stirred at room temperature for 21 h. HPTLC analysis of the mixture in solvent CHCl_3_:MeOH:NH_3_ 100:30:1 visualised under longwave UV light after primulin staining^76^, showed the complete conversion of DHPE to DBCO-DH (Rf 0.63). The product was purified by preparative-HPTLC (CHCl_3_:MeOH:H_2_O 100:30:1) with a yield of 91% (8.6 mg). MALDI-MS analysis of the purified DBCO-DH gave a [M-H]- ion at m/z 949.8 as predicted (C_56_H_91_N_2_O_8_P, calculated 950.6513 [M]). HR-ESI-MS, m/z: 949.6464 ([M-H]-, calc. 949.6435).

#### General procedure for the preparation of DBCO-DH NGLs

A total of 24 azidopropyl Lewis^x^ trisaccharide analogues were converted into DBCO-DH NGLs via micro-scale SPAAC reaction (Supplementary Fig. 2). with DBCO-DH in quantitative yields using the general procedure described below. To a solution of DBCO-DH (400 nmol) in CHCl_3_:MeOH 1:1 (200 μL), a solution of 3-azido propyl glycan (100 nmol) in water (30 μL) was added. The reaction mixture was shaken at room temperature for 4 h. HPTLC analysis of the mixture (1 nmol of glycan starting material) in solvent CHCl_3_:MeOH:H_2_O 130:50:9 visualised under longwave UV light after primulin staining^76^ showed the formation Glycan-DBCO-DH NGL, as a mixture of regioisomers (Supplementary Fig. 12) with an efficiency greater than 90%. The products were purified by semi-preparative HPTLC (CHCl_3_:MeOH:H_2_O 60:25:4) or Silica cartridge using established conditions for conventional NGLs^76^. The purified NGLs were analysed by HPTLC (Supplementary Fig. 12) and MALDI-MS. The molecular masses detected were in accord with theoretical values (Supplementary Table 5). The 24 DBCO-DH NGLs were quantified using established protocol as described for conventional DH-NGLs^77^. The quantified NGL stock solutions were stored at −20 °C in CHCl_3_:MeOH:H_2_O 25:25:8.

For analysis of the NGL probes, matrix-assisted laser desorption/ionisation mass spectrometry (MALDI-MS) was carried out on Shimadzu AXIMA Assurance Resonance instrument with a QIT-TOF configuration (Shimadzu, Milton Keynes, England). The NGLs were dissolved in CHCl3:MeOH:H2O 25:25:8 at a concentration of ~10 pmol/μL, and 0.5 μL was deposited on the sample target together with a matrix solution of 2′,4′,6′-trihydroxyacetophenone in MeOH (1 μg/μL) for analysis. A nitrogen laser was used to irradiate the samples at 337 nm with a laser energy at 100% (coarse) and 50% (fine); resolution was at 1000. For high resolution MS, electrospray (ESI-MS) was used at a resolution of 10,000 on a Waters Synapt G2-S instrument (Waters, Wilmslow, England). Samples (1 μL) were introduced by flow injection and cone voltage was set at 80 V.

#### Glycan microarray screening analyses

Microarray analyses were carried out using the NGL-based microarray system^78^. Details of the glycan probe library, the generation of the microarrays, the glycan binding protein samples and detection systems and assay protocol used in the microarray binding experiments, imaging, and data analysis are in Supplementary Table 3 in accordance with the Minimum Information Required for A Glycomics Experiment (MIRAGE) guidelines for reporting glycan microarray-based data^77^. These analyses serve to measure the relative strengths of interaction with the proteins (relative avidities) rather than binding affinities. Microarray data at the protein concentration with the optimal signals for the Lewis^x^ glycan library are presented to demonstrate that differing binding patterns are obtained for different Lewis^x^ binding proteins.

### Gold nanoparticle platform

#### Materials

*N-*Hydroxyethyl acrylamide (97%), 4,4′-azobis(4-cyanovaleric acid) (98%), mesitylene (reagent grade), triethylamine ( > 99%), sodium citrate tribasic dihydrate ( > 99%), gold(III) chloride trihydrate (99.9%), dibenzocyclooctyne-amine and cholera toxin B subunit were all purchased from Sigma-Aldrich and used without further purification. 2-(dodecylthiocarbonothioylthio)-2-methylpropionic acid pentafluorophenyl ester was synthesised as previously outlined by Richards et al.^25^. HEPES buffer with 0.15 M NaCl, 0.1 mM CaCl_2_ and 0.01 mM MnCl_2_ (pH 7.4) was used for the aggregation studies.

#### Polymerisation of hydroxyethyl acrylamide using PFP-DMP

Polymerisation of hydroxyethyl acrylamide using PFP-DMP as a chain transfer agent was carried out as previously outlined^25,71^. Monomer *N-*hydroxyethyl acrylamide (HEA) (0.5 g, 4.34 mmol), chain transfer agent pentafluorophenyl 2-(dodecylthiocarbonothioylthio)-2-methylpropionic acid (PFP-DMP) (0.092 g, 0.17 mmol), and intiator 4,4′-azobis(4-cyanovaleric acid) (ACVA) (0.0097 g, 0.034 mmol were dissolved in 1:1 mixture of toluene and methanol (4 mL). Mesitylene (150 μL) was added as an internal reference for NMR. A 25 μL aliquot was taken for ^1^H NMR analysis of the conversion in CDCl_3_. The solution was degassed under a stream N_2_ for 30 mins. The reaction was stirred at 70 °C for 90 mins. Another 25 μL aliquot was taken for the NMR analysis of conversion in MeOD. The reaction was quenched in liquid nitrogen and precipitated into diethyl ether (50 mL). The polymer was reprecipitated into diethyl ether from methanol twice more to yield a yellow polymer product that was dried under vacuum. 96% conversion by NMR, M_n_ (Theoretical) = 3400 g.mol^−1^ M_n_ (SEC) = 5800 g.mol^−1^ M_n_/M_w_ (SEC) = 1.16. (SEC = Size Exclusion Chromatography).

#### Gold nanoparticle synthesis

55 nm gold nanoparticles were synthesised by a modified step growth method developed by Bastús et al.^79^ as outlined previously^25^. A seed solution was made as follows: 150 mL of 2.2 mM sodium citrate in MilliQ water was heated under reflux for 15 min with vigorous stirring. Once the solution as boiling, 1 mL of HAuCl_4_ (25 mM) was added. The colour of the solution changed from yellow to blue-ish grey initially and then to pale pink within 10 min, 1 mL was taken for DLS (Dynamic Light Scattering) and UV-Vis analysis. This Au seed solution was cooled to 90 °C. Once cooled, 1 mL HAuCl_4_ (25 mM) was added. After stirring for 20 min, two further additions of 1 mL HAuCl_4_ (25 mM) were added with 20 min between each addition. 1 mL was taken for DLS and UV/Vis analysis. Following this, the sample was diluted by adding 85 mL of MilliQ water and 3.1 mL of 60 mM sodium citrate was added. This solution was then used as a seed solution, and three further portions of 1.6 mL of 25 mM HAuCl_4_ were added with 20 min between each addition. Following completion of this step 1 mL was taken for DLS and UV/Vis analysis. The sample was diluted by adding 135 mL of MilliQ water and 4.9 mL of 60 mM sodium citrate was added. This solution was then used as a seed solution, and the process was repeated with three further additions of 2.5 mL of 25 mM HAuCl_4_ with 20 min waiting period between addition. Following completion of this step 1 mL was taken for DLS and UV/Vis analysis. The sample was diluted by adding 215 mL of MilliQ water and 7.8 mL of 60 mM sodium citrate was added. This solution was then used as a seed solution, and the process was repeated with three further additions of 3.9 mL of 25 mM HAuCl_4_ with 20 min between additions, this solution was analysed by DLS and UV/Vis and target size of 55 nm was reached, and the solution was cooled. AuNPs had an OD (optical density) of 4.16 @ SPR_max_ = 533 nm. This solution was stored in the dark and used without further purification.

#### Functionalisation of PHEA with DBCO

PFP-PHEA (500 mg, 0.15 mmol), dibenzocylclooctyne-amine (DBCO) (81 mg, 0.29 mmol) were dissolved in 2 mL dimethyl formamide (DMF). The reaction was stirred at room temperature for 16 h. The polymer was precipitated into diethyl ether from methanol three times and dried under vacuum. The resulting polymer was an off white solid. IR indicated loss of C = O stretch corresponding to the PFP ester. ^19^F NMR also indicated the removal of the PFP ester.

#### Capture of Le^x^ derivatives onto DBCO-PHEA

In a typical reaction, DBCO-PHEA (1 mg, 0.32 μmol) and azidopropyl-linked glycan (2 equiv.) was dissolved in 1 mL MilliQ water and left to react overnight on a tube roller. The solution was used immediately for immobilisation onto AuNPs.

#### Gold nanoparticle functionalisation using Le^x^-functionalised PHEAs

100 μL of 1 mg.mL^−1^ of polymer solution was added to 1 mL of ~OD_533_ 4 particles and left for 30 min at room temperature on a tube roller. After 30 min, particles were centrifuged at 6000 rpm (3381 g), the resulting supernatant was removed, and the particles were resuspended in 1 mL MilliQ H_2_O. This was repeated a further two times to ensure complete removal of any unattached polymer. Stability was confirmed by incubating in 10 mM HEPES buffer for 30 min and measuring UV-Vis and comparing to AuNPs in MilliQ H_2_O. Particles were diluted to OD_max_ 1 for testing against CTB.

#### Nanoparticle characterisation

Particles were characterised by UV-Vis (Supplementary Fig. 13) and DLS (Supplementary Fig. 14), the analyses of these are summarised in Supplementary Table 6.

#### Absence of aggregation was confirmed with TEM analysis (Supplementary Figs. 15)

AuNPs (OD_540_ = 1) were drop cast on Holey Carbon Film 300 mesh copper grids (Agar Scientific). TEM images were obtained with a Thermo Fisher Scientific—Talos F200X microscope equipped with an X-FEG electron source. The experiment was performed using an acceleration voltage of 200 kV and a beam current of approximately 5 nA. Images were recorded with a Thermo Scientific—CETA 4k x 4k CMOS camera. Analysis of particle diameter was conducted in Image J2 Version 2.14.0/1.54 f.

#### Cholera toxin-induced aggregation studies by Absorbance

A stock solution of CTB was made up 0.2 mg.mL^−1^ in 10 mM HEPES buffer with 0.15 M NaCl, 0.1 mM CaCl_2_ and 0.01 mM MnCl_2_. 25 μL 2-fold serial dilution was made up in the same buffer in a clear, flat bottom, half-area 96-well microtitre plate (7 dilutions and a buffer control). 25 μL of the glycoAuNP were added to each well and incubated at room temperature for 30 min. After 30 min, an absorbance spectrum was recorded from 450 nm -700 nm with 10 nm intervals (Supplementary Fig. 16). **LeX8**-, **LeX11**-, **LeX16**- and **LeX24**-funtionalised AuNPs required further serial dilutions to realise the full isotherm. Three repeats for each Le^x^-derivative-functionalised AuNPs were carried out and average binding isotherms were plotted in origin and EC_50_ were derived from the Hill1 function (Eq. 1).

1 $${{{\rm{y}}}}={{{\rm{START}}}}+({{{\rm{END}}}}-{{{\rm{START}}}})\frac{{{{{\rm{x}}}}}^{{{{\rm{n}}}}}}{{{{{\rm{k}}}}}^{{{{\rm{n}}}}}+{{{{\rm{x}}}}}^{{{{\rm{n}}}}}}$$

### Lateral flow strip production and running

#### Protocol for manufacturing lateral flow dipsticks

Dipsticks were made using the same procedure as outlined by Baker et al.^80^. Briefly, 20 mm of the backing cards (part for the sample pad) were removed using a guillotine. Nitrocellulose was added to the backing card using the self-adhesive on the card. The wick material was then added to the backing card with an overlap with the nitrocellulose of ~5 mm. The lateral flow strips were cut to size of width 2–3 mm using a guillotine. A small “v” ( ~ 3 mm) was cut into the test strips at the non-wick end to aid in fluid flow.

#### Protocol for test line addition to the lateral flow dipsticks

1 µL of 10 mg.mL^−1^ GM1 was applied to the dipstick ~1 cm from the non-wick end of the strip. The strips were dried at 50 °C in an oven for 5 min. The tests strips were allowed to cool to room temperature before testing.

#### Lateral flow assay buffer

10× HEPES buffer (20% PVP400) in 100 mL H_2_O. 2.38 g (100 mmol.dm^−3^) of HEPES, 8.77 g (1.50 mol.dm^−3^) of NaCl, 0.011 g (1.0 mmol.dm^−3^) of CaCl2, 0.8 g (0.8% w/v., 123 mmol.dm^−3^) of NaN_3_, 0.5 g (0.5% w/v., 4.07 mmol.dm^−3^) of Tween-20 and 20 g (20% w/v.) of poly(vinyl pyrrolidone)_40_ (PVP_40_, Average Mw ~40,000) were dissolved in 100 mL of water. The buffer was not pH adjusted.

#### Protocol for running lateral flow test

5 µL Le^x^-functionalised AuNPs (OD10), 5 µL lateral flow assay buffer – 10 × HEPES buffer, 5 µL 10 × desired concentration of CTB (or an extra 5 µL water for negative control) and 35 µL of H_2_O in a well of a half area 96 well plate. The strips were added to the wells, one test per well. All tests were run in triplicate. The tests were run for 20 min before removal from the wells. The test strips were allowed to dry at room temperature for ~5 min. The test strips were mounted test-face down onto a clear and colourless piece of acetate sheeting and scanned.

### Reporting summary

Further information on research design is available in the Nature Portfolio Reporting Summary linked to this article.

## Supplementary information

Supplementary Information

Peer Review File

Reporting Summary

## Source data

Source Data

## Data Availability

Supplementary information (Supplementary Figs., tables, supplementary discussion about bacterial toxin-Lewis glycan complexes, detailed experimental procedures for ITag, Lewis^x^ and glycofluoroform synthesis, copies of HRMS screening assays of both LacNAc and Lewis^x^ synthesis, NMR spectra of the Lewis^x^ and the glycofluoroforms, and MALDI-MS spectra of the 24 Lewis^x^ NGLs). Source data files of NMR spectroscopy and mass spectrometry data associated with glycan systhesis are available through the Leeds Research Data Repository (10.5518/1412). The glycan array datasets will be deposited and shared via the GlyGen Glycan Array Repository currently under its final testing phase as part of the NIH-funded GlyGen initiative (https://www.glygen.org/). As of now there is no publicly accessible glycan array data repository. The GlyGen Glycan Array Data Repository is undergoing its final testing phase as part of the NIH-funded GlyGen initiative https://glygen.ccrc.uga.edu/array/. Once the repository is officially launched, we will share our data through our Facility web portal (https://glycosciences.med.ic.ac.uk/data.html). In the interim, the Source data of microarray analyses (quantified raw fluorescence intensities) are provided as a Source Data file. The gold nanoparticle binding data generated in this study have been deposited in the University of Manchester Figshare database https://figshare.manchester.ac.uk. Correspondence and requests for materials should be addressed to Bruno Linclau. Source data are provided with this paper.
