# Supplementary material for: Synthesis and screening of a library of Lewisx deoxyfluoro-analogues reveals differential recognition by glycan-binding partners
Source: Nat Commun. 2024 Sep 13;15:7925. doi: 10.1038/s41467-024-51081-7 (PMC11399408; doi:10.1038/s41467-024-51081-7)
Supplement: Supplementary file 1 — Supplementary Information [file 41467_2024_51081_MOESM1_ESM.pdf]

Supplementary Information

Synthesis and screening of a library of Lewis^x deoxyfluoro-analogues reveals differential recognition by glycan-binding partners

Kristian Hollingsworth,[‡1] Antonio Di Maio,[‡2] Sarah-Jane Richards,[3,4] Jean-Baptiste Vendeville,[5] David E. Wheatley,[5] Claire E. Council,[5] Tessa Keenan,[6] Helene Ledru,[7] Harriet Chidwick,[6] Kun Huang,[4] Fabio Parmeggiani,[4] Andrea Marchesi,[4] Wengang Chai,[2] Ryan McBerney,[1] Tomasz P. Kamiński,[1] Matthew R. Balmforth,[1] Alexandra Tamasanu,[1] James D. Finnigan,[8] Carl Young,[8] Stuart L. Warriner,[1] Michael E. Webb,[1] Martin A. Fascione,[6] Sabine Flitsch,[4] M. Carmen Galan,[7] Ten Feizi,*[2] Matthew I. Gibson,*[3,4,9] Yan Liu,*[2] W. Bruce Turnbull,*[1] Bruno Linclau*[5,10]

‡ Equal contribution

[1] School of Chemistry and Astbury Centre for Structural Molecular Biology, University of Leeds, Leeds, LS2 9JT, UK.

[2] Department of Metabolism, Digestion and Reproduction, Glycosciences Laboratory, Imperial College London, Du Cane Road, London W12 0NN, UK.

[3] Department of Chemistry, University of Warwick, CV4 7AL, UK.

[4] Manchester Institute of Biotechnology (MIB), Department of Chemistry, University of Manchester, 131 Princess Street, Manchester M1 7DN, UK.

[5] School of Chemistry, University of Southampton, Highfield, Southampton SO17 1BJ, UK.

[6] Department of Chemistry, University of York, Heslington, York YO10 5DD, UK.

[7] School of Chemistry, Cantock's Close, University of Bristol, Bristol, BS8 1TS, UK.

[8] Prozomix Limited, Haltwhistle Industrial Estate, Haltwhistle, Northumberland NE49 9HA, UK.

[9] Division of Biomedical Sciences, Warwick Medical School, University of Warwick, CV4 7AL, UK.

[10] Department of Organic and Macromolecular Chemistry, Ghent University, Campus Sterre, Krijgslaan 281-S4, 9000 Ghent, Belgium.

t.feizi@imperial.ac.uk, M.I.Gibson@warwick.ac.uk, yan.liu2@imperial.ac.uk, W.B.Turnbull@leeds.ac.uk, Bruno.linclau@ugent.be

Table of Contents

1 Supplementary methods ... 13

1.1 ITag synthesis ... 13

1.1.1 1-(4-Carboxybenzyl)-3-methyl-1*H*-imidazol-3-ium ... 13

1.1.2 1-Methyl-3-((4-[(prop-2-yn-1-yl)carbamoyl]phenyl)methyl)imidazol-1-ium ... 13

1.2 Lewis^x synthesis ... 13

1.2.1 Gal β1-4 (Fuc α1-3) GlcNAc-N_3 (**LeX1**) ... 13

1.2.2 Gal β1-4 (Fuc α1-3) 6F-GlcNAc-N_3 (**LeX2**) ... 14

1.2.3 Gal β1-4 (Fuc α1-3) 6,6-diFGlcNAc-N_3 (**LeX3**) ... 15

1.2.4 Gal β1-4 (Fuc α1-3) GlcNTFA-N_3 (**LeX4**) ... 15

1.2.5 Gal β1-4 (Fuc α1-3) 6F-GlcNTFA-N_3 (**LeX5**) ... 16

1.2.6 Gal β1-4 (Fuc α1-3) 6,6-diFGlcNTFA-N_3 (**LeX6**) ... 16

1.2.7 3F-Gal β1-4 (Fuc α1-3) GlcNAc-N3 (**LeX7**) ... 17

1.2.8 4F-Gal β1-4 (Fuc α1-3) GlcNAc-N_3 (**LeX8**) ... 18

1.2.9 6F-Gal β1-4 (Fuc α1-3) GlcNAc-N_3 (**LeX9**) ... 18

1.2.10 6d-Gal β1-4 (Fuc α1-3) GlcNAc-N_3 (**LeX10**) ... 19

1.2.11 Gal β1-4 (3F-Fuc α1-3) GlcNAc-N_3 (**LeX11**) ... 20

1.2.12 Gal β1-4 (4F-Fuc α1-3) GlcNAc-N_3 (**LeX12**) ... 20

1.2.13 Gal β1-4 (6F-Fuc α1-3) GlcNAc-N_3 (**LeX13**) ... 21

1.2.14 Gal β1-4 (Ara α1-3) GlcNAc-N_3 (**LeX14**) ... 22

1.2.15 3F-Gal β1-4 (6F-Fuc α1-3) GlcNAc-N3 (**LeX15**) ... 22

1.2.16 4F-Gal β1-4 (6F-Fuc α1-3) GlcNAc-N_3 (**LeX16**) ... 23

1.2.17 6F-Gal β1-4 (Fuc α1-3) 6F-GlcNAc-N_3 (**LeX17**) ... 23

1.2.18 Gal β1-4 (3F-Fuc α1-3) 6F-GlcNTFA-N_3 (**LeX18**) ... 24

1.2.19 Gal β1-4 (3F-Fuc α1-3) 6,6-diFGlcNTFA-N_3 (**LeX19**) ... 25

1.2.20 Gal β1-4 (4F-Fuc α1-3) 6F-GlcNTFA-N_3 (**LeX20**) ... 26

1.2.21 Gal β1-4 (6F-Fuc α1-3) 6F-GlcNTFA-N_3 (**LeX21**) ... 26

1.2.22 4F-Gal β1-4 (Fuc α1-3) 6F-GlcNTFA-N_3 (**LeX22**) ... 27

1.2.23 6d-Gal β1-4 (Fuc α1-3) 6,6-diGlcNTFA-N_3 (**LeX23**) ... 28

1.2.24 4F-Gal β1-4 (6F-Fuc α1-3) GlcNTFA-N_3 (Le**X24**) ... 28

2 Supplementary Figures ... 30

2.1 Supplementary Figure 1. Synthesis of DBCO-DH. ... 30

2.2 Supplementary Figure 2. Synthesis of DBCO-DH NGLs via SPAAC 'click' reaction. ... 30

2.3 Supplementary Figure 3. Crystal structure of β(1,4)-galactosyltransferase bound to UDP-Gal 31

2.4 Supplementary Figure 4. Crystal structure of Hp-α1-3FucT bound to GDP-Fuc ... 31

2.5 Supplementary Figure 5. Increased chemical shift of fucose H-5 ... 32

2.6 Supplementary Figure 6. Further NMR-evidence of the glycofluoroform closed conformation for 6F-Fuc glycofluoroforms ... 32

2.7 Supplementary Figure 7. Histogram chart representation of the results of binding of hDC-SIGNR, Rhesus Langerin and anti-Lewis^x (BG-7) to the NGL probes in the microarray set tested. ... 34

2.8 Supplementary Figure 8. Histogram charts showing the influence of fluorination of the Lewis^x trisaccharide on the binding signals with hDC-SIGN, Classical CTB, LTBh, and anti-Lewis^x antibodies anti-L5, anti-SSEA-1 and anti-BG7. ... 35

2.9 Supplementary Figure 9. 'Fingerprints' of the binding of the Classical CTB, LTBh, hDC-SIGN, and anti-Lewis^X antibodies anti-L5, anti-SSEA-1 and BG-7 showing effects of fluorination of the Lewis^X trisaccharide. ... 37

2.10 Supplementary Figure 10. Individual spider charts showing the influence of fluorination of the Lewis^X trisaccharide on the binding signal intensities of hDC-SIGN, the Classical CTB and LTBh, and the anti-Lewis^X antibodies anti-L5, anti-SSEA-1 and BG-7. ... 38

2.11 Supplementary Figure 11. Isothermal Titration Calorimetry of (a) Classical and (b) El Tor CTB with Lewis^x ... 39

2.12 Supplementary Figure 12. HPTLC analyses of the 24 NGLs prepared from the azido-terminated Lewis^x structures ... 40

2.13 Supplementary Figure 13. UV-Vis analysis of unfunctionalised ($AuNP_{55}$ – black dotted line) and functionalised AuNPs used in this study *40*
2.14 Supplementary Figure 14 Dynamic Light Scattering analysis of unfunctionalised ($AuNP_{55}$ – black dotted line) and functionalised AuNPs used in this study *41*
2.15 Supplementary Figure 15: TEM-analysis of unfunctionalised and functionalised AuNPs used in this study *42*
2.16 Supplementary Figure 16. UV-Vis analysis of CTB-induced Aggregation *44*
2.17 Supplementary Figure 17a-c. Negative and positive controls for the aggregation assay. *45*
2.18 Supplementary figures 18-197. HRMS ITag screening assay mass spectra of both LacNAc and Lewis^{X} synthesis *47*
2.18.1 Supplementary Figure 18. HRMS ITag screening assay mass spectrum of synthesis of Gal β1-4 GlcNAc-ITag 47
2.18.2 Supplementary Figure 19. HRMS ITag screening assay mass spectrum of synthesis of Gal β1-4 GlcNTFA-ITag 48
2.18.3 Supplementary Figure 20. HRMS ITag screening assay mass spectrum of synthesis of Gal β1-4 6F-GlcNAc-ITag 49
2.18.4 Supplementary Figure 21. HRMS ITag screening assay mass spectrum of synthesis of Gal β1-4 6F-GlcNTFA-ITag 50
2.18.5 Supplementary Figure 22. HRMS ITag screening assay mass spectrum of synthesis of Gal β1-4 6,6-diFGlcNAc-ITag 51
2.18.6 Supplementary Figure 23. HRMS ITag screening assay mass spectrum of synthesis of Gal β1-4 6,6-diFGlcNTFA-ITag 52
2.18.7 Supplementary Figure 24. HRMS ITag screening assay mass spectrum of synthesis of 3F-Gal β1-4 GlcNAc-ITag 53
2.18.8 Supplementary Figure 25. HRMS ITag screening assay mass spectrum of synthesis of 3F-Gal β1-4 GlcNTFA-ITag 54
2.18.9 Supplementary Figure 26. HRMS ITag screening assay mass spectrum of synthesis of 3F-Gal β1-4 6F-GlcNAc-ITag 55
2.18.10 Supplementary Figure 27. HRMS ITag screening assay mass spectrum of synthesis of 3F-Gal β1-4 6F-GlcNTFA-ITag 56
2.18.11 Supplementary Figure 28. HRMS ITag screening assay mass spectrum of synthesis of 3F-Gal β1-4 6,6-diFGlcNAc-ITag 57
2.18.12 Supplementary Figure 29. HRMS ITag screening assay mass spectrum of synthesis of 3F-Gal β1-4 6,6-diFGlcNTFA-ITag 58
2.18.13 Supplementary Figure 30. HRMS ITag screening assay mass spectrum of synthesis of 4F-Gal β1-4 GlcNAc-ITag 59
2.18.14 Supplementary Figure 31. HRMS ITag screening assay mass spectrum of synthesis of 4F-Gal β1-4 GlcNTFA-ITag 60
2.18.15 Supplementary Figure 32. HRMS ITag screening assay mass spectrum of synthesis of 4F-Gal β1-4 6F-GlcNAc-ITag 61
2.18.16 Supplementary Figure 33. HRMS ITag screening assay mass spectrum of synthesis of 4F-Gal β1-4 6F-GlcNTFA-ITag 62
2.18.17 Supplementary Figure 34. HRMS ITag screening assay mass spectrum of synthesis of 4F-Gal β1-4 6,6-diFGlcNAc-ITag 63
2.18.18 Supplementary Figure 35. HRMS ITag screening assay mass spectrum of synthesis of 4F-Gal β1-4 6,6-diFGlcNTFA-ITag 64
2.18.19 Supplementary Figure 36. HRMS ITag screening assay mass spectrum of synthesis of 6F-Gal β1-4 GlcNAc-ITag 65
2.18.20 Supplementary Figure 37. HRMS ITag screening assay mass spectrum of synthesis of 6F-Gal β1-4 GlcNTFA-ITag 66
2.18.21 Supplementary Figure 38. HRMS ITag screening assay mass spectrum of synthesis of 6F-Gal β1-4 6F-GlcNAc-ITag 67
2.18.22 Supplementary Figure 39. HRMS ITag screening assay mass spectrum of synthesis of 6F-Gal β1-4 6F-GlcNTFA-ITag 68
2.18.23 Supplementary Figure 40. HRMS ITag screening assay mass spectrum of synthesis of 6F-Gal β1-4 6,6-diFGlcNAc-ITag 69

2.18.24 Supplementary Figure 41. HRMS ITag screening assay mass spectrum of synthesis of 6F-Gal β1-4 6,6-diFGlcNTFA-ITag........ 70
2.18.25 Supplementary Figure 42. HRMS ITag screening assay mass spectrum of synthesis of 6d-Gal β1-4 GlcNAc-ITag........ 71
2.18.26 Supplementary Figure 43. HRMS ITag screening assay mass spectrum of synthesis of 6d-Gal β1-4 GlcNTFA-ITag........ 72
2.18.27 Supplementary Figure 44. HRMS ITag screening assay mass spectrum of synthesis of 6d-Gal β1-4 6F-GlcNAc-ITag........ 73
2.18.28 Supplementary Figure 45. HRMS ITag screening assay mass spectrum of synthesis of 6d-Gal β1-4 6F-GlcNTFA-ITag........ 74
2.18.29 Supplementary Figure 46. HRMS ITag screening assay mass spectrum of synthesis of 6d-Gal β1-4 6,6-diFGlcNAc-ITag........ 75
2.18.30 Supplementary Figure 47. HRMS ITag screening assay mass spectrum of synthesis of 6d-Gal β1-4 6,6-diFGlcNTFA-ITag........ 76
2.18.31 Supplementary Figure 48. HRMS ITag screening assay mass spectrum of synthesis of Gal β1-4 (Fuc α1-3) GlcNAc-ITag (LeX1)........ 77
2.18.32 Supplementary Figure 49. HRMS ITag screening assay mass spectrum of synthesis of Gal β1-4 (Fuc α1-3) GlcNTFA-ITag (LeX4)........ 77
2.18.33 Supplementary Figure 50. HRMS ITag screening assay mass spectrum of synthesis of Gal β1-4 (Fuc α1-3) 6F-GlcNAc-ITag (LeX2)........ 78
2.18.34 Supplementary Figure 51. HRMS ITag screening assay mass spectrum of synthesis of Gal β1-4 (Fuc α1-3) 6F-GlcNTFA-ITag (LeX5)........ 79
2.18.35 Supplementary Figure 52. HRMS ITag screening assay mass spectrum of synthesis of Gal β1-4 (Fuc α1-3) 6,6-diFGlcNAc-ITag (LeX3)........ 80
2.18.36 Supplementary Figure 53. HRMS ITag screening assay mass spectrum of synthesis of Gal β1-4 (Fuc α1-3) 6,6-diFGlcNTFA-ITag (LeX6)........ 81
2.18.37 Supplementary Figure 54. HRMS ITag screening assay mass spectrum of synthesis of 3F-Gal β1-4 (Fuc α1-3) GlcNAc-ITag (LeX7)........ 82
2.18.38 Supplementary Figure 55. HRMS ITag screening assay mass spectrum of synthesis of 3F-Gal β1-4 (Fuc α1-3) GlcNTFA-ITag........ 83
2.18.39 Supplementary Figure 56. HRMS ITag screening assay mass spectrum of synthesis of 3F-Gal β1-4 (Fuc α1-3) 6F-GlcNAc-ITag........ 84
2.18.40 Supplementary Figure 57. HRMS ITag screening assay mass spectrum of synthesis of 3F-Gal β1-4 (Fuc α1-3) 6F-GlcNTFA-ITag........ 85
2.18.41 Supplementary Figure 58. HRMS ITag screening assay mass spectrum of synthesis of 3F-Gal β1-4 (Fuc α1-3) 6,6-diFGlcNAc-ITag........ 86
2.18.42 Supplementary Figure 59. HRMS ITag screening assay mass spectrum of synthesis of 3F-Gal β1-4 (Fuc α1-3) 6,6-diFGlcNTFA-ITag........ 87
2.18.43 Supplementary Figure 60. HRMS ITag screening assay mass spectrum of synthesis of 4F-Gal β1-4 (Fuc α1-3) GlcNAc-ITag (LeX8)........ 88
2.18.44 Supplementary Figure 61. HRMS ITag screening assay mass spectrum of synthesis of 4F-Gal β1-4 (Fuc α1-3) GlcNTFA-ITag........ 89
2.18.45 Supplementary Figure 62. HRMS ITag screening assay mass spectrum of synthesis of 4F-Gal β1-4 (Fuc α1-3) 6F-GlcNAc-ITag........ 90
2.18.46 Supplementary Figure 63. HRMS ITag screening assay mass spectrum of synthesis of 4F-Gal β1-4 (Fuc α1-3) 6F-GlcNTFA-ITag (LeX22)........ 91
2.18.47 Supplementary Figure 64. HRMS ITag screening assay mass spectrum of synthesis of 4F-Gal β1-4 (Fuc α1-3) 6,6-diFGlcNAc-ITag........ 92
2.18.48 Supplementary Figure 65. HRMS ITag screening assay mass spectrum of synthesis of 4F-Gal β1-4 (Fuc α1-3) 6,6-diFGlcNTFA-ITag........ 93
2.18.49 Supplementary Figure 66. HRMS ITag screening assay mass spectrum of synthesis of 6F-Gal β1-4 (Fuc α1-3) GlcNAc-ITag (LeX9)........ 94
2.18.50 Supplementary Figure 67. HRMS ITag screening assay mass spectrum of synthesis of 6F-Gal β1-4 (Fuc α1-3) GlcNTFA-ITag........ 95
2.18.51 Supplementary Figure 68. HRMS ITag screening assay mass spectrum of synthesis of 6F-Gal β1-4 (Fuc α1-3) 6F-GlcNAc-ITag (LeX17)........ 96

2.18.52 Supplementary Figure 69. HRMS ITag screening assay mass spectrum of synthesis of 6F-Gal β1-4 (Fuc α1-3) 6F-GlcNTFA-ITag ... 97
2.18.53 Supplementary Figure 70. HRMS ITag screening assay mass spectrum of synthesis of 6F-Gal β1-4 (Fuc α1-3) 6,6-diFGlcNAc-ITag ... 98
2.18.54 Supplementary Figure 71. HRMS ITag screening assay mass spectrum of synthesis of 6F-Gal β1-4 (Fuc α1-3) 6,6-diFGlcNTFA-ITag ... 99
2.18.55 Supplementary Figure 72. HRMS ITag screening assay mass spectrum of synthesis of 6d-Gal β1-4 (Fuc α1-3) GlcNAc-ITag (LeX10) ... 100
2.18.56 Supplementary Figure 73. HRMS ITag screening assay mass spectrum of synthesis of 6d-Gal β1-4 (Fuc α1-3) GlcNTFA-ITag ... 101
2.18.57 Supplementary Figure 74. HRMS ITag screening assay mass spectrum of synthesis of 6d-Gal β1-4 (Fuc α1-3) 6F-GlcNAc-ITag ... 102
2.18.58 Supplementary Figure 75. HRMS ITag screening assay mass spectrum of synthesis of 6d-Gal β1-4 (Fuc α1-3) 6F-GlcNTFA-ITag ... 103
2.18.59 Supplementary Figure 76. HRMS ITag screening assay mass spectrum of synthesis of 6d-Gal β1-4 (Fuc α1-3) 6,6-diFGlcNAc-ITag ... 104
2.18.60 Supplementary Figure 77. HRMS ITag screening assay mass spectrum of synthesis of 6d-Gal β1-4 (Fuc α1-3) 6,6-diFGlcNTFA-ITag (LeX23) ... 105
2.18.61 Supplementary Figure 78. HRMS ITag screening assay mass spectrum of synthesis of Gal β1-4 (3F-Fuc α1-3) GlcNAc-ITag (LeX11) ... 106
2.18.62 Supplementary Figure 79. HRMS ITag screening assay mass spectrum of synthesis of Gal β1-4 (3F-Fuc α1-3) GlcNTFA-ITag ... 107
2.18.63 Supplementary Figure 80. HRMS ITag screening assay mass spectrum of synthesis of Gal β1-4 (3F-Fuc α1-3) 6F-GlcNAc-ITag ... 108
2.18.64 Supplementary Figure 81. HRMS ITag screening assay mass spectrum of synthesis of Gal β1-4 (3F-Fuc α1-3) 6F-GlcNTFA-ITag (LeX18) ... 109
2.18.65 Supplementary Figure 82. HRMS ITag screening assay mass spectrum of synthesis of Gal β1-4 (3F-Fuc α1-3) 6,6-diFGlcNAc-ITag ... 110
2.18.66 Supplementary Figure 83. HRMS ITag screening assay mass spectrum of synthesis of Gal β1-4 (3F-Fuc α1-3) 6,6-diFGlcNTFA-ITag (LeX19) ... 111
2.18.67 Supplementary Figure 84. HRMS ITag screening assay mass spectrum of synthesis of 3F-Gal β1-4 (3F-Fuc α1-3) GlcNAc-ITag ... 112
2.18.68 Supplementary Figure 85. HRMS ITag screening assay mass spectrum of synthesis of 3F-Gal β1-4 (3F-Fuc α1-3) GlcNTFA-ITag ... 113
2.18.69 Supplementary Figure 86. HRMS ITag screening assay mass spectrum of synthesis of 3F-Gal β1-4 (3F-Fuc α1-3) 6F-GlcNAc-ITag ... 114
2.18.70 Supplementary Figure 87. HRMS ITag screening assay mass spectrum of synthesis of 3F-Gal β1-4 (3F-Fuc α1-3) 6F-GlcNTFA-ITag ... 115
2.18.71 Supplementary Figure 88. HRMS ITag screening assay mass spectrum of synthesis of 3F-Gal β1-4 (3F-Fuc α1-3) 6,6-diFGlcNAc-ITag ... 116
2.18.72 Supplementary Figure 89. HRMS ITag screening assay mass spectrum of synthesis of 3F-Gal β1-4 (3F-Fuc α1-3) 6,6-diFGlcNTFA-ITag ... 117
2.18.73 Supplementary Figure 90. HRMS ITag screening assay mass spectrum of synthesis of 4F-Gal β1-4 (3F-Fuc α1-3) GlcNAc-ITag ... 118
2.18.74 Supplementary Figure 91. HRMS ITag screening assay mass spectrum of synthesis of 4F-Gal β1-4 (3F-Fuc α1-3) GlcNTFA-ITag ... 119
2.18.75 Supplementary Figure 92. HRMS ITag screening assay mass spectrum of synthesis of 4F-Gal β1-4 (3F-Fuc α1-3) 6F-GlcNAc-ITag ... 120
2.18.76 Supplementary Figure 93. HRMS ITag screening assay mass spectrum of synthesis of 4F-Gal β1-4 (3F-Fuc α1-3) 6F-GlcNTFA-ITag ... 121
2.18.77 Supplementary Figure 94. HRMS ITag screening assay mass spectrum of synthesis of 4F-Gal β1-4 (3F-Fuc α1-3) 6,6-diFGlcNAc-ITag ... 122
2.18.78 Supplementary Figure 95. HRMS ITag screening assay mass spectrum of synthesis of 4F-Gal β1-4 (3F-Fuc α1-3) 6,6-diFGlcNTFA-ITag ... 123
2.18.79 Supplementary Figure 96. HRMS ITag screening assay mass spectrum of synthesis of 6F-Gal β1-4 (3F-Fuc α1-3) GlcNAc-ITag ... 124

2.18.80 Supplementary Figure 97. HRMS ITag screening assay mass spectrum of synthesis of 6F-Gal β1-4 (3F-Fuc α1-3) GlcNTFA-ITag 125
2.18.81 Supplementary Figure 98. HRMS ITag screening assay mass spectrum of synthesis of 6F-Gal β1-4 (3F-Fuc α1-3) 6F-GlcNAc-ITag 126
2.18.82 Supplementary Figure 99. HRMS ITag screening assay mass spectrum of synthesis of 6F-Gal β1-4 (3F-Fuc α1-3) 6F-GlcNTFA-ITag 127
2.18.83 Supplementary Figure 100. HRMS ITag screening assay mass spectrum of synthesis of 6F-Gal β1-4 (3F-Fuc α1-3) 6,6-diFGlcNAc-ITag 128
2.18.84 Supplementary Figure 101. HRMS ITag screening assay mass spectrum of synthesis of 6F-Gal β1-4 (3F-Fuc α1-3) 6,6-diFGlcNTFA-ITag 129
2.18.85 Supplementary Figure 102. HRMS ITag screening assay mass spectrum of synthesis of 6d-Gal β1-4 (3F-Fuc α1-3) GlcNAc-ITag 130
2.18.86 Supplementary Figure 103. HRMS ITag screening assay mass spectrum of synthesis of 6d-Gal β1-4 (3F-Fuc α1-3) GlcNTFA-ITag 131
2.18.87 Supplementary Figure 104. HRMS ITag screening assay mass spectrum of synthesis of 6d-Gal β1-4 (3F-Fuc α1-3) 6F-GlcNAc-ITag 132
2.18.88 Supplementary Figure 105. HRMS ITag screening assay mass spectrum of synthesis of 6d-Gal β1-4 (3F-Fuc α1-3) 6F-GlcNTFA-ITag 133
2.18.89 Supplementary Figure 106. HRMS ITag screening assay mass spectrum of synthesis of 6d-Gal β1-4 (3F-Fuc α1-3) 6,6-diFGlcNAc-ITag 134
2.18.90 Supplementary Figure 107. HRMS ITag screening assay mass spectrum of synthesis of 6d-Gal β1-4 (3F-Fuc α1-3) 6,6-diFGlcNTFA-ITag 135
2.18.91 Supplementary Figure 108. HRMS ITag screening assay mass spectrum of synthesis of Gal β1-4 (4F-Fuc α1-3) GlcNAc-ITag (LeX12) 136
2.18.92 Supplementary Figure 109. HRMS ITag screening assay mass spectrum of synthesis of Gal β1-4 (4F-Fuc α1-3) GlcNTFA-ITag 137
2.18.93 Supplementary Figure 110. HRMS ITag screening assay mass spectrum of synthesis of Gal β1-4 (4F-Fuc α1-3) 6F-GlcNAc-ITag 138
2.18.94 Supplementary Figure 111. HRMS ITag screening assay mass spectrum of synthesis of Gal β1-4 (4F-Fuc α1-3) 6F-GlcNTFA-ITag (LeX20) 139
2.18.95 Supplementary Figure 112. HRMS ITag screening assay mass spectrum of synthesis of Gal β1-4 (4F-Fuc α1-3) 6,6-diFGlcNAc-ITag 140
2.18.96 Supplementary Figure 113. HRMS ITag screening assay mass spectrum of synthesis of Gal β1-4 (4F-Fuc α1-3) 6,6-diFGlcNTFA-ITag 141
2.18.97 Supplementary Figure 114. HRMS ITag screening assay mass spectrum of synthesis of 3F-Gal β1-4 (4F-Fuc α1-3) GlcNAc-ITag 142
2.18.98 Supplementary Figure 115. HRMS ITag screening assay mass spectrum of synthesis of 3F-Gal β1-4 (4F-Fuc α1-3) GlcNTFA-ITag 143
2.18.99 Supplementary Figure 116. HRMS ITag screening assay mass spectrum of synthesis of 3F-Gal β1-4 (4F-Fuc α1-3) 6F-GlcNAc-ITag 144
2.18.100 Supplementary Figure 117. HRMS ITag screening assay mass spectrum of synthesis of 3F-Gal β1-4 (4F-Fuc α1-3) 6F-GlcNTFA-ITag 145
2.18.101 Supplementary Figure 118. HRMS ITag screening assay mass spectrum of synthesis of 3F-Gal β1-4 (4F-Fuc α1-3) 6,6-diFGlcNAc-ITag 146
2.18.102 Supplementary Figure 119. HRMS ITag screening assay mass spectrum of synthesis of 3F-Gal β1-4 (4F-Fuc α1-3) 6,6-diFGlcNTFA-ITag 147
2.18.103 Supplementary Figure 120. HRMS ITag screening assay mass spectrum of synthesis of 4F-Gal β1-4 (4F-Fuc α1-3) GlcNAc-ITag 148
2.18.104 Supplementary Figure 121. HRMS ITag screening assay mass spectrum of synthesis of 4F-Gal β1-4 (4F-Fuc α1-3) GlcNTFA-ITag 149
2.18.105 Supplementary Figure 122. HRMS ITag screening assay mass spectrum of synthesis of 4F-Gal β1-4 (4F-Fuc α1-3) 6F-GlcNAc-ITag 150
2.18.106 Supplementary Figure 123. HRMS ITag screening assay mass spectrum of synthesis of 4F-Gal β1-4 (4F-Fuc α1-3) 6F-GlcNTFA-ITag 151
2.18.107 Supplementary Figure 124. HRMS ITag screening assay mass spectrum of synthesis of 4F-Gal β1-4 (4F-Fuc α1-3) 6,6-diFGlcNAc-ITag 152

2.18.108 Supplementary Figure 125. HRMS ITag screening assay mass spectrum of synthesis of 4F-Gal β1-4 (4F-Fuc α1-3) 6,6-diFGlcNTFA-ITag 153
2.18.109 Supplementary Figure 126. HRMS ITag screening assay mass spectrum of synthesis of 6F-Gal β1-4 (4F-Fuc α1-3) GlcNAc-ITag 154
2.18.110 Supplementary Figure 127. HRMS ITag screening assay mass spectrum of synthesis of 6F-Gal β1-4 (4F-Fuc α1-3) GlcNTFA-ITag 155
2.18.111 Supplementary Figure 128. HRMS ITag screening assay mass spectrum of synthesis of 6F-Gal β1-4 (4F-Fuc α1-3) 6F-GlcNAc-ITag 156
2.18.112 Supplementary Figure 129. HRMS ITag screening assay mass spectrum of synthesis of 6F-Gal β1-4 (4F-Fuc α1-3) 6F-GlcNTFA-ITag 157
2.18.113 Supplementary Figure 130. HRMS ITag screening assay mass spectrum of synthesis of 6F-Gal β1-4 (4F-Fuc α1-3) 6,6-diFGlcNAc-ITag 158
2.18.114 Supplementary Figure 131. HRMS ITag screening assay mass spectrum of synthesis of 6F-Gal β1-4 (4F-Fuc α1-3) 6,6-diFGlcNTFA-ITag 159
2.18.115 Supplementary Figure 132. HRMS ITag screening assay mass spectrum of synthesis of 6d-Gal β1-4 (3F-Fuc α1-3) GlcNAc-ITag 160
2.18.116 Supplementary Figure 133. HRMS ITag screening assay mass spectrum of synthesis of 6d-Gal β1-4 (4F-Fuc α1-3) GlcNTFA-ITag 161
2.18.117 Supplementary Figure 134. HRMS ITag screening assay mass spectrum of synthesis of 6d-Gal β1-4 (3F-Fuc α1-3) 6F-GlcNAc-ITag 162
2.18.118 Supplementary Figure 135. HRMS ITag screening assay mass spectrum of synthesis of 6d-Gal β1-4 (3F-Fuc α1-3) 6F-GlcNTFA-ITag 163
2.18.119 Supplementary Figure 136. HRMS ITag screening assay mass spectrum of synthesis of 6d-Gal β1-4 (3F-Fuc α1-3) 6,6-diFGlcNAc-ITag 164
2.18.120 Supplementary Figure 137. HRMS ITag screening assay mass spectrum of synthesis of 6d-Gal β1-4 (3F-Fuc α1-3) 6,6-diFGlcNTFA-ITag 165
2.18.121 Supplementary Figure 138. HRMS ITag screening assay mass spectrum of synthesis of Gal β1-4 (6F-Fuc α1-3) GlcNAc-ITag (LeX13) 166
2.18.122 Supplementary Figure 139. HRMS ITag screening assay mass spectrum of synthesis of Gal β1-4 (6F-Fuc α1-3) GlcNTFA-ITag 167
2.18.123 Supplementary Figure 140. HRMS ITag screening assay mass spectrum of synthesis of Gal β1-4 (6F-Fuc α1-3) 6F-GlcNAc-ITag 168
2.18.124 Supplementary Figure 141. HRMS ITag screening assay mass spectrum of synthesis of Gal β1-4 (6F-Fuc α1-3) 6F-GlcNTFA-ITag (LeX21) 169
2.18.125 Supplementary Figure 142. HRMS ITag screening assay mass spectrum of synthesis of Gal β1-4 (6F-Fuc α1-3) 6,6-diFGlcNAc-ITag 170
2.18.126 Supplementary Figure 143. HRMS ITag screening assay mass spectrum of synthesis of Gal β1-4 (6F-Fuc α1-3) 6,6-diFGlcNTFA-ITag 171
2.18.127 Supplementary Figure 144. HRMS ITag screening assay mass spectrum of synthesis of 3F-Gal β1-4 (6F-Fuc α1-3) GlcNAc-ITag (LeX15) 172
2.18.128 Supplementary Figure 145. HRMS ITag screening assay mass spectrum of synthesis of 3F-Gal β1-4 (6F-Fuc α1-3) GlcNTFA-ITag 173
2.18.129 Supplementary Figure 146. HRMS ITag screening assay mass spectrum of synthesis of 3F-Gal β1-4 (6F-Fuc α1-3) 6F-GlcNAc-ITag 174
2.18.130 Supplementary Figure 147. HRMS ITag screening assay mass spectrum of synthesis of 3F-Gal β1-4 (6F-Fuc α1-3) 6F-GlcNTFA-ITag 175
2.18.131 Supplementary Figure 148. HRMS ITag screening assay mass spectrum of synthesis of 3F-Gal β1-4 (6F-Fuc α1-3) 6,6-diFGlcNAc-ITag 176
2.18.132 Supplementary Figure 149. HRMS ITag screening assay mass spectrum of synthesis of 3F-Gal β1-4 (6F-Fuc α1-3) 6,6-diFGlcNTFA-ITag 177
2.18.133 Supplementary Figure 150. HRMS ITag screening assay mass spectrum of synthesis of 4F-Gal β1-4 (6F-Fuc α1-3) GlcNAc-ITag (LeX16) 178
2.18.134 Supplementary Figure 151. HRMS ITag screening assay mass spectrum of synthesis of 4F-Gal β1-4 (6F-Fuc α1-3) GlcNTFA-ITag (LeX24) 179
2.18.135 Supplementary Figure 152. HRMS ITag screening assay mass spectrum of synthesis of 4F-Gal β1-4 (6F-Fuc α1-3) 6F-GlcNAc-ITag 180

2.18.136 Supplementary Figure 153. HRMS ITag screening assay mass spectrum of synthesis of 4F-Gal β1-4 (6F-Fuc α1-3) 6F-GlcNTFA-ITag 181
2.18.137 Supplementary Figure 154. HRMS ITag screening assay mass spectrum of synthesis of 4F-Gal β1-4 (6F-Fuc α1-3) 6,6-diFGlcNAc-ITag 182
2.18.138 Supplementary Figure 155. HRMS ITag screening assay mass spectrum of synthesis of 4F-Gal β1-4 (6F-Fuc α1-3) 6,6-diFGlcNTFA-ITag 183
2.18.139 Supplementary Figure 156. HRMS ITag screening assay mass spectrum of synthesis of 6F-Gal β1-4 (6F-Fuc α1-3) GlcNAc-ITag 184
2.18.140 Supplementary Figure 157. HRMS ITag screening assay mass spectrum of synthesis of 6F-Gal β1-4 (6F-Fuc α1-3) GlcNTFA-ITag 185
2.18.141 Supplementary Figure 158. HRMS ITag screening assay mass spectrum of synthesis of 6F-Gal β1-4 (6F-Fuc α1-3) 6F-GlcNAc-ITag 186
2.18.142 Supplementary Figure 159. HRMS ITag screening assay mass spectrum of synthesis of 6F-Gal β1-4 (6F-Fuc α1-3) 6F-GlcNTFA-ITag 187
2.18.143 Supplementary Figure 160. HRMS ITag screening assay mass spectrum of synthesis of 6F-Gal β1-4 (6F-Fuc α1-3) 6,6-diFGlcNAc-ITag 188
2.18.144 Supplementary Figure 161. HRMS ITag screening assay mass spectrum of synthesis of 6F-Gal β1-4 (6F-Fuc α1-3) 6,6-diFGlcNTFA-ITag 189
2.18.145 Supplementary Figure 162. HRMS ITag screening assay mass spectrum of synthesis of 6d-Gal β1-4 (6F-Fuc α1-3) GlcNAc-ITag 190
2.18.146 Supplementary Figure 163. HRMS ITag screening assay mass spectrum of synthesis of 6d-Gal β1-4 (6F-Fuc α1-3) GlcNTFA-ITag 191
2.18.147 Supplementary Figure 164. HRMS ITag screening assay mass spectrum of synthesis of 6d-Gal β1-4 (6F-Fuc α1-3) 6F-GlcNAc-ITag 192
2.18.148 Supplementary Figure 165. HRMS ITag screening assay mass spectrum of synthesis of 6d-Gal β1-4 (6F-Fuc α1-3) 6F-GlcNTFA-ITag 193
2.18.149 Supplementary Figure 166. HRMS ITag screening assay mass spectrum of synthesis of 6d-Gal β1-4 (6F-Fuc α1-3) 6,6-diFGlcNAc-ITag 194
2.18.150 Supplementary Figure 167. HRMS ITag screening assay mass spectrum of synthesis of 6d-Gal β1-4 (6F-Fuc α1-3) 6,6-diFGlcNTFA-ITag 195
2.18.151 Supplementary Figure 168. HRMS ITag screening assay mass spectrum of synthesis of Gal β1-4 (Ara α1-3) GlcNAc-ITag (LeX14) 196
2.18.152 Supplementary Figure 169. HRMS ITag screening assay mass spectrum of synthesis of Gal β1-4 (Ara α1-3) GlcNTFA-ITag 197
2.18.153 Supplementary Figure 170. HRMS ITag screening assay mass spectrum of synthesis of Gal β1-4 (Ara α1-3) 6F-GlcNAc-ITag 198
2.18.154 Supplementary Figure 171. HRMS ITag screening assay mass spectrum of synthesis of Gal β1-4 (Ara α1-3) 6F-GlcNTFA-ITag 199
2.18.155 Supplementary Figure 172. HRMS ITag screening assay mass spectrum of synthesis of Gal β1-4 (Ara α1-3) 6,6-diFGlcNAc-ITag 200
2.18.156 Supplementary Figure 173. HRMS ITag screening assay mass spectrum of synthesis of Gal β1-4 (Ara α1-3) 6,6-diFGlcNTFA-ITag 201
2.18.157 Supplementary Figure 174. HRMS ITag screening assay mass spectrum of synthesis of 3F-Gal β1-4 (Ara α1-3) GlcNAc-ITag 202
2.18.158 Supplementary Figure 175. HRMS ITag screening assay mass spectrum of synthesis of 3F-Gal β1-4 (Ara α1-3) GlcNTFA-ITag 203
2.18.159 Supplementary Figure 176. HRMS ITag screening assay mass spectrum of synthesis of 3F-Gal β1-4 (Ara α1-3) 6F-GlcNAc-ITag 204
2.18.160 Supplementary Figure 177. HRMS ITag screening assay mass spectrum of synthesis of 3F-Gal β1-4 (Ara α1-3) 6F-GlcNTFA-ITag 205
2.18.161 Supplementary Figure 178. HRMS ITag screening assay mass spectrum of synthesis of 3F-Gal β1-4 (Ara α1-3) 6,6-diFGlcNAc-ITag 206
2.18.162 Supplementary Figure 179. HRMS ITag screening assay mass spectrum of synthesis of 3F-Gal β1-4 (Ara α1-3) 6,6-diFGlcNTFA-ITag 207
2.18.163 Supplementary Figure 180. HRMS ITag screening assay mass spectrum of synthesis of 4F-Gal β1-4 (Ara α1-3) GlcNAc-ITag 208

2.18.164 Supplementary Figure 181. HRMS ITag screening assay mass spectrum of synthesis of 4F-Gal β1-4 (Ara α1-3) GlcNTFA-ITag ... 209
2.18.165 Supplementary Figure 182. HRMS ITag screening assay mass spectrum of synthesis of 4F-Gal β1-4 (Ara α1-3) 6F-GlcNAc-ITag ... 210
2.18.166 Supplementary Figure 183. HRMS ITag screening assay mass spectrum of synthesis of 4F-Gal β1-4 (Ara α1-3) 6F-GlcNTFA-ITag ... 211
2.18.167 Supplementary Figure 184. HRMS ITag screening assay mass spectrum of synthesis of 4F-Gal β1-4 (Ara α1-3) 6,6-diFGlcNAc-ITag ... 212
2.18.168 Supplementary Figure 185. HRMS ITag screening assay mass spectrum of synthesis of 4F-Gal β1-4 (Ara α1-3) 6,6-diFGlcNTFA-ITag ... 213
2.18.169 Supplementary Figure 186. HRMS ITag screening assay mass spectrum of synthesis of 6F-Gal β1-4 (Ara α1-3) GlcNAc-ITag ... 214
2.18.170 Supplementary Figure 187. HRMS ITag screening assay mass spectrum of synthesis of 6F-Gal β1-4 (Ara α1-3) GlcNTFA-ITag ... 215
2.18.171 Supplementary Figure 188. HRMS ITag screening assay mass spectrum of synthesis of 6F-Gal β1-4 (Ara α1-3) 6F-GlcNAc-ITag ... 216
2.18.172 Supplementary Figure 189. HRMS ITag screening assay mass spectrum of synthesis of 6F-Gal β1-4 (Ara α1-3) 6F-GlcNTFA-ITag ... 217
2.18.173 Supplementary Figure 190. HRMS ITag screening assay mass spectrum of synthesis of 6F-Gal β1-4 (Ara α1-3) 6,6-diFGlcNAc-ITag ... 218
2.18.174 Supplementary Figure 191. HRMS ITag screening assay mass spectrum of synthesis of 6F-Gal β1-4 (Ara α1-3) 6,6-diFGlcNTFA-ITag ... 219
2.18.175 Supplementary Figure 192. HRMS ITag screening assay mass spectrum of synthesis of 6d-Gal β1-4 (Ara α1-3) GlcNAc-ITag ... 220
2.18.176 Supplementary Figure 193. HRMS ITag screening assay mass spectrum of synthesis of 6d-Gal β1-4 (Ara α1-3) GlcNTFA-ITag ... 221
2.18.177 Supplementary Figure 194. HRMS ITag screening assay mass spectrum of synthesis of 6d-Gal β1-4 (Ara α1-3) 6F-GlcNAc-ITag ... 222
2.18.178 Supplementary Figure 195. HRMS ITag screening assay mass spectrum of synthesis of 6d-Gal β1-4 (Ara α1-3) 6F-GlcNTFA-ITag ... 223
2.18.179 Supplementary Figure 196. HRMS ITag screening assay mass spectrum of synthesis of 6d-Gal β1-4 (Ara α1-3) 6,6-diFGlcNAc-ITag ... 224
2.18.180 Supplementary Figure 197. HRMS ITag screening assay mass spectrum of synthesis of 6d-Gal β1-4 (Ara α1-3) 6,6-diFGlcNTFA-ITag ... 225
2.19 Supplementary Figures 198-266. NMR spectra of the upscaled Lewis^x and its glycofluoroforms ... 226
2.19.1 LeX1 ... 226
2.19.1.1 Supplementary Figure 198. ^{1}H NMR spectrum for compound **LeX** ... 226
2.19.1.2 Supplementary Figure 199. ^{13}C NMR spectrum for compound **LeX1** ... 227
2.19.2 LeX2 ... 227
2.19.2.1 Supplementary Figure 200. ^{1}H NMR spectrum for compound **LeX2** ... 227
2.19.2.2 Supplementary Figure 201. ^{13}C NMR spectrum for compound **LeX2** ... 228
2.19.2.3 Supplementary Figure 202. ^{19}F{1H} NMR spectrum for compound **LeX2** ... 228
2.19.3 LeX3 ... 229
2.19.3.1 Supplementary Figure 203. ^{1}H NMR spectrum for compound **LeX3** ... 229
2.19.3.2 Supplementary Figure 204. HSQC NMR spectrum for compound **LeX3** ... 229
2.19.3.3 Supplementary Figure 205. ^{19}F{1H} NMR spectrum for compound **LeX3** ... 230
2.19.4 LeX4 ... 231
2.19.4.1 Supplementary Figure 206. ^{1}H NMR spectrum for compound **LeX4** ... 231
2.19.4.2 Supplementary Figure 207. ^{13}C NMR spectrum for compound **LeX4** ... 231
2.19.4.3 Supplementary Figure 208. ^{19}F{1H} NMR spectrum for compound **LeX4** ... 232
2.19.5 LeX5 ... 233
2.19.5.1 Supplementary Figure 209. ^{1}H NMR spectrum for compound **LeX5** ... 233
2.19.5.2 Supplementary Figure 210. ^{13}C NMR spectrum for compound **LeX5** ... 233
2.19.5.3 Supplementary Figure 211. ^{19}F{1H} NMR spectrum for compound **LeX5** ... 234
2.19.6 LeX6 ... 235
2.19.6.1 Supplementary Figure 212. ^{1}H NMR spectrum for compound **LeX6** ... 235

2.19.6.2 Supplementary Figure 213. ^{13}C NMR spectrum for compound **LeX6** 235
2.19.6.3 Supplementary Figure 214. $^{19}F\{1H\}$ NMR (Insert: ^{19}F NMR expansion) spectrum for compound **LeX6** 236
2.19.7 LeX7 237
2.19.7.1 Supplementary Figure 215. ^{1}H NMR spectrum for compound **LeX7** 237
2.19.7.2 Supplementary Figure 216. ^{13}C NMR spectrum for compound **LeX7** 237
2.19.7.3 Supplementary Figure 217. $^{19}F\{1H\}$ NMR (Insert: ^{19}F NMR expansion) spectrum for compound **LeX7** 238
2.19.8 LeX8 239
2.19.8.1 Supplementary Figure 218. ^{1}H NMR spectrum for compound **LeX8** 239
2.19.8.2 Supplementary Figure 219. ^{13}C NMR spectrum for compound **LeX8** 239
2.19.8.3 Supplementary Figure 220. $^{19}F\{1H\}$ NMR (Insert: ^{19}F NMR expansion) spectrum for compound **LeX8** 240
2.19.9 LeX9 241
2.19.9.1 Supplementary Figure 221. ^{1}H NMR spectrum for compound **LeX9** 241
2.19.9.2 Supplementary Figure 222. HSQC NMR spectrum for compound **LeX9** 241
2.19.9.3 Supplementary Figure 223. $^{19}F\{1H\}$ NMR**9** 242
2.19.10 LeX10 243
2.19.10.1 Supplementary Figure 224. ^{1}H NMR spectrum for compound **LeX10** 243
2.19.10.2 Supplementary Figure 225. ^{13}C NMR spectrum for compound **LeX10** 243
2.19.11 LeX11 244
2.19.11.1 Supplementary Figure 226. ^{1}H NMR spectrum for compound **LeX11** 244
2.19.11.2 Supplementary Figure 227. ^{13}C NMR spectrum for compound **LeX11** 244
2.19.11.3 Supplementary Figure 228. $^{19}F\{1H\}$ NMR (Insert: ^{19}F NMR expansion) spectrum for compound **LeX11** 245
2.19.12 LeX12 246
2.19.12.1 Supplementary Figure 229. ^{1}H NMR spectrum for compound **LeX12** 246
2.19.12.2 Supplementary Figure 230. ^{13}C NMR spectrum for compound **LeX12** 246
2.19.12.3 Supplementary Figure 231. $^{19}F\{1H\}$ NMR (Insert: ^{19}F NMR expansion) spectrum for compound **LeX12** 247
2.19.13 LeX13 248
2.19.13.1 Supplementary Figure 232. ^{1}H NMR spectrum for compound **LeX13** 248
2.19.13.2 Supplementary Figure 233. ^{13}C NMR spectrum for compound **LeX13** 248
2.19.13.3 Supplementary Figure 234. $^{19}F\{1H\}$ NMR (Insert: ^{19}F NMR expansion) spectrum for compound **LeX13** 249
2.19.14 LeX14 250
2.19.14.1 Supplementary Figure 235. ^{1}H NMR spectrum for compound **LeX14** 250
2.19.14.2 Supplementary Figure 236. ^{13}C NMR spectrum for compound **LeX14** 250
2.19.15 LeX15 251
2.19.15.1 Supplementary Figure 237. ^{1}H NMR spectrum for compound **LeX15** 251
2.19.15.2 Supplementary Figure 238. ^{13}C NMR spectrum for compound **LeX15** 251
2.19.15.3 Supplementary Figure 239. $^{19}F\{1H\}$ NMR (Insert: ^{19}F NMR expansions) spectrum for compound **LeX15** 252
2.19.16 LeX16 253
2.19.16.1 Supplementary Figure 240. ^{1}H NMR spectrum for compound **LeX16** 253
2.19.16.2 Supplementary Figure 241. ^{13}C NMR spectrum for compound **LeX16** 253
2.19.16.3 Supplementary Figure 242. $^{19}F\{1H\}$ NMR (Insert: ^{19}F NMR expansions) spectrum for compound **LeX16** 254
2.19.17 LeX17 255
2.19.17.1 Supplementary Figure 243. ^{1}H NMR spectrum for compound **LeX17** 255
2.19.17.2 Supplementary Figure 244. ^{13}C NMR spectrum for compound **LeX17** 256
2.19.17.3 Supplementary Figure 245. $^{19}F\{1H\}$ NMR (Insert: ^{19}F NMR expansions) spectrum for compound **LeX17** 256
2.19.18 LeX18 257
2.19.18.1 Supplementary Figure 246. ^{1}H NMR spectrum for compound **LeX18** 257
2.19.18.2 Supplementary Figure 247. ^{13}C NMR spectrum for compound **LeX18** 257

2.19.18.3 Supplementary Figure 248. $^{19}F\{1H\}$ NMR (Insert: ^{19}F NMR expansions) spectrum for compound **LeX18** 258
2.19.19 LeX19 259
2.19.19.1 Supplementary Figure 249. ^{1}H NMR spectrum for compound **LeX19** 259
2.19.19.2 Supplementary Figure 250. ^{13}C NMR spectrum for compound **LeX19** 259
2.19.19.3 Supplementary Figure 251. $^{19}F\{1H\}$ NMR (Insert: ^{19}F NMR expansions) spectrum for compound **LeX19** 260
2.19.20 LeX20 261
2.19.20.1 Supplementary Figure 252. ^{1}H NMR spectrum for compound **LeX20** 261
2.19.20.2 Supplementary Figure 253. ^{13}C NMR spectrum for compound **LeX20** 261
2.19.20.3 Supplementary Figure 254. $^{19}F\{1H\}$ NMR spectrum for compound **LeX20** 262
2.19.21 LeX21 263
2.19.21.1 Supplementary Figure 255. ^{1}H NMR spectrum for compound **LeX21** 263
2.19.21.2 Supplementary Figure 256. ^{13}C NMR spectrum for compound **LeX21** 263
2.19.21.3 Supplementary Figure 257. $^{19}F\{1H\}$ NMR (Insert: ^{19}F NMR expansions) spectrum for compound **LeX21** 264
2.19.22 LeX22 265
2.19.22.1 Supplementary Figure 258. ^{1}H NMR spectrum for compound **LeX22** 265
2.19.22.2 Supplementary Figure 259. ^{13}C NMR spectrum for compound **LeX22** 265
2.19.22.3 Supplementary Figure 260. $^{19}F\{1H\}$ NMR spectrum for compound **LeX22** 266
2.19.23 LeX23 267
2.19.23.1 Supplementary Figure 261. ^{1}H NMR spectrum for compound **LeX23** 267
2.19.23.2 Supplementary Figure 262. ^{13}C NMR spectrum for compound **LeX23** 267
2.19.23.3 Supplementary Figure 263. $^{19}F\{1H\}$ NMR (Insert: ^{19}F NMR expansion) spectrum for compound **LeX23** 268
2.19.24 LeX24 269
2.19.24.1 Supplementary Figure 264. ^{1}H NMR spectrum for compound **LeX24** 269
2.19.24.2 Supplementary Figure 265. ^{13}C NMR spectrum for compound **LeX24** 269
2.19.24.3 Supplementary Figure 266. $^{19}F\{1H\}$ NMR (Insert: ^{19}F NMR expansion) spectrum for compound **LeX24** 270
2.20 Supplementary Figures 267-268. NGL related *270*
2.20.1 Supplementary Figure 267. Negative-ion MALDI-MS spectrum of the DBCO-DH lipid reagent. 270
2.20.2 Supplementary Figure 268. Negative-ion MALDI-MS spectra of the 24 Lewis^{X} related NGLs. R_1= OCH2-CH2-CH2-N_3-DBCO-DH. 271

3 Supplementary Tables 274

3.1 Supplementary Table 1. ESMS-derived conversion efficiencies of GlcNAc derivatives bearing ITags to LacNAc analogues, and from LacNAc analogues to Lewis^{x} analogues containing fucose, 3F-Fuc, 4F-Fuc, 6F-Fuc, and Ara 274
3.2 Supplementary Table 2. List of the NGL probes included in the microarray study with their glycan sequences. 275
*3.3 Supplementary Table 3. Supplemental glycan microarray document based on MIRAGE Guidelines **(doi:10.3762/mirage.3)**[2] 278*
3.4 Supplementary Table 4. Summary of effects on glycan binding by proteins with the 11 individual modifications on the Lewis^{X} structure. 282
3.5 Supplementary Table 5 Summary of results of negative-ion MALDI-MS analysis of the 24 LewisX NGLs. 282
3.6 Supplementary Table 6: Characterisation of non-functionalised and functionalised AuNPs used in this study. 283

4 Supplementary Notes 284

4.1 Structures of bacterial toxin-Lewis glycan complexes (Supplementary Figures 269–270) 284
4.2 *Proof-of-concept: AuNP-based detection of CTB using **LeX4** and **LeX16** (Figures 271–272) 286*

5 Supplementary References 288

1 Supplementary methods

1.1 ITag synthesis

1.1.1 1-(4-Carboxybenzyl)-3-methyl-1*H*-imidazol-3-ium

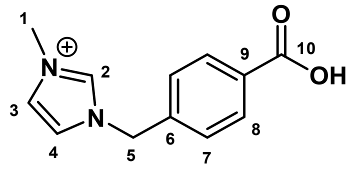

4-(bromomethyl) benzoic acid (500 mg, 2.33 mmol, 1 eq) in MeCN (5ml, 0.5 M) had 1-Methyl imidazole (1.5 ml, 18.64 mmol, 8 eq) added. A white precipitate formed after 15 mins. The white precipitate was collected by filtration washing with MeCN (393 mg, 78%). ^{1}H NMR (501 MHz, MeOD) δ 9.09 (1H, H-4, s), 7.98-8.02 (2H, H-7, d, *J* = 8.5 Hz), 7.60-7.66 (2H, H-2, H-3, dt, *J* = 21.7, 1.89 Hz), 7.43-7.47 (2H, H-6, d, *J* = 8.5 Hz), 5.49 (2H, H-5, s), 3.94 (3H, H-1, s). ^{13}C NMR (126 MHz, MeOD) δ 171.6 (C-10), 138.6 (C-2), 138.2 (C-9), 136.5 (C-6), 131.4 (C-8), 129.3 (C-7), 125.4 (C-3), 123.8 (C-4), 53.6 (C-5), 36.7 (C-1). HRMS- $C_{12}H13N_2O_2^+$ requires 217.0972. Measured m/z $[M]^+$ = 217.0983.

1.1.2 1-Methyl-3-((4-[(prop-2-yn-1-yl)carbamoyl]phenyl)methyl)imidazol-1-ium

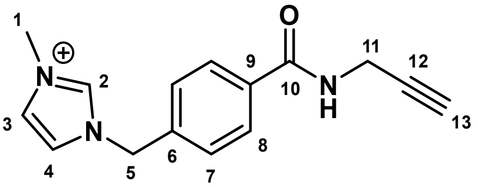

To a stirred solution of 1-(4-carboxybenzyl)-3-methyl-1*H*-imidazol-3-ium (100mg, 0.46 mmol, 1 eq), HCTU (248 mg, 0.58 mmol, 1.3 eq) and DIPEA (0.16 ml, 0.92 mmol, 2.5 eq) in DMF (2 ml, 0.2 M), propargylamine was added dropwise over 30 min. The reaction was stirred for 18 hrs. The product was then purified by cation exchange chromatography eluting in a gradient of 0.1-1M $(NH_4)HCO_3$, the fractions between 0.1 M and 0.5 M were combined and the product was isolated by lyophilisation to yield an off white solid (60%). ^{1}H NMR (501 MHz, MeOD) δ 7.88-7.92 (2H, H-8, d, *J* = 8.4), 7.59-7.66 (2H, H-3, H-4, dd, *J* = 14.86, 2.0), 7.48 (2H, H-7, d, *J* = 8.4), 5.49 (2H, H-5, s), 4.15 (2H, H-11, s), 3.93 (3H, H-1, s), 3.01 (1H, H-13, s). ^{13}C NMR (126 MHz, MeOD) δ 168.8 (C-10). 161.5 (C-2), 138.9 (C-9), 136.0 (C-6), 129.6 (C-8), 129.4 (C-7), 125.4 (C-3), 123.8 (C-4), 52.1 (C-5), 40.0 (C-13), 36.6 (C-11), 36.5 (C-12), 29.9 (C-1). HRMS- $C_{15}H_{16}N_3O^+$ requires 254.3125. Measured m/z $[M]^+$ =254.1307.

1.2 Lewis^x synthesis

1.2.1 Gal β1-4 (Fuc α1-3) GlcNAc-N_3 (LeX1)

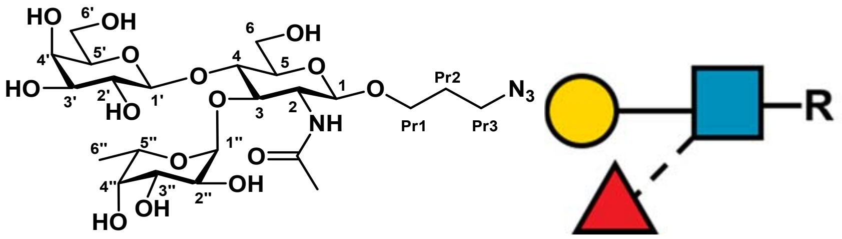

GlcNAc-N_3 (10 mM), UDP-Gal (11 mM), $MnCl_2$ (10 mM), BSA (1 mg / ml), B4GalT1 HS (30 μM), Tris buffer (100 mM, pH 8.0) in H_2O at a total volume of 3.25 ml were incubated overnight at 37 °C. To the crude Gal β1-4 GlcNAc-N3 (4 mM) was added Tris, pH 7.0 (200 mM), $MgCl_2$ (10 mM), ATP (16 mM), GTP (8 mM), Fuc (8 mM), α1-3 FucT HP (8 uM) and FKP (5 uM) in H_2O at a total volume of 8.12 ml this was incubated overnight at 37 °C. The reaction mixture was passed

through a 10K MWCO spin concentrator and the filtrate was dried under reduced pressure with 600 mg silica gel. The resulting powder was loaded into a 4 g empty dry load cartridge and connected to a 12 g flash silica cartridge. The separation was run on a biotage flash system (20:80 MeOH:EtOAc → 50:50 MeOH:EtOAc over 15 CV). The fractions containing the product were pooled, the solvent removed under reduced pressure and re-dissolved in water. The mixture was then finally purified by size exclusion on a 10/300 biogel P2 column and the fractions containing product pooled and freeze dried to give the product as a white powder. (19.2 mg, 58%). 1H NMR (501 MHz, Deuterium Oxide) δ 5.12 (d, J = 4.0 Hz, 1H, H-1''), 4.84 (q, J = 6.6, 1H, H-5''), 4.55 (d, J = 8.0 Hz, 1H, H-1), 4.46 (d, J = 7.8 Hz, 1H, H-1'), 4.01 (dd, J = 12.4, 2.5 Hz, 1H, H-6a), 3.98 – 3.82 (m, 7H, H-2, H-3, H-3'', H-4, H-4', H-6b, H-Pr1a), 3.80 (d, J = 3.3 Hz, 1H, H-4''), 3.77 – 3.64 (m, 5H, H-2'', H-3', H-6a', H-6b', H-Pr1b), 3.63 – 3.57 (m, 2H, H-5, H-5'), 3.50 (dd, J = 9.9, 7.8 Hz, 1H, H-2'), 3.38 (td, J = 6.5, 4.5 Hz, 2H, H-Pr3), 2.05 (s, 3H, H-Ac), 1.85 (p, J = 6.6 Hz, 2H, H-Pr2), 1.18 (d, J = 6.6 Hz, 3H, H-6''). 13C NMR (126 MHz, D2O) δ 174.2 (C=O), 101.8 (C-1'), 100.9 (C-1), 98.6 (C-1''), 75.3 (C-5'), 74.9 (C-3, C-5), 73.3 (C-4), 72.4 (C-3'), 71.9 (C-4''), 71.0 (C-2'), 69.2 (C-4'), 68.3 (C-3''), 67.7 (C-2''), 67.2 (C-Pr1), 66.7 (C-5''), 61.5 (C-6'), 59.8 (C-6), 55.8 (C-2), 47.7 (C-Pr3), 28.1 (C-Pr2), 22.2 (C-Ac), 15.3 (C-6''). HRMS: Found ($[M+Na]^+$) 635.2400, $C_{23}H_{40}N_4O_{15}Na^+$ requires 635.2382.

1.2.2 Gal β1-4 (Fuc α1-3) 6F-GlcNAc-N_3 (LeX2)

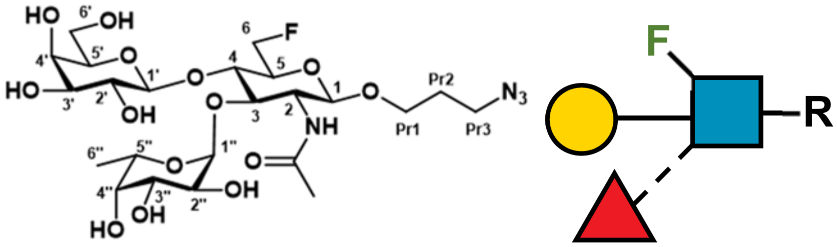

6F-GlcNAc-N3 (SG16131-01, 10 mM), UDP-Gal (11 mM), MnCl2 (10 mM), BSA (1 mg / ml), B4GalT1 HS (30 mM), Tris buffer (100 mM, pH 8.0) in H2O at a total volume of 6.5 ml were incubated overnight at 37 °C. To the crude Gal β1-4 6F-GlcNAc-N3 (4 mM) was added Tris, pH 7.0 (200 mM), MgCl2 (10 mM), ATP (16 mM), GTP (8 mM), Fuc (8 mM), α1-3 FucT HP (8 uM) and FKP (5 uM) in H2O at a total volume of 16.25 ml this was incubated overnight at 37 °C. The reaction mixture was precipitated by adding EtOH (16.25 ml) and the solid removed by centrifugation, the filtrate was subsequently dried under reduced pressure with 600 mg silica gel. The resulting powder was loaded into a 4 g empty dry load cartridge and connected to a 12 g flash silica cartridge. The separation was run on a biotage flash system (30:70 MeOH:EtOAc → 50:50 MeOH:EtOAc over 15 CV). The fractions containing the product were pooled, the solvent removed under reduced pressure and re-dissolved in water. The mixture was then finally purified by size exclusion on a 26/1000 biogel P2 column (20 mM ammonium formate) and the fractions containing product pooled and freeze dried to give a white powder (10 mg, 24%). 1H NMR (400 MHz, Deuterium Oxide) δ 5.10 (d, J = 4.0 Hz, 1H, H-1''), 4.94 – 4.69 (m, 3H, H-5'', H-6a, H-6b), 4.58 (d, J = 7.9 Hz, 1H, H-1), 4.42 (d, J = 7.7 Hz, 1H, H-1'), 4.05 – 3.85 (m, 6H, H-2, H-3, H-3'', H-4, H-4', H-Pr1a), 3.79 (d, J = 3.2 Hz, 1H, H-4''), 3.77 – 3.63 (m, 6H, H-2'', H-3', H-5, H-5', H-6', H-Pr1b), 3.60 (dd, J = 7.7, 4.5 Hz, 1H, H-5'), 3.50 (dd, J = 9.9, 7.7 Hz, 1H, H-2'), 3.37 (td, J = 6.6, 3.3 Hz, 2H, H-Pr3), 2.04 (s, 3H, H-Ac), 1.88 – 1.77 (m, 2H, H-Pr2), 1.17 (d, J = 6.6 Hz, 3H, H-6''). 13C NMR (126 MHz, D2O) δ 171.0 (C=O), 101.9 (C-1'), 101.1 (C-1), 98.7 (C-1''), 81.2 (d, J = 169 Hz, C-6), 74.9 (C-5'), 74.7 (C-3), 73.8 (d, J = 19 Hz, C-5), 72.6 (d, J = 4.3 Hz, C-4), 72.4 (C-3'), 71.9 (C-4''), 71.0 (C-2'), 69.2 (C-4'), 68.3 (C-3''), 67.6 (C-2''), 67.3 (C-Pr1), 66.7 (C-5''), 61.4 (C-6'), 55.7 (C-2), 47.7 (C-Pr3), 28.1 (C-Pr2), 22.2 (C-Ac), 15.3 (C-6''). 19F NMR (376 MHz, Deuterium Oxide) δ -232.49 (dt, J = 47.7, 31.6 Hz, F-6). HRMS: Found 637.2364 ($[M+Na]^+$), $C_{23}H_{39}FN_4O_{14}Na^+$ requires 637.2339.

1.2.3 Gal β1-4 (Fuc α1-3) 6,6-diFGlcNAc-N_3 (**LeX3**)

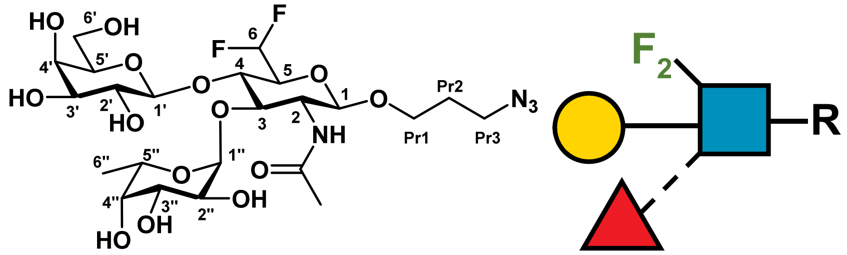

Gal β1-4 6,6-diFGlcNAc-N3 (2 mM), Tris, pH 7.4 (50 mM), $MgCl_2$ (10 mM), ATP (8 mM), GTP (4 mM), Fuc (4 mM), α1-3 FucT HP (4 uM) and FKP (2.4 uM) in H_2O at a total volume of 6.52 ml were incubated overnight at 37 °C. The reaction mixture was passed through a 10K MWCO spin concentrator and the filtrate was lyophilised. The powder was re-dissolved in 500 ul of water, 500 mg of silica was added and the water removed in vacuo. The resulting powder was loaded into a 4 g empty dry load cartridge and connected to a 4 g flash silica cartridge. The separation was run on a biotage flash system (0:100 MeOH:EtOAc → 50:50 MeOH:EtOAc over 15 CV). The fractions containing the product were pooled, the solvent removed under reduced pressure and re-dissolved in water. The mixture was then finally purified by size exclusion on a 10/300 biogel P2 column and the fractions containing product pooled and freeze dried to give the product as a white powder (3.0 mg, 38%). 1H NMR (501 MHz, Deuterium Oxide) δ 6.31 (t, J = 53.0 Hz, 1H, H-6), 5.13 (d, J = 4.1 Hz, 1H, H-1''), 4.79 (m, 1H, H-5''), 4.66 (d, J = 7.8 Hz, 1H, H-1), 4.43 (d, J = 7.8 Hz, 1H, H-1'), 4.08 (t, J = 9.0 Hz, 1H, H-4), 4.02 – 3.93 (m, 4H, H-2, H-3, H-5, H-Pr1a), 3.91 (d, J = 3.4 Hz, 1H, H-4'), 3.90 (dd, J = 9.8, 3.4 Hz, 1H, H-3''), 3.82 – 3.77 (m, 1H, H-4''), 3.76 – 3.68 (m, 3H, H-2'', H-6a', H-6b'), 3.69 – 3.62 (m, 3H, H-3', H-5', H-Pr1b), 3.52 (dd, J = 9.9, 7.7 Hz, 1H, H-2'), 3.38 (td, J = 6.5, 4.1 Hz, 2H, H-Pr3), 2.05 (s, 3H, H-Ac), 1.90 – 1.81 (m, 2H, H-Pr2), 1.19 (d, J = 6.6 Hz, 3H, H-6''). 13C NMR (126 MHz, D2O) δ101.8 (C-1'), 101.3 (C-1), 98.8 (C-1''), 75.1 (C-5'), 74.3 (C-3), 73.3 (C-5), 73.2 (C-4), 72.3 (C-3'), 72.1 (C-4''), 68.7 (C-3'', C-4'), 67.6 (C-2''), 67.5 (C-Pr1), 66.6 (C-5''), 61.9 (C-6'), 55.2 (C-2), 47.8 (C-Pr3), 28.2 (C-Pr2), 22.3 (C-Ac), 15.3 (C-6''). C-6 and C=O were not observed. 19F NMR (376 MHz, Deuterium Oxide) δ -132.29 (m, F-6a, F-6b). 19F{1H} NMR (376 MHz, Deuterium Oxide) δ -132.29 (m, F-6a, F-6b). HRMS: Found ([M+Na]+) 655.2255, $C_{23}H_{38}F_2N_4O_{14}Na^+$ requires 655.2245.

1.2.4 Gal β1-4 (Fuc α1-3) GlcNTFA-N_3 (**LeX4**)

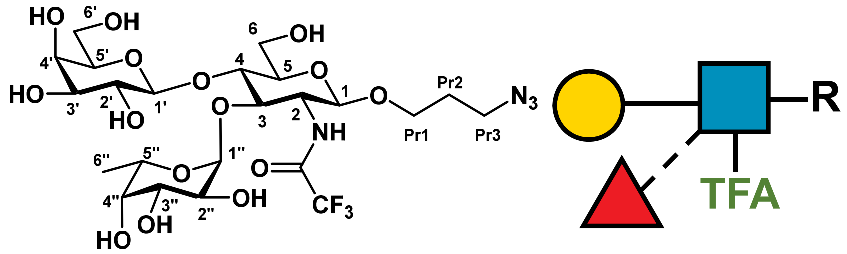

GlcNTFA-N_3 (10 mM), UDP-Gal (11 mM), $MnCl_2$ (10 mM), BSA (1 mg / ml), B4GalT1 HS (30 μM), Tris buffer (100 mM, pH 8.0) in H_2O at a total volume of 3.25 ml were incubated overnight at 37 °C. To the crude Gal β1-4 GlcNAc-N3 (4 mM) was added Tris, pH 7.0 (200 mM), $MgCl_2$ (10 mM), ATP (16 mM), GTP (8 mM), Fuc (8 mM), α1-3 FucT HP (8 uM) and FKP (5 uM) in H_2O at a total volume of 8.12 ml this was incubated overnight at 37 °C. The reaction mixture was passed through a 10K MWCO spin concentrator and the filtrate was dried under reduced pressure with 600 mg silica gel. The resulting powder was loaded into a 4 g empty dry load cartridge and connected to a 12 g flash silica cartridge. The separation was run on a biotage flash system (20:80 MeOH:EtOAc → 50:50 MeOH:EtOAc over 15 CV). The fractions containing the product were pooled, the solvent removed under reduced pressure and re-dissolved in water. The mixture was then finally purified by size exclusion on a 10/300 biogel P2 column and the fractions containing product pooled and freeze dried to give the product as a white powder. (11.6 mg, 63%). 1H NMR (501 MHz, Deuterium Oxide) δ 5.05 (d, J = 4.0 Hz, 1H, H-1''), 4.86 (q, J = 7.4, 6.7 Hz, 1H, H-5''),

4.62 (d, J = 8.4 Hz, 1H, H-1), 4.47 (d, J = 7.8 Hz, 1H, H-1'), 4.04 (dd, J = 10.0, 8.6 Hz, 1H, H-2), 4.02 – 3.96 (m, 4H, H-3, H-4, H-6a, H-Pr1), 3.95 (t, J = 9.3 Hz, 1H, H-3), 3.92 - 3.86 (m, 4H, H-3'', H-4', H-6b, H-Pr1b), 3.80 (d, J = 3.3 Hz, 1H, H-4''), 3.76 – 3.72 (m, 2H, H-6a, H-6b), 3.71 – 3.65 (m, 2H, H-2'', H-3'), 3.62 (m, 2H, H-5, H-5'), 3.52 (dd, J = 9.9, 7.8 Hz, 1H, H-2'), 3.37 (td, J = 6.6, 2.4 Hz, 2H, H-Pr3), 1.85 (p, J = 6.4 Hz, 2H, H-Pr2), 1.18 (d, J = 6.6 Hz, 3H, H-6''). 13C NMR (126 MHz, D2O) δ 159.1 (C=O), 101.8 (C-1'), 100.3 (C-1), 98.9 (C-1''), 75.4 (C-5'), 74.9 (C-5), 74.7 (C-3), 73.2 (C-4), 72.5 (C-3'), 71.9 (C-4''), 71.0 (C-2'), 69.2 (C-3''), 68.4 (C-4'), 67.5 (C-2''), 67.4 (C-Pr1), 66.8 (C-5''), 61.5 (C-6), 59.6 (C-6'), 56.3 (C-2), 47.7 (C-Pr2), 28.0 (C-Pr1), 15.3 (C-6''). 19F NMR (376 MHz, Deuterium Oxide) δ -132.12 (m, F-6a), -75.55 (s, CF_3). HRMS: Found ([M+Na]+) 689.2118, $C_{23}H_{37}F_3N_4O_{15}Na^+$ requires 689.2100.

1.2.5 Gal β1-4 (Fuc α1-3) 6F-GlcNTFA-N_3 (LeX5)

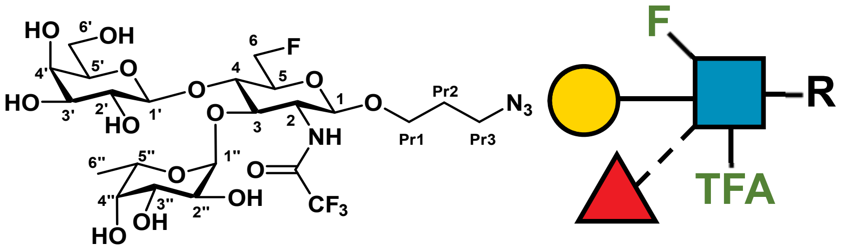

Gal β1-4 6F-GlcNTFA-N3 (2 mM), Tris, pH 7.4 (50 mM), $MgCl_2$ (10 mM), ATP (8 mM), GTP (4 mM), Fuc (4 mM), α1-3 FucT HP (4 uM) and FKP (2.4 uM) in H_2O at a total volume of 6.52 ml were incubated overnight at 37 °C. The reaction mixture was passed through a 10K MWCO spin concentrator and the filtrate was lyophilised. The powder was re-dissolved in 500 ul of water, 500 mg of silica was added and the water removed in vacuo. The resulting powder was loaded into a 4 g empty dry load cartridge and connected to a 4 g flash silica cartridge. The separation was run on a biotage flash system (0:100 MeOH:EtOAc → 50:50 MeOH:EtOAc over 15 CV). The fractions containing the product were pooled, the solvent removed under reduced pressure and re-dissolved in water. The mixture was then finally purified by size exclusion on a 10/300 biogel P2 column and the fractions containing product pooled and freeze dried to give the product as a white powder (9.4 mg, 79%). ^{1}H NMR (501 MHz, Deuterium Oxide) δ 5.03 (d, *J* = 3.9 Hz, 1H, H-1''), 4.94 – 4.72 (m, 2H, H-6a, H-6b), 4.65 (d, *J* = 8.4 Hz, 1H, H-1), 4.43 (d, *J* = 7.7 Hz, 1H, H-1'), 4.05 (t, *J* = 9.3 Hz, 1H, H-2), 4.04 (t, *J* = 9.3 Hz, 1H, H-4), 4.00 – 3.92 (m, 2H, H-3, H-Xa), 3.90 (d, J = Hz, 1H, H-4'), 3.87 (dd, J = 10.2, 2.8 Hz, 1H, H-3''), 3.78 (d, *J* = 3.0 Hz, 1H, H-4''), 3.73 – 3.60 (m, 5H, H-2'', H-3', H-5, H-6a', H-Xb), 3.56 - 3.51 (m, 1H, H-6b'), 3.51 (t, *J* = 8.9 Hz, 1H, H-2'), 3.35 (td, *J* = 6.5, 2.5 Hz, 2H, H-Z), 1.83 (quin, *J* = 6.4 Hz, 2H, H-Y), 1.17 (d, *J* = 6.6 Hz, 3H, H-6''). ^{13}C NMR (126 MHz, D_2O) δ 101.9 (C-1'), 100.4 (C-1), 99.0 (C-1''), 81.1 (d, J = 163 Hz, C-6), 74.9 (C-5'), 74.5 (C-3), 73.9 (d, J = 17 Hz, C-5), 72.5 (d, J = 4 Hz, C-4), 72.4 (C-3'), 71.9 (C-4''), 70.9 (C-2'), 69.2 (C-3''), 68.3 (C-4'), 67.5 (C-X), 67.4 (C-2''), 66.8 (C-5''), 62.4 (C-6'), 56.2 (C-2), 47.7 (C-Z), 28.0 (C-Y), 15.3 (C-6''). C=O peak not observed. 19F NMR (376 MHz, Deuterium Oxide) δ -232.51 (F-6), -75.55 (s, CF_3). HRMS: Found ([M+Na]+) 691.2060, $C_{23}H_{36}F_4N_4O_{14}Na^+$ requires 691.2056.

1.2.6 Gal β1-4 (Fuc α1-3) 6,6-diFGlcNTFA-N_3 (LeX6)

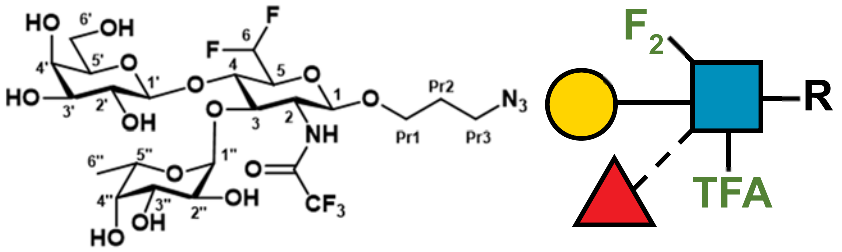

6,6-diGlcNTFA-N3 (10 mM), UDP-Gal (11 mM), MnCl2 (10 mM), BSA (1 mg / ml), B4GalT1 HS (30 μM), Tris buffer (100 mM, pH 8.0) in H_2O at a total volume of 6.5 ml were incubated overnight

at 37 °C. To the crude Gal β1-4 6,6-diGlcNTFA-N3 (4 mM) was added Tris, pH 7.0 (200 mM), MgCl2 (10 mM), ATP (16 mM), GTP (8 mM), Fuc (8 mM), α1-3 FucT HP (8 uM) and FKP (5 uM) in H2O at a total volume of 16.25 ml this was incubated overnight at 37 °C. The reaction mixture was precipitated by adding EtOH (16.25 ml) and the solid removed by centrifugation, the filtrate was subsequently dried under reduced pressure with 600 mg silica gel. The resulting powder was loaded into a 4 g empty dry load cartridge and connected to a 12 g flash silica cartridge. The separation was run on a biotage flash system (30:70 MeOH:EtOAc → 50:50 MeOH:EtOAc over 15 CV). The fractions containing the product were pooled, the solvent removed under reduced pressure and re-dissolved in water. The mixture was then finally purified by size exclusion on a 26/1000 biogel P2 column (20 mM ammonium formate) and the fractions containing product pooled and freeze dried to give a white powder (5.4 mg, 12%). 1H NMR (501 MHz, Deuterium Oxide) δ 6.31 (t, J = 52.6 Hz, 2H, H-6), 5.05 (d, J = 4.0 Hz, 1H, H-1''), 4.82 (q, J = 6.4 Hz, 1H, H-5''), 4.72 (d, J = 8.4 Hz, 1H, H-1), 4.43 (d, J = 7.8 Hz, 1H, H-1'), 4.14 (dd, J = 9.8, 9.0 Hz, 1H, H-4), 4.10 (dd, J = 10.1, 8.4 Hz, 1H, H-2), 4.04 – 3.97 (m, 3H, H-3, H-5, H-Pr1a), 3.91 (d, J = 3.4, 1H, H-4'), 3.88 (dd, J = 10.4, 3.3 Hz, 1H, H-3''), 3.80 (d, J = 3.4, 1H, H-4''), 3.76 – 3.61 (m, 6H, H-2'', H-3', H-5', H-6a', H-6b', H-Pr1b), 3.52 (dd, J = 9.9, 7.8 Hz, 1H, H-2'), 3.37 (td, J = 6.6, 2.1 Hz, 2H, H-Pr3), 1.90 – 1.78 (m, 2H, H-Pr2), 1.19 (d, J = 6.6 Hz, 3H, H-6''). 13C NMR (126 MHz, D2O) δ 170.8 (C=O), 114.6 (t, J = 226 Hz, C-6), 101.8 (C-1''), 100.6 (C-1), 99.0 (C-1'), 75.0 (C-5'), 74.2 (C-3), 73.2 (m, C-4), 72.8 (dd, J = 21, 1.7 Hz, C-5), 72.3 (C-3'), 71.8 (C-4''), 70.9 (C-2'), 69.2 (C-3''), 68.2 (C-4'), 67.8 (C-Pr1), 67.4 (C-2''), 66.9 (C-5''), 61.4 (C-6'), 55.8 (C-2), 47.6 (C-Pr3), 28.0 (C-Pr2), 15.3 (C-6''). 19F NMR (376 MHz, Deuterium Oxide) δ -75.57 (s, F-CF_3), -132.20 (dd, J = 53.0, 13.1 Hz, F-6). 19F{1H} NMR (376 MHz, Deuterium Oxide) δ -75.57 (s, CF_3), -132.20 (s, F-6a, F-6b). HRMS: Found 709.1984 ($[M+Na]^+$), $C_{23}H_{35}F_5N_4O_{14}Na^+$ requires 709.1962.

1.2.7 3F-Gal β1-4 (Fuc α1-3) GlcNAc-N3 (LeX7)

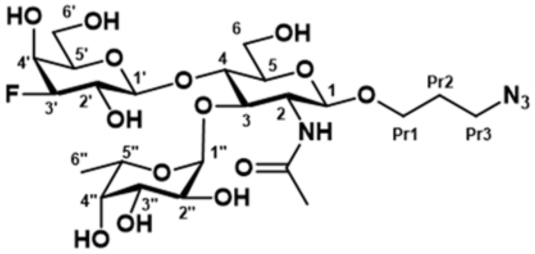

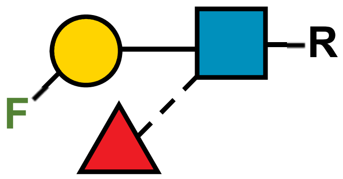

GlcNAc-N3 (SG17011-03, 10 mM), UDP-3F-Gal (11 mM), MnCl2 (10 mM), BSA (1 mg / ml), B4GalT1 HS (30 mM), Tris buffer (100 mM, pH 8.0) in H2O at a total volume of 6.5 ml were incubated overnight at 37 °C. To the crude 3F-Gal β1-4 GlcNAc-N3 (4 mM) was added Tris, pH 7.0 (200 mM), MgCl2 (10 mM), ATP (16 mM), GTP (8 mM), Fuc (8 mM), α1-3 FucT HP (8 uM) and FKP (5 uM) in H2O at a total volume of 16.25 ml this was incubated overnight at 37 °C. The reaction mixture was precipitated by adding EtOH (16.25 ml) and the solid removed by centrifugation, the filtrate was subsequently dried under reduced pressure with 600 mg silica gel. The resulting powder was loaded into a 4 g empty dry load cartridge and connected to a 12 g flash silica cartridge. The separation was run on a biotage flash system (30:70 MeOH:EtOAc → 50:50 MeOH:EtOAc over 15 CV). The fractions containing the product were pooled, the solvent removed under reduced pressure and re-dissolved in water. The mixture was then finally purified by size exclusion on a 26/1000 biogel P2 column (20 mM ammonium formate) and the fractions containing product pooled and freeze dried to give a white powder (1.9 mg, 5%). 1H NMR (400 MHz, Deuterium Oxide) δ 5.11 (d, J = 3.9 Hz, 1H, H-1''), 4.82 (q, J = 6.8 Hz, 1H, H-5''), 4.57 (ddd, J = 49.6, 9.6, 3.6 Hz, 1H, H-3'), 4.53 (d, J = 7.3 Hz, 1H, H-1), 4.50 (d, J = 7.7 Hz, 1H, H-1'), 4.20 (dd, J = 6.5, 3.5 Hz, 1H, H-4'), 4.00 (dd, J = 13.2, 2.7 Hz, 1H, H-6a'), 3.97 – 3.81 (m, 6H, H-2, H-3, H-3'', H-4, H-6b, H-Pr1a), 3.81 – 3.62 (m, 5H, H-2', H-2'', H-4'', H-6', H-Pr1b), 3.60 (m, 2H, H-5, H-5'), 3.36 (ddt, J = 7.6, 3.9, 1.9 Hz, 2H, H-Pr3), 2.04 (s, 3H, H-Ac), 1.89 – 1.79 (m, 2H), 1.17 (d, J = 6.7, 3H, H-6''). 13C NMR (126 MHz, D2O) δ 171.3 (C=O), 101.1 (C-1, C-1'), 98.6 (C-1''), 93.1 (d, J = 185 Hz, C-3'), 74.9 (d, J = 49 Hz, C-5'), 74.6 (C-3), 74.5 (C-5), 74.0 (C-4), 71.5 (C-5), 70.4 (d, J = 17 Hz,

C-2'), 69.1 (C-3''), 68.2 (C-2''), 67.3 (C-Pr1), 66.6 (d, J = 16 Hz, C-4'), 66.4 (C-5''), 61.1 (C-6'), 59.3 (C-6), 55.9 (C-2), 28.1 (C-Pr2), 22.2 (C-Ac), 15.2 (C-6''). 19F NMR (376 MHz, Deuterium Oxide) δ -198.60 (ddd, J = 48.5, 12.6, 5.3 Hz, F-3'). HRMS: Found 637.2355 ($[M+Na]^+$), $C_{23}H_{39}FN_4O_{14}Na^+$ requires 637.2339.

1.2.8 4F-Gal β1-4 (Fuc α1-3) GlcNAc-N_3 (LeX8)

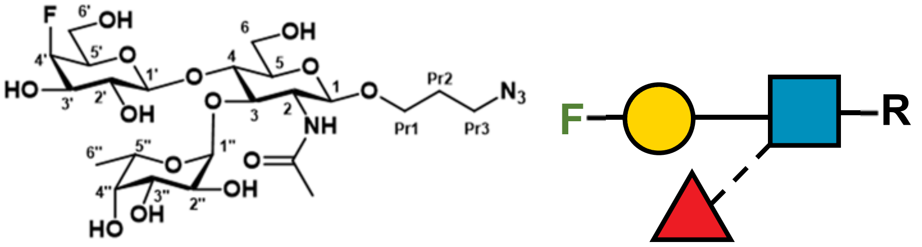

GlcNAc-N3 (SG17011-03, 10 mM), UDP-4F-Gal (11 mM), MnCl2 (10 mM), BSA (1 mg / ml), B4GalT1 HS (30 mM), Tris buffer (100 mM, pH 8.0) in H2O at a total volume of 6.5 ml were incubated overnight at 37 °C. To the crude 4F-Gal β1-4 GlcNAc-N3 (4 mM) was added Tris, pH 7.0 (200 mM), MgCl2 (10 mM), ATP (16 mM), GTP (8 mM), Fuc (8 mM), α1-3 FucT HP (8 uM) and FKP (5 uM) in H2O at a total volume of 16.25 ml this was incubated overnight at 37 °C. The reaction mixture was precipitated by adding EtOH (16.25 ml) and the solid removed by centrifugation, the filtrate was subsequently dried under reduced pressure with 600 mg silica gel. The resulting powder was loaded into a 4 g empty dry load cartridge and connected to a 12 g flash silica cartridge. The separation was run on a biotage flash system (30:70 MeOH:EtOAc → 50:50 MeOH:EtOAc over 15 CV). The fractions containing the product were pooled, the solvent removed under reduced pressure and re-dissolved in water. The mixture was then finally purified by size exclusion on a 26/1000 biogel P2 column (20 mM ammonium formate) and the fractions containing product pooled and freeze dried to give a white powder (6.1 mg, 15%). 1H NMR (501 MHz, Deuterium Oxide) δ 5.10 (d, J = 4.0 Hz, 1H, H-1''), 4.82 – 4.78 (m, 1H, H-5''), 4.80 (d, J = 50.5, 2.7 Hz, 1H, H-4'), 4.53 (dd, J = 7.8, 2H, H-1, H-1'), 3.99 (dd, J = 12.4, 2.2 Hz, 1H, H-6a), 3.97 – 3.82 (m, 7H, H-2, H-3, H-3'', H-4, H-4'', H-6b, H-Pr1a), 3.81 – 3.70 (m, 4H, H-3', H-5', H-6a', H-6b'), 3.70 – 3.63 (m, 2H, H-2'', H-Pr1b), 3.58 (ddd, J = 9.8, 4.7, 2.3 Hz, 1H, H-5), 3.49 (dd, J = 10.0, 7.8, 1H, H-2'), 3.37 (td, J = 6.5, 4.6 Hz, 2H, H-Pr3), 2.03 (s, 3H, H-Ac), 1.87 – 1.78 (m, 2H, H-Pr2), 1.16 (d, J = 6.6 Hz, 3H, H-6''). 13C NMR (126 MHz, D2O) δ 174.2 (C=O), 101.2 (C-1, C-1'), 98.6 (C-1''), 89.4 (d, J = 179 Hz, C-4'), 75.2 (C-5), 74.8 (C-3), 73.4 (C-4), 73.2 (d, J = 17 Hz, C-5'), 71.9 (C-2'), 71.2 (d, J = 18 Hz, C-3'), 69.0 (C-3'', C-4''), 68.2 (C-2''), 67.3 (C-Pr1), 66.6 (C-5''), 60.1 (d, J = 5 Hz, C-6'), 59.7 (C-6), 55.9 (C-2), 47.8 (C-Pr3), 28.3 (C-Pr2), 22.3 (C-Ac), 15.3 (C-6''). 19F NMR (376 MHz, Deuterium Oxide) δ -218.04 (dt, J = 49.5, 30.2 Hz, F-4'). HRMS: Found 637.2356 ($[M+Na]^+$), $C_{23}H_{39}FN_4O_{14}Na^+$ requires 637.2339.

1.2.9 6F-Gal β1-4 (Fuc α1-3) GlcNAc-N_3 (LeX9)

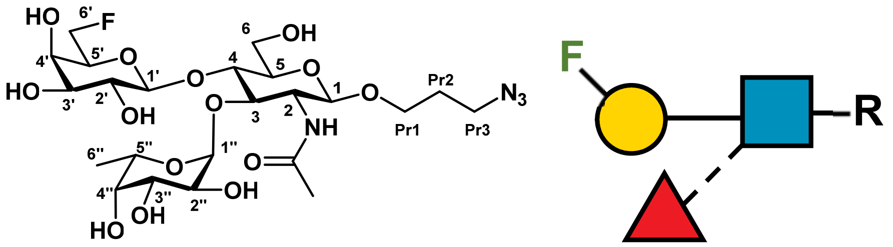

6,6-diFGlcNTFA-N3 (SG16251-01, 10 mM), UDP-6d-Gal (11 mM), MnCl2 (10 mM), BSA (1 mg / ml), B4GalT1 HS (30 μM), Tris buffer (100 mM, pH 8.0) in H2O at a total volume of 3.25 ml were incubated overnight at 37 °C. To the crude 6d-Gal β1-4 6,6-diFGlcNTFA-N3 (4 mM) was added Tris, pH 7.0 (200 mM), MgCl2 (10 mM), ATP (16 mM), GTP (8 mM), Fuc (8 mM), α1-3 FucT HP (8 uM) and FKP (5 uM) in H2O at a total volume of 8.12 ml this was incubated overnight at 37 °C. The reaction mixture was passed through a 10K MWCO spin concentrator and the filtrate was dried under reduced pressure with 600 mg silica gel. The resulting powder was loaded into a 4 g

empty dry load cartridge and connected to a 12 g flash silica cartridge. The separation was run on a biotage flash system (30:70 MeOH:EtOAc → 50:50 MeOH:EtOAc over 15 CV). The fractions containing the product were pooled, the solvent removed under reduced pressure and re-dissolved in water. The mixture was then finally purified by size exclusion on a 26/1000 biogel P2 column and the fractions containing product pooled and freeze dried to give the product as a white powder. (2.4 mg, 16%). 1H NMR (501 MHz, Deuterium Oxide) δ 5.10 (d, J = 4.0 Hz, 1H, H-1''), 4.88 – 4.82 (q, J = 6.4 Hz, 1H, H-5''), 4.63 (ddd, J = 45.85, 9.8, 4.3 Hz, 1H, H-6a'), 4.56 (ddd, J = 47.8, 9.8, 7.1 Hz, 1H, H-6b'), 4.53 (d, J = 7.6 Hz, 1H, H-1), 4.48 (d, J = 7.8 Hz, 1H, H-1'), 4.00 (dd, J = 12.3, 2.3 Hz, 1H, H-6a), 3.98 – 3.93 (m, 2H, H-4, H-Pr1a), 3.93 – 3.82 (m, 6H, H-2, H-3, H-3'', H-4', H-5', H-6b), 3.78 (d, J = 3.5 Hz, 1H, H-4''), 3.69 – 3.63 (m, 3H, H-2'', H-3', H-Pr1b), 3.58 (ddd, J = 9.4, 4.7, 2.2 Hz, 1H, H-5), 3.51 (dd, J = 9.9, 7.8 Hz, 1H, H-2'), 3.42 – 3.32 (m, 2H, H-Pr3), 2.04 (s, 3H, H-Ac), 1.88 – 1.79 (m, 2H, H-Pr2), 1.17 (d, J = 6.6 Hz, 3H, H-6''). 13C NMR (126 MHz, D2O) δ 101.7 (C-1'), 101.0 (C-1), 98.4 (C-1''), 82.9 (m, C-6'), 75.4 (C-5), 74.6 (C-3), 73.9 (C-5'), 73.4 (C-4), 72.4 (C-3'), 72.0 (C-4''), 70.8 (C-2'), 69.3 (C-4'), 68.8 (C-2''), 67.2 (C-Pr1), 66.7 (C-5''), 59.7 (C-6), 55.8 (C-2), 47.7 (C-Pr3), 28.1 (C-Pr2), 22.1 (C-Ac), 15.2 (C-6''). C=O peak not observed. 19F NMR (376 MHz, D2O) δ -229.99 (td, J = 46.53, 14.87 Hz, F-6'). HRMS: Found 637.2362 ([M+Na]$^+$), $C_{23}H_{39}FN_4O_{14}Na^+$ requires 637.2339.

1.2.10 6d-Gal β1-4 (Fuc α1-3) GlcNAc-N$_3$ (LeX10)

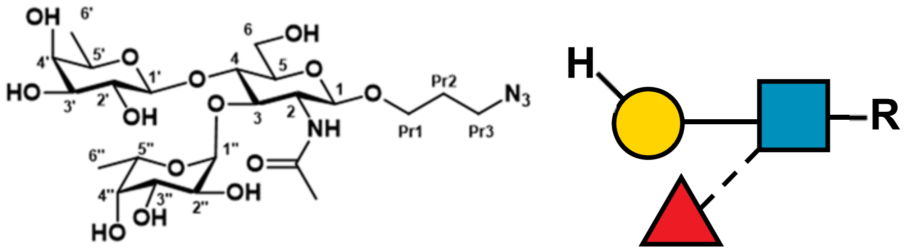

GlcNAc-N3 (10 mM), UDP-6d-Gal (11 mM), MnCl2 (10 mM), BSA (1 mg / ml), B4GalT1 HS (30 mM), Tris buffer (100 mM, pH 8.0) in H2O at a total volume of 6.5 ml were incubated overnight at 37 °C. To the crude 6d-Gal β1-4 GlcNAc-N3 (4 mM) was added Tris, pH 7.0 (200 mM), MgCl2 (10 mM), ATP (16 mM), GTP (8 mM), Fuc (8 mM), α1-3 FucT HP (8 uM) and FKP (5 uM) in H2O at a total volume of 16.25 ml this was incubated overnight at 37 °C. The reaction mixture was precipitated by adding EtOH (16.25 ml) and the solid removed by centrifugation, the filtrate was subsequently dried under reduced pressure with 600 mg silica gel. The resulting powder was loaded into a 4 g empty dry load cartridge and connected to a 12 g flash silica cartridge. The separation was run on a biotage flash system (30:70 MeOH:EtOAc → 50:50 MeOH:EtOAc over 15 CV). The fractions containing the product were pooled, the solvent removed under reduced pressure and re-dissolved in water. The mixture was then finally purified by size exclusion on a 26/1000 biogel P2 column (20 mM ammonium formate) and the fractions containing product pooled and freeze dried to give a white powder (4.5 mg, 11%). 1H NMR (501 MHz, Deuterium Oxide) δ 5.10 (d, J = 4.0 Hz, 1H, H-1''), 4.89 – 4.82 (q, J = 6.7 Hz, 1H, H-5''), 4.52 (d, J = 7.7 Hz, 1H, H-1), 4.41 (d, J = 7.8 Hz, 1H, H-1''), 4.00 (dd, J = 12.3, 2.4 Hz, 1H, H-6a), 3.97 – 3.91 (m, 2H, H-3'', H-Pr1a), 3.90 – 3.82 (m, 4H, H-2, H-3, H-4, H-6b), 3.81 (d, J = 3.3, 1H, H-4''), 3.72 (d, J = 3.4 Hz, 1H, H-4'), 3.69 – 3.62 (m, 5H, H-2'', H-3', H-4', H-5', H-Pr1b), 3.57 (ddt, J = 7.1, 4.8, 2.2 Hz, 1H, H-5), 3.45 (dd, J = 9.9, 7.8 Hz, 1H, H-2'), 3.37 (td, J = 6.5, 4.4 Hz, 2H, H-Pr3), 2.04 (s, 3H, H-Ac), 1.89 – 1.77 (m, 2H, H-Pr2), 1.22 (d, J = 6.4 Hz, 3H, H-6'), 1.17 (d, J = 6.6 Hz, 3H, H-6''). 13C NMR (126 MHz, D2O) δ 101.9 (C-1'), 100.9 (C-1), 98.5 (C-1''), 75.3 (C-5), 74.9 (C-3), 73.6 (C-4), 72.5 (C-3'), 71.9 (C-4''), 71.0 (C-2'), 70.8 (C-4'), 70.3 (C-2''), 69.1 (C-3''), 67.8 (C-5'), 67.2 (C-Pr1), 66.7 (C-5''), 59.7 (C-6), 55.8 (C-2), 47.7 (C-Pr3), 28.1 (C-Pr2), 22.2 (C-Ac), 15.8 (C-6'), 15.3 (C-6''). C=O peak not observed. HRMS: Found 619.2443 ([M+Na]$^+$), $C_{23}H_{40}N_4O_{14}Na^+$ requires 619.2433.

1.2.11 Gal β1-4 (3F-Fuc α1-3) GlcNAc-N_3 (LeX11)

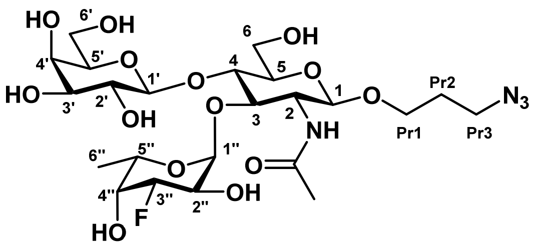

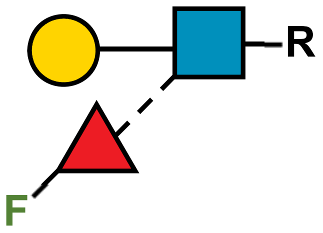

GlcNAc-N_3 (SG17011-02, 10 mM, 15 mg), UDP-Gal (11 mM), $MnCl_2$ (10 mM), BSA (1 mg / ml), B4GalT1 HS (30 μM), Tris buffer (100 mM, pH 8.0) in H_2O at a total volume of 3.25 ml were incubated overnight at 37 °C. To the crude 4F-Gal β1-4 GlcNTFA-N3 (4 mM) was added Tris, pH 7.0 (200 mM), $MgCl_2$ (10 mM), ATP (16 mM), GTP (8 mM), 3F-Fuc (SG18030-01, 8 mM), α1-3 FucT HP (8 uM) and FKP (5 uM) in H_2O at a total volume of 8.12 ml this was incubated overnight at 37 °C. The reaction mixture was passed through a 10K MWCO spin concentrator and the filtrate was dried under reduced pressure with 600 mg silica gel. The resulting powder was loaded into a 4 g empty dry load cartridge and connected to a 12 g flash silica cartridge. The separation was run on a biotage flash system (20:80 MeOH:EtOAc → 50:50 MeOH:EtOAc over 15 CV). The fractions containing the product were pooled, the solvent removed under reduced pressure and re-dissolved in water. The mixture was then finally purified by size exclusion on a 10/300 biogel P2 column and the fractions containing product pooled and freeze dried to give the product as a white powder. (10.5 mg, 50%). 1H NMR (501 MHz, Deuterium Oxide) δ 5.19 (t, J = 4.3 Hz, 1H, H-1''), 4.85 (ddd, J = 50.3, 10.2, 3.4 Hz, 1H, H-3''), 4.84 (q, J = 6.9 Hz, 1H, H-5''), 4.54 (d, J = 7.7 Hz, 1H, H-1), 4.46 (d, J = 7.8 Hz, 1H, H-1''), 4.07 (dd, J = 8.0, 3.4 Hz, 1H, H-4''), 4.01 (dd, J = 12.2, 2.3 Hz, 1H, H-6a), 3.99 – 3.88 (m, 6H, H-2, H-2'', H-3, H-4, H-4', H-Pr1a), 3.86 (dd, J = 12.3, 4.8 Hz, 1H, H-6b), 3.80 – 3.69 (m, 2H, H-6a', H-6b'), 3.70 – 3.64 (m, 1H, H-Pr1b), 3.67 (dd, J = 9.4, 3.5 Hz, 1H, H-3'), 3.64 – 3.57 (m, 2H, H-5, H-5'), 3.48 (dd, J = 9.9, 7.8 Hz, 1H, H-2'), 3.38 (td, J = 6.5, 4.5 Hz, 2H, H-Pr3), 2.05 (s, 3H, H-Ac), 1.85 (p, J = 6.4 Hz, 2H, H-Pr2), 1.22 (d, J = 6.6 Hz, 3H, H-6''). 13C NMR (126 MHz, D2O) δ 174.2 (C=O), 101.8 (C-1'), 100.9 (C-1), 98.4 (d, J = 11 Hz, C-1''), 90.8 (d, J = 176 Hz, C-3''), 75.3 (C-5), 74.9 (C-5'), 74.5 (C-3), 73.3 (C-4), 72.4 (C-3'), 71.1 (C-2'), 70.2 (d, J = 15 Hz, C-4''), 68.3 (C-4'), 67.2 (C-Pr1), 66.3 (C-5''), 66.2 (d, J = 13 Hz, C-2''), 61.4 (C-6'), 59.7 (C-6), 55.8 (C-2), 47.7 (C-Pr3), 28.1 (C-Pr2), 22.2 (C-Ac), 15.0 (C-6''). 19F NMR (471 MHz, D2O) δ -202.8 (1F, m, a coupling of J 50.4 Hz could be isolated, F-3’’), $^{19}F\{^1H\}$ NMR (471 MHz, D2O) δ -202.8 (1F, s, F-3’’), HRMS: Found 637.2340 ([M+Na]+), C23H39FN4O14Na+ requires 637.2339.

1.2.12 Gal β1-4 (4F-Fuc α1-3) GlcNAc-N_3 (LeX12)

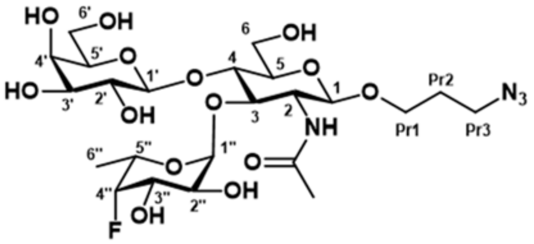

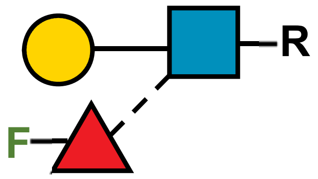

GlcNAc-N3 (SG17011-03, 10 mM), UDP-Gal (11 mM), MnCl2 (10 mM), BSA (1 mg / ml), B4GalT1 HS (30 μM), Tris buffer (100 mM, pH 8.0) in H2O at a total volume of 6.5 ml were incubated overnight at 37 °C. To the crude Gal β1-4 GlcNAc-N3 (4 mM) was added Tris, pH 7.0 (200 mM), MgCl2 (10 mM), ATP (16 mM), GTP (8 mM), 4F-Fuc (SG18010-01, 8 mM), α1-3 FucT HP (8 uM) and FKP (5 uM) in H2O at a total volume of 16.25 ml this was incubated overnight at 37 °C. The reaction mixture was precipitated by adding EtOH (16.25 ml) and the solid removed by centrifugation, the filtrate was subsequently dried under reduced pressure with 600 mg silica gel. The resulting powder was loaded into a 4 g empty dry load cartridge and connected to a 12 g flash silica cartridge. The separation was run on a biotage flash system (30:70 MeOH:EtOAc →

50:50 MeOH:EtOAc over 15 CV). The fractions containing the product were pooled, the solvent removed under reduced pressure and re-dissolved in water. The mixture was then finally purified by size exclusion on a 26/1000 biogel P2 column (20 mM ammonium formate) and the fractions containing product pooled and freeze dried. Impurities still remained therefore a final purification step was performed using a C8 HPLC column (5:95 MeCN → 50:50 MeCN:H2O, 10 min) the fractions were lyophilised to give a white powder (8.8 mg, 22%). 1H NMR (400 MHz, Deuterium Oxide) δ 5.15 (d, J = 3.8 Hz, 1H, H-1''), 4.90 (dq, J = 32.9, 6.8 Hz, 1H, H-5''), 4.58 (dd, J = 36.3, 2.7 Hz, 1H, H-4''), 4.53 (d, J = 7.9 Hz, 1H, H-1), 4.45 (d, J = 7.8 Hz, 1H, H-1'), 4.05 – 3.87 (m, 6H, H-2, H-3, H-3'', H-4, H-4', H-6a, H-Pr1a), 3.85 (dd, J = 12.3, 4.8 Hz, 1H, H-6b), 3.74 (dd, J = 10.4, 3.9 Hz, 1H, H-2''), 3.72 – 3.61 (m, 4H, H-3', H-6a', H-6b', H-Pr1b), 3.61 – 3.53 (m, 2H, H-5, H-5'), 3.46 (dd, J = 9.8, 7.8 Hz, 1H, H-2'), 3.37 (td, J = 6.6, 3.1 Hz, 2H, H-Pr3), 2.03 (s, 3H, H-Ac), 1.83 (dd, J = 6.9, 5.6 Hz, 2H, H-Pr2), 1.25 (d, J = 6.7 Hz, 3H, H-6''). 13C NMR (126 MHz, D2O) δ 174.2 (C=O), 101.8 (C-1'), 100.9 (C-1), 98.4 (C-1''), 93.2 (d, J = 178 Hz,C-4''), 75.2 (C-5), 74.9 (C-3, C-5'), 73.3 (C-4), 72.4 (C-3'), 71.1 (C-2'), 68.2 (C-4'), 68.0 (d, J = 18 Hz, C-3''), 67.7 (d, J = 3 Hz, C-2''), 67.2 (C-Pr1), 65.7 (d, J = 18 Hz, C-5''), 61.4 (C-6'), 59.7 (C-6), 55.8 (C-2), 47.7 (C-Pr3), 28.1 (C-Pr2), 22.2 (C-Ac), 14.6 (d, J = 5 Hz, C-6''). 19F NMR (376 MHz, Deuterium Oxide) δ -220.86 (dt, J = 50.5, 31.2 Hz, F-4''). HRMS: Found 637.2362 ([M+Na]+), $C_{23}H_{39}FN_4O_{14}Na^+$ requires 637.2339.

1.2.13 Gal β1-4 (6F-Fuc α1-3) GlcNAc-N_3 (LeX13)

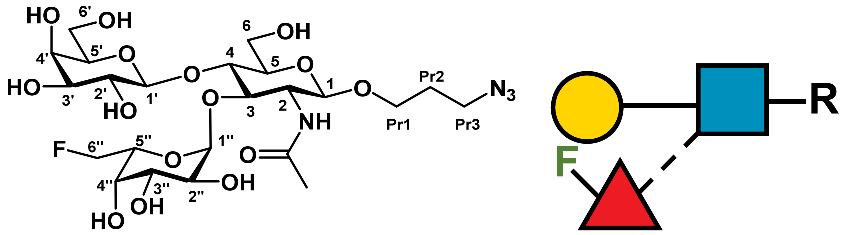

GlcNAc-N_3 (10 mM), UDP-Gal (11 mM), $MnCl_2$ (10 mM), BSA (1 mg / ml), B4GalT1 HS (30 μM), Tris buffer (100 mM, pH 8.0) in H_2O at a total volume of 3.25 ml were incubated overnight at 37 °C. To the crude Gal β1-4 GlcNAc-N3 (4 mM) was added Tris, pH 7.0 (200 mM), $MgCl_2$ (10 mM), ATP (16 mM), GTP (8 mM), 6F-Fuc (8 mM), α1-3 FucT HP (8 uM) and FKP (5 uM) in H_2O at a total volume of 8.12 ml this was incubated overnight at 37 °C. The reaction mixture was passed through a 10K MWCO spin concentrator and the filtrate was dried under reduced pressure with 600 mg silica gel. The resulting powder was loaded into a 4 g empty dry load cartridge and connected to a 12 g flash silica cartridge. The separation was run on a biotage flash system (20:80 MeOH:EtOAc → 50:50 MeOH:EtOAc over 15 CV). The fractions containing the product were pooled, the solvent removed under reduced pressure and re-dissolved in water. The mixture was then finally purified by size exclusion on a 10/300 biogel P2 column and the fractions containing product pooled and freeze dried to give the product as a white powder. (10.5 mg, 51%). 1H NMR (501 MHz, Deuterium Oxide) δ 5.24 (d, J = 3.9 Hz, 1H, H-1''), 5.09 (ddd, J = 17.4, 8.2, 3.2 Hz, 1H, H-5''), 4.67 (ddd, J = 45.4, 10.2, 3.2 Hz, 1H, H-6a''), 4.63 (ddd, J = 49.0, 10.3, 7.9 Hz, 1H, H-6b''), 4.54 (m, 1H, H-1), 4.46 (d, J = 7.5 Hz, 1H, H-1''), 4.02 (d, J = 2.5 Hz, 1H, H-4''), 4.00 (dd, J = 10.2, 2.0 Hz, 1H, H-6a), 3.97 (dt, J = 10.6, 5.4 Hz, 1H, H-Pr1a), 3.92 (m, 5H, H-2, H-3, H-3'', H-4, H-4'), 3.87 (dd, J = 12.3, 4.8 Hz, 1H, H-6b), 3.75 (dd, J = 10.4, 4.1 Hz, 1H, H-2''), 3.76 – 3.73 (m, 2H, H-6a', H-6b'), 3.71 – 3.63 (m, 2H, H-3', H-Pr1b), 3.63 – 3.56 (m, 3H, H-2', H-5, H-5'), 3.38 (td, J = 6.5, 4.6 Hz, 2H, H-Pr3), 2.06 (s, 3H, H-Ac), 1.85 (p, J = 6.4 Hz, 2H, H-Pr2). 13C NMR (126 MHz, D2O) δ 174.2 (C=O), 102.1 (C-1'), 100.9 (C-1), 98.6 (C-1''), 84.1 (d, J = 165 Hz, C-6''), 75.3 (C-5'), 74.9 (C-5), 74.8 (C-3), 73.6 (C-4), 72.5 (C-3'), 70.8 (d, J = 3 Hz, C-2'), 69.3 (d, J = 19 Hz, C-5''), 69.1 (d, J = 8 Hz, C-4''), 68.6 (C-3''), 68.3 (C-4'), 67.7 (C-2''), 67.2 (C-Pr1), 61.4 (C-6'), 59.7 (C-6), 55.8 (C-2), 47.7 (C-Pr3), 28.1 (C-Pr2), 22.2 (C-Ac). 19F NMR (376 MHz, Deuterium Oxide) δ -229.53 (dt, J = 47.0, 17.0 Hz, F-6''). HRMS: Found ([M+Na]+) 653.2309, $C_{23}H_{38}F_2N_4O_{14}Na^+$ requires 653.2288.

1.2.14 Gal β1-4 (Ara α1-3) GlcNAc-N3 (**LeX14**)

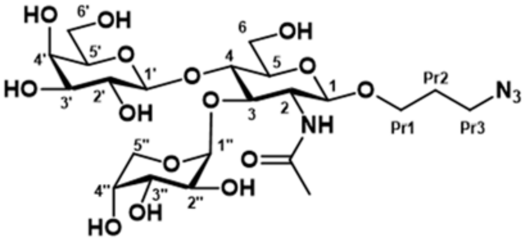

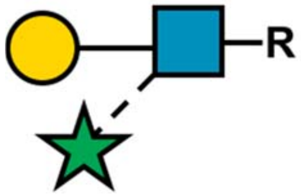

GlcNAc-N3 (SG17011-03, 10 mM), UDP-6F-Gal (11 mM), MnCl2 (10 mM), BSA (1 mg / ml), B4GalT1 HS (30 mM), Tris buffer (100 mM, pH 8.0) in H2O at a total volume of 6.5 ml were incubated overnight at 37 °C. To the crude Gal β1-4 GlcNAc-N3 (4 mM) was added Tris, pH 7.0 (200 mM), MgCl2 (10 mM), ATP (16 mM), GTP (8 mM), Ara (8 mM), α1-3 FucT HP (8 uM) and FKP (5 uM) in H2O at a total volume of 16.25 ml this was incubated overnight at 37 °C. The reaction mixture was precipitated by adding EtOH (16.25 ml) and the solid removed by centrifugation, the filtrate was subsequently dried under reduced pressure with 600 mg silica gel. The resulting powder was loaded into a 4 g empty dry load cartridge and connected to a 12 g flash silica cartridge. The separation was run on a biotage flash system (30:70 MeOH:EtOAc → 50:50 MeOH:EtOAc over 15 CV). The fractions containing the product were pooled, the solvent removed under reduced pressure and re-dissolved in water. The mixture was then finally purified by size exclusion on a 26/1000 biogel P2 column (20 mM ammonium formate) and the fractions containing product pooled and freeze dried to give a white powder (5.2 mg, 13%). 1H NMR (501 MHz, Deuterium Oxide) δ 5.17 (d, J = 3.8 Hz, 1H, H-1''), 4.64 (d, J = 12.8 Hz, 1H, H-5ax''), 4.52 (d, J = 7.8 Hz, 1H, H-1), 4.47 (d, J = 7.8 Hz, 1H, H-1'), 4.02 – 3.83 (m, 9H, H-2, H-3, H-3'', H-4, H-4', H-4'', H-6a, H-6b, H-Pr1a), 3.77 – 3.70 (m, 3H, H-2'', H-6a', H-6b'), 3.70 – 3.65 (m, 1H, H-Pr1b), 3.66 (dd, J = 9.9, 3.3 Hz, 1H, H-3'), 3.60 (dd, J = 7.5, 4.4 Hz, 1H, H-5'), 3.58 (ddd, J = 9.8, 4.8, 2.3 Hz, 1H, H-5), 3.54 – 3.49 (m, 2H, H-2', H-5eq''), 3.37 (td, J = 6.5, 4.4 Hz, 2H, H-Pr3), 2.04 (s, 3H, H-Ac), 1.83 (dd, J = 7.0, 5.6 Hz, 2H, H-Pr2). 13C NMR (126 MHz, D2O) δ 171.0 (C=O), 101.8 (C-1'), 101.0 (C-1), 98.9 (C-1''), 75.3 (C-5'), 75.2 (C-5), 75.0 (C-3), 73.3 (C-4), 72.4 (C-3'), 71.0 (C-2'), 69.0 (C-4''), 68.4 (C-3''), 68.3 (C-4'), 68.0 (C-2''), 67.2 (C-Pr1), 63.6 (C-5''), 61.4 (C-6'), 59.8 (C-6), 55.7 (C-2), 47.7 (C-Pr3), 28.1 (C-Pr2), 22.2 (C-Ac). HRMS: Found 621.2241 ([M+Na]$^+$), $C_{22}H_{38}N_4O_{15}Na^+$ requires 621.2226.

1.2.15 3F-Gal β1-4 (6F-Fuc α1-3) GlcNAc-N3 (**LeX15**)

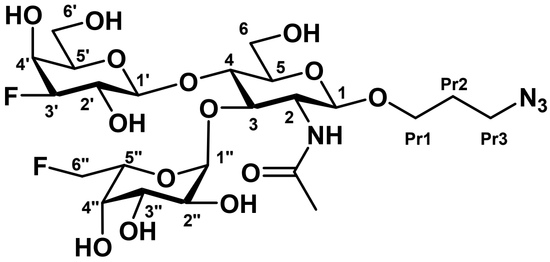

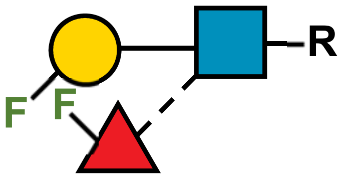

GlcNAc-N3 (SG17011-03, 10 mM), UDP-3F-Gal (11 mM), MnCl2 (10 mM), BSA (1 mg / ml), B4GalT1 HS (30 ?M), Tris buffer (100 mM, pH 8.0) in H2O at a total volume of 3.25 ml were incubated overnight at 37 °C. To the crude 3F-Gal β1-4 GlcNAc-N3 (4 mM) was added Tris, pH 7.0 (200 mM), MgCl2 (10 mM), ATP (16 mM), GTP (8 mM), 6F-Fuc (SG18010-2, 8 mM), α1-3 FucT HP (8 uM) and FKP (5 uM) in H2O at a total volume of 8.12 ml this was incubated overnight at 37 °C. The reaction mixture was passed through a 10K MWCO spin concentrator and the filtrate was dried under reduced pressure with 600 mg silica gel. The resulting powder was loaded into a 4 g empty dry load cartridge and connected to a 12 g flash silica cartridge. The separation was run on a biotage flash system (30:70 MeOH:EtOAc → 50:50 MeOH:EtOAc over 15 CV). The fractions containing the product were pooled, the solvent removed under reduced pressure and re-dissolved in water. The mixture was then finally purified by size exclusion on a 26/1000 biogel P2 column and the fractions containing product pooled and freeze dried to give the product as a

white powder. (2.4 mg, 16%). 1H NMR (501 MHz, Deuterium Oxide) δ 5.22 (d, J = 3.9 Hz, 1H, H-1'), 5.07 (ddd, J = 17.2, 8.0, 3.2 Hz, 1H, H-5''), 4.64 (ddd, J = 45.0, 10.3, 3.3 Hz, 1H, H-6a''), 4.58 (ddd, J = 48.5, 10.2, 2.9 Hz, 1H, H-6b''), 4.57 – 4.51 (m, 2H, H-3', H-1'), 4.50 (d, J = 7.9 Hz, 1H, H-1), 4.20 (dd, J = 6.6, 3.5 Hz, 1H, H-4'), 4.01 – 3.83 (m, 9H, H-2, H-2', H-3, H-3'', H-4, H-4'', H-6a, H-6b, H-Pr1a), 3.78 – 3.69 (m, 2H, H-2'', H-6a', H-6b'), 3.66 (dt, J = 10.4, 6.3 Hz, 1H, H-Pr1b), 3.63 – 3.56 (m, 2H, H-5, H-5'), 3.43 – 3.31 (m, 2H, H-Pr3), 2.04 (s, 3H, H-Ac), 1.83 (p, J = 6.4 Hz, 2H, H-Pr2). 13C NMR (126 MHz, D2O) δ 101.2 (C-1, C-1'), 98.6 (C-1''), 92.8 (d, C-3'), 84.9 (d, C-6''), 74.6 (C-5'), 74.4 (C-2', C-3, C-5), 73.5 (C-4), 69.4 (C-4''), 69.2 (C-5''), 68.9 (C-3''), 68.0 (C-2''), 68.7 (C-4'), 67.1 (C-Pr1), 61.0 (C-6'), 59.8 (C-6), 55.9 (C-2), 47.7 (C-Pr3), 28.1 (C-Pr2), 22.1 (C-Ac). C=O peak not observed. 19F NMR (376 MHz, D2O) δ -198.33 (ddd, J = 47.31, 12.36, 5.53 Hz, F-3'), -229.28 (td, J = 45.9, 17.2 Hz, F-6''). HRMS: Found 633.2433 ([M+H]+), C23H40FN4O14+ requires 633.2425.

1.2.16 4F-Gal β1-4 (6F-Fuc α1-3) GlcNAc-N_3 (LeX16)

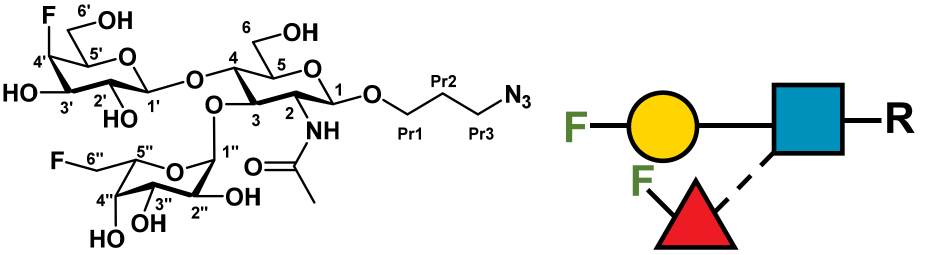

GlcNAc-N_3 (10 mM), UDP-4F-Gal (11 mM), $MnCl_2$ (10 mM), BSA (1 mg / ml), B4GalT1 HS (30 μM), Tris buffer (100 mM, pH 8.0) in H_2O at a total volume of 3.25 ml were incubated overnight at 37 °C. To the crude 4F-Gal β1-4 GlcNAc-N3 (4 mM) was added Tris, pH 7.0 (200 mM), $MgCl_2$ (10 mM), ATP (16 mM), GTP (8 mM), 6F-Fuc (8 mM), α1-3 FucT HP (8 uM) and FKP (5 uM) in H_2O at a total volume of 8.12 ml this was incubated overnight at 37 °C. The reaction mixture was passed through a 10K MWCO spin concentrator and the filtrate was dried under reduced pressure with 600 mg silica gel. The resulting powder was loaded into a 4 g empty dry load cartridge and connected to a 12 g flash silica cartridge. The separation was run on a biotage flash system (20:80 MeOH:EtOAc → 50:50 MeOH:EtOAc over 15 CV). The fractions containing the product were pooled, the solvent removed under reduced pressure and re-dissolved in water. The mixture was then finally purified by size exclusion on a 10/300 biogel P2 column and the fractions containing product pooled and freeze dried to give the product as a white powder. (2.4 mg, 15%). 1H NMR (501 MHz, Deuterium Oxide) δ 5.12 (d, J = 4.0 Hz, 1H, H-1''), 4.90 (ddd, J = 17.0, 8.1, 3.9 Hz, 1H, H-5''), 4.71 (dd, J = 50.6, 3.2 Hz, 1H, H-4'), 4.59 – 4.40 (m, 2H, H-6a'', H-6b''), 4.43 (d, J = 7.3 Hz, 1H, H-1'), 4.42 (d, J = 7.3 Hz, 1H, H-1), 3.89 (dd, J = 12.3, 2.3 Hz, 1H, H-6a), 3.87 – 3.79 (m, 5H, H-2, H-3, H-3'', H-4, H-4''), 3.75 (dd, J = 12.2, 4.7 Hz, 1H, H-6b), 3.66 (ddd, J = 31.6, 10.0, 2.7 Hz, 1H, H-3'), 3.67 – 3.59 (m, 4H, H-2'', H-5', H-6a', H-6b'), 3.59 – 3.46 (m, 3H, H-2', H-5, H-Pr1a), 3.26 (td, J = 6.5, 4.8 Hz, 2H, H-Pr3), 1.94 (s, 3H, H-Ac), 1.73 (h, J = 6.0, 5.5 Hz, 2H, H-Pr2). 13C NMR (126 MHz, D2O) δ 174.3 (C=O), 101.3 (C-1, C-1'), 98.5 (C-1''), 89.0 (d, C-4'), 83.8 (d, C-6''), 75.0 (C-5), 74.1 (C-3, C-4), 72.8 (C-5'), 71.2 (d, C-3'), 70.9 (C-2'), 69.1 (C-5''), 68.8 (C-4''), 68.3 (C-3''), 67.6 (C-2''), 67.1 (C-Pr1), 59.9 (C-6'), 59.5 (C-6), 55.9 (C-2), 47.6 (C-Pr3), 28.1 (C-Pr2), 22.0 (C-Ac). HRMS: Found ([M+Na]+) 709.1974, $C_{23}H_{38}F_2N_4O_{14}Na^+$ requires 709.1962. 19F NMR (376 MHz, D2O) δ -218.18 (td, J = 50.6, 31.1 Hz, F-4'), -229.74 (td, J = 47.0, 14.5 Hz, F-6'').

1.2.17 6F-Gal β1-4 (Fuc α1-3) 6F-GlcNAc-N_3 (LeX17)

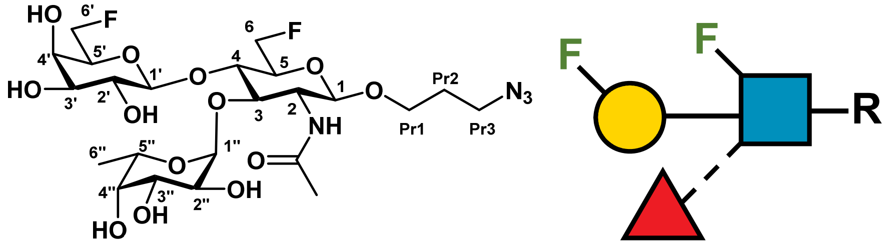

6F-GlcNAc-N3 (SG16131-01, 10 mM), UDP-6F-Gal (11 mM), MnCl2 (10 mM), BSA (1 mg / ml), B4GalT1 HS (30 □M), Tris buffer (100 mM, pH 8.0) in H2O at a total volume of 3.25 ml were incubated overnight at 37 °C. To the crude 6F-Gal β1-4 6F-GlcNAc-N3 (4 mM) was added Tris, pH 7.0 (200 mM), MgCl2 (10 mM), ATP (16 mM), GTP (8 mM), Fuc (8 mM), α1-3 FucT HP (8 uM) and FKP (5 uM) in H2O at a total volume of 8.12 ml this was incubated overnight at 37 °C. The reaction mixture was passed through a 10K MWCO spin concentrator and the filtrate was dried under reduced pressure with 600 mg silica gel. The resulting powder was loaded into a 4 g empty dry load cartridge and connected to a 12 g flash silica cartridge. The separation was run on a biotage flash system (30:70 MeOH:EtOAc → 50:50 MeOH:EtOAc over 15 CV). The fractions containing the product were pooled, the solvent removed under reduced pressure and re-dissolved in water. The mixture was then finally purified by size exclusion on a 26/1000 biogel P2 column and the fractions containing product pooled and freeze dried to give the product as a white powder. (2.4 mg, 16%). 19F NMR (376 MHz, Deuterium Oxide) δ -232.56 (dt, J = 47.3, 31.2 Hz, F-6), -230.04 (dt, J = 46.8, 14.2 Hz, F-6'). LCMS: Found 617.06 ([M+H]+), C23H38F2N4O13+ requires 617.2476. 1H NMR (501 MHz, Deuterium Oxide) δ 5.10 (d, J = 4.0 Hz, 1H, H-1''), 4.87 (q, J = 7.0 Hz, 1H, H-5''), 4.80 (ddd, J = 47.1, 11.1, 2.4 Hz, 1H, H-6a), 4.88 (ddd, J = 47.1, 11.1, 4.8 Hz, 1H, H-6b), 4.63 (ddd, J = 45.3, 9.7, 4.2 Hz, 1H, H-6a'), 4.56 (ddd, J = 45.3, 7.0, 4.2 Hz, 1H, H-6b'), 4.56 (d, J = 9.2 Hz, 1H, H-1), 4.46 (d, J = 7.8 Hz, 1H, H-1'), 4.01 – 3.94 (m, 2H, H-4, H-Pr1a), 3.91 – 3.85 (m, 5H, H-2, H-3, H-3'', H-4', H-5'), 3.80 – 3.78 (m, 1H, H-4''), 3.76 – 3.64 (m, 4H, H-2'', H-3', H-5, H-Pr1b), 3.52 (dd, J = 7.8, 9.9 Hz, 1H, H-2'), 3.42 – 3.31 (m, 2H, H-Pr3), 2.04 (s, 3H, H-Ac), 1.83 (p, J = 6.5 Hz, 1H, H-Pr2), 1.17 (d, J = 6.7 Hz, 3H, H-6'').13C NMR (126 MHz, D2O) δ 101.9 (C-1'), 101.2 (C-1), 98.6 (C-1''), 82.9 (C-6'), 80.1 (C-6), 74.0 (C-3), 73.8 (C-5), 73.4 (C-4), 73.9 (C-5'), 73.2 (C-3'), 71.8 (C-4''), 71.2 (C-2'), 69.5 (C-4'), 69.5 (C-3''), 68.5 (C-2''), 66.9 (C-5''), 67.1 (C-Pr1), 54.6 (C-2), 47.7 (C-Pr3), 28.1 (C-Pr2), 22.2 (C-Ac), 15.1 (C-6''). C=O peak not observed. 19F NMR (376 MHz, Deuterium Oxide) δ -232.56 (dt, J = 47.3, 31.2 Hz, F-6), -230.04 (dt, J = 46.8, 14.2 Hz, F-6'). LRMS: Found 617.2481 ($[M+H]^+$), $C_{23}H_{38}F_2N_4O_{13}^+$ requires 617.2477.

1.2.18 Gal β1-4 (3F-Fuc α1-3) 6F-GlcNTFA-N_3 (LeX18)

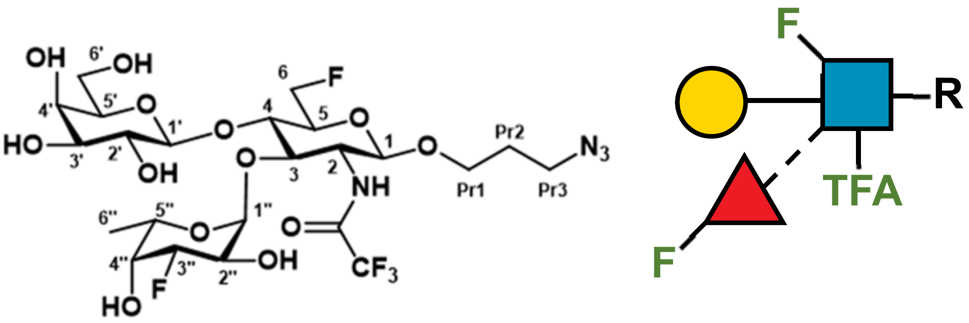

6F-GlcNTFA-N3 (SG18030-01, 10 mM), UDP-6d-Gal (11 mM), MnCl2 (10 mM), BSA (1 mg / ml), B4GalT1 HS (30 mM), Tris buffer (100 mM, pH 8.0) in H2O at a total volume of 6.5 ml were incubated overnight at 37 °C. To the crude Gal β1-4 6F-GlcNTFA-N3 (4 mM) was added Tris, pH 7.0 (200 mM), MgCl2 (10 mM), ATP (16 mM), GTP (8 mM), 3F-Fuc (SG18030, 8 mM), α1-3 FucT HP (8 uM) and FKP (5 uM) in H2O at a total volume of 16.25 ml this was incubated overnight at 37 °C. The reaction mixture was precipitated by adding EtOH (16.25 ml) and the solid removed by centrifugation, the filtrate was subsequently dried under reduced pressure with 600 mg silica gel. The resulting powder was loaded into a 4 g empty dry load cartridge and connected to a 12 g flash silica cartridge. The separation was run on a biotage flash system (30:70 MeOH:EtOAc → 50:50 MeOH:EtOAc over 15 CV). The fractions containing the product were pooled, the solvent removed under reduced pressure and re-dissolved in water. The mixture was then finally purified by size exclusion on a 26/1000 biogel P2 column (20 mM ammonium formate) and the fractions containing product pooled and freeze dried to give a white powder (8 mg, 18%). 1H NMR (501 MHz, Deuterium Oxide) δ 5.11 (t, J = 4.3 Hz, 1H, H-1''), 4.80 (m, 4H, H-3'', H-5'', H-6a, H-6b), 4.66 (d, J = 8.5 Hz, 1H, H-1), 4.45 (dd=, J = 7.8, 1H, H-1'), 4.10 – 4.04 (m, 3H, H-2, H-4, H-4''), 4.02 –

3.95 (m, 3H, H-2'', H-3, H-Pr1a), 3.92 (d, J = 3.3, 1H, H-4'), 3.81 – 3.73 (m, 3H, H-5, H-6a', H-6b'), 3.73 – 3.66 (m, 2H, H-3', H-Pr1b), 3.63 (dd, J = 7.6, 4.4 Hz, 1H, H-5'), 3.52 (dd, J = 9.8, 7.8 Hz, 1H, H-2'), 3.37 (td, J = 6.6, 2.8 Hz, 2H, H-Pr3), 1.91 – 1.80 (m, 2H, H-Pr2), 1.22 (d, J = 6.6 Hz, 3H, H-6''). 13C NMR (126 MHz, D2O) δ 159.2 (C=O), 101.9 (C-1'), 100.4 (C-1), 99.0 (d, J = 11 Hz, C-1''), 90.8 (d, J = 181 Hz, C-3''), 81.1 (d, J = 167 Hz, C-6), 74.9 (C-5'), 74.5 (C-3), 73.8 (d, J = 17 Hz, C-5), 72.5 (d, J = 5 Hz, C-4), 72.4 (C-3'), 71.0 (C-2'), 70.2 (d, J = 16 Hz, C-4''), 68.3 (C-4'), 67.5 (C-Pr1), 66.4 (d, J = 7 Hz, C-5''), 65.9 (d, J = 19 Hz, C-2''), 61.4 (C-6'), 56.12 (C-2), 47.7 (C-Pr3), 28.0 (C-Pr2), 15.1 (C-6''). 19F NMR (376 MHz, Deuterium Oxide) δ -75.63 (s, F-CF_3), -202.49 (d, J = 50.0 Hz, F-3''), -232.57 (dt, J = 47.3, 31.2 Hz, F-6). HRMS: Found 693.2032 ($[M+Na]^+$), $C_{23}H_{35}F_5N_4O_{13}Na^+$ requires 693.2013.

1.2.19 Gal β1-4 (3F-Fuc α1-3) 6,6-diFGlcNTFA-N_3 (LeX19)

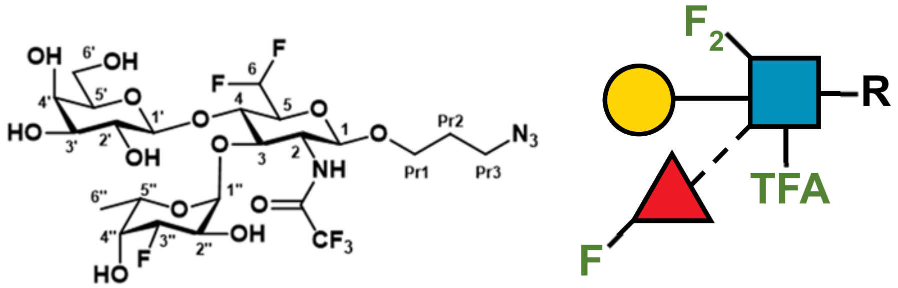

6,6-diFGlcNTFA-N3 (SG16251-01, 10 mM), UDP-6d-Gal (11 mM), MnCl2 (10 mM), BSA (1 mg / ml), B4GalT1 HS (30 mM), Tris buffer (100 mM, pH 8.0) in H2O at a total volume of 6.5 ml were incubated overnight at 37 °C. To the crude Gal β1-4 6,6-diFGlcNTFA-N3 (4 mM) was added Tris, pH 7.0 (200 mM), MgCl2 (10 mM), ATP (16 mM), GTP (8 mM), 3F-Fuc (SG18030, 8 mM), α1-3 FucT HP (8 uM) and FKP (5 uM) in H2O at a total volume of 16.25 ml this was incubated overnight at 37 °C. The reaction mixture was precipitated by adding EtOH (16.25 ml) and the solid removed by centrifugation, the filtrate was subsequently dried under reduced pressure with 600 mg silica gel. The resulting powder was loaded into a 4 g empty dry load cartridge and connected to a 12 g flash silica cartridge. The separation was run on a biotage flash system (30:70 MeOH:EtOAc → 50:50 MeOH:EtOAc over 15 CV). The fractions containing the product were pooled, the solvent removed under reduced pressure and re-dissolved in water. The mixture was then finally purified by size exclusion on a 26/1000 biogel P2 column (20 mM ammonium formate) and the fractions containing product pooled and freeze dried. Impurities still remained therefore a final purification step was performed using a C8 HPLC column (5:95 MeCN → 50:50 MeCN:H2O, 10 min) the fractions were lyophilised to give a white powder (1.4 mg, 3%). 1H NMR (400 MHz, Deuterium Oxide) δ 6.32 (t, J = 52.9 Hz, 1H, H-6), 5.11 (t, J = 4.3 Hz, 1H, H-1''), 4.87 – 4.70 (m, 2H, H-3'', H-5''), 4.72 (d, J = 8.3 Hz, 1H, H-1), 4.44 (d, J = 7.8 Hz, 1H, H-1'), 4.15 (t, J = 9.3 Hz, 1H, H-4), 4.13 (t, J = 9.3 Hz, 1H, H-2), 4.09 – 3.94 (m, 5H, H-2'', H-3, H-4'', H-5, H-Pr1a), 3.92 (d, J = 3.4 Hz, 1H, H-4'), 3.79 – 3.61 (m, 5H, H-3', H-5', H-6a', H-6b', H-Pr1b), 3.51 (dd, J = 9.8, 7.7 Hz, 1H, H-2'), 3.40 – 3.33 (m, 2H, H-Pr3), 1.85 (p, J = 6.3 Hz, 2H, H-Pr2), 1.22 (d, J = 6.6 Hz, 3H, H-6''). 13C NMR (126 MHz, D2O) δ 112.5 (t, C-6), 101.8 (C-1''), 100.6 (C-1), 99.0 (C-1'), 90.7 (d, C-3''), 75.0 (C-5'), 74.2 (C-5), 73.2 (C-3), 73.1 (C-4), 72.3 (C-3'), 70.9 (C-2'), 70.1 (C-4''), 68.3 (C-4'), 67.8 (C-Pr1), 66.5 (C-5'', d), 65.8 (C-2'', d), 61.3 (C-6'), 55.8 (C-2), 47.6 (C-Pr3), 28.0 (C-Pr2), 15.1 (C-6''). C=O peak not observed.NMR (376 MHz, Deuterium Oxide) δ -75.63 (s, F-CF_3), -132.33 (m, F-6a, F-6b), -202.53 (d, J = 51.7 Hz, F-3''). 19F{1H} NMR (376 MHz, Deuterium Oxide) δ -75.57 (s, CF_3), -132.34 (s, F-6a, F-6b), -202.67 (s, F-3''). HRMS: Found 711.1933 ($[M+Na]^+$), $C_{23}H_{34}F_6N_4O_{13}Na^+$ requires 711.1919.

1.2.20 Gal β1-4 (4F-Fuc α1-3) 6F-GlcNTFA-N_3 (**LeX20**)

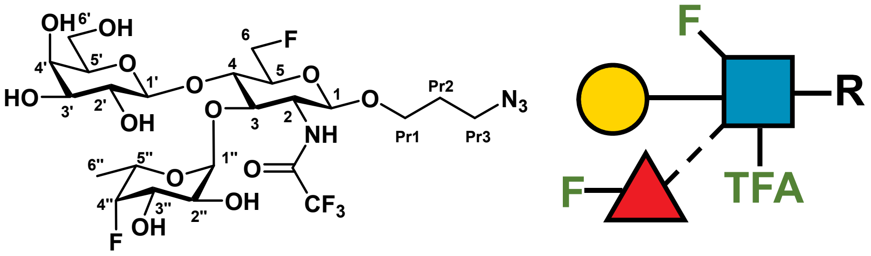

Gal β1-4 6F-GlcNTFA-N3 (SG16231-02, 2 mM), Tris, pH 7.4 (50 mM), MgCl2 (10 mM), ATP (8 mM), GTP (4 mM), 4F-Fuc (4 mM), α1-3 FucT HP (4 uM) and FKP (2.4 uM) in H2O at a total volume of 6.52 ml were incubated overnight at 37 °C. The reaction mixture was passed through a 10K MWCO spin concentrator and the filtrate was lyophilised. The powder was re-dissolved in 500 ul of water, 500 mg of silica was added and the water removed in vacuo. The resulting powder was loaded into a 4 g empty dry load cartridge and connected to a 4 g flash silica cartridge. The separation was run on a biotage flash system (0:100 MeOH:EtOAc → 50:50 MeOH:EtOAc over 15 CV). The fractions containing the product were pooled, the solvent removed under reduced pressure and re-dissolved in water. The mixture was then finally purified by size exclusion on a 10/300 biogel P2 column and the fractions containing product pooled and freeze dried to give the product as a white powder (?). 1H NMR (501 MHz, CDCl3) δ 5.14 (d, J = 3.9 Hz, 1H, H-1''), 5.00 (dq, J = 33.7, 6.7 Hz, 1H, H-5''), 5.00 – 4.81 (m, 3H, H-6a, H-6b, H-4''), 4.72 (d, J = 8.5 Hz, 1H, H-1), 4.50 (d, J = 7.7 Hz, 1H, H-1'), 4.15 – 4.09 (m, 2H, H-2, H-4), 4.09 – 3.98 (m, 3H, H-3, H-3'', H-Pr1a), 3.97 (d, J = 3.4 Hz, 1H, H-4'), 3.86 – 3.76 (m, 3H, H-2'', H-5, H-6'), 3.76 – 3.70 (m, 2H, H-3', H-5'), 3.70 – 3.65 (m, 1H, H-5'), 3.55 (dd, J = 9.8, 7.8 Hz, 1H, H-2'), 3.42 (qd, J = 6.7, 5.9, 2.7 Hz, 2H, H-Pr3), 1.91 (ddd, J = 8.8, 6.1, 3.2 Hz, 4H, H-Pr2), 1.32 (d, J = 6.7 Hz, 3H, H-6''). 13C NMR (126 MHz, D2O) δ 101.8 (C-1'), 100.4 (C-1), 98.9 (C-1''), 80.7 (d, C-6), 75.1 (C-5'), 74.4 (C-3), 73.7 (C-5), 73.0 (C-4), 72.3 (C-3'), 70.9 (C-2'), 68.0 (C-3'', C-4'), 67.4 (C-2''), 66.9 (C-Pr1), 66.7 (C-4''), 66.0 (d, C-5''), 61.7 (C-6'), 56.1 (C-2), 47.4 (C-Pr3), 28.1 (C-Pr2), 14.6 (C-6''). C=O peak not observed. 19F NMR (376 MHz, D2O) δ -75.57 (s, CF_3), -220.89 (F-4''), -232.59 (F-6). HRMS: Found 693.2006 ($[M+Na]^+$), $C_{23}H_{35}F_5N_4NaO_{13}^+$ requires 693.2013

1.2.21 Gal β1-4 (6F-Fuc α1-3) 6F-GlcNTFA-N_3 (**LeX21**)

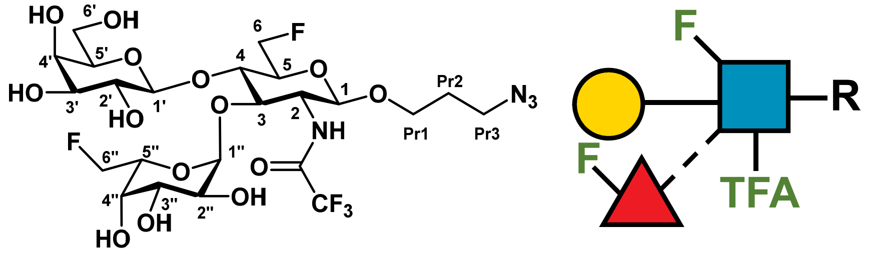

6FGlcNTA-N_3 (10 mM), UDP-Gal (11 mM), $MnCl_2$ (10 mM), BSA (0.5 mg / ml), B4GalT1 HS (20 μM), Tris buffer (50 mM, pH 8.0) and NaCl (100 mM) in H_2O at a total volume of 1.3 ml were incubated overnight at 37 °C. The reaction mixture was passed through a 10K MWCO spin concentrator and used directly in the next reaction. Gal β1-4 6F-GlcNTFA-N3 (2 mM), Tris, pH 7.4 (50 mM), $MgCl_2$ (10 mM), ATP (8 mM), GTP (4 mM), 6F-Fuc (4 mM), α1-3 FucT HP (4 uM) and FKP (2.4 uM) in H_2O at a total volume of 6.52 ml were incubated overnight at 37 °C. The reaction mixture was passed through a 10K MWCO spin concentrator and the filtrate was lyophilised. The powder was re-dissolved in 500 ul of water, 500 mg of silica was added and the water removed in vacuo. The resulting powder was loaded into a 4 g empty dry load cartridge and connected to a 4 g flash silica cartridge. The separation was run on a biotage flash system (0:100 MeOH:EtOAc → 50:50 MeOH:EtOAc over 15 CV). The fractions containing the product were pooled, the solvent removed under reduced pressure and re-dissolved in water. The mixture was then finally purified by size exclusion on a 10/300 biogel P2 column and the fractions containing product pooled and

freeze dried to give the product as a white powder (2.7 mg, 36%). ^{1}H NMR (501 MHz, Deuterium Oxide) δ 5.16 (d, *J* = 3.9 Hz, 1H, H-1''), 5.16 – 5.09 (m, 1H, H-5''), 4.90 (ddd, *J* = 45.7, 11.2, 2.4 Hz, 1H, H-6a), 4.85 – 4.77 (m, 1H, H-6b), 4.77 – 4.68 (m, 1H, H-6a''), 4.67 (d, *J* = 8.1 Hz, 1H, H-1), 4.60 (ddd, *J* = 35.6, 10.0, 7.7 Hz, 1H, H-6b''), 4.44 (d, *J* = 6.6 Hz, 1H, H-1'), 4.11 – 4.03 (m, 3H, H-2, H-3, H-4), 4.02 (d, *J* = 4.0 Hz, 1H, H-4''), 3.99 (dt, *J* = 10.4, 4.4 Hz, 1H, H-Xa), 3.92 (d, *J* = 3.0 Hz, 1H, H-4'), 3.90 (dd, *J* = 10.3, 3.3 Hz, 1H, H-3''), 3.81 – 3.73 (m, 3H, H-2'', H-5, H-6a'), 3.73 – 3.66 (m, 2H, H-6b', H-Xb), 3.66 – 3.61 (m, 3H, H-2', H-3', H-5'), 3.37 (td, *J* = 6.6, 2.8 Hz, 2H, H-Z), 1.85 (quin, *J* = 6.8 Hz, 2H, H-Y). ^{13}C NMR (126 MHz, D_2O) δ 102.2 (C-1'), 100.5 (C-1), 99.0 (C-1''), 84.2 (d, J = 165 Hz, C-6''), 81.1 (d, J = 169 Hz, C-6), 74.8 (C-5'), 74.5 (C-3), 73.8 (d, J = 18 Hz, C-5), 72.8 (d, J = 4 Hz, C-4), 72.5 (C-3'), 70.7 (C-2'), 69.5 (d, J = 19 Hz, C-5''), 69.1 (d, J = 8 Hz, C-4''), 68.7 (C-3''), 68.3 (C-4'), 67.6 (C-X), 67.5 (C-2''), 61.4 (C-6'), 56.2 (C-2), 47.7 (C-Z), 28.1 (C-Y). C=O peak not observed. 19F NMR (376 MHz, D2O) δ -75.59 (s, CF_3), -229.34 (td, J = 47.2, 16.4 Hz, F-6''), -232.65 (td, J = 49.6, 31.9 Hz, F-6). HRMS: Found 704.2413 ([M+NH_4]$^+$), $C_{23}H_{39}F_5N_5O_{14}^+$ requires 704.2409.

1.2.22 4F-Gal β1-4 (Fuc α1-3) 6F-GlcNTFA-N_3 (LeX22)

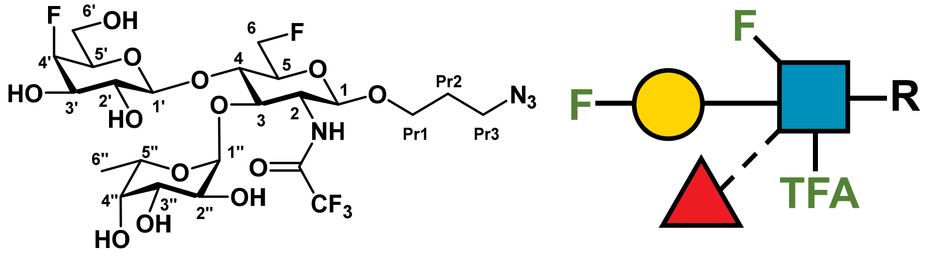

6F-GlcNTFA-N_3 (10 mM), UDP-4F-Gal (11 mM), $MnCl_2$ (10 mM), BSA (1 mg / ml), B4GalT1 HS (30 µM), Tris buffer (100 mM, pH 8.0) in H_2O at a total volume of 3.25 ml were incubated overnight at 37 °C. To the crude 4F-Gal β1-4 GlcNTFA-N3 (4 mM) was added Tris, pH 7.0 (200 mM), $MgCl_2$ (10 mM), ATP (16 mM), GTP (8 mM), Fuc (8 mM), α1-3 FucT HP (8 uM) and FKP (5 uM) in H_2O at a total volume of 8.12 ml this was incubated overnight at 37 °C. The reaction mixture was passed through a 10K MWCO spin concentrator and the filtrate was dried under reduced pressure with 600 mg silica gel. The resulting powder was loaded into a 4 g empty dry load cartridge and connected to a 12 g flash silica cartridge. The separation was run on a biotage flash system (20:80 MeOH:EtOAc → 50:50 MeOH:EtOAc over 15 CV). The fractions containing the product were pooled, the solvent removed under reduced pressure and re-dissolved in water. The mixture was then finally purified by size exclusion on a 10/300 biogel P2 column and the fractions containing product pooled and freeze dried to give the product as a white powder. (2.4 mg, 16%). 1H NMR (501 MHz, Chloroform-d) δ 5.09 (d, J = 3.9 Hz, 1H, H-1''), 5.01 – 4.78 (m, 2H, H-6a, H-6b), 4.99 (dq, J = 30.0, 6.8 Hz, 1H, H-5''), 4.84 – 4.67 (m, 1H, H-4'), 4.67 (d, J = 8.4 Hz, 1H, H-1), 4.45 (d, J = 7.8 Hz, 1H, H-1'), 4.08 (m, 2H, H-2, H-4), 4.04 – 3.93 (m, 3H, H-3, H-3'', H-Pr1a), 3.92 (d, J = 3.4 Hz, 1H, H-4''), 3.81 – 3.71 (m, 3H, H-5, H-6a', H-6b'), 3.77 (dd, J = 10.6, 4.1 Hz, 1H, H-2''), 3.71 – 3.65 (m, H-3', H-Pr1b), 3.63 (t, J = 6.1 Hz, 1H, H-5'), 3.50 (dd, J = 9.8, 7.8 Hz, 1H, H-2'), 3.37 (td, J = 6.6, 2.7 Hz, 2H, H-Pr3), 1.91 – 1.81 (m, 2H, H-Pr3), 1.27 (d, J = 6.7 Hz, 3H, H-6''). 13C NMR (126 MHz, Deuterium Oxide) δ 101.93 (C-1'), 100.5 (C-1), 98.9 (C-1''), 93.2 (d, C-4'), 81.2 (d, C-6), 75.0 (C-5'), 74.5 (C-3), 73.8 (C-5), 72.5 (C-4), 72.5 (C-3'), 71.2 (C-2'), 68.8 (d, C-3''), 68.2 (C-4''), 67.5 (C-2''), 67.2 (C-Pr1), 65.9 (C-5''), 59.2 (C-6'), 56.2 (C-2), 47.7 (C-Pr3), 28.1 (C-Pr2), 14.7 (C-6''). C=O peak not observed. 19F NMR (376 MHz, D2O) δ -75.59 (s, CF3), -220.89 (td, J = 50.6, 31.4 Hz, F-4'), -232.59 (td, J = 47.1, 30.9 Hz, F-6). HRMS: Found 693.2008 ([M+Na]$^+$), $C_{23}H_{35}F_5N_4NaO_{13}^+$ requires 693.2013.

1.2.23 6d-Gal β1-4 (Fuc α1-3) 6,6-diGlcNTFA-N_3 (LeX23)

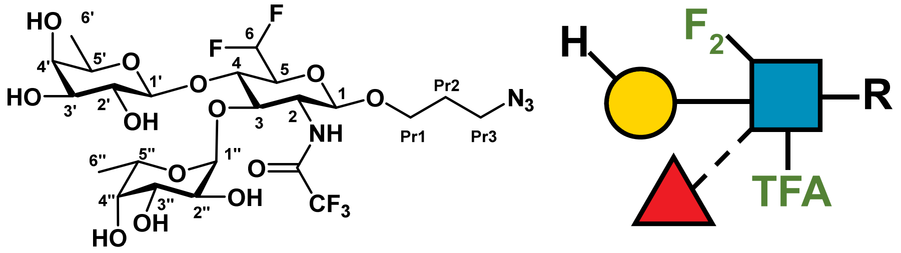

6,6-diFGlcNTFA-N3 (SG17011-03, 10 mM), UDP-6d-Gal (11 mM), MnCl2 (10 mM), BSA (1 mg / ml), B4GalT1 HS (30 µM), Tris buffer (100 mM, pH 8.0) in H2O at a total volume of 3.25 ml were incubated overnight at 37 °C. To the crude 6d-Gal β1-4 6,6-diFGlcNTFA-N3 (4 mM) was added Tris, pH 7.0 (200 mM), MgCl2 (10 mM), ATP (16 mM), GTP (8 mM), Fuc (8 mM), α1-3 FucT HP (8 uM) and FKP (5 uM) in H2O at a total volume of 8.12 ml this was incubated overnight at 37 °C. The reaction mixture was passed through a 10K MWCO spin concentrator and the filtrate was dried under reduced pressure with 600 mg silica gel. The resulting powder was loaded into a 4 g empty dry load cartridge and connected to a 12 g flash silica cartridge. The separation was run on a biotage flash system (30:70 MeOH:EtOAc → 50:50 MeOH:EtOAc over 15 CV). The fractions containing the product were pooled, the solvent removed under reduced pressure and re-dissolved in water. The mixture was then finally purified by size exclusion on a 26/1000 biogel P2 column and the fractions containing product pooled and freeze dried to give the product as a white powder. (2.4 mg, 16%).1H NMR (501 MHz, Deuterium Oxide) δ 6.33 (t, J = 52.9 Hz, 1H, H-6), 5.05 (d, J = 4.0 Hz, 1H, H-1''), 4.84 (q, J = 6.8 Hz, 1H, H-5''), 4.72 (d, J = 8.2 Hz, 1H, H-1), 4.39 (d, J = 7.8 Hz, 1H, H-1'), 4.11 – 3.95 (m, 5H, H-2, H-3, H-4, H-5, H-Pr1b), 3.93 (dd, J = 10.5, 3.3 Hz, 1H, H-3''), 3.82 (dd, J = 3.4, 1.1 Hz, 1H, H-4''), 3.75 – 3.63 (m, 5H, H-2'', H-3', H-4', H-5', H-Pr1b), 3.48 (dd, J = 9.9, 7.8 Hz, 1H, H-2'), 3.37 (td, J = 6.7, 2.1 Hz, 2H, H-Pr3), 1.90 – 1.79 (m, 2H, H-Pr2), 1.23 (d, J = 6.5 Hz, 3H, H-6'), 1.18 (d, J = 6.6 Hz, 3H, H-6''). 13C NMR (126 MHz, D2O) δ 170.9 (C=O), 114.8 (t, C-6), 101.9 (C-1'), 100.6 (C-1), 98.9 (C-1''), 74.1 (C-3), 73.5 (C-4), 72.5 (C-5), 72.0 (C-3'), 71.9 (C-4''), 70.9 (C-2'), 70.6 (C-5'), 70.4 (C-4'), 69.1 (C-3''), 67.8 (C-Pr3), 67.5 (C-2''), 66.9 (C-5''), 55.8 (C-2), 47.7 (C-Pr3), 28.01 (C-Pr2), 15.7 (C-6'), 15.3 (C-6''). 19F NMR (376 MHz, D2O) δ -75.56 (s, F-CF3), -132.42 (m, F-6a), -132.54 (m, F-6b). 19F{1H} NMR (376 MHz, Deuterium Oxide) δ -75.57 (s, CF_3), -132.04 (d, J = 285 Hz, F-6a), -132.92 (d, J = 285 Hz, F-6b). HRMS: Found 693.2008 ($[M+Na]^+$), $C_{23}H_{35}F_5N_4NaO_{13}^+$ requires 693.2013.

1.2.24 4F-Gal β1-4 (6F-Fuc α1-3) GlcNTFA-N_3 (LeX24)

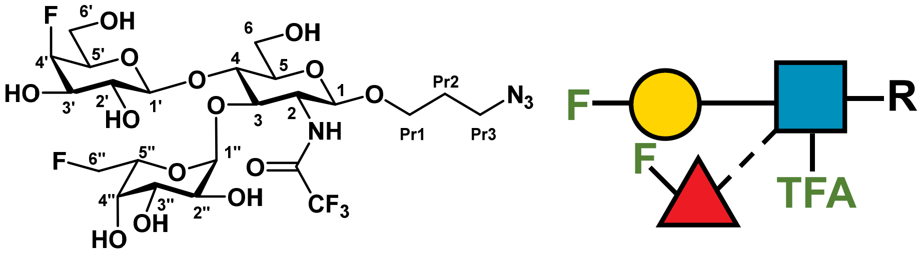

GlcNTFA-N_3 (10 mM), UDP-4F-Gal (11 mM), $MnCl_2$ (10 mM), BSA (1 mg / ml), B4GalT1 HS (30 µM), Tris buffer (100 mM, pH 8.0) in H_2O at a total volume of 3.25 ml were incubated overnight at 37 °C. To the crude 4F-Gal β1-4 GlcNTFA-N3 (4 mM) was added Tris, pH 7.0 (200 mM), $MgCl_2$ (10 mM), ATP (16 mM), GTP (8 mM), 6F-Fuc (8 mM), α1-3 FucT HP (8 uM) and FKP (5 uM) in H_2O at a total volume of 8.12 ml this was incubated overnight at 37 °C. The reaction mixture was passed through a 10K MWCO spin concentrator and the filtrate was dried under reduced pressure with 600 mg silica gel. The resulting powder was loaded into a 4 g empty dry load cartridge and connected to a 12 g flash silica cartridge. The separation was run on a biotage flash system (20:80 MeOH:EtOAc → 50:50 MeOH:EtOAc over 15 CV). The fractions containing the product were pooled, the solvent removed under reduced pressure and re-dissolved in water.

The mixture was then finally purified by size exclusion on a 10/300 biogel P2 column and the fractions containing product pooled and freeze dried to give the product as a white powder. (2.2 mg, 13%). 1H NMR (501 MHz, Deuterium Oxide) δ 5.04 (d, J = 4.0 Hz, 1H, H-1''), 4.93 (ddd, J = 17.0, 8.1, 3.9 Hz, 1H, H-5''), 4.72 – 4.65 (dd, J = 48.2, 2.8 Hz, 1H, H-4'), 4.58 – 4.40 (m, 2H, H-6a'', H-6b''), 4.50 (d, J = 8.4 Hz, 1H, H-1), 4.44 (d, J = 7.8 Hz, 1H, H-1'), 3.97 – 3.85 (m, 4H, H-2, H-3, H-4, H-Pr1a), 3.85 – 3.83 (d, J = 3.0 Hz, 1H, H-4''), 3.80 – 3.73 (m, 2H, H-3'', H-6b), 3.66 (ddd, J = 30.8, 9.7, 2.5 Hz, 1H, H-3'), 3.67 – 3.60 (m, 4H, H-2'', H-5', H-6a', H-6b'), 3.60 – 3.49 (m, 3H, H-2', H-5, H-Pr1b), 3.25 (td, J = 6.6, 2.7 Hz, 2H, H-Pr3), 1.73 (p, J = 6.4 Hz, 2H, H-Pr2). 13C NMR (126 MHz, D2O) δ 101.7 (C-1'), 100.4 (C-1), 98.9 (C-1''), 90.7 (d, C-4'), 83.9 (d, C-6''), 75.4 (C-5), 74.2 (C-3), 73.9 (C-4), 72.6 (C-5'), 71.5 (C-3'), 70.6 (C-2'), 69.2 (C-5''), 68.8 (C-4''), 68.6 (C-3''), 67.5 (C-2''), 67.4 (C-Pr1), 60.0 (C-6'), 59.5 (C-6), 56.2 (C-2), 47.6 (C-Pr3), 28.0 (C-Pr2). C=O peak not observed. 19F NMR (376 MHz, D2O) δ -75.6 (CF_3), -218.18 (F-4'), -229.59 (F-6''). HRMS: Found 709.1748 ($[M+Na]^+$), $C_{23}H_{35}F_5N_4NaO_{14}^+$ requires 709.1963.

2 Supplementary Figures

2.1 Supplementary Figure 1. Synthesis of DBCO-DH.

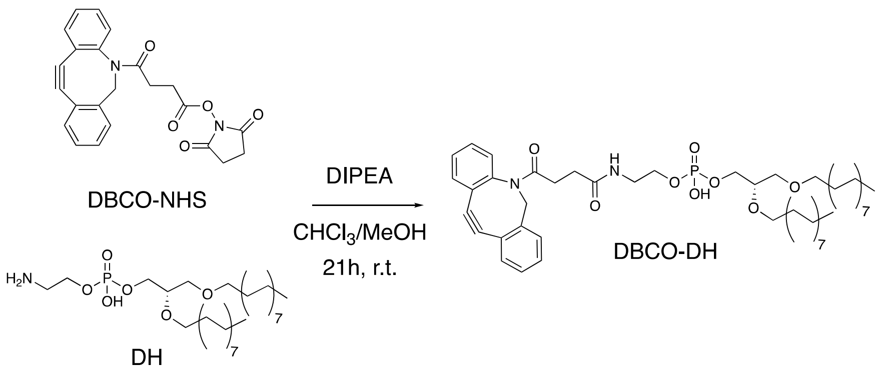

Supplementary Figure 1. Synthesis of DBCO-DH compound.

2.2 Supplementary Figure 2. Synthesis of DBCO-DH NGLs via SPAAC 'click' reaction.

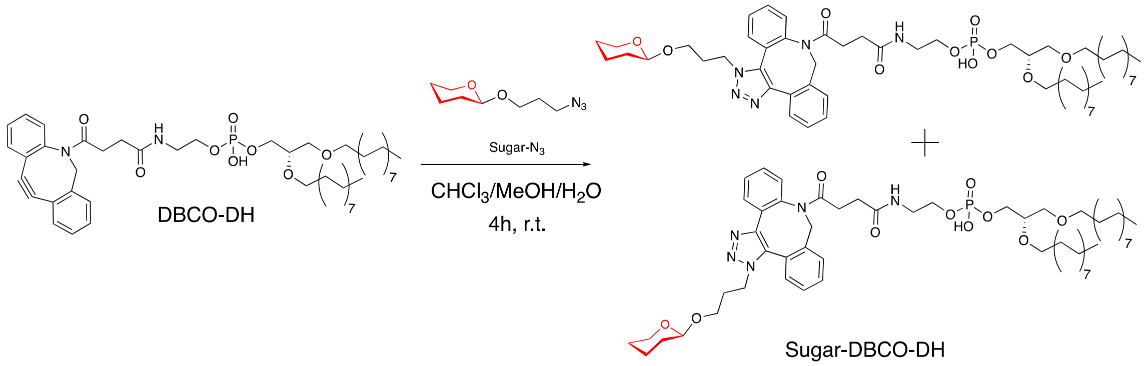

Supplementary Figure 2. Synthesis of DBCO-DH NGLs via SPAAC 'click' reaction.

2.3 Supplementary Figure 3. Crystal structure of β(1,4)-galactosyltransferase bound to UDP-Gal

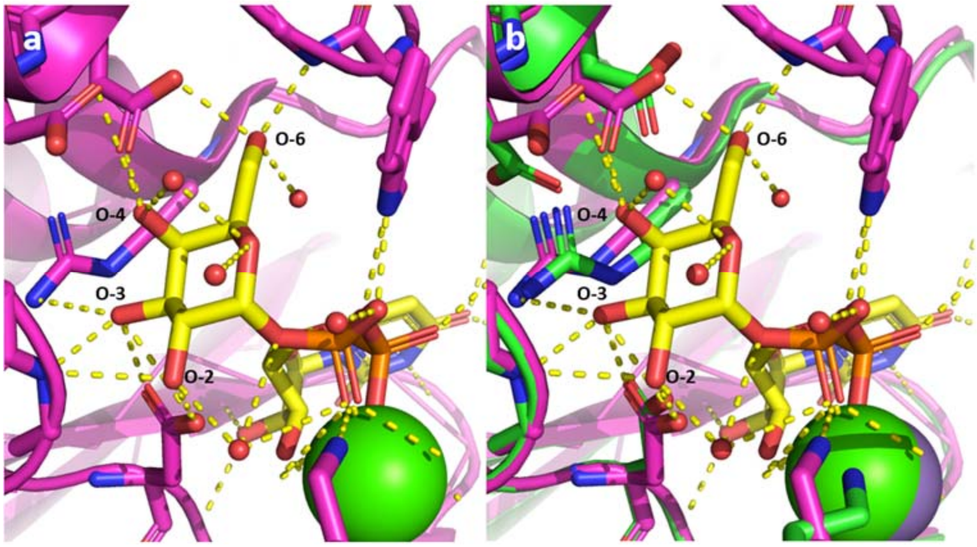

Supplementary Figure 3. Crystal structure of β(1,4)-galactosyltransferase bound to UDP-Gal. a) UDP-Galactose (yellow) bound to bovine β-1,4-galactosyltransferase (pink) with potential hydrogen-bonding interactions between the UDP-Gal and protein/bound water molecules shown as dotted lines. Image generated in PyMOL using Protein Data Bank file 2fyc.pdb. b) overlay of image a) with the structure of human b-1,4-galactosyltransferase (green) derived from Protein Data Bank file 2fyb.pdb shows that the key residues involved in interactions with the galactose residue are conserved between the bovine and human enzymes.

2.4 Supplementary Figure 4. Crystal structure of *Hp*-α1-3FucT bound to GDP-Fuc

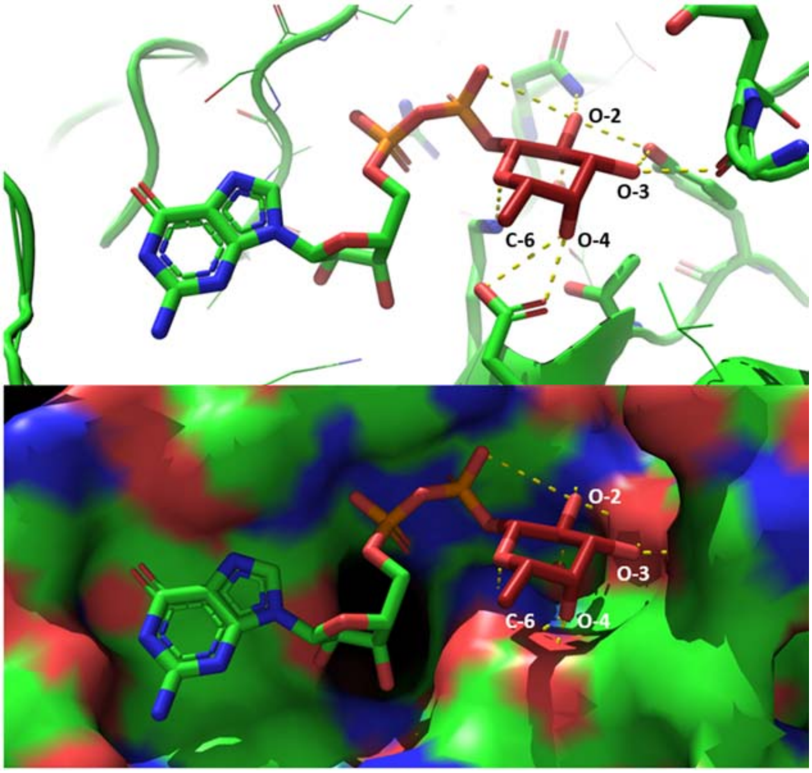

Supplementary Figure 4. GDP-Fucose binding to Helicobacter pylori α-1,3-fucosyltransferase. Potential hydrogen bonding interactions between heavy atoms are indicated by the yellow dashed lines. Figure was created in PyMOL using Protein Data Bank file 2nzy.pdb.

2.5 Supplementary Figure 5. Increased chemical shift of fucose H-5

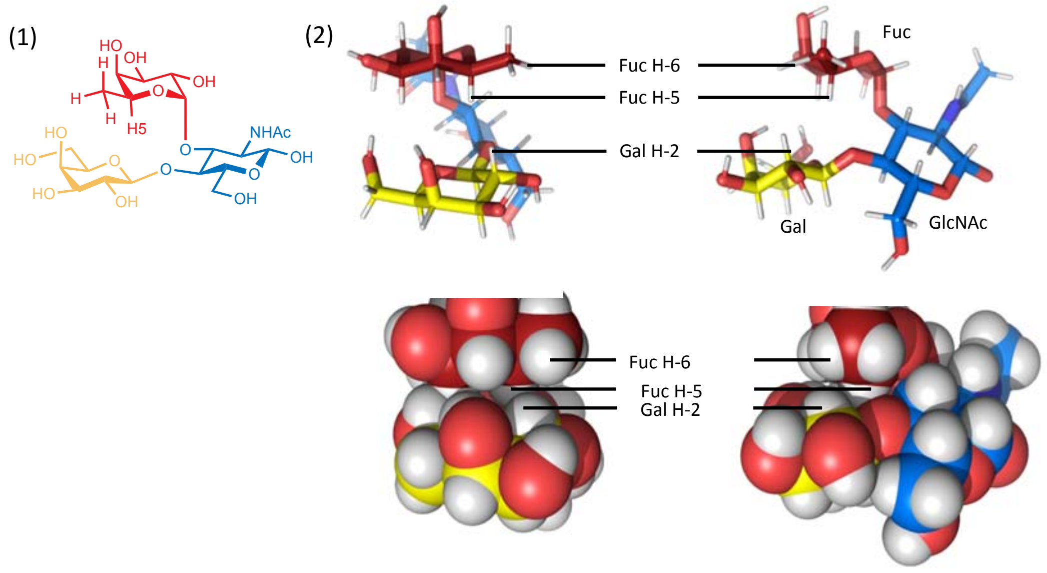

Sugar	δ H-5'' (ppm)	Sugar	δ H-5'' (ppm)	Sugar	δ H-5'' (ppm)
**LeX 1**	**4.83**	LeX 9	4.84	LeX 17	4.86
LeX 2	4.85	LeX 10	4.84	LeX 18	4.84
LeX 3	4.77	LeX 11	4.82	LeX 19	4.83
LeX 4	4.86	LeX 12	4.9	LeX 20	4.94
LeX 5	4.88	LeX 13	5.07	LeX 21	5.13
LeX 6	4.82	LeX 14	4.64	LeX 22	4.8
LeX 7	4.82	LeX 15	5.07	LeX 23	4.84
LeX 8	4.8	LeX 16	5.01	LeX 24	5.05

Supplementary Figure 5. The 'closed conformation of Lewis^x.

The increased chemical shift of fucose H-5 was observed for the Lewisx glycofluoroforms (4.77-4.88 ppm for Lewis^x structures containing Fuc **4a**, 3F-Fuc **4b**, 4F-Fuc **4c**; 5.01-5.53 ppm for Lewis^x structures containing 6F-Fuc **4d**, Figure 3), as is the case for nonfluorinated Lewis^x (referencing to water signal) This evidenced that all analogues adopted the well-defined 'closed conformation' when in aqueous medium, that is known to result from a non-conventional CH-O hydrogen bond between fucose H-5 and the ring oxygen of the galactosyl residue.[1]

2.6 Supplementary Figure 6. Further NMR-evidence of the glycofluoroform closed conformation for 6F-Fuc glycofluoroforms

An interesting result when comparing the NMR of the Lewis^x analogues was the effect of fluorination at the 6-position of fucose on H-2 of the galactose. These two are not close through bonding but in structures of Lewis^x are held spatially close to each other. The axial Fuc OH-4 would force the fluorine in the 6-position into the tg or gt conformation (Supplementary Figure S6a). This is supported by coupling constant analysis: for all glycofluoroforms fluorinated at the fucose 6-position, the vicinal $^3J_{H5\text{-}F6}$ value is around 17 Hz, which clearly indicates a *gauche*-dihedral angle, which gives two possible conformers as shown. This renders the two H6-protons non-equivalent, with one having an *anti*-periplanar dihedral angle with H5, leading to a large $^3J_{H5\text{-}H6}$ value (~8 Hz), and the other hydrogen having a *gauche*-dihedral, leading to a small $^3J_{H5\text{-}H6}$ value (~3–4 Hz).

Hence, this results in the fluorine atom pointing it towards the H-2 of the galactose, which has a deshielding effect on Gal H-2 (4b). Interestingly, for one glycofluoroform, the galactose C2 signal in the ^{13}C NMR spectrum displayed a doublet, despite no fluorine was present in the same ring (Supplementary Figure 6b). We propose this arises from the vicinity of the fucose F6 fluorine atom.

(a) 6-fluorinated fucose fluoroforms: Conformation of fluoromethyl group

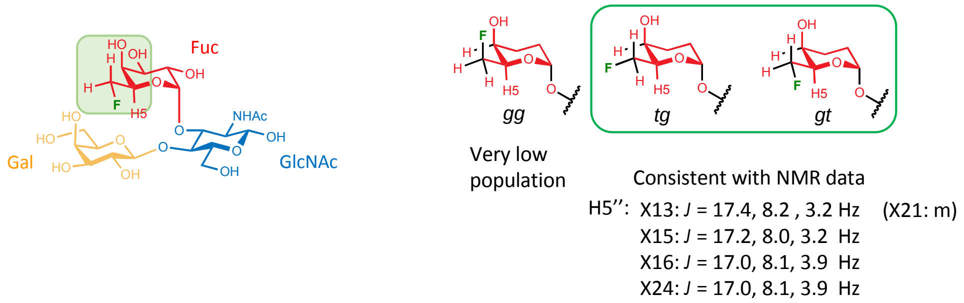

(b) 6-fluorinated fucose fluoroforms: further NMR support of closed conformation

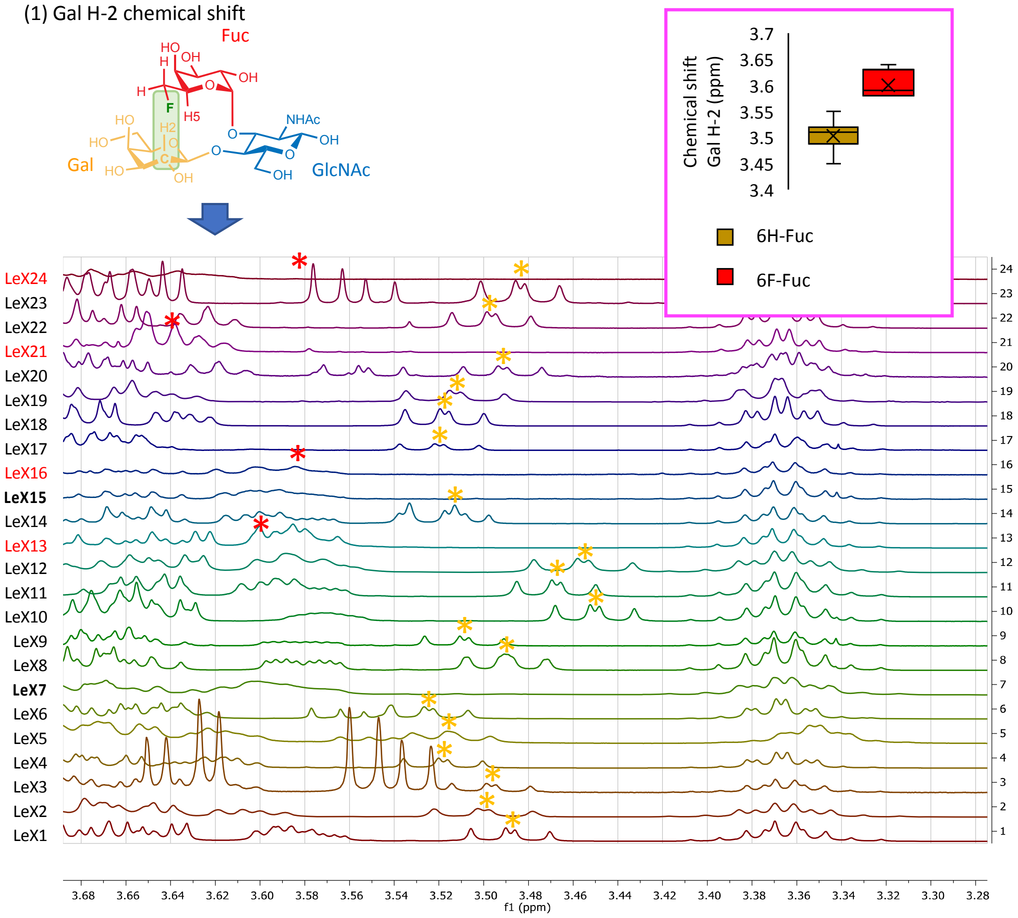

Collated ^{1}H NMR of all 24 Lewis^{x} analogues. Asterisks highlight position of Gal H-2: red donates sugars containing 6FFuc and gold donates all other sugars. The two sugars containing 3FGal are shown in bold and are not included in the analysis due to the proximal effect of fluorine next to Gal H-2. Inset: A box and whisker plot comparing 6FFuc containing Lewis^{x} analogues (red) to the rest.

Supplementary Figure 6. Explanation of the observed increased chemical shift of the galactose H-2 when the fucose residue is fluorinated at C6.

2.7 Supplementary Figure 7. Histogram chart representation of the results of binding of hDC-SIGNR, Rhesus Langerin and anti-Lewis^{x} (BG-7) to the NGL probes in the microarray set tested.

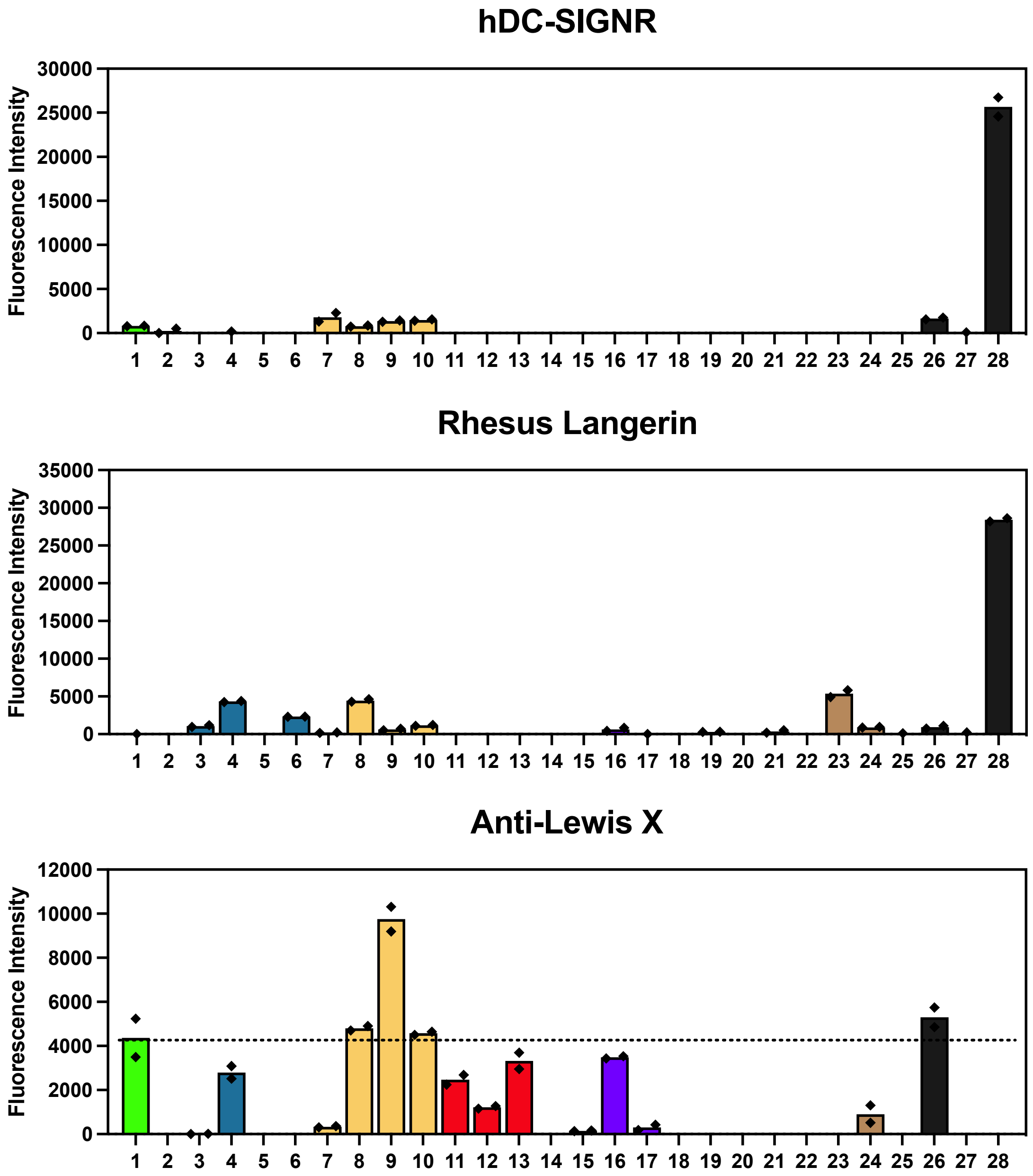

Supplementary Figure 7. Histogram chart representation of the results of binding of hDC-SIGNR, Rhesus Langerin and anti-Lewis^{x} (BG-7) to the NGL probes in the microarray set tested. The microarray consists of 28 NGL probes: 24 Lewis^{x} trisaccharide derived NGLs 4 control NGLs; the probe list and the glycan sequences are in Table S2. Colours used for the intensity bars: green, LeX1, the native Lewis^{x} trisaccharide NGL; purple, NGLs of the unnatural Lewis^{x} trisaccharides; black, the NGL standards.

2.8 Supplementary Figure 8. Histogram charts showing the influence of fluorination of the Lewis^{x} trisaccharide on the binding signals with hDC-SIGN, Classical CTB, LTBh, and anti-Lewis^{x} antibodies anti-L5, anti-SSEA-1 and anti-BG7.

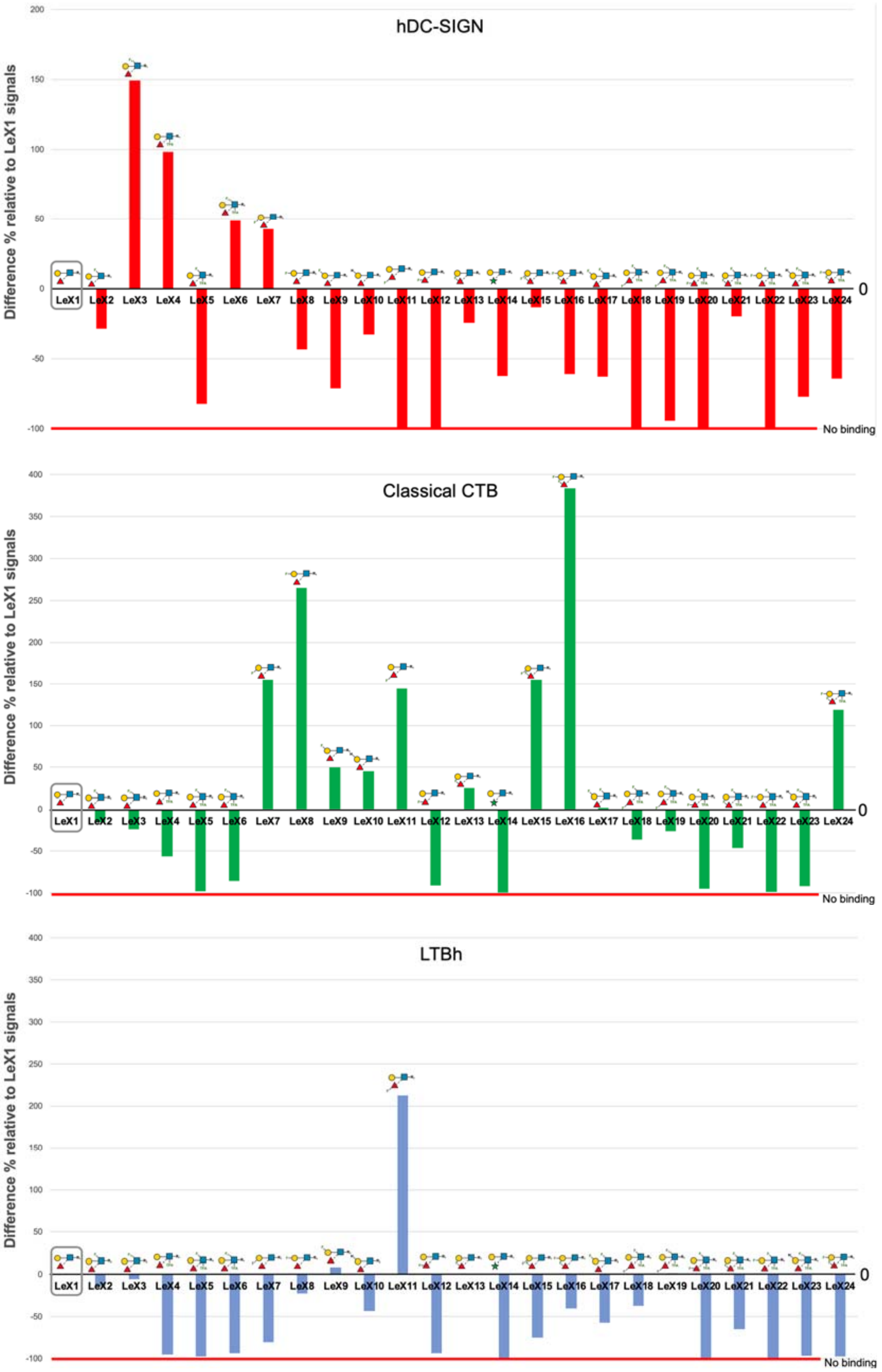

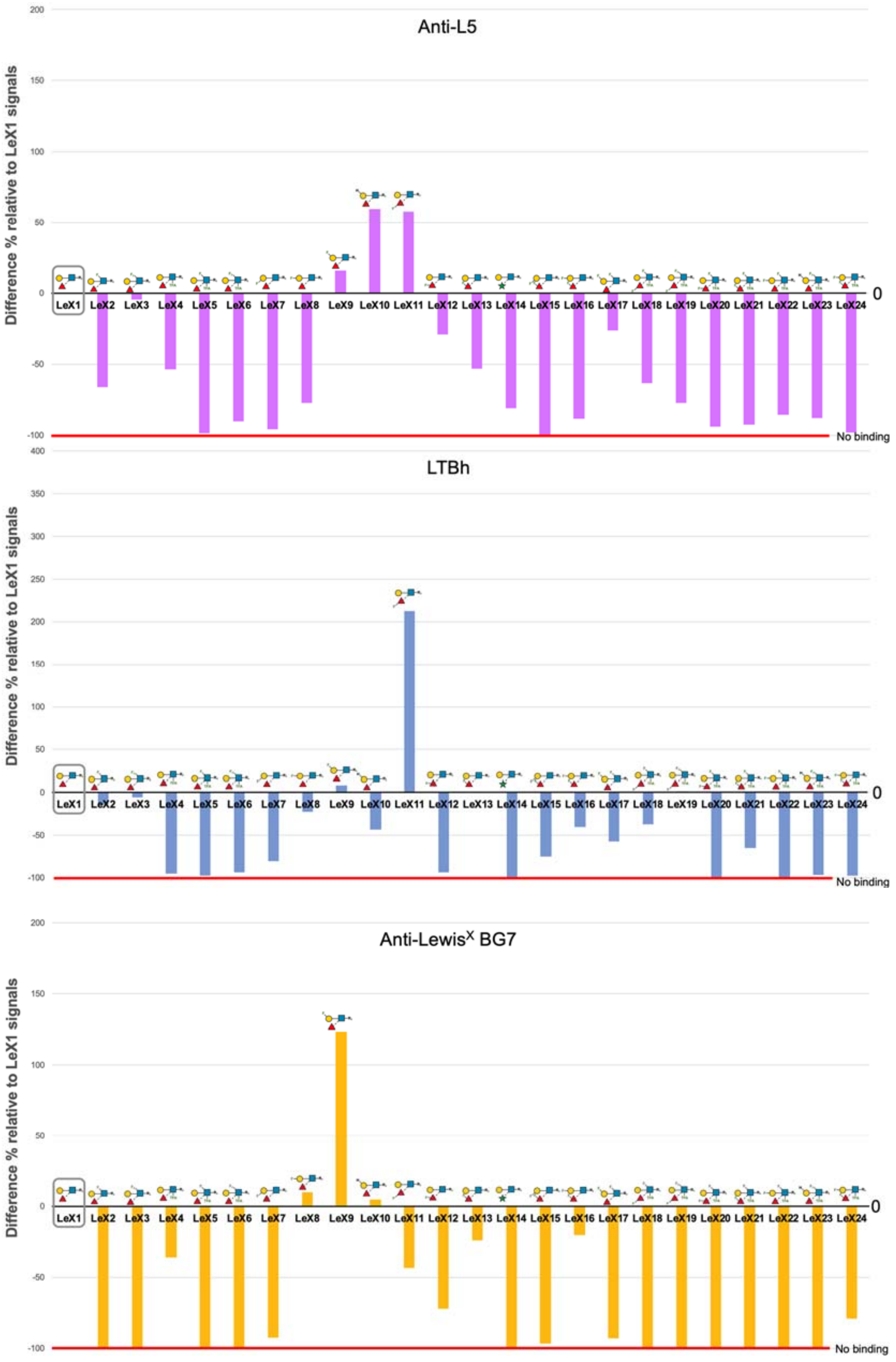

Supplementary Figure 8. Histogram charts showing the influence of fluorination of the Lewis^x trisaccharide on the binding signals with hDC-SIGN, Classical CTB, LTBh, and anti- Lewis^x antibodies anti-L5, anti-SSEA-1 and anti-BG7. With each protein the fluorescence intensity values are normalized against those of the unmodified Lewis^x probe LeX1. The enhancement or diminution of binding signals are shown as percentage values (LeX1 shown as 0%), positive or negative, respectively. -100% indicates no binding detected. The raw fluorescence intensity and the normalized values are in SOURCE DATA file.

.

2.9 Supplementary Figure 9. ‘Fingerprints’ of the binding of the Classical CTB, LTBh, hDC-SIGN, and anti-Lewis^X antibodies anti-L5, anti-SSEA-1 and BG-7 showing effects of fluorination of the Lewis^X trisaccharide.

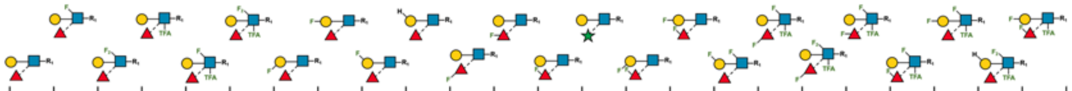

	LeX1	LeX2	LeX3	LeX4	LeX5	LeX6	LeX7	LeX8	LeX9	LeX10	LeX11	LeX12	LeX13	LeX14	LeX15	LeX16	LeX17	LeX18	LeX19	LeX20	LeX21	LeX22	LeX23	LeX24
hDC-SIGN	0	-29	+149	+98	-82	+49	+42	-43	-71	-33	-100	-100	-25	-62	-13	-61	-63	-100	-94	-100	-20	-100	-77	-64
Anti-L5	0	-66	-5	-53	-99	-90	-96	-77	+16	+60	+58	-29	-53	-81	-99	-88	-26	-63	-77	-94	-92	-85	-88	-98
Anti-SSEA-1	0	-100	-100	-35	-100	-100	-100	-56	+157	+10	-76	-100	-82	-100	-100	-90	-100	-100	-100	-100	-100	-100	-100	-100
Anti-Lewis^X (BG-7)	0	-100	-100	-36	-100	-100	-92	+10	+123	+5	-43	-72	-24	-100	-97	-20	-93	-100	-100	-100	-100	-100	-100	-79
Classical CTB	0	-15	-24	-56	-98	-85	+155	+265	+50	+46	+144	-91	+25	-99	+156	+384	+2	-36	-26	-95	-46	-99	-92	+119
LTBh	0	-14	-6	-95	-97	-93	-80	-23	+8	-43	+213	-93	+1	-99	-75	-40	-57	-37	+1	-99	-65	-100	-97	-97

Supplementary Figure 9. ‘Fingerprints’ of the binding of the Classical CTB, LTBh, hDC-SIGN, and anti-LewisX antibodies anti-L5, anti-SSEA-1 and BG-7 showing effects of fluorination of the Lewis^X trisaccharide.

The fingerprints have been generated by normalizing the fluorescence intensity values against those of the native Lewis^X trisaccharide LeX1. The difference, enhancement and diminution of binding signals is shown as a percentage value: Negative values mean that the binding intensities are lower than that of LeX1 which is shown as 0%, e.g. -100% is for no binding detected. The raw fluorescence intensity and the normalized values are in the SOURCE DATA File. Colour code for binding intensity is as follows: yellow, similar to the native (plus or minus 25%); green, increased by greater than 25%; red, decreased to less than 25%.

2.10 Supplementary Figure 10. Individual spider charts showing the influence of fluorination of the Lewis^X trisaccharide on the binding signal intensities of hDC-SIGN, the Classical CTB and LTBh, and the anti-Lewis^X antibodies anti-L5, anti-SSEA-1 and BG-7.

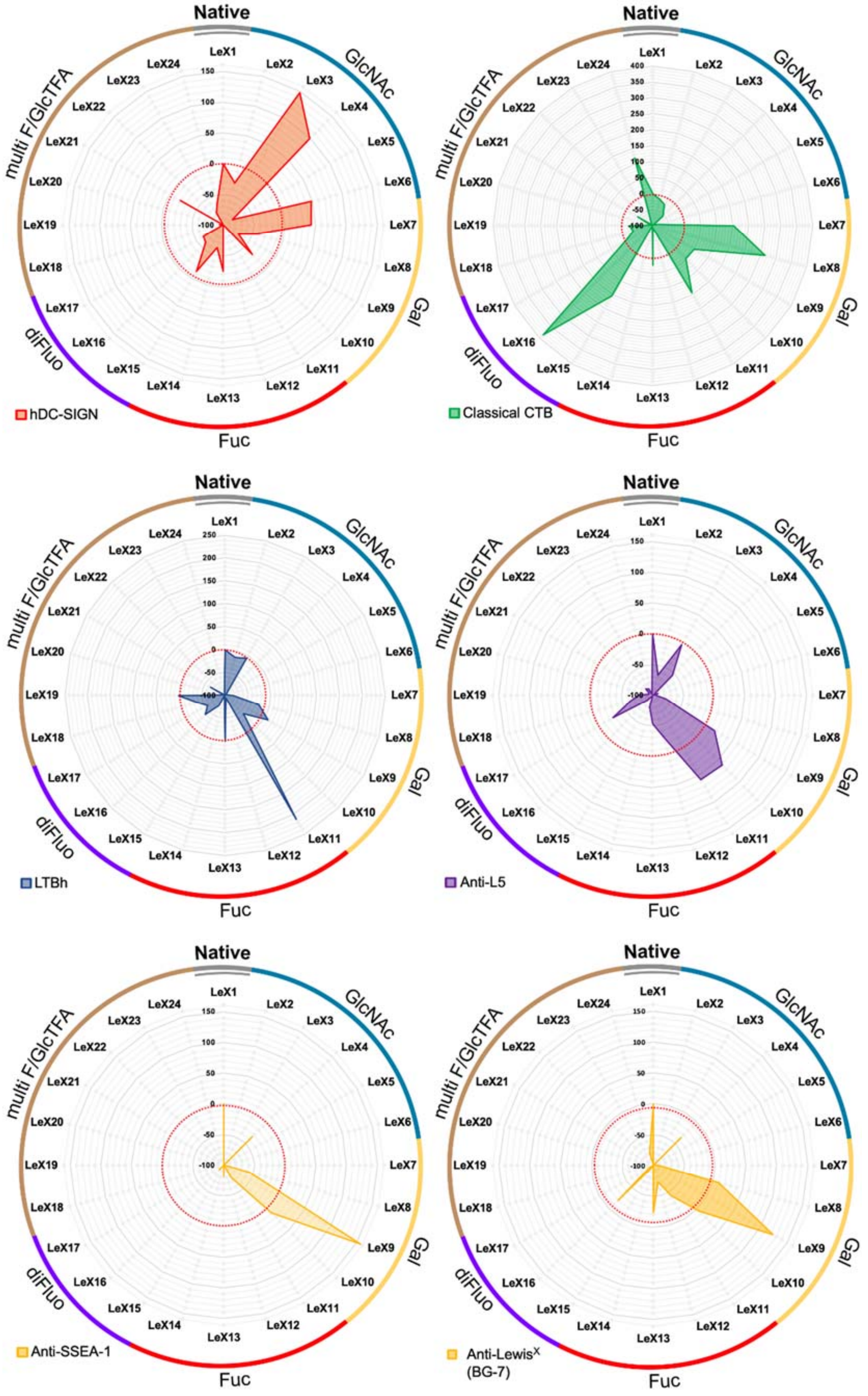

Supplementary Figure 10. Individual spider charts showing the influence of fluorination of the Lewis^x trisaccharide on the binding signal intensities of hDC-SIGN, the Classical CTB and LTBh, and the anti- Lewis^x antibodies anti-L5, anti-SSEA-1 and BG-7. The raw fluorescence intensity and the normalized values are in the SOURCE DATA file.

2.11 Supplementary Figure 11. Isothermal Titration Calorimetry of (a) Classical and (b) El Tor CTB with Lewis^x

Isothermal titration calorimetry was conducted with a MicroCal iTC200 calorimeter (202.8 μL cell volume) operating at 25 °C. Lewis^x (47 mM) in phosphate-buffered saline (PBS) at pH 7.4 was titrated into a solution of either Classical CTB (275 μM protomer / 55 μM pentamer) or El Tor CTB (250 μM protomer / 50 μM pentamer) in the same PBS buffer. A single 2 μL injection was followed by 19 injections of 4 μL at 120 second intervals while stirring at 750 rpm. Data was processed using the Malvern PEAK ITC software using the standard one-site binding model and fixing the binding stoichiometry to 1 ligand per CTB protomer. Titrations were conducted in triplicate and a control experiment in which Lewis^x was titrated into matched PBS buffer was subtracted from each dataset to account for heat of dilution of the ligand.

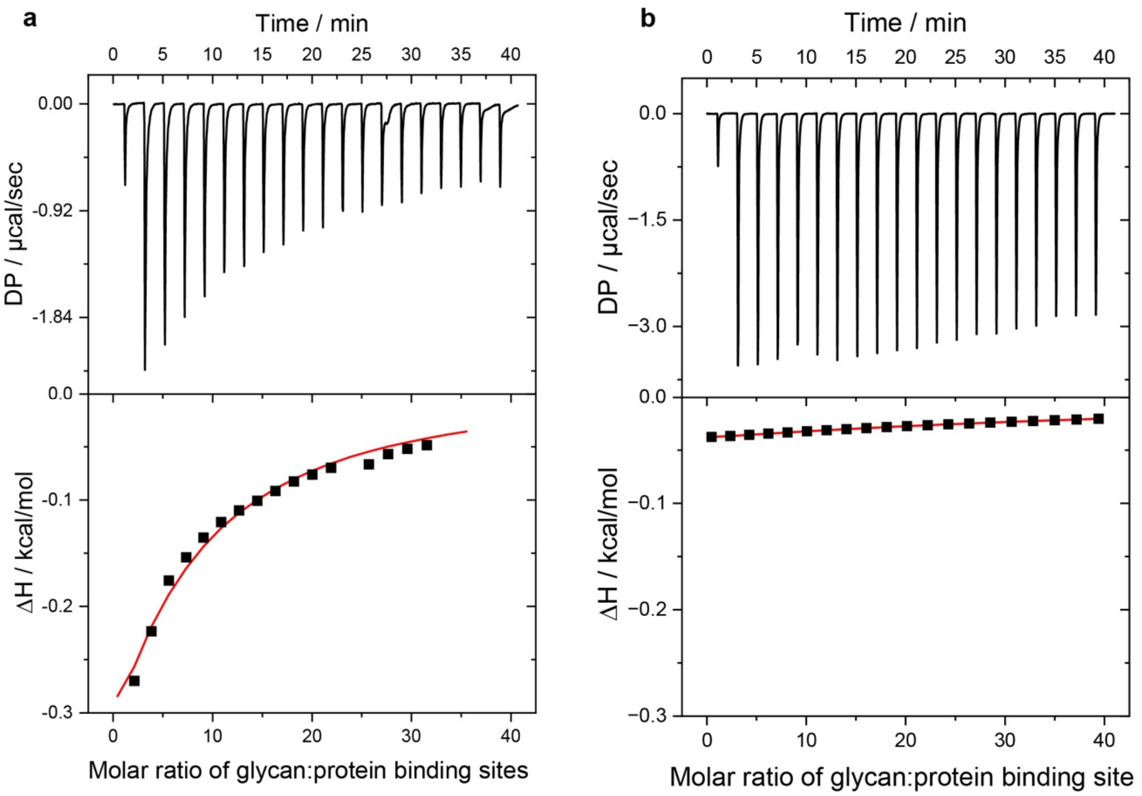

Supplementary Figure 11. Isothermal Titration Calorimetry of (a) Classical and (b) El Tor CTB with Lewis^x showing examples of the thermograms (top) and integrated heat data after subtracting for heat of dilution of Lewis^x (bottom). DP is the difference in power supplied to the sample cell and reference cell during the titration. Titrations conducted in triplicate gave an average K_d of 6.1 ±0.3 mM and ΔH of -6.84 ±0.47 kcal/mol for Classical CTB, whereas no binding could be detected for El Tor CTB under these conditions.

2.12 Supplementary Figure 12. HPTLC analyses of the 24 NGLs prepared from the azido-terminated Lewis^x structures

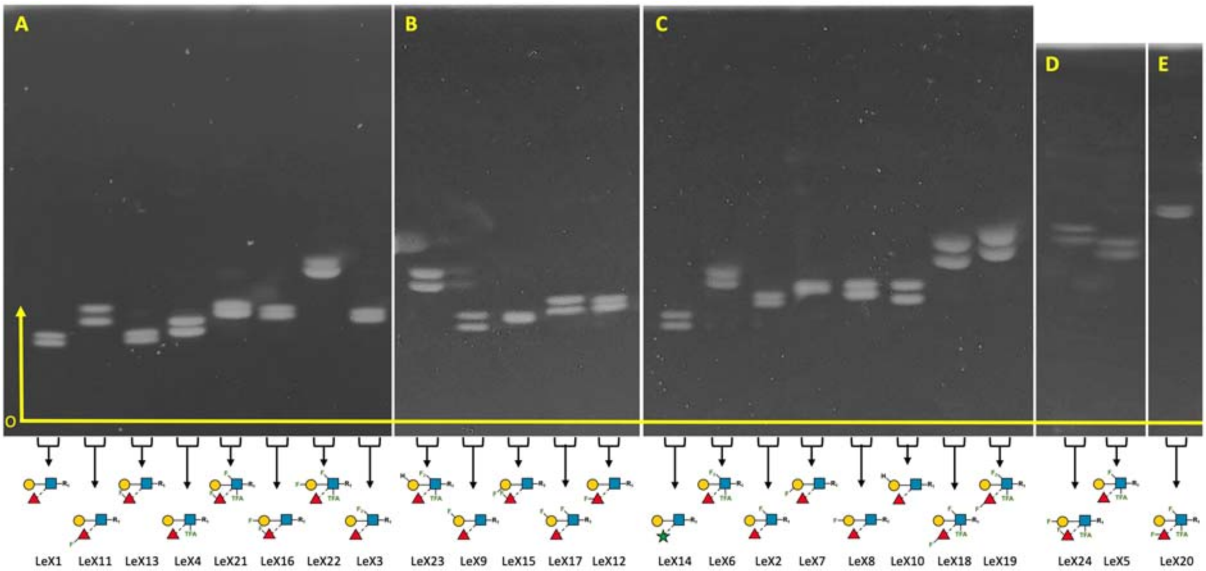

Supplementary Figure 12. HPTLC analyses of the 24 NGLs prepared from the azido-terminated Lewis^X structures. Amounts applied 250pmol in panels A, B and C, and 100pmol in panels D and E. Double bands are visualised for the NGLs due to the presence of the two regioisomeric products from the DBCO-based SPAAC reaction (Supplementary Figure 2). Solvent System $CHCl_3$:MeOH:H_2O, 130:50:9 (by volume). O, the origin of the HPTLC plate. R_1= OCH2-CH2-CH2-N_3-DBCO-DH. The un-cropped HPTLC images are in the SOURCE DATA File.

2.13 Supplementary Figure 13. UV-Vis analysis of unfunctionalised ($AuNP_{55}$ – black dotted line) and functionalised AuNPs used in this study

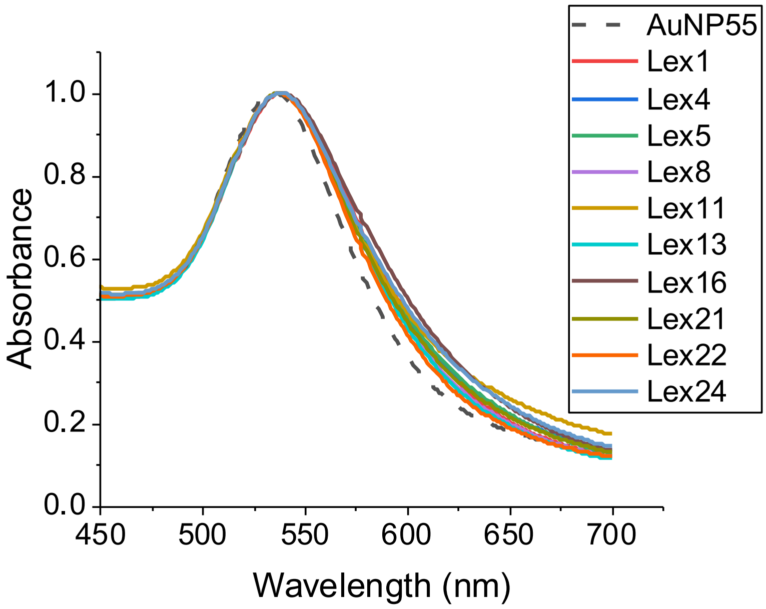

Supplementary Figure 13. UV-Vis analysis of unfunctionalised ($AuNP_{55}$ – black dotted line) and functionalised AuNPs used in this study. In an aggregated state one would observe an increase in absorbance at 700 nm.

2.14 Supplementary Figure 14 Dynamic Light Scattering analysis of unfunctionalised ($AuNP_{55}$ – black dotted line) and functionalised AuNPs used in this study

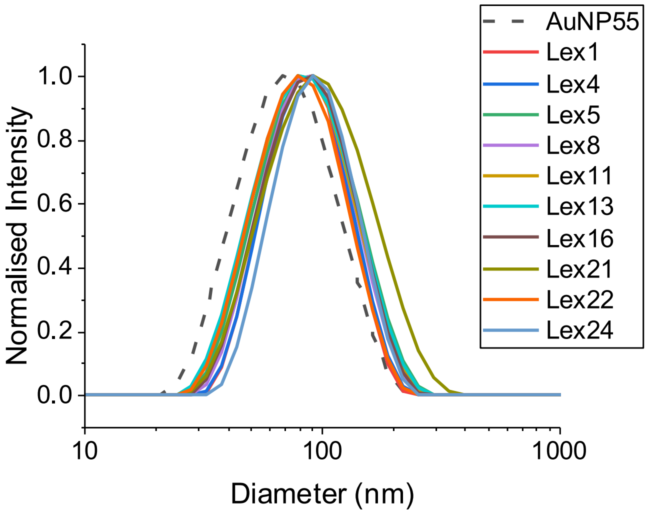

Supplementary Figure 14. Dynamic Light Scattering analysis of unfunctionalised ($AuNP_{55}$ – black dotted line) and functionalised AuNPs used in this study.

This Figure shows that compared to the unfunctionalized AuNP (black dotted line), the functionalised AuNPs have a ~10 nm increase in size. This confirms their non-aggregated state. See Table S6 for actual measurement data.

2.15 Supplementary Figure 15: TEM-analysis of unfunctionalised and functionalised AuNPs used in this study

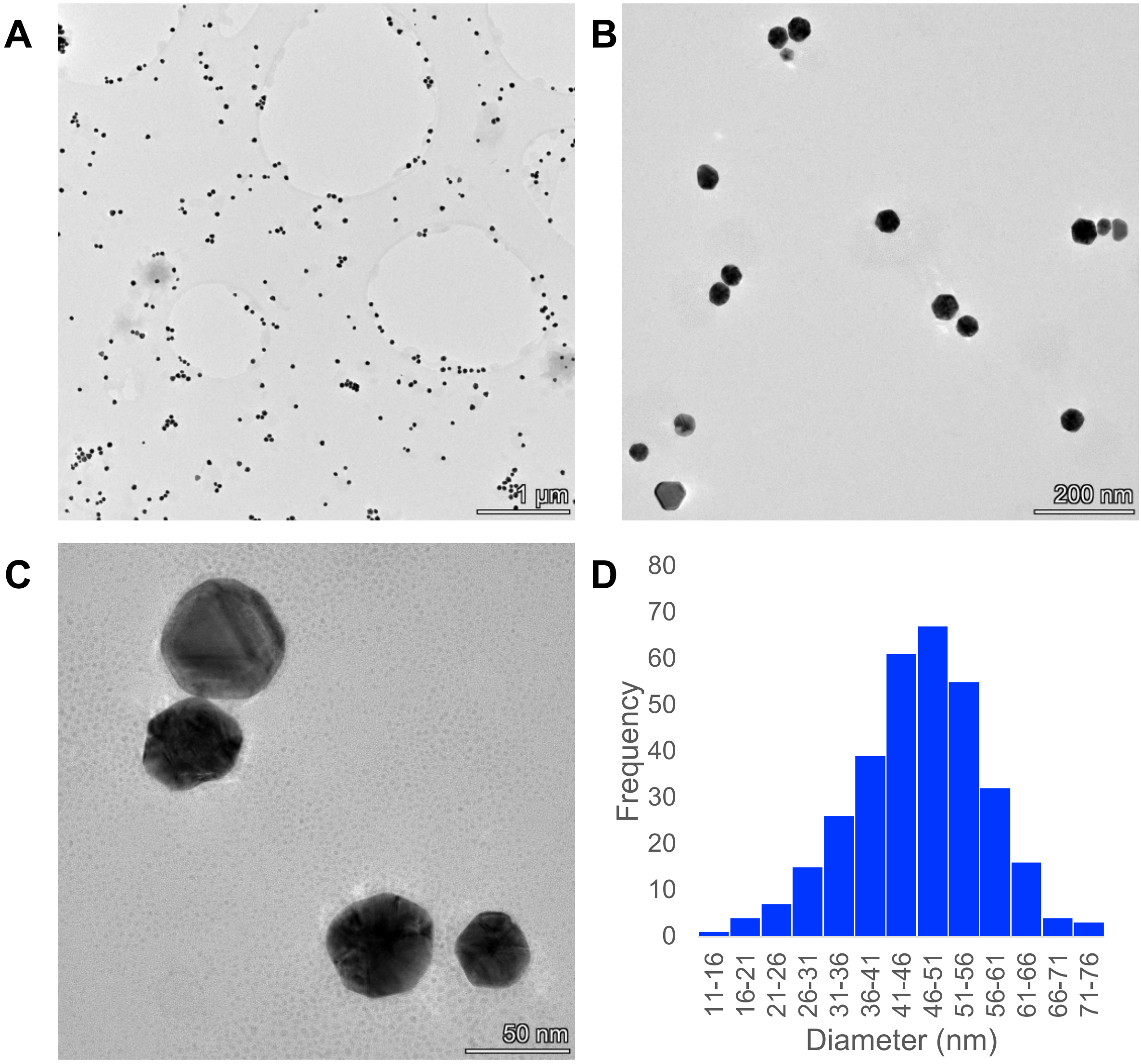

Supplementary Figure 15a. TEM-analysis of unfunctionalised AuNPs used in this study. A)-C) Representative TEM images of unfunctionalised AuNPs. D) Histogram of sizes from analysis from 330 particles.

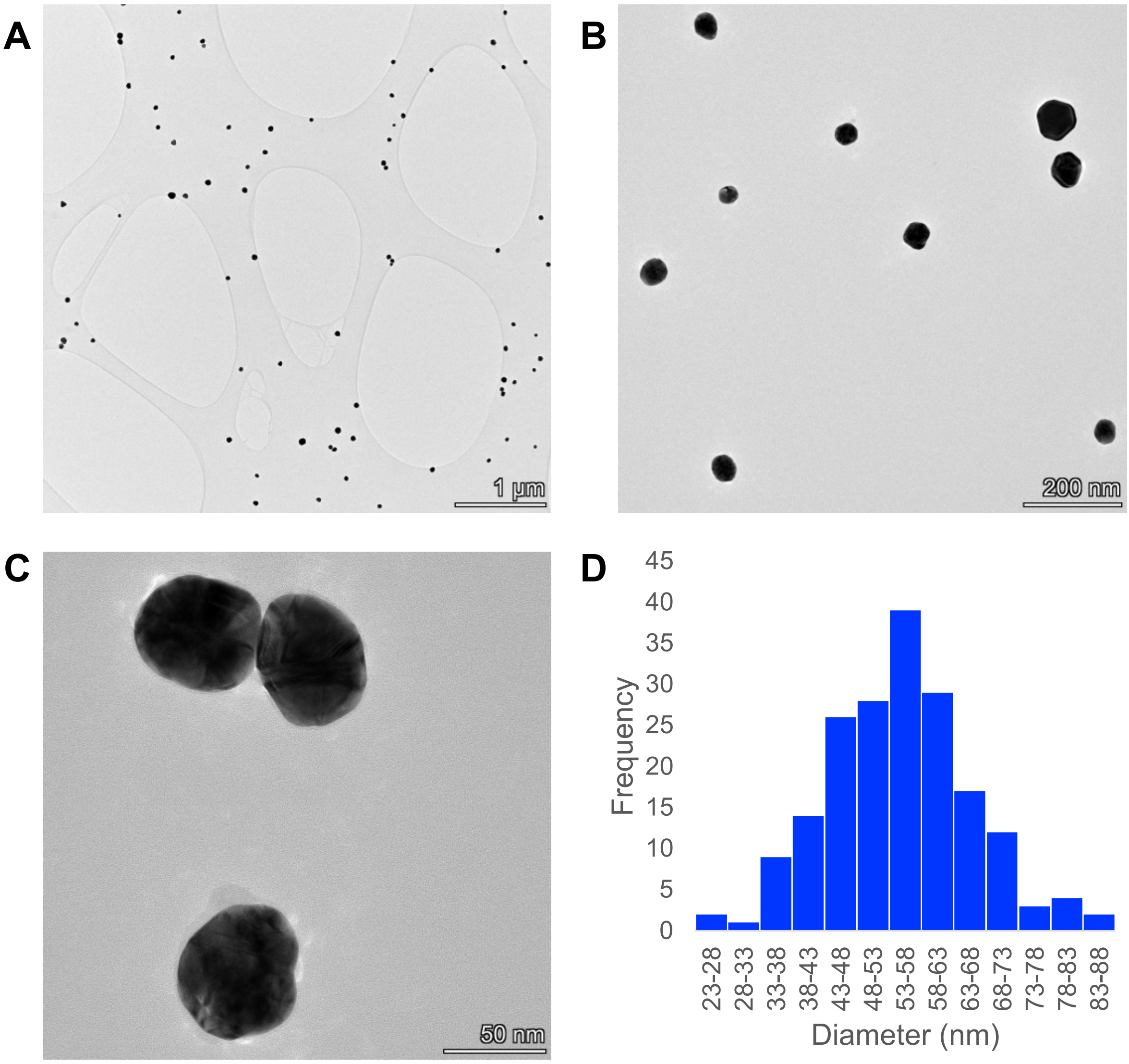

Supplementary Figure 15b. TEM-analysis of functionalised AuNPs used in this study. A)-C) Representative TEM images of (functionalised) LeX16-PHEA@AuNPs. D) Histogram of sizes from analysis from 186 particles.

2.16 Supplementary Figure 16. UV-Vis analysis of CTB-induced Aggregation

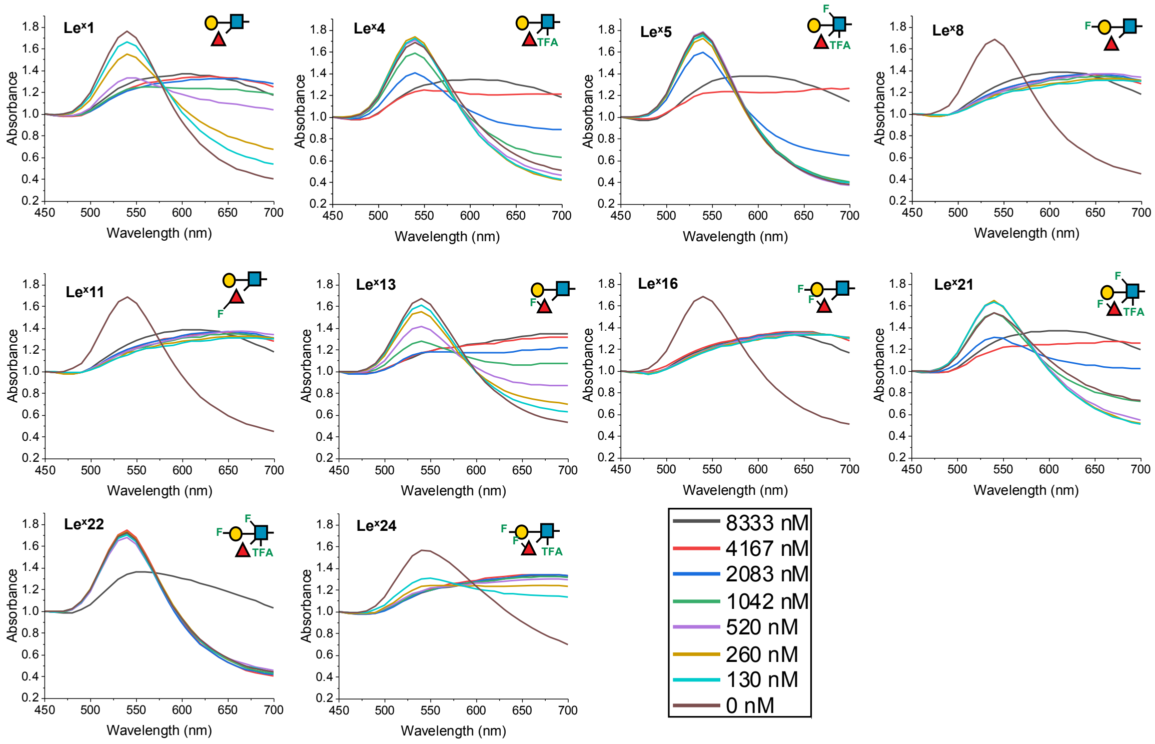

Supplementary Figure 16: Initial UV-Vis analysis of Le^x-functionalised AuNPs in response to a serial dilution of CTB (8333 nM – (0.1 mg.mL^{-1} start).

2.17 Supplementary Figure 17a-c. Negative and positive controls for the aggregation assay.

Glyconanoparticle aggregation on addition of enterotoxigenic *Escherichia coli* heat-labile toxin B subunit (LTB) (another bacterial toxin) was carried out in the same way as the CTB aggregation assay (Figure 5 main article). Supplementary Figure 17a shows the complete dose-dependent binding responses of the Lewis^x glycoforms versus LTB.

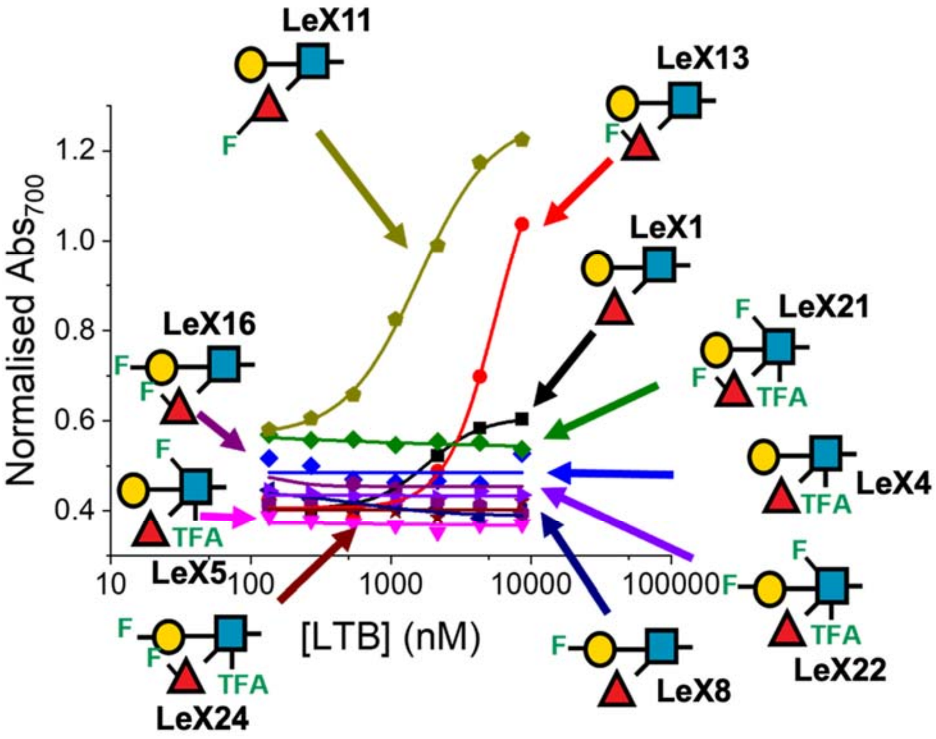

Supplementary Figure 17a. Dose-dependent response of library of Lewis^x glyconanoparticles to LTB. Data is presented as normalised Abs_{700} from UV-Visible spectroscopy.

LeX1 the native Lewis X glycan gave a very weak binding response. LeX11 and LeX13, 3F-Fuc and 6F-Fuc respectively, gave increased binding responses, which again correlates well with the microarray data shown in Figure 4b from the main article. However, the microarray data suggest increase binding should be observed with LeX8 also, however this is not observed here. All other glycoforms gave no response in the concentration range tested which agrees with the microarray data.

Two plant lectins were also tested as a positive and negative control for binding. Again, glyconanoparticle aggregation on addition of lectin was carried out in the same way as the CTB aggregation assay (Figure 5 main article). *Aleuria Aurantia* Lectin (AAL) prefers fucose a1,3-linked on *N*-acetyllactosamine related structures therefore we expect to see good binding against the structures presented here. Supplementary Figure 17b shows the complete dose-dependent binding responses of the Lewis^x glycoforms versus AAL.

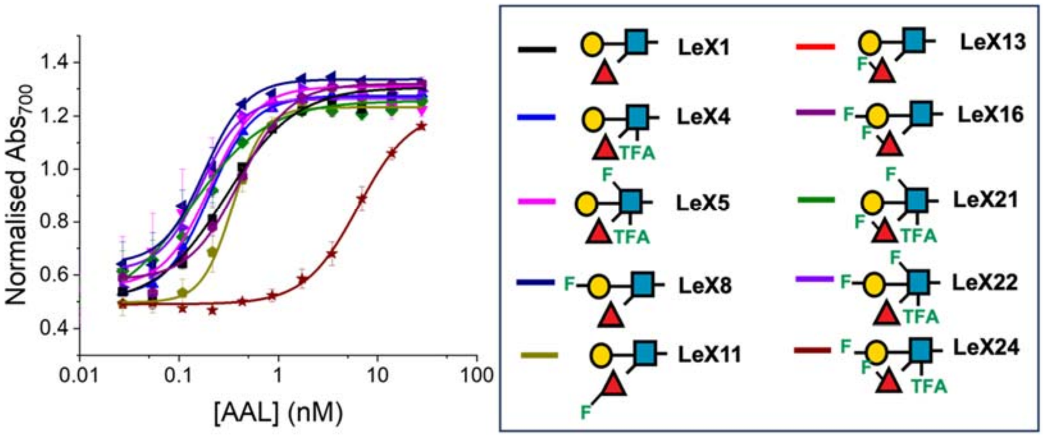

Supplementary Figure 17b. Dose-dependent response of library of Lewis^{x} glyconanoparticles to AAL. Data is presented as normalised Abs_{700} from UV-Visible spectroscopy.

LeX1 the native Lewis X glycan, gave a strong binding response as expected. All other glycoforms gave similar responses, except LeX24 which significantly reduced the binding response over 10-fold. The reason for this is unknown as the 4F-Gal, 6F-Fuc and GlcTFA is tolerated in other structures (e.g Lex4, LeX8, LeX13, LeX16, LeX 21 and LeX22).

Soybean agglutinin has a preference to *N*-Acetylgalactosamine therefore no/limited binding is expected against these structures. Supplementary Figure 17c shows the complete dose-dependent binding responses of the Lewis^{x} glycoforms versus SBA.

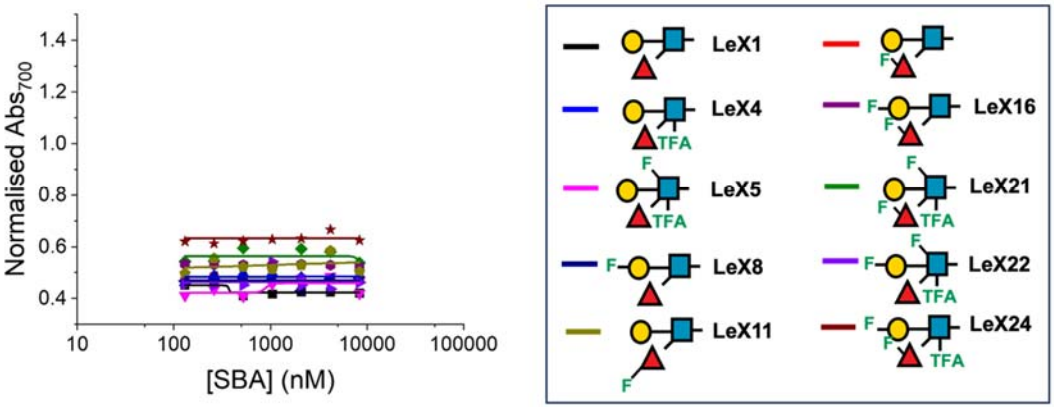

Supplementary Figure 17c. Dose-dependent response of library of Lewis^{x} glyconanoparticles to SBA. Data is presented as normalised Abs_{700} from UV-Visible spectroscopy.

As expected, no response to SBA was observed for any of the derivatives in the concentration range tested.

2.18 Supplementary figures 18-197. HRMS ITag screening assay mass spectra of both LacNAc and Lewis^{X} synthesis

The product peak is indicated by a red asterix.

2.18.1 Supplementary Figure 18. HRMS ITag screening assay mass spectrum of synthesis of Gal β1-4 GlcNAc-ITag

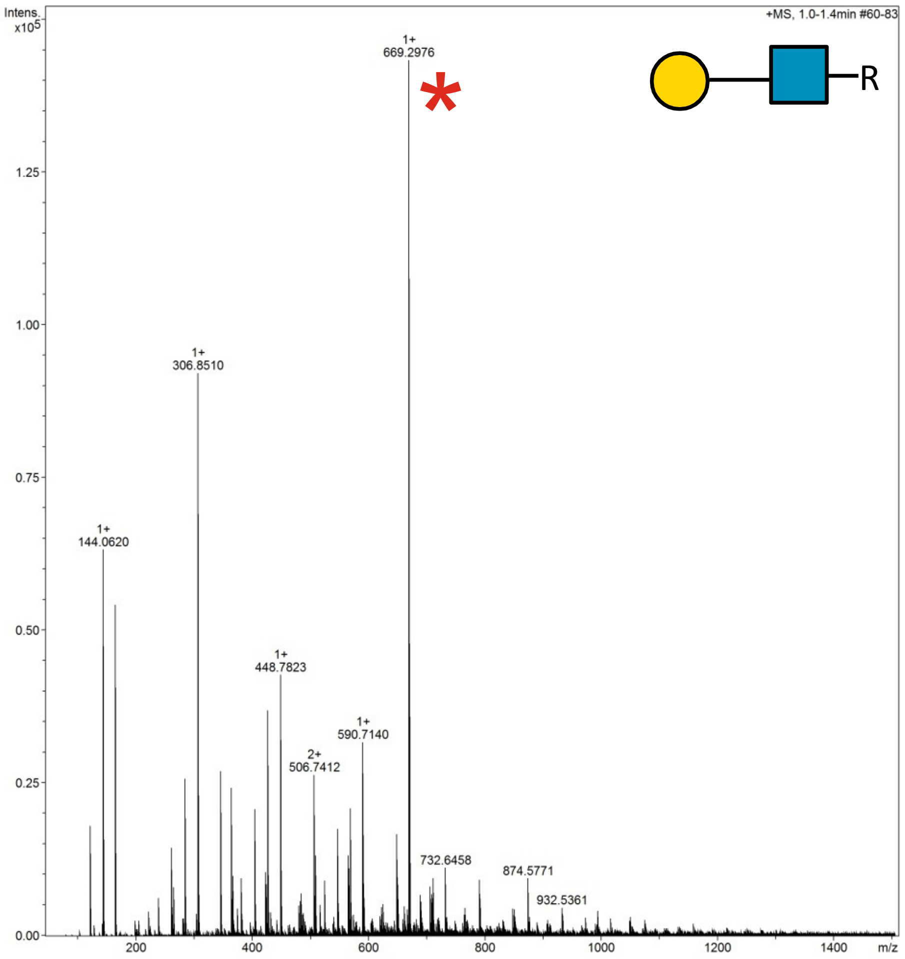

2.18.2 Supplementary Figure 19. HRMS ITag screening assay mass spectrum of synthesis of Gal β1-4 GlcNTFA-ITag

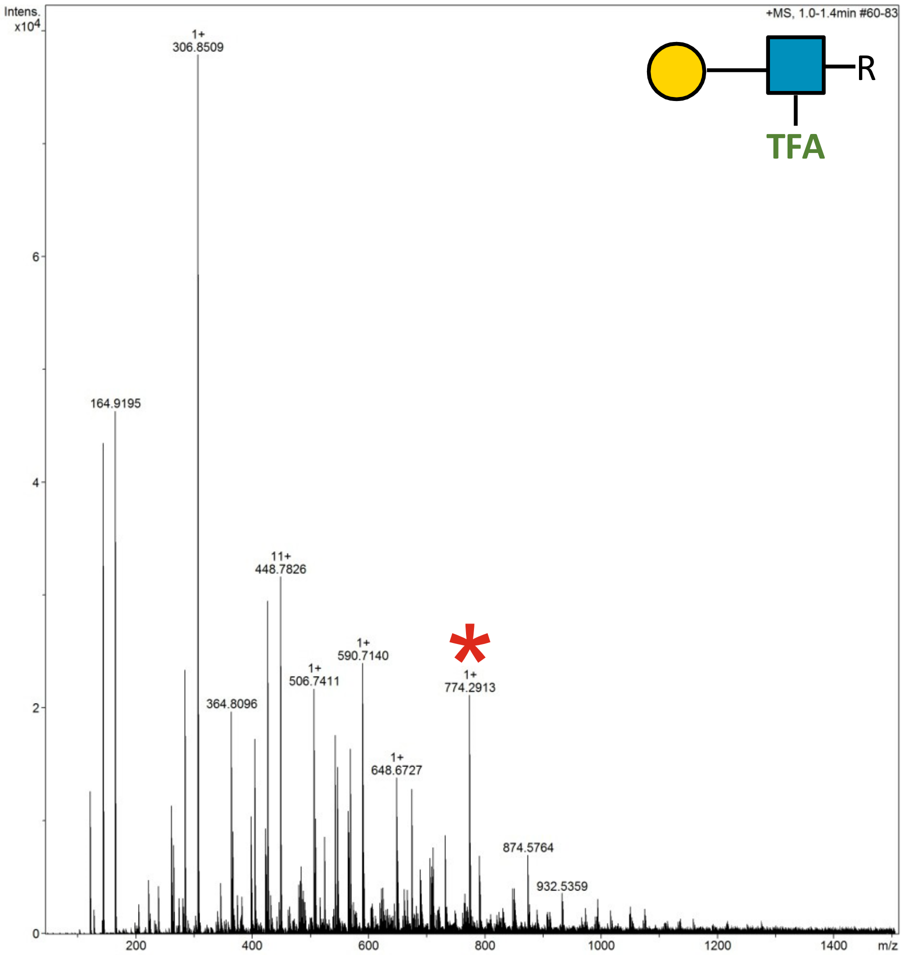

2.18.3 Supplementary Figure 20. HRMS ITag screening assay mass spectrum of synthesis of Gal β1-4 6F-GlcNAc-ITag

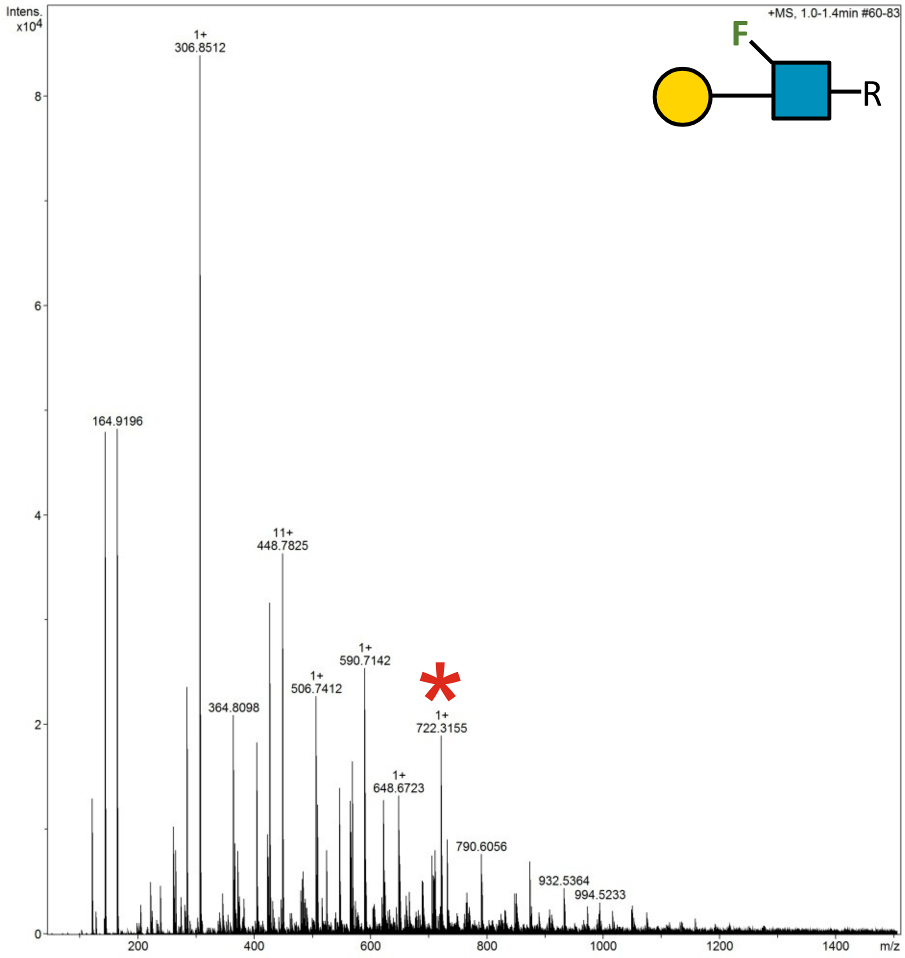

2.18.4 Supplementary Figure 21. HRMS ITag screening assay mass spectrum of synthesis of Gal β1-4 6F-GlcNTFA-ITag

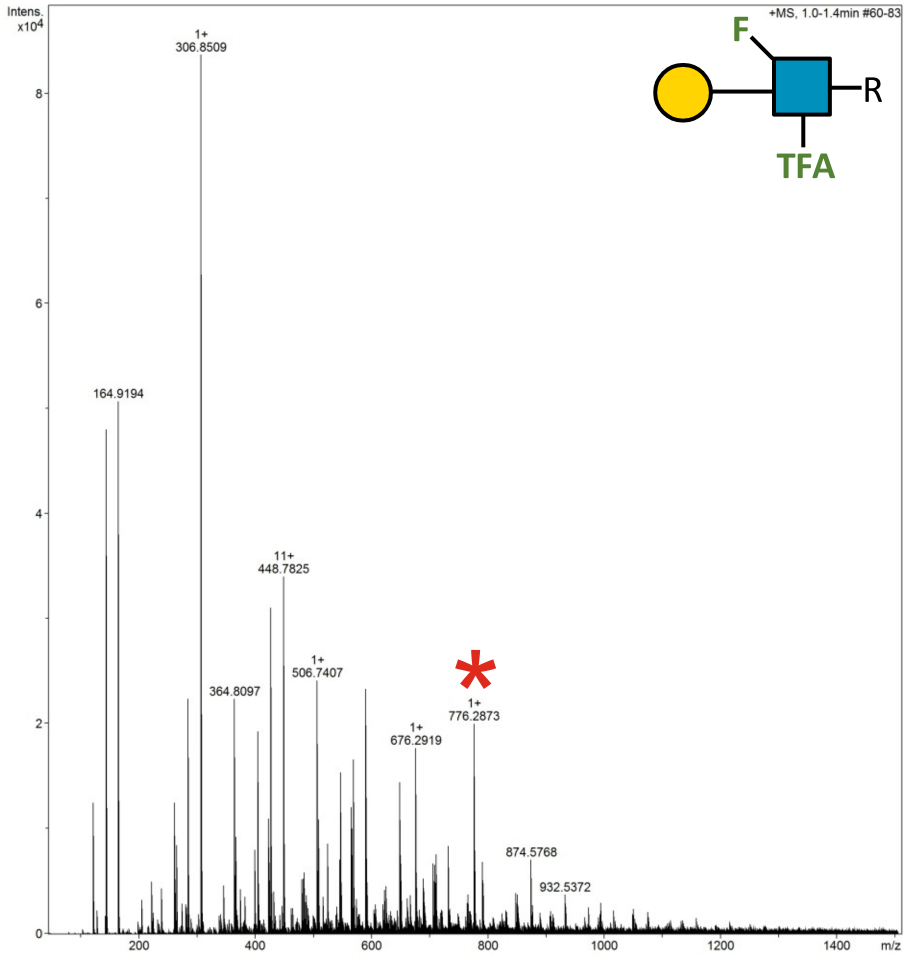

2.18.5 Supplementary Figure 22. HRMS ITag screening assay mass spectrum of synthesis of Gal β1-4 6,6-diFGlcNAc-ITag

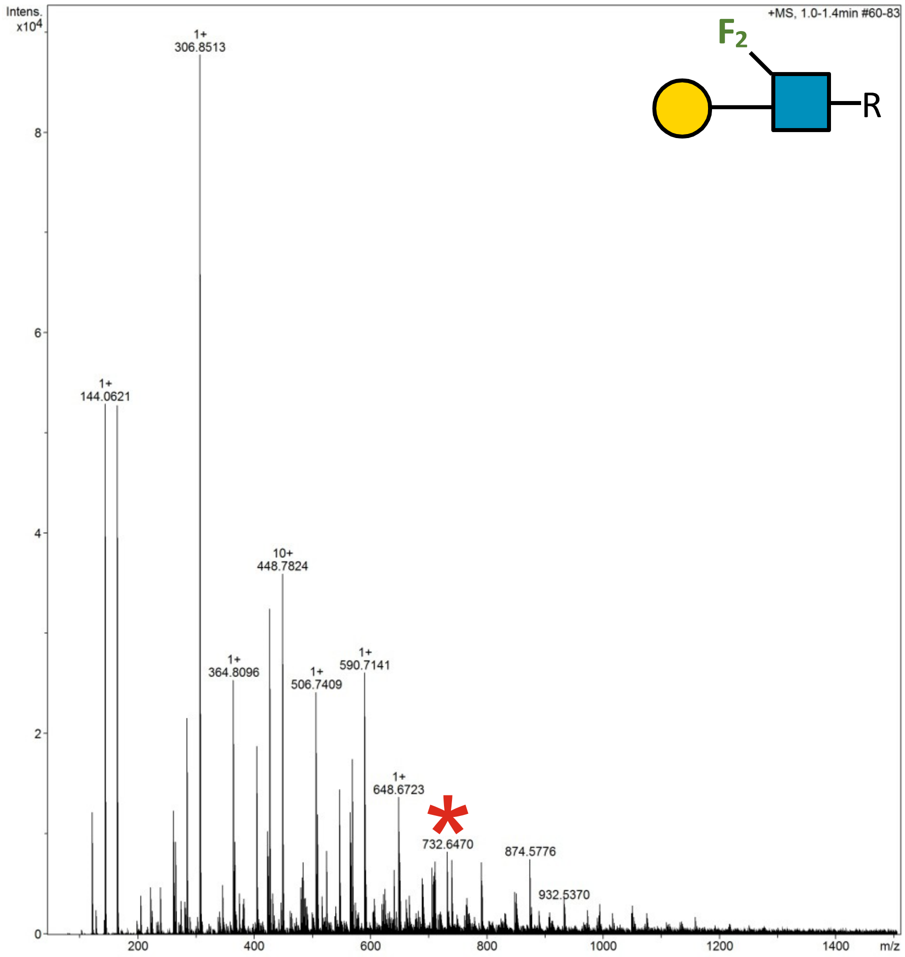

2.18.6 Supplementary Figure 23. HRMS ITag screening assay mass spectrum of synthesis of Gal β1-4 6,6-diFGlcNTFA-ITag

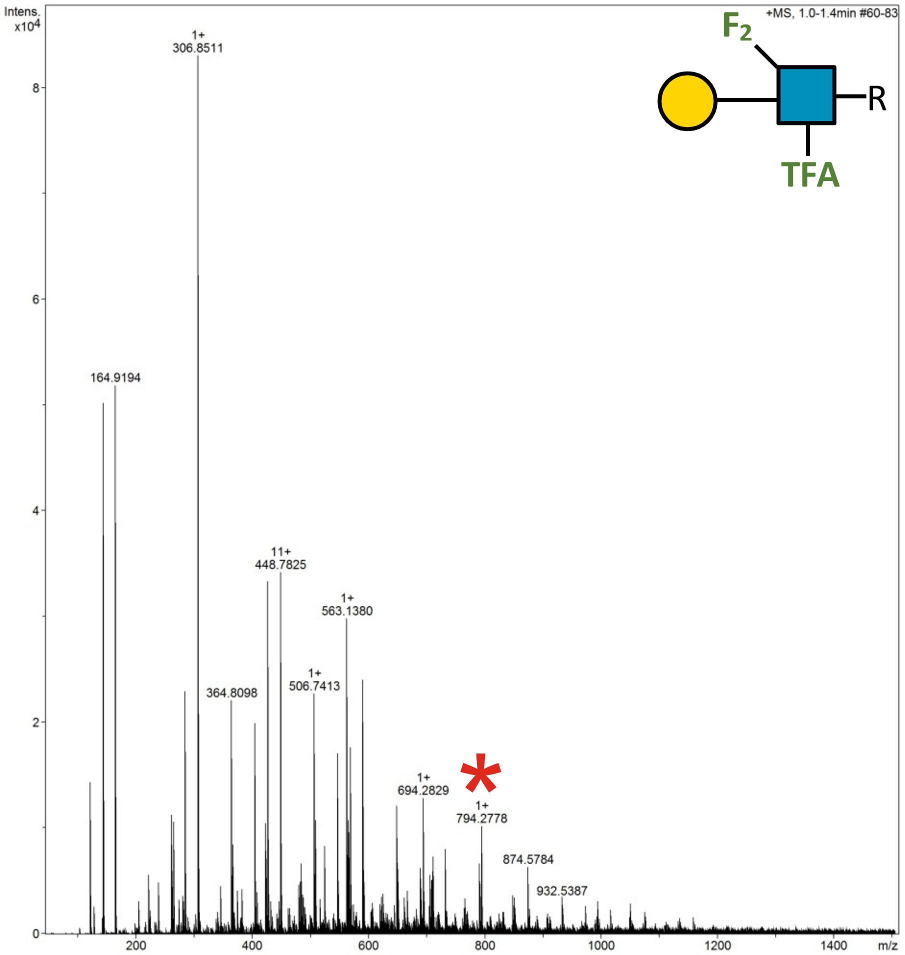

2.18.7 Supplementary Figure 24. HRMS ITag screening assay mass spectrum of synthesis of 3F-Gal β1-4 GlcNAc-ITag

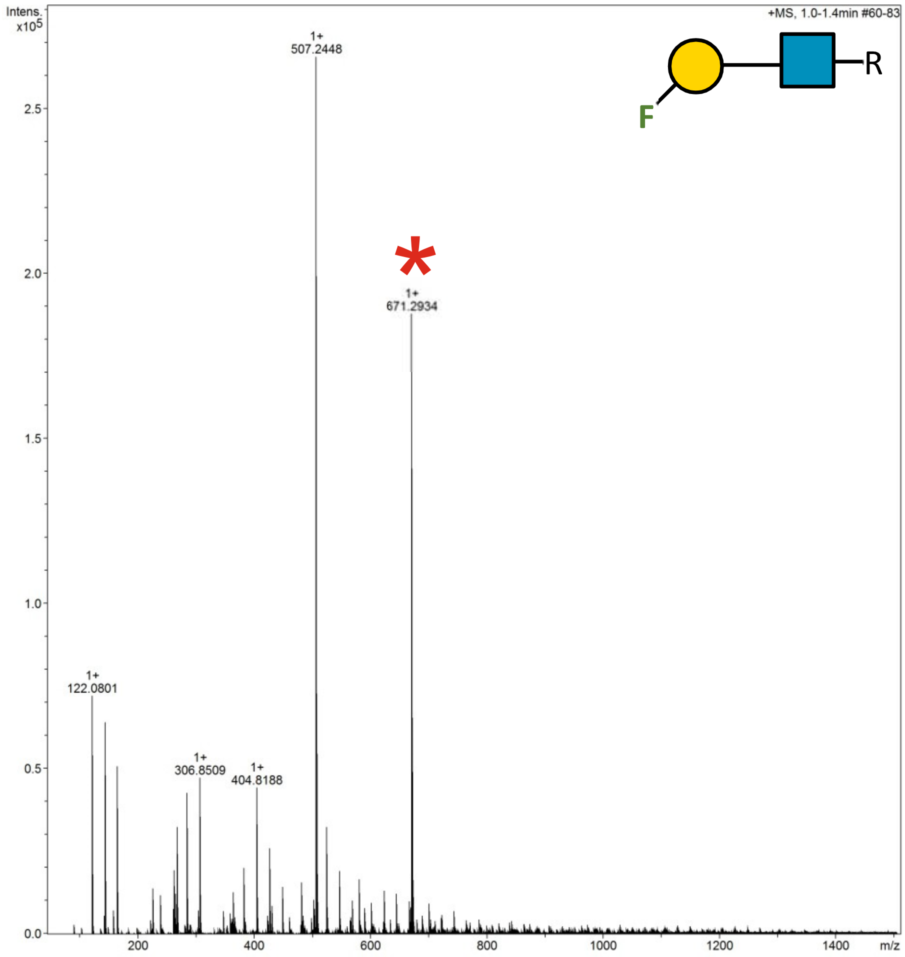

2.18.8 Supplementary Figure 25. HRMS ITag screening assay mass spectrum of synthesis of 3F-Gal β1-4 GlcNTFA-ITag

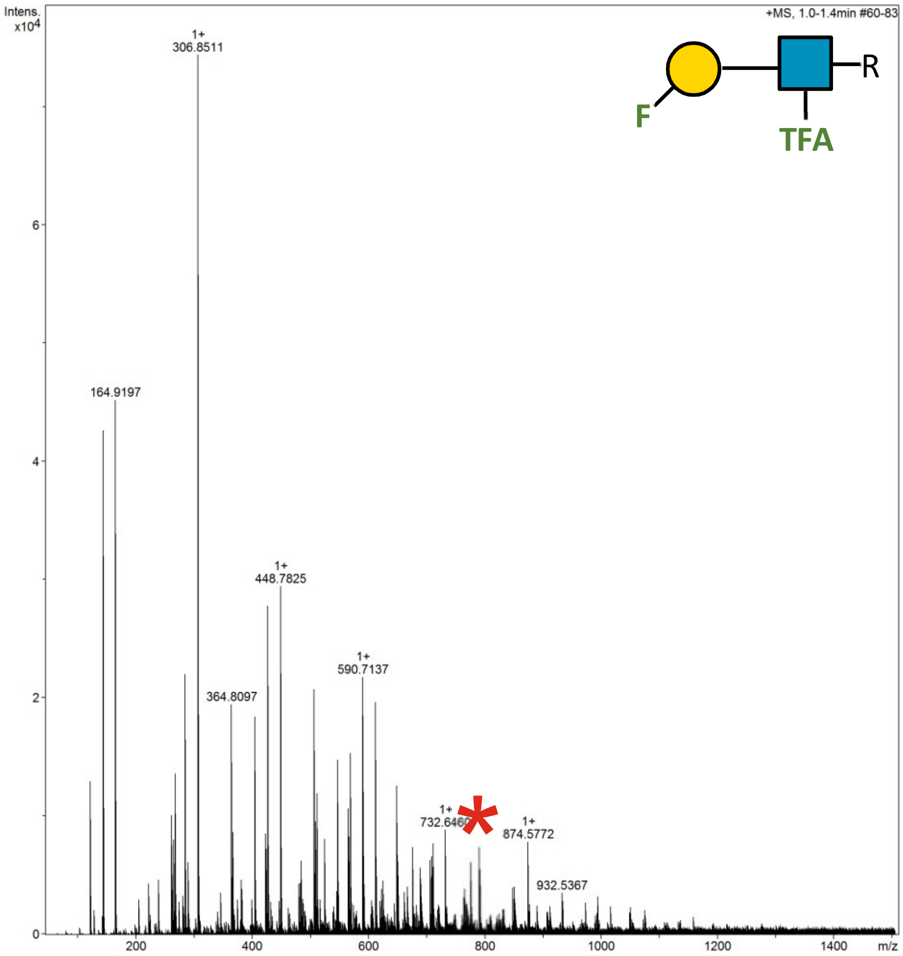

2.18.9 Supplementary Figure 26. HRMS ITag screening assay mass spectrum of synthesis of 3F-Gal β1-4 6F-GlcNAc-ITag

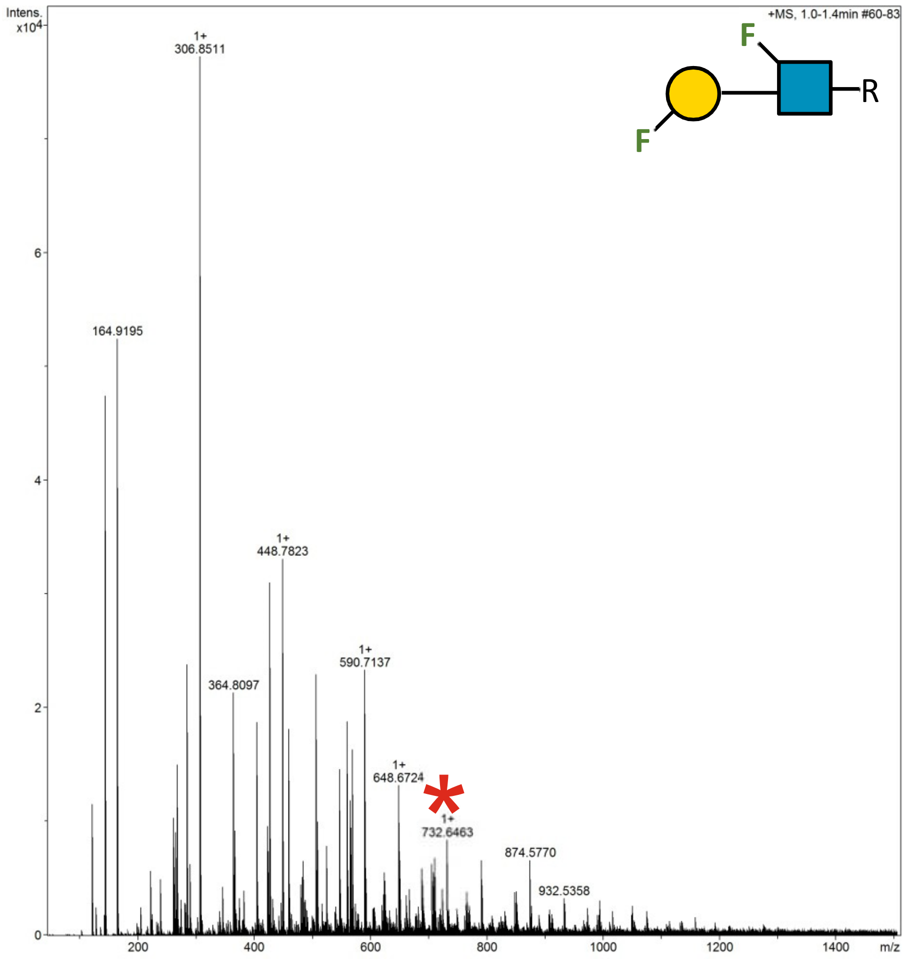

2.18.10 Supplementary Figure 27. HRMS ITag screening assay mass spectrum of synthesis of 3F-Gal β1-4 6F-GlcNTFA-ITag

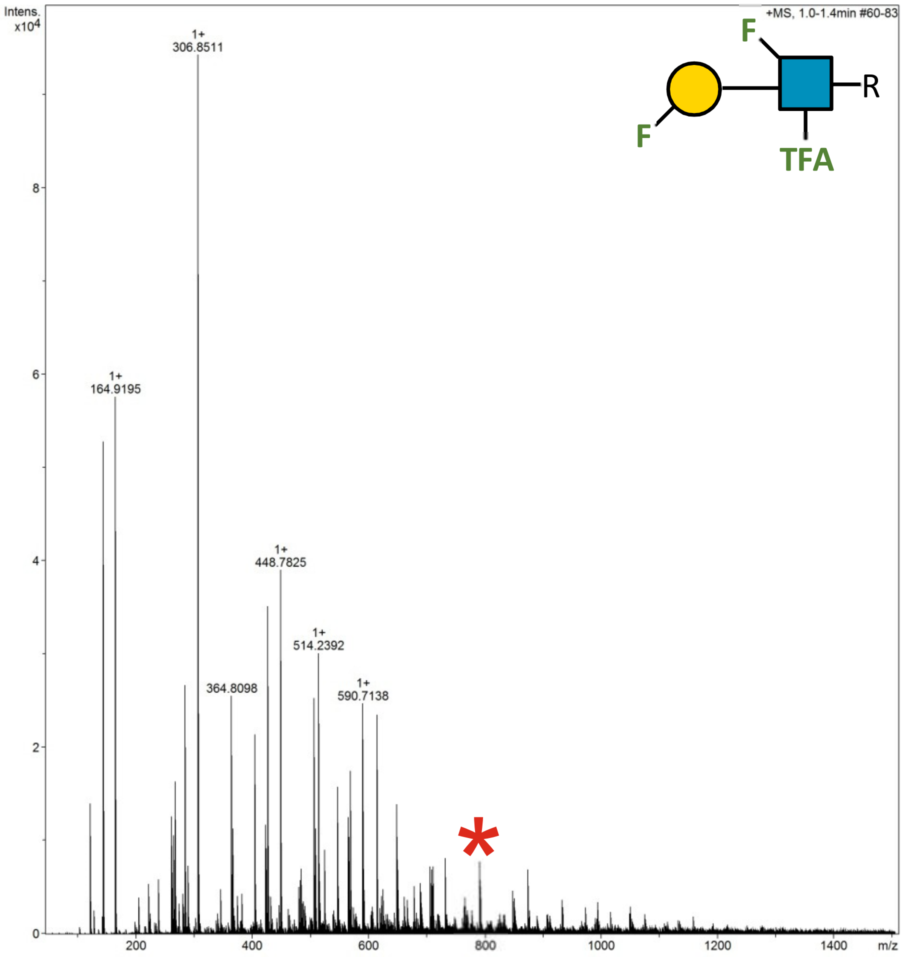

2.18.11 Supplementary Figure 28. HRMS ITag screening assay mass spectrum of synthesis of 3F-Gal β1-4 6,6-diFGlcNAc-ITag

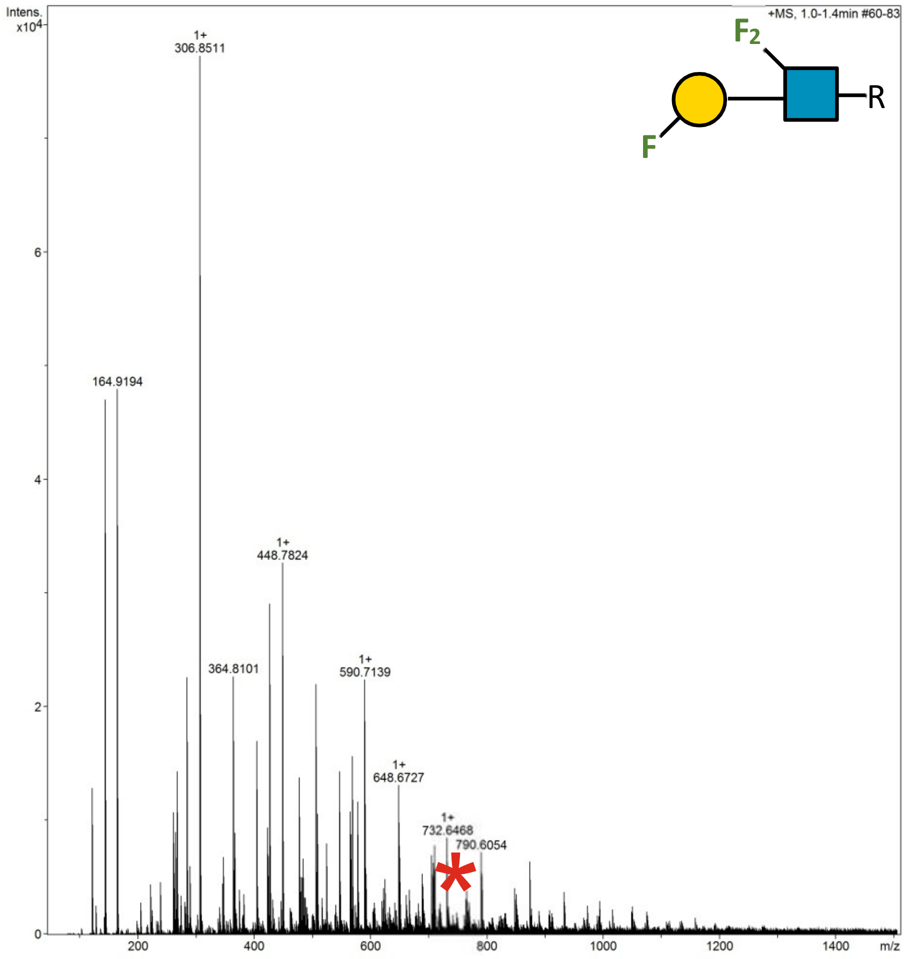

2.18.12 Supplementary Figure 29. HRMS ITag screening assay mass spectrum of synthesis of 3F-Gal β1-4 6,6-diFGlcNTFA-ITag

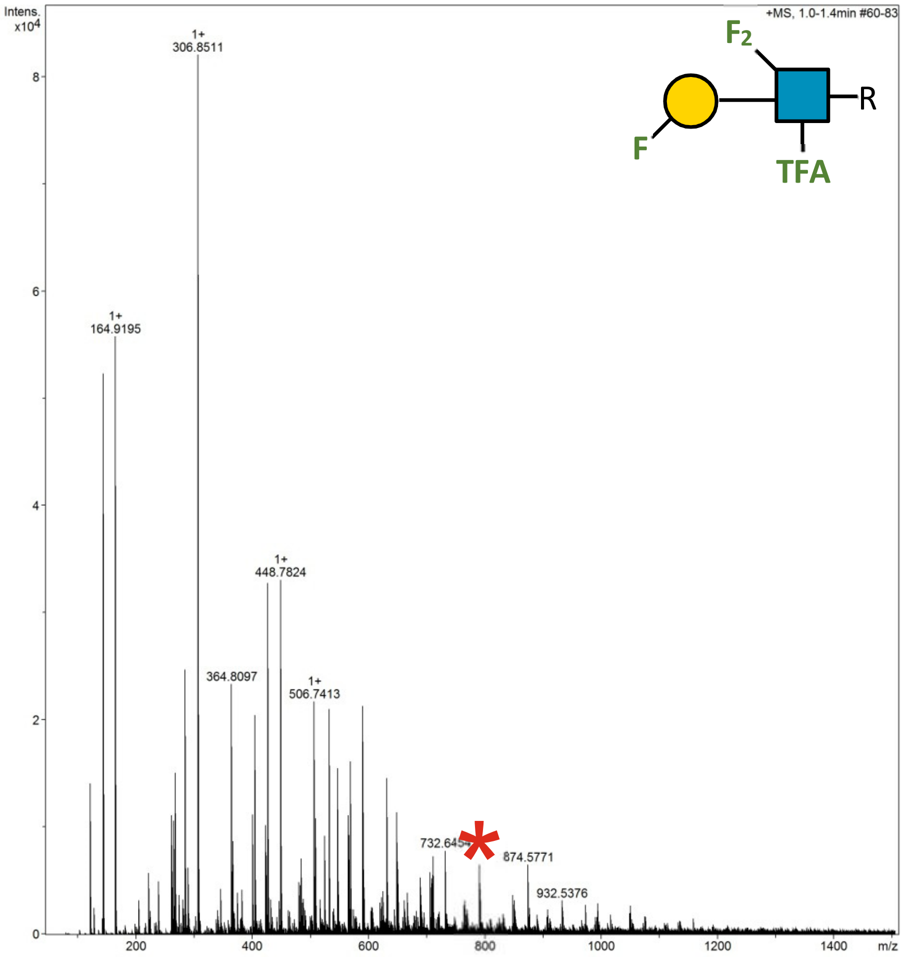

2.18.13 Supplementary Figure 30. HRMS ITag screening assay mass spectrum of synthesis of 4F-Gal β1-4 GlcNAc-ITag

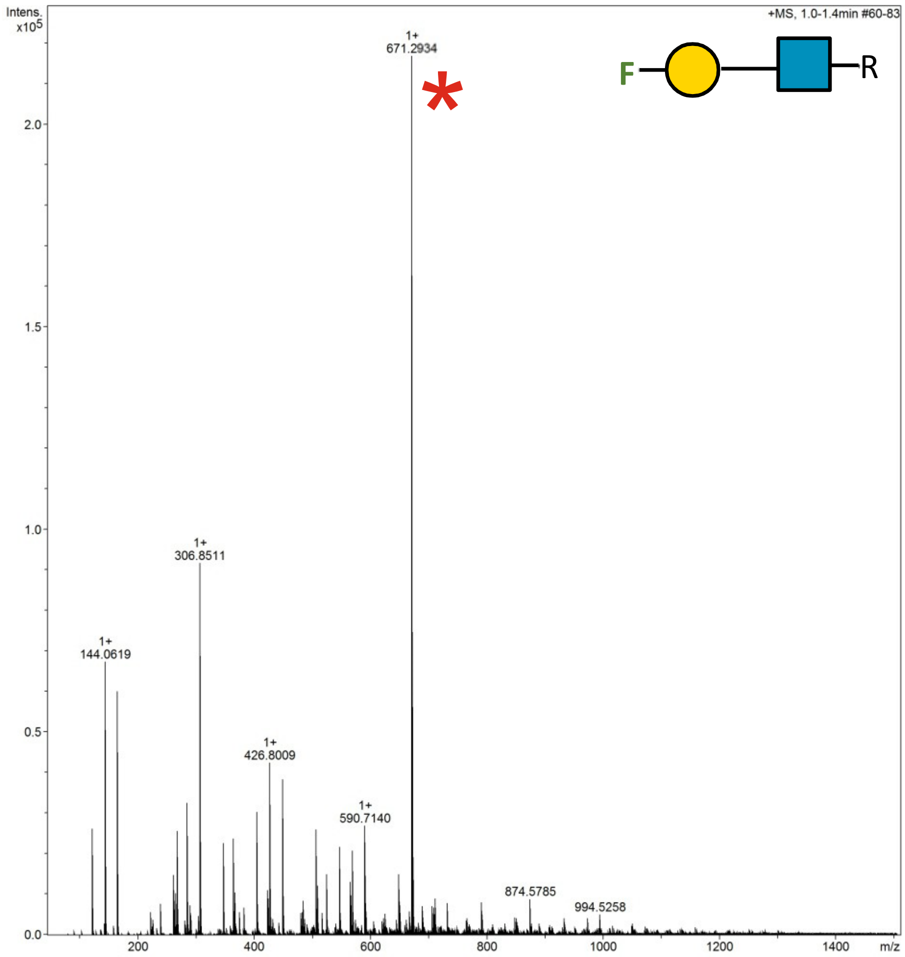

2.18.14 Supplementary Figure 31. HRMS ITag screening assay mass spectrum of synthesis of 4F-Gal β1-4 GlcNTFA-ITag

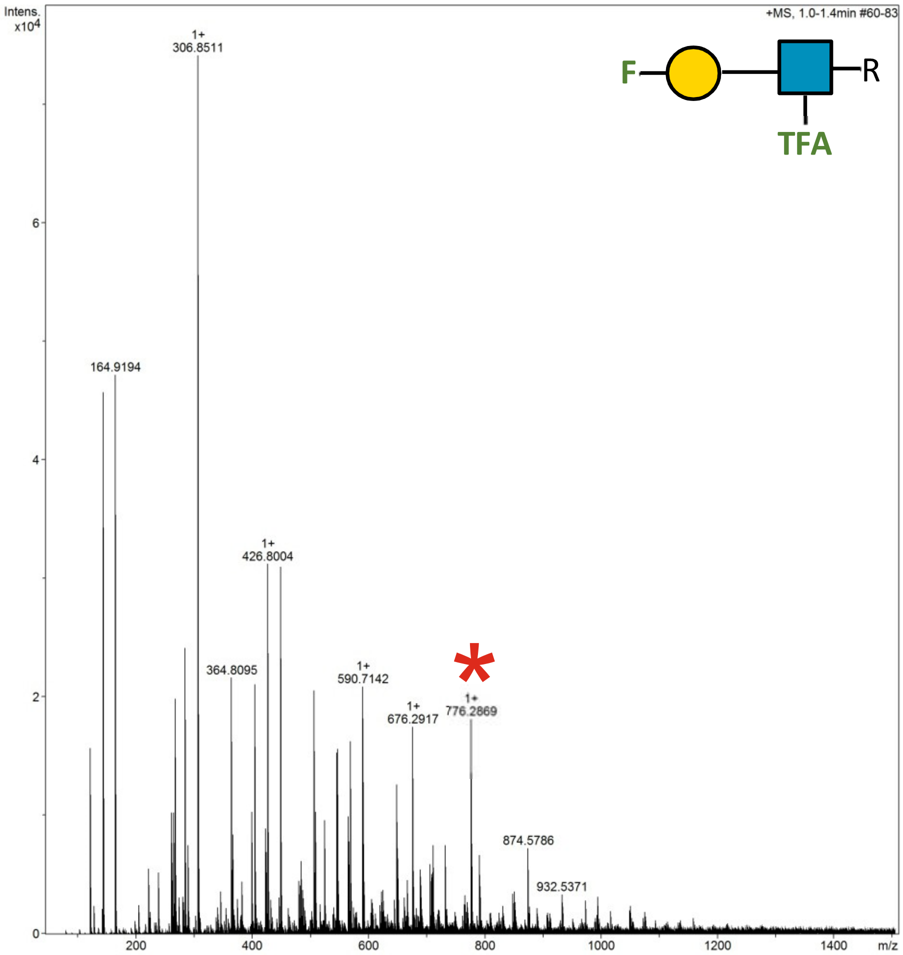

2.18.15 Supplementary Figure 32. HRMS ITag screening assay mass spectrum of synthesis of 4F-Gal β1-4 6F-GlcNAc-ITag

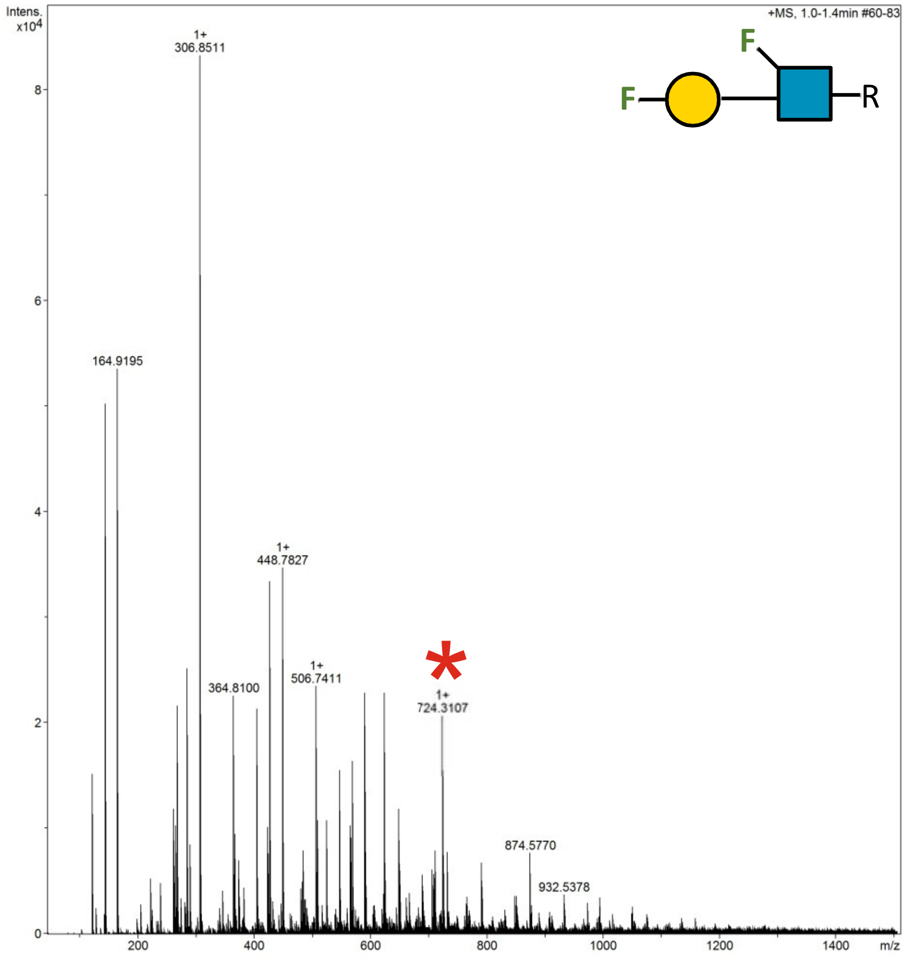

2.18.16 Supplementary Figure 33. HRMS ITag screening assay mass spectrum of synthesis of 4F-Gal β1-4 6F-GlcNTFA-ITag

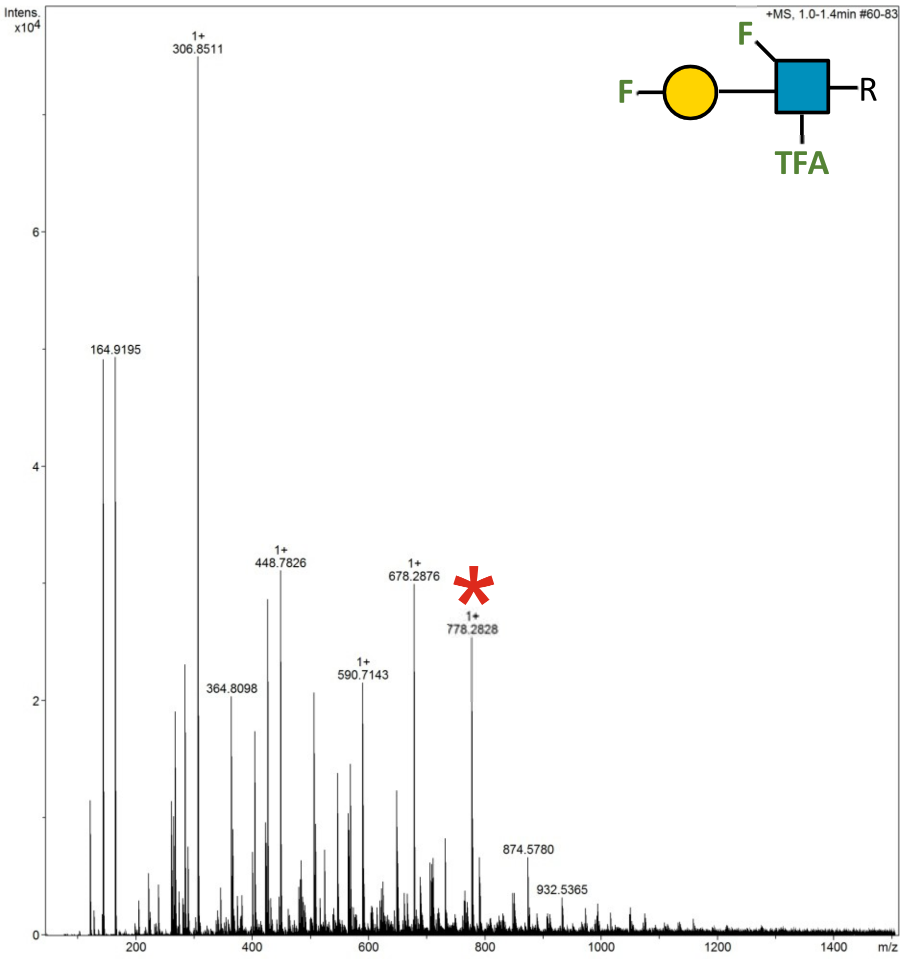

2.18.17 Supplementary Figure 34. HRMS ITag screening assay mass spectrum of synthesis of 4F-Gal β1-4 6,6-diFGlcNAc-ITag

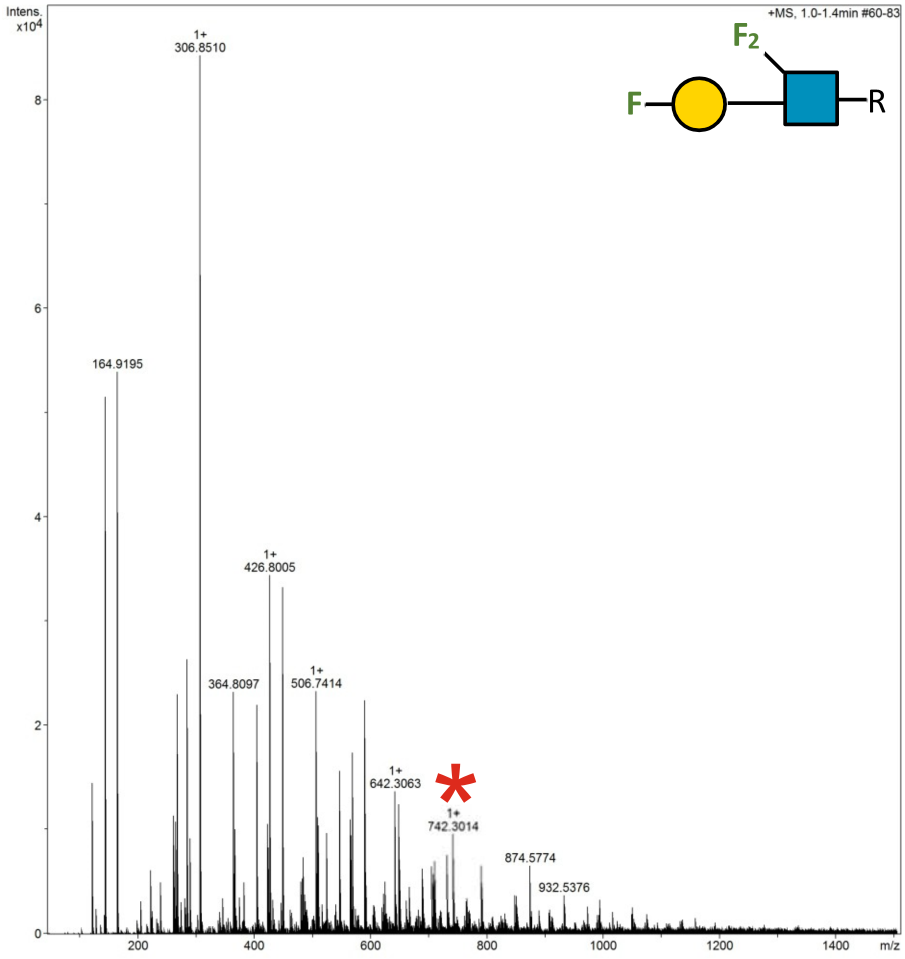

2.18.18 Supplementary Figure 35. HRMS ITag screening assay mass spectrum of synthesis of 4F-Gal β1-4 6,6-diFGlcNTFA-ITag

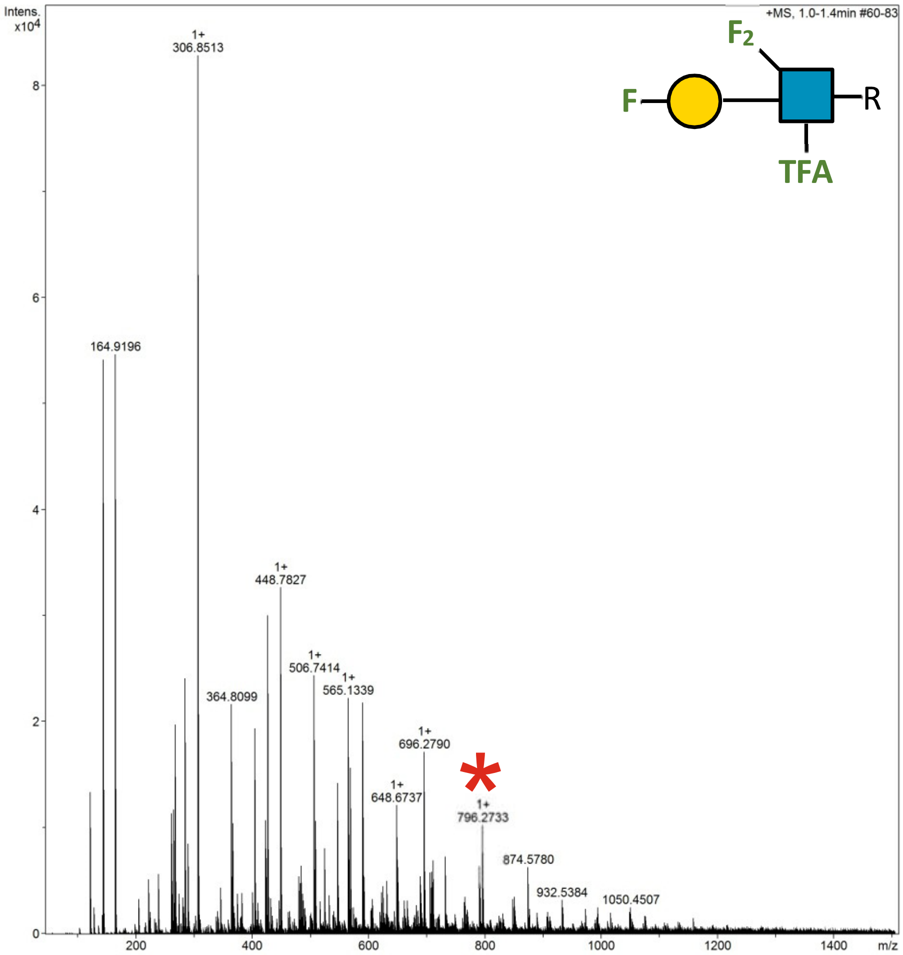

2.18.19 Supplementary Figure 36. HRMS ITag screening assay mass spectrum of synthesis of 6F-Gal β1-4 GlcNAc-ITag

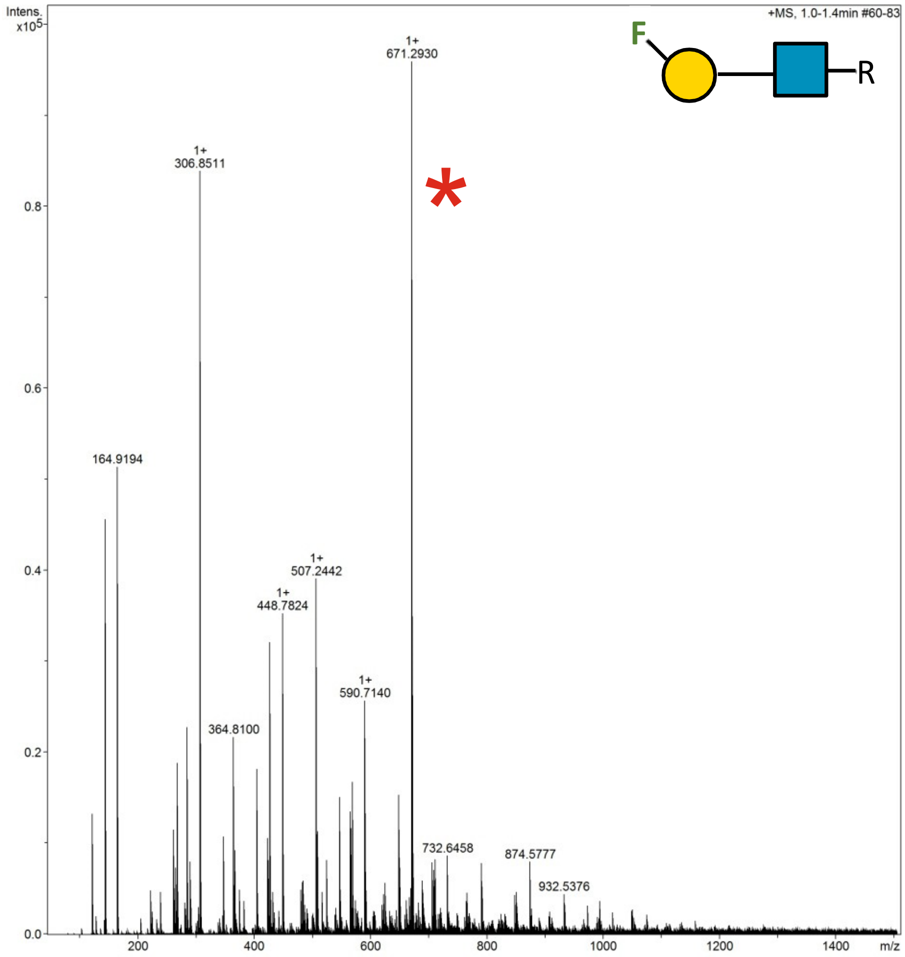

2.18.20 Supplementary Figure 37. HRMS ITag screening assay mass spectrum of synthesis of 6F-Gal β1-4 GlcNTFA-ITag

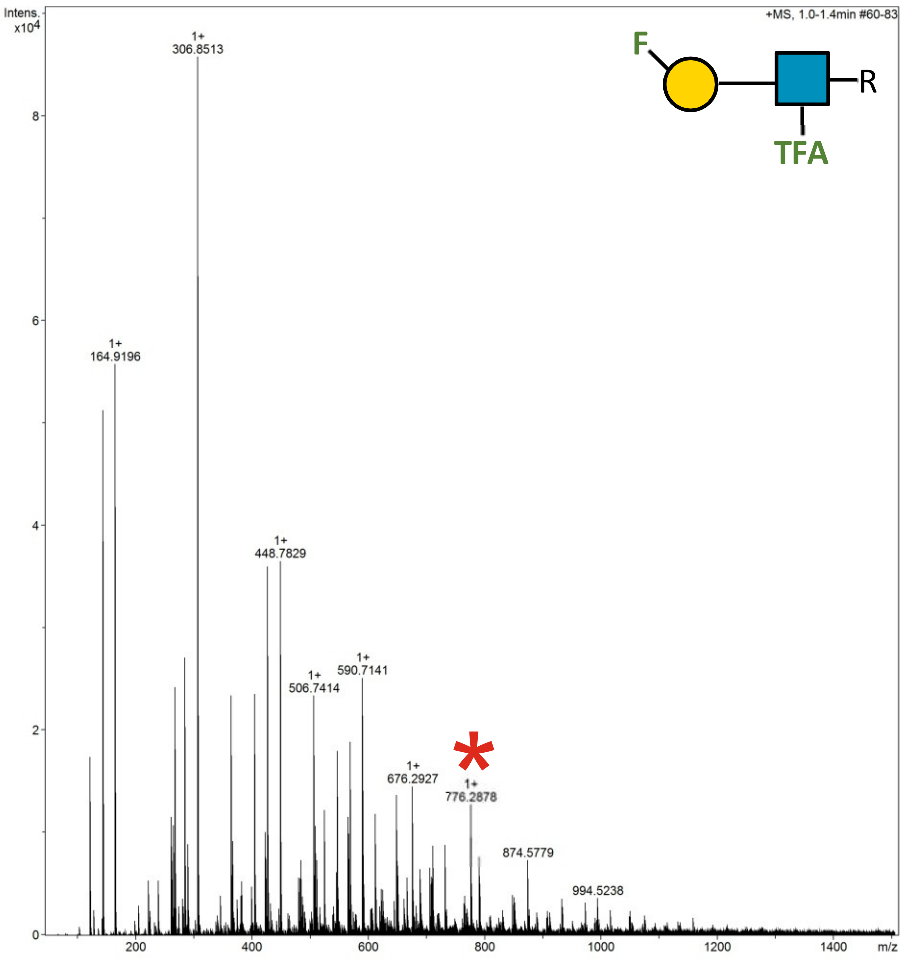

2.18.21 Supplementary Figure 38. HRMS ITag screening assay mass spectrum of synthesis of 6F-Gal β1-4 6F-GlcNAc-ITag

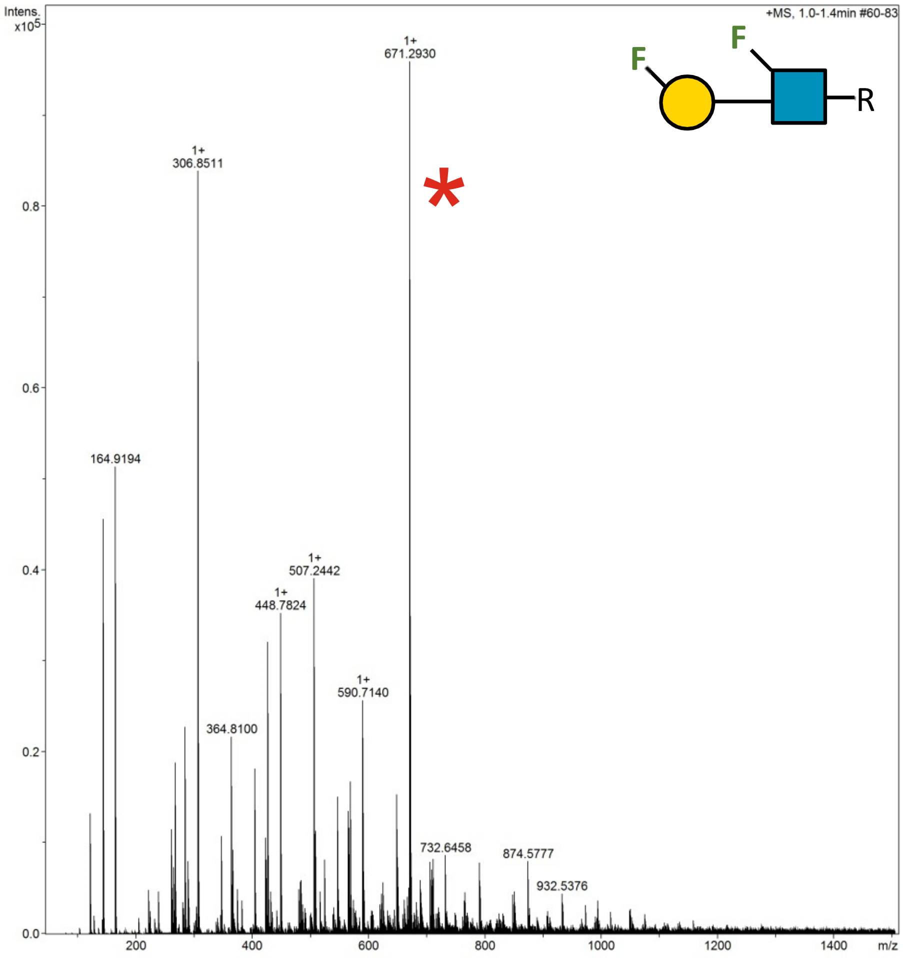

2.18.22 Supplementary Figure 39. HRMS ITag screening assay mass spectrum of synthesis of 6F-Gal β1-4 6F-GlcNTFA-ITag

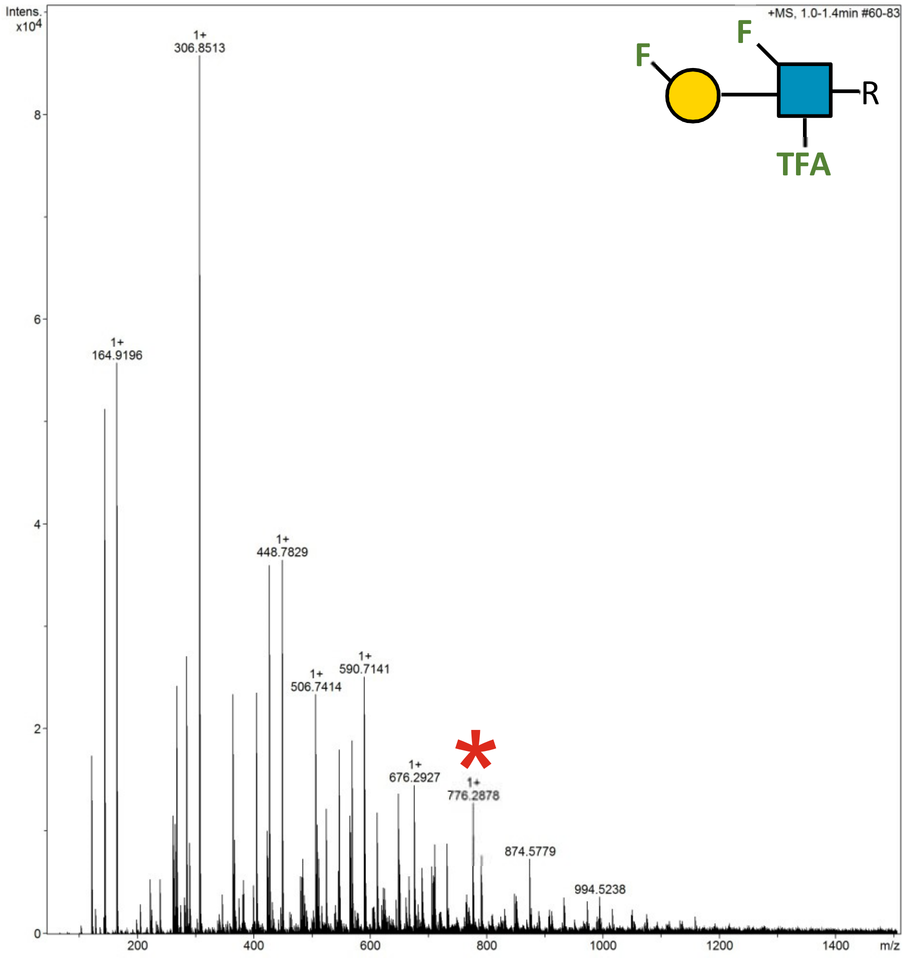

2.18.23 Supplementary Figure 40. HRMS ITag screening assay mass spectrum of synthesis of 6F-Gal β1-4 6,6-diFGlcNAc-ITag

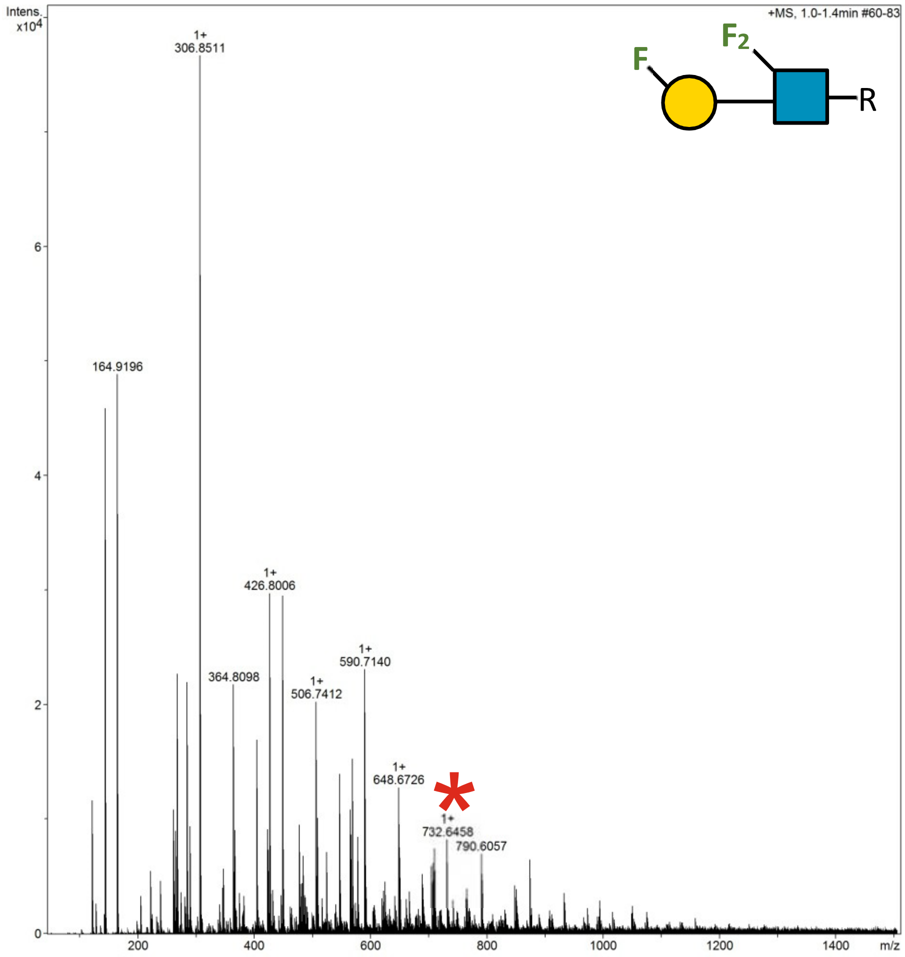

2.18.24 Supplementary Figure 41. HRMS ITag screening assay mass spectrum of synthesis of 6F-Gal β1-4 6,6-diFGlcNTFA-ITag

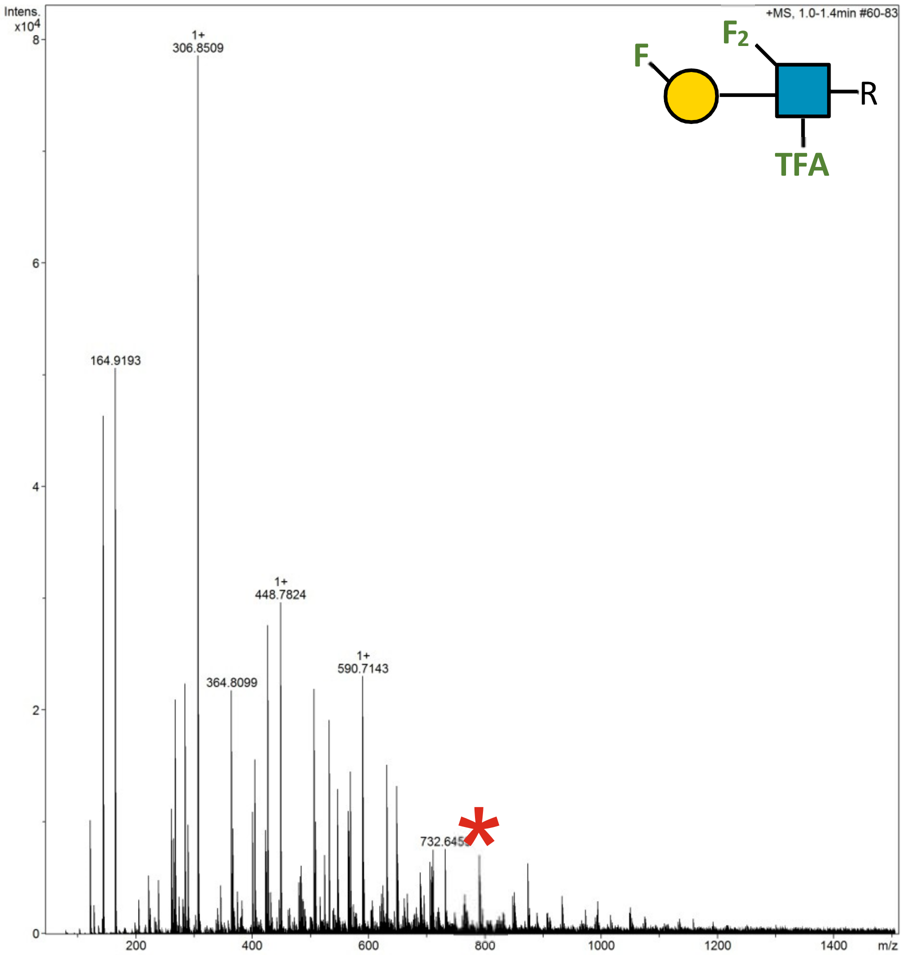

2.18.25 Supplementary Figure 42. HRMS ITag screening assay mass spectrum of synthesis of 6d-Gal β1-4 GlcNAc-ITag

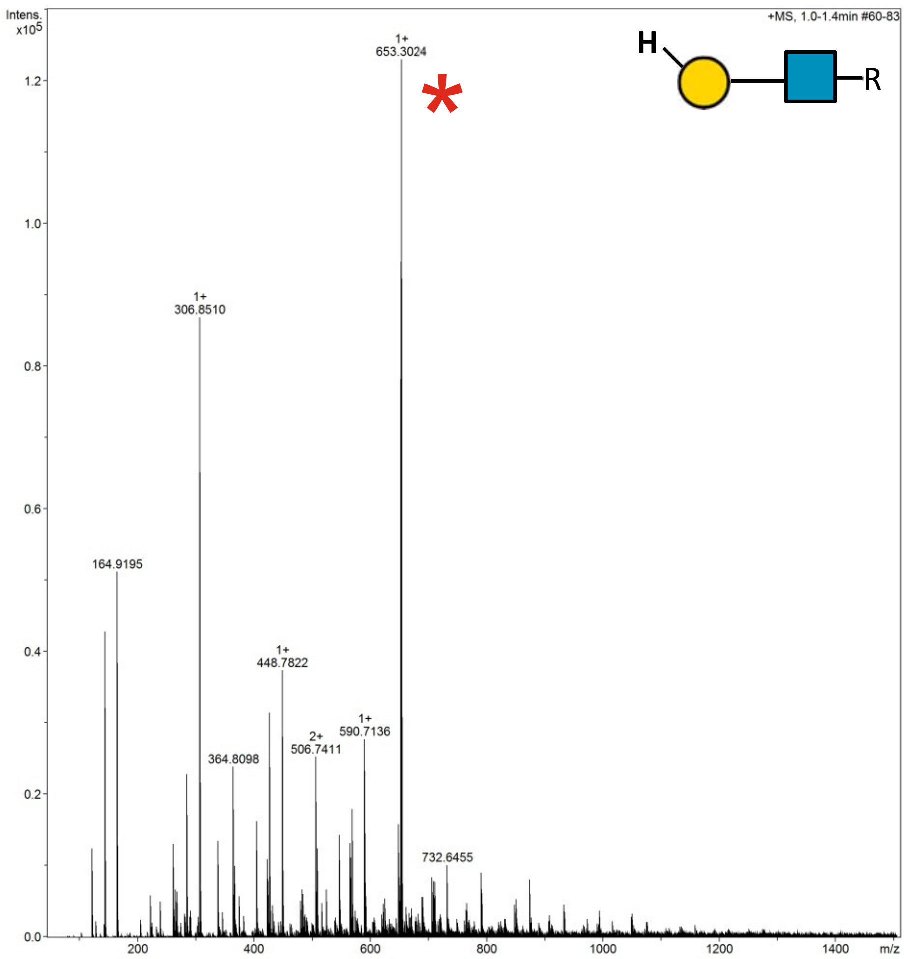

2.18.26 Supplementary Figure 43. HRMS ITag screening assay mass spectrum of synthesis of 6d-Gal β1-4 GlcNTFA-ITag

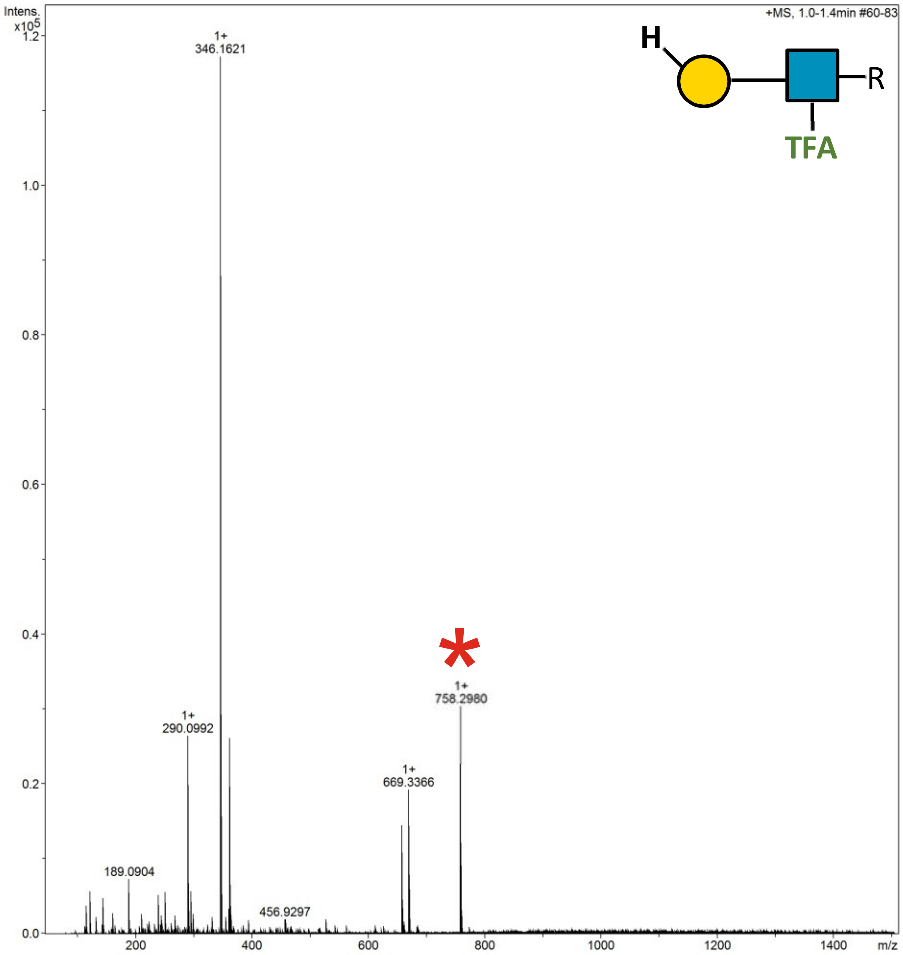

2.18.27 Supplementary Figure 44. HRMS ITag screening assay mass spectrum of synthesis of 6d-Gal β1-4 6F-GlcNAc-ITag

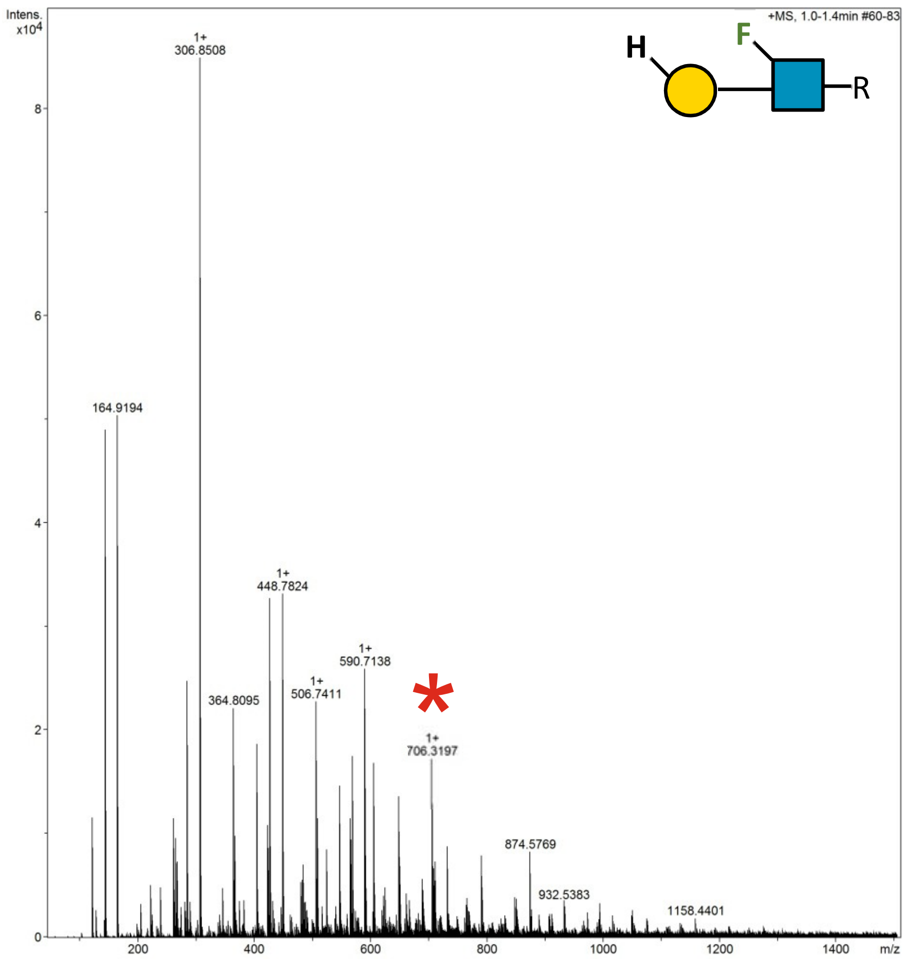

2.18.28 Supplementary Figure 45. HRMS ITag screening assay mass spectrum of synthesis of 6d-Gal β1-4 6F-GlcNTFA-ITag

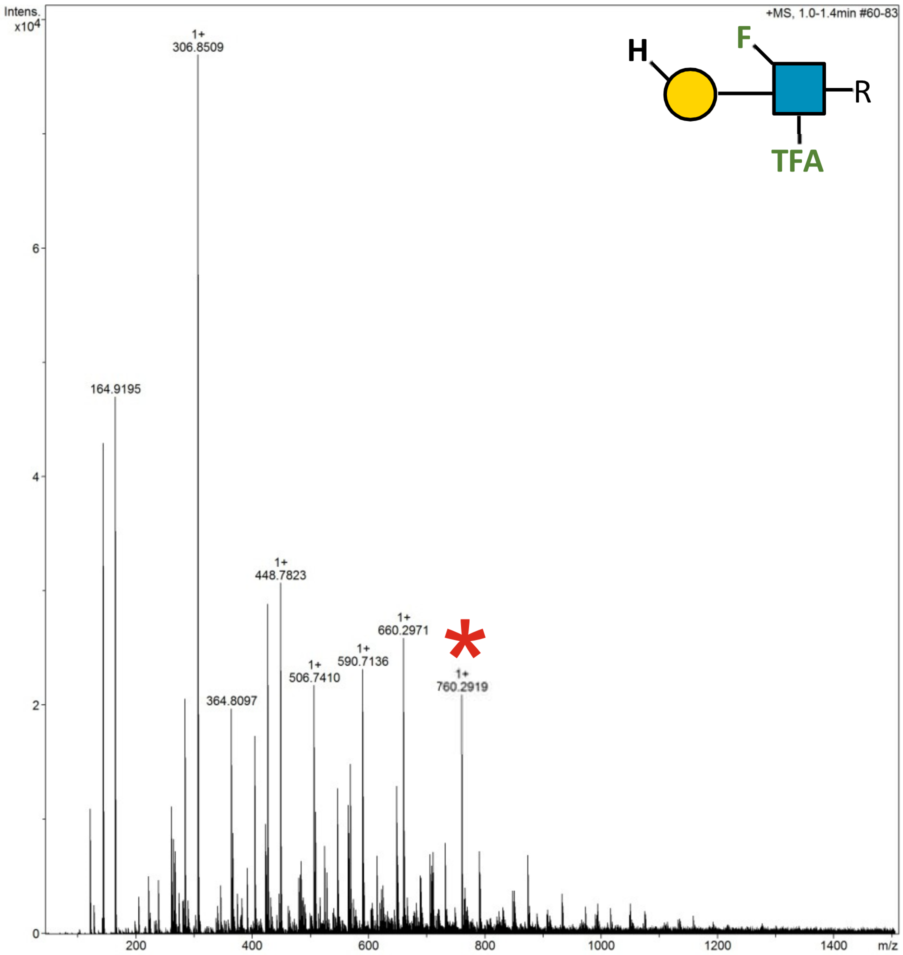

2.18.29 Supplementary Figure 46. HRMS ITag screening assay mass spectrum of synthesis of 6d-Gal β1-4 6,6-diFGlcNAc-ITag

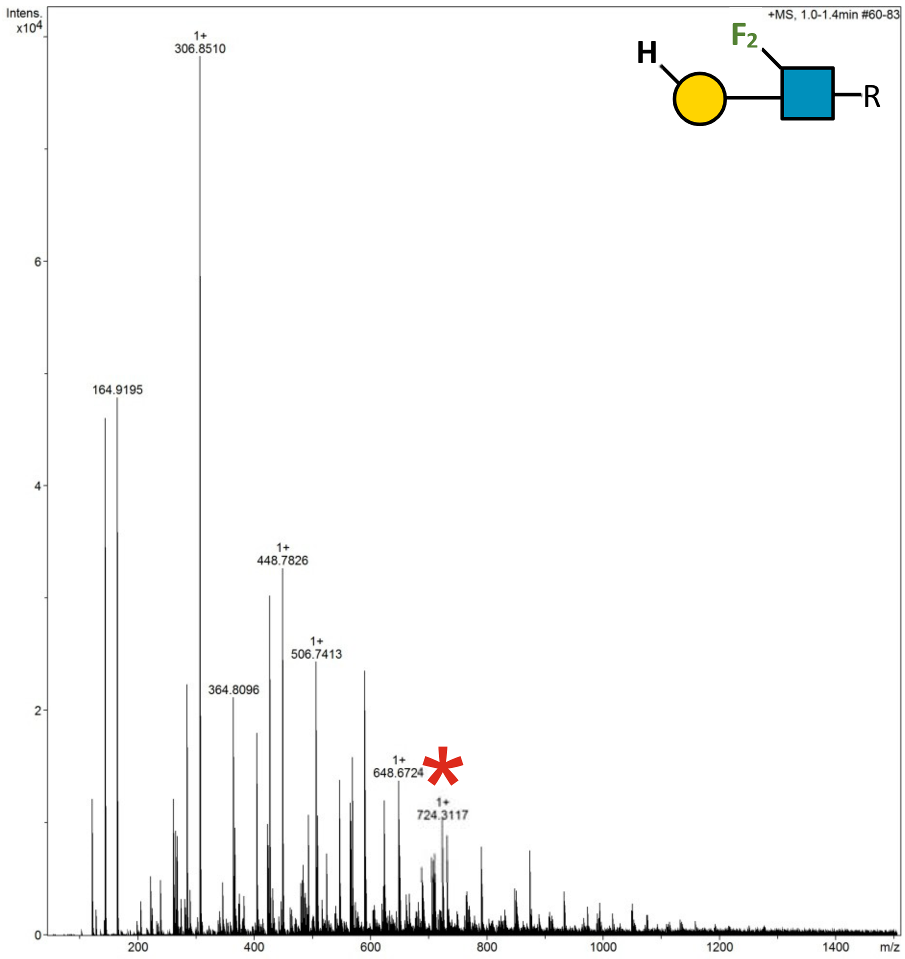

2.18.30 Supplementary Figure 47. HRMS ITag screening assay mass spectrum of synthesis of 6d-Gal β1-4 6,6-diFGlcNTFA-ITag

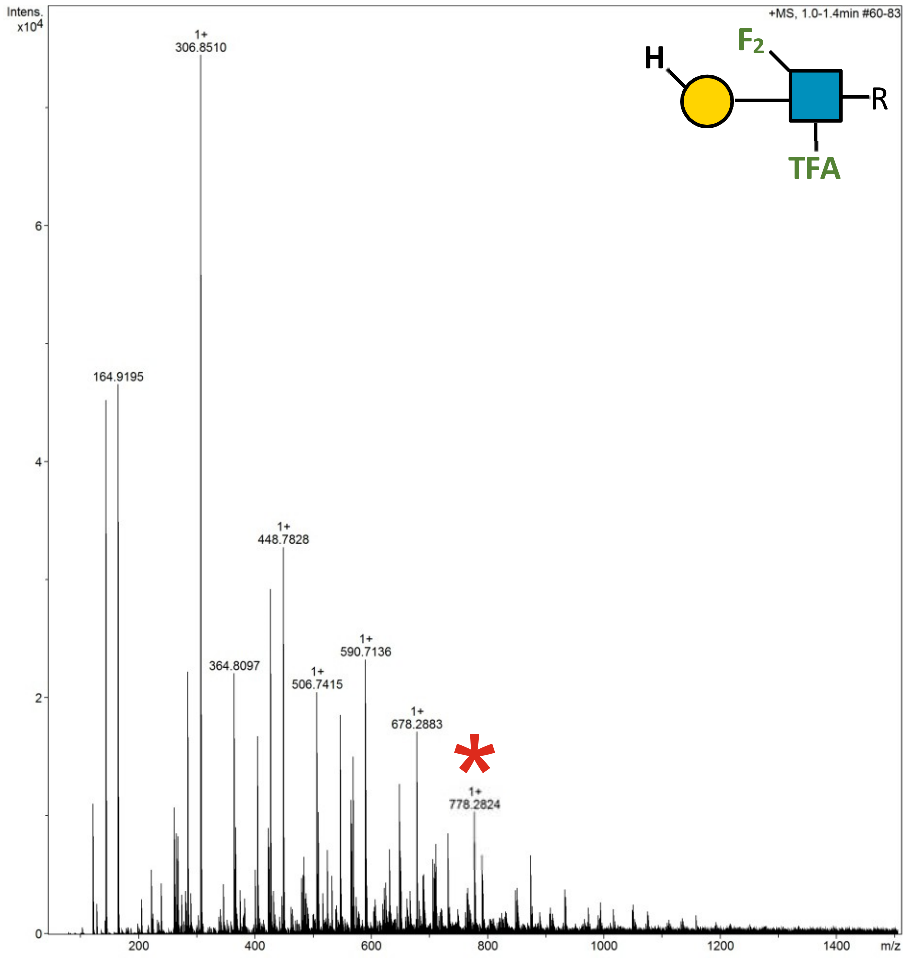

2.18.31 Supplementary Figure 48. HRMS ITag screening assay mass spectrum of synthesis of Gal β1-4 (Fuc α1-3) GlcNAc-ITag (LeX1)

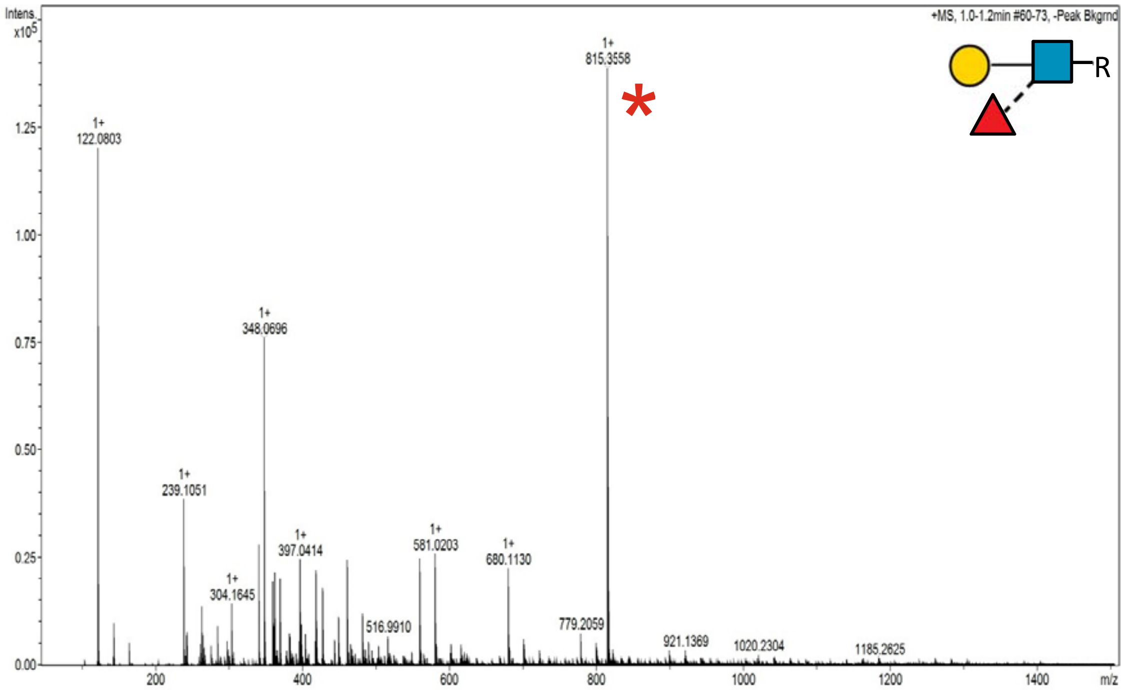

2.18.32 Supplementary Figure 49. HRMS ITag screening assay mass spectrum of synthesis of Gal β1-4 (Fuc α1-3) GlcNTFA-ITag (LeX4)

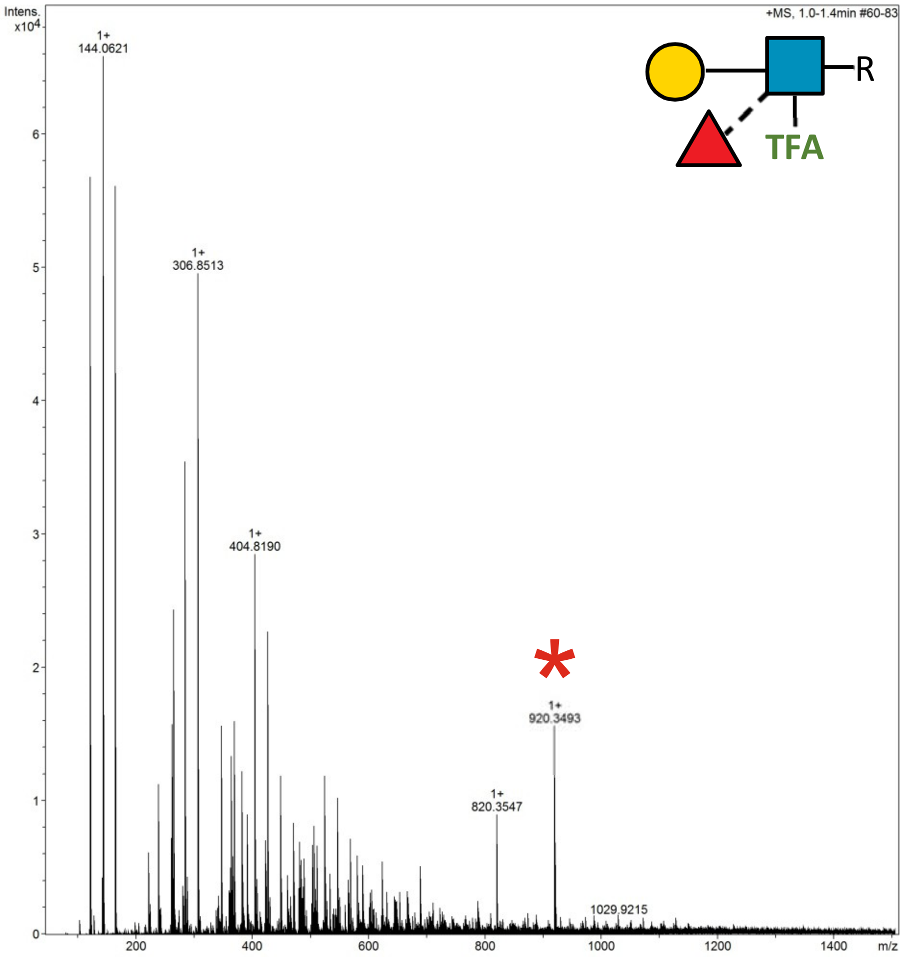

2.18.33 Supplementary Figure 50. HRMS ITag screening assay mass spectrum of synthesis of Gal β1-4 (Fuc α1-3) 6F-GlcNAc-ITag (LeX2)

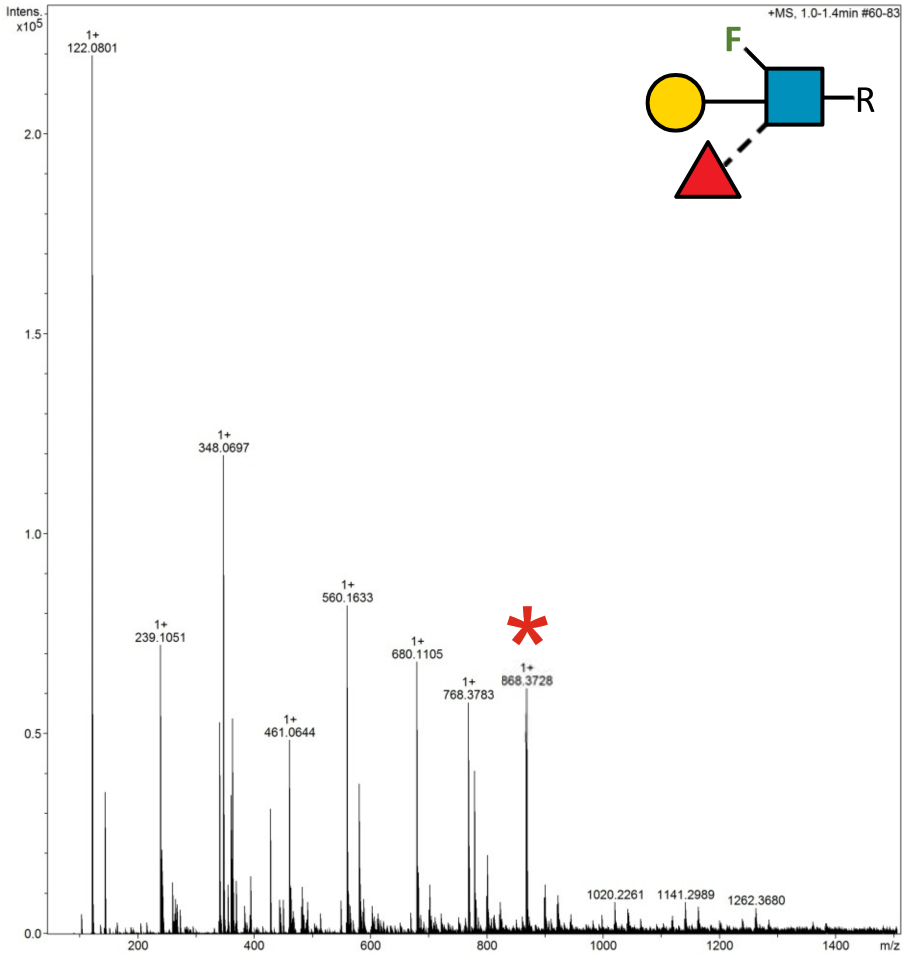

2.18.34 Supplementary Figure 51. HRMS ITag screening assay mass spectrum of synthesis of Gal β1-4 (Fuc α1-3) 6F-GlcNTFA-ITag (LeX5)

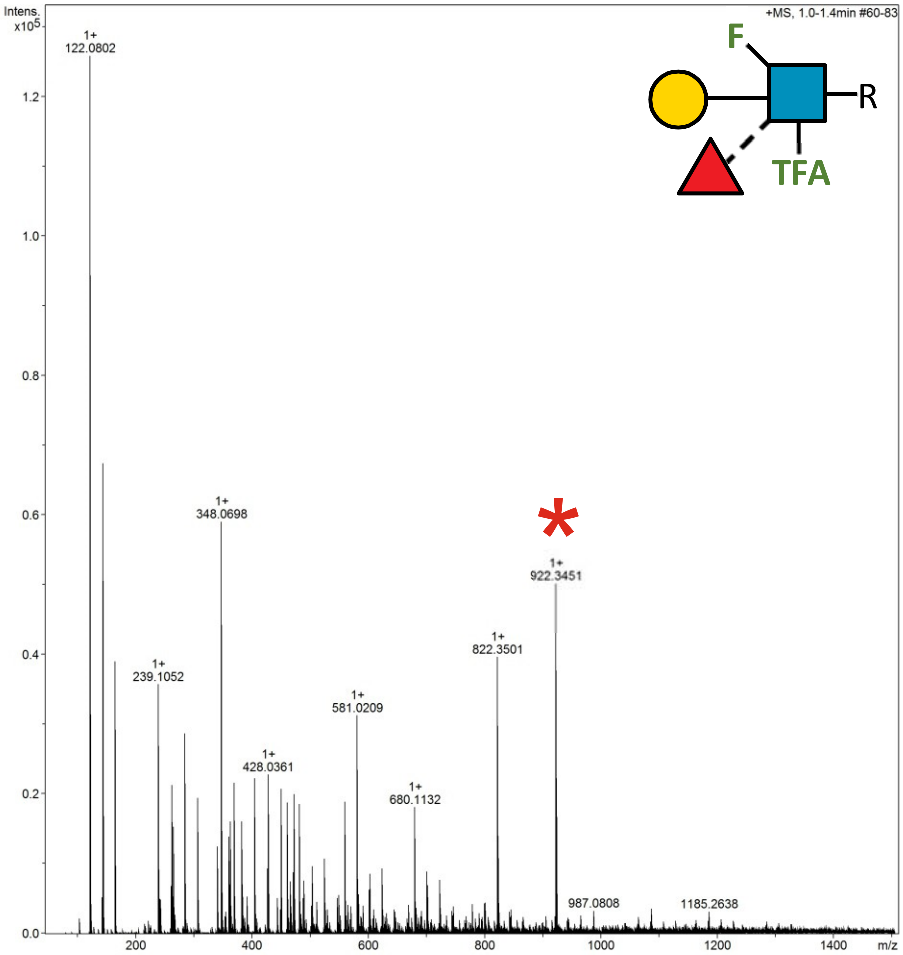

2.18.35 Supplementary Figure 52. HRMS ITag screening assay mass spectrum of synthesis of Gal β1-4 (Fuc α1-3) 6,6-diFGlcNAc-ITag (LeX3)

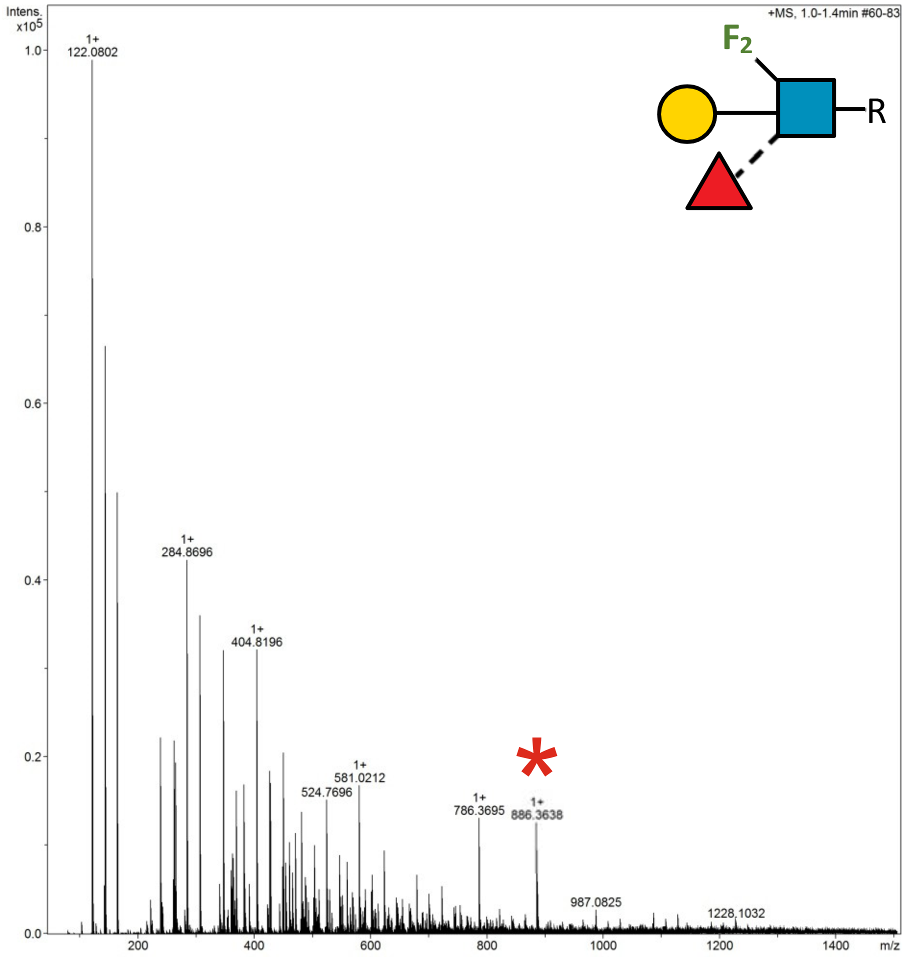

2.18.36 Supplementary Figure 53. HRMS ITag screening assay mass spectrum of synthesis of Gal β1-4 (Fuc α1-3) 6,6-diFGlcNTFA-ITag (LeX6)

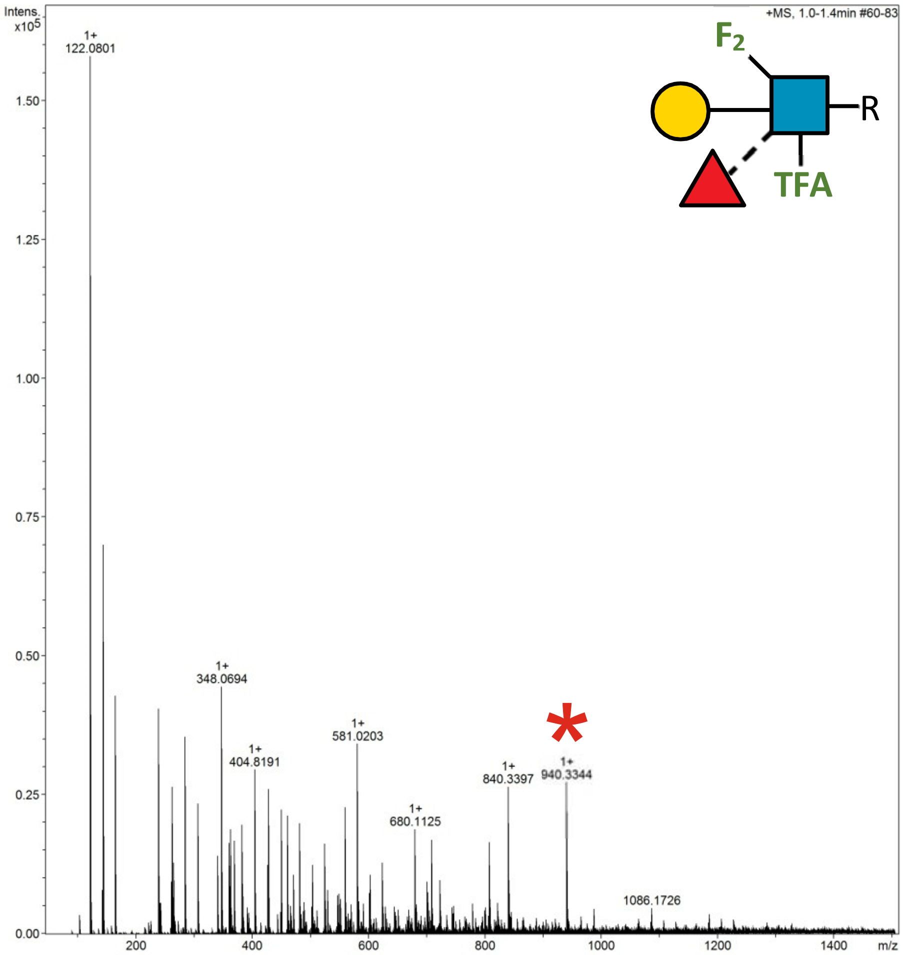

2.18.37 Supplementary Figure 54. HRMS ITag screening assay mass spectrum of synthesis of 3F-Gal β1-4 (Fuc α1-3) GlcNAc-ITag (LeX7)

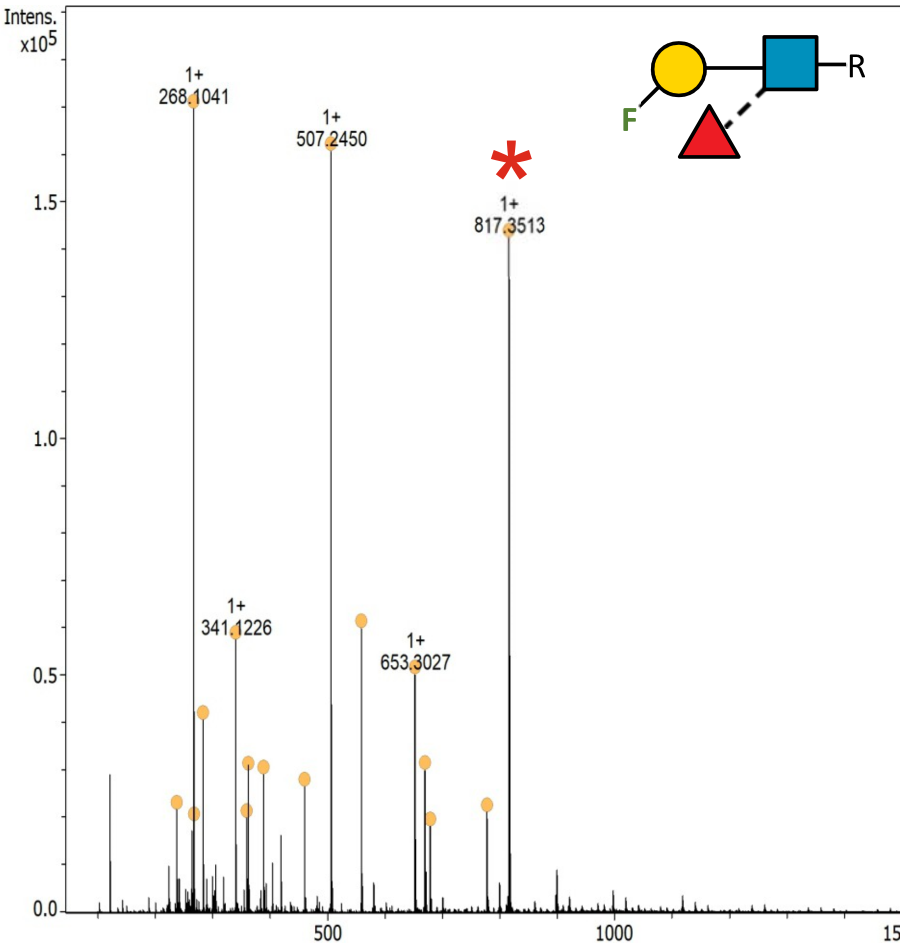

2.18.38 Supplementary Figure 55. HRMS ITag screening assay mass spectrum of synthesis of 3F-Gal β1-4 (Fuc α1-3) GlcNTFA-ITag

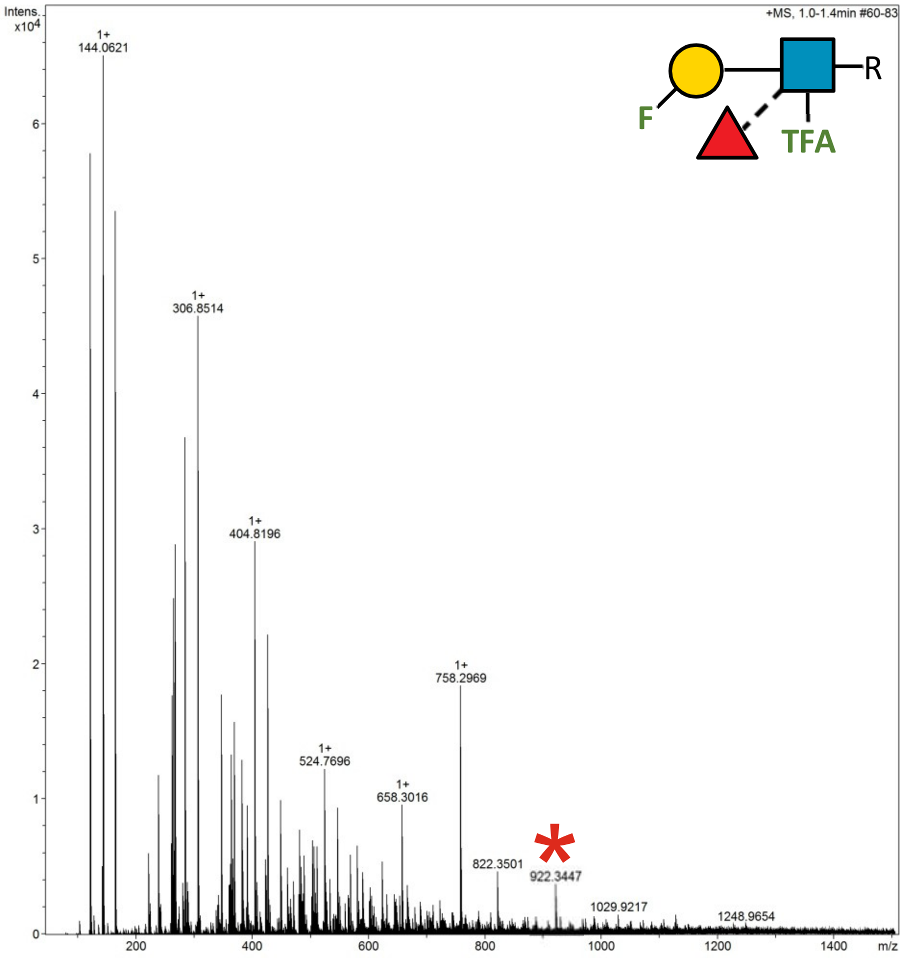

2.18.39 Supplementary Figure 56. HRMS ITag screening assay mass spectrum of synthesis of 3F-Gal β1-4 (Fuc α1-3) 6F-GlcNAc-ITag

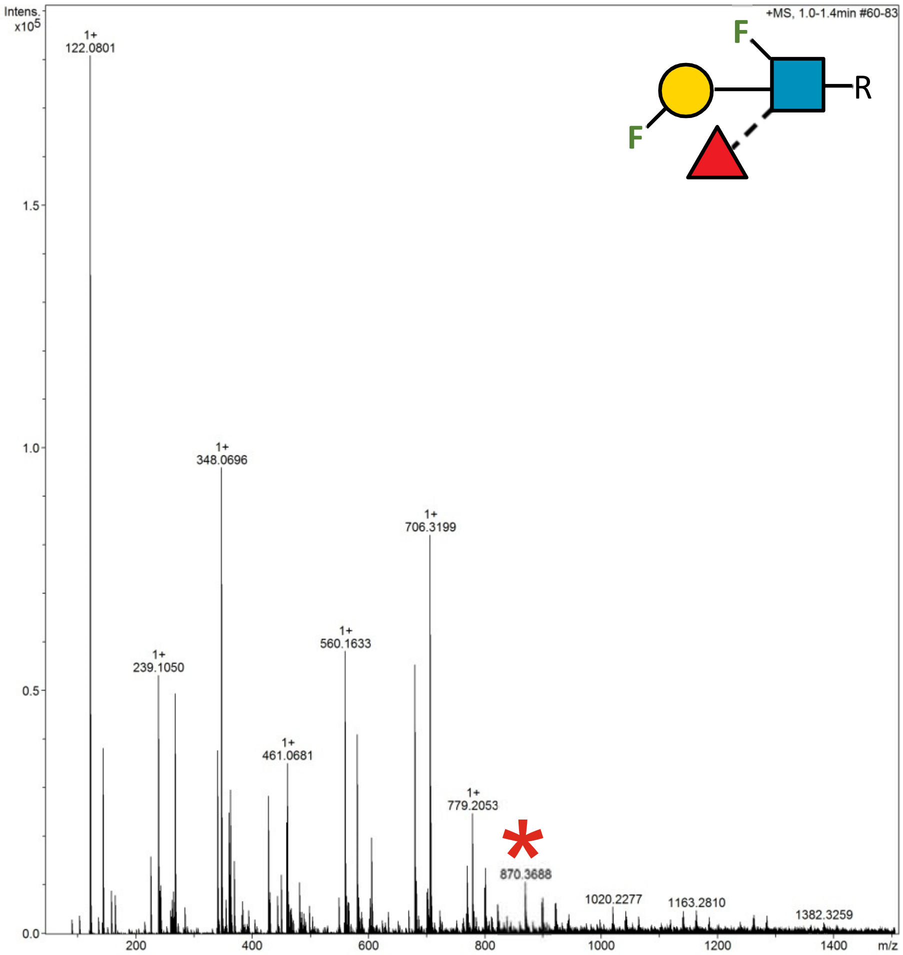

2.18.40 Supplementary Figure 57. HRMS ITag screening assay mass spectrum of synthesis of 3F-Gal β1-4 (Fuc α1-3) 6F-GlcNTFA-ITag

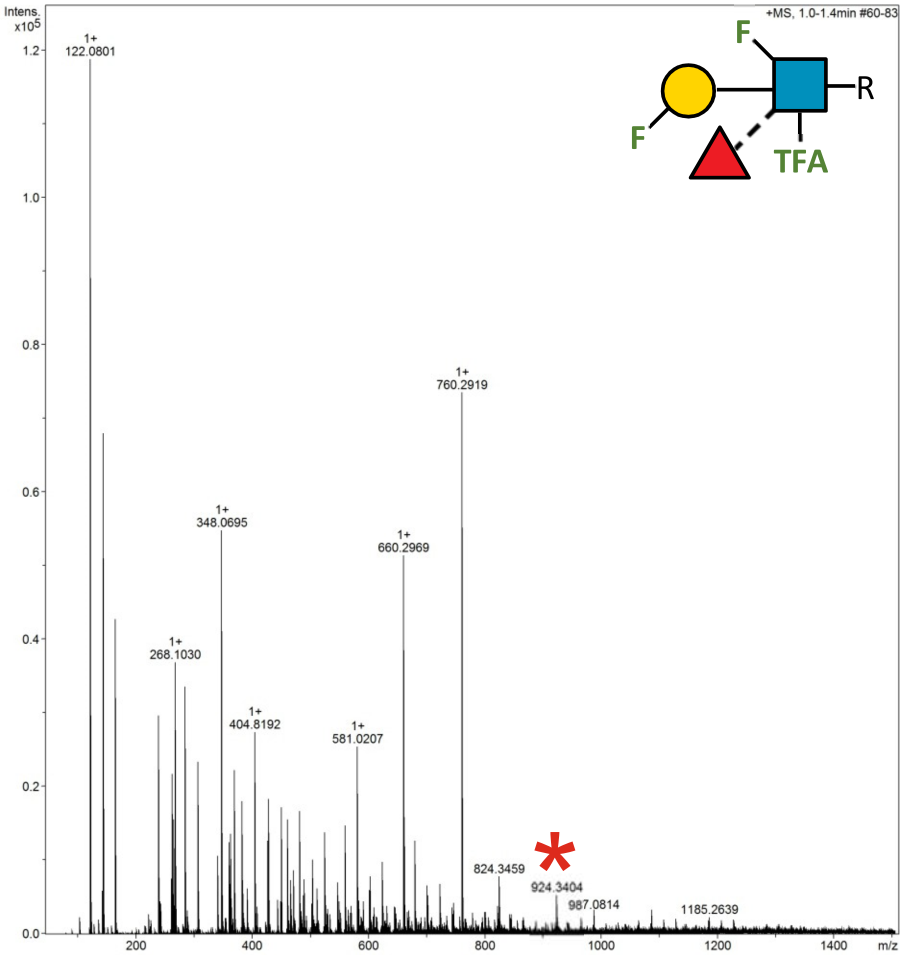

2.18.41 Supplementary Figure 58. HRMS ITag screening assay mass spectrum of synthesis of 3F-Gal β1-4 (Fuc α1-3) 6,6-diFGlcNAc-ITag

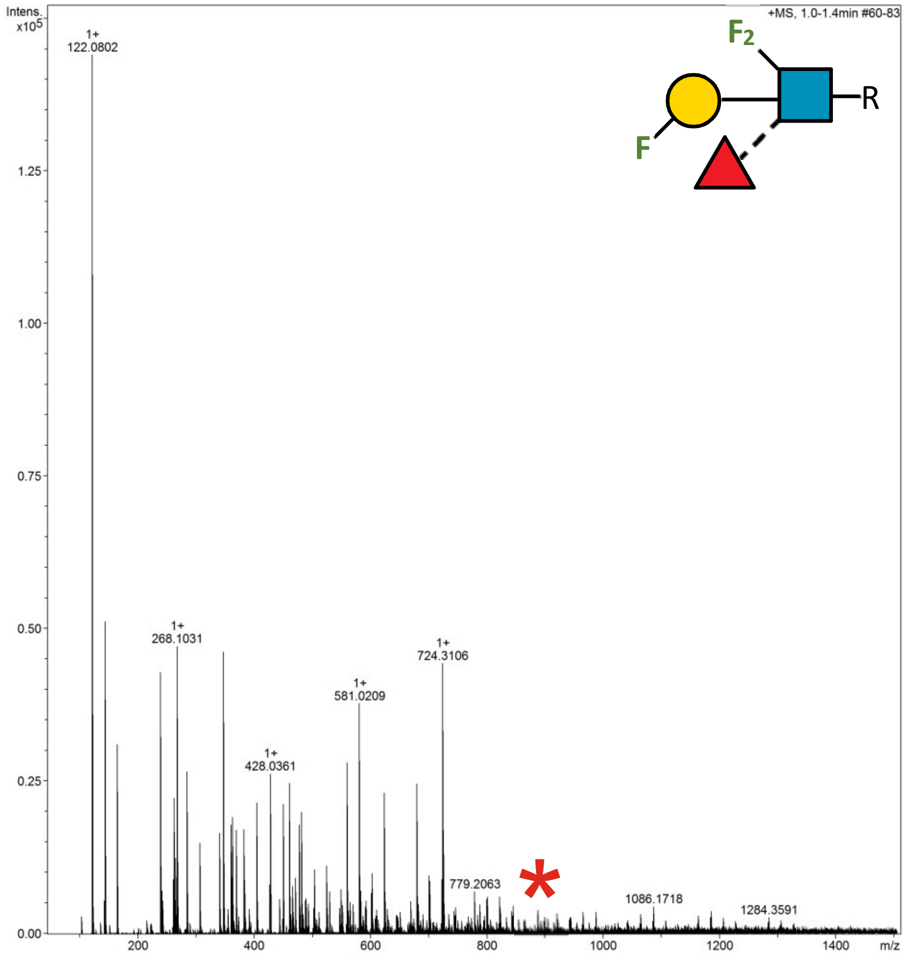

2.18.42 Supplementary Figure 59. HRMS ITag screening assay mass spectrum of synthesis of 3F-Gal β1-4 (Fuc α1-3) 6,6-diFGlcNTFA-ITag

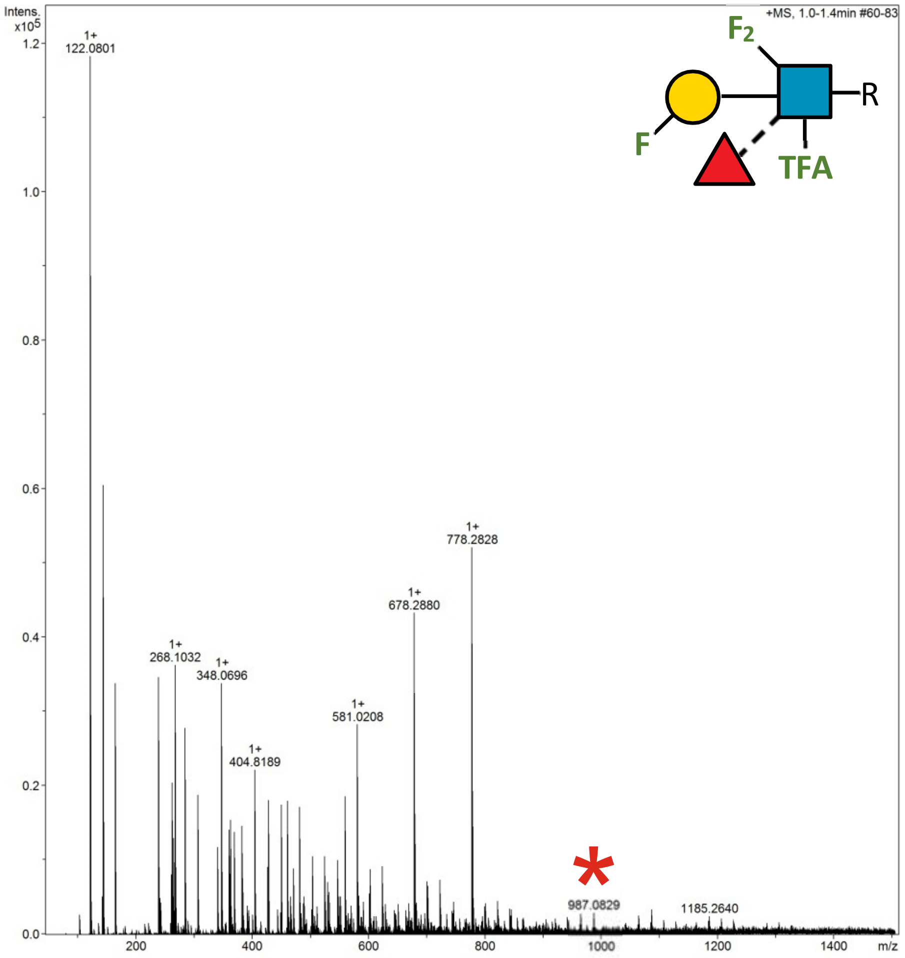

2.18.43 Supplementary Figure 60. HRMS ITag screening assay mass spectrum of synthesis of 4F-Gal β1-4 (Fuc α1-3) GlcNAc-ITag (LeX8)

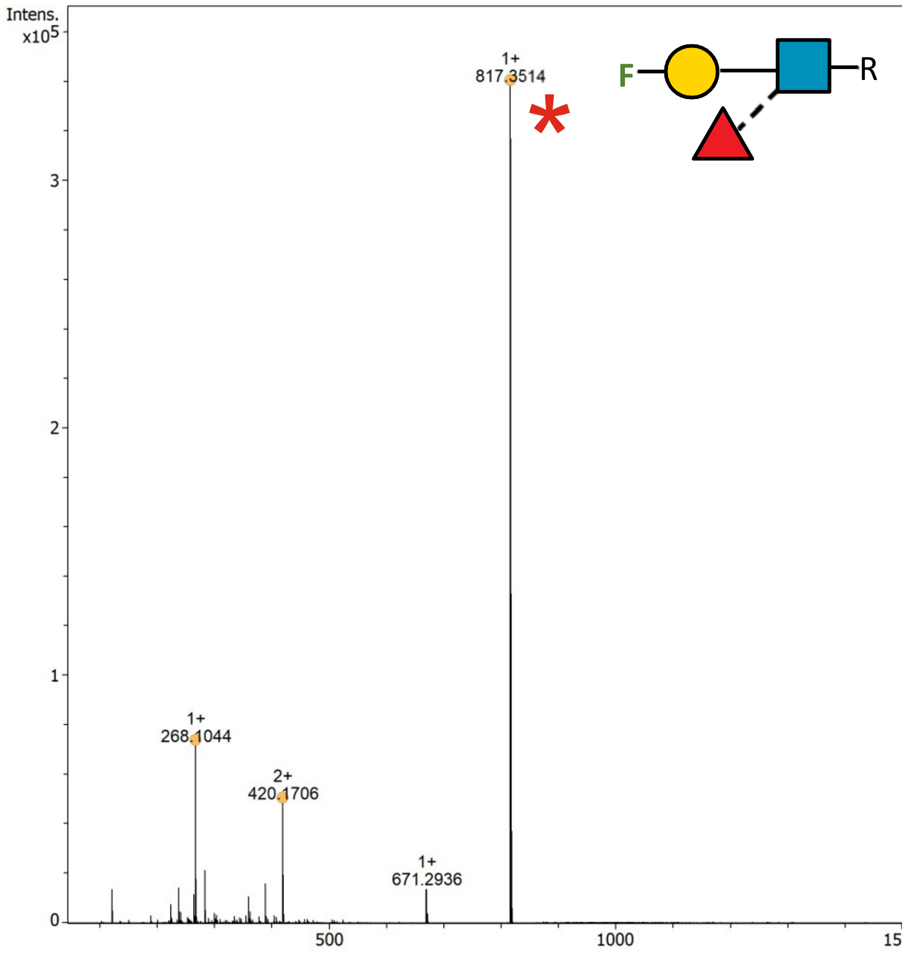

2.18.44 Supplementary Figure 61. HRMS ITag screening assay mass spectrum of synthesis of 4F-Gal β1-4 (Fuc α1-3) GlcNTFA-ITag

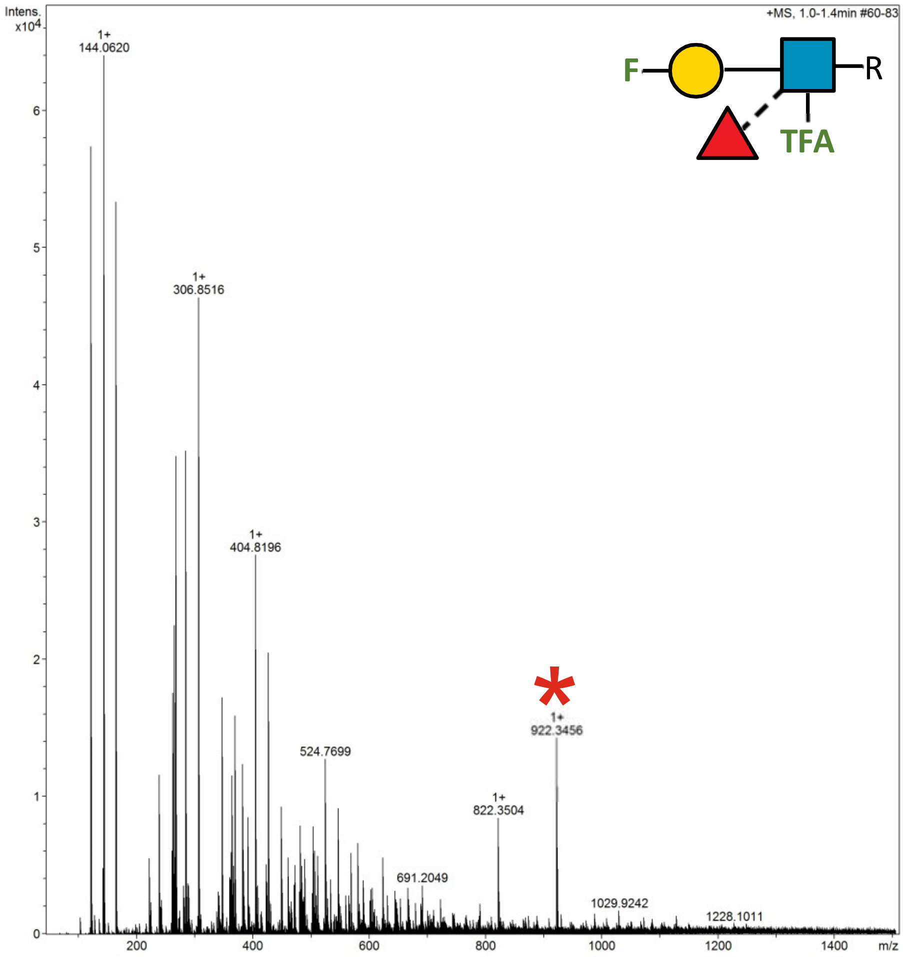

2.18.45 Supplementary Figure 62. HRMS ITag screening assay mass spectrum of synthesis of 4F-Gal β1-4 (Fuc α1-3) 6F-GlcNAc-ITag

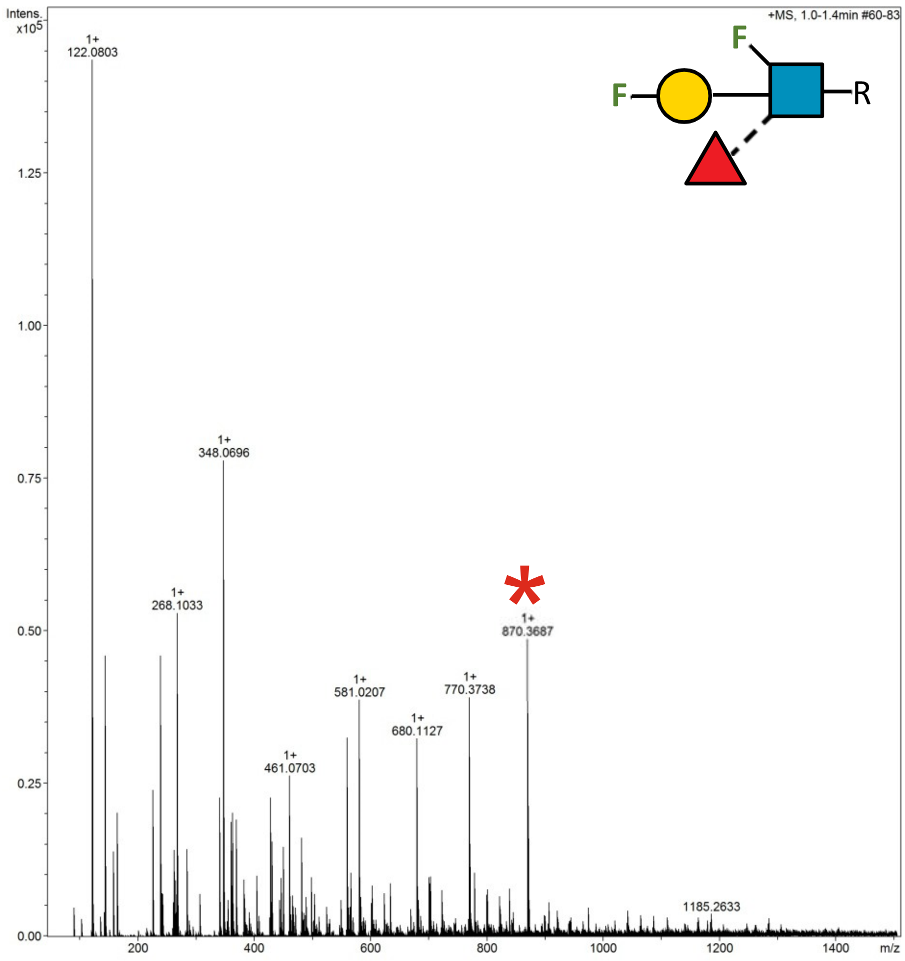

2.18.46 Supplementary Figure 63. HRMS ITag screening assay mass spectrum of synthesis of 4F-Gal β1-4 (Fuc α1-3) 6F-GlcNTFA-ITag (LeX22)

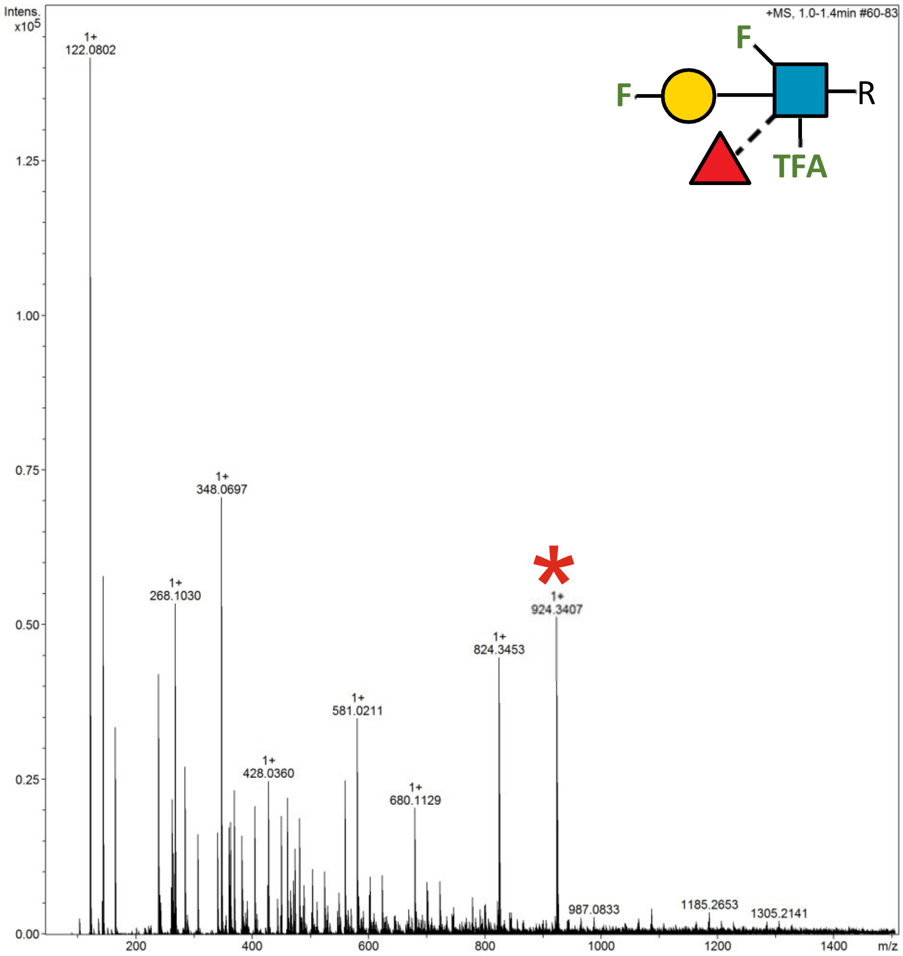

2.18.47 Supplementary Figure 64. HRMS ITag screening assay mass spectrum of synthesis of 4F-Gal β1-4 (Fuc α1-3) 6,6-diFGlcNAc-ITag

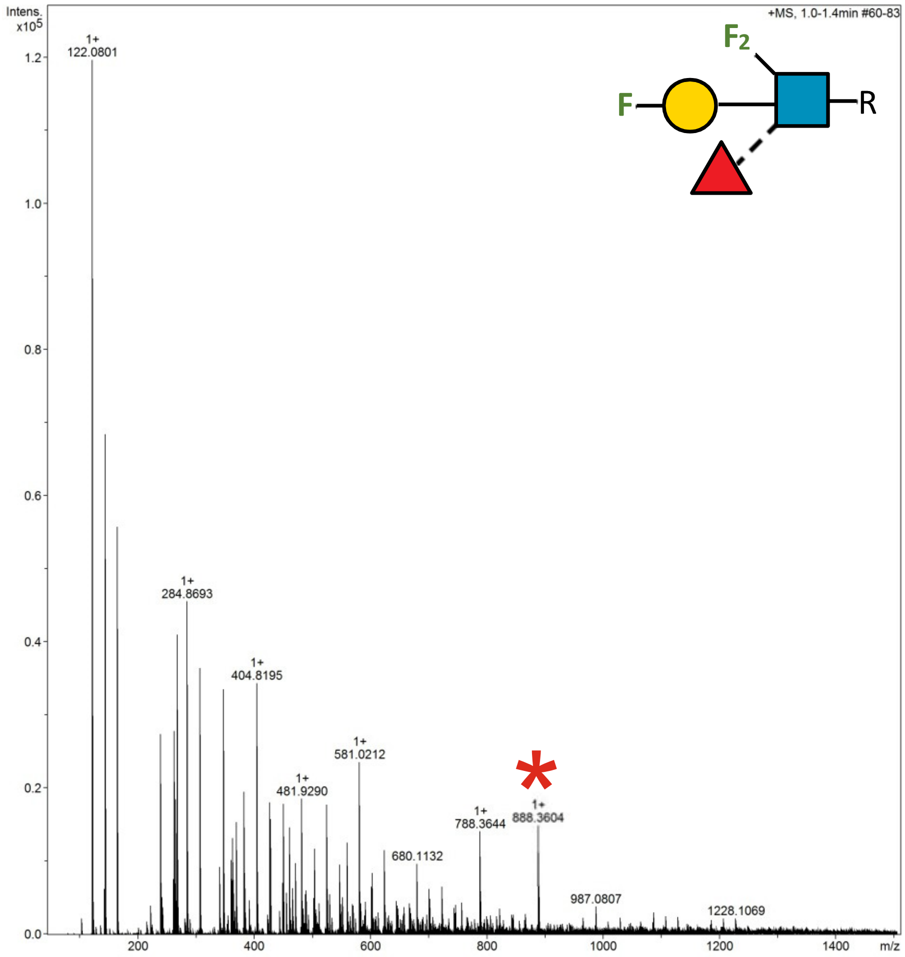

2.18.48 Supplementary Figure 65. HRMS ITag screening assay mass spectrum of synthesis of 4F-Gal β1-4 (Fuc α1-3) 6,6-diFGlcNTFA-ITag

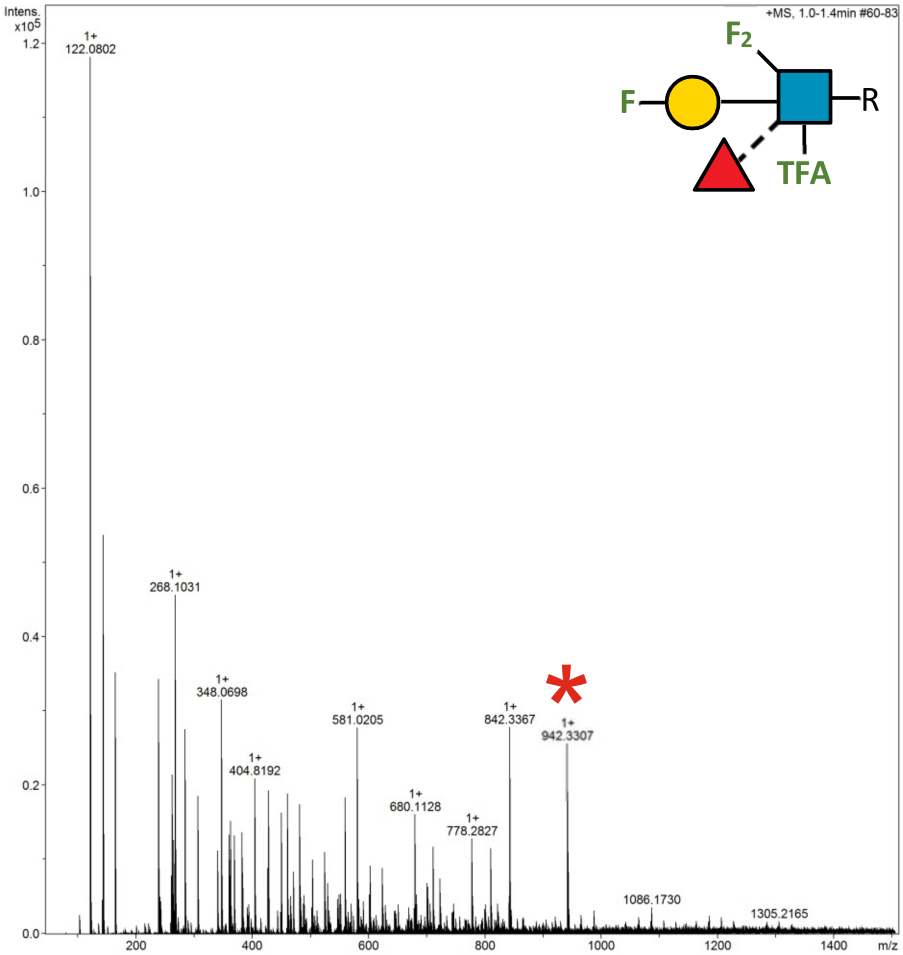

2.18.49 Supplementary Figure 66. HRMS ITag screening assay mass spectrum of synthesis of 6F-Gal β1-4 (Fuc α1-3) GlcNAc-ITag (LeX9)

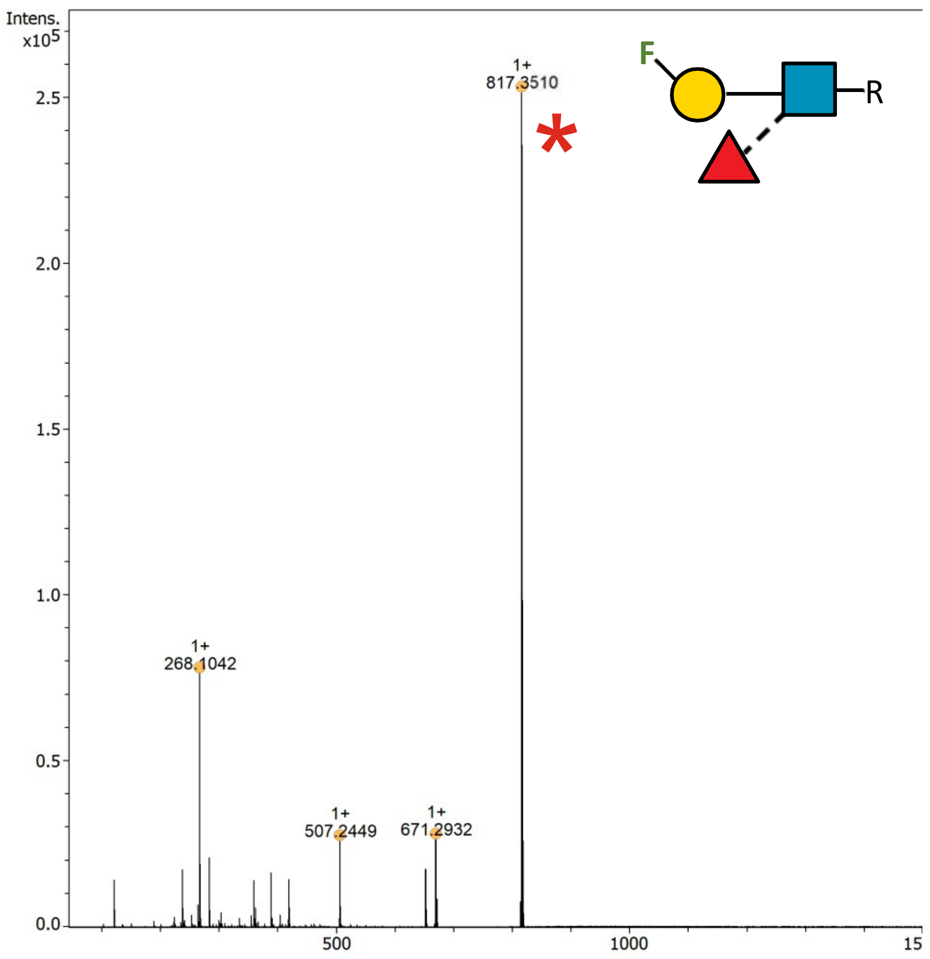

2.18.50 Supplementary Figure 67. HRMS ITag screening assay mass spectrum of synthesis of 6F-Gal β1-4 (Fuc α1-3) GlcNTFA-ITag

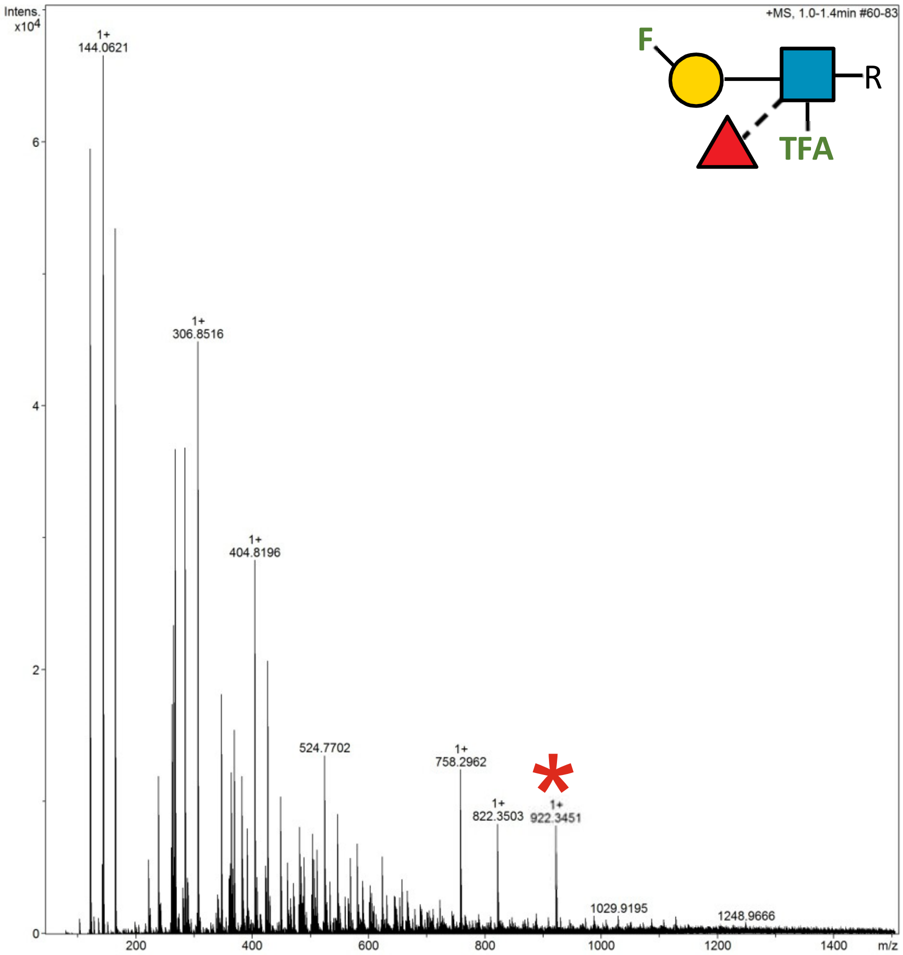

2.18.51 Supplementary Figure 68. HRMS ITag screening assay mass spectrum of synthesis of 6F-Gal β1-4 (Fuc α1-3) 6F-GlcNAc-ITag (LeX17)

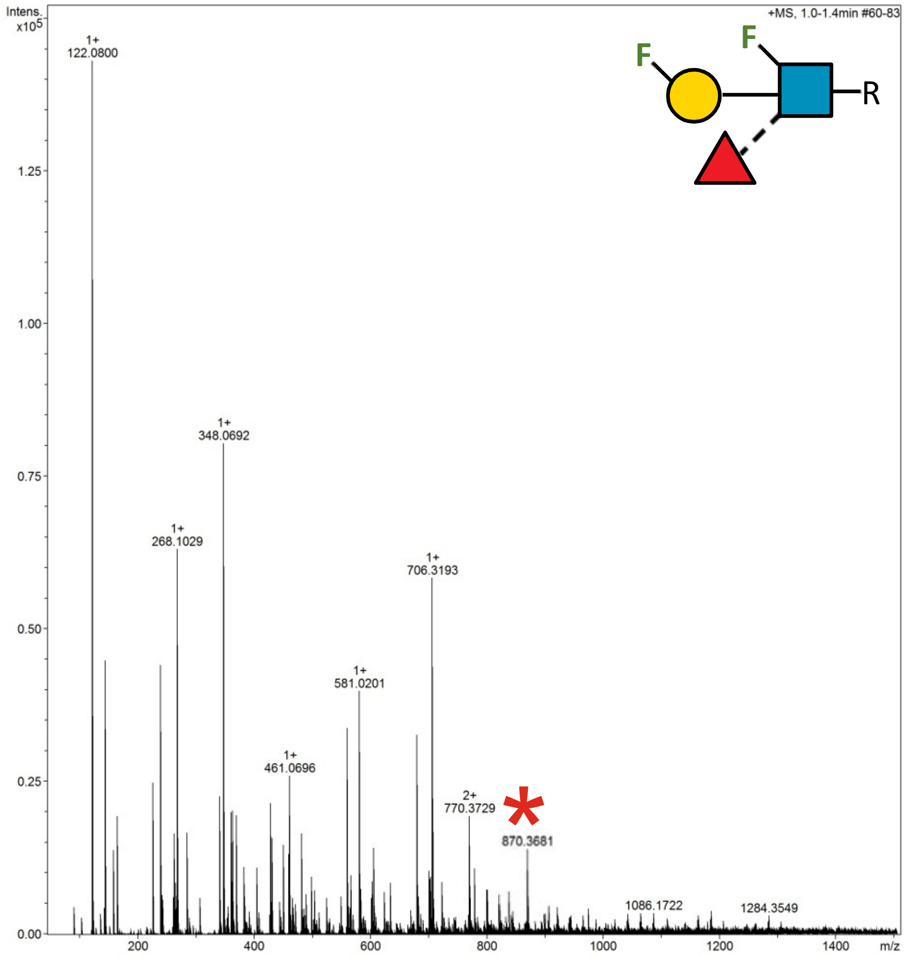

2.18.52 Supplementary Figure 69. HRMS ITag screening assay mass spectrum of synthesis of 6F-Gal β1-4 (Fuc α1-3) 6F-GlcNTFA-ITag

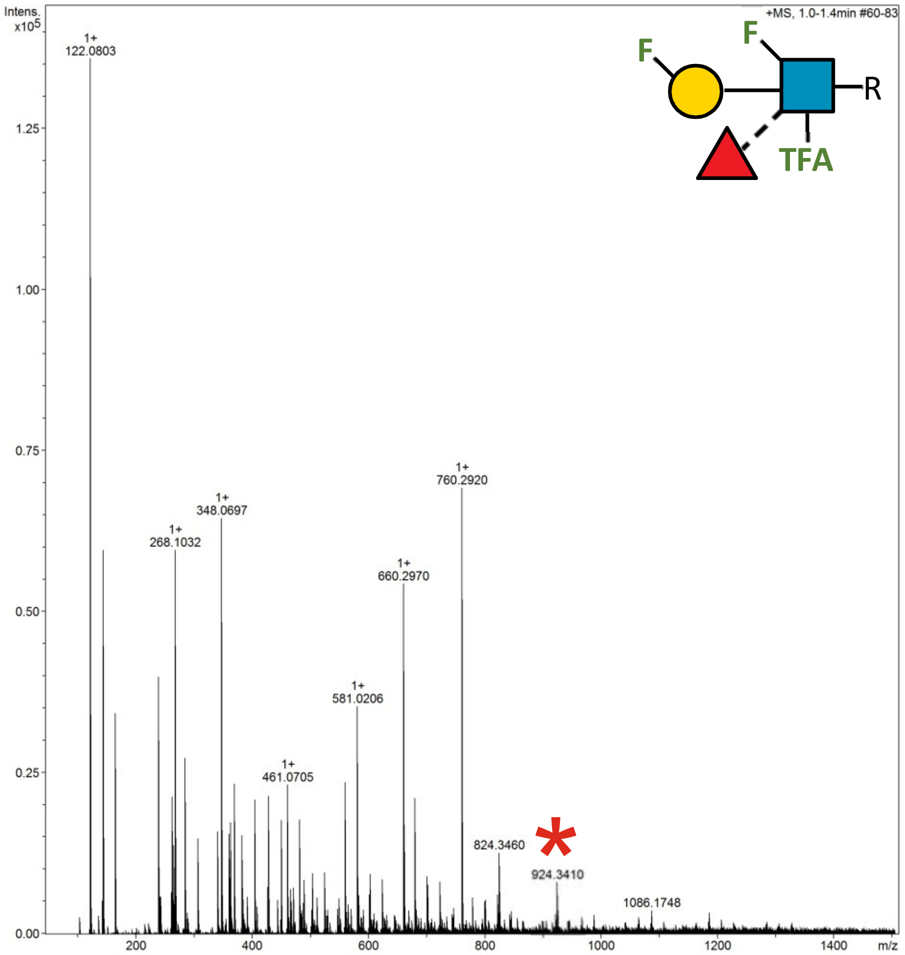

2.18.53 Supplementary Figure 70. HRMS ITag screening assay mass spectrum of synthesis of 6F-Gal β1-4 (Fuc α1-3) 6,6-diFGlcNAc-ITag

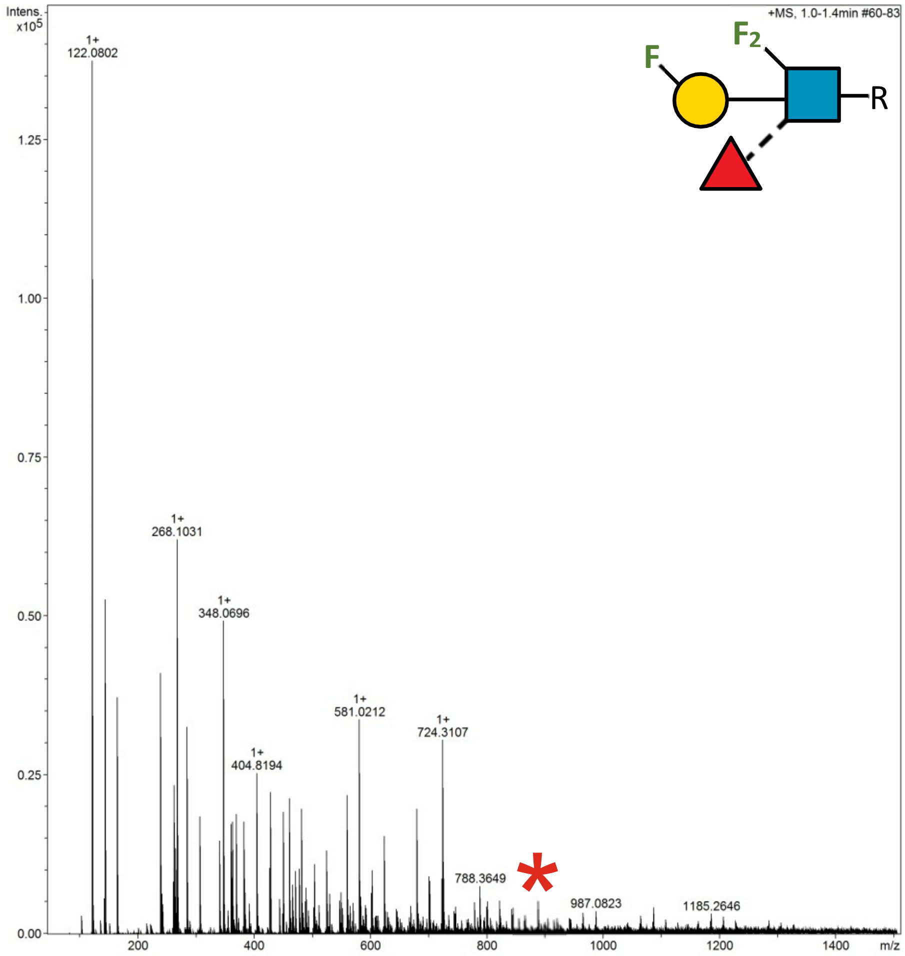

2.18.54 Supplementary Figure 71. HRMS ITag screening assay mass spectrum of synthesis of 6F-Gal β1-4 (Fuc α1-3) 6,6-diFGlcNTFA-ITag

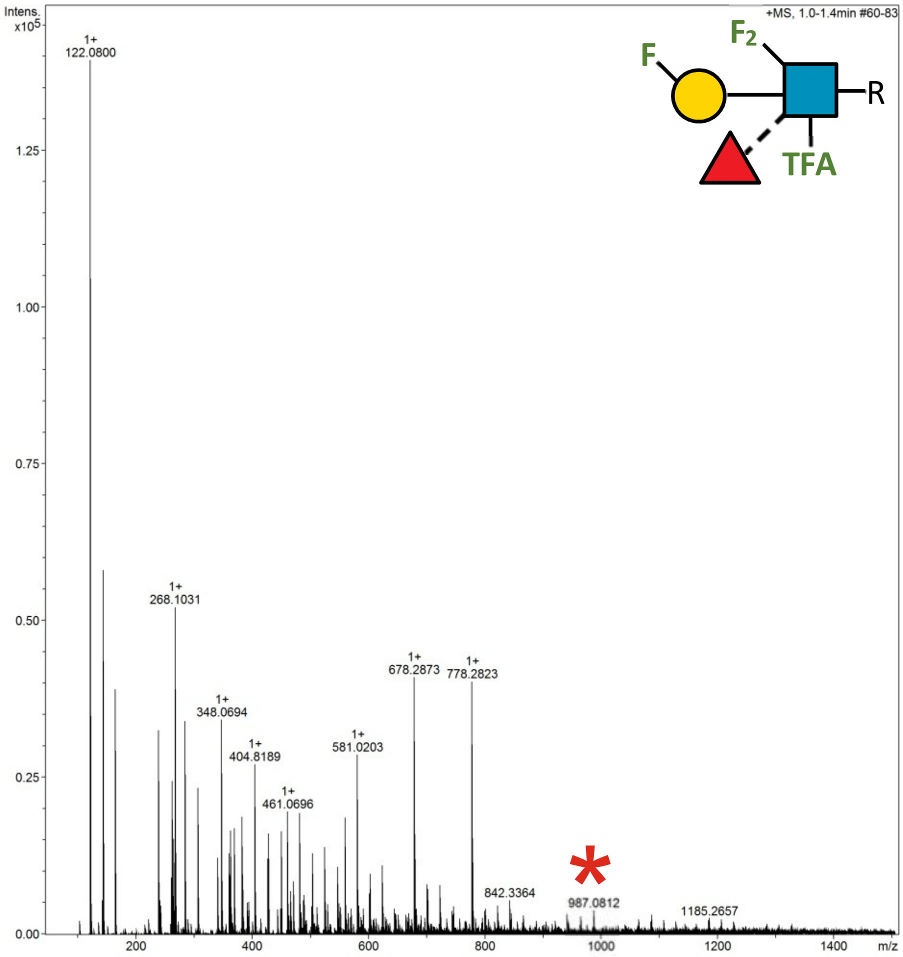

2.18.55 Supplementary Figure 72. HRMS ITag screening assay mass spectrum of synthesis of 6d-Gal β1-4 (Fuc α1-3) GlcNAc-ITag (LeX10)

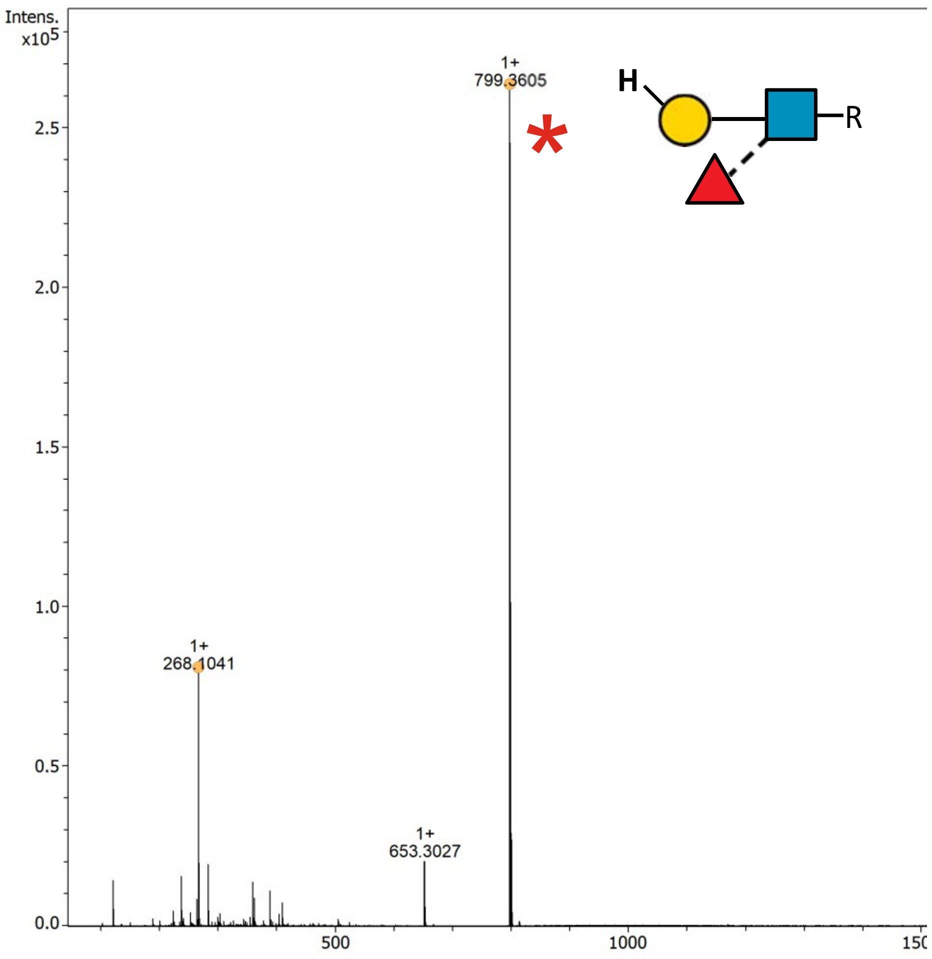

2.18.56 Supplementary Figure 73. HRMS ITag screening assay mass spectrum of synthesis of 6d-Gal β1-4 (Fuc α1-3) GlcNTFA-ITag

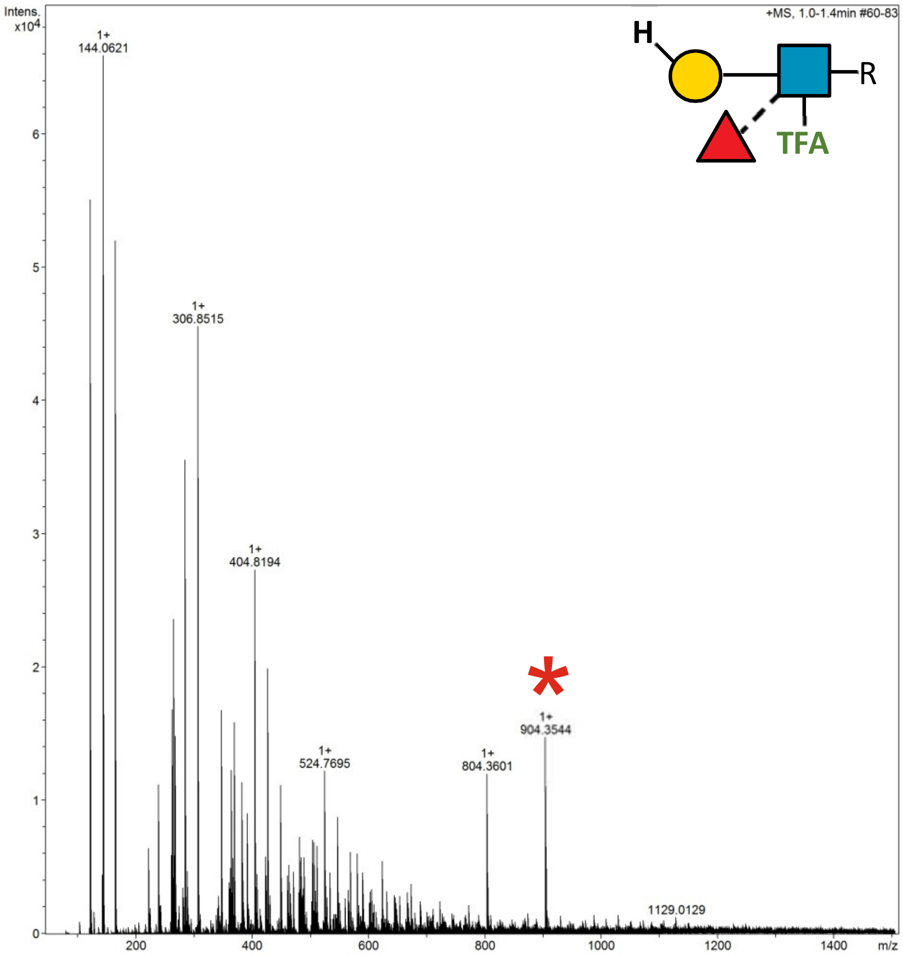

2.18.57 Supplementary Figure 74. HRMS ITag screening assay mass spectrum of synthesis of 6d-Gal β1-4 (Fuc α1-3) 6F-GlcNAc-ITag

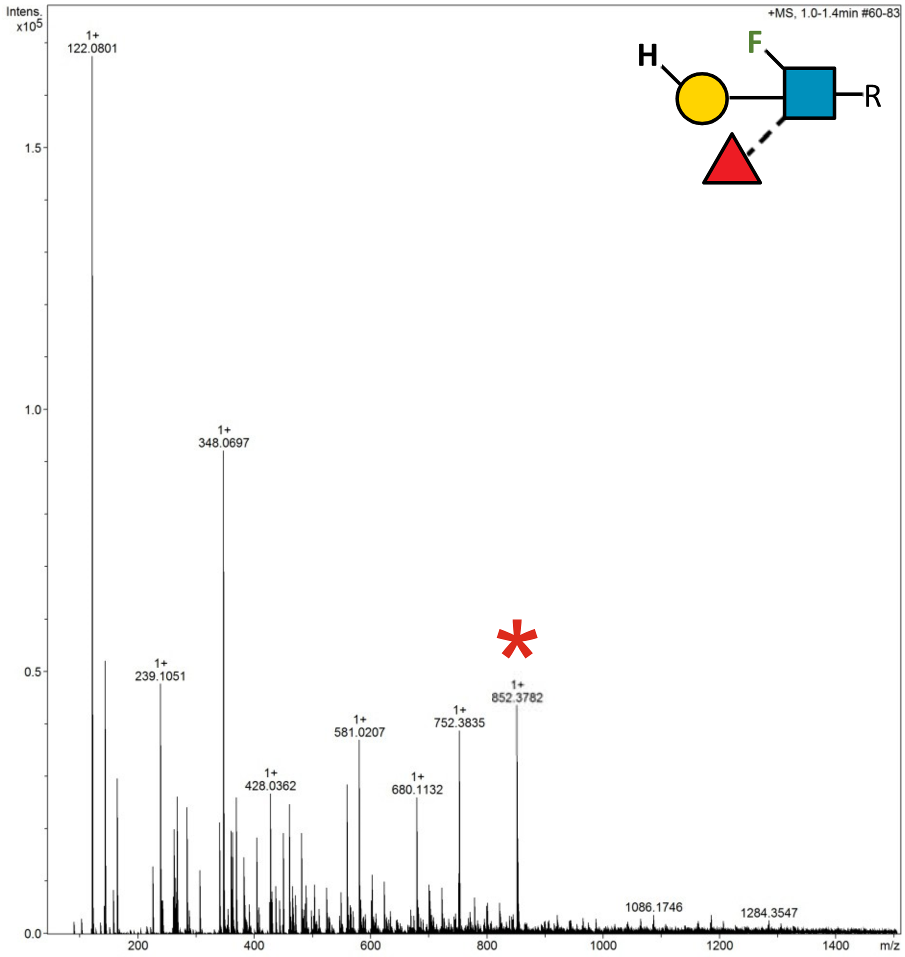

2.18.58 Supplementary Figure 75. HRMS ITag screening assay mass spectrum of synthesis of 6d-Gal β1-4 (Fuc α1-3) 6F-GlcNTFA-ITag

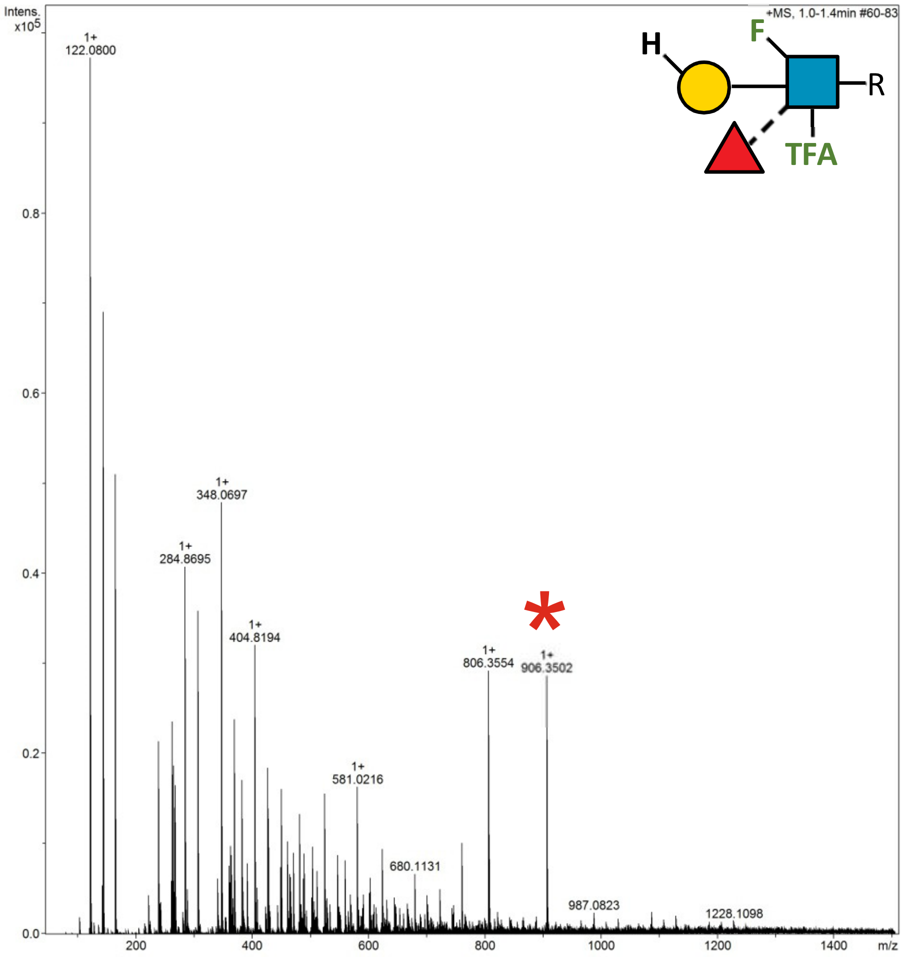

2.18.59 Supplementary Figure 76. HRMS ITag screening assay mass spectrum of synthesis of 6d-Gal β1-4 (Fuc α1-3) 6,6-diFGlcNAc-ITag

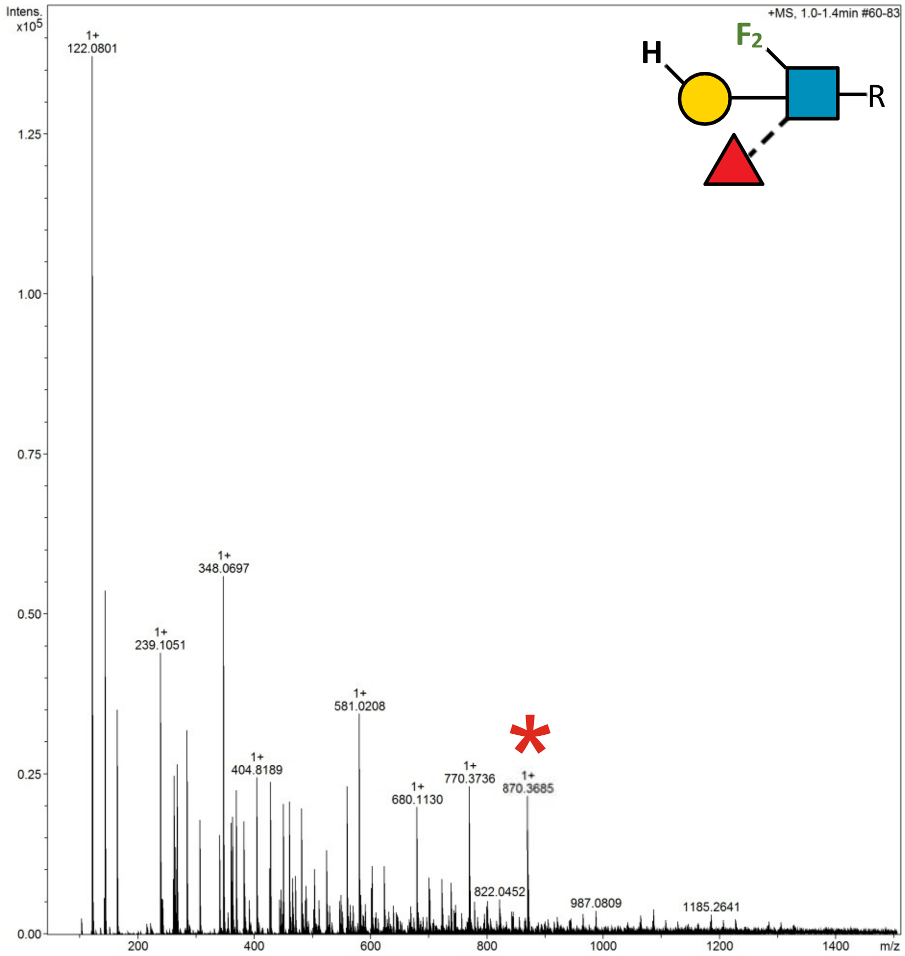

2.18.60 Supplementary Figure 77. HRMS ITag screening assay mass spectrum of synthesis of 6d-Gal β1-4 (Fuc α1-3) 6,6-diFGlcNTFA-ITag (LeX23)

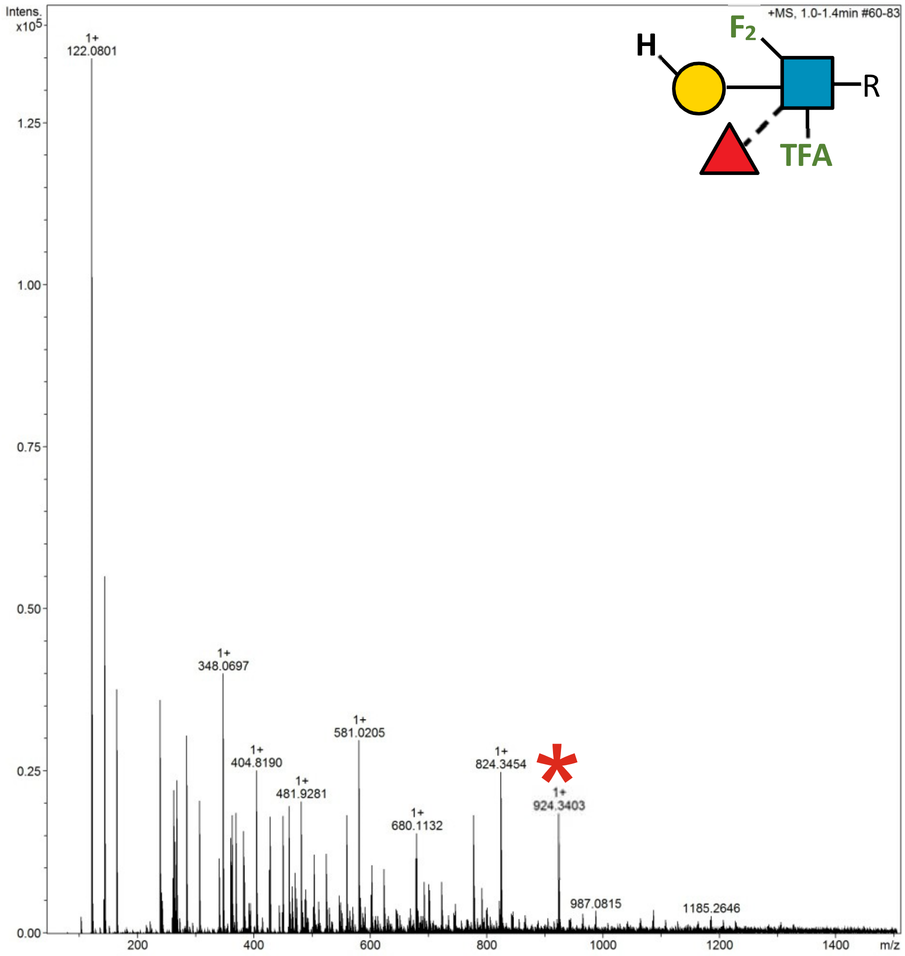

2.18.61 Supplementary Figure 78. HRMS ITag screening assay mass spectrum of synthesis of Gal β1-4 (3F-Fuc α1-3) GlcNAc-ITag (LeX11)

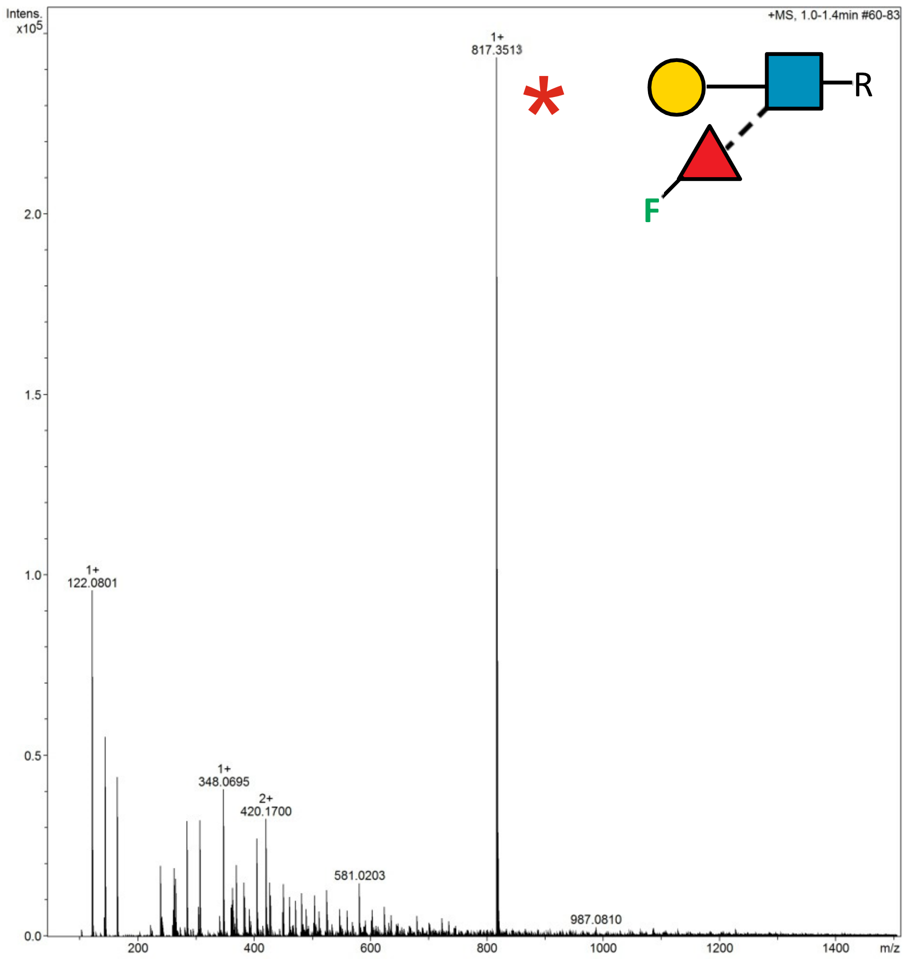

2.18.62 Supplementary Figure 79. HRMS ITag screening assay mass spectrum of synthesis of Gal β1-4 (3F-Fuc α1-3) GlcNTFA-ITag

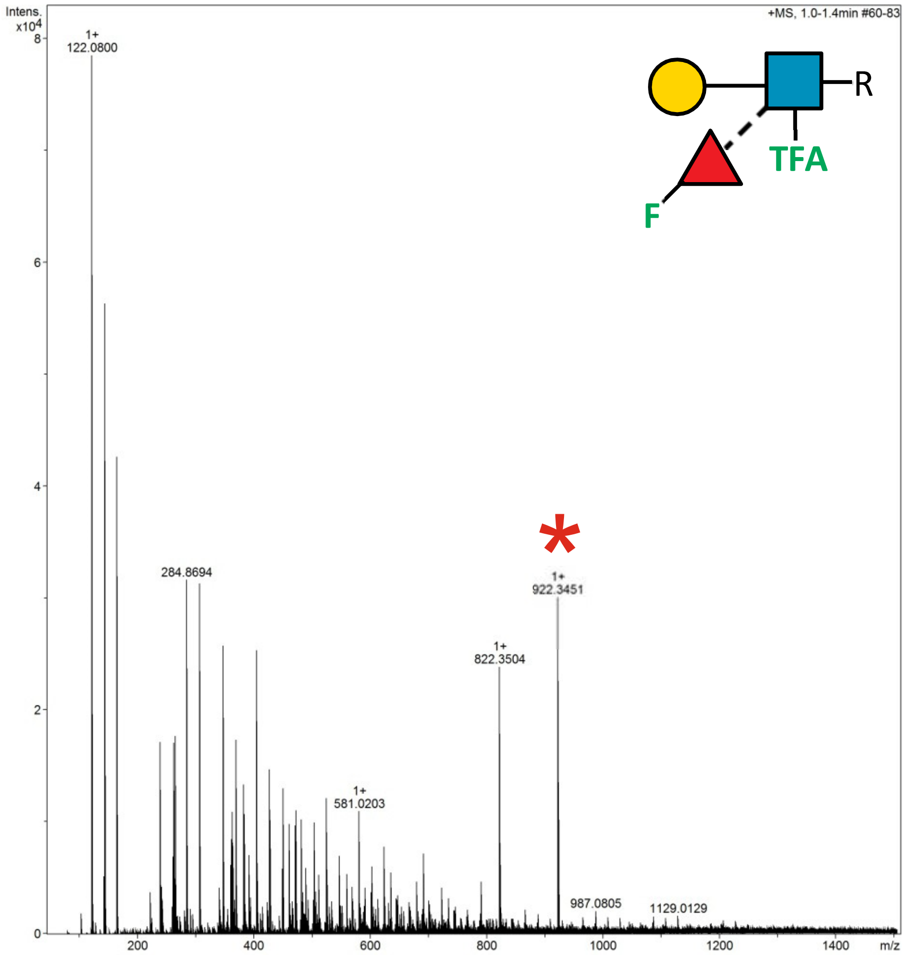

2.18.63 Supplementary Figure 80. HRMS ITag screening assay mass spectrum of synthesis of Gal β1-4 (3F-Fuc α1-3) 6F-GlcNAc-ITag

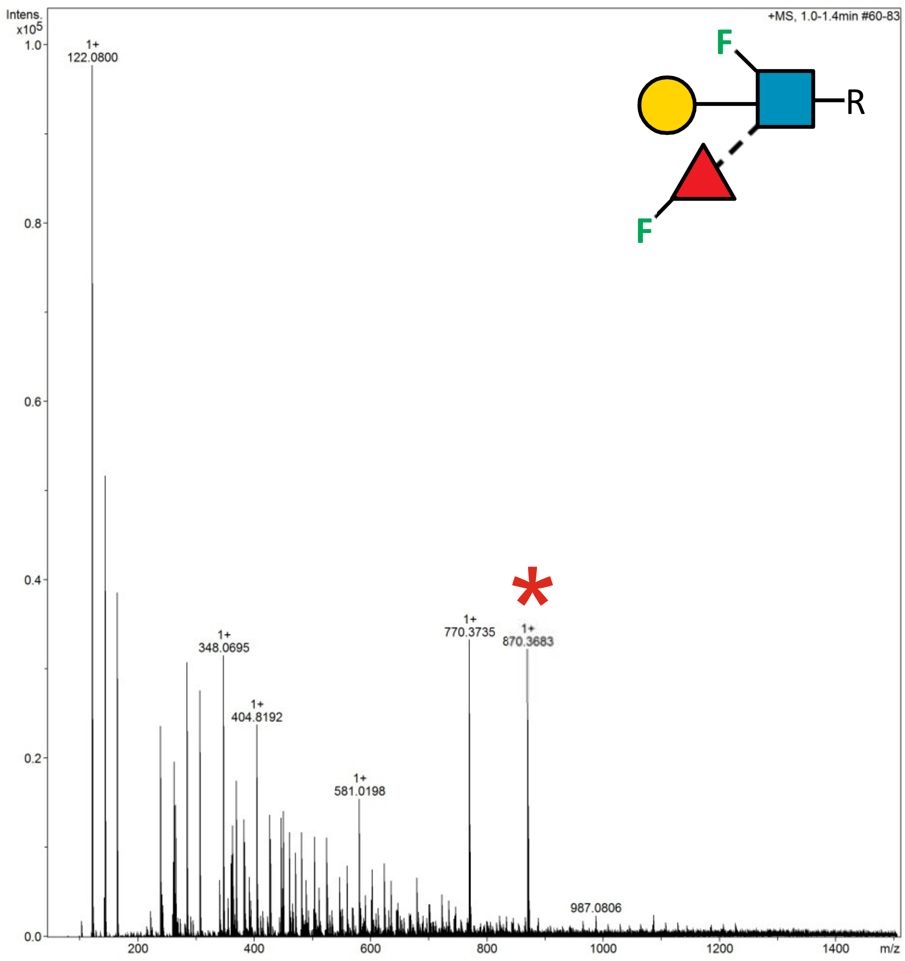

2.18.64 Supplementary Figure 81. HRMS ITag screening assay mass spectrum of synthesis of Gal β1-4 (3F-Fuc α1-3) 6F-GlcNTFA-ITag (LeX18)

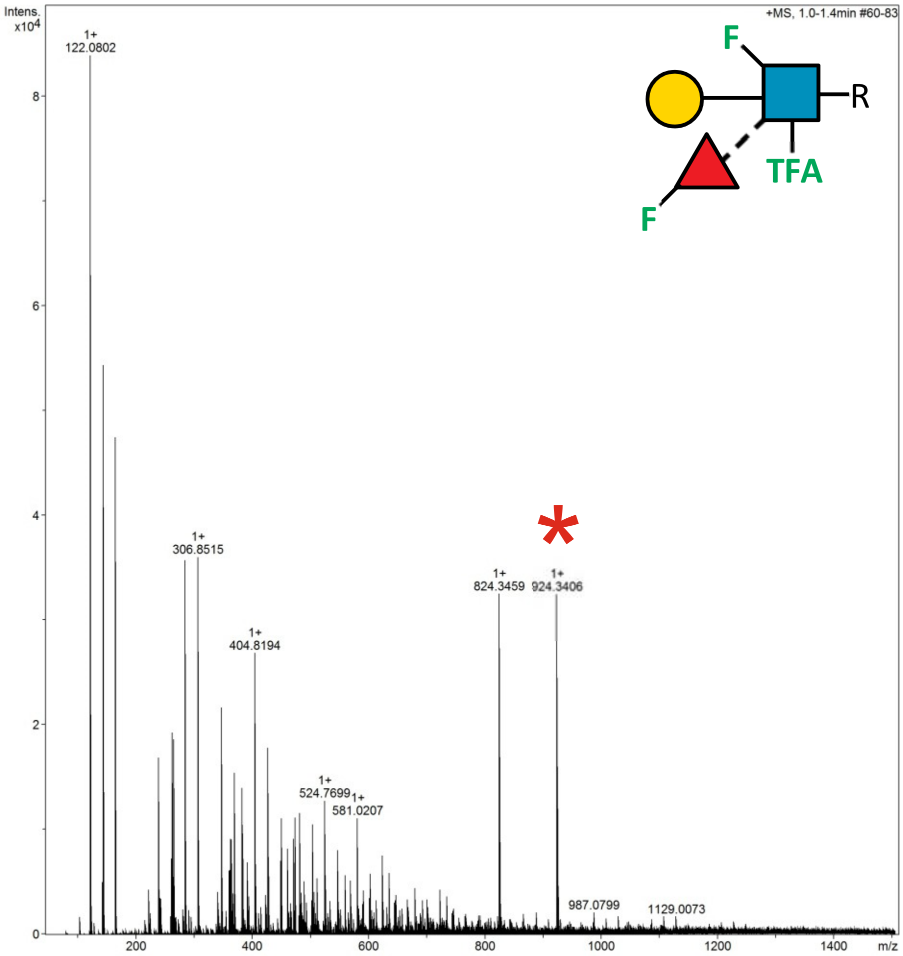

2.18.65 Supplementary Figure 82. HRMS ITag screening assay mass spectrum of synthesis of Gal β1-4 (3F-Fuc α1-3) 6,6-diFGlcNAc-ITag

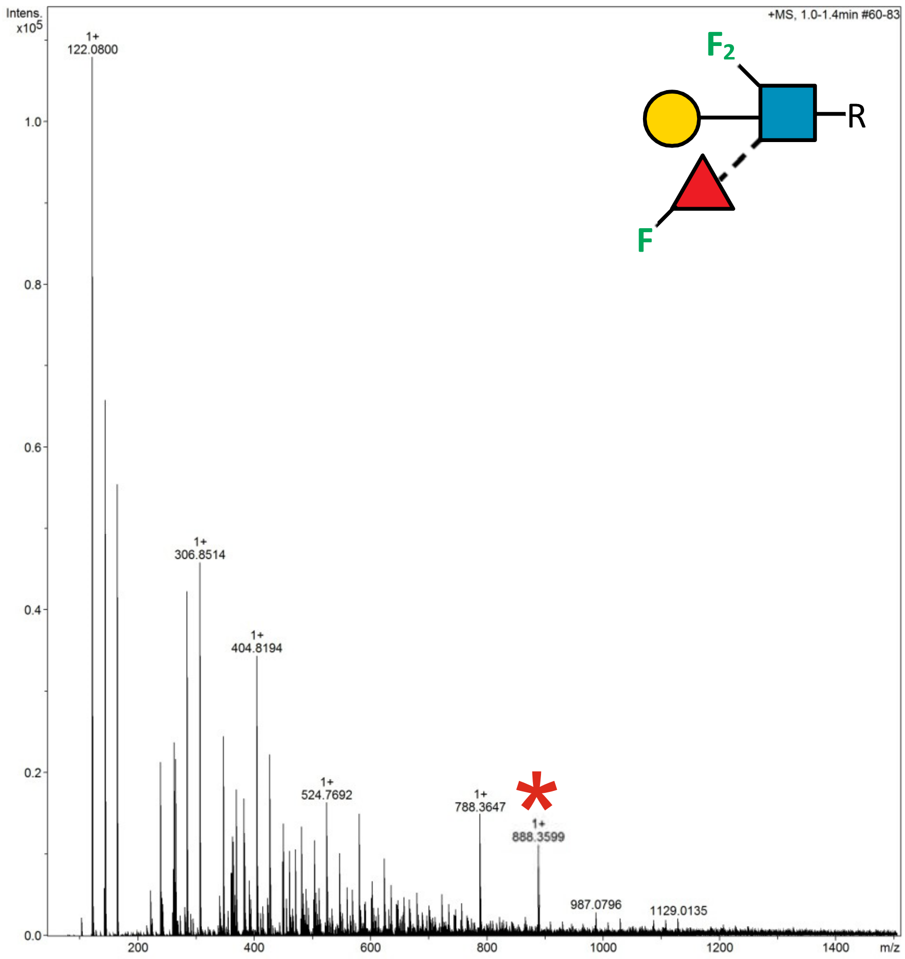

2.18.66 Supplementary Figure 83. HRMS ITag screening assay mass spectrum of synthesis of Gal β1-4 (3F-Fuc α1-3) 6,6-diFGlcNTFA-ITag (LeX19)

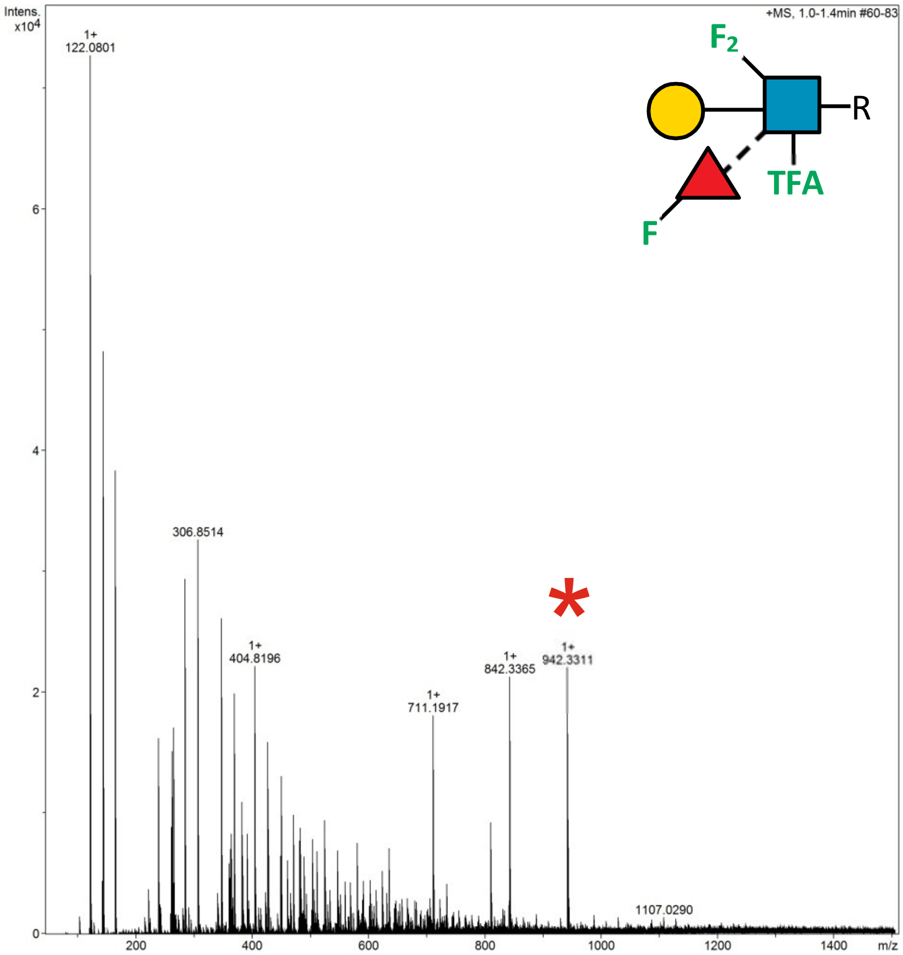

2.18.67 Supplementary Figure 84. HRMS ITag screening assay mass spectrum of synthesis of 3F-Gal β1-4 (3F-Fuc α1-3) GlcNAc-ITag

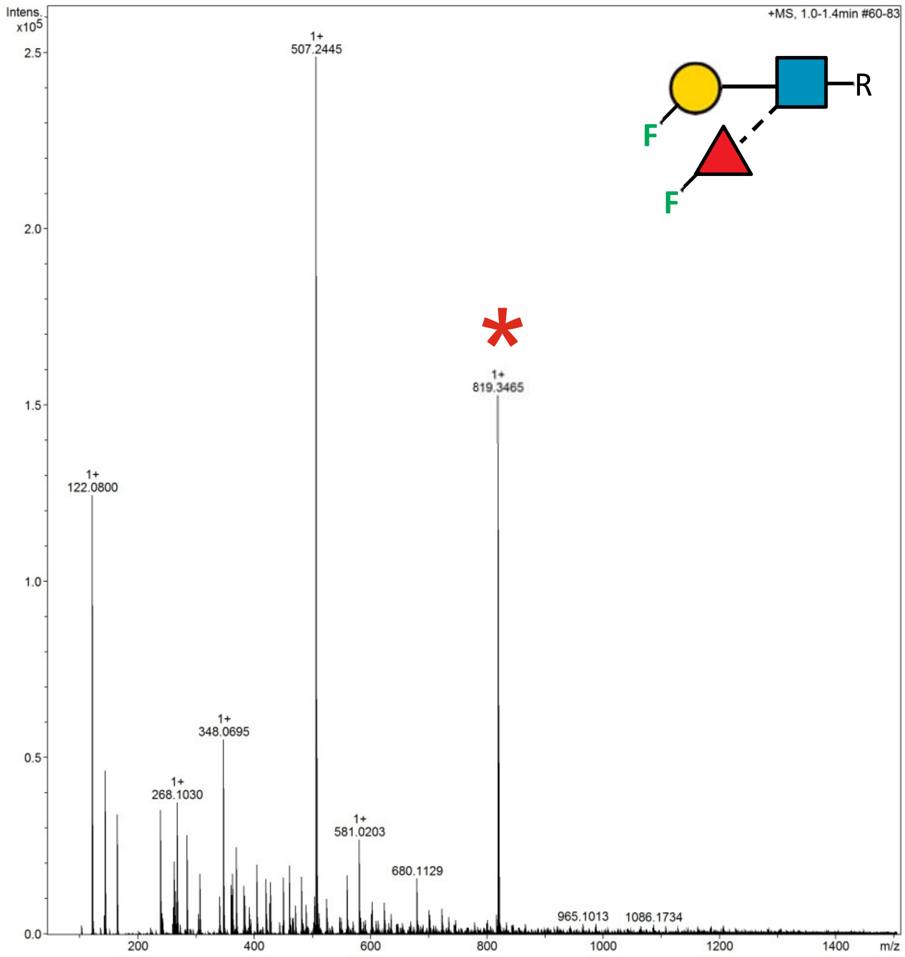

2.18.68 Supplementary Figure 85. HRMS ITag screening assay mass spectrum of synthesis of 3F-Gal β1-4 (3F-Fuc α1-3) GlcNTFA-ITag

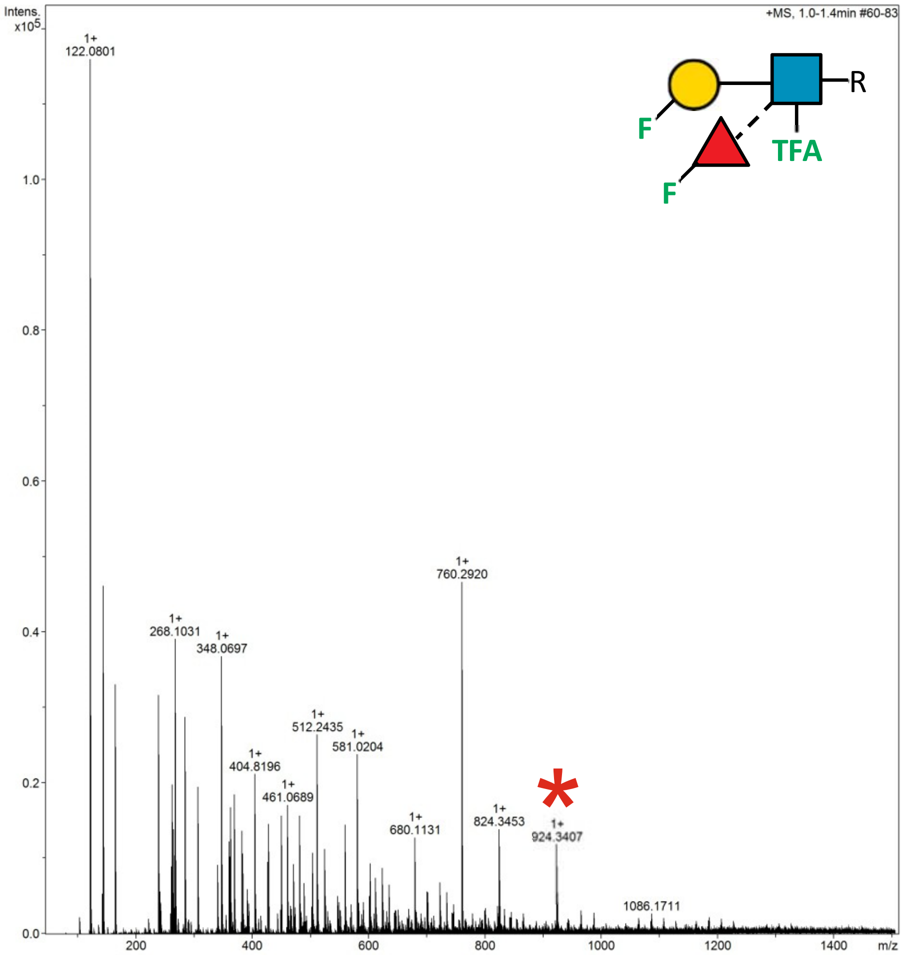

2.18.69 Supplementary Figure 86. HRMS ITag screening assay mass spectrum of synthesis of 3F-Gal β1-4 (3F-Fuc α1-3) 6F-GlcNAc-ITag

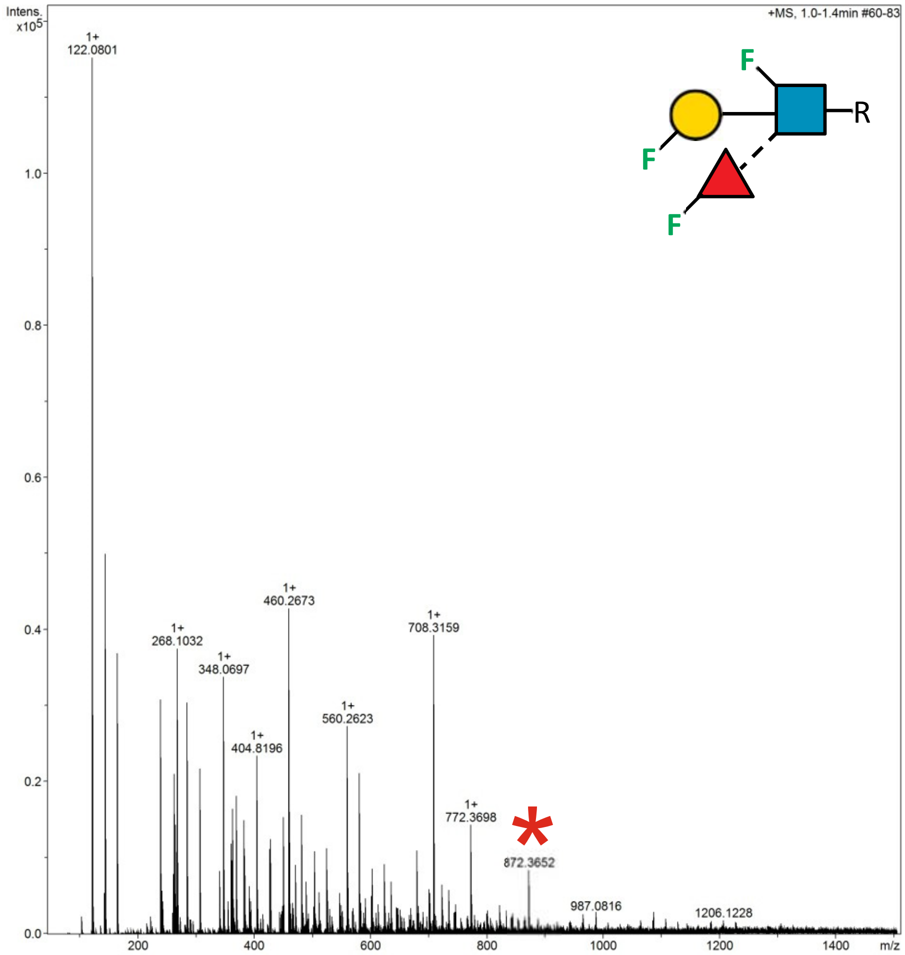

2.18.70 Supplementary Figure 87. HRMS ITag screening assay mass spectrum of synthesis of 3F-Gal β1-4 (3F-Fuc α1-3) 6F-GlcNTFA-ITag

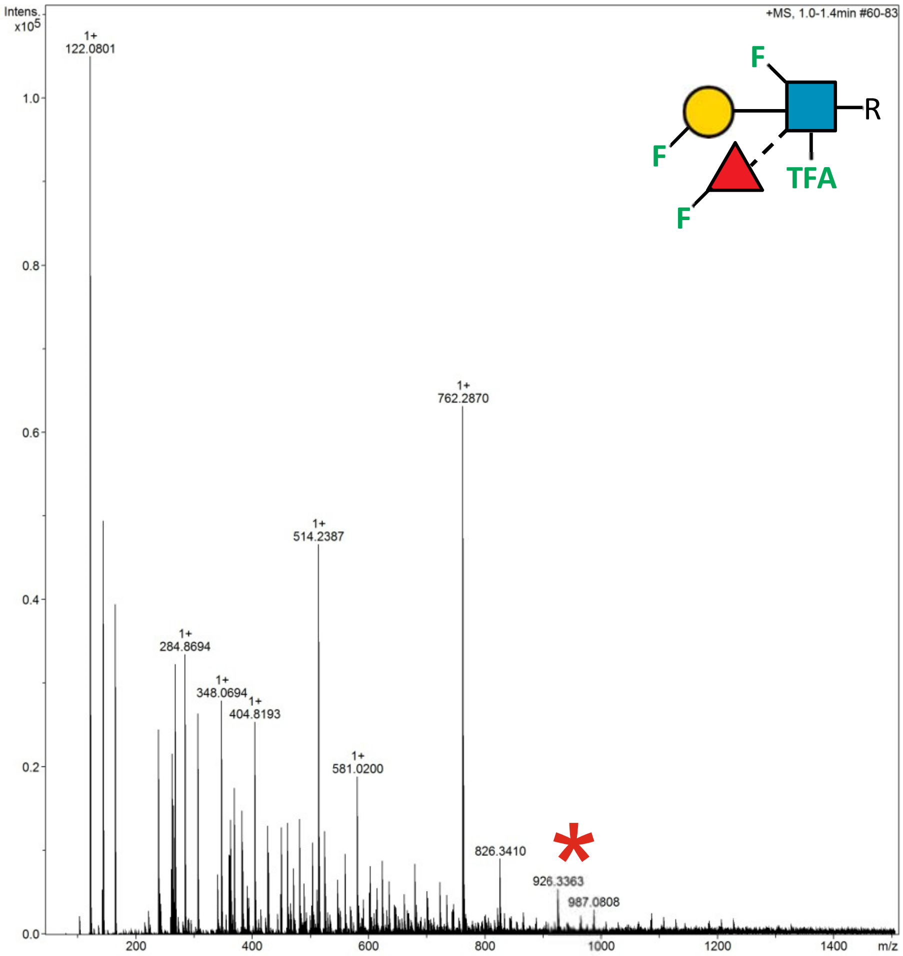

2.18.71 Supplementary Figure 88. HRMS ITag screening assay mass spectrum of synthesis of 3F-Gal β1-4 (3F-Fuc α1-3) 6,6-diFGlcNAc-ITag

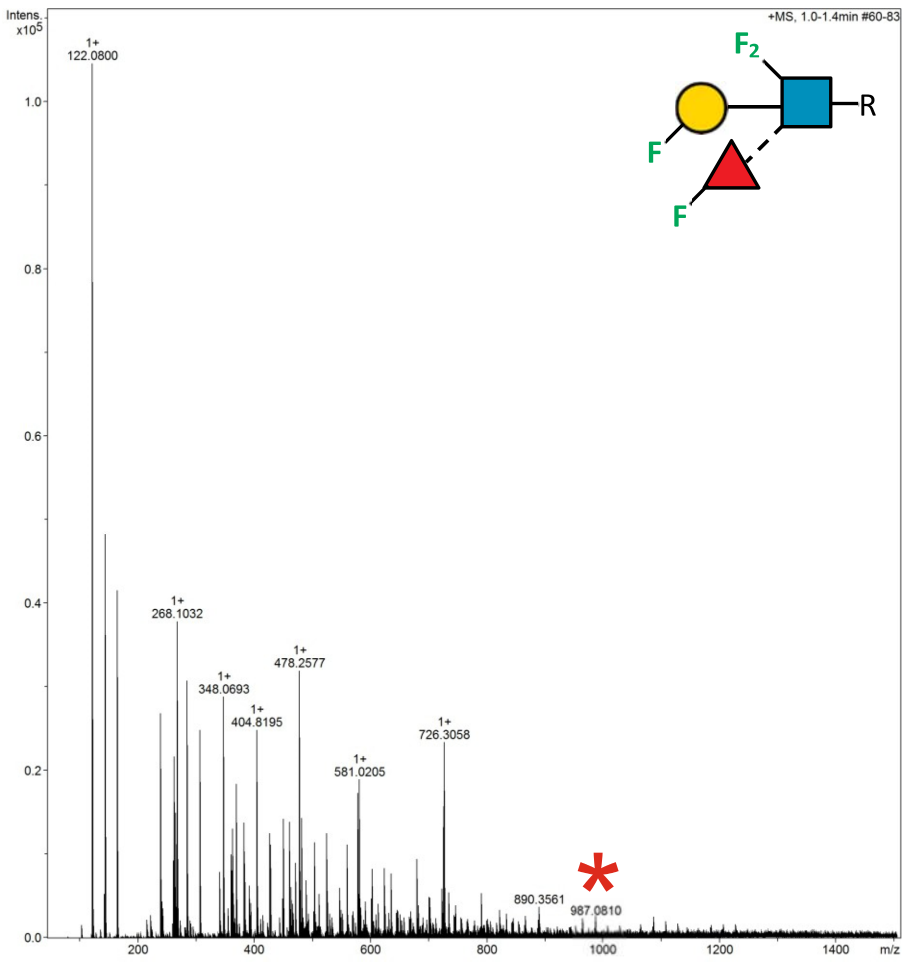

2.18.72 Supplementary Figure 89. HRMS ITag screening assay mass spectrum of synthesis of 3F-Gal β1-4 (3F-Fuc α1-3) 6,6-diFGlcNTFA-ITag

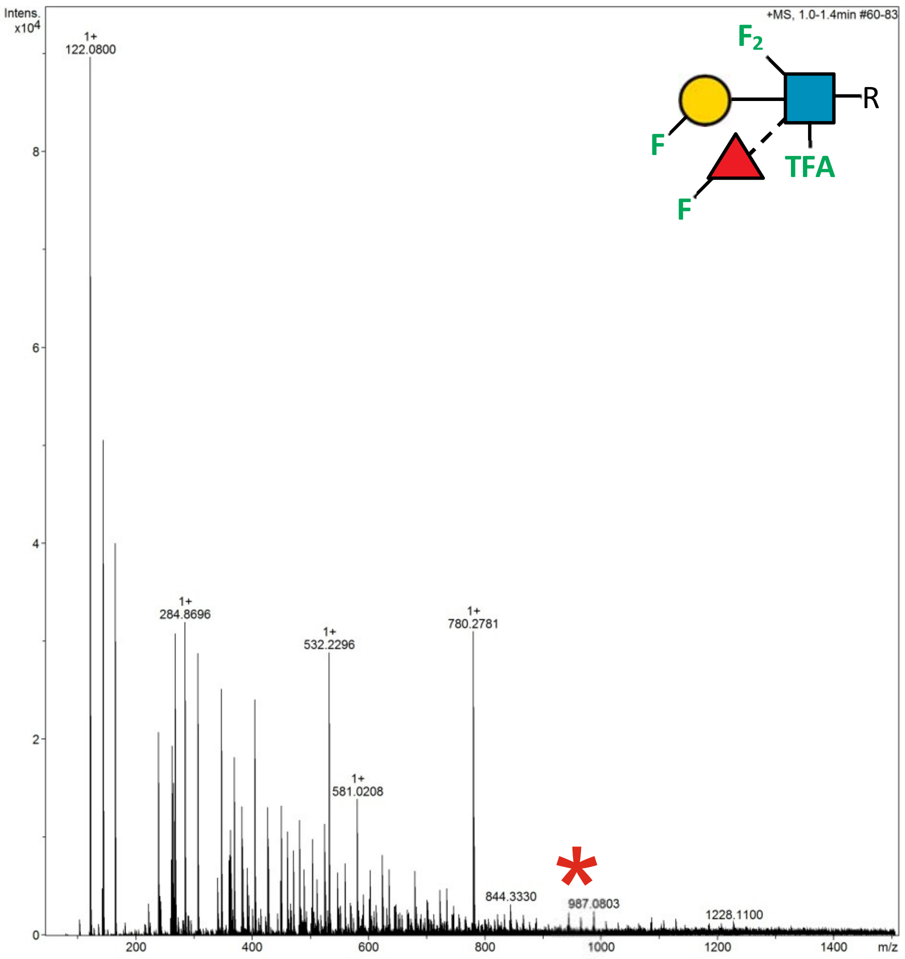

2.18.73 Supplementary Figure 90. HRMS ITag screening assay mass spectrum of synthesis of 4F-Gal β1-4 (3F-Fuc α1-3) GlcNAc-ITag

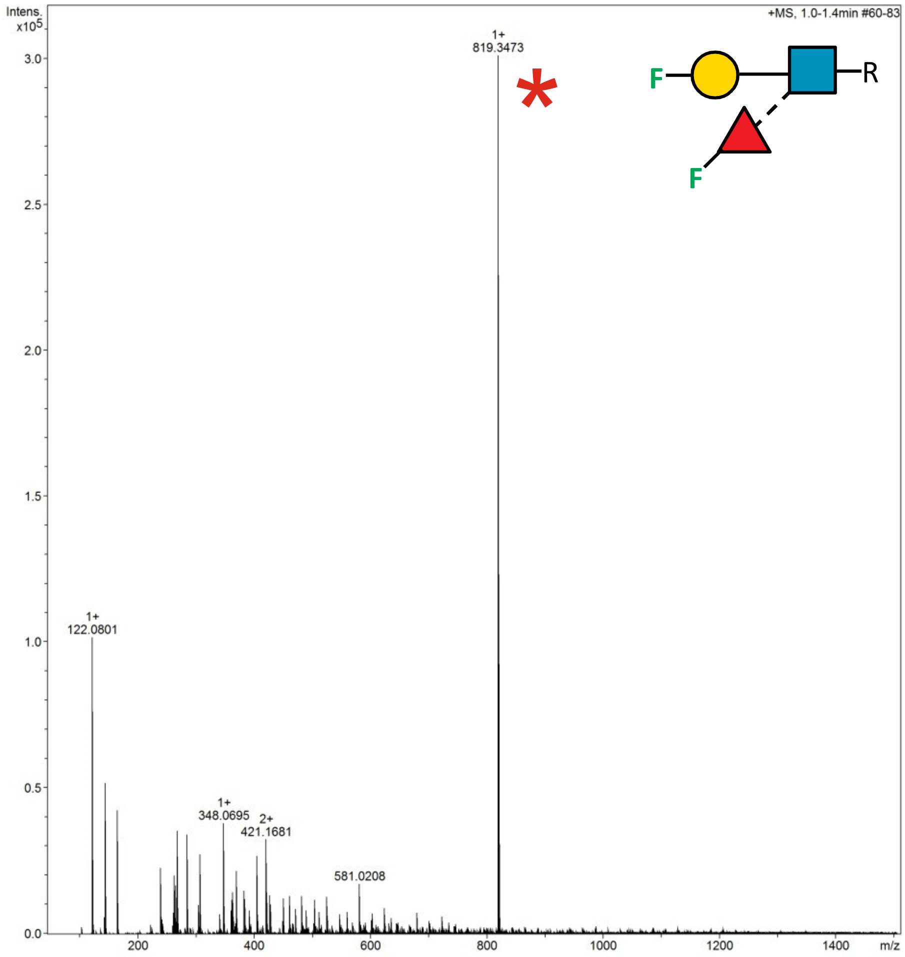

2.18.74 Supplementary Figure 91. HRMS ITag screening assay mass spectrum of synthesis of 4F-Gal β1-4 (3F-Fuc α1-3) GlcNTFA-ITag

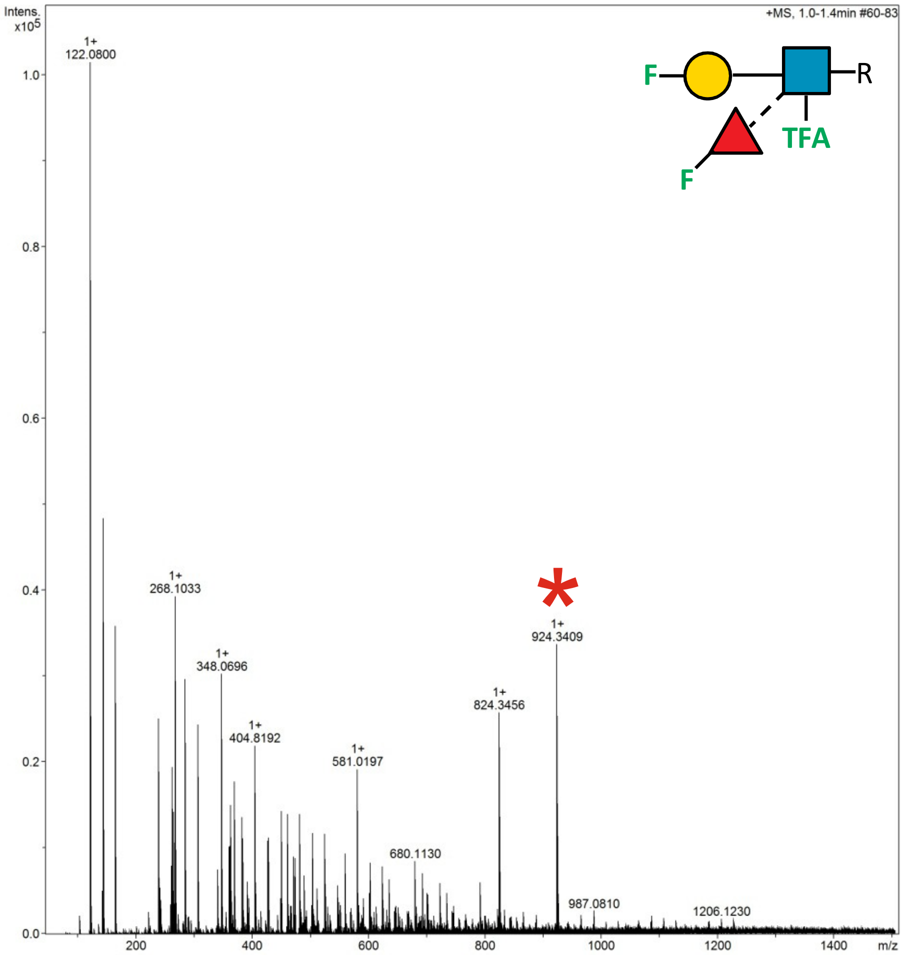

2.18.75 Supplementary Figure 92. HRMS ITag screening assay mass spectrum of synthesis of 4F-Gal β1-4 (3F-Fuc α1-3) 6F-GlcNAc-ITag

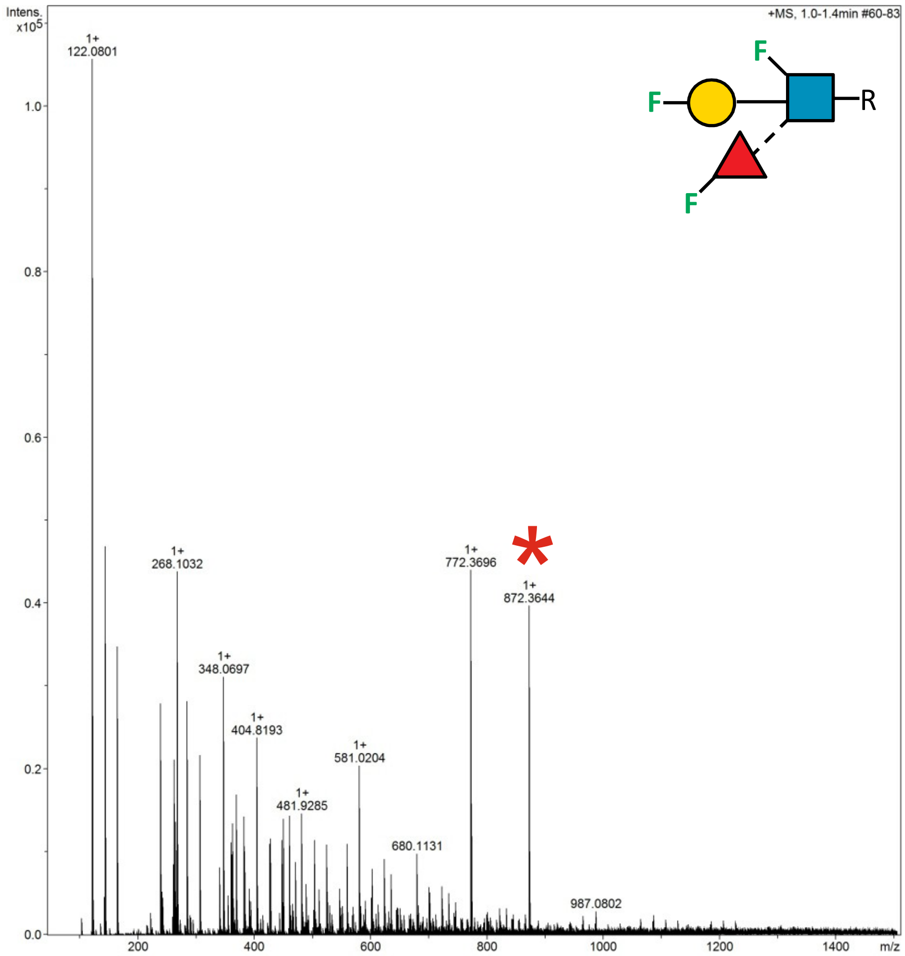

2.18.76 Supplementary Figure 93. HRMS ITag screening assay mass spectrum of synthesis of 4F-Gal β1-4 (3F-Fuc α1-3) 6F-GlcNTFA-ITag

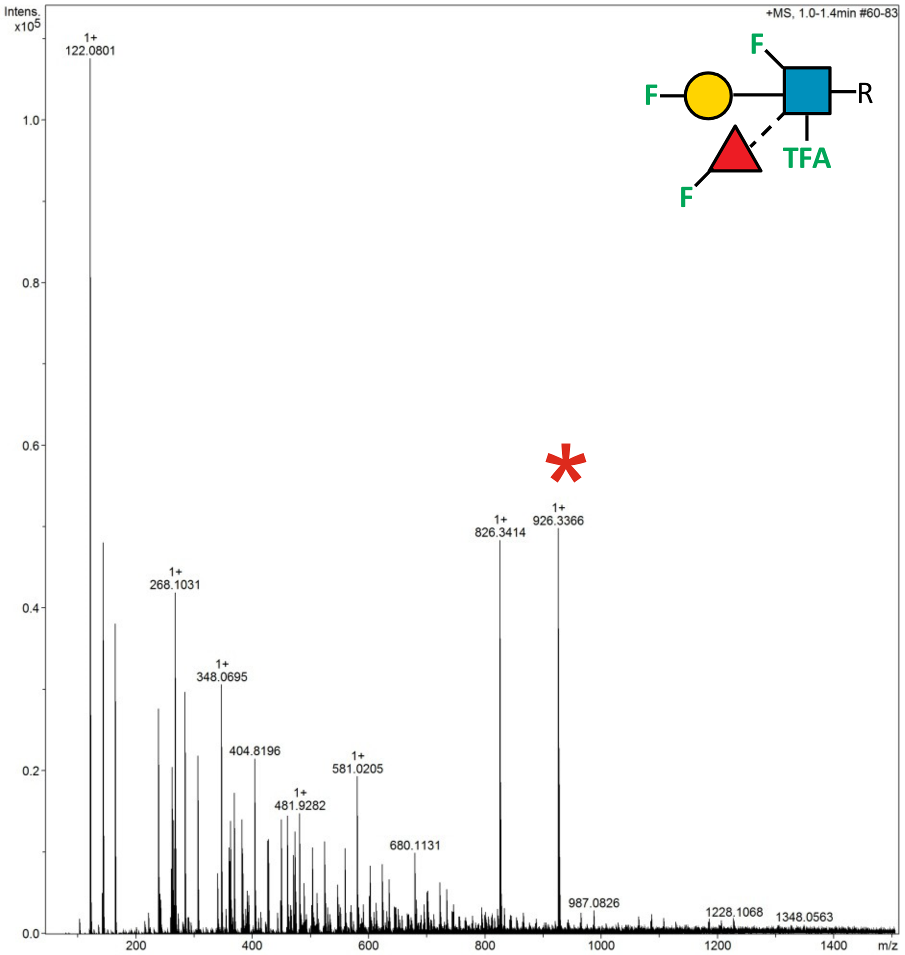

2.18.77 Supplementary Figure 94. HRMS ITag screening assay mass spectrum of synthesis of 4F-Gal β1-4 (3F-Fuc α1-3) 6,6-diFGlcNAc-ITag

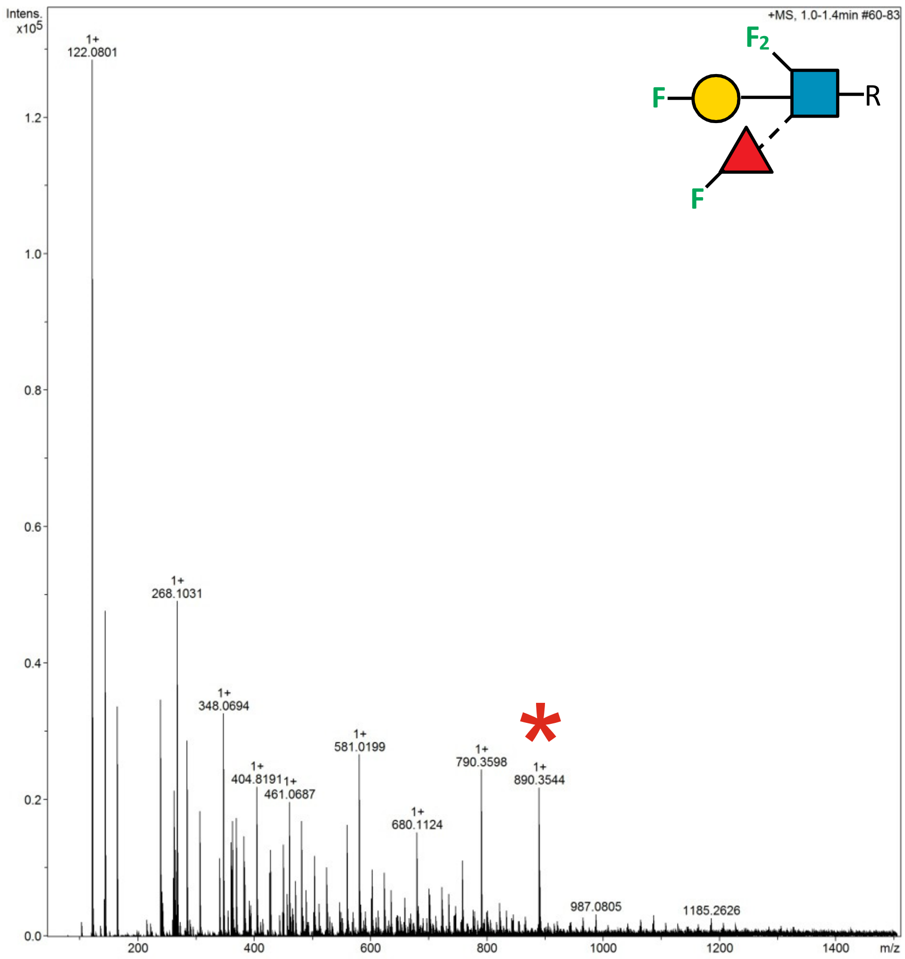

2.18.78 Supplementary Figure 95. HRMS ITag screening assay mass spectrum of synthesis of 4F-Gal β1-4 (3F-Fuc α1-3) 6,6-diFGlcNTFA-ITag

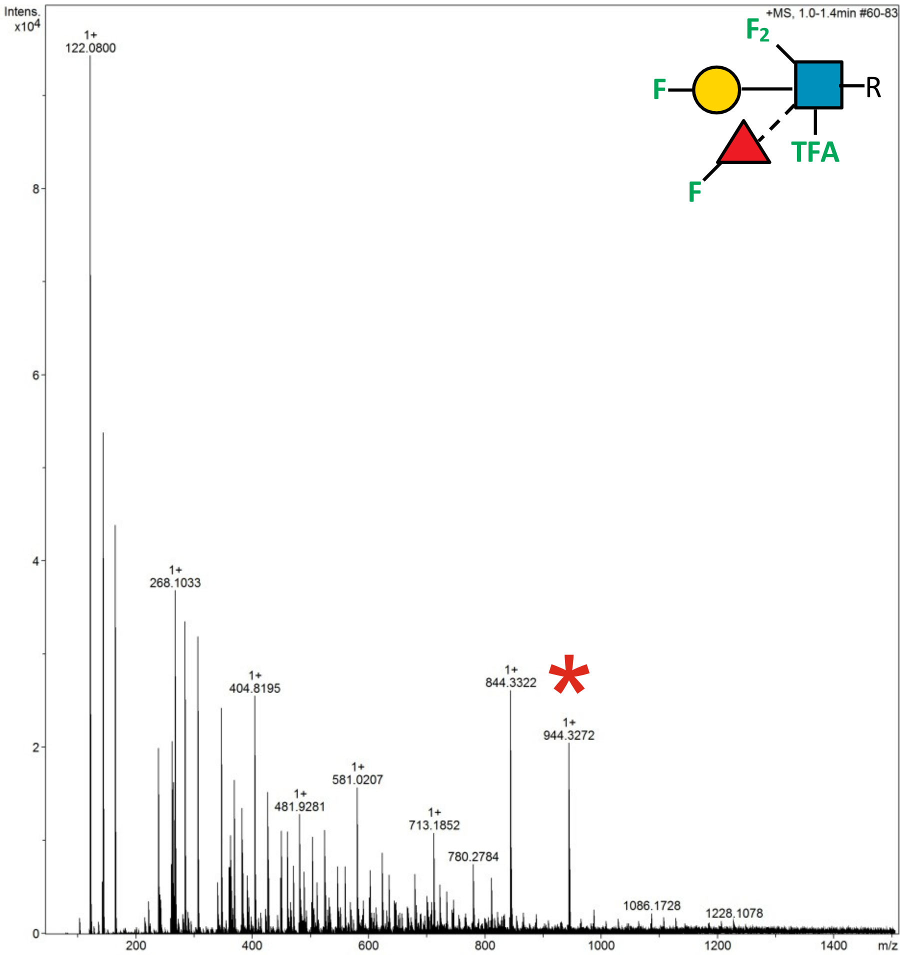

2.18.79 Supplementary Figure 96. HRMS ITag screening assay mass spectrum of synthesis of 6F-Gal β1-4 (3F-Fuc α1-3) GlcNAc-ITag

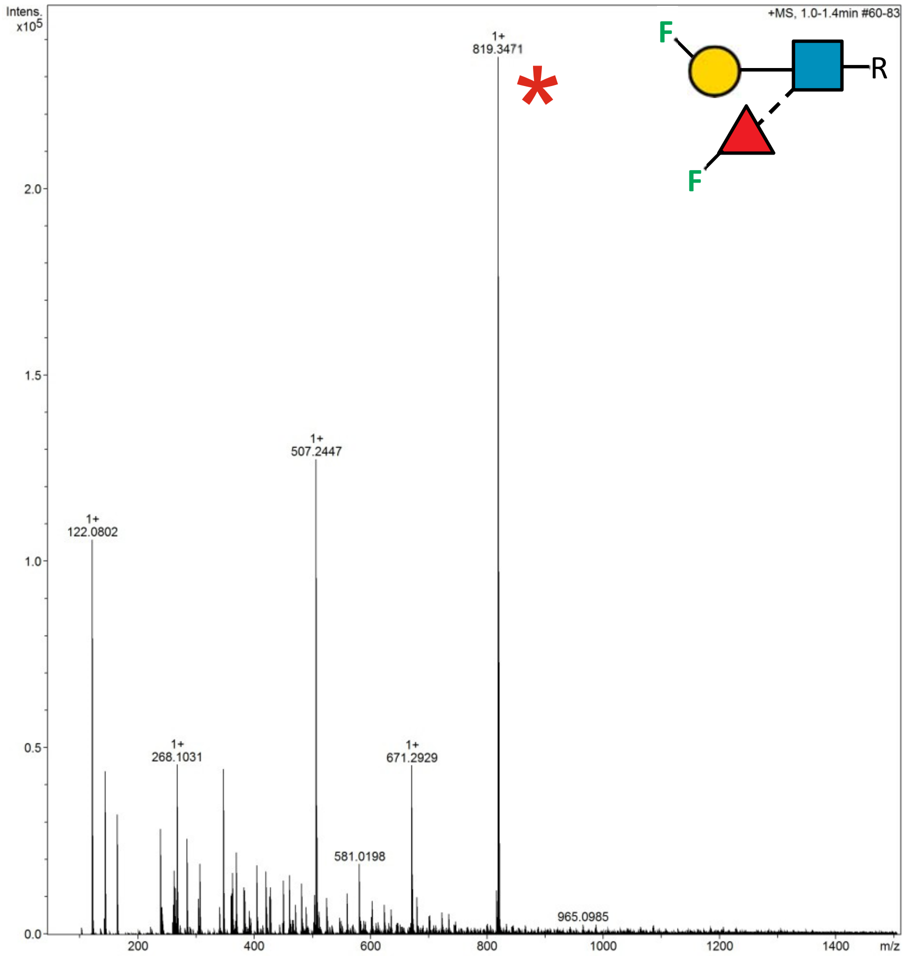

2.18.80 Supplementary Figure 97. HRMS ITag screening assay mass spectrum of synthesis of 6F-Gal β1-4 (3F-Fuc α1-3) GlcNTFA-ITag

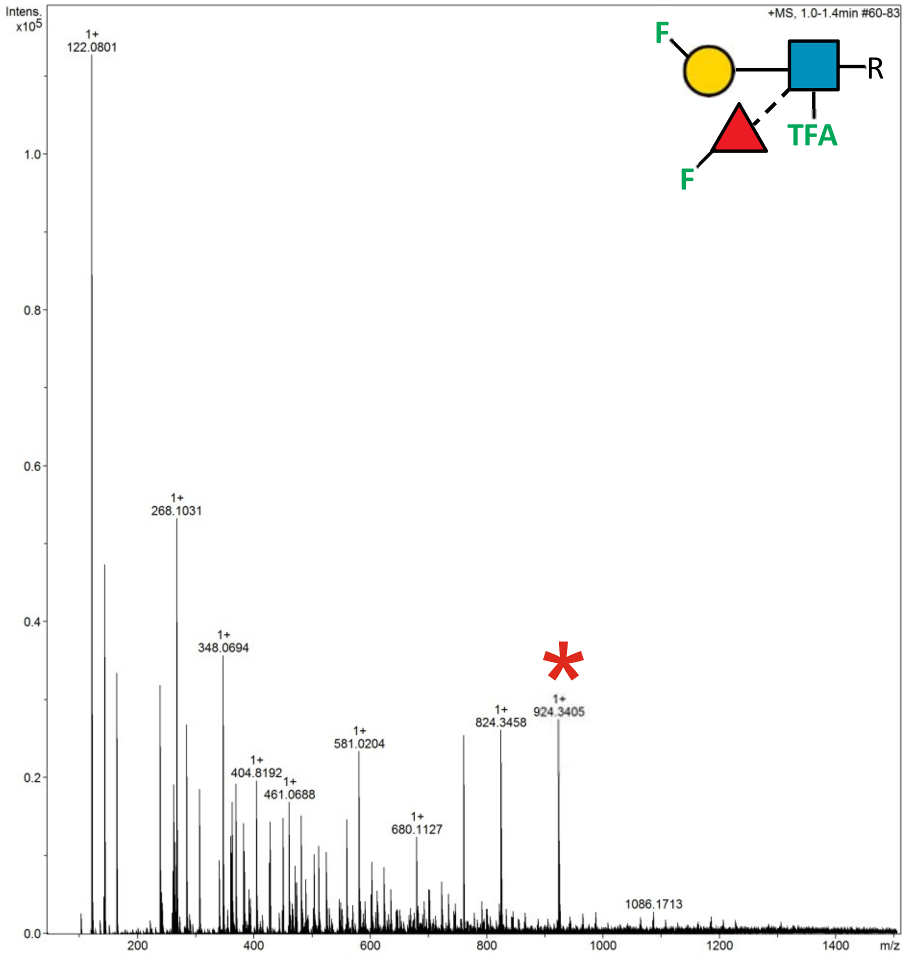

2.18.81 Supplementary Figure 98. HRMS ITag screening assay mass spectrum of synthesis of 6F-Gal β1-4 (3F-Fuc α1-3) 6F-GlcNAc-ITag

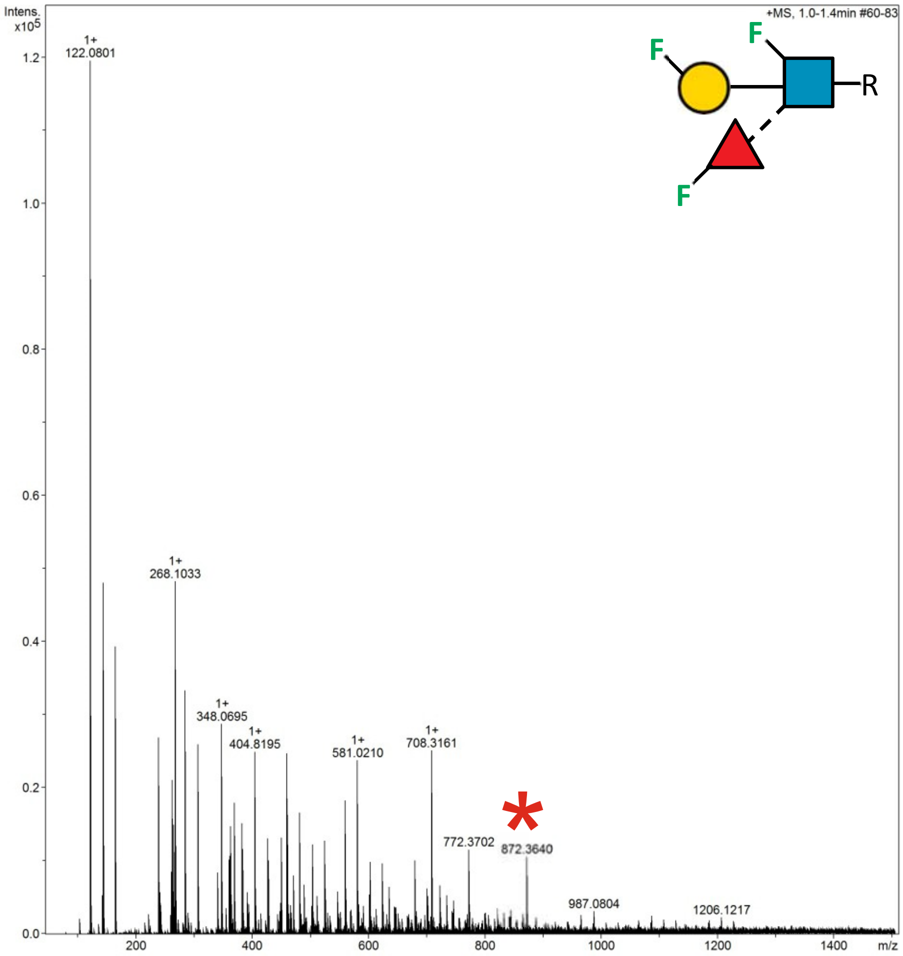

2.18.82 Supplementary Figure 99. HRMS ITag screening assay mass spectrum of synthesis of 6F-Gal β1-4 (3F-Fuc α1-3) 6F-GlcNTFA-ITag

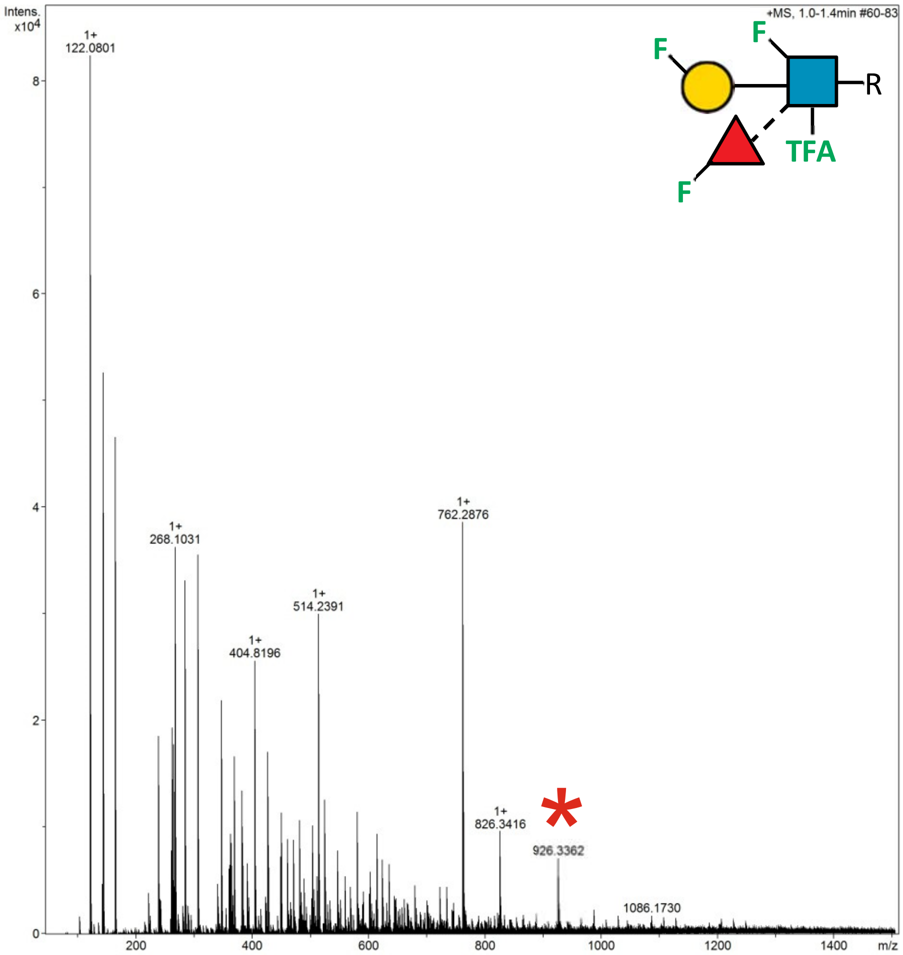

2.18.83 Supplementary Figure 100. HRMS ITag screening assay mass spectrum of synthesis of 6F-Gal β1-4 (3F-Fuc α1-3) 6,6-diFGlcNAc-ITag

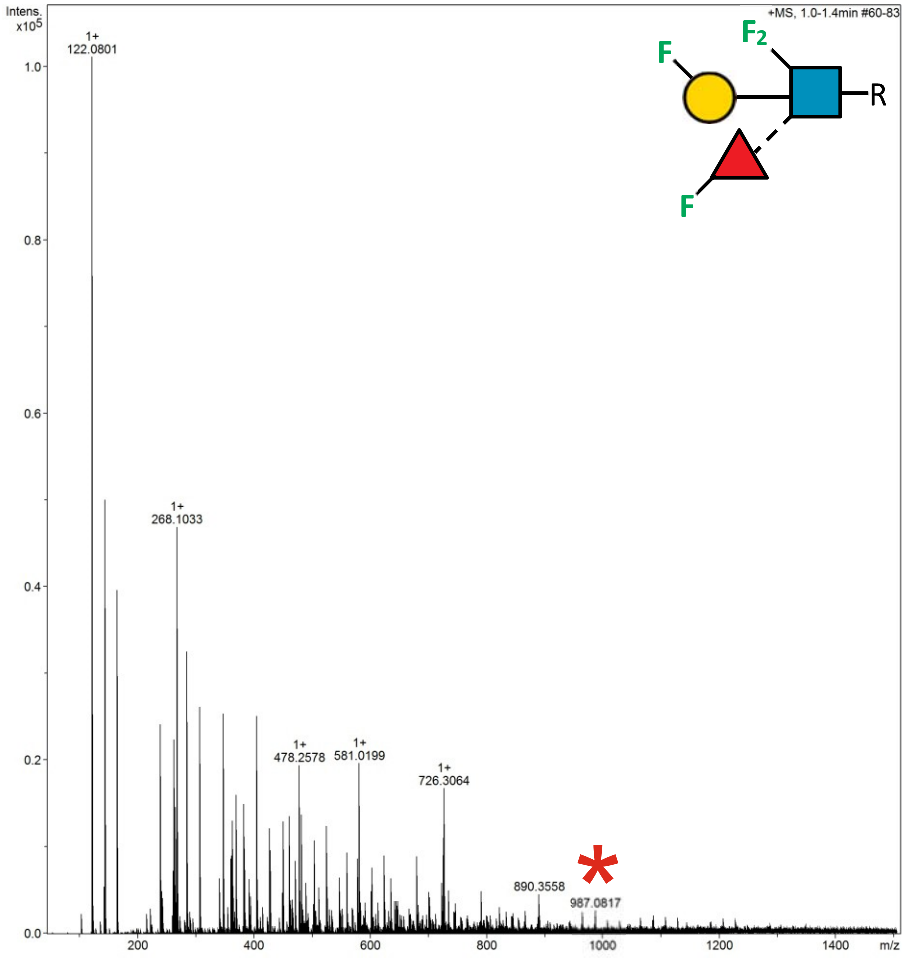

2.18.84 Supplementary Figure 101. HRMS ITag screening assay mass spectrum of synthesis of 6F-Gal β1-4 (3F-Fuc α1-3) 6,6-diFGlcNTFA-ITag

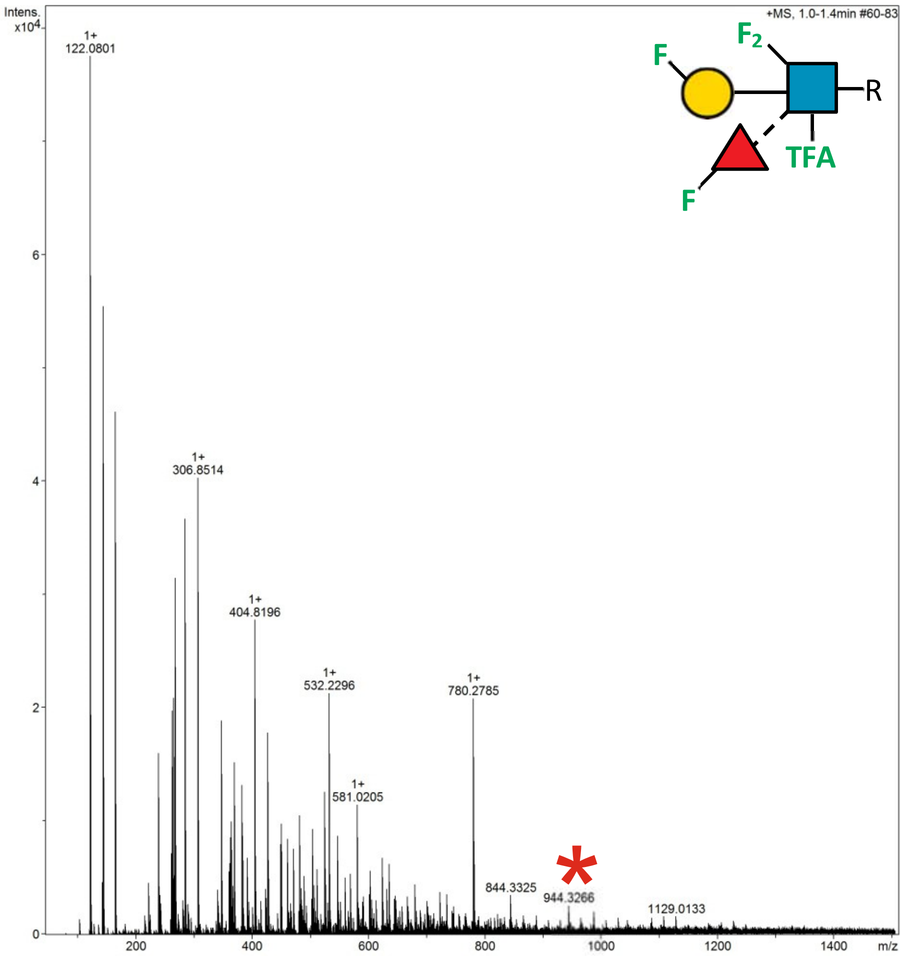

2.18.85 Supplementary Figure 102. HRMS ITag screening assay mass spectrum of synthesis of 6d-Gal β1-4 (3F-Fuc α1-3) GlcNAc-ITag

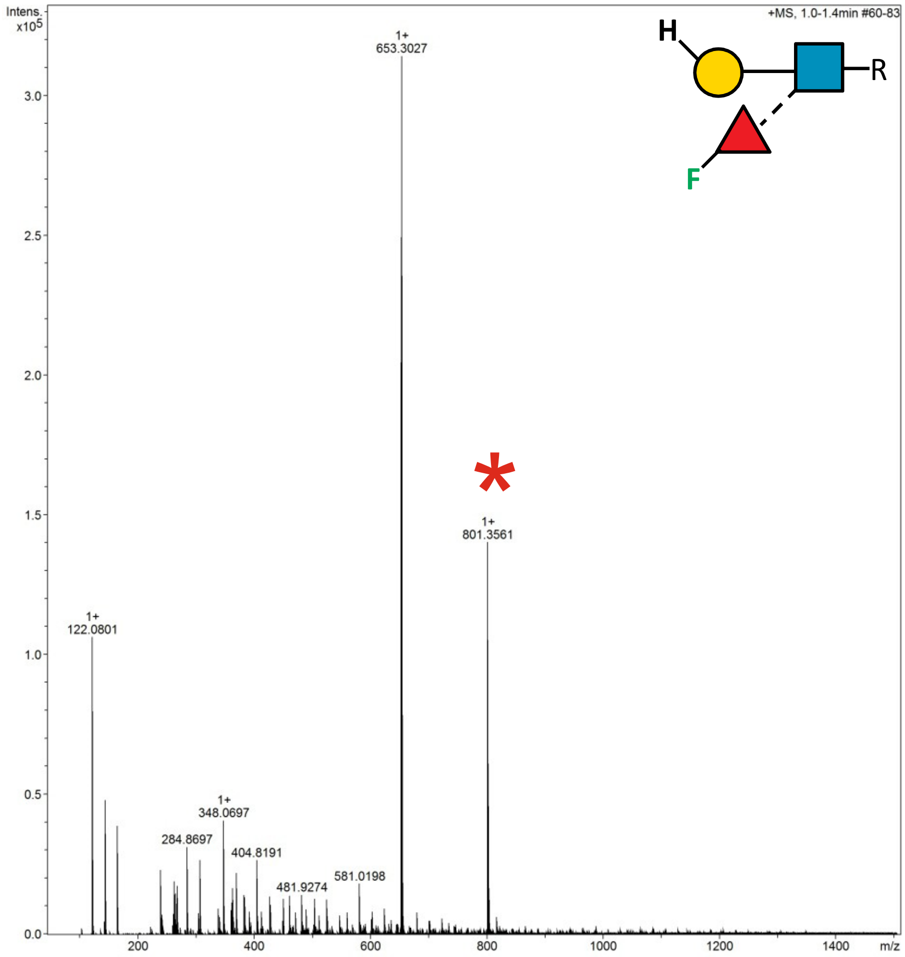

2.18.86 Supplementary Figure 103. HRMS ITag screening assay mass spectrum of synthesis of 6d-Gal β1-4 (3F-Fuc α1-3) GlcNTFA-ITag

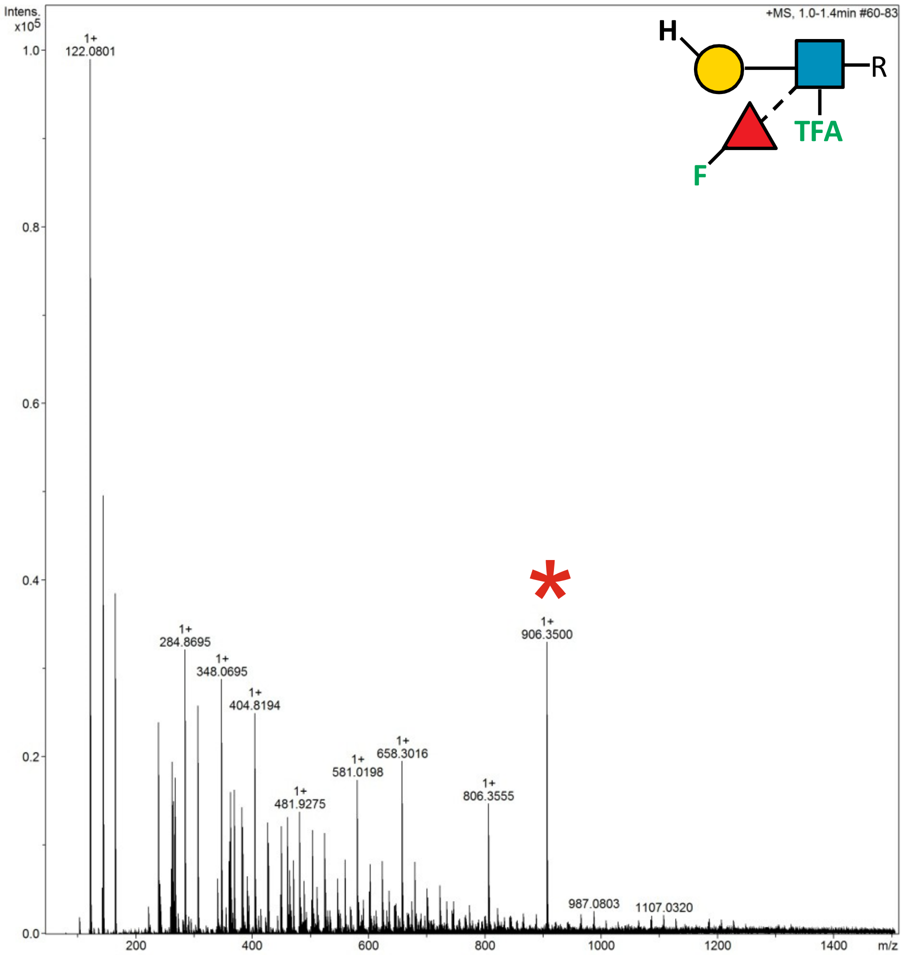

2.18.87 Supplementary Figure 104. HRMS ITag screening assay mass spectrum of synthesis of 6d-Gal β1-4 (3F-Fuc α1-3) 6F-GlcNAc-ITag

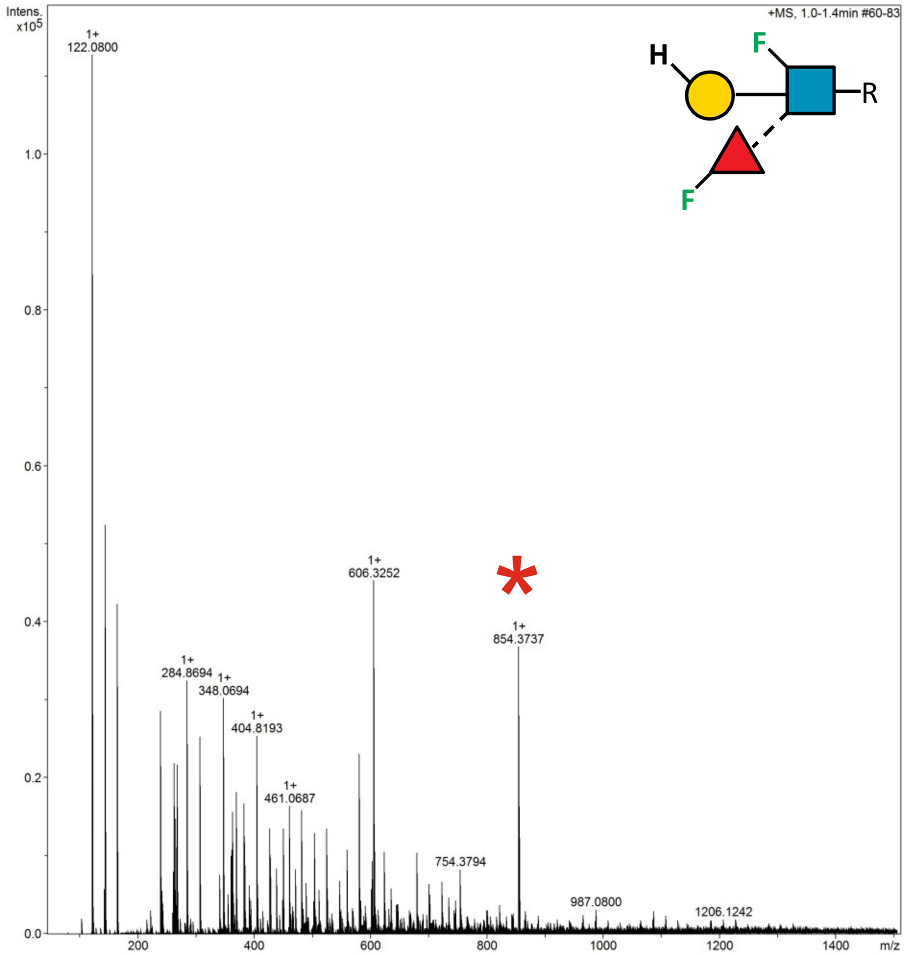

2.18.88 Supplementary Figure 105. HRMS ITag screening assay mass spectrum of synthesis of 6d-Gal β1-4 (3F-Fuc α1-3) 6F-GlcNTFA-ITag

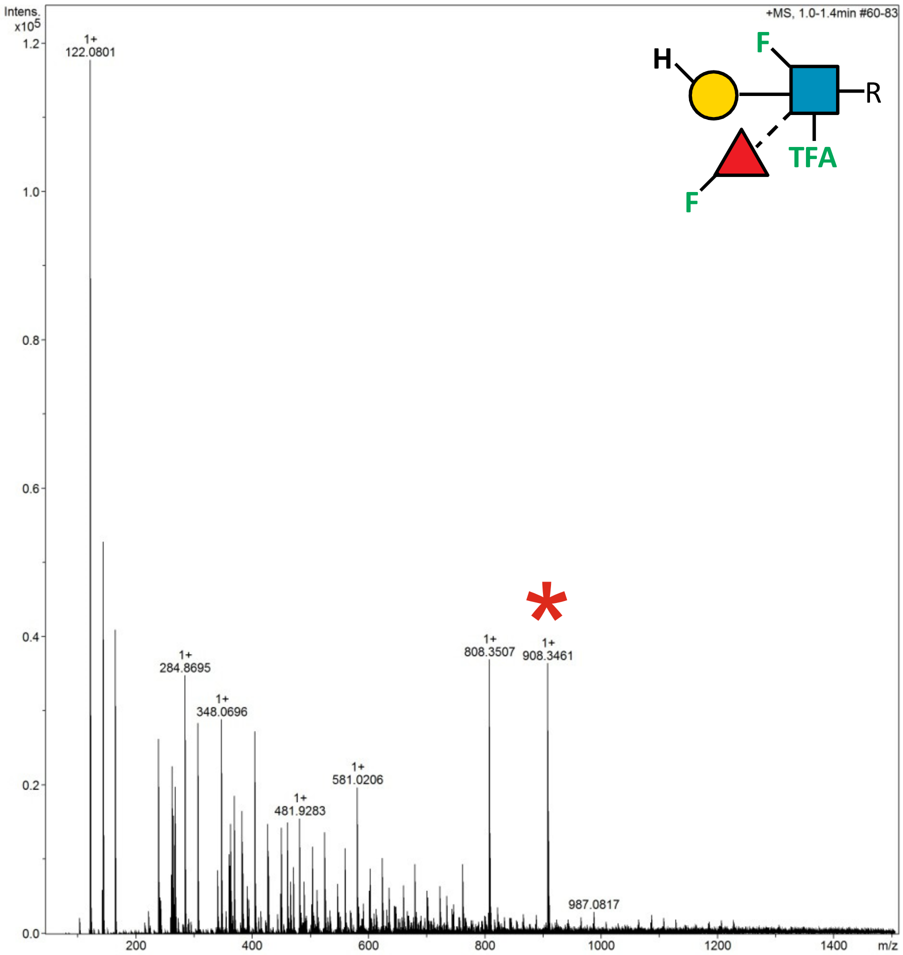

2.18.89 Supplementary Figure 106. HRMS ITag screening assay mass spectrum of synthesis of **6d-Gal β1-4 (3F-Fuc α1-3) 6,6-diFGlcNAc-ITag**

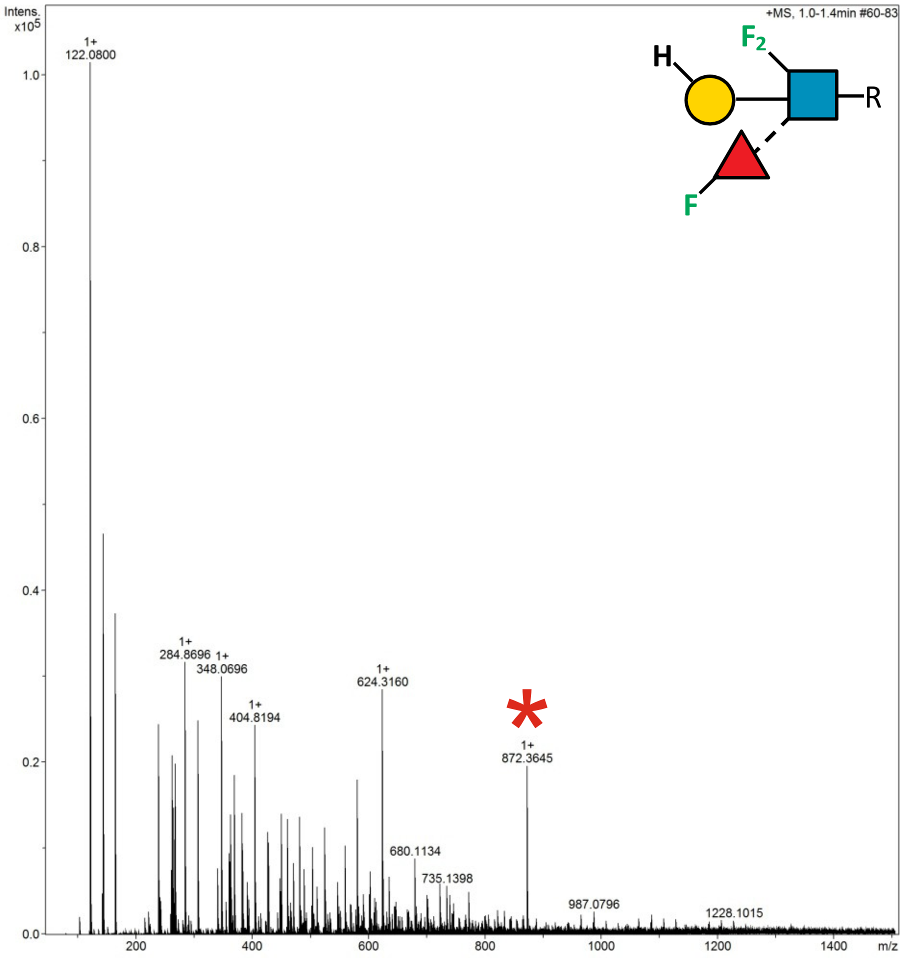

2.18.90 Supplementary Figure 107. HRMS ITag screening assay mass spectrum of synthesis of **6d-Gal β1-4 (3F-Fuc α1-3) 6,6-diFGlcNTFA-ITag**

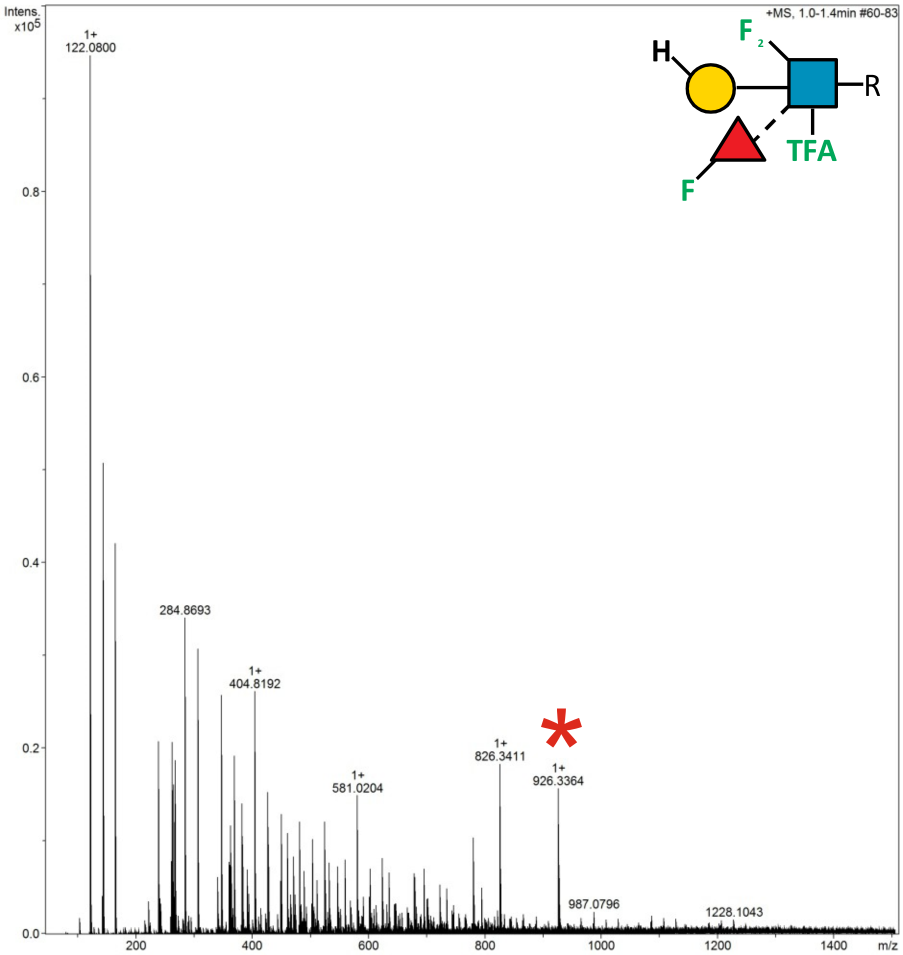

2.18.91 Supplementary Figure 108. HRMS ITag screening assay mass spectrum of synthesis of **Gal β1-4 (4F-Fuc α1-3) GlcNAc-ITag (LeX12)**

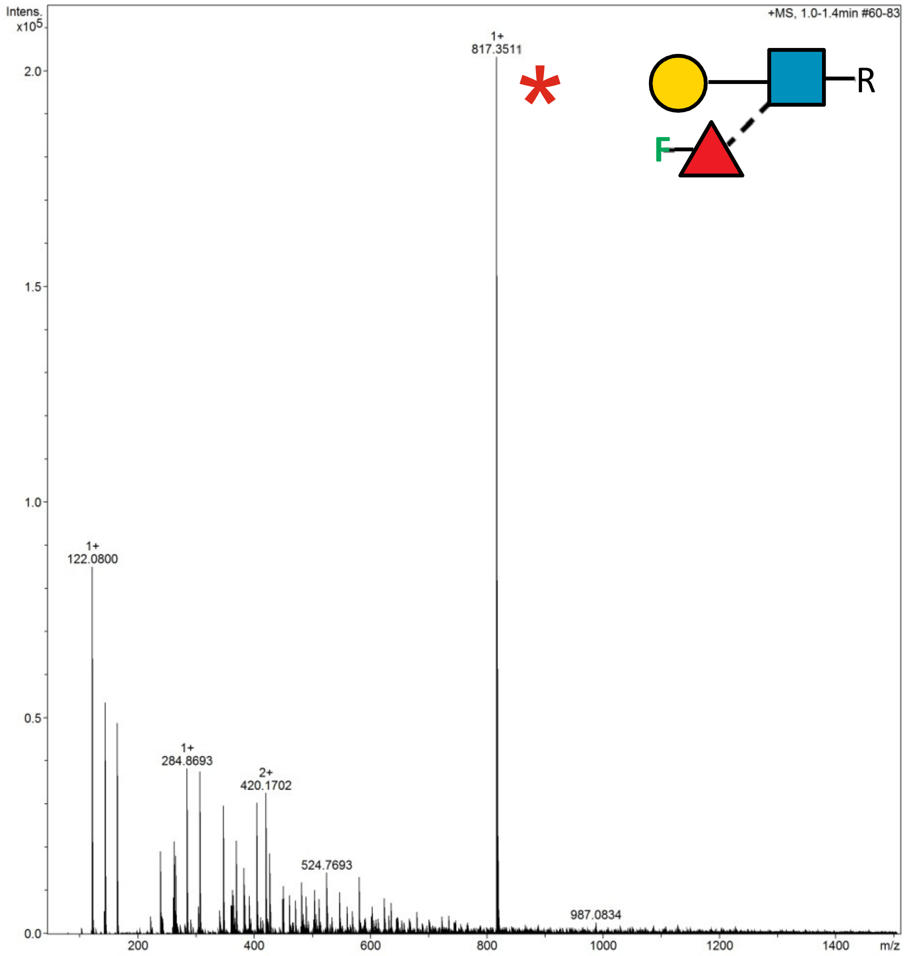

2.18.92 Supplementary Figure 109. HRMS ITag screening assay mass spectrum of synthesis of **Gal β1-4 (4F-Fuc α1-3) GlcNTFA-ITag**

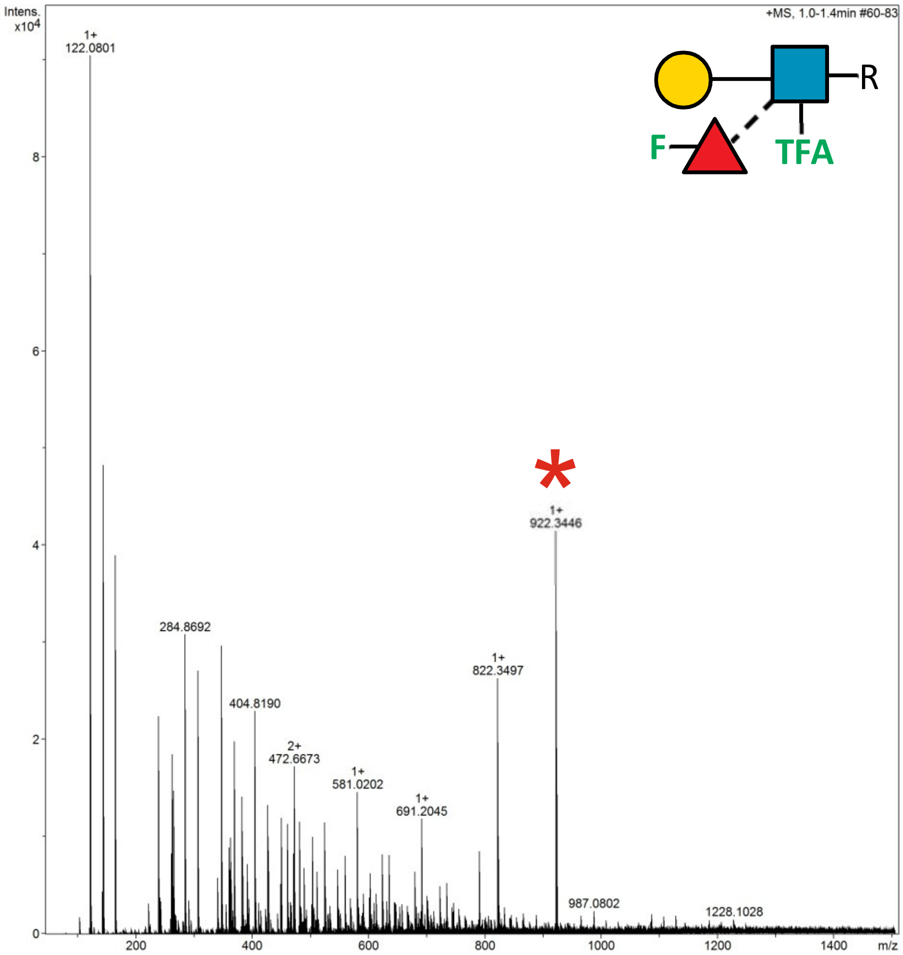

2.18.93 Supplementary Figure 110. HRMS ITag screening assay mass spectrum of synthesis of **Gal β1-4 (4F-Fuc α1-3) 6F-GlcNAc-ITag**

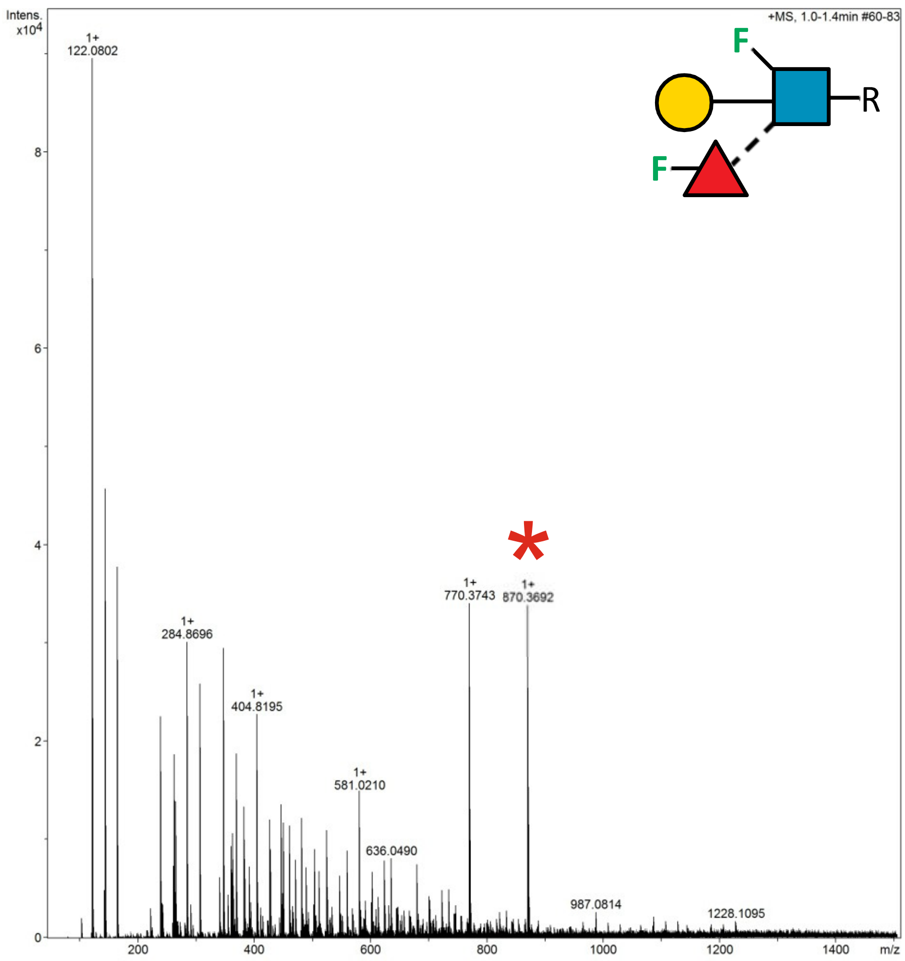

2.18.94 Supplementary Figure 111. HRMS ITag screening assay mass spectrum of synthesis of Gal β1-4 (4F-Fuc α1-3) 6F-GlcNTFA-ITag (LeX20)

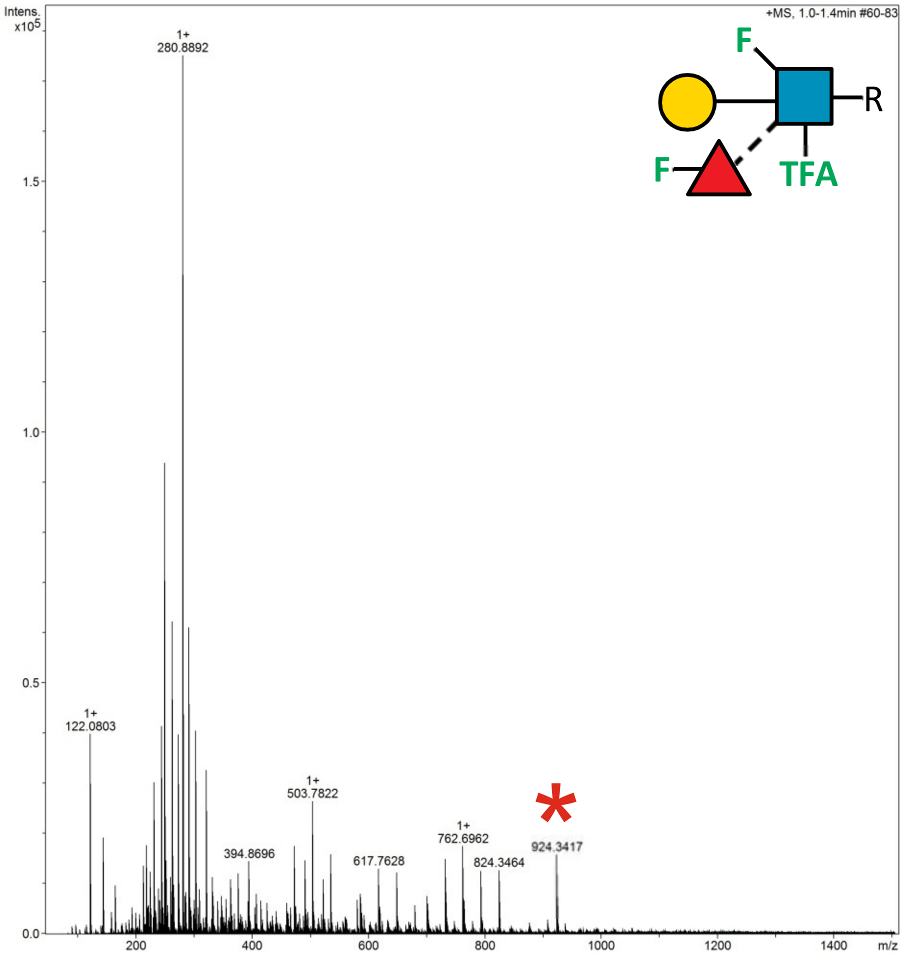

2.18.95 Supplementary Figure 112. HRMS ITag screening assay mass spectrum of synthesis of **Gal β1-4 (4F-Fuc α1-3) 6,6-diFGlcNAc-ITag**

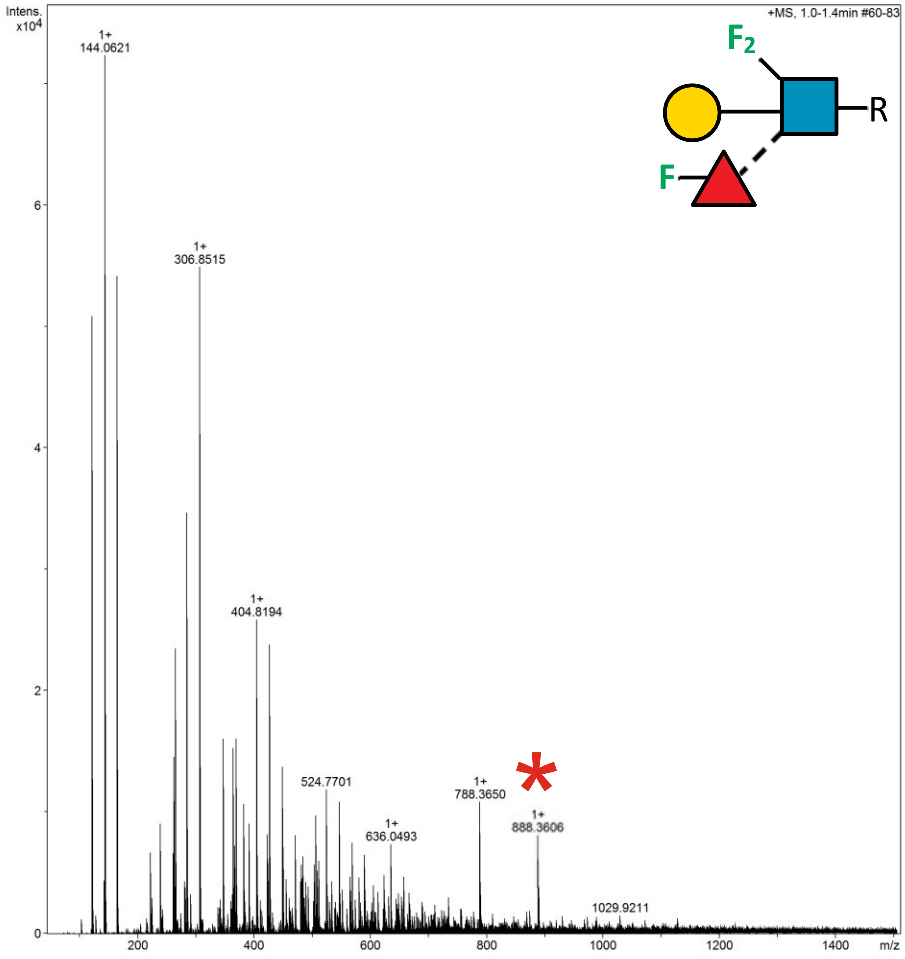

2.18.96 Supplementary Figure 113. HRMS ITag screening assay mass spectrum of synthesis of **Gal β1-4 (4F-Fuc α1-3) 6,6-diFGlcNTFA-ITag**

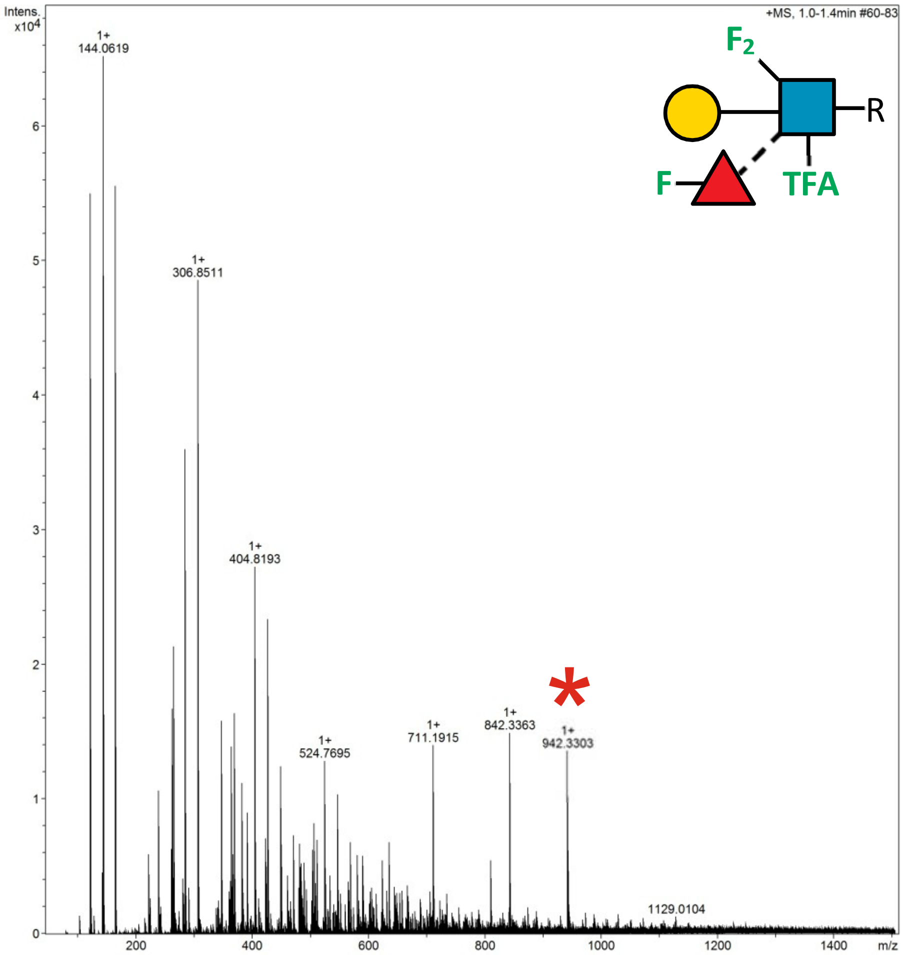

2.18.97 Supplementary Figure 114. HRMS ITag screening assay mass spectrum of synthesis of **3F-Gal β1-4 (4F-Fuc α1-3) GlcNAc-ITag**

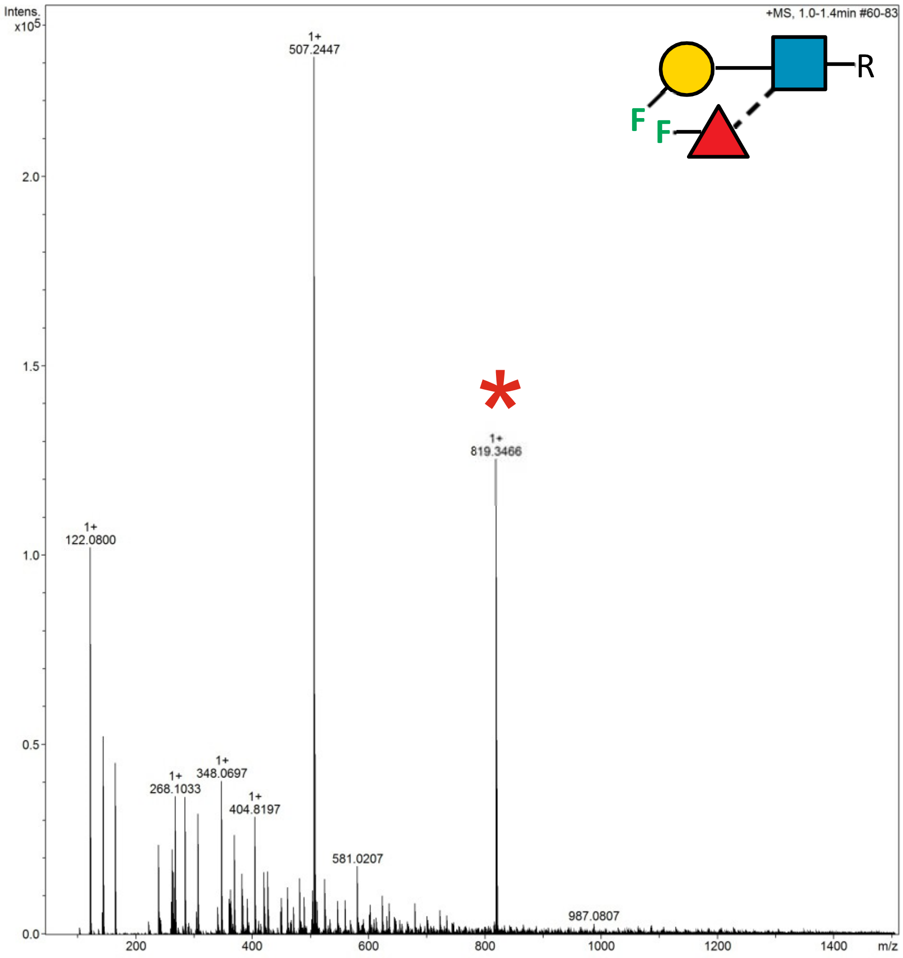

2.18.98 Supplementary Figure 115. HRMS ITag screening assay mass spectrum of synthesis of **3F-Gal β1-4 (4F-Fuc α1-3) GlcNTFA-ITag**

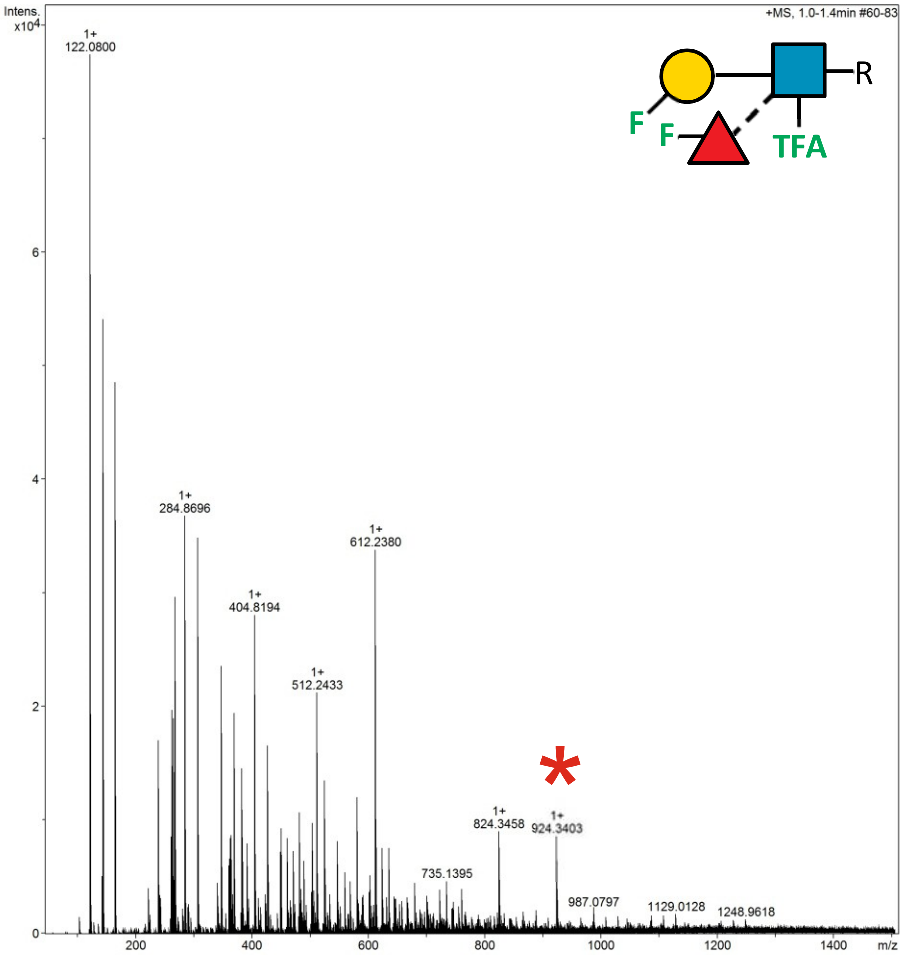

2.18.99 Supplementary Figure 116. HRMS ITag screening assay mass spectrum of synthesis of **3F-Gal β1-4 (4F-Fuc α1-3) 6F-GlcNAc-ITag**

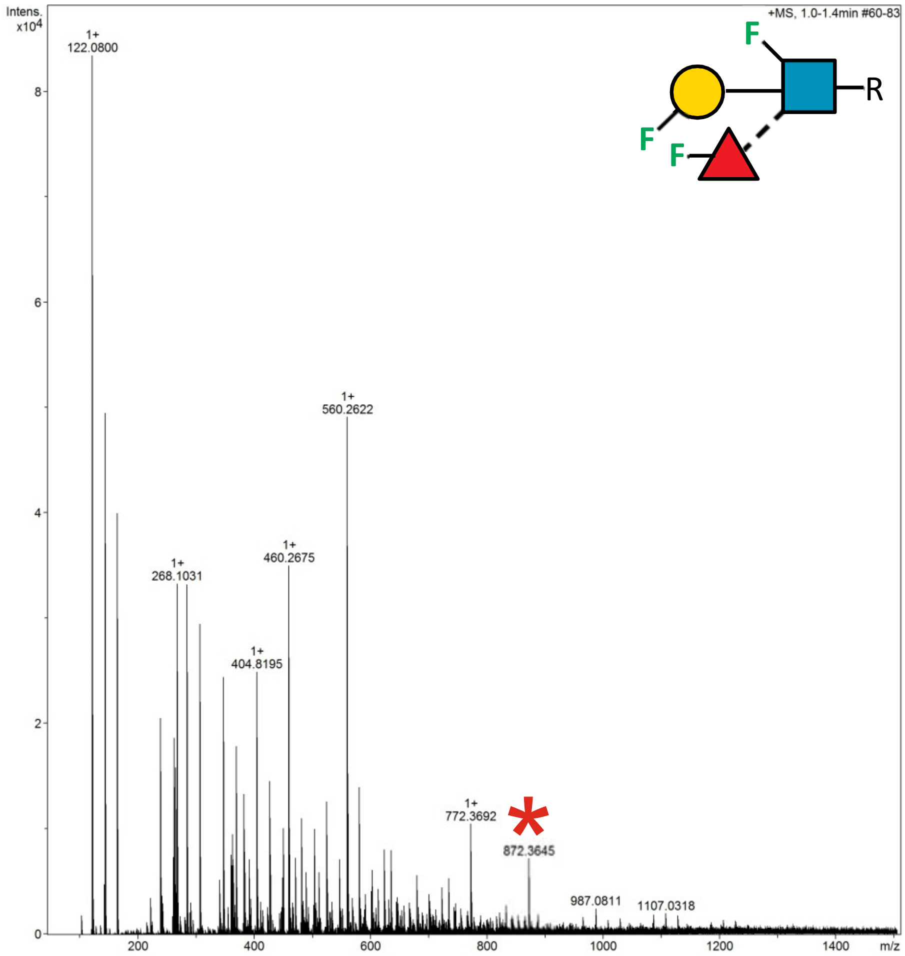

2.18.100 Supplementary Figure 117. HRMS ITag screening assay mass spectrum of synthesis of 3F-Gal β1-4 (4F-Fuc α1-3) 6F-GlcNTFA-ITag

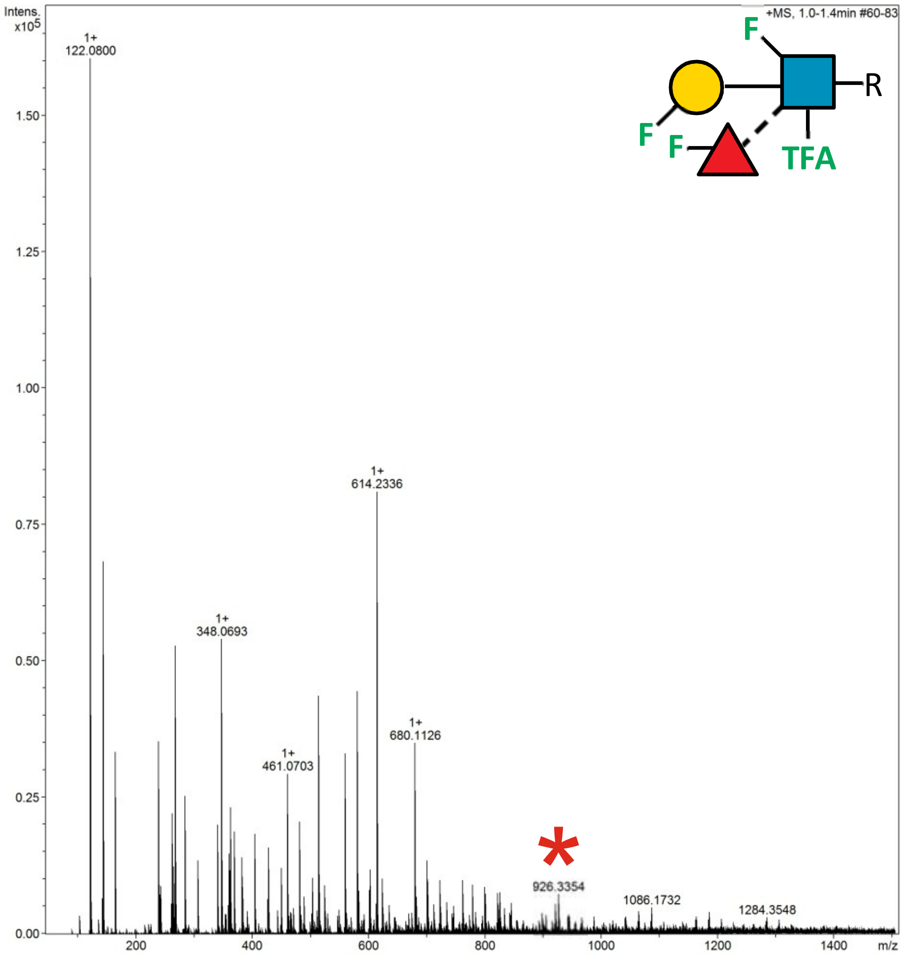

2.18.101 Supplementary Figure 118. HRMS ITag screening assay mass spectrum of synthesis of **3F-Gal β1-4 (4F-Fuc α1-3) 6,6-diFGlcNAc-ITag**

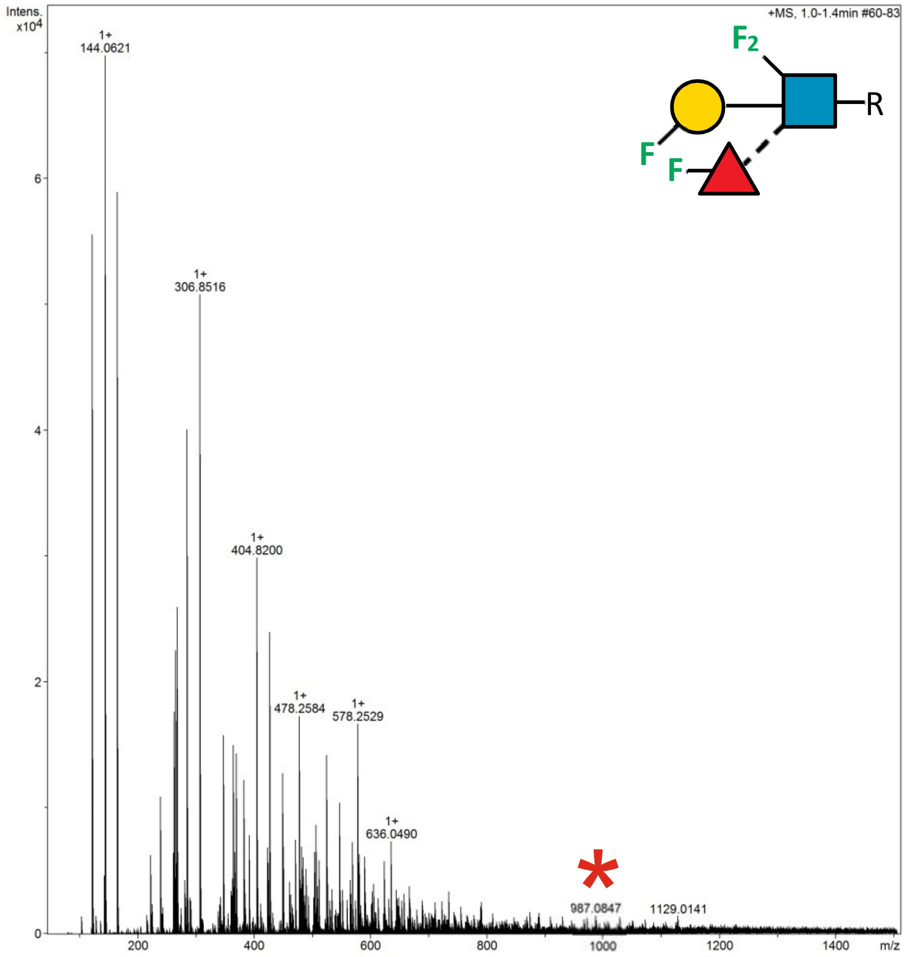

2.18.102 Supplementary Figure 119. HRMS ITag screening assay mass spectrum of synthesis of **3F-Gal β1-4 (4F-Fuc α1-3) 6,6-diFGlcNTFA-ITag**

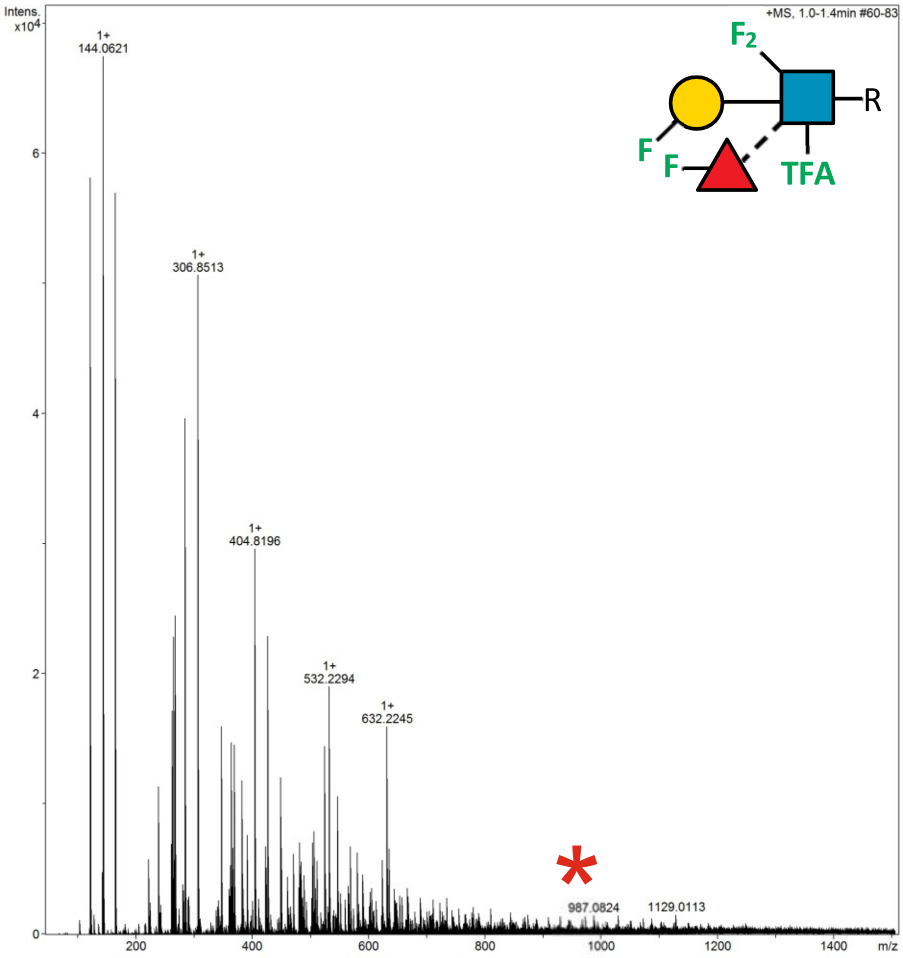

2.18.103 Supplementary Figure 120. HRMS ITag screening assay mass spectrum of synthesis of **4F-Gal β1-4 (4F-Fuc α1-3) GlcNAc-ITag**

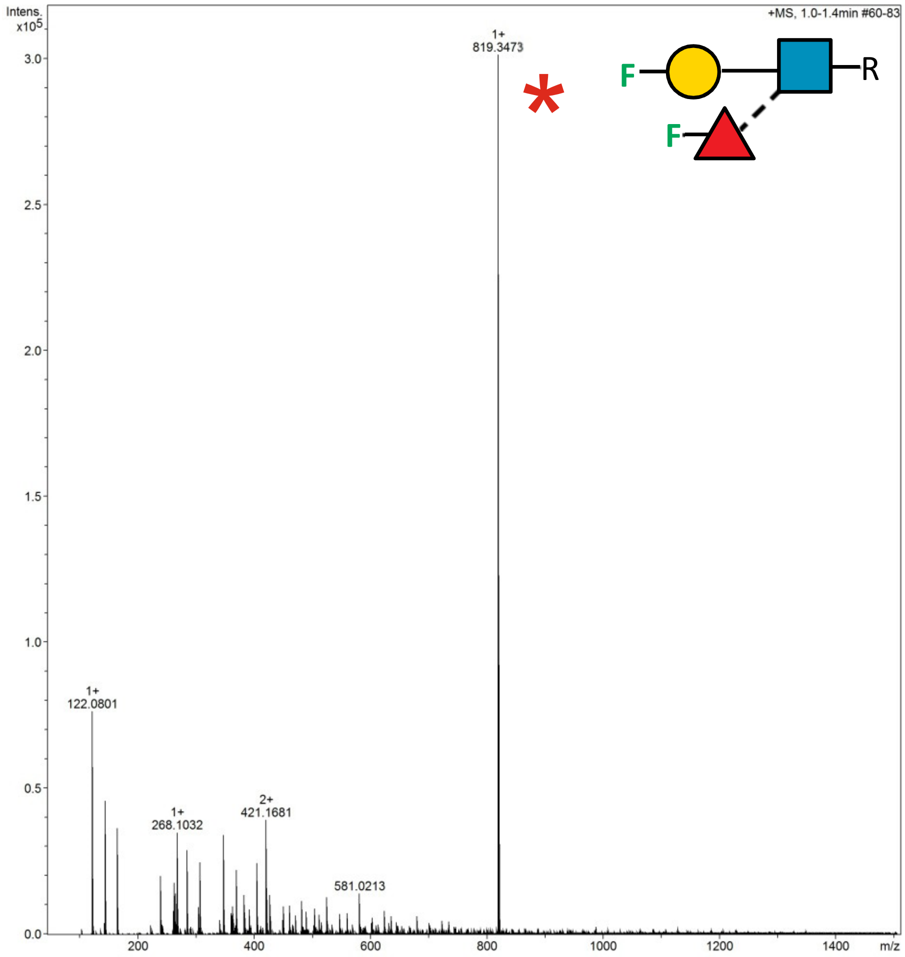

2.18.104 Supplementary Figure 121. HRMS ITag screening assay mass spectrum of synthesis of **4F-Gal β1-4 (4F-Fuc α1-3) GlcNTFA-ITag**

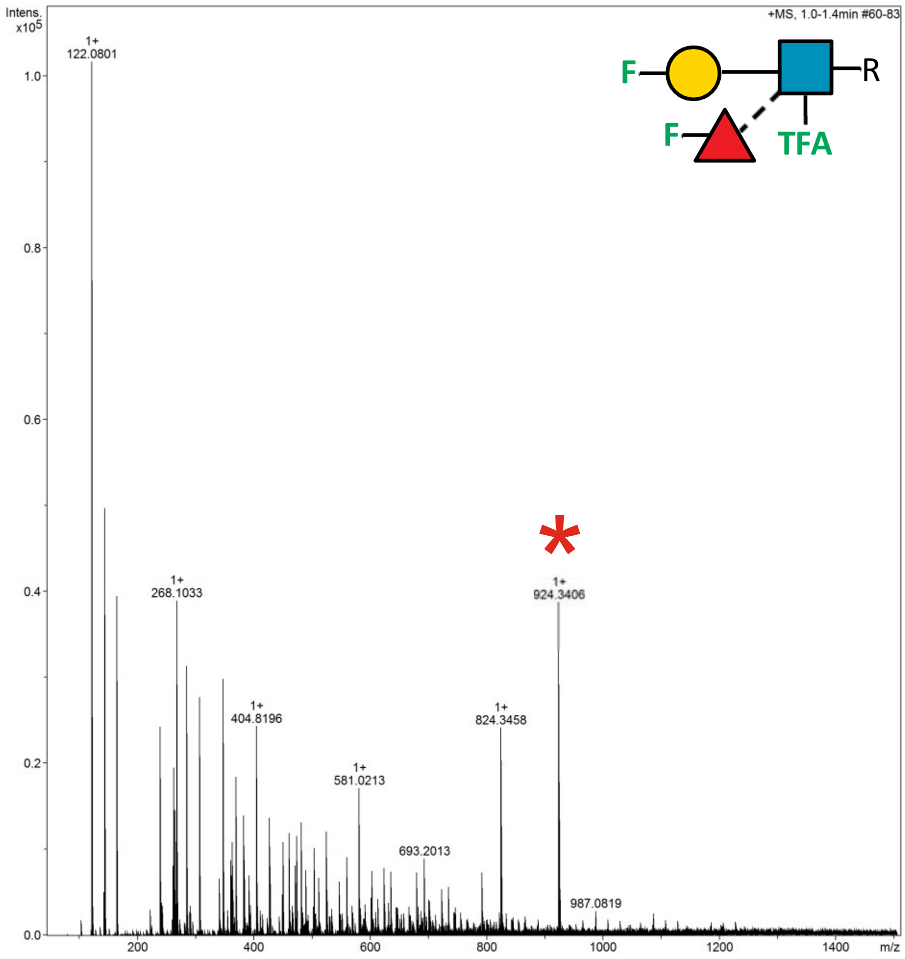

2.18.105 Supplementary Figure 122. HRMS ITag screening assay mass spectrum of synthesis of **4F-Gal β1-4 (4F-Fuc α1-3) 6F-GlcNAc-ITag**

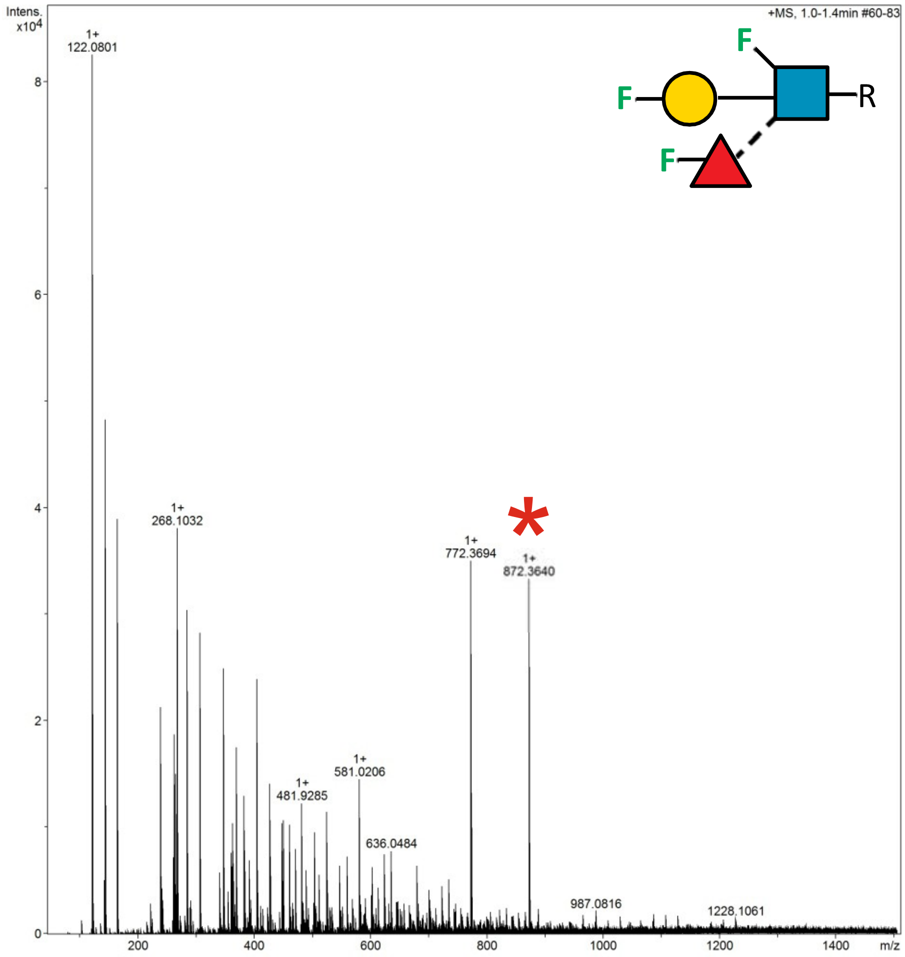

2.18.106 Supplementary Figure 123. HRMS ITag screening assay mass spectrum of synthesis of **4F-Gal β1-4 (4F-Fuc α1-3) 6F-GlcNTFA-ITag**

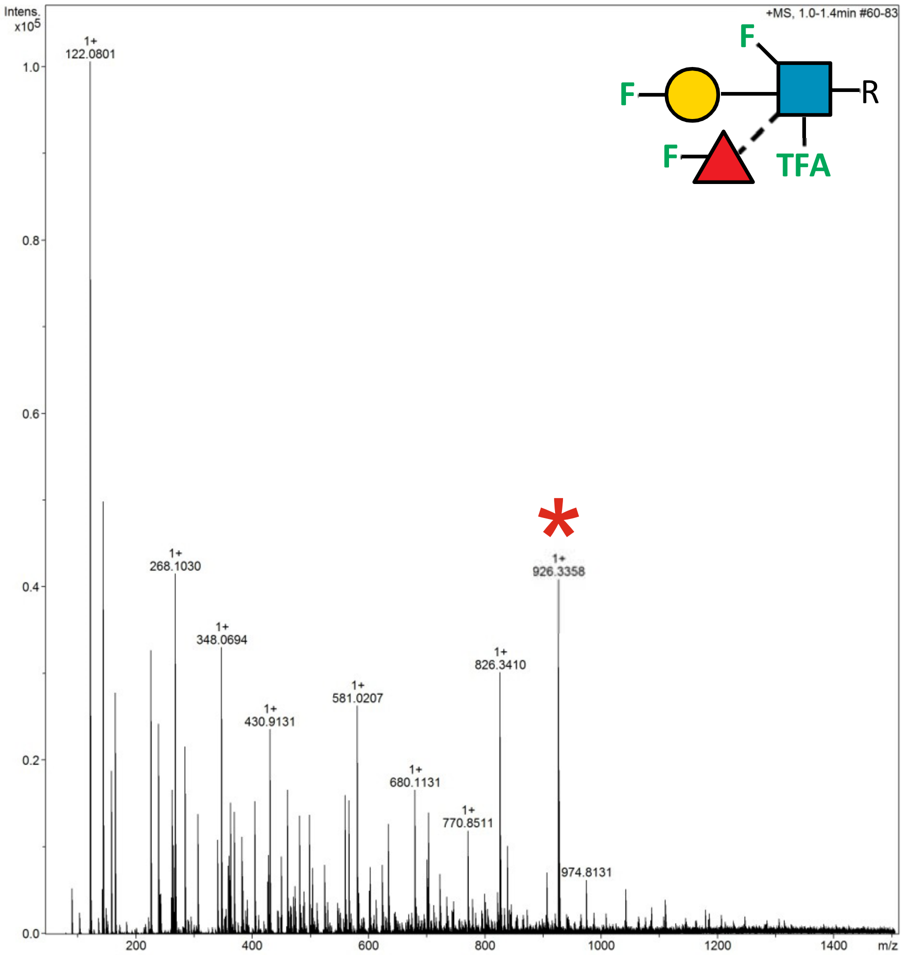

2.18.107 Supplementary Figure 124. HRMS ITag screening assay mass spectrum of synthesis of **4F-Gal β1-4 (4F-Fuc α1-3) 6,6-diFGlcNAc-ITag**

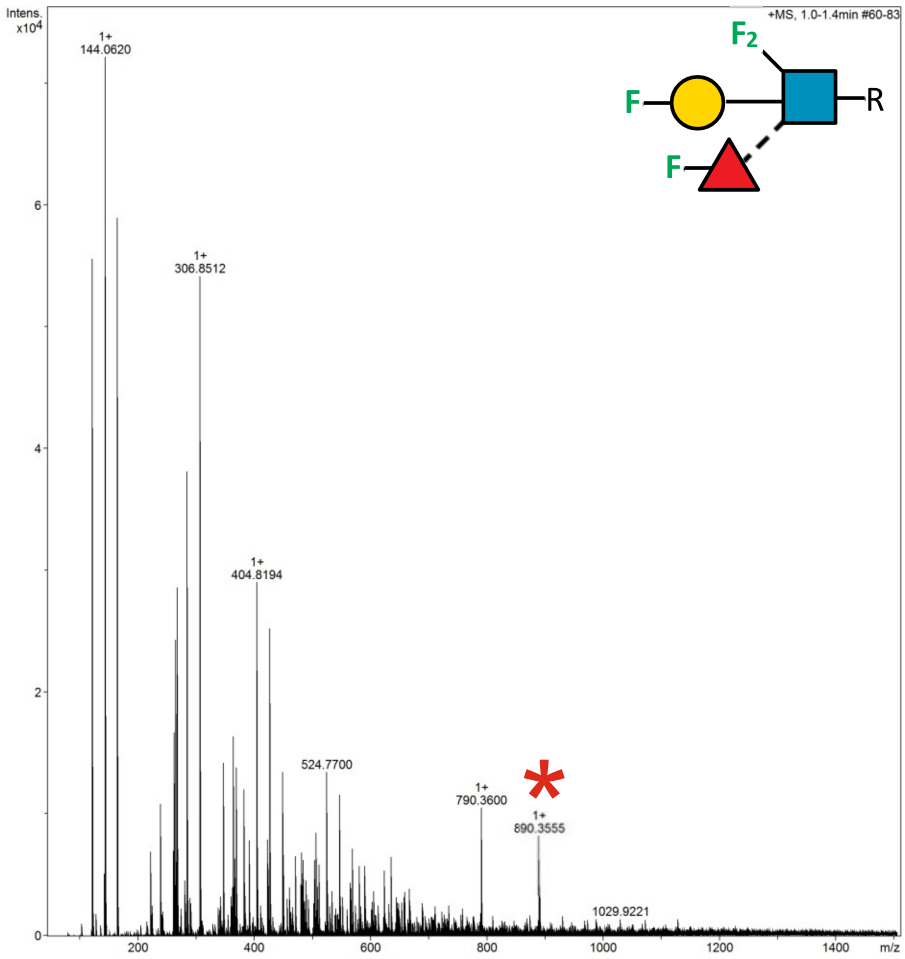

2.18.108 Supplementary Figure 125. HRMS ITag screening assay mass spectrum of synthesis of 4F-Gal β1-4 (4F-Fuc α1-3) 6,6-diFGlcNTFA-ITag

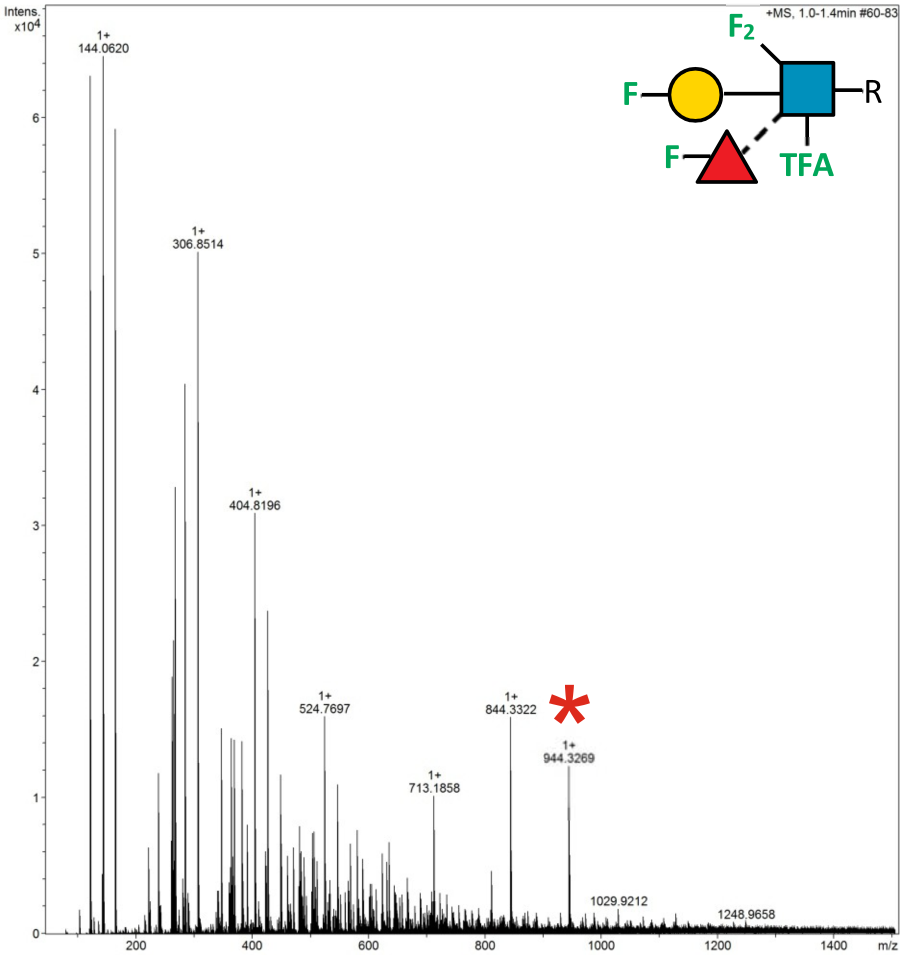

2.18.109 Supplementary Figure 126. HRMS ITag screening assay mass spectrum of synthesis of **6F-Gal β1-4 (4F-Fuc α1-3) GlcNAc-ITag**

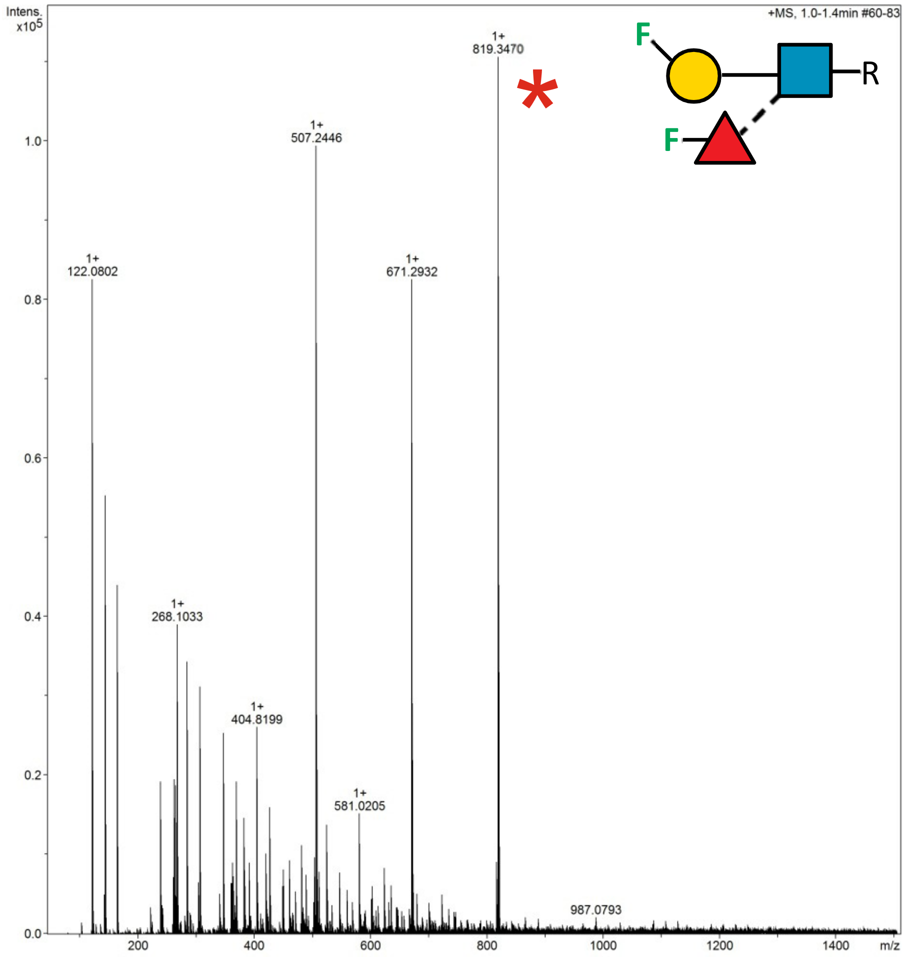

2.18.110 Supplementary Figure 127. HRMS ITag screening assay mass spectrum of synthesis of **6F-Gal β1-4 (4F-Fuc α1-3) GlcNTFA-ITag**

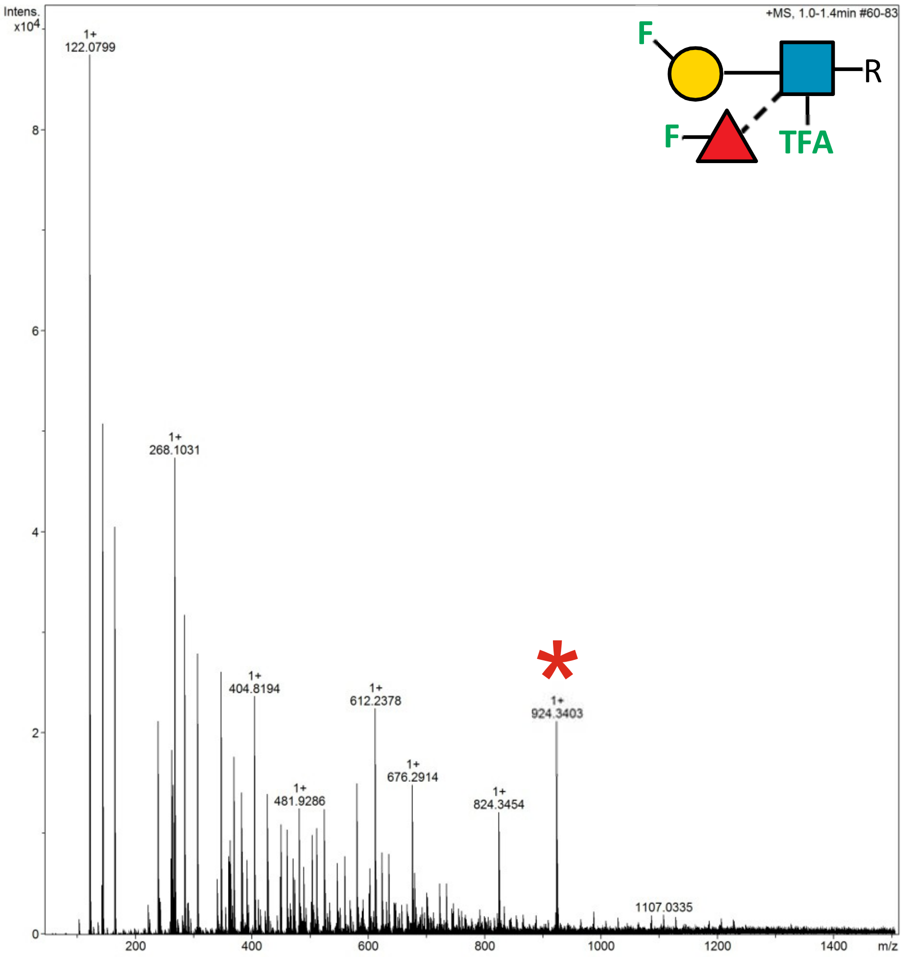

2.18.111 Supplementary Figure 128. HRMS ITag screening assay mass spectrum of synthesis of **6F-Gal β1-4 (4F-Fuc α1-3) 6F-GlcNAc-ITag**

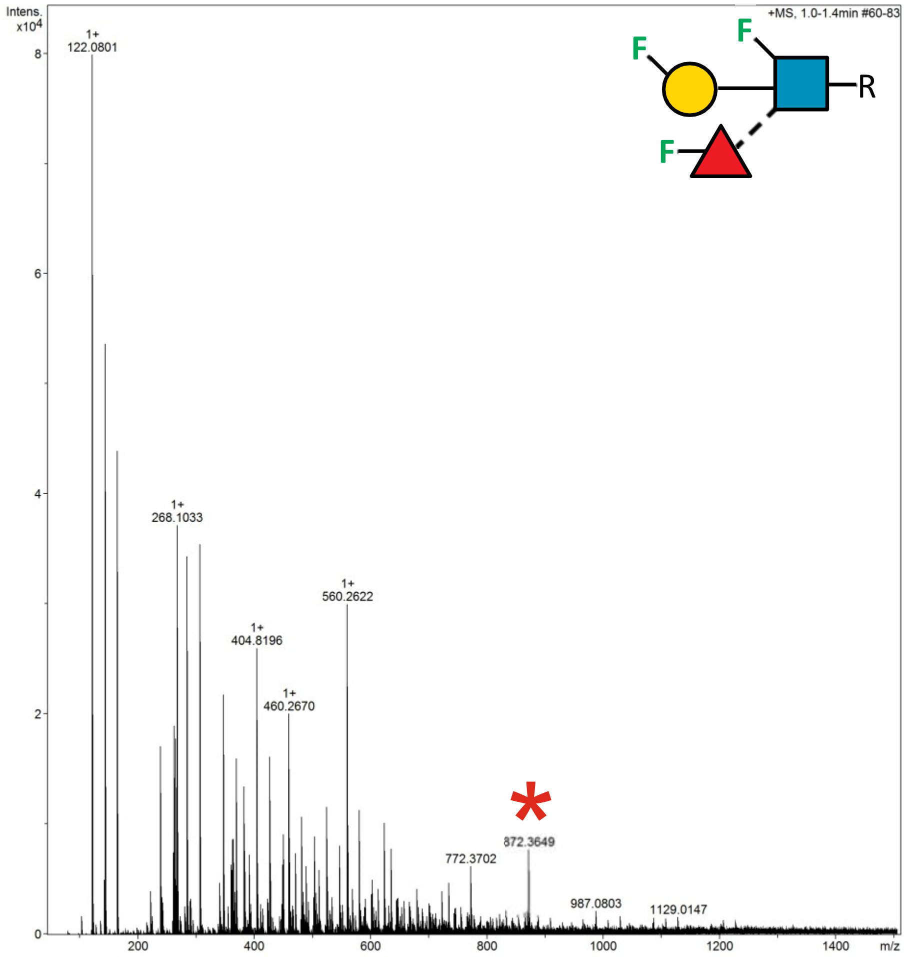

2.18.112 Supplementary Figure 129. HRMS ITag screening assay mass spectrum of synthesis of **6F-Gal β1-4 (4F-Fuc α1-3) 6F-GlcNTFA-ITag**

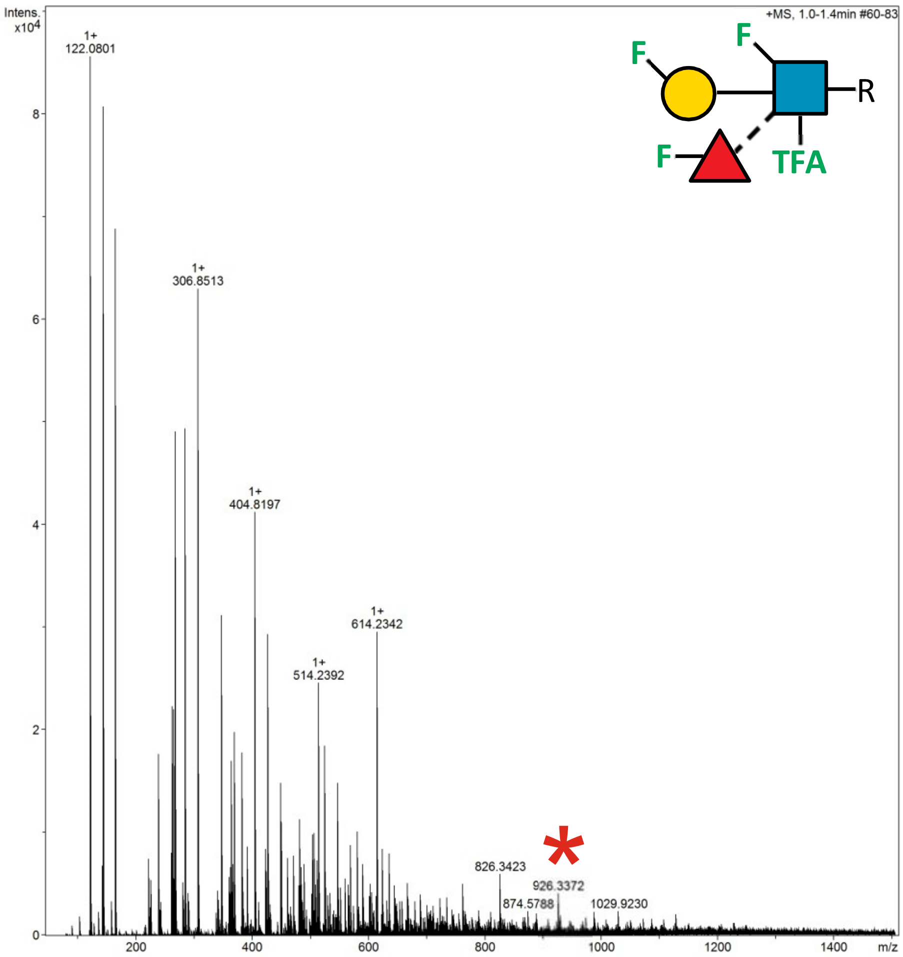

2.18.113 Supplementary Figure 130. HRMS ITag screening assay mass spectrum of synthesis of **6F-Gal β1-4 (4F-Fuc α1-3) 6,6-diFGlcNAc-ITag**

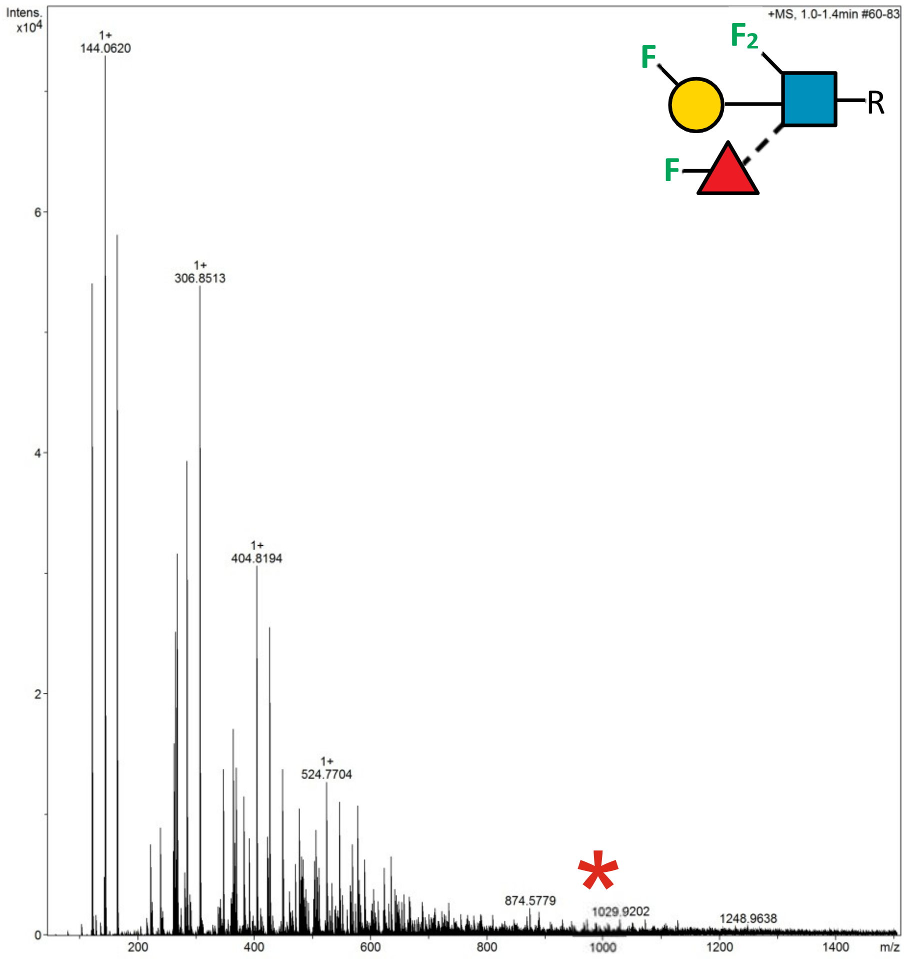

2.18.114 Supplementary Figure 131. HRMS ITag screening assay mass spectrum of synthesis of **6F-Gal β1-4 (4F-Fuc α1-3) 6,6-diFGlcNTFA-ITag**

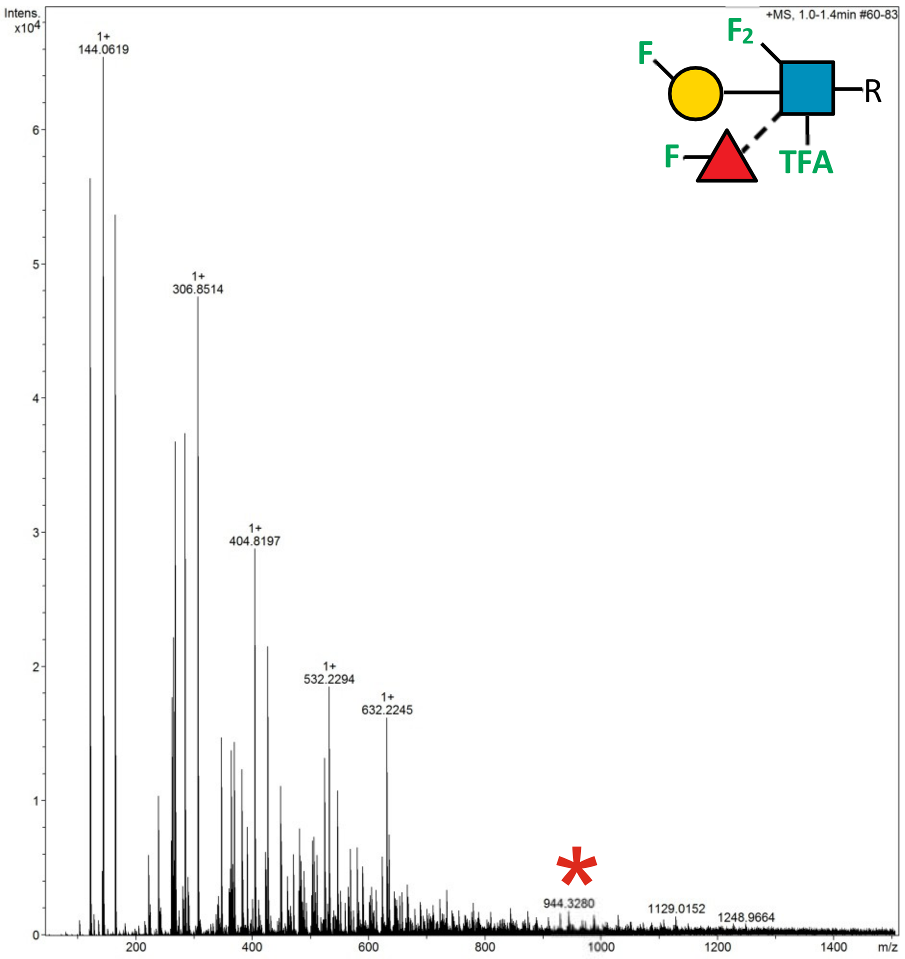

2.18.115 Supplementary Figure 132. HRMS ITag screening assay mass spectrum of synthesis of **6d-Gal β1-4 (3F-Fuc α1-3) GlcNAc-ITag**

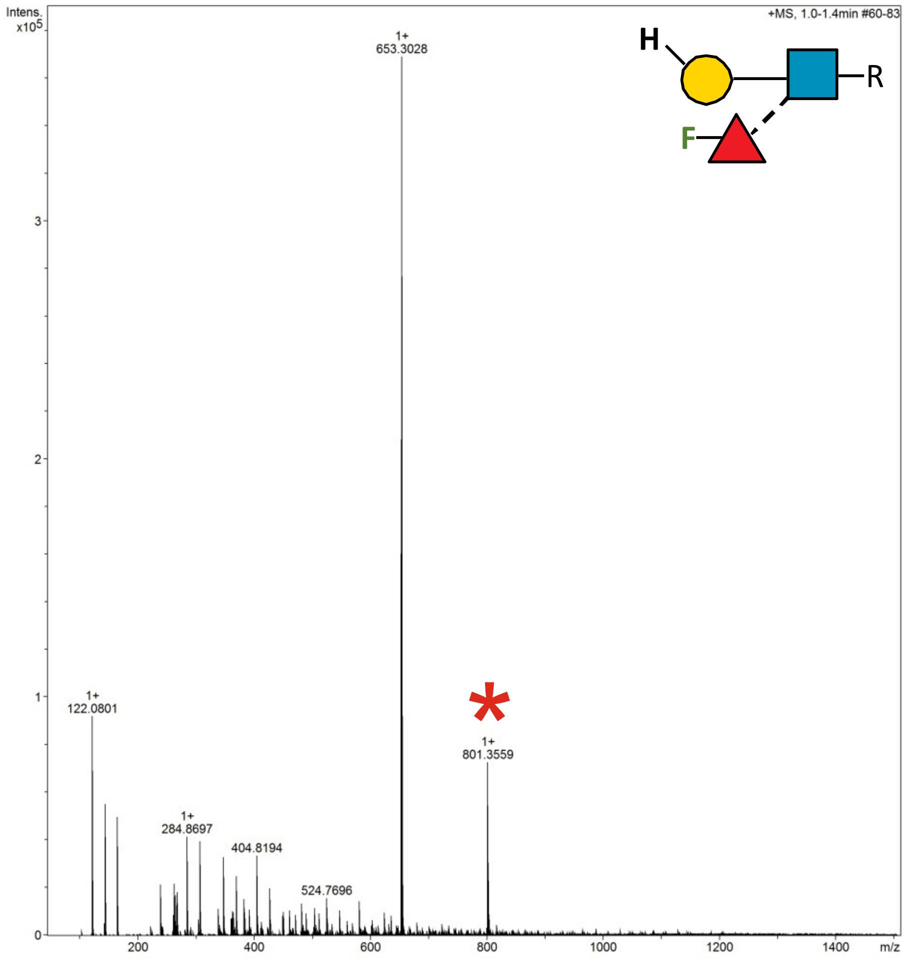

2.18.116 Supplementary Figure 133. HRMS ITag screening assay mass spectrum of synthesis of **6d-Gal β1-4 (4F-Fuc α1-3) GlcNTFA-ITag**

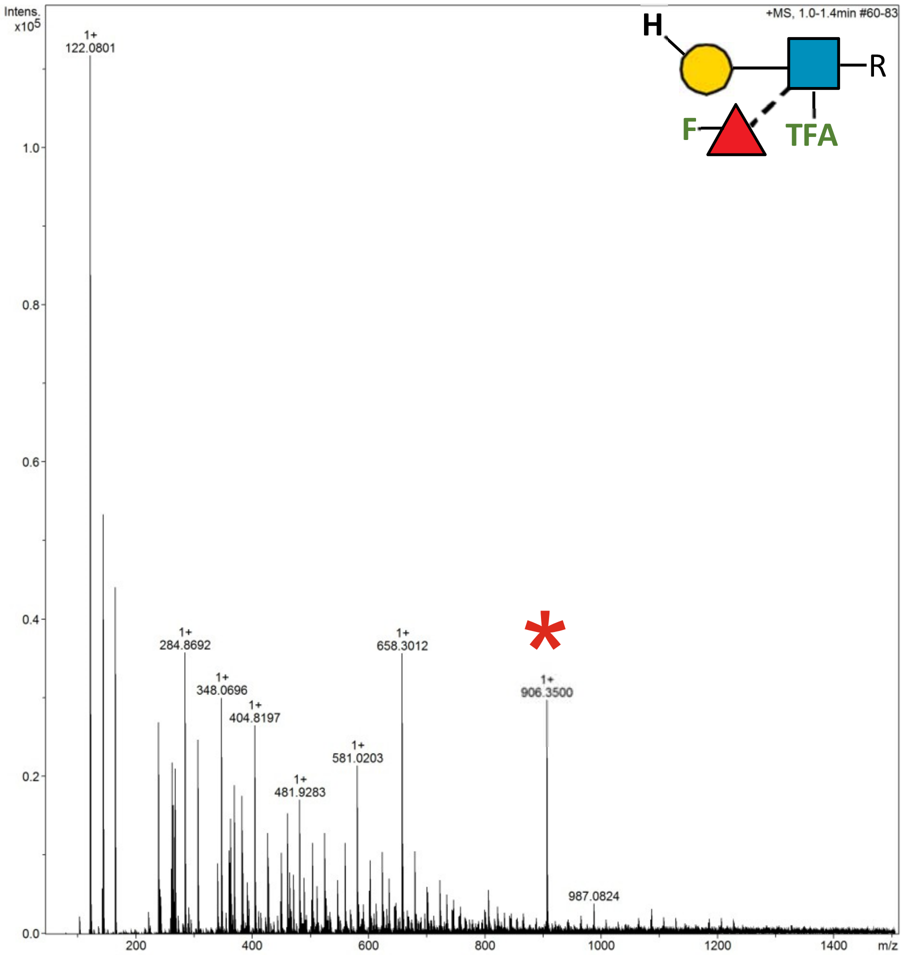

2.18.117 Supplementary Figure 134. HRMS ITag screening assay mass spectrum of synthesis of **6d-Gal β1-4 (3F-Fuc α1-3) 6F-GlcNAc-ITag**

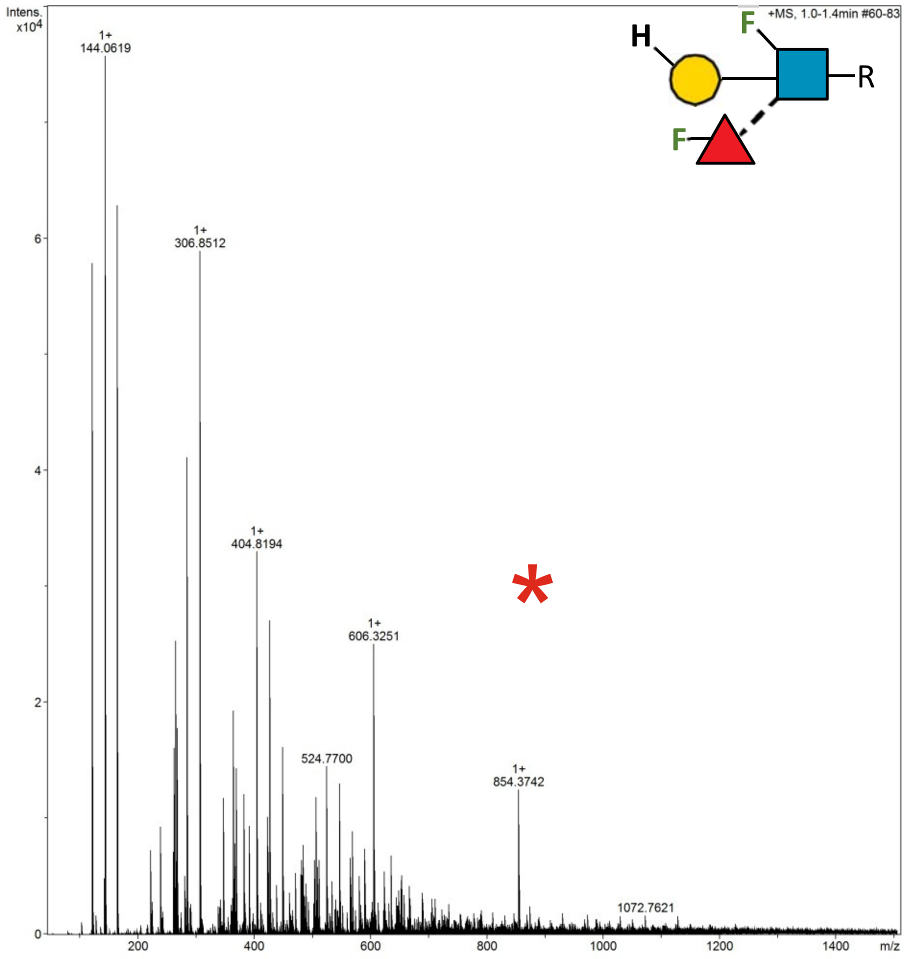

2.18.118 Supplementary Figure 135. HRMS ITag screening assay mass spectrum of synthesis of 6d-Gal β1-4 (3F-Fuc α1-3) 6F-GlcNTFA-ITag

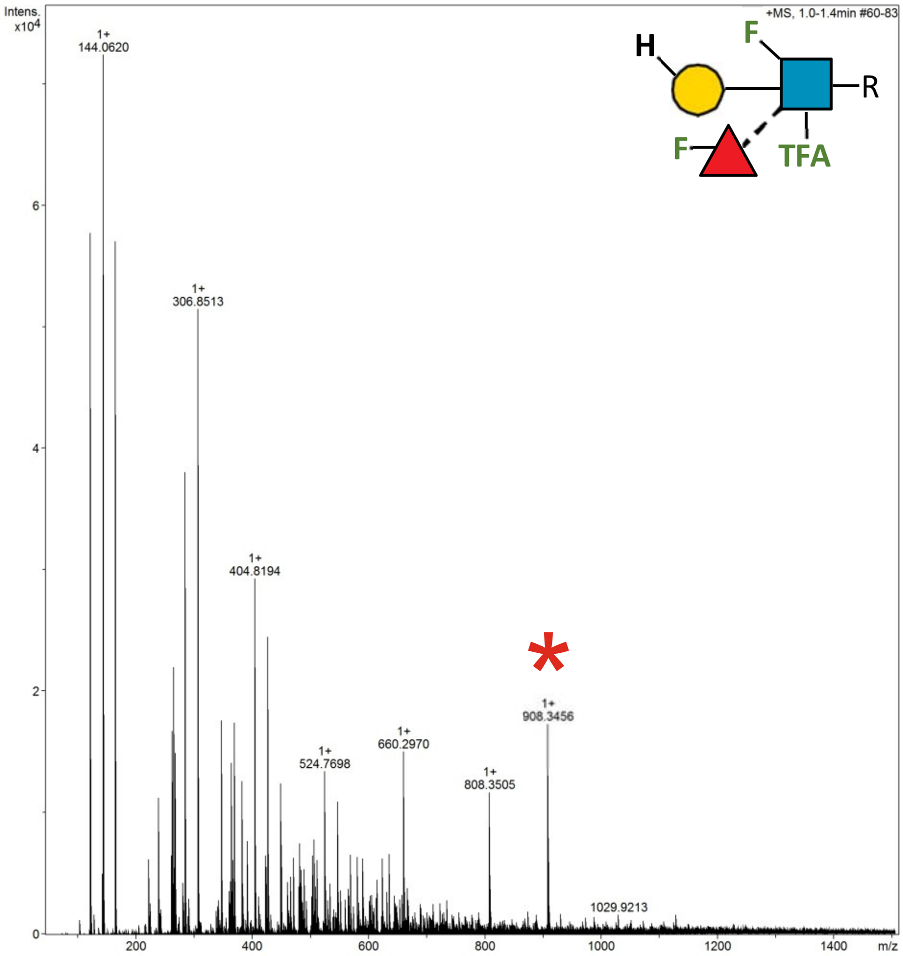

2.18.119 Supplementary Figure 136. HRMS ITag screening assay mass spectrum of synthesis of 6d-Gal β1-4 (3F-Fuc α1-3) 6,6-diFGlcNAc-ITag

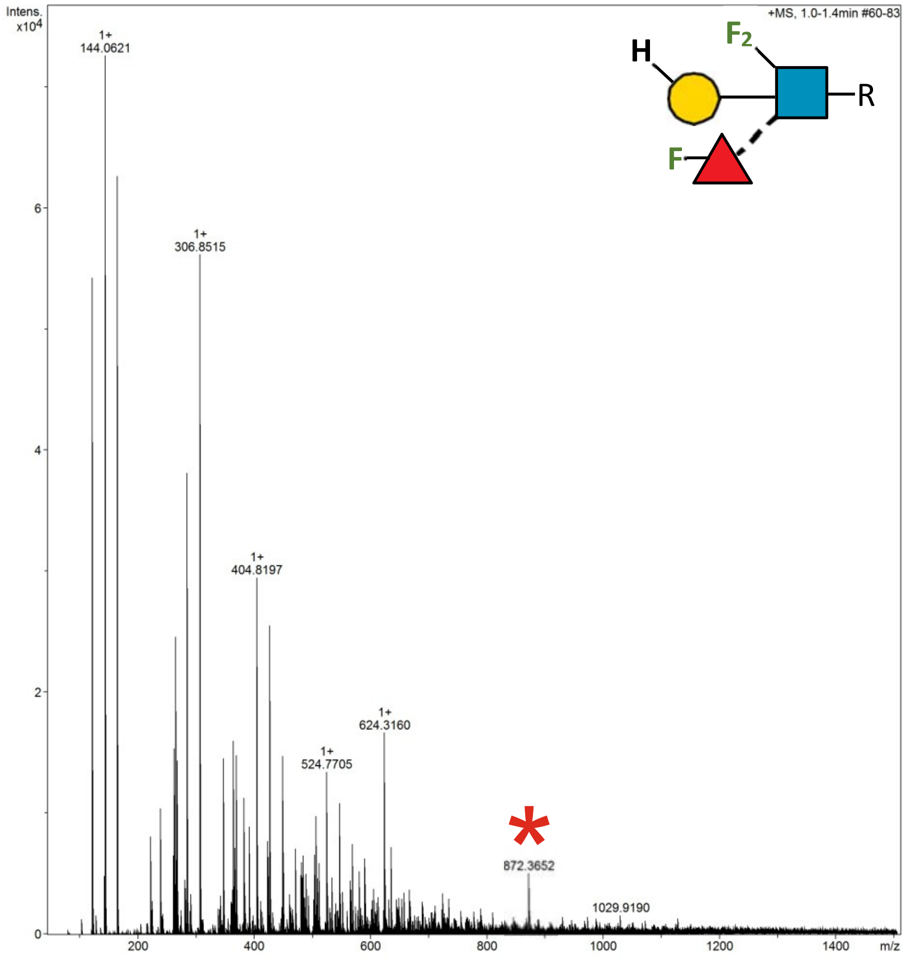

2.18.120 Supplementary Figure 137. HRMS ITag screening assay mass spectrum of synthesis of **6d-Gal β1-4 (3F-Fuc α1-3) 6,6-diFGlcNTFA-ITag**

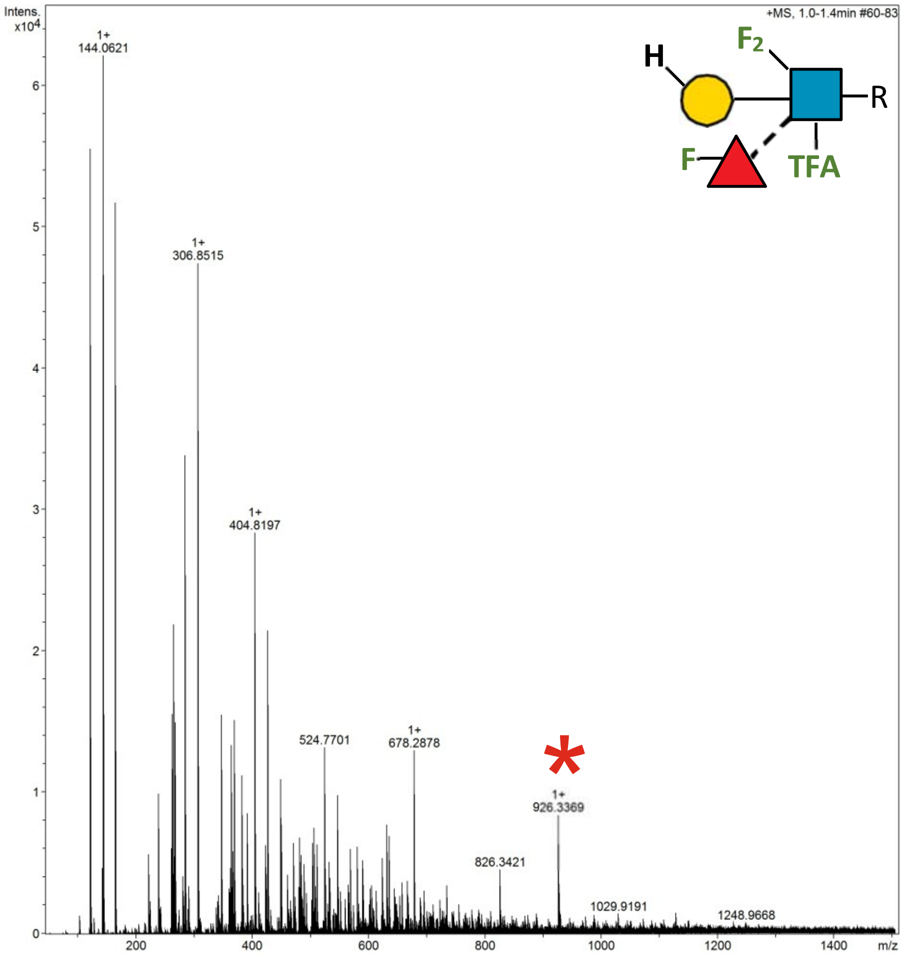

2.18.121 Supplementary Figure 138. HRMS ITag screening assay mass spectrum of synthesis of **Gal β1-4 (6F-Fuc α1-3) GlcNAc-ITag (LeX13)**

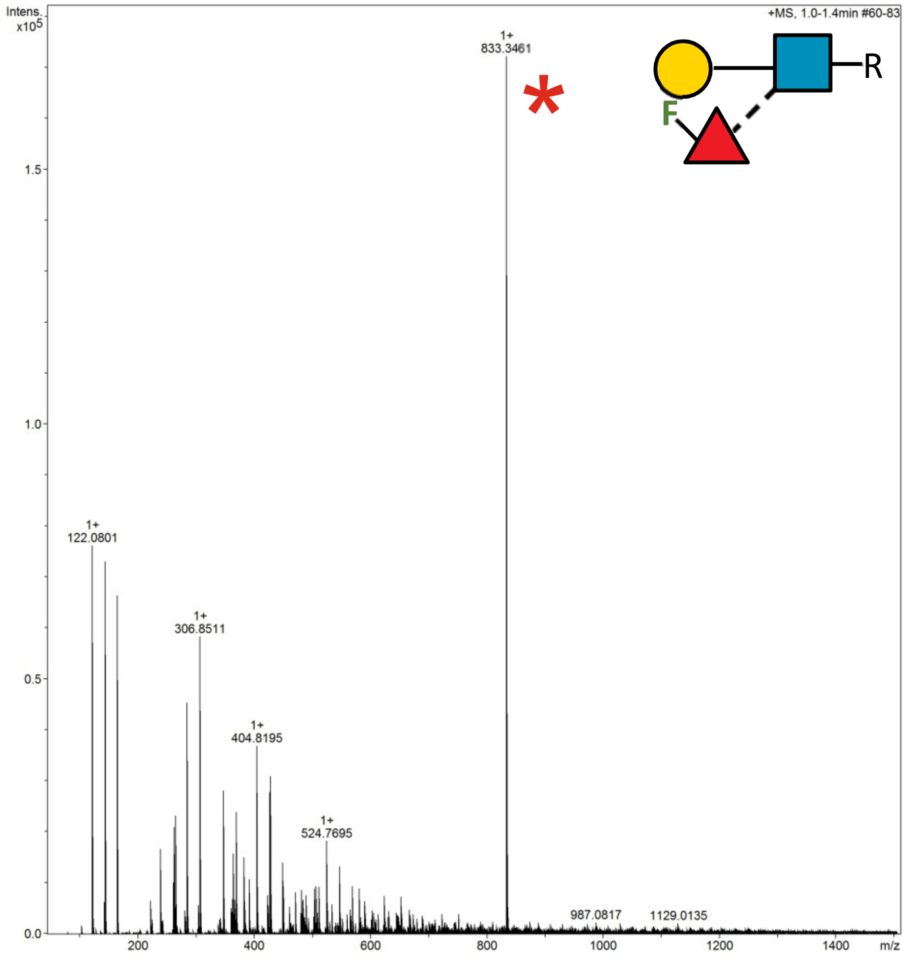

2.18.122 Supplementary Figure 139. HRMS ITag screening assay mass spectrum of synthesis of **Gal β1-4 (6F-Fuc α1-3) GlcNTFA-ITag**

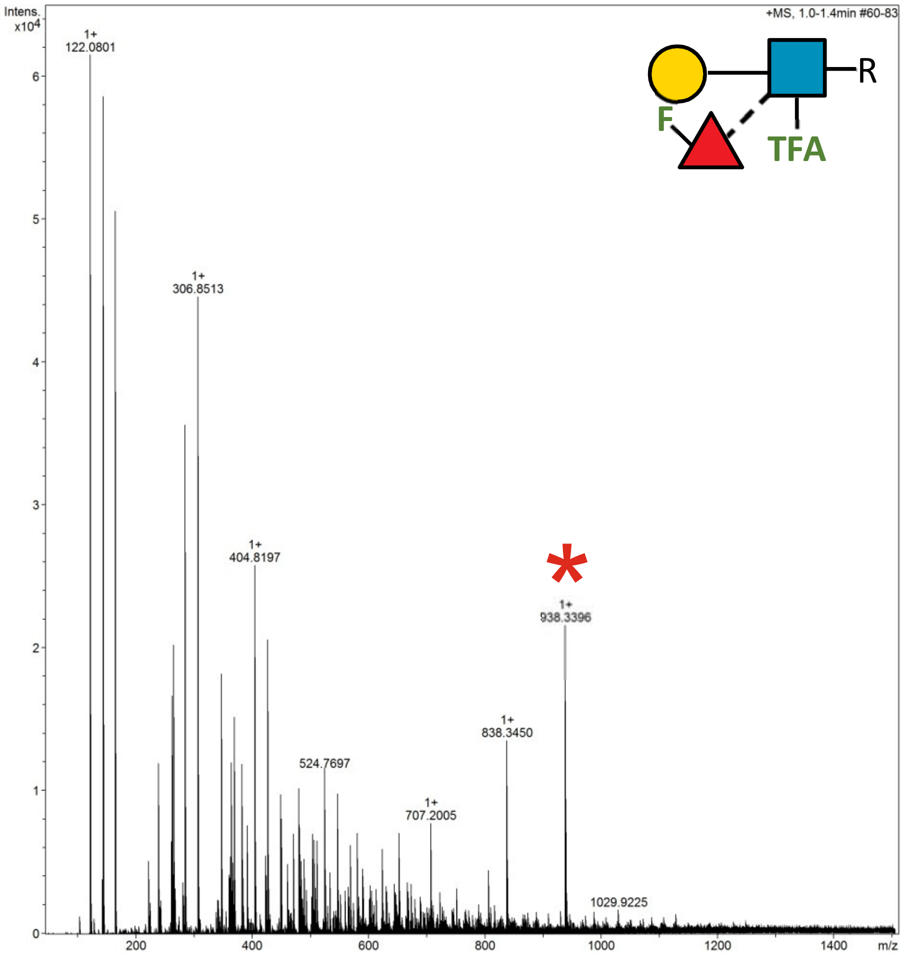

2.18.123 Supplementary Figure 140. HRMS ITag screening assay mass spectrum of synthesis of **Gal β1-4 (6F-Fuc α1-3) 6F-GlcNAc-ITag**

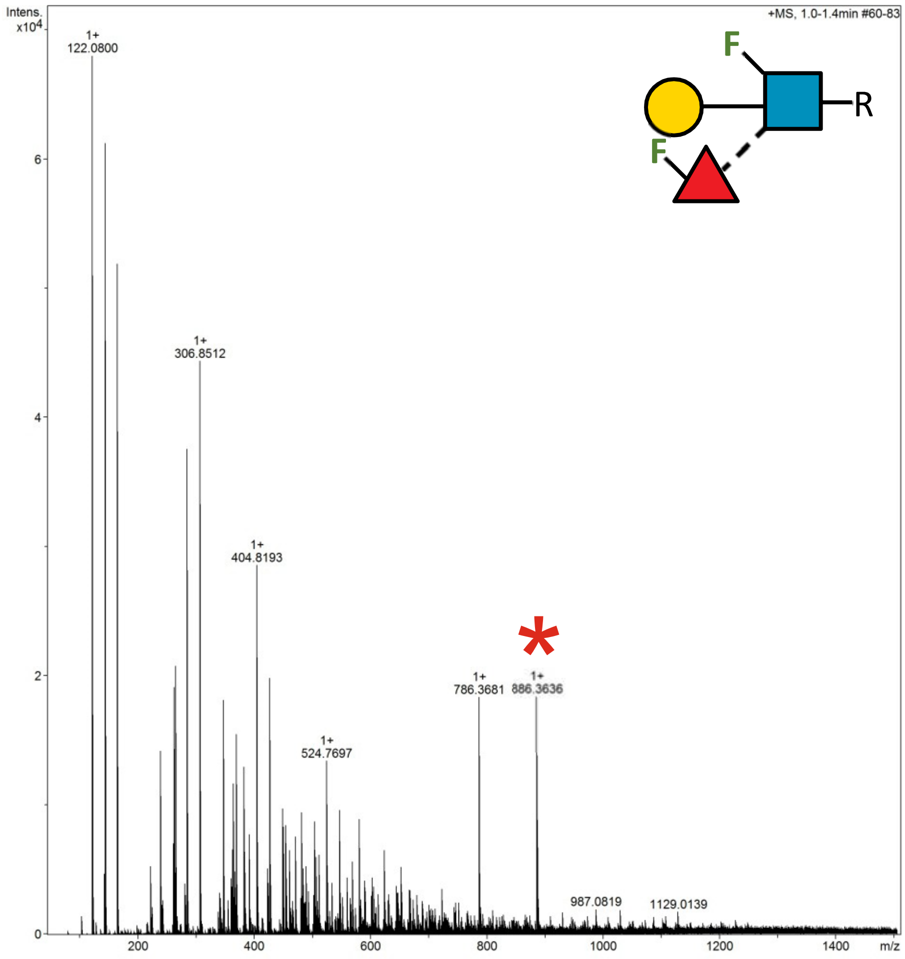

2.18.124 Supplementary Figure 141. HRMS ITag screening assay mass spectrum of synthesis of **Gal β1-4 (6F-Fuc α1-3) 6F-GlcNTFA-ITag (LeX21)**

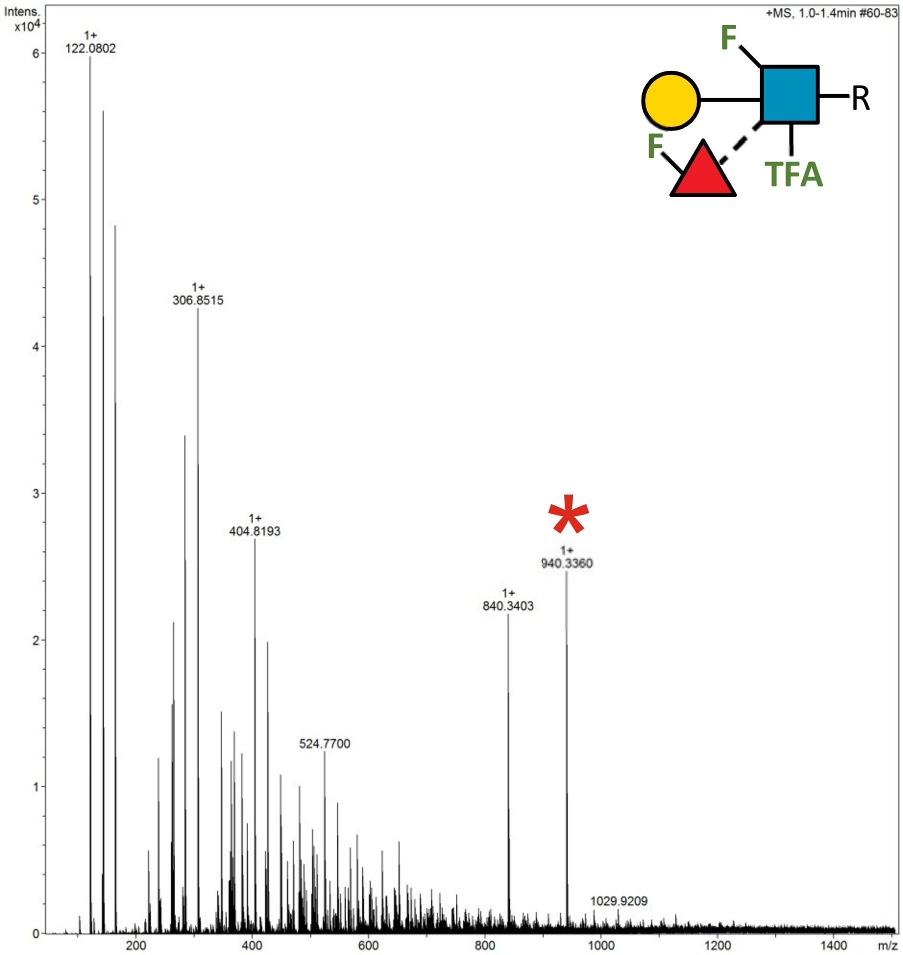

2.18.125 Supplementary Figure 142. HRMS ITag screening assay mass spectrum of synthesis of **Gal β1-4 (6F-Fuc α1-3) 6,6-diFGlcNAc-ITag**

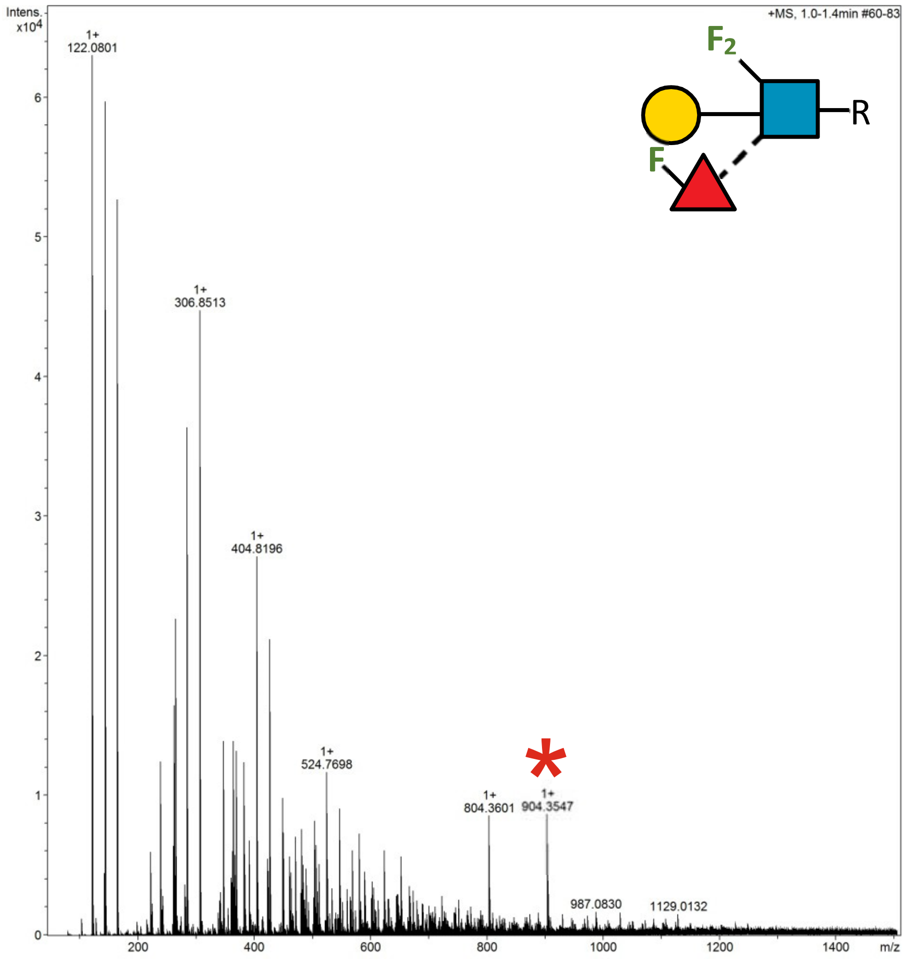

2.18.126 Supplementary Figure 143. HRMS ITag screening assay mass spectrum of synthesis of **Gal β1-4 (6F-Fuc α1-3) 6,6-diFGlcNTFA-ITag**

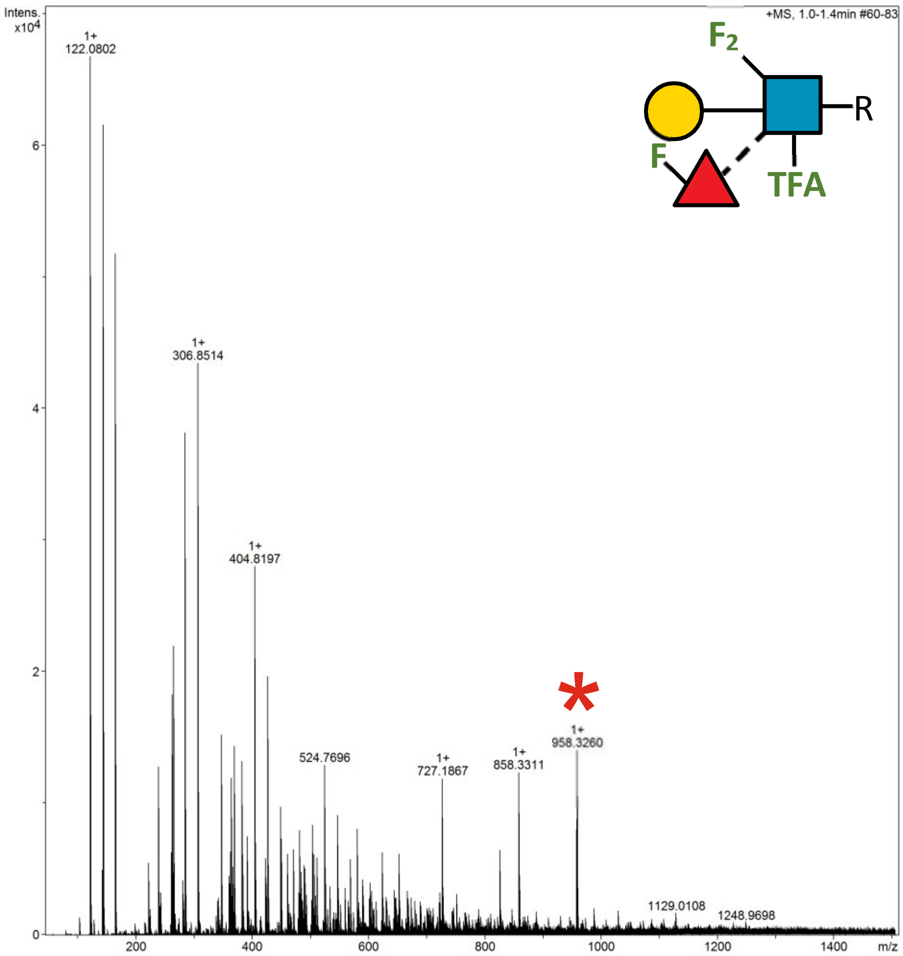

2.18.127 Supplementary Figure 144. HRMS ITag screening assay mass spectrum of synthesis of **3F-Gal β1-4 (6F-Fuc α1-3) GlcNAc-ITag (LeX15)**

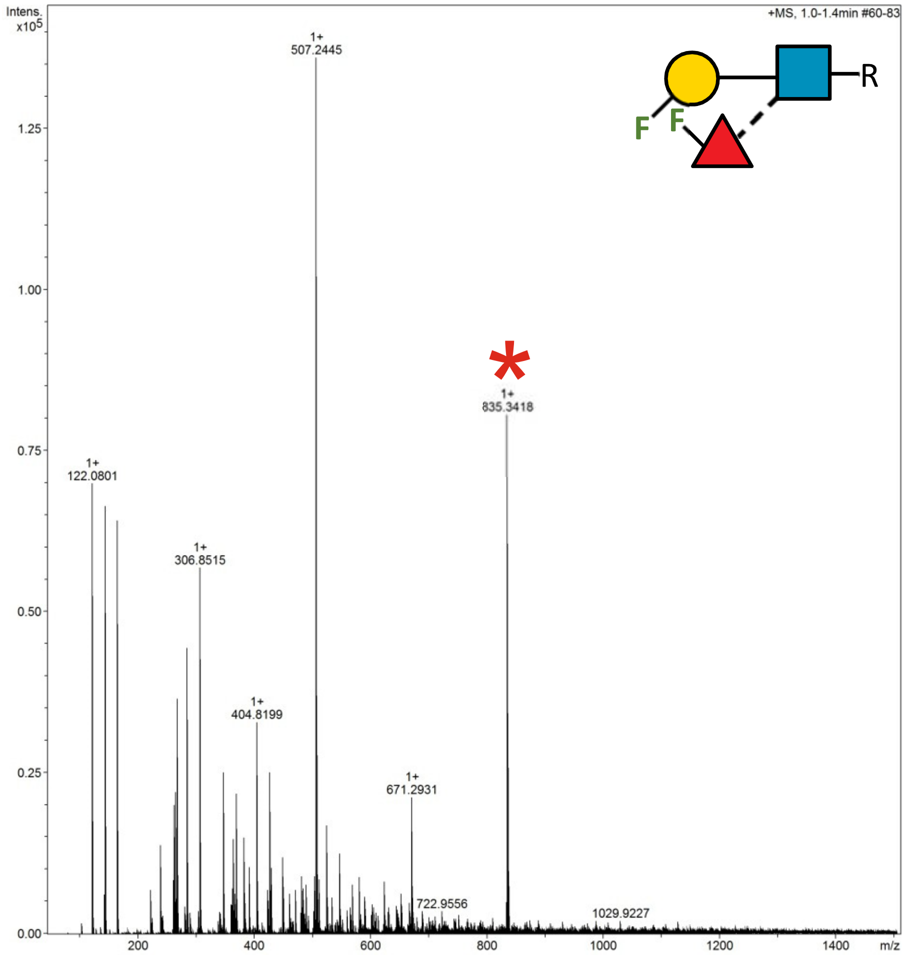

2.18.128 Supplementary Figure 145. HRMS ITag screening assay mass spectrum of synthesis of **3F-Gal β1-4 (6F-Fuc α1-3) GlcNTFA-ITag**

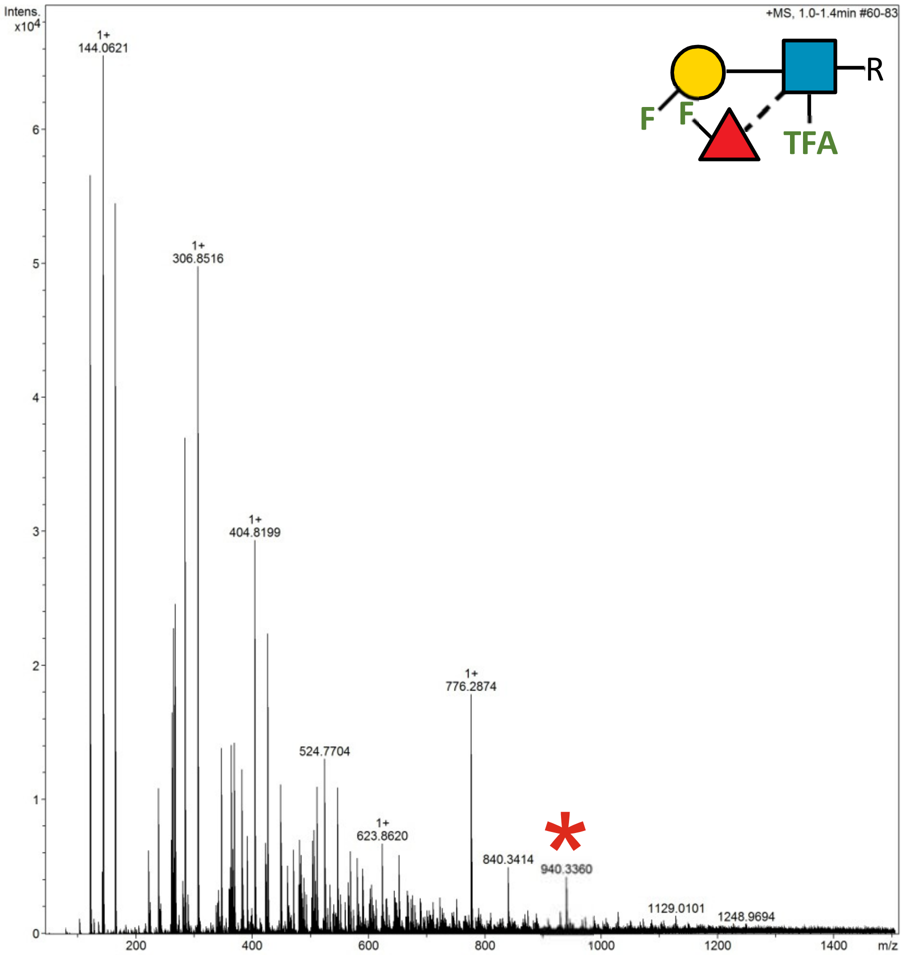

2.18.129 Supplementary Figure 146. HRMS ITag screening assay mass spectrum of synthesis of **3F-Gal β1-4 (6F-Fuc α1-3) 6F-GlcNAc-ITag**

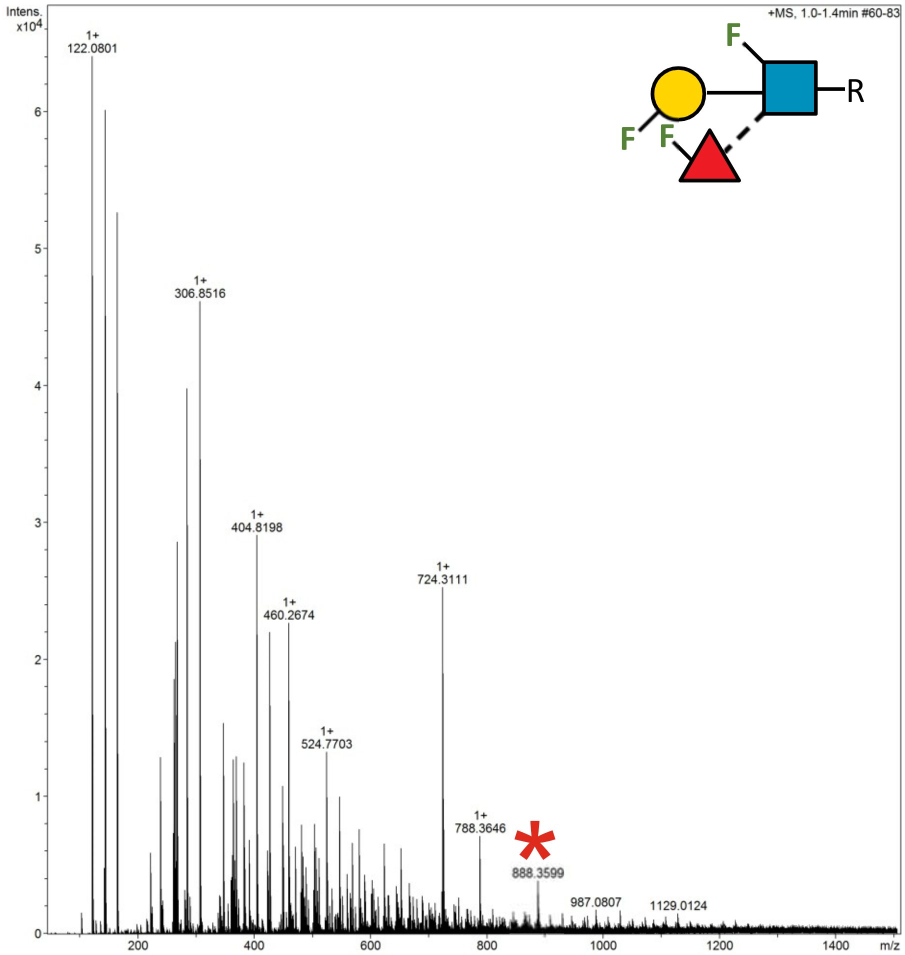

2.18.130 Supplementary Figure 147. HRMS ITag screening assay mass spectrum of synthesis of **3F-Gal β1-4 (6F-Fuc α1-3) 6F-GlcNTFA-ITag**

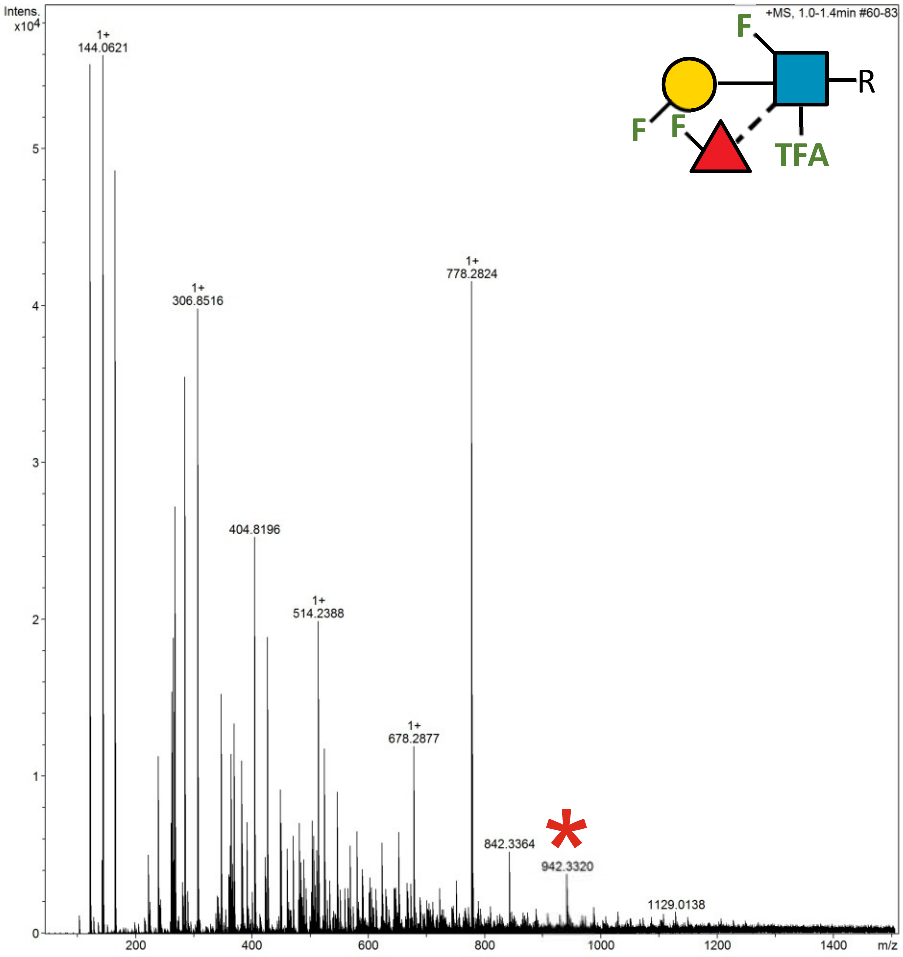

2.18.131 Supplementary Figure 148. HRMS ITag screening assay mass spectrum of synthesis of **3F-Gal β1-4 (6F-Fuc α1-3) 6,6-diFGlcNAc-ITag**

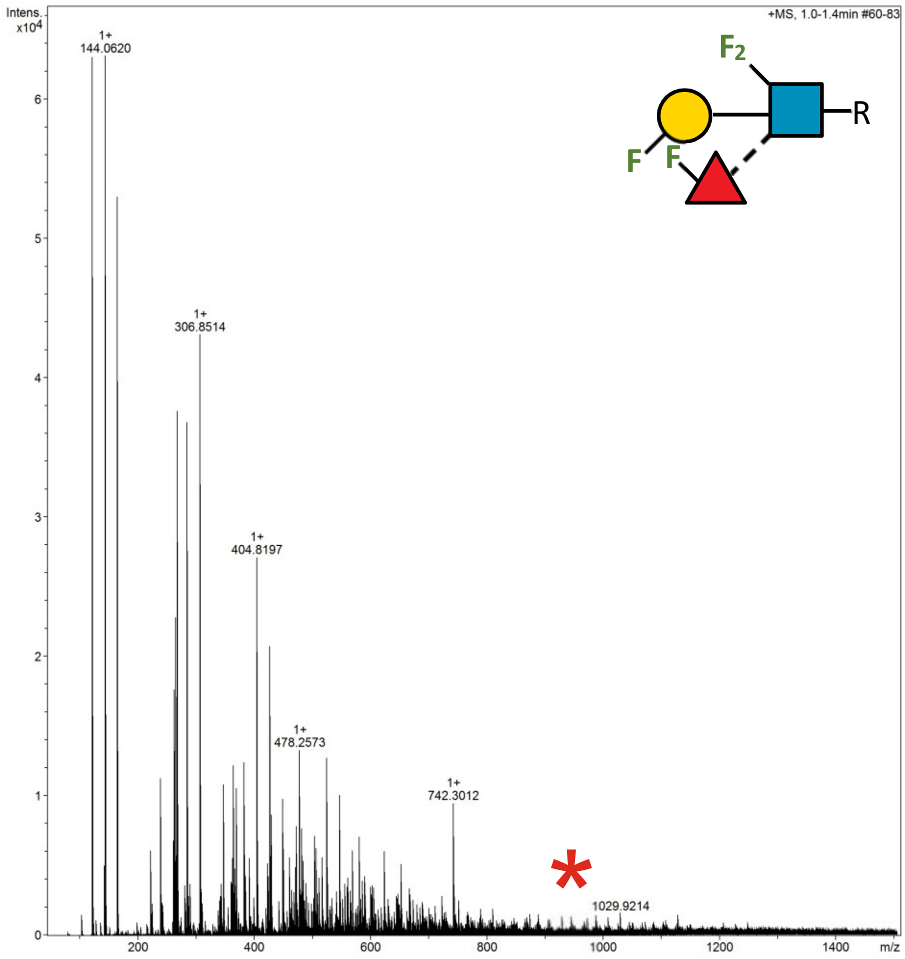

2.18.132 Supplementary Figure 149. HRMS ITag screening assay mass spectrum of synthesis of **3F-Gal β1-4 (6F-Fuc α1-3) 6,6-diFGlcNTFA-ITag**

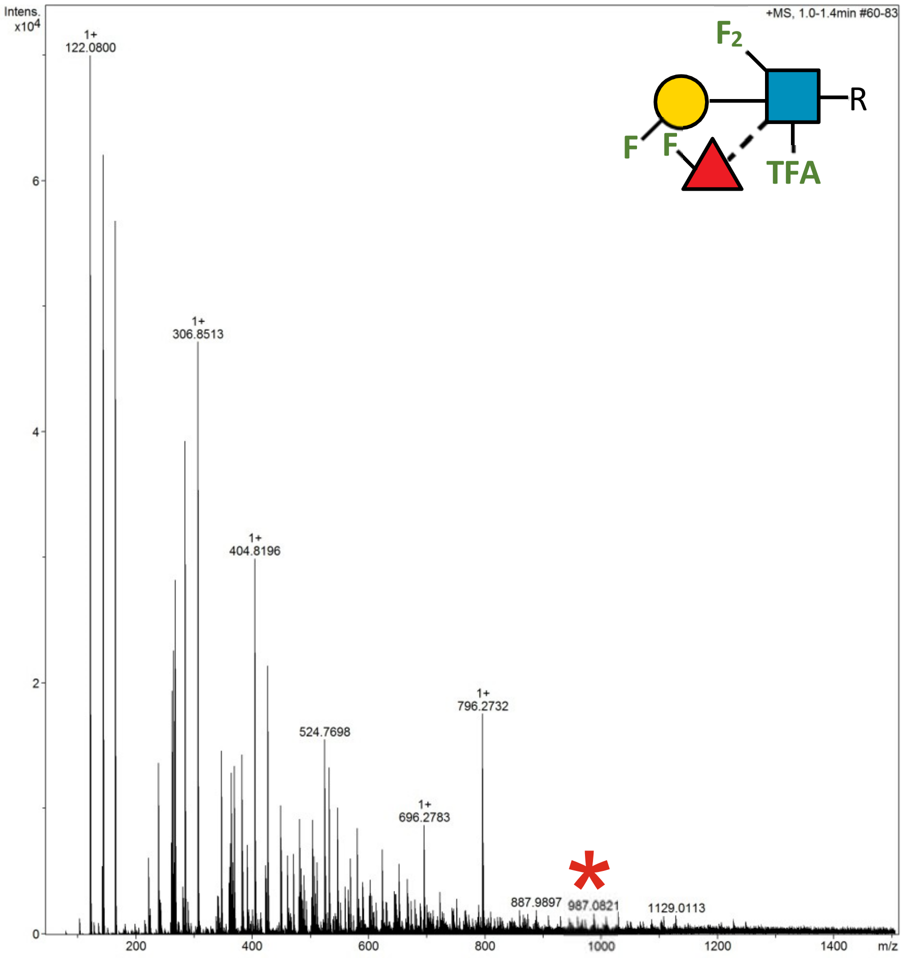

2.18.133 Supplementary Figure 150. HRMS ITag screening assay mass spectrum of synthesis of **4F-Gal β1-4 (6F-Fuc α1-3) GlcNAc-ITag (LeX16)**

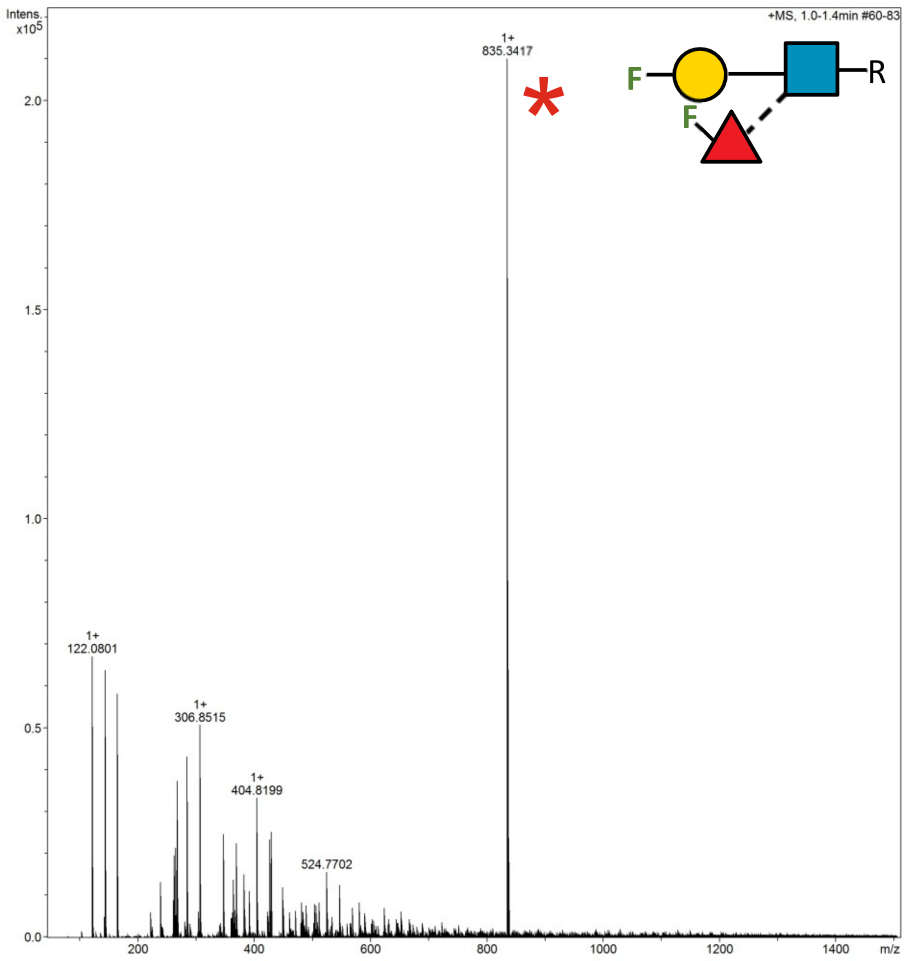

2.18.134 Supplementary Figure 151. HRMS ITag screening assay mass spectrum of synthesis of **4F-Gal β1-4 (6F-Fuc α1-3) GlcNTFA-ITag (LeX24)**

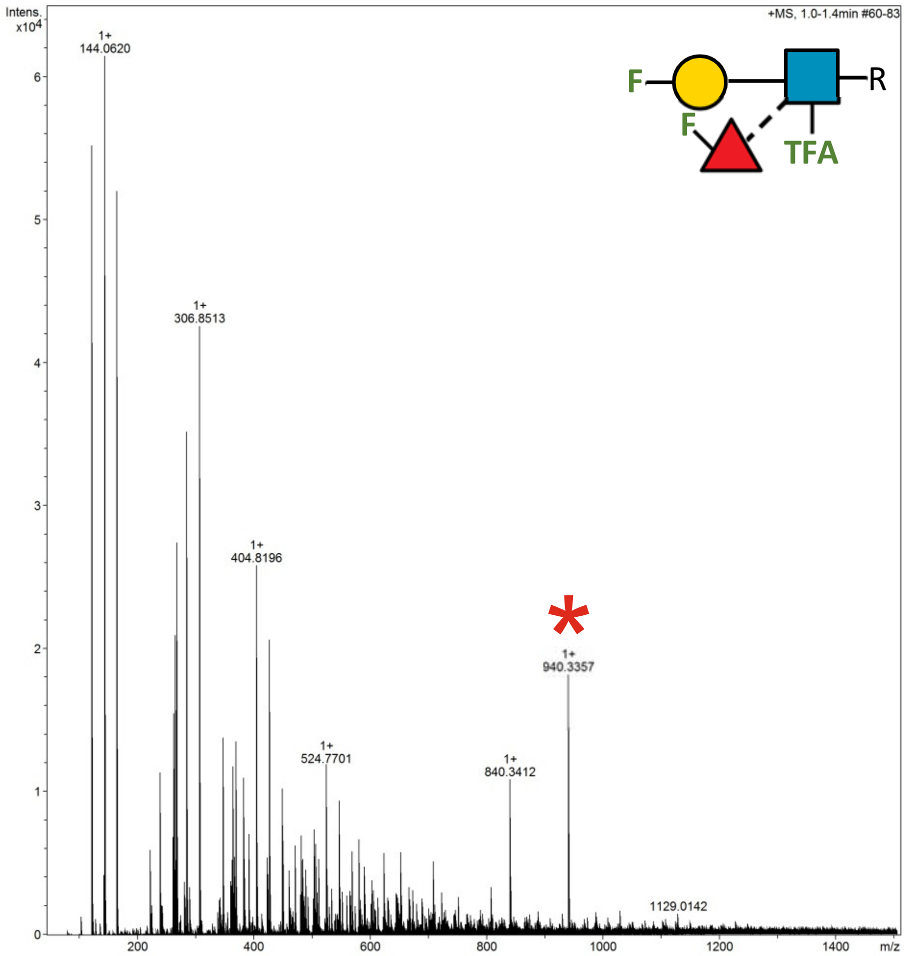

2.18.135 Supplementary Figure 152. HRMS ITag screening assay mass spectrum of synthesis of **4F-Gal β1-4 (6F-Fuc α1-3) 6F-GlcNAc-ITag**

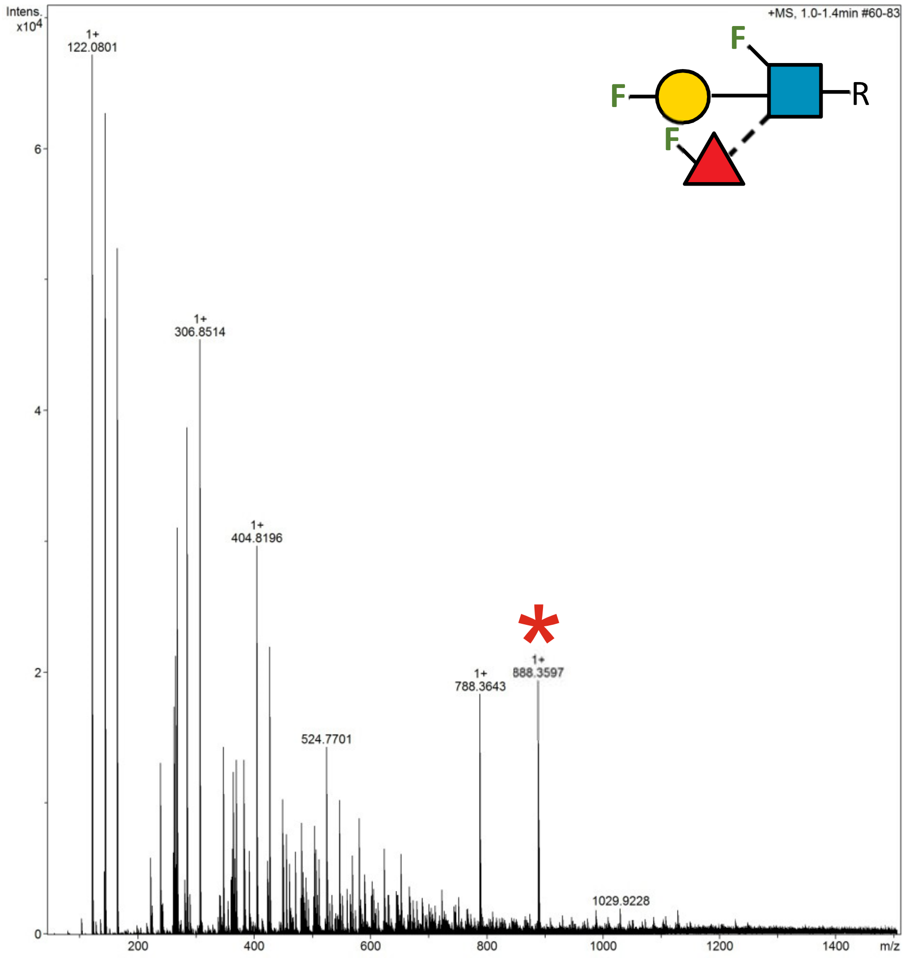

2.18.136 Supplementary Figure 153. HRMS ITag screening assay mass spectrum of synthesis of **4F-Gal β1-4 (6F-Fuc α1-3) 6F-GlcNTFA-ITag**

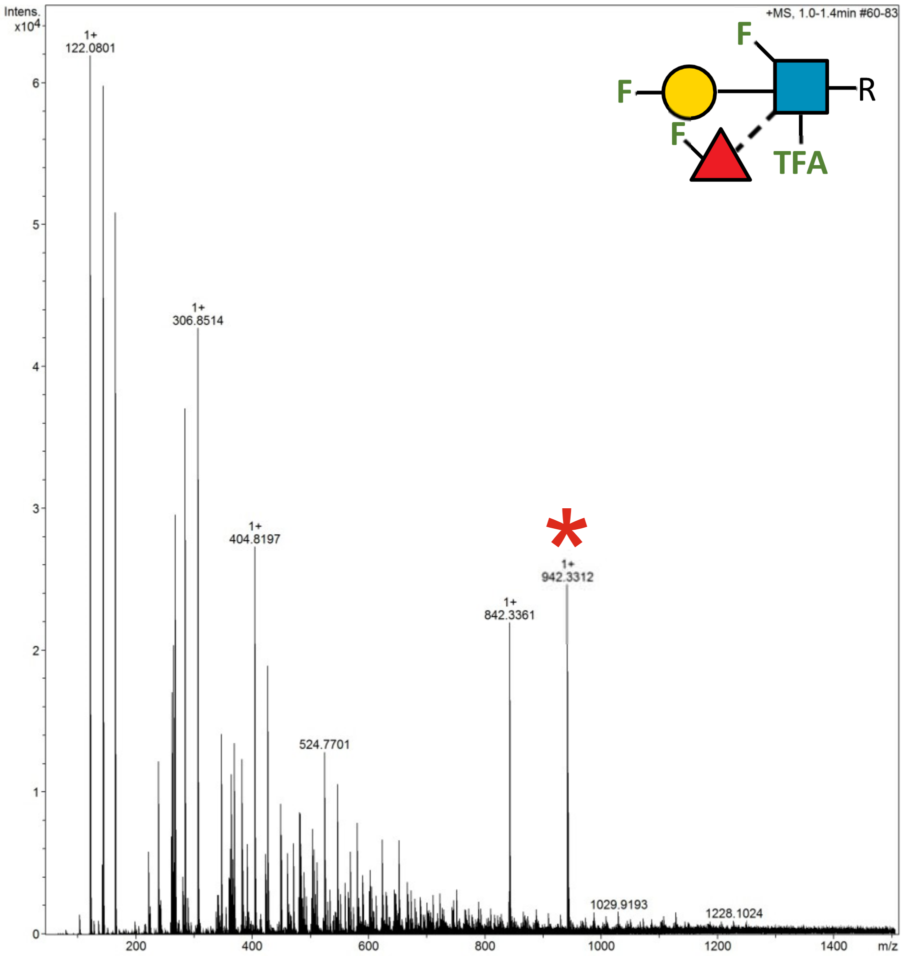

2.18.137 Supplementary Figure 154. HRMS ITag screening assay mass spectrum of synthesis of **4F-Gal β1-4 (6F-Fuc α1-3) 6,6-diFGlcNAc-ITag**

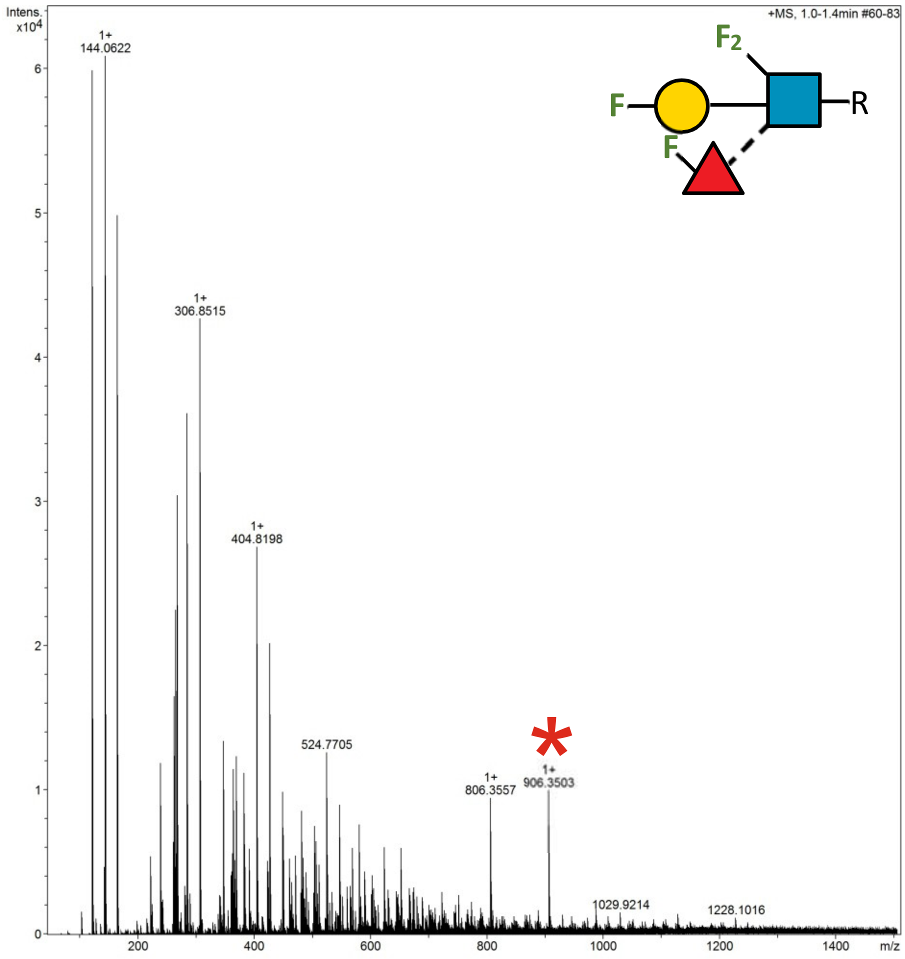

2.18.138 Supplementary Figure 155. HRMS ITag screening assay mass spectrum of synthesis of **4F-Gal β1-4 (6F-Fuc α1-3) 6,6-diFGlcNTFA-ITag**

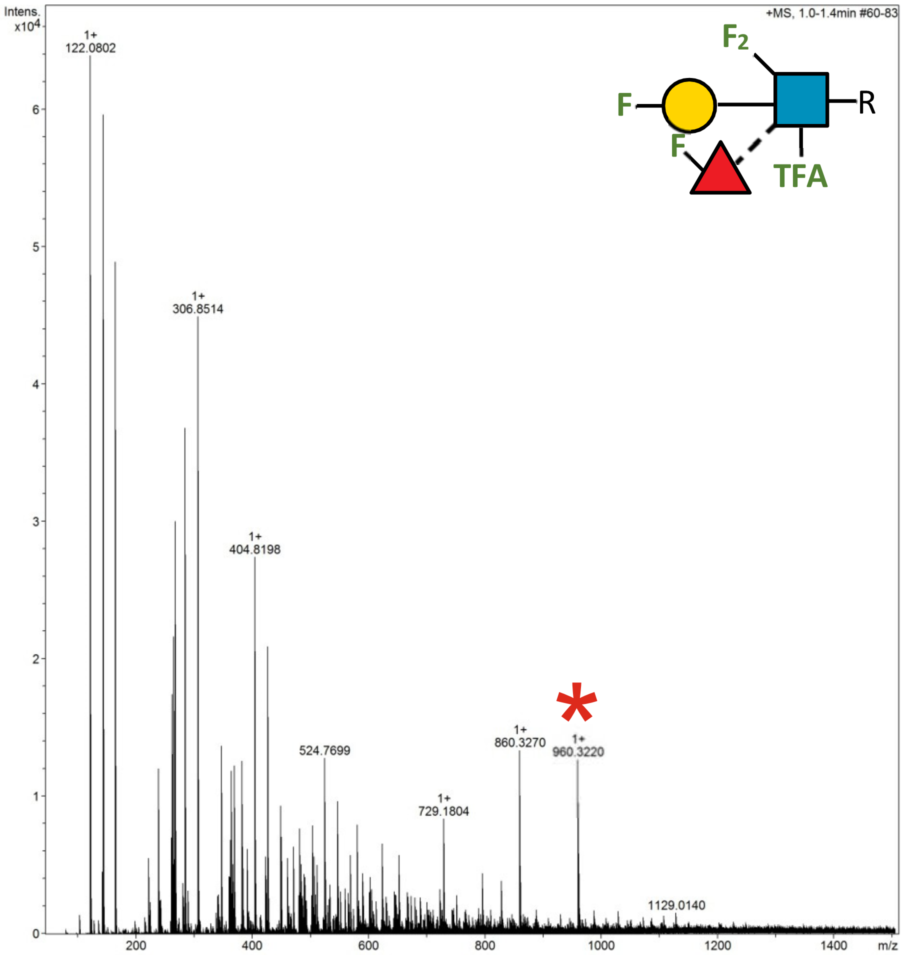

2.18.139 Supplementary Figure 156. HRMS ITag screening assay mass spectrum of synthesis of **6F-Gal β1-4 (6F-Fuc α1-3) GlcNAc-ITag**

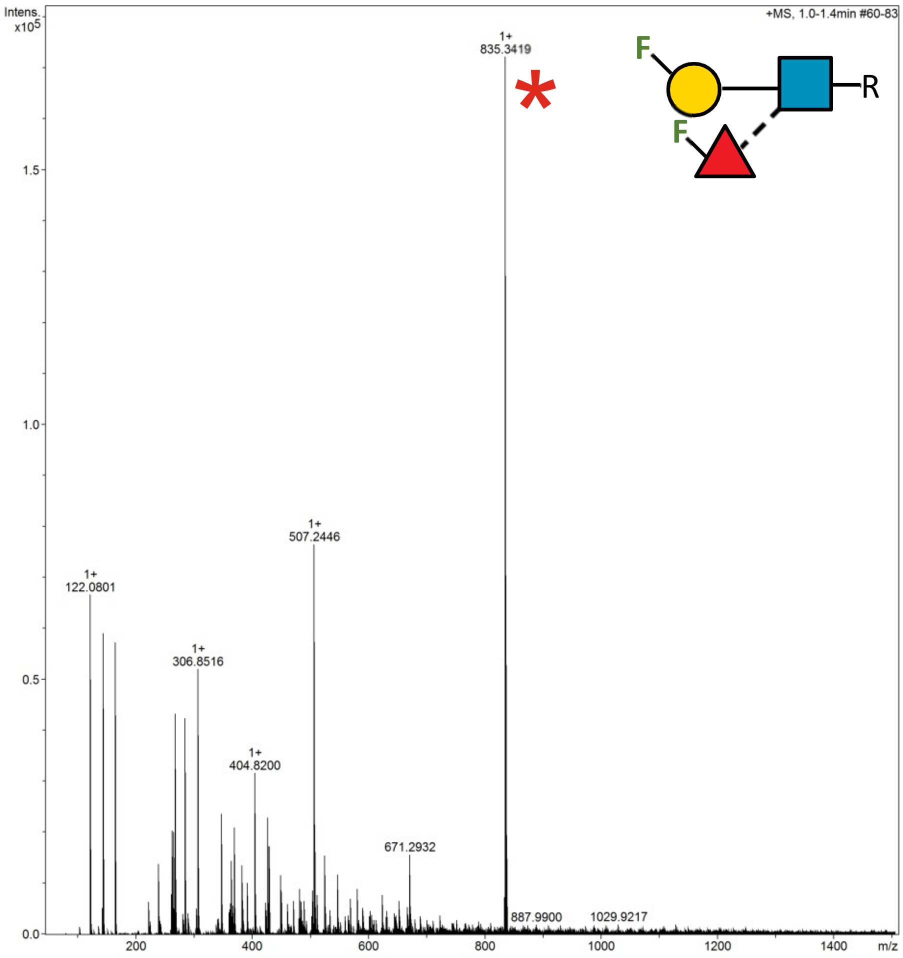

2.18.140 Supplementary Figure 157. HRMS ITag screening assay mass spectrum of synthesis of **6F-Gal β1-4 (6F-Fuc α1-3) GlcNTFA-ITag**

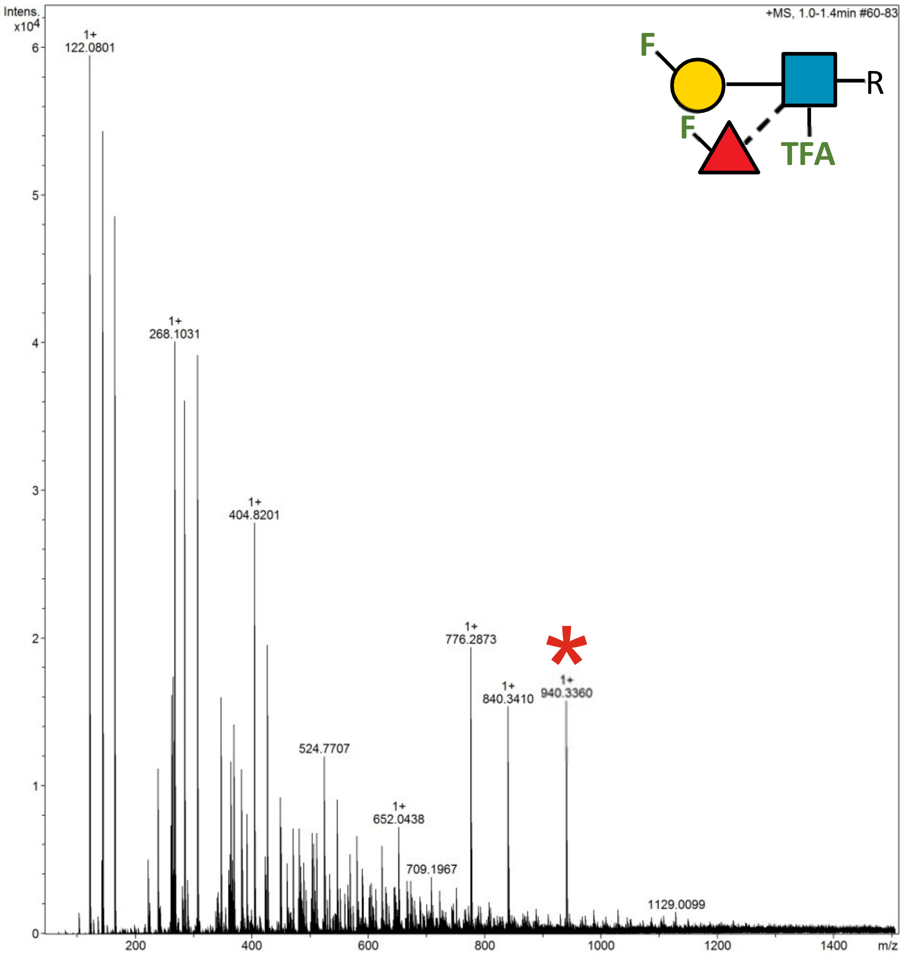

2.18.141 Supplementary Figure 158. HRMS ITag screening assay mass spectrum of synthesis of **6F-Gal β1-4 (6F-Fuc α1-3) 6F-GlcNAc-ITag**

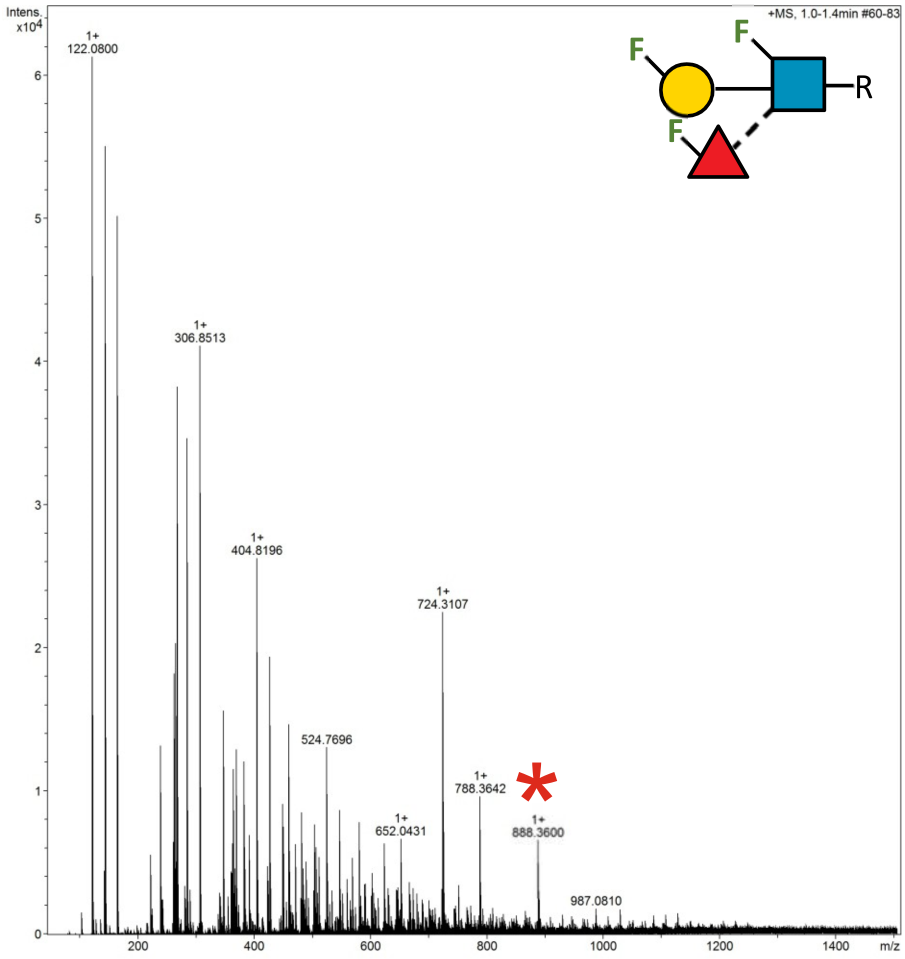

2.18.142 Supplementary Figure 159. HRMS ITag screening assay mass spectrum of synthesis of **6F-Gal β1-4 (6F-Fuc α1-3) 6F-GlcNTFA-ITag**

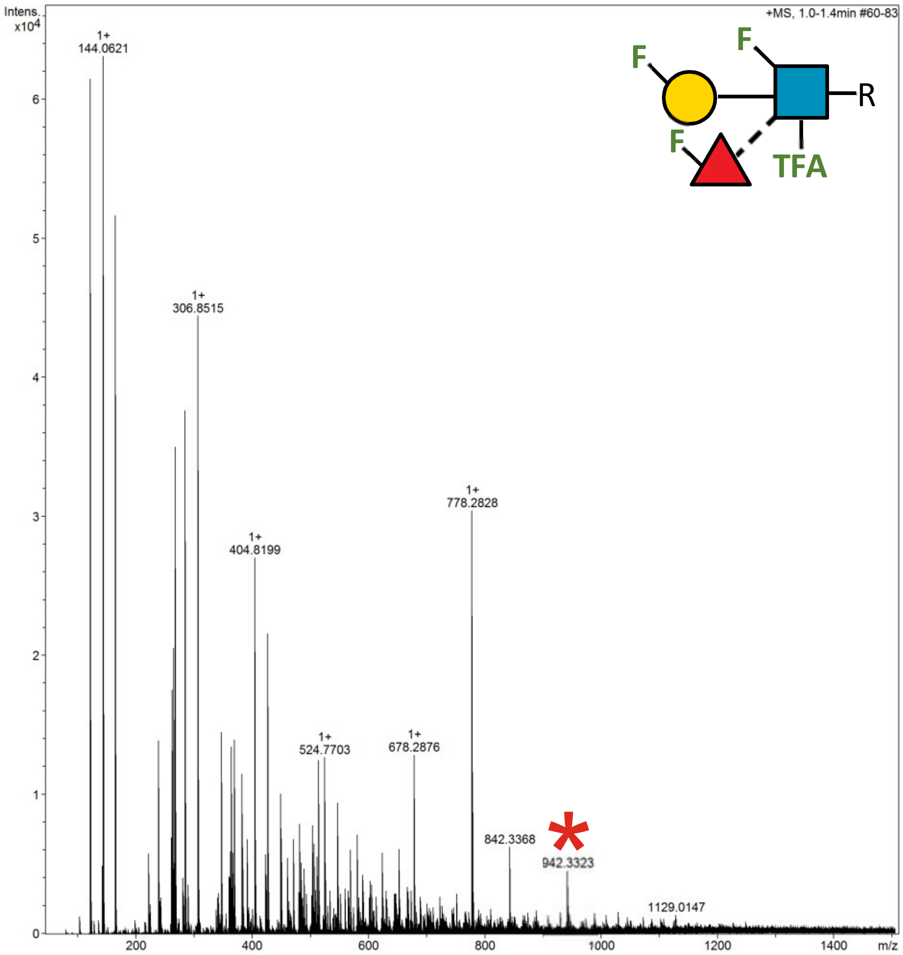

2.18.143 Supplementary Figure 160. HRMS ITag screening assay mass spectrum of synthesis of **6F-Gal β1-4 (6F-Fuc α1-3) 6,6-diFGlcNAc-ITag**

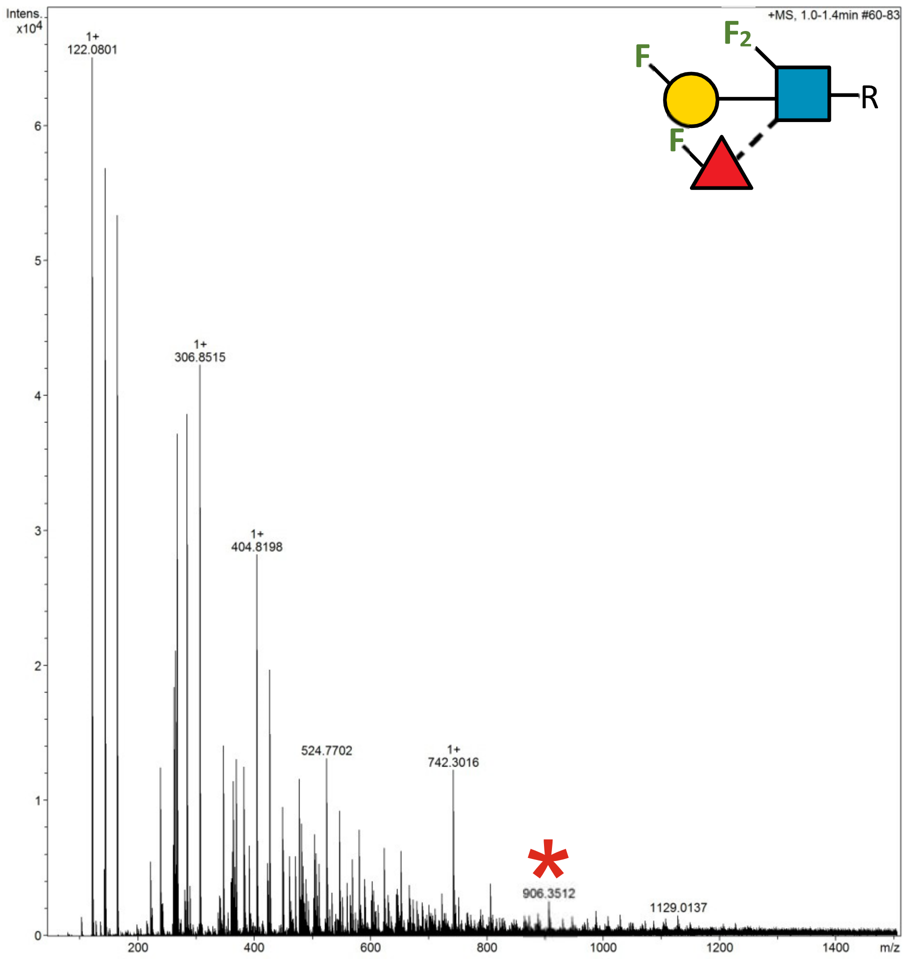

2.18.144 Supplementary Figure 161. HRMS ITag screening assay mass spectrum of synthesis of **6F-Gal β1-4 (6F-Fuc α1-3) 6,6-diFGlcNTFA-ITag**

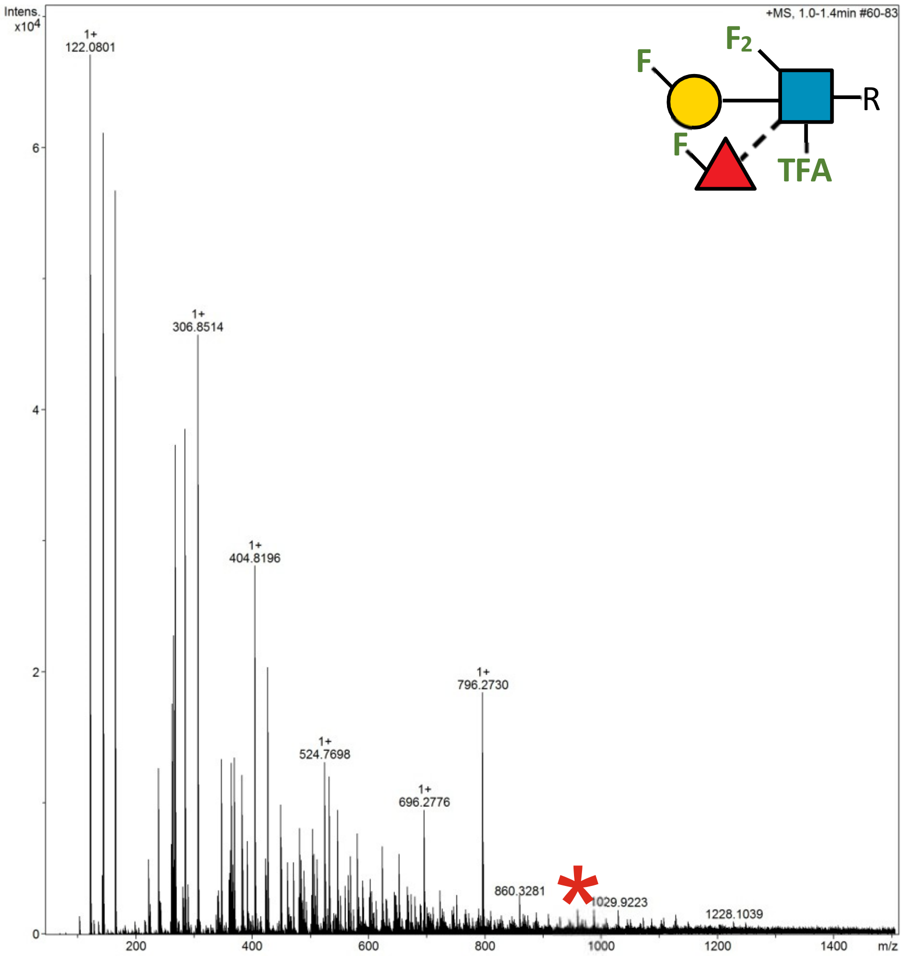

2.18.145 Supplementary Figure 162. HRMS ITag screening assay mass spectrum of synthesis of **6d-Gal β1-4 (6F-Fuc α1-3) GlcNAc-ITag**

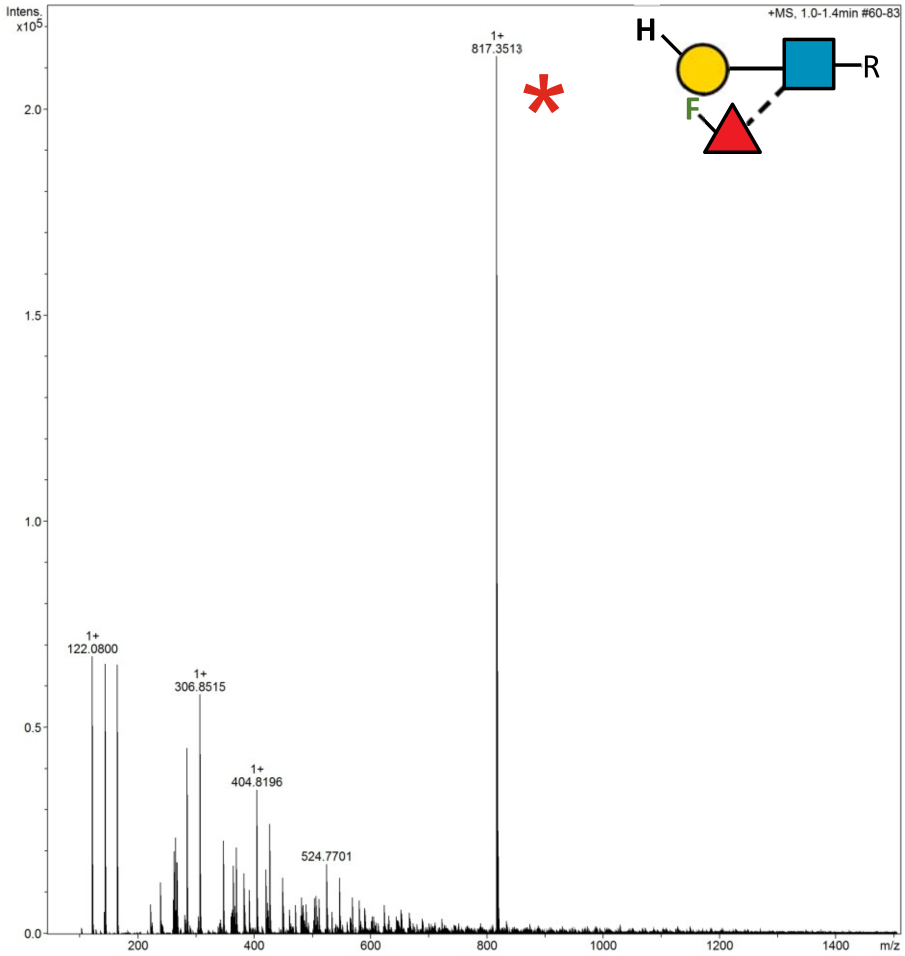

2.18.146 Supplementary Figure 163. HRMS ITag screening assay mass spectrum of synthesis of **6d-Gal β1-4 (6F-Fuc α1-3) GlcNTFA-ITag**

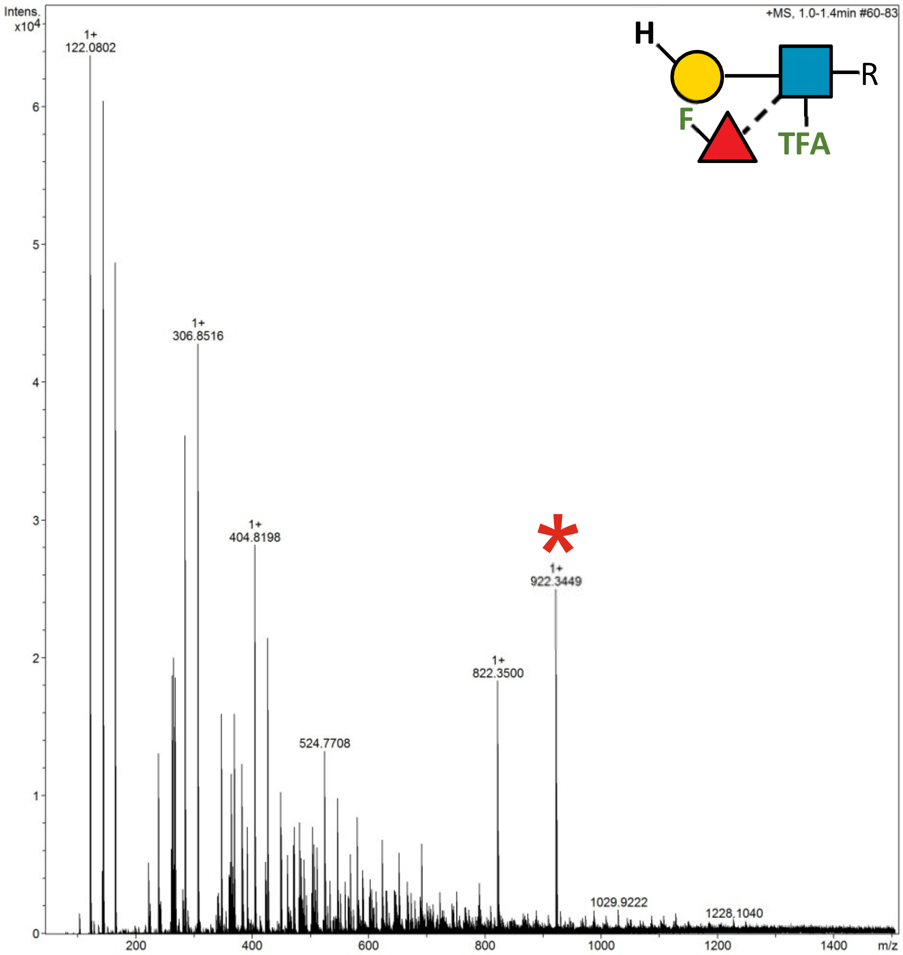

2.18.147 Supplementary Figure 164. HRMS ITag screening assay mass spectrum of synthesis of **6d-Gal β1-4 (6F-Fuc α1-3) 6F-GlcNAc-ITag**

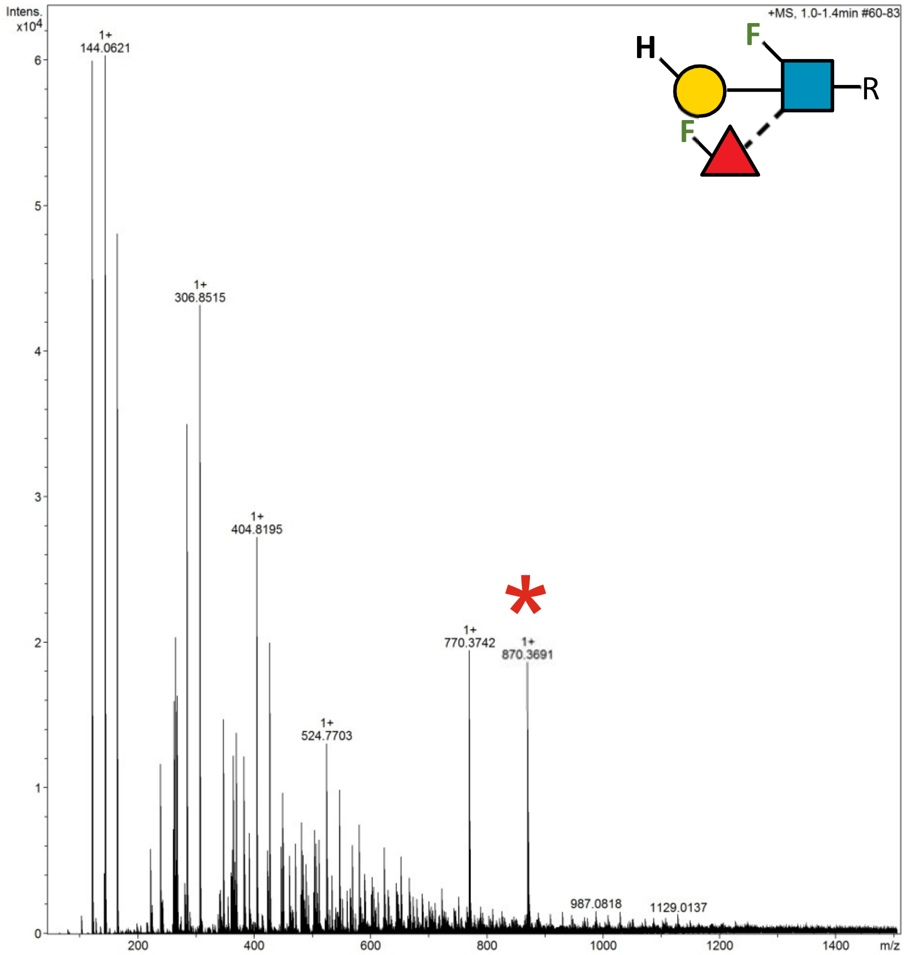

2.18.148 Supplementary Figure 165. HRMS ITag screening assay mass spectrum of synthesis of **6d-Gal β1-4 (6F-Fuc α1-3) 6F-GlcNTFA-ITag**

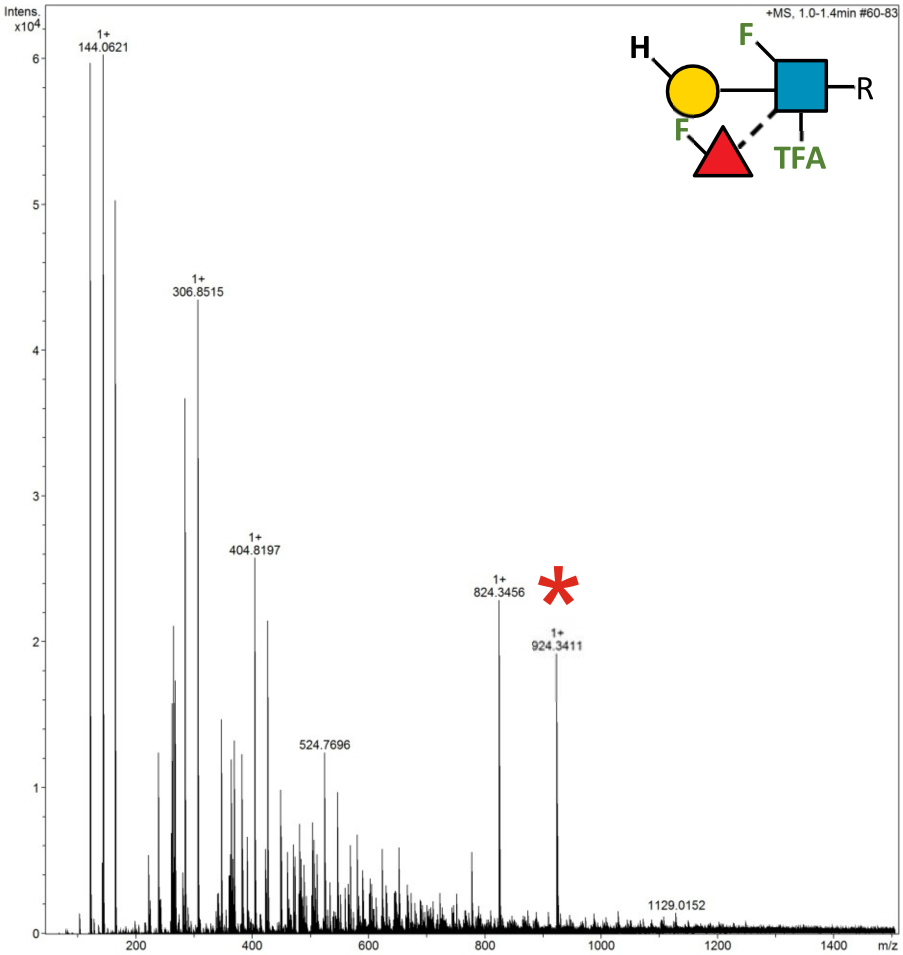

2.18.149 Supplementary Figure 166. HRMS ITag screening assay mass spectrum of synthesis of **6d-Gal β1-4 (6F-Fuc α1-3) 6,6-diFGlcNAc-ITag**

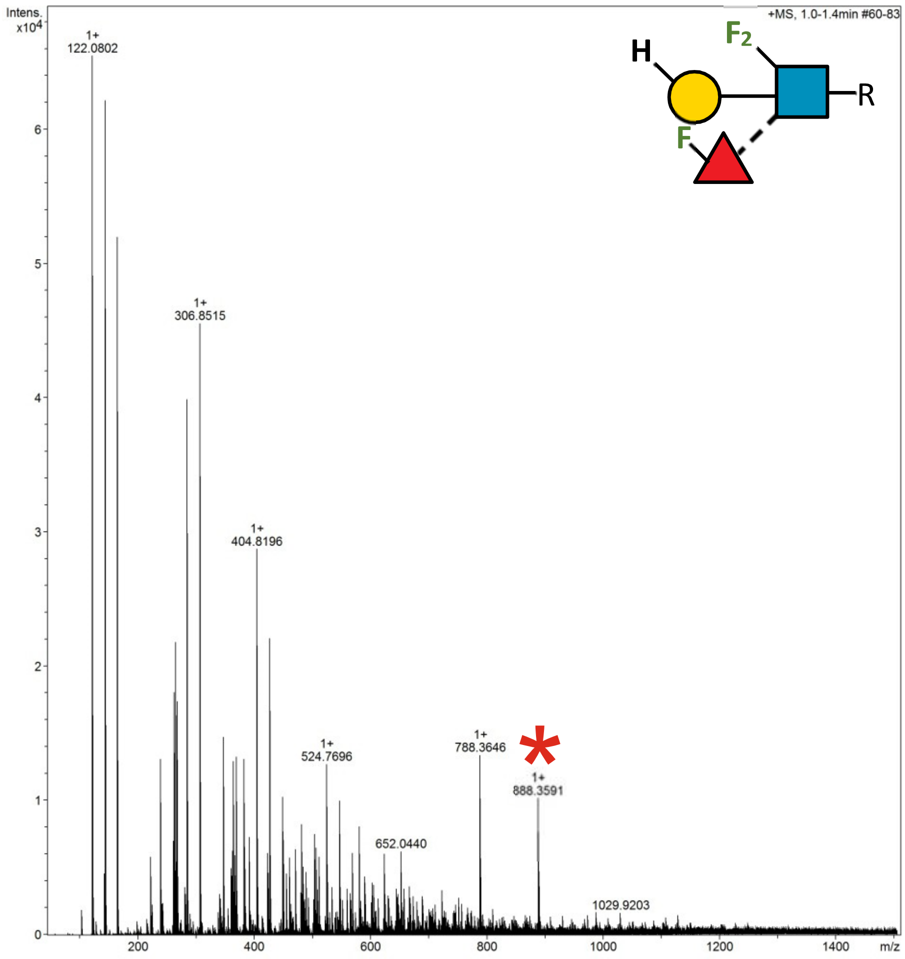

2.18.150 Supplementary Figure 167. HRMS ITag screening assay mass spectrum of synthesis of **6d-Gal β1-4 (6F-Fuc α1-3) 6,6-diFGlcNTFA-ITag**

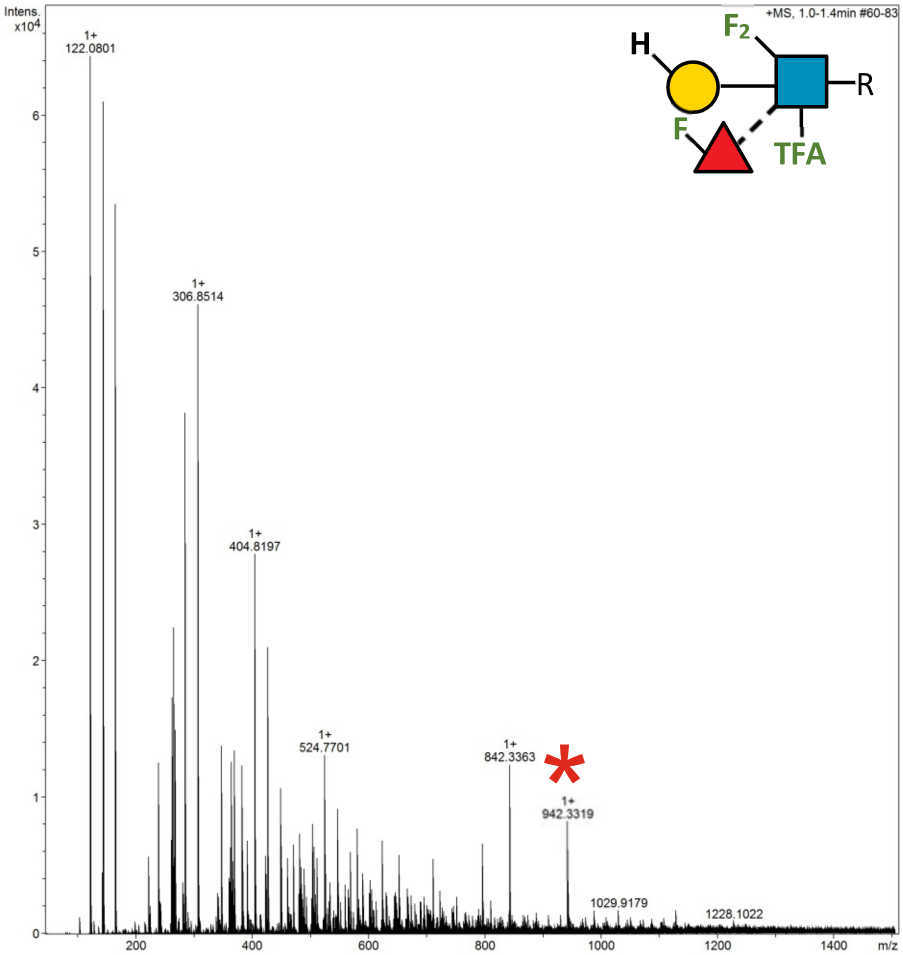

2.18.151 Supplementary Figure 168. HRMS ITag screening assay mass spectrum of synthesis of **Gal β1-4 (Ara α1-3) GlcNAc-ITag (LeX14)**

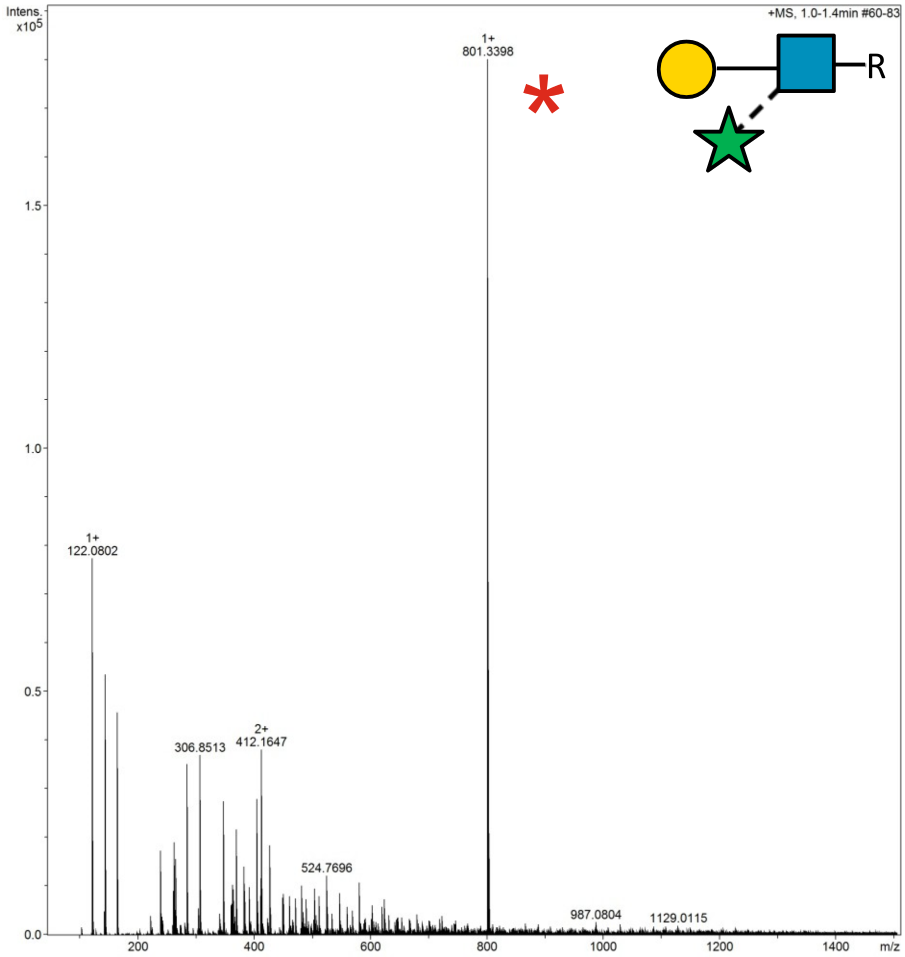

2.18.152 Supplementary Figure 169. HRMS ITag screening assay mass spectrum of synthesis of **Gal β1-4 (Ara α1-3) GlcNTFA-ITag**

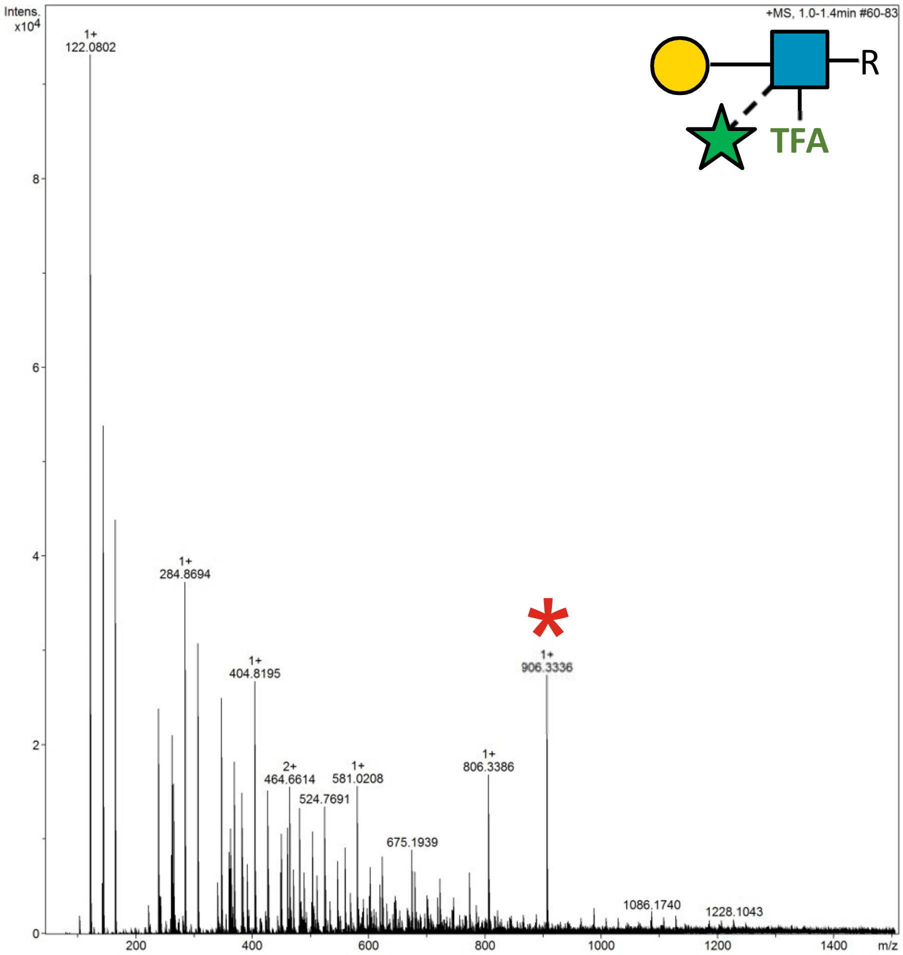

2.18.153 Supplementary Figure 170. HRMS ITag screening assay mass spectrum of synthesis of **Gal β1-4 (Ara α1-3) 6F-GlcNAc-ITag**

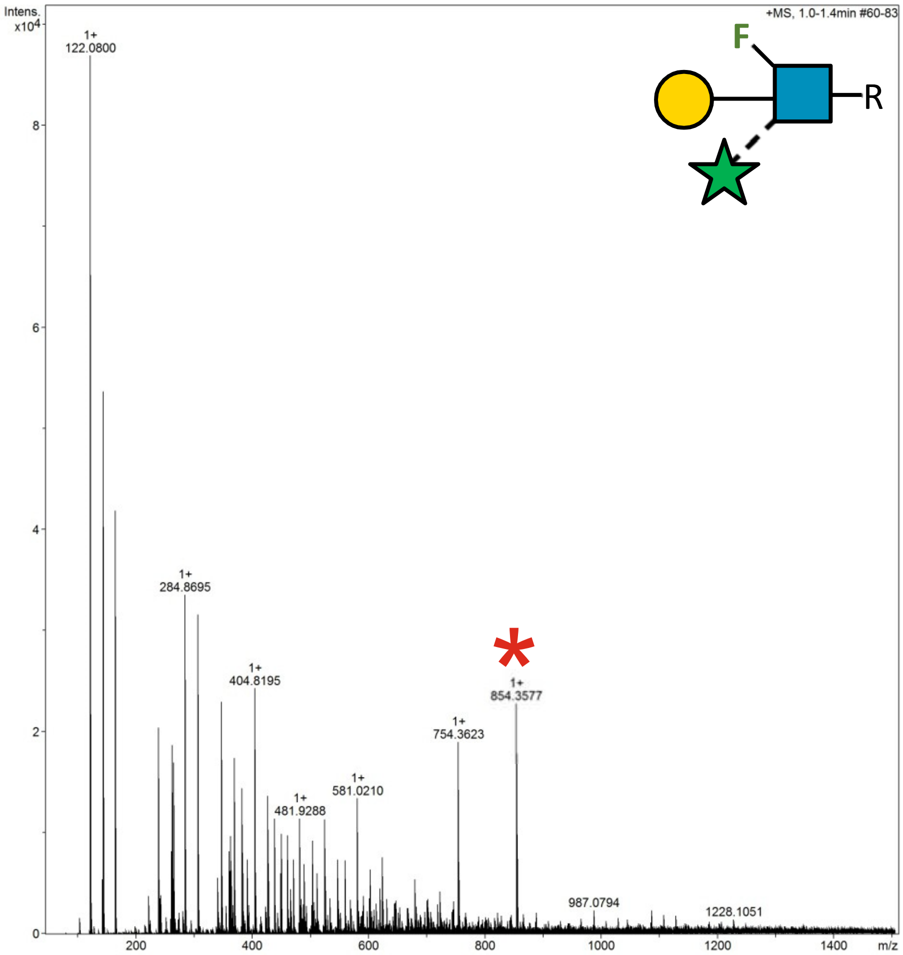

2.18.154 Supplementary Figure 171. HRMS ITag screening assay mass spectrum of synthesis of **Gal β1-4 (Ara α1-3) 6F-GlcNTFA-ITag**

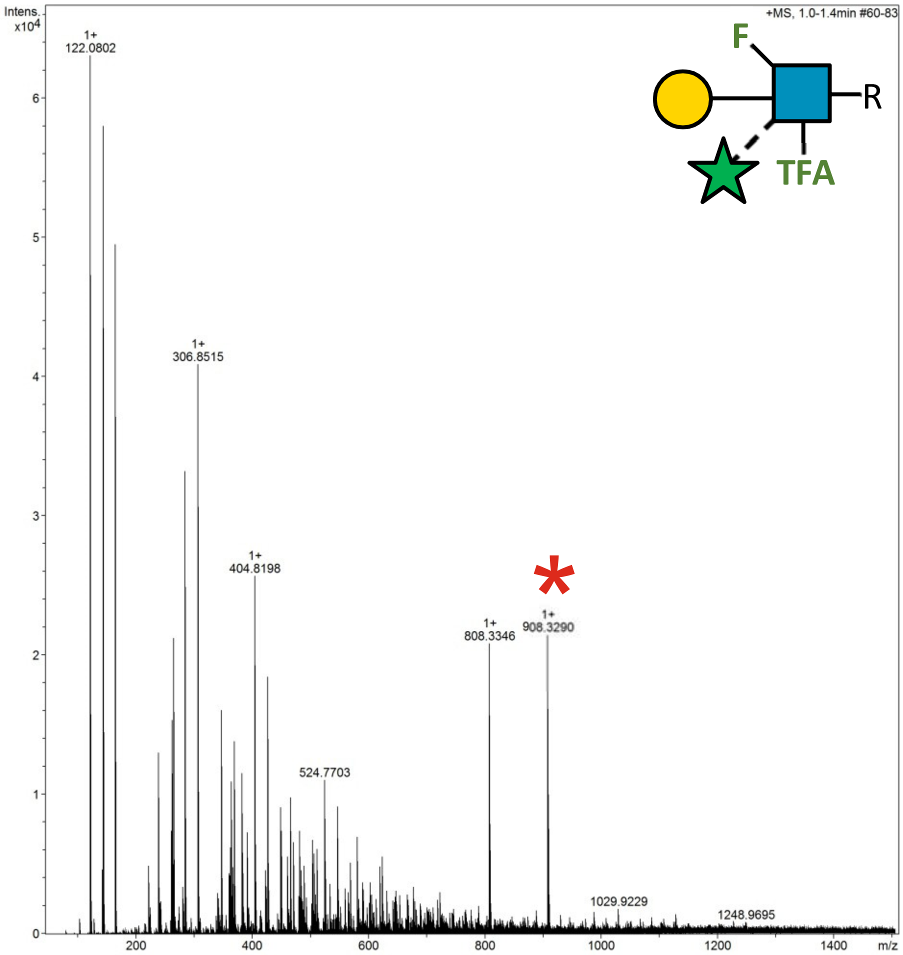

2.18.155 Supplementary Figure 172. HRMS ITag screening assay mass spectrum of synthesis of **Gal β1-4 (Ara α1-3) 6,6-diFGlcNAc-ITag**

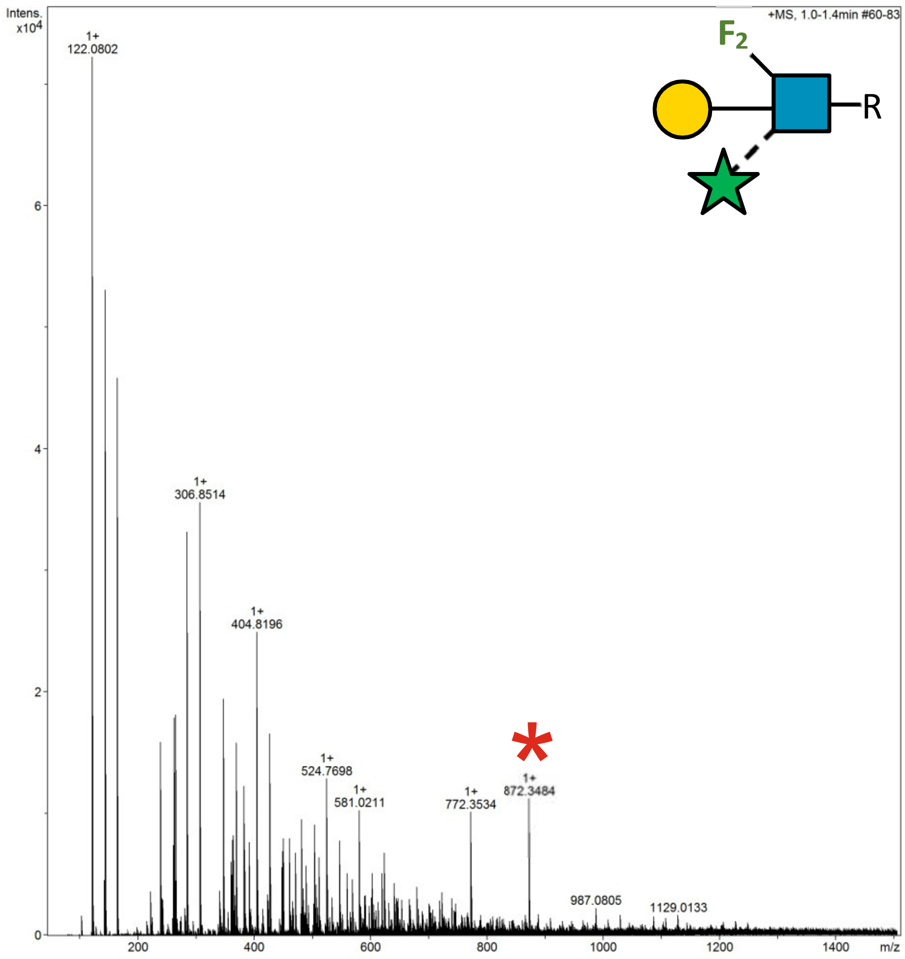

2.18.156 Supplementary Figure 173. HRMS ITag screening assay mass spectrum of synthesis of **Gal β1-4 (Ara α1-3) 6,6-diFGlcNTFA-ITag**

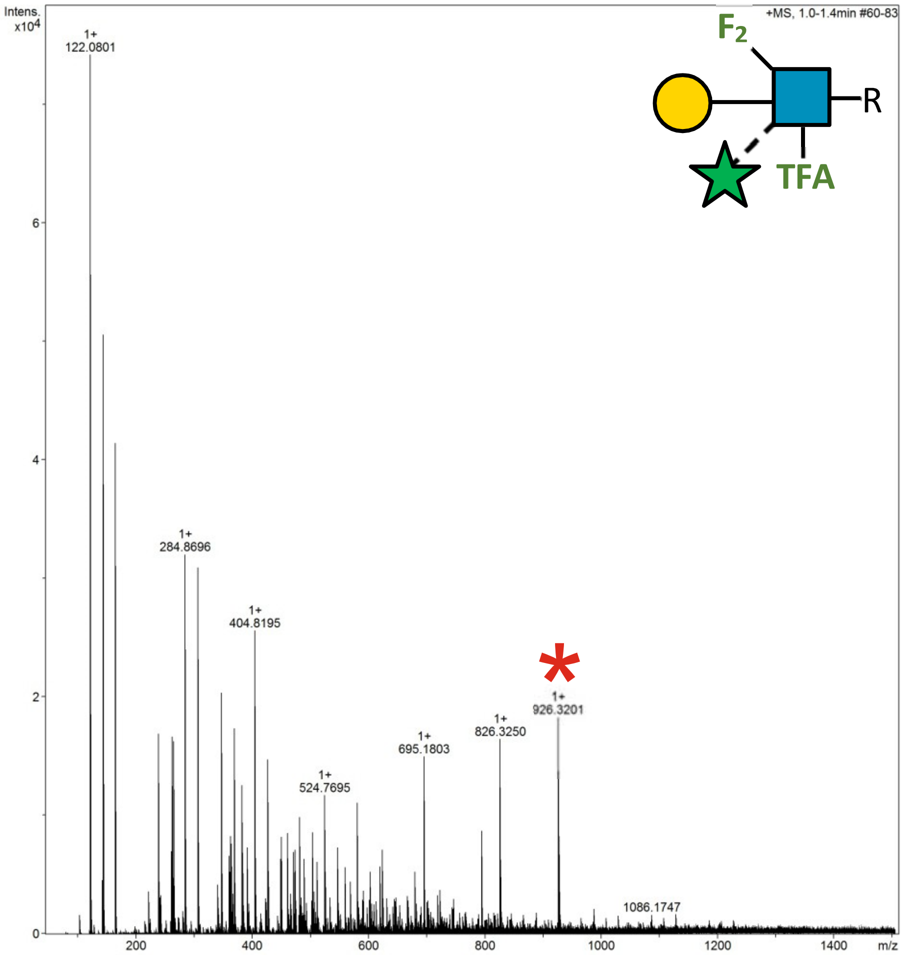

2.18.157 Supplementary Figure 174. HRMS ITag screening assay mass spectrum of synthesis of **3F-Gal β1-4 (Ara α1-3) GlcNAc-ITag**

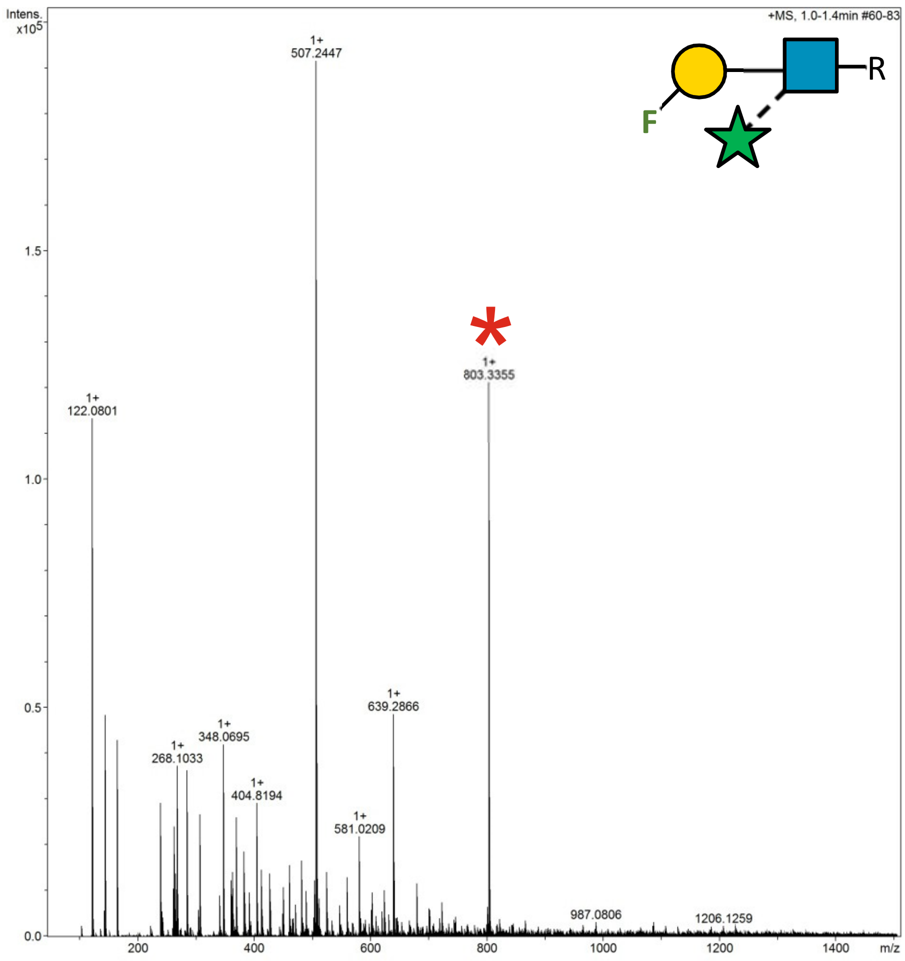

2.18.158 Supplementary Figure 175. HRMS ITag screening assay mass spectrum of synthesis of **3F-Gal β1-4 (Ara α1-3) GlcNTFA-ITag**

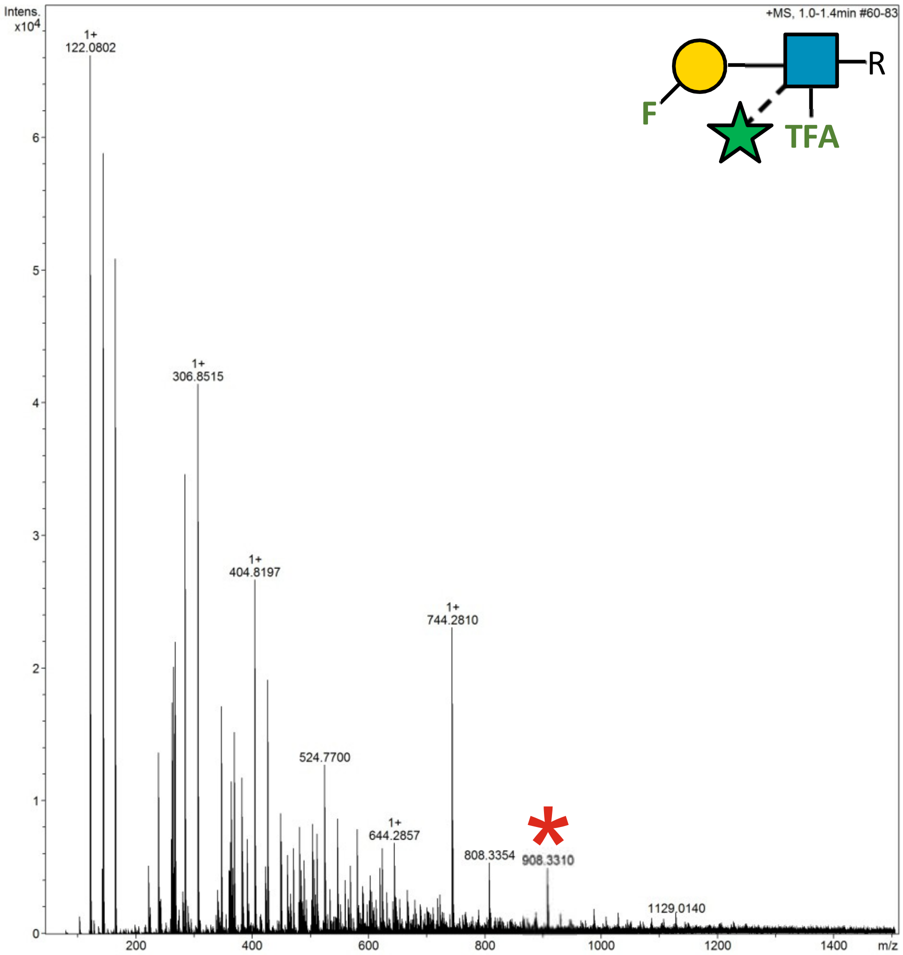

2.18.159 Supplementary Figure 176. HRMS ITag screening assay mass spectrum of synthesis of **3F-Gal β1-4 (Ara α1-3) 6F-GlcNAc-ITag**

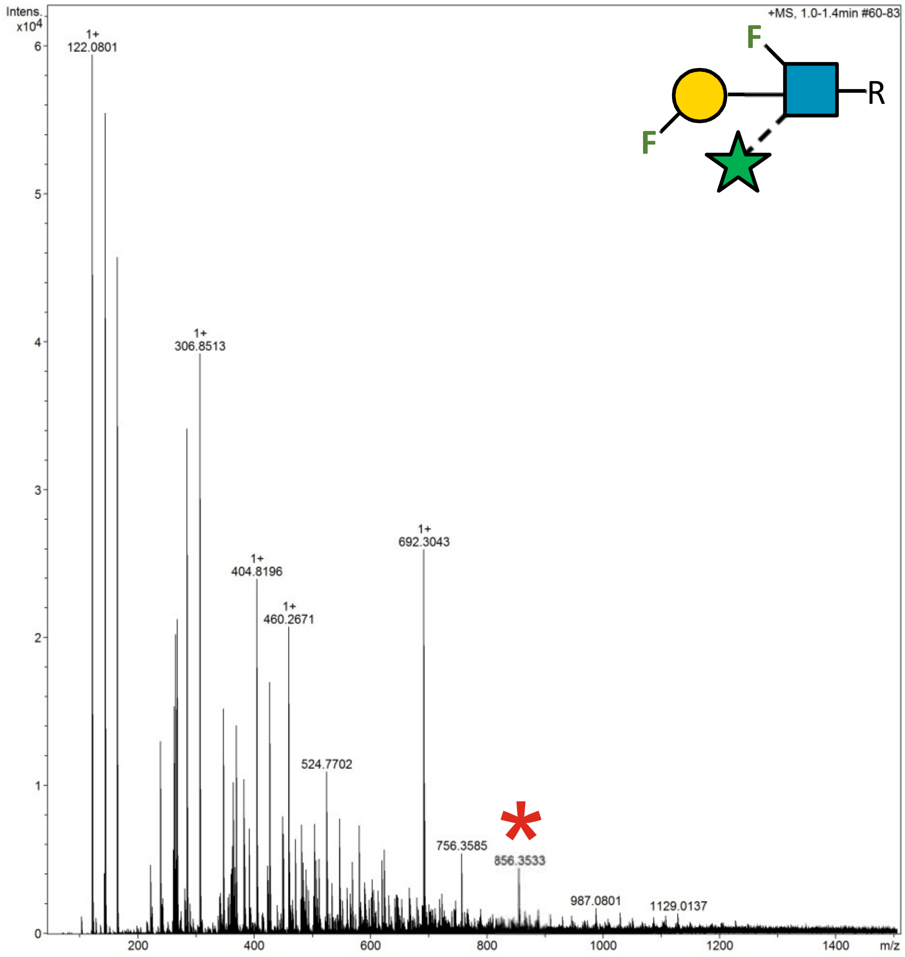

2.18.160 Supplementary Figure 177. HRMS ITag screening assay mass spectrum of synthesis of **3F-Gal β1-4 (Ara α1-3) 6F-GlcNTFA-ITag**

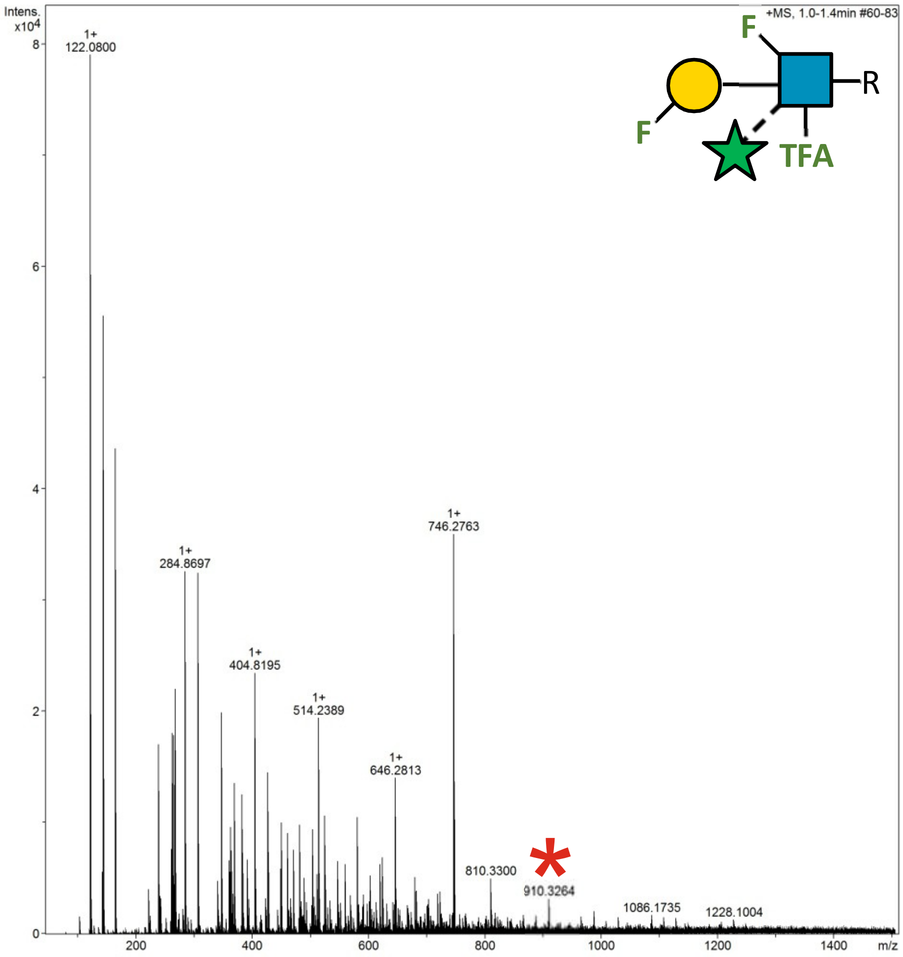

2.18.161 Supplementary Figure 178. HRMS ITag screening assay mass spectrum of synthesis of **3F-Gal β1-4 (Ara α1-3) 6,6-diFGlcNAc-ITag**

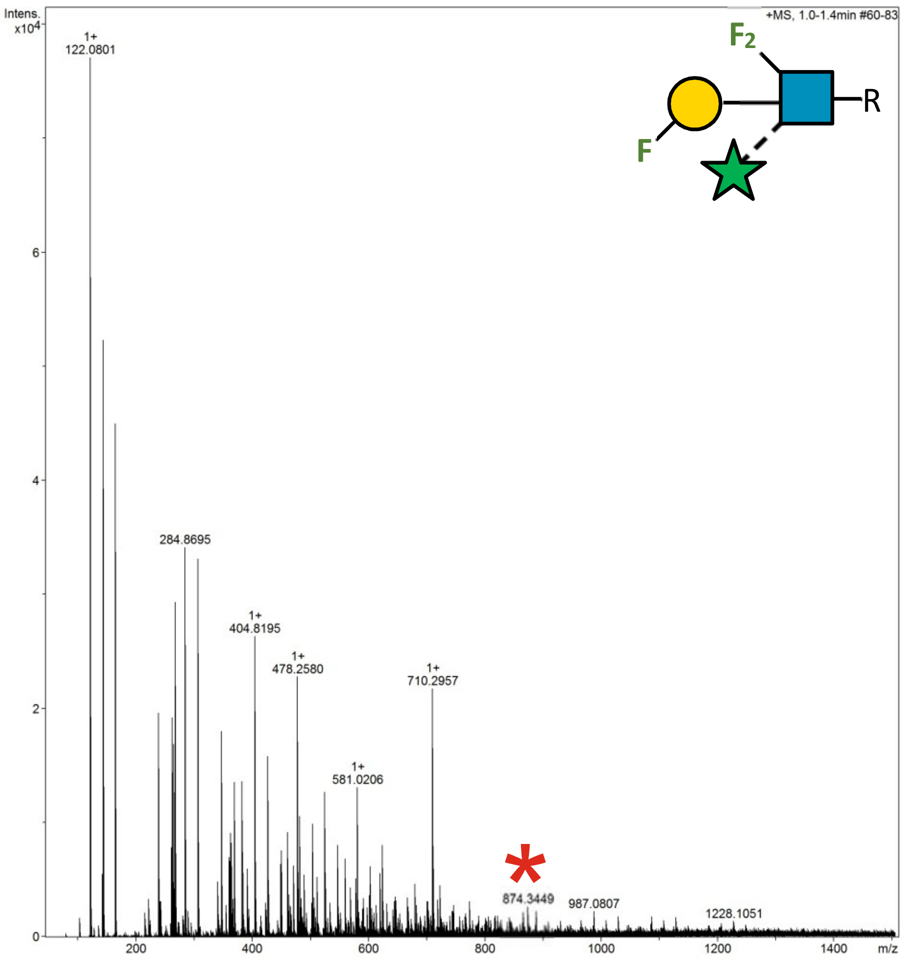

2.18.162 Supplementary Figure 179. HRMS ITag screening assay mass spectrum of synthesis of **3F-Gal β1-4 (Ara α1-3) 6,6-diFGlcNTFA-ITag**

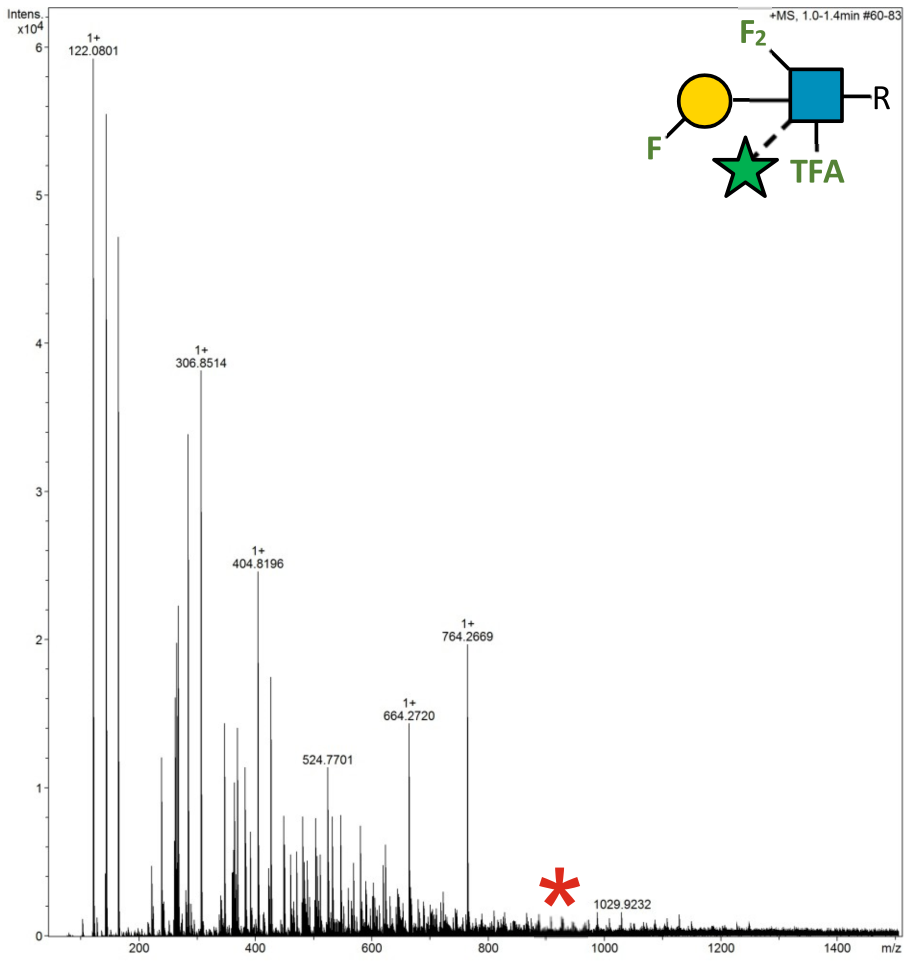

2.18.163 Supplementary Figure 180. HRMS ITag screening assay mass spectrum of synthesis of **4F-Gal β1-4 (Ara α1-3) GlcNAc-ITag**

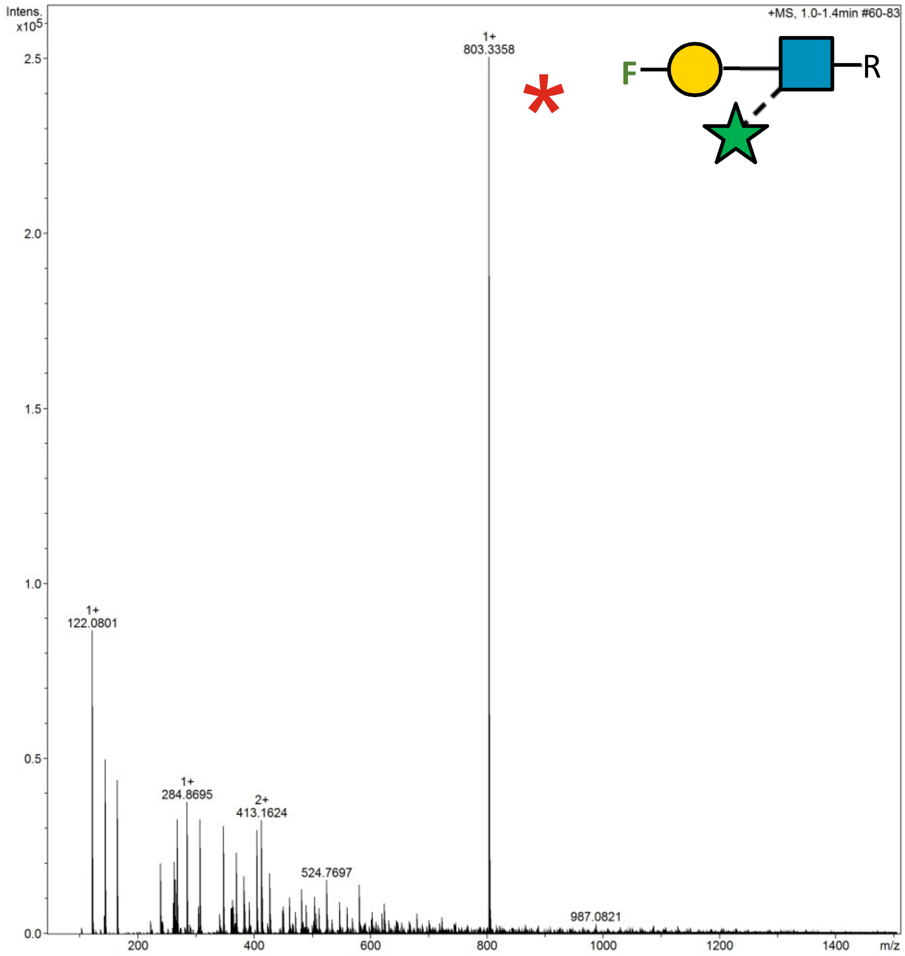

2.18.164 Supplementary Figure 181. HRMS ITag screening assay mass spectrum of synthesis of **4F-Gal β1-4 (Ara α1-3) GlcNTFA-ITag**

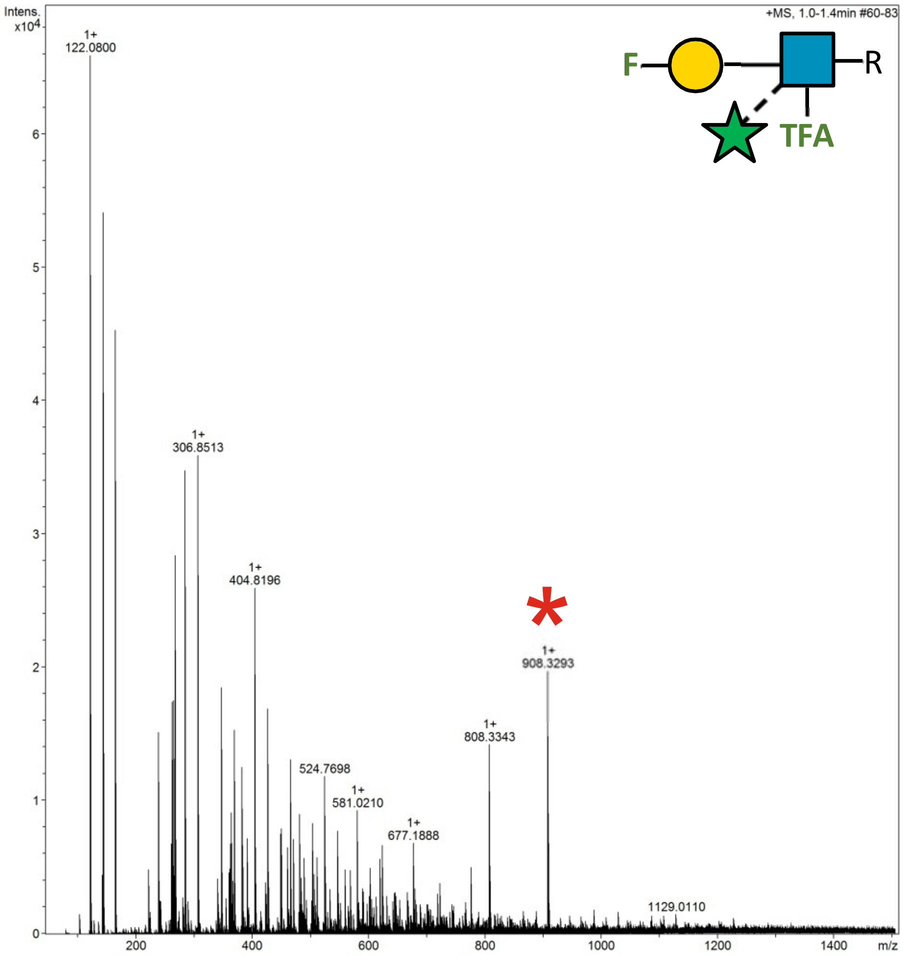

2.18.165 Supplementary Figure 182. HRMS ITag screening assay mass spectrum of synthesis of **4F-Gal β1-4 (Ara α1-3) 6F-GlcNAc-ITag**

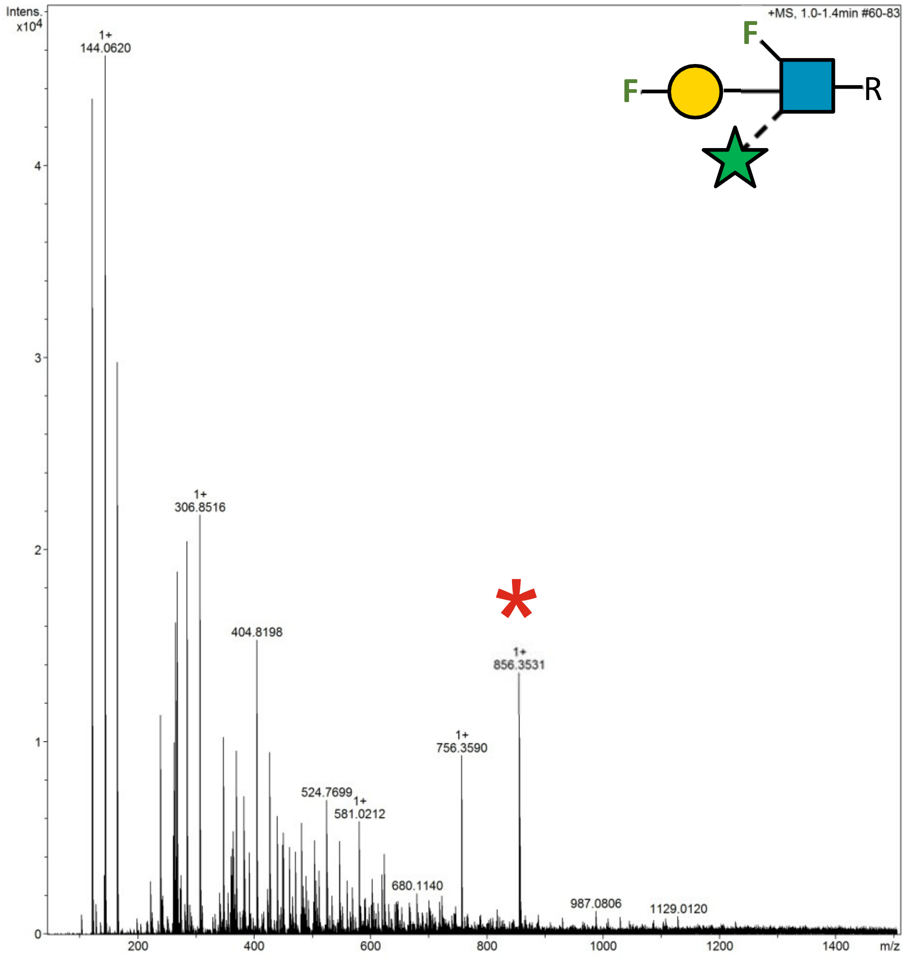

2.18.166 Supplementary Figure 183. HRMS ITag screening assay mass spectrum of synthesis of **4F-Gal β1-4 (Ara α1-3) 6F-GlcNTFA-ITag**

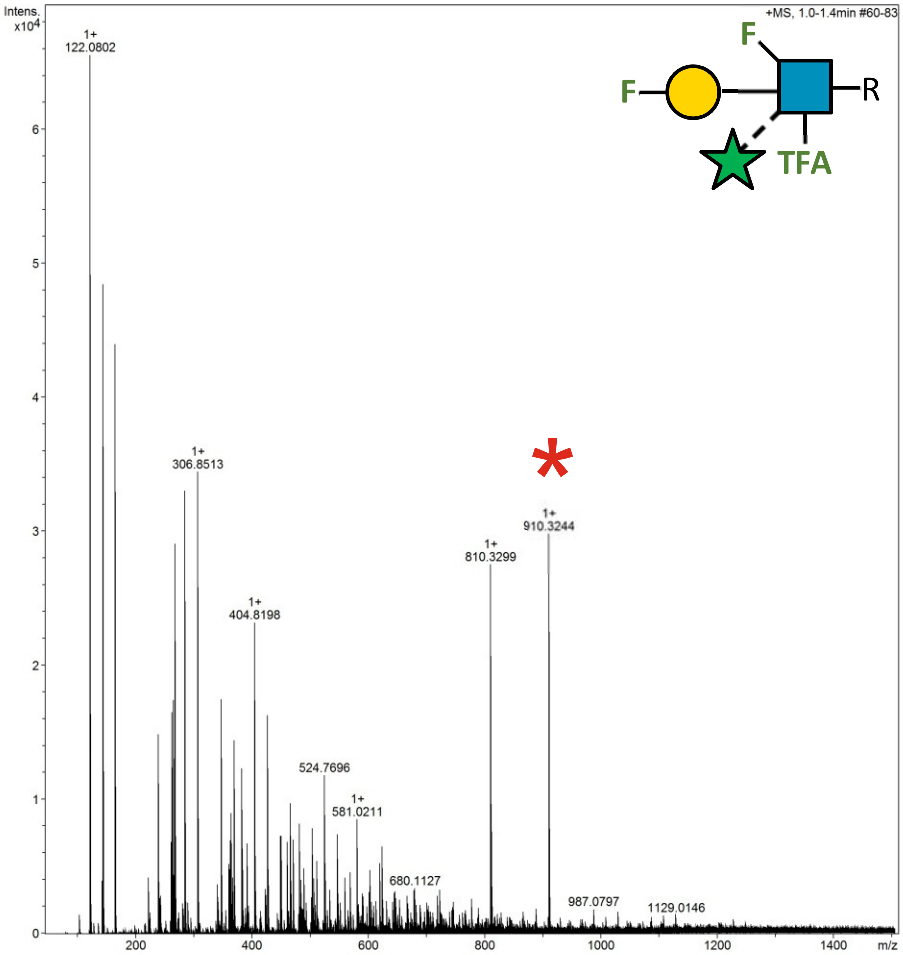

2.18.167 Supplementary Figure 184. HRMS ITag screening assay mass spectrum of synthesis of **4F-Gal β1-4 (Ara α1-3) 6,6-diFGlcNAc-ITag**

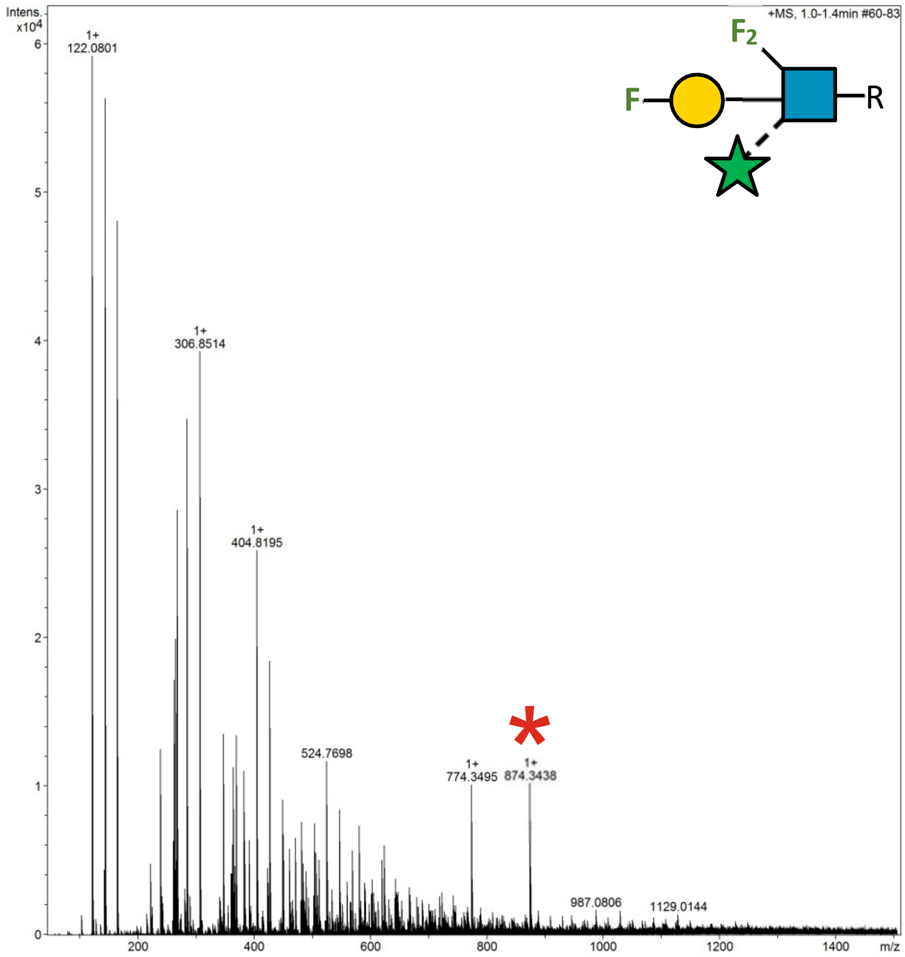

2.18.168 Supplementary Figure 185. HRMS ITag screening assay mass spectrum of synthesis of **4F-Gal β1-4 (Ara α1-3) 6,6-diFGlcNTFA-ITag**

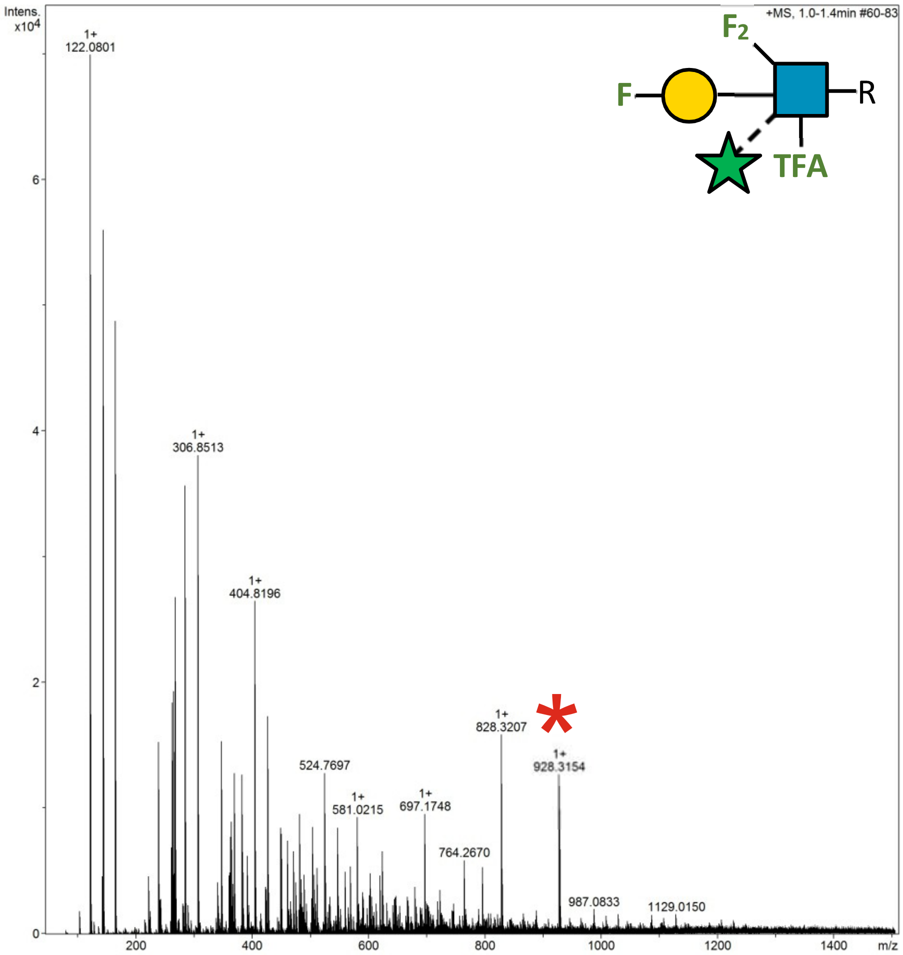

2.18.169 Supplementary Figure 186. HRMS ITag screening assay mass spectrum of synthesis of **6F-Gal β1-4 (Ara α1-3) GlcNAc-ITag**

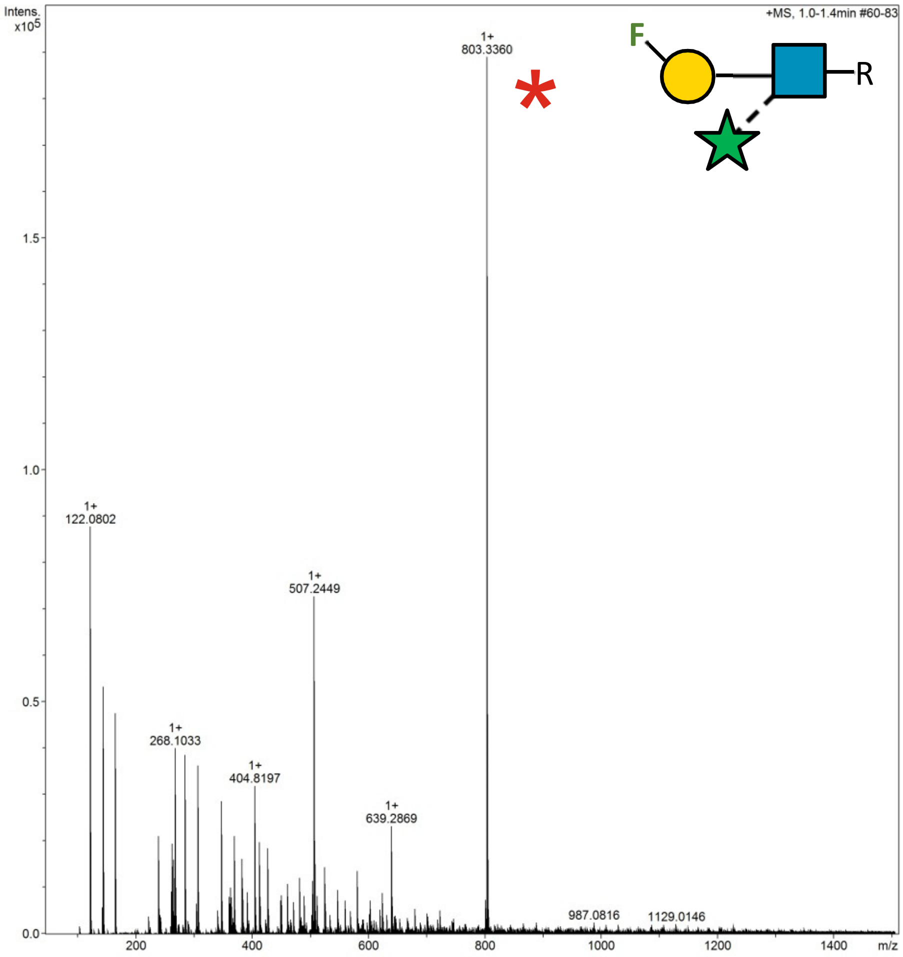

2.18.170 Supplementary Figure 187. HRMS ITag screening assay mass spectrum of synthesis of **6F-Gal β1-4 (Ara α1-3) GlcNTFA-ITag**

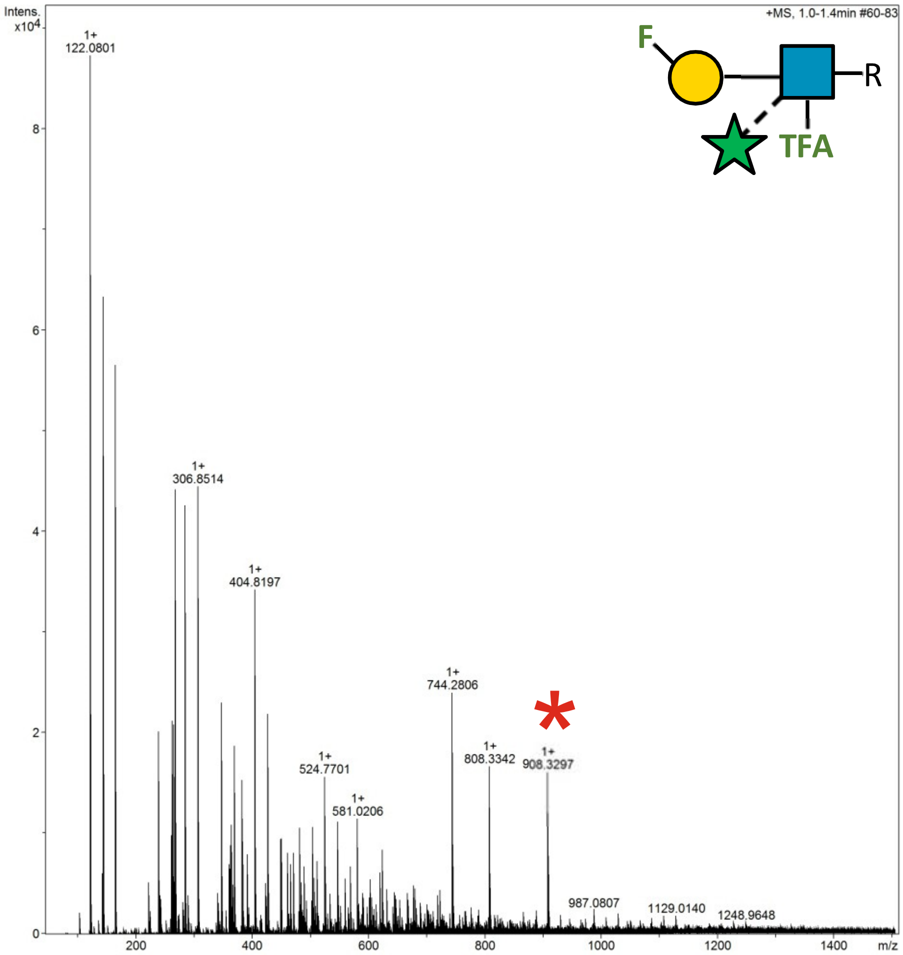

2.18.171 Supplementary Figure 188. HRMS ITag screening assay mass spectrum of synthesis of **6F-Gal β1-4 (Ara α1-3) 6F-GlcNAc-ITag**

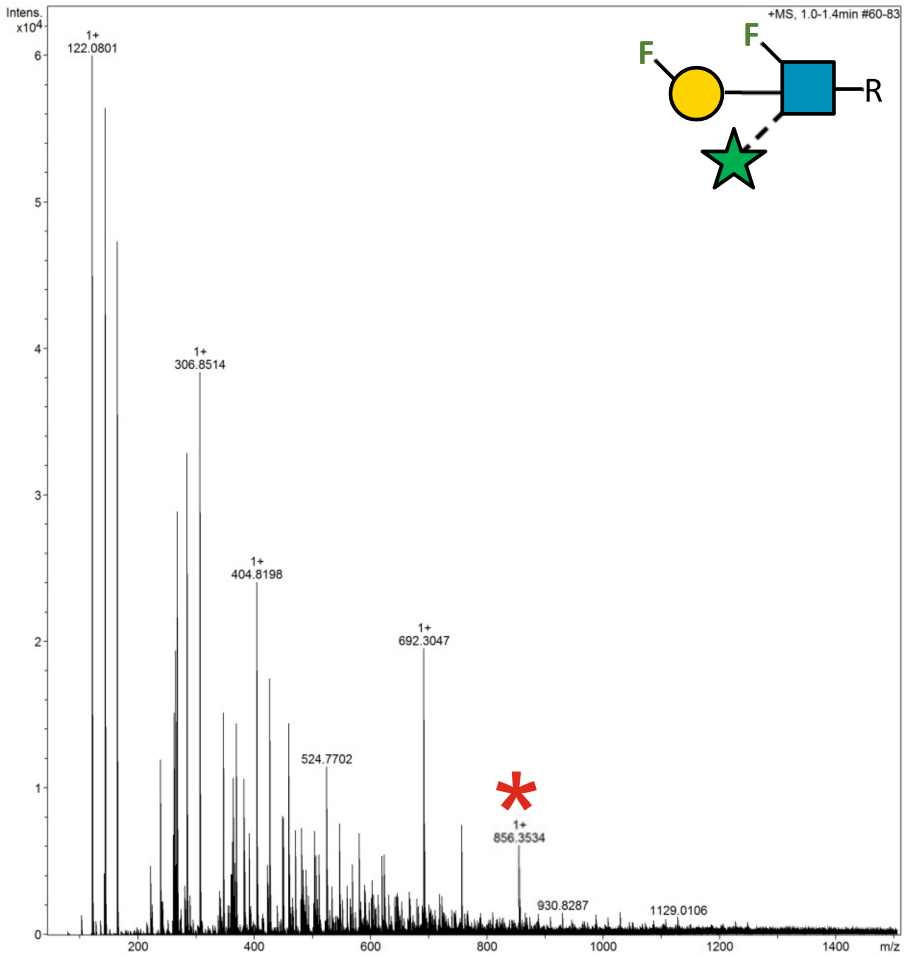

2.18.172 Supplementary Figure 189. HRMS ITag screening assay mass spectrum of synthesis of **6F-Gal β1-4 (Ara α1-3) 6F-GlcNTFA-ITag**

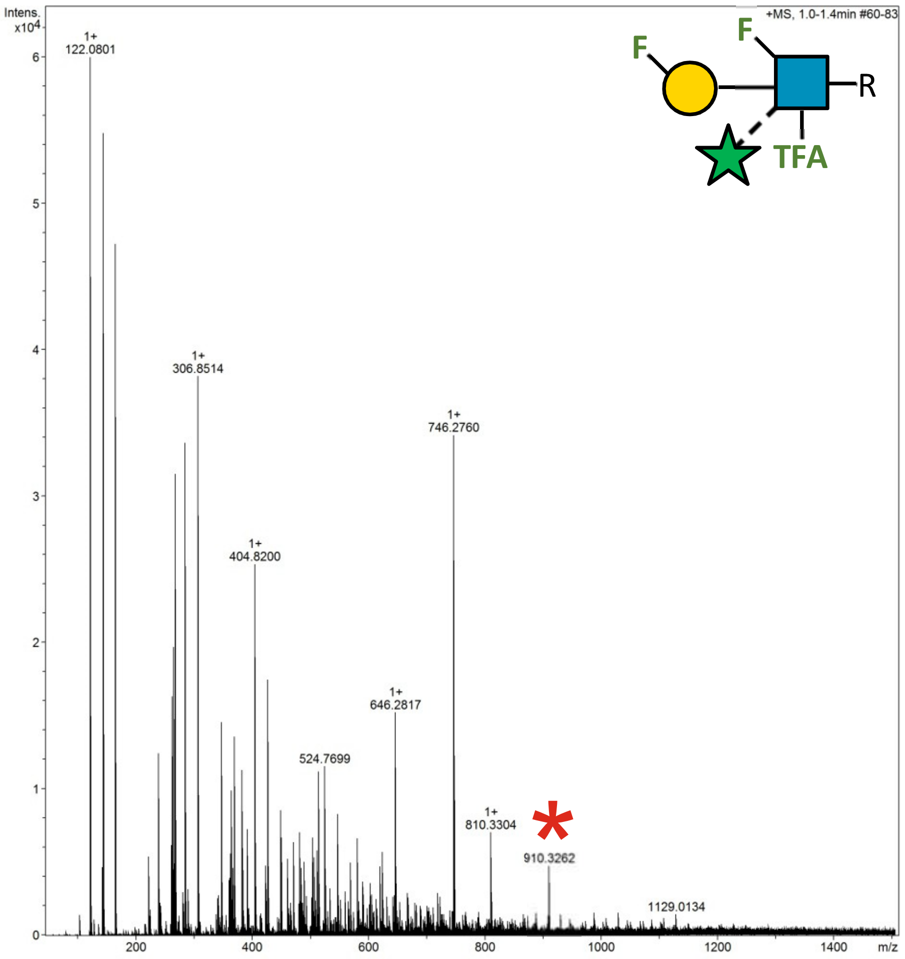

2.18.173 Supplementary Figure 190. HRMS ITag screening assay mass spectrum of synthesis of **6F-Gal β1-4 (Ara α1-3) 6,6-diFGlcNAc-ITag**

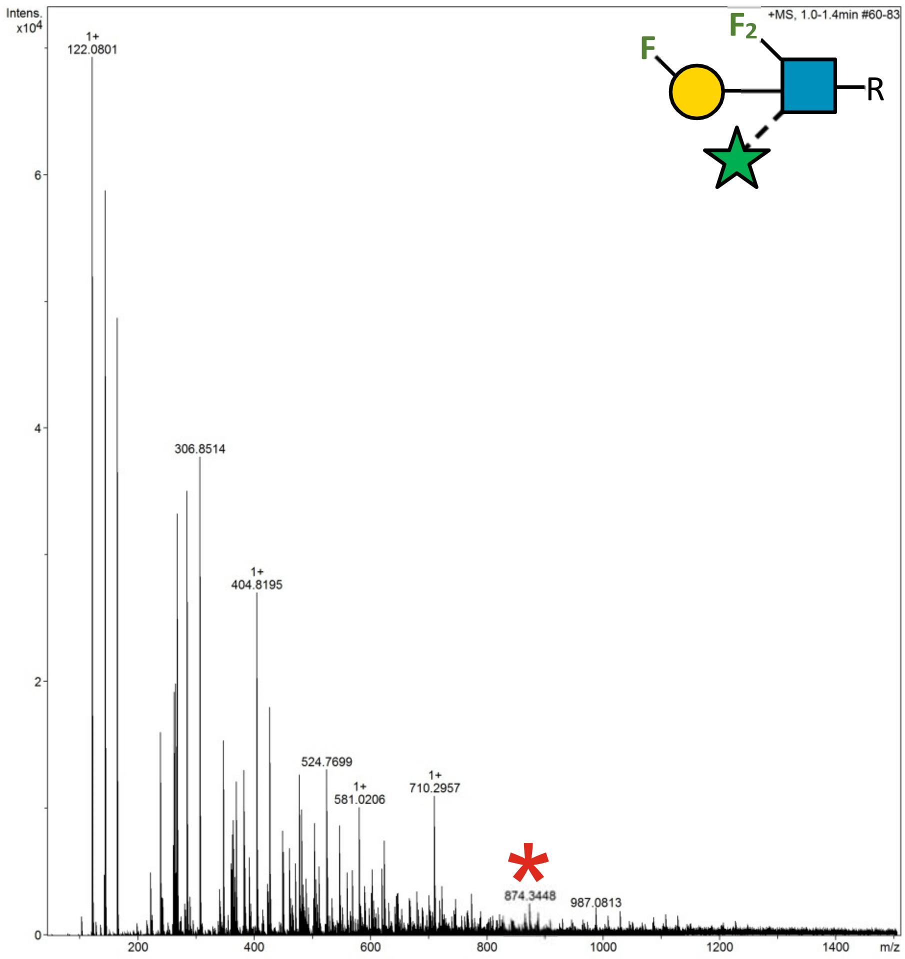

2.18.174 Supplementary Figure 191. HRMS ITag screening assay mass spectrum of synthesis of 6F-Gal β1-4 (Ara α1-3) 6,6-diFGlcNTFA-ITag

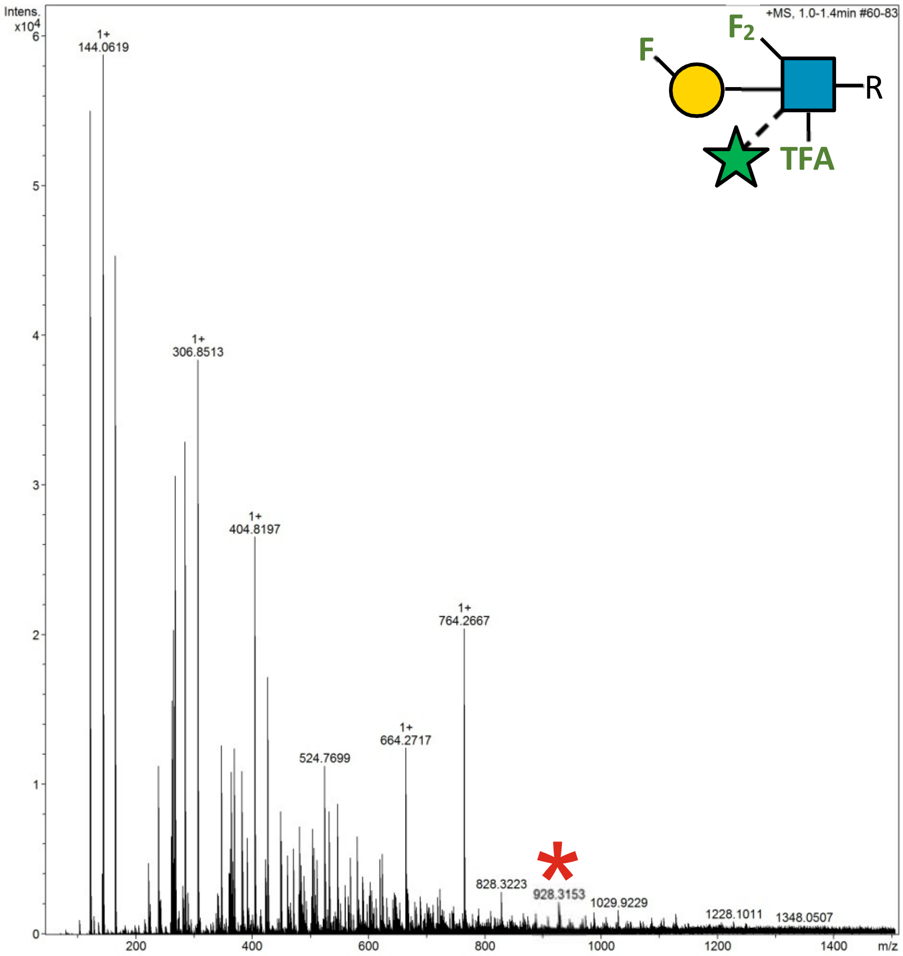

2.18.175 Supplementary Figure 192. HRMS ITag screening assay mass spectrum of synthesis of **6d-Gal β1-4 (Ara α1-3) GlcNAc-ITag**

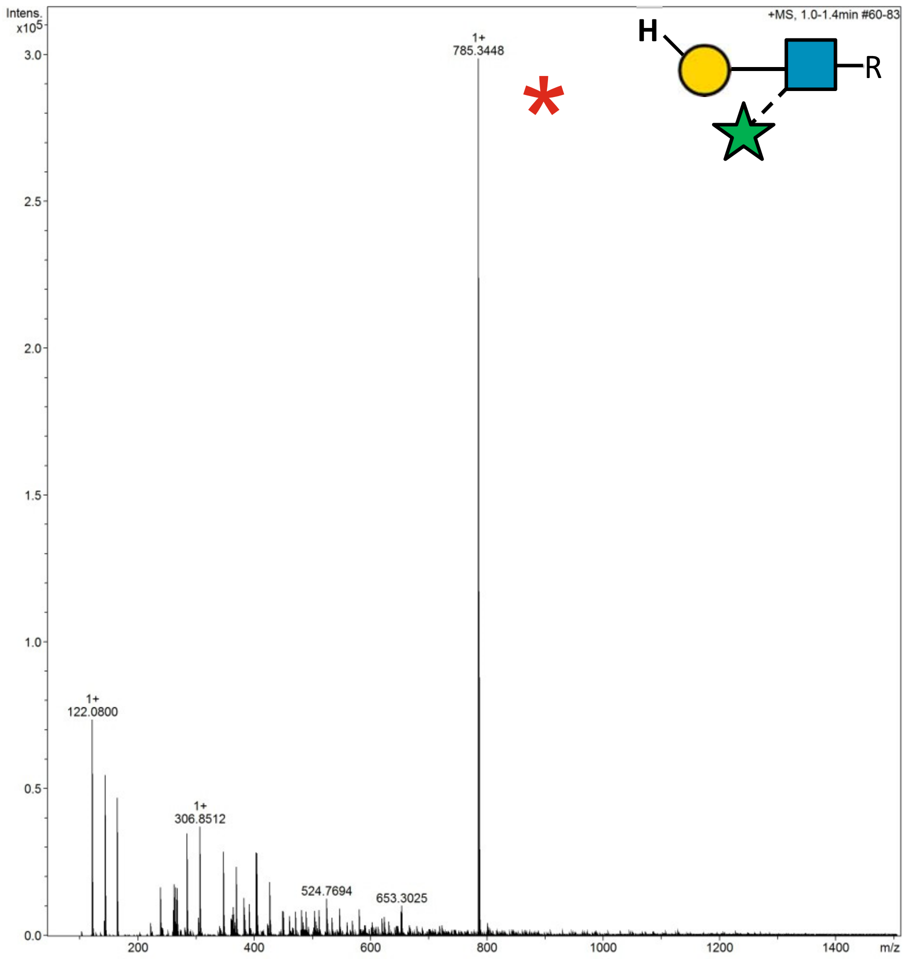

2.18.176 Supplementary Figure 193. HRMS ITag screening assay mass spectrum of synthesis of **6d-Gal β1-4 (Ara α1-3) GlcNTFA-ITag**

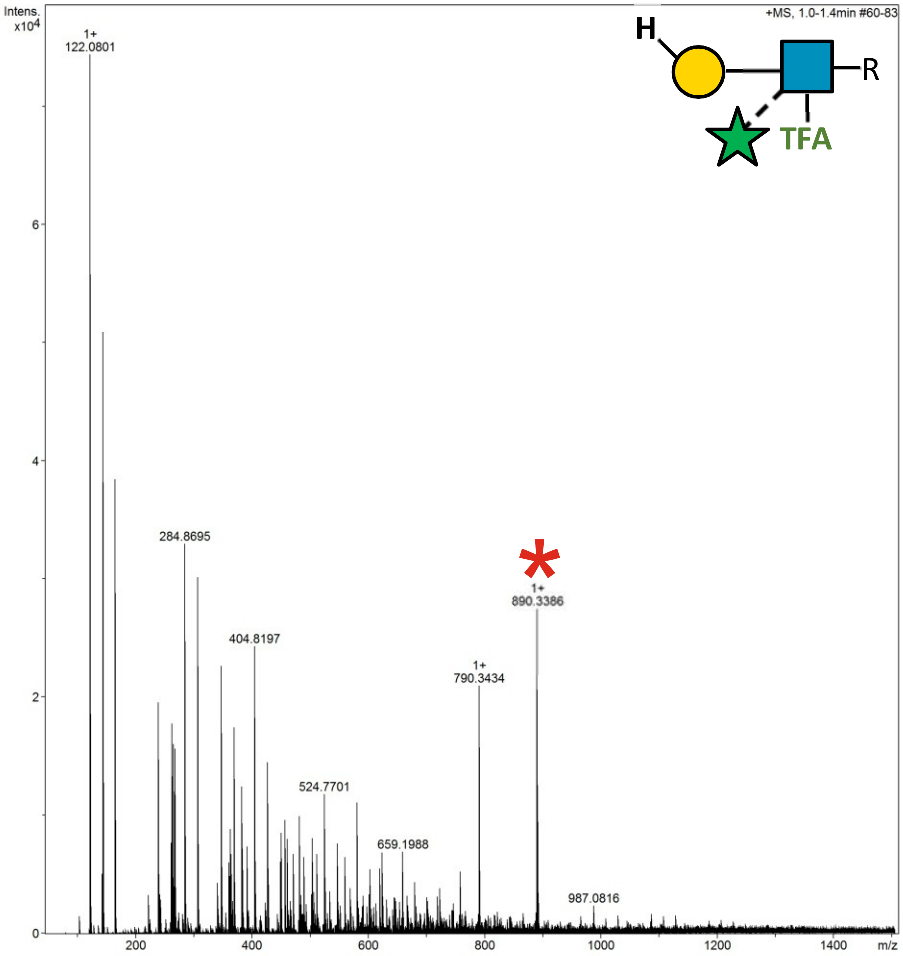

2.18.177 Supplementary Figure 194. HRMS ITag screening assay mass spectrum of synthesis of **6d-Gal β1-4 (Ara α1-3) 6F-GlcNAc-ITag**

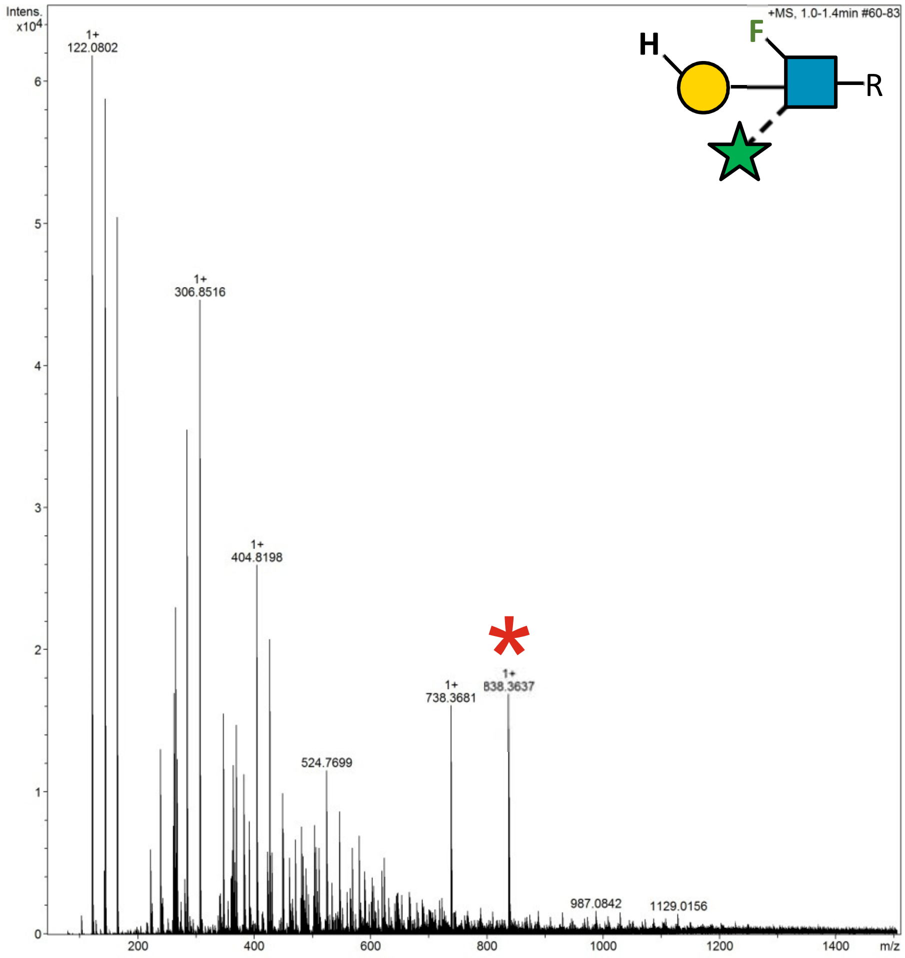

2.18.178 Supplementary Figure 195. HRMS ITag screening assay mass spectrum of synthesis of **6d-Gal β1-4 (Ara α1-3) 6F-GlcNTFA-ITag**

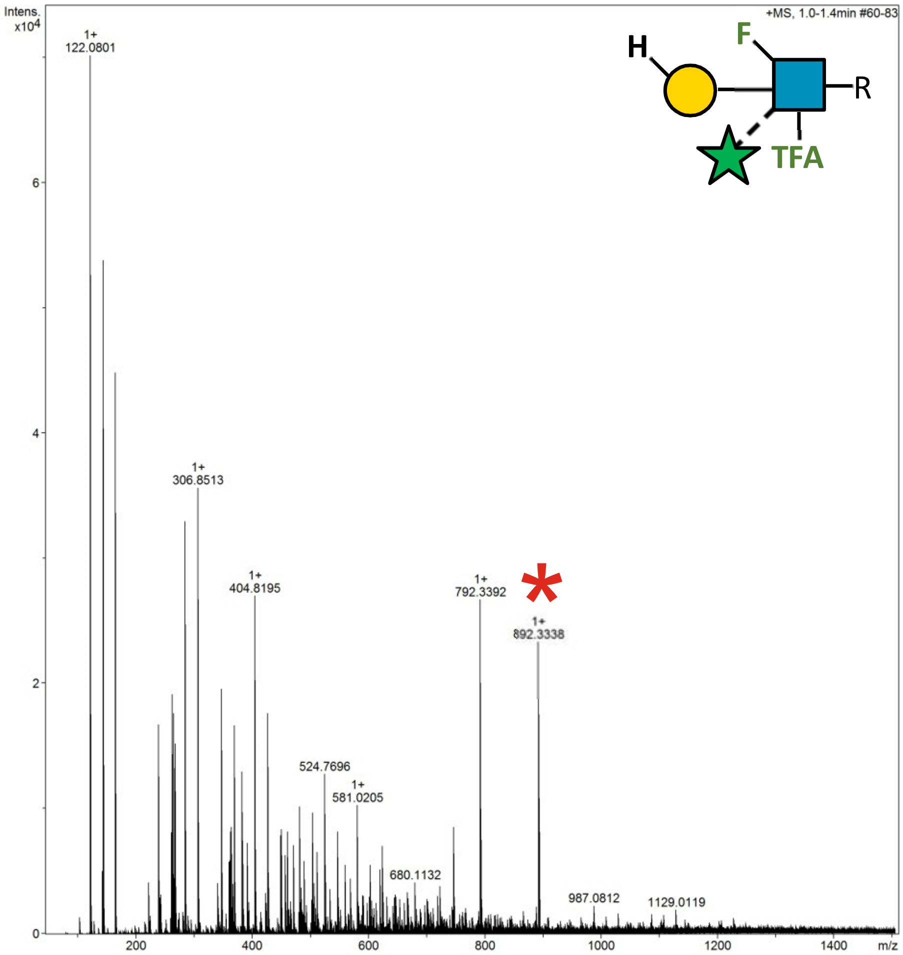

2.18.179 Supplementary Figure 196. HRMS ITag screening assay mass spectrum of synthesis of **6d-Gal β1-4 (Ara α1-3) 6,6-diFGlcNAc-ITag**

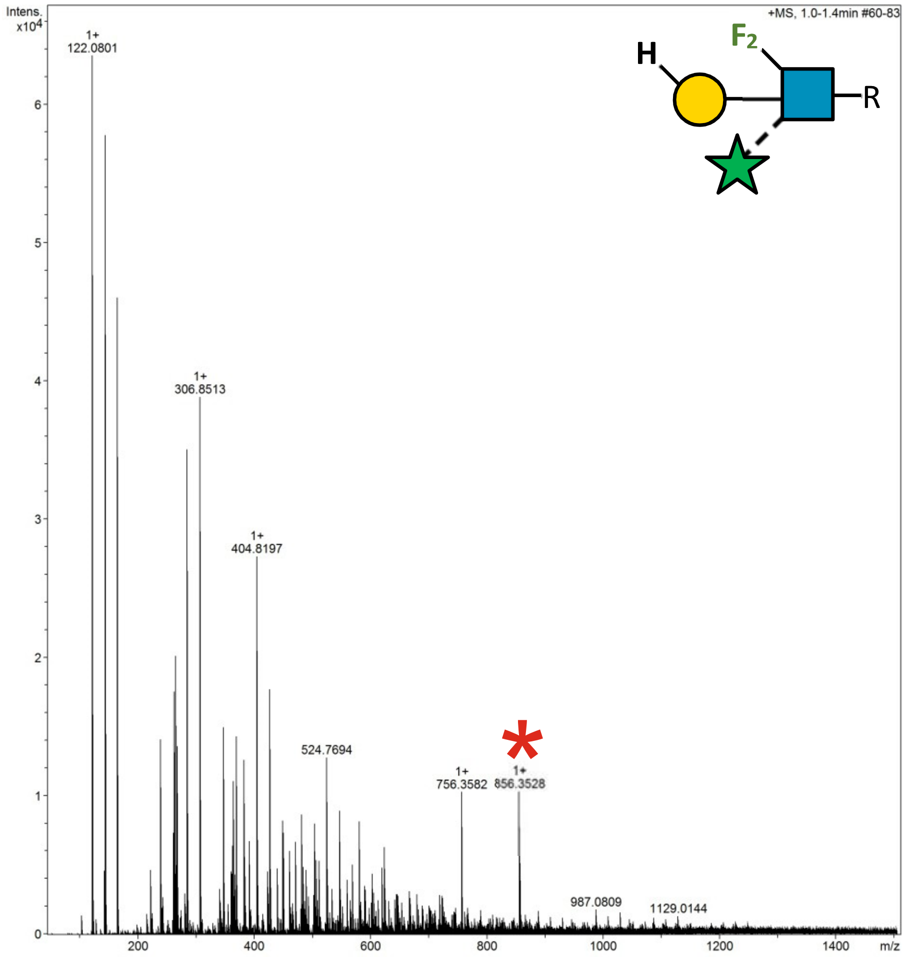

2.18.180 Supplementary Figure 197. HRMS ITag screening assay mass spectrum of synthesis of 6d-Gal β1-4 (Ara α1-3) 6,6-diFGlcNTFA-ITag

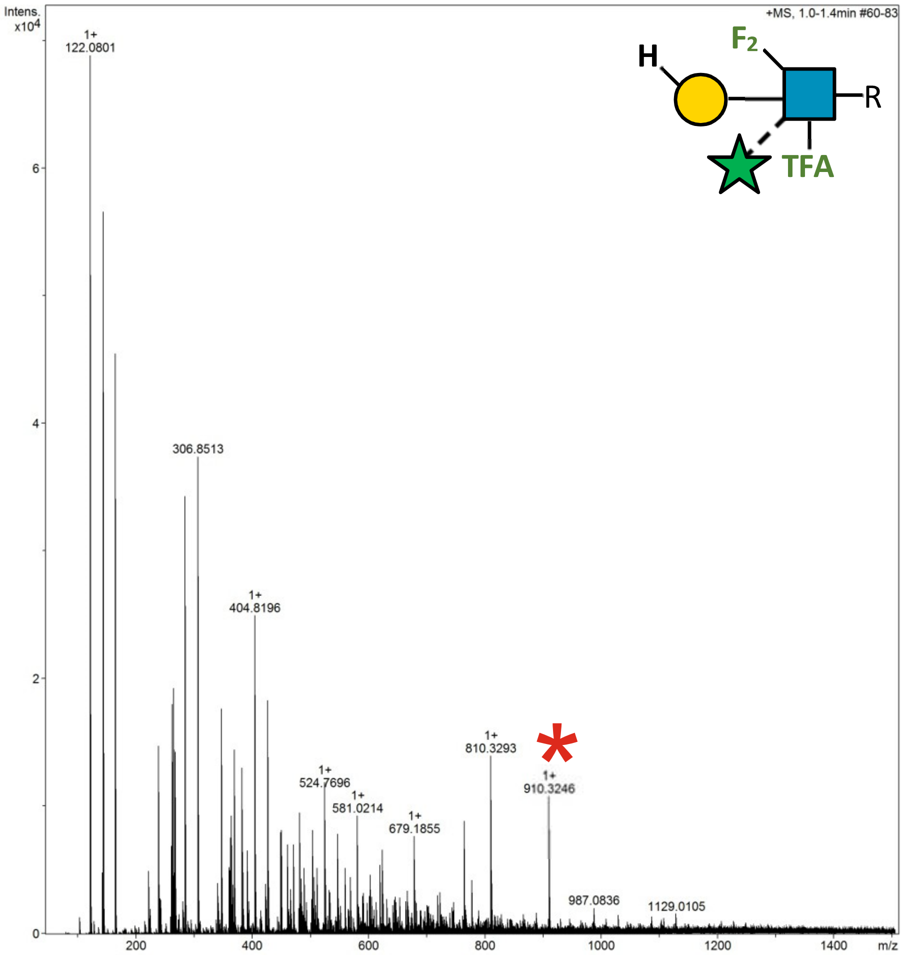

2.19 Supplementary Figures 198-266. NMR spectra of the upscaled Lewis^x and its glycofluoroforms

2.19.1 LeX1

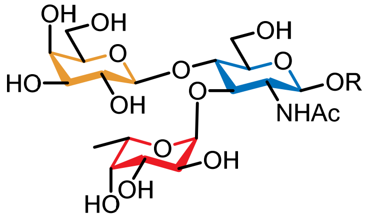

2.19.1.1 Supplementary Figure 198. ^{1}H NMR spectrum for compound **LeX**

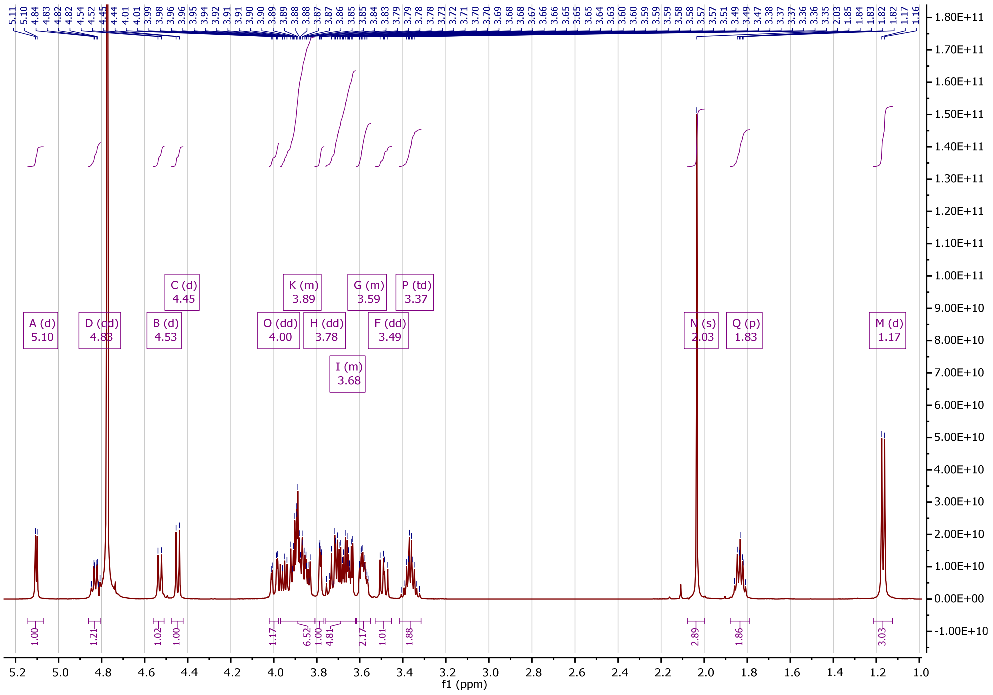

2.19.1.2 Supplementary Figure 199. ^{13}C NMR spectrum for compound **LeX1**

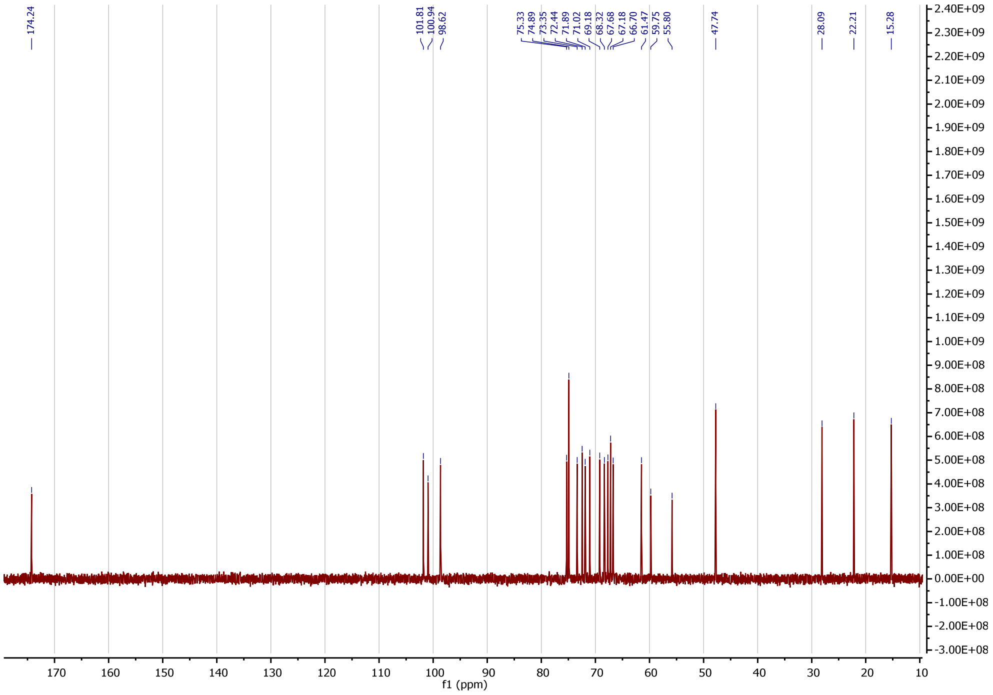

2.19.2 LeX2

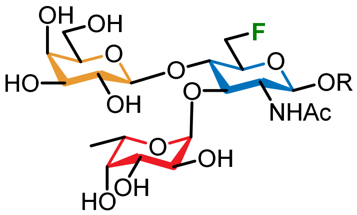

2.19.2.1 Supplementary Figure 200. ^{1}H NMR spectrum for compound **LeX2**

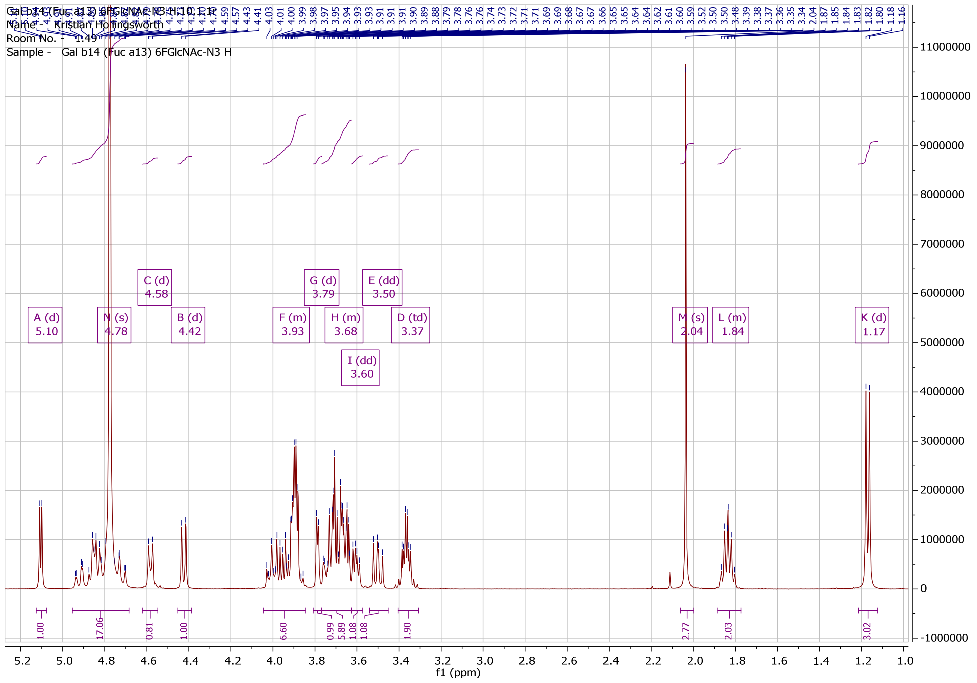

2.19.2.2 Supplementary Figure 201. ^{13}C NMR spectrum for compound **LeX2**

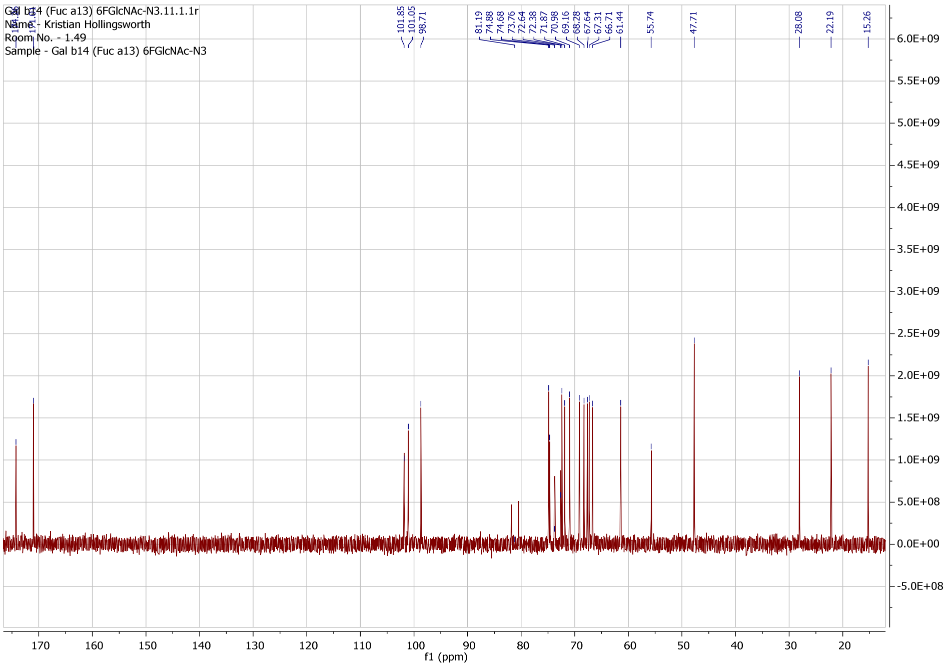

2.19.2.3 Supplementary Figure 202. $^{19}F\{1H\}$ NMR spectrum for compound **LeX2**

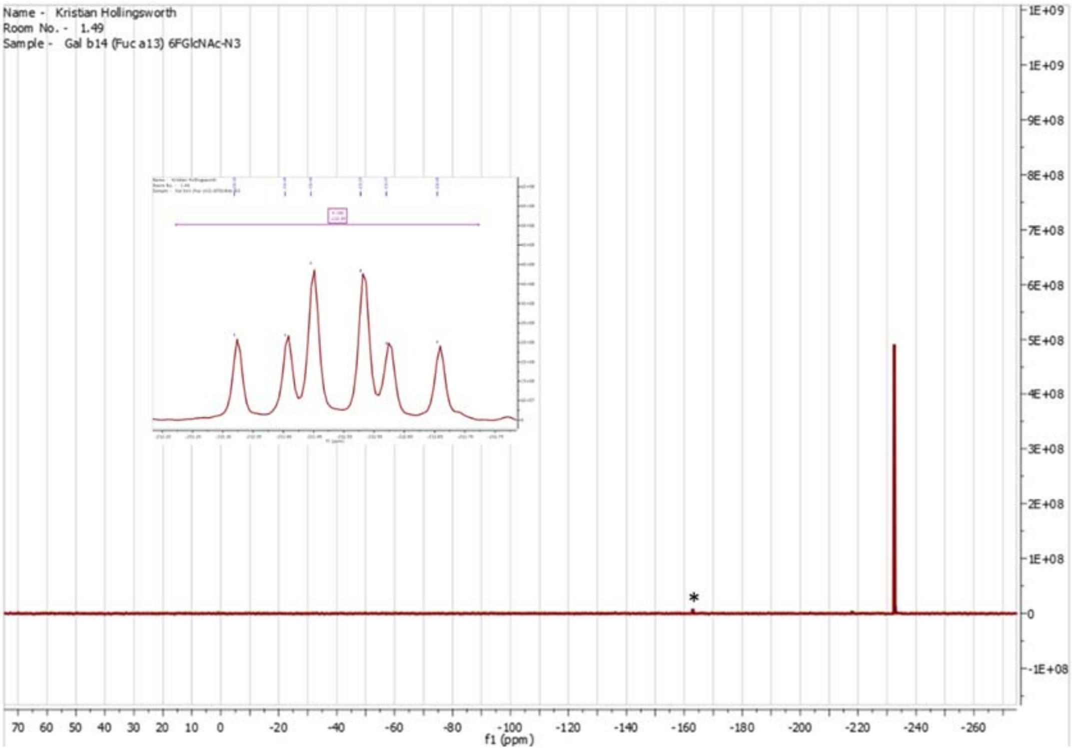

2.19.3 LeX3

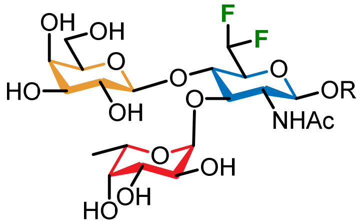

2.19.3.1 Supplementary Figure 203. ^1H NMR spectrum for compound **LeX3**

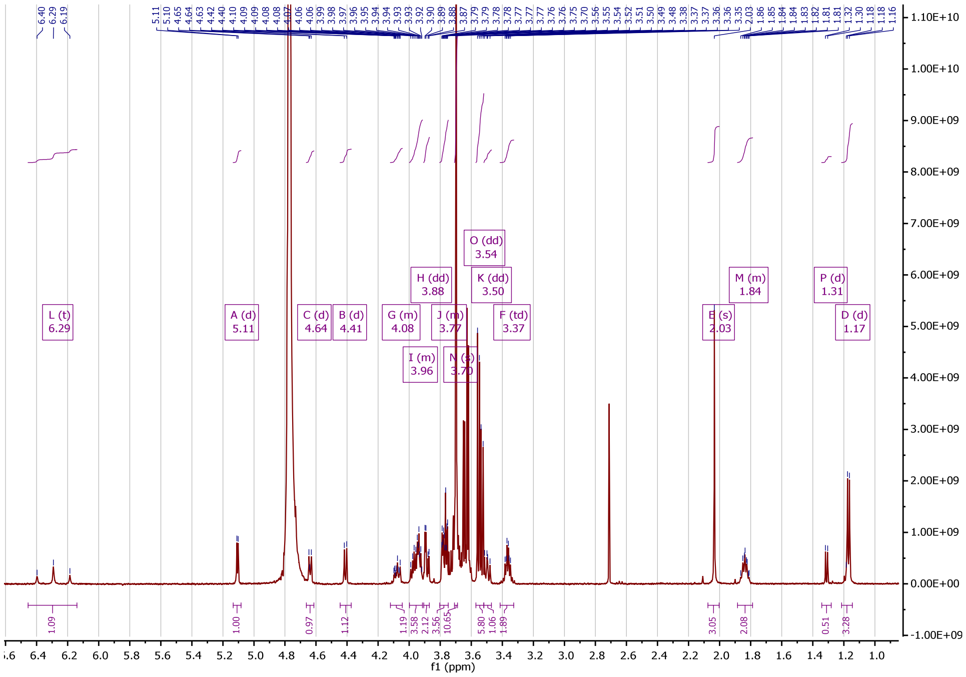

2.19.3.2 Supplementary Figure 204. HSQC NMR spectrum for compound **LeX3**

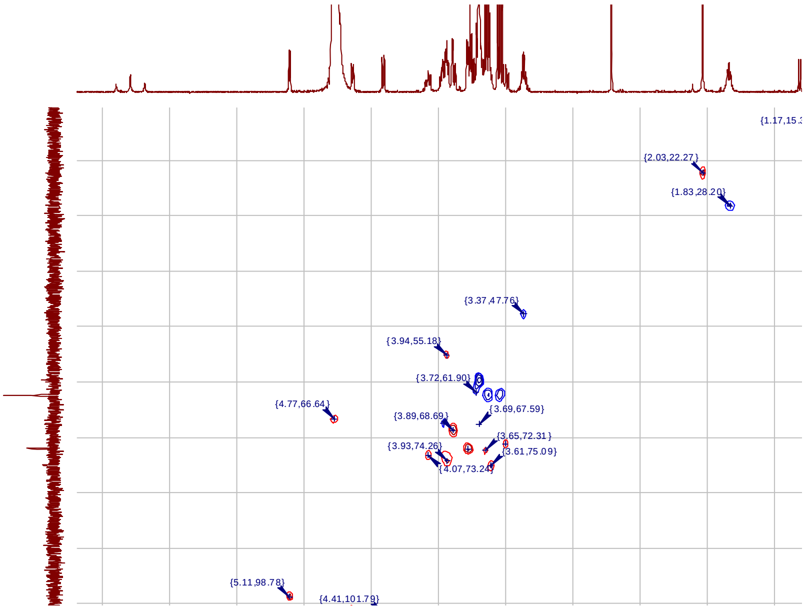

2.19.3.3 Supplementary Figure 205. $^{19}F\{1H\}$ NMR spectrum for compound **LeX3**

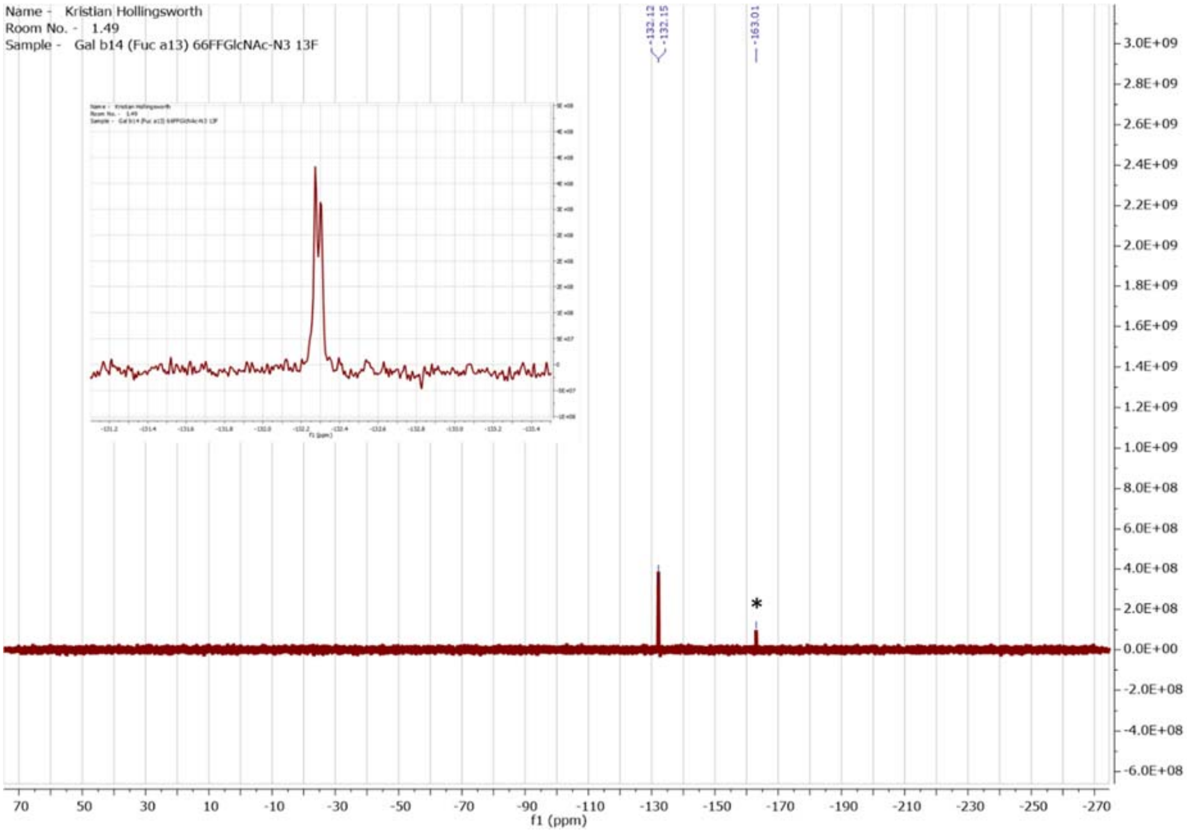

2.19.4 LeX4

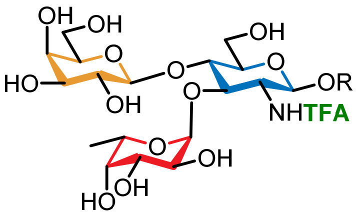

2.19.4.1 Supplementary Figure 206. ^{1}H NMR spectrum for compound **LeX4**

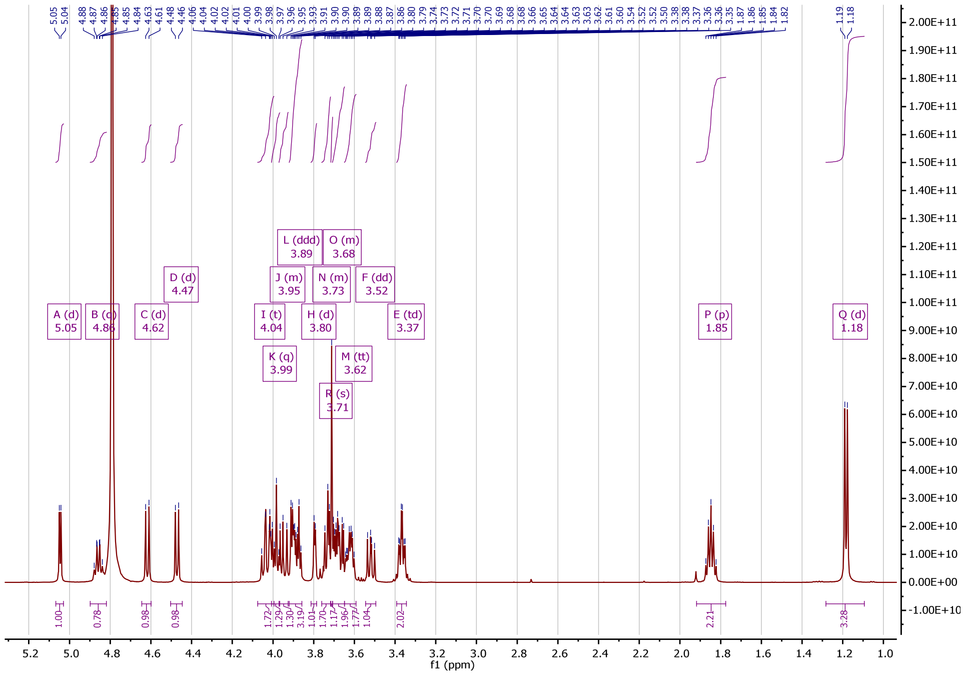

2.19.4.2 Supplementary Figure 207. ^{13}C NMR spectrum for compound **LeX4**

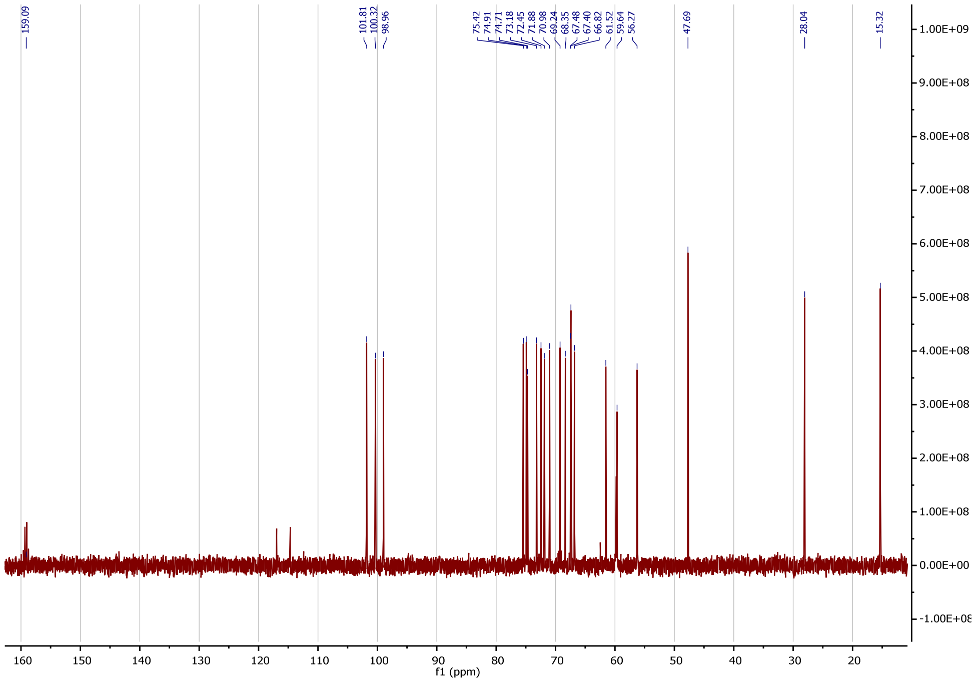

2.19.4.3 Supplementary Figure 208. $^{19}F\{1H\}$ NMR spectrum for compound LeX4

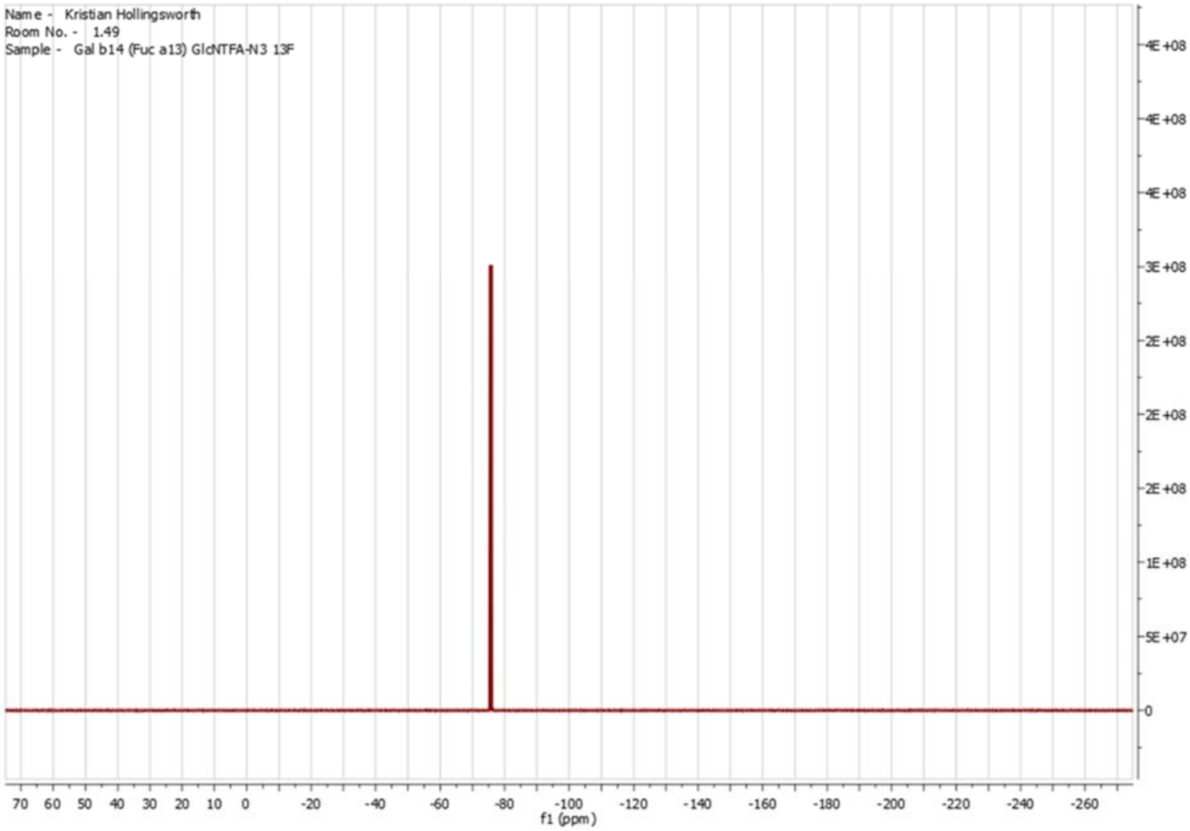

2.19.5 LeX5

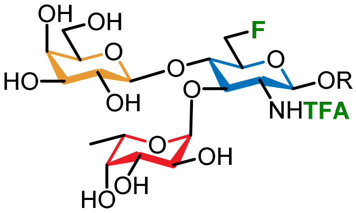

2.19.5.1 Supplementary Figure 209. ^{1}H NMR spectrum for compound **LeX5**

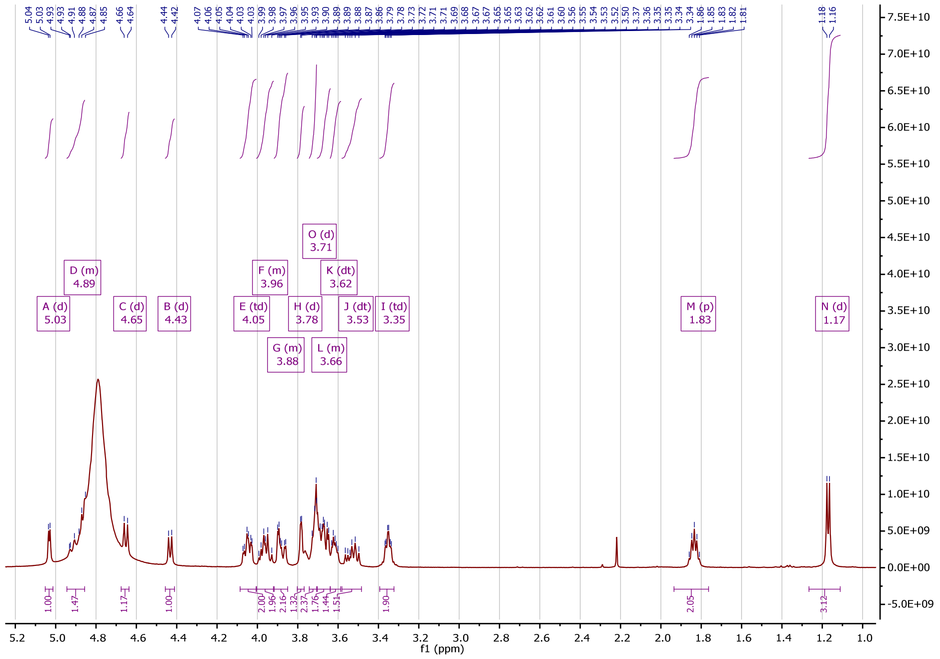

2.19.5.2 Supplementary Figure 210. ^{13}C NMR spectrum for compound **LeX5**

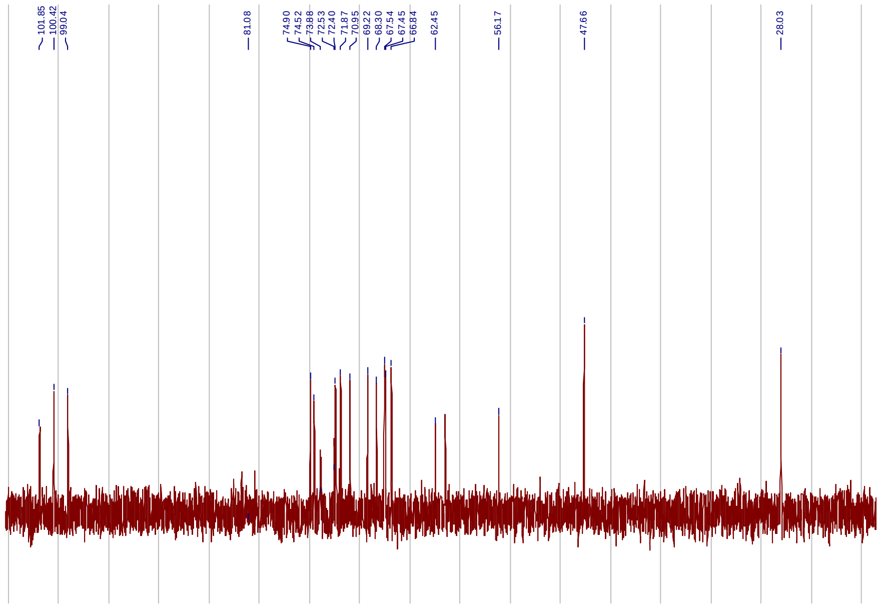

2.19.5.3 Supplementary Figure 211. $^{19}F\{1H\}$ NMR spectrum for compound LeX5

Note: contains traces of trifluoroacetic acid.

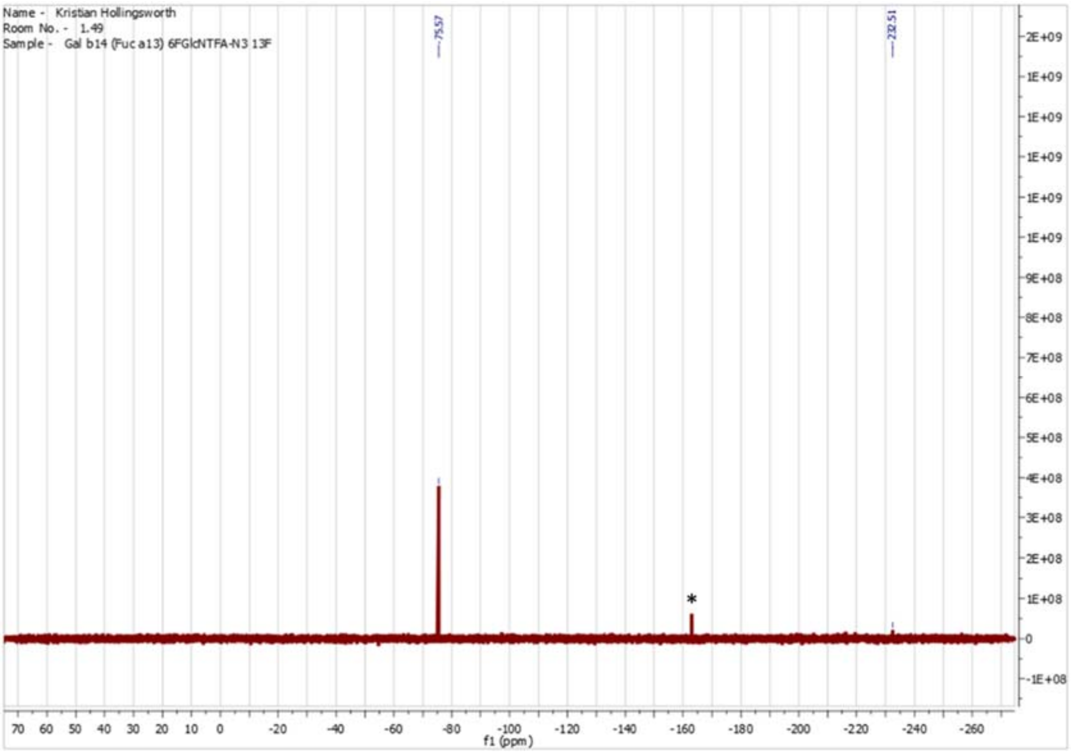

2.19.6 LeX6

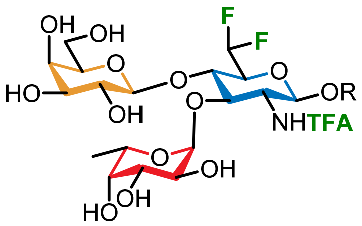

2.19.6.1 Supplementary Figure 212. ^{1}H NMR spectrum for compound **LeX6**

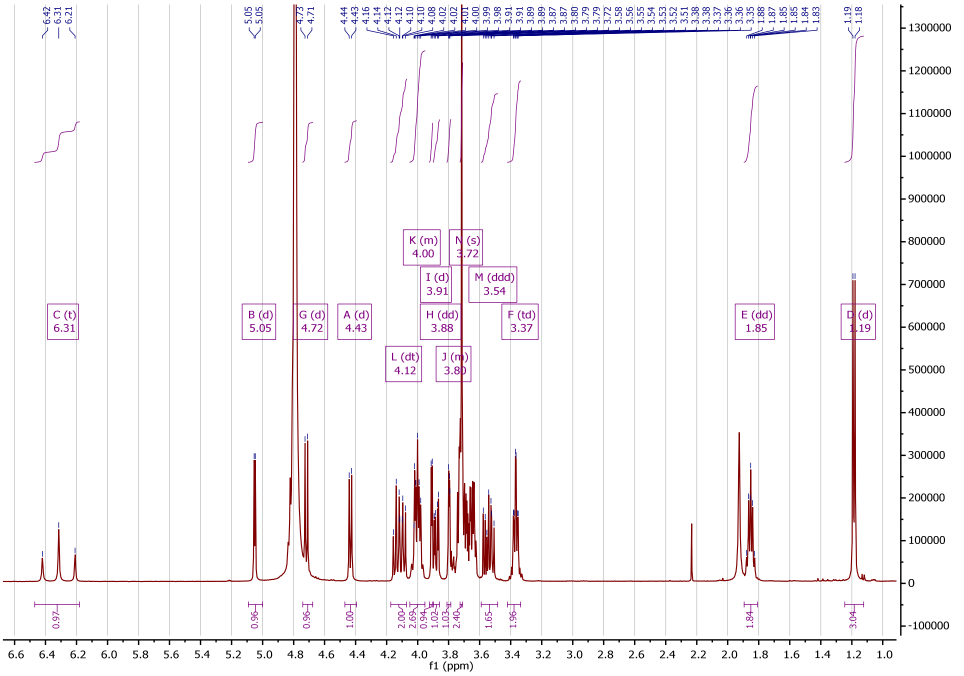

2.19.6.2 Supplementary Figure 213. ^{13}C NMR spectrum for compound **LeX6**

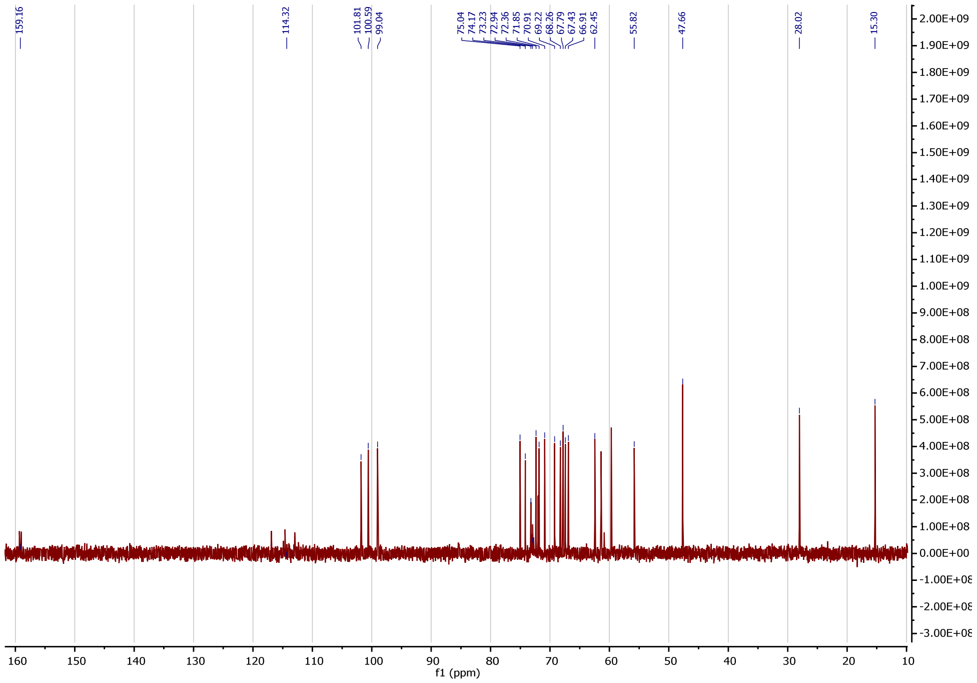

2.19.6.3 Supplementary Figure 214. $^{19}F\{1H\}$ NMR (Insert: ^{19}F NMR expansion) spectrum for compound LeX6

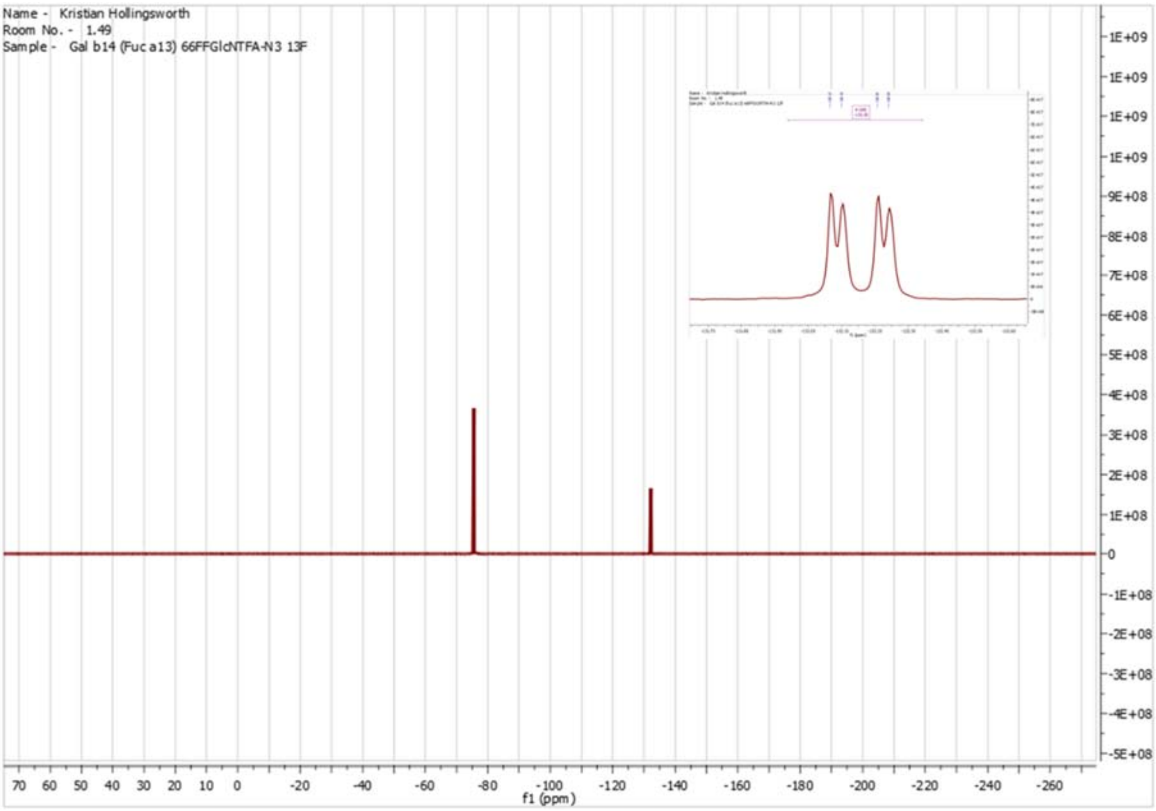

2.19.7 LeX7

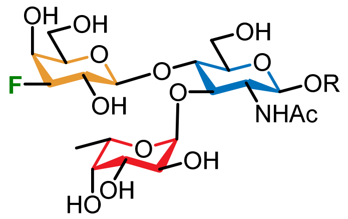

2.19.7.1 Supplementary Figure 215. ^{1}H NMR spectrum for compound **LeX7**

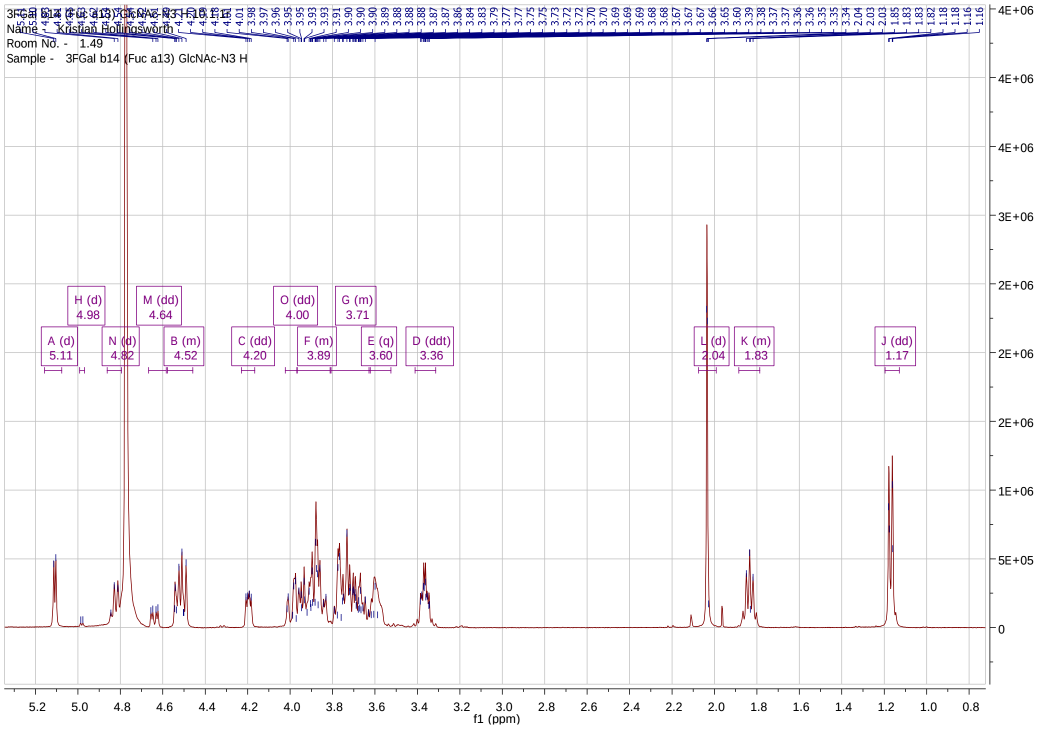

2.19.7.2 Supplementary Figure 216. ^{13}C NMR spectrum for compound **LeX7**

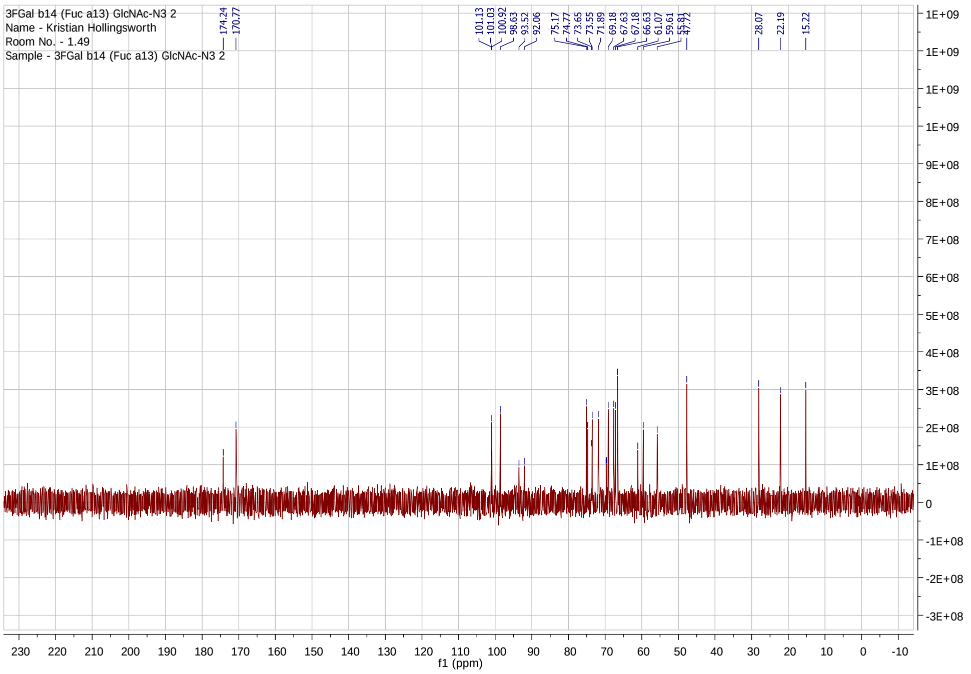

2.19.7.3 Supplementary Figure 217. $^{19}F\{1H\}$ NMR (Insert: ^{19}F NMR expansion) spectrum for compound **LeX7**

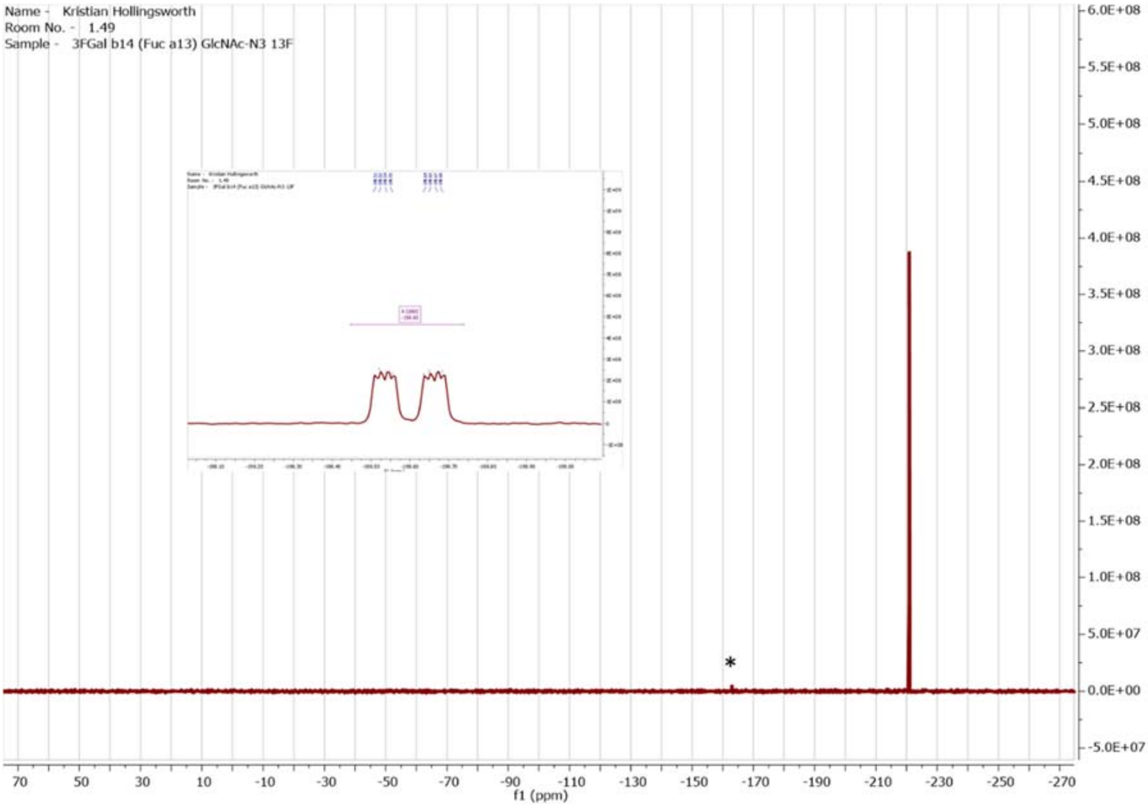

2.19.8 LeX8

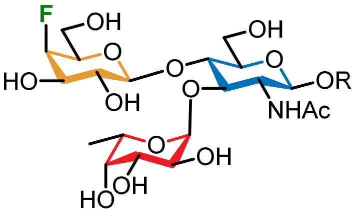

2.19.8.1 Supplementary Figure 218. ^{1}H NMR spectrum for compound **LeX8**

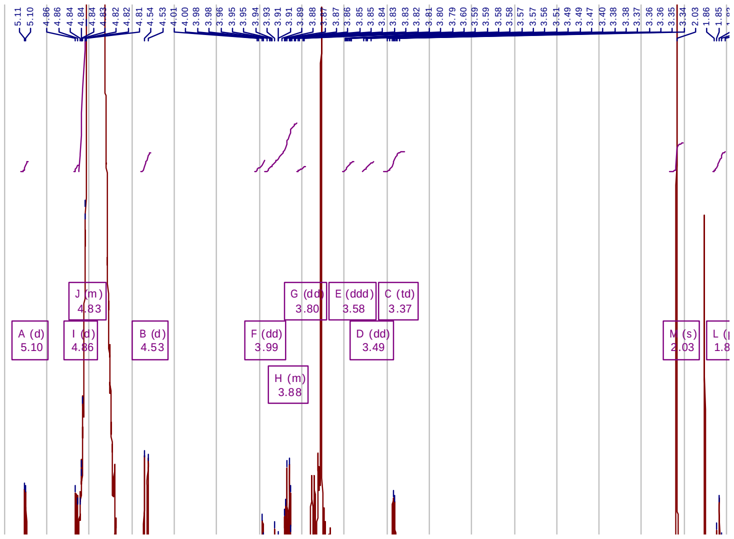

2.19.8.2 Supplementary Figure 219. ^{13}C NMR spectrum for compound **LeX8**

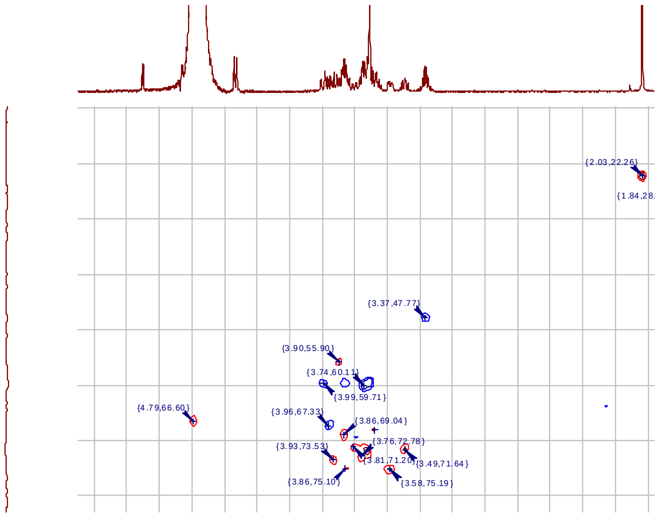

2.19.8.3 Supplementary Figure 220. $^{19}F\{1H\}$ NMR (Insert: ^{19}F NMR expansion) spectrum for compound **LeX8**

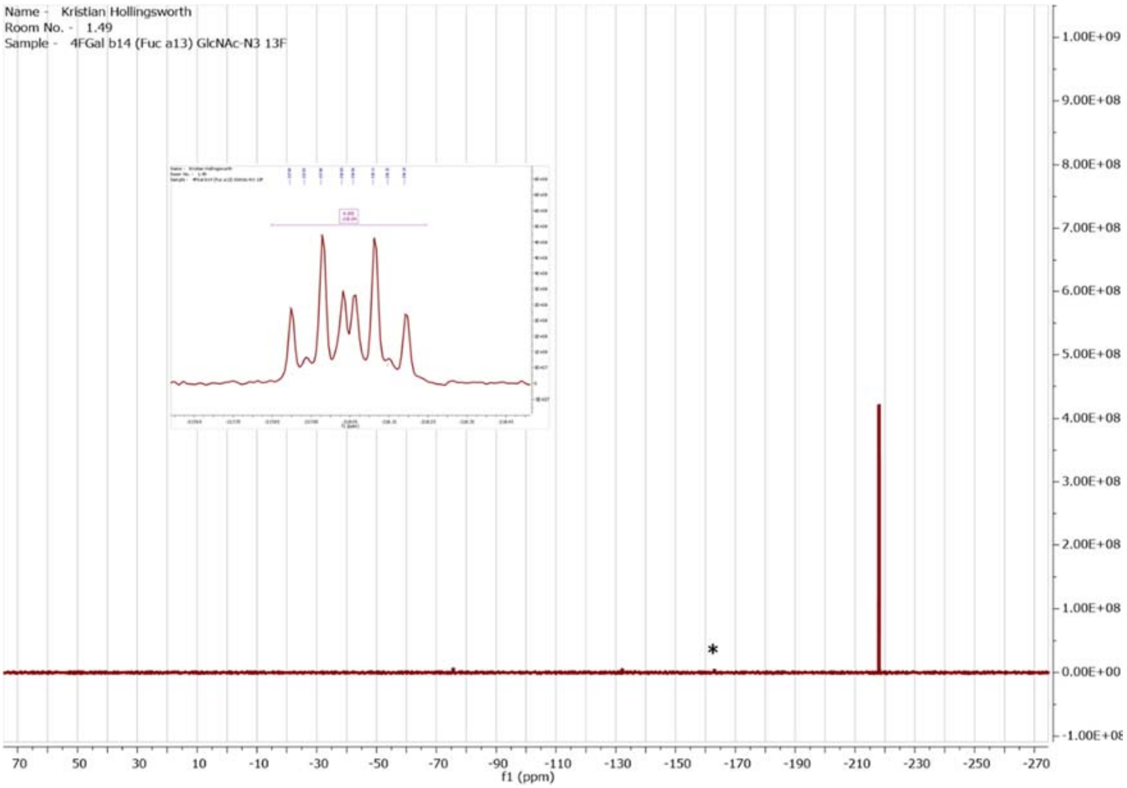

2.19.9 LeX9

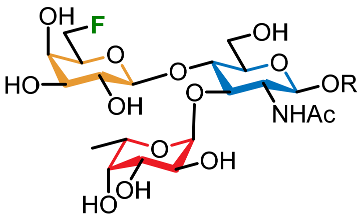

2.19.9.1 Supplementary Figure 221. ^{1}H NMR spectrum for compound **LeX9**

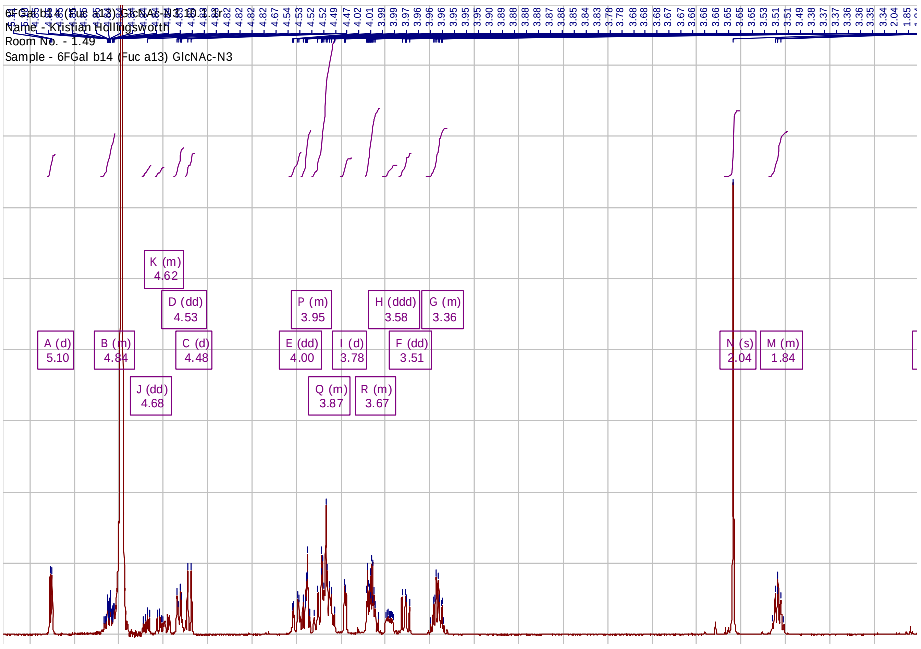

2.19.9.2 Supplementary Figure 222. HSQC NMR spectrum for compound **LeX9**

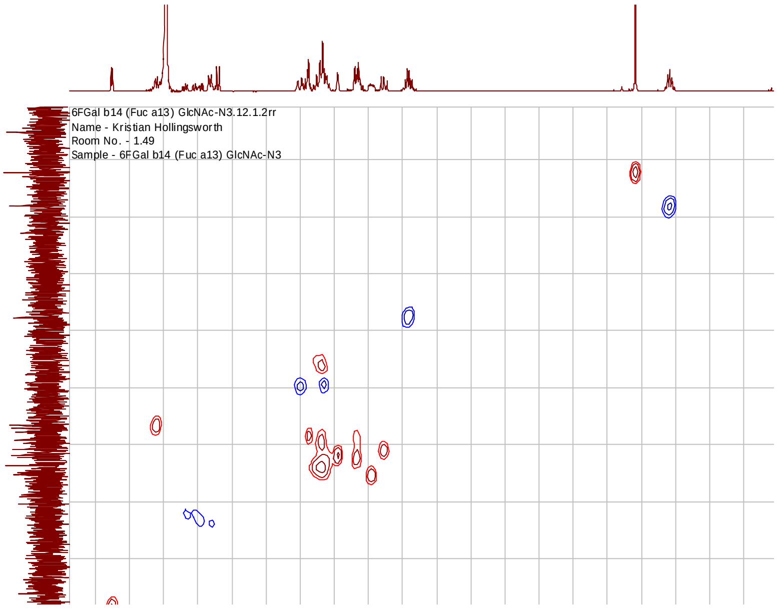

2.19.9.3 Supplementary Figure 223. $^{19}F\{1H\}$ NMR9

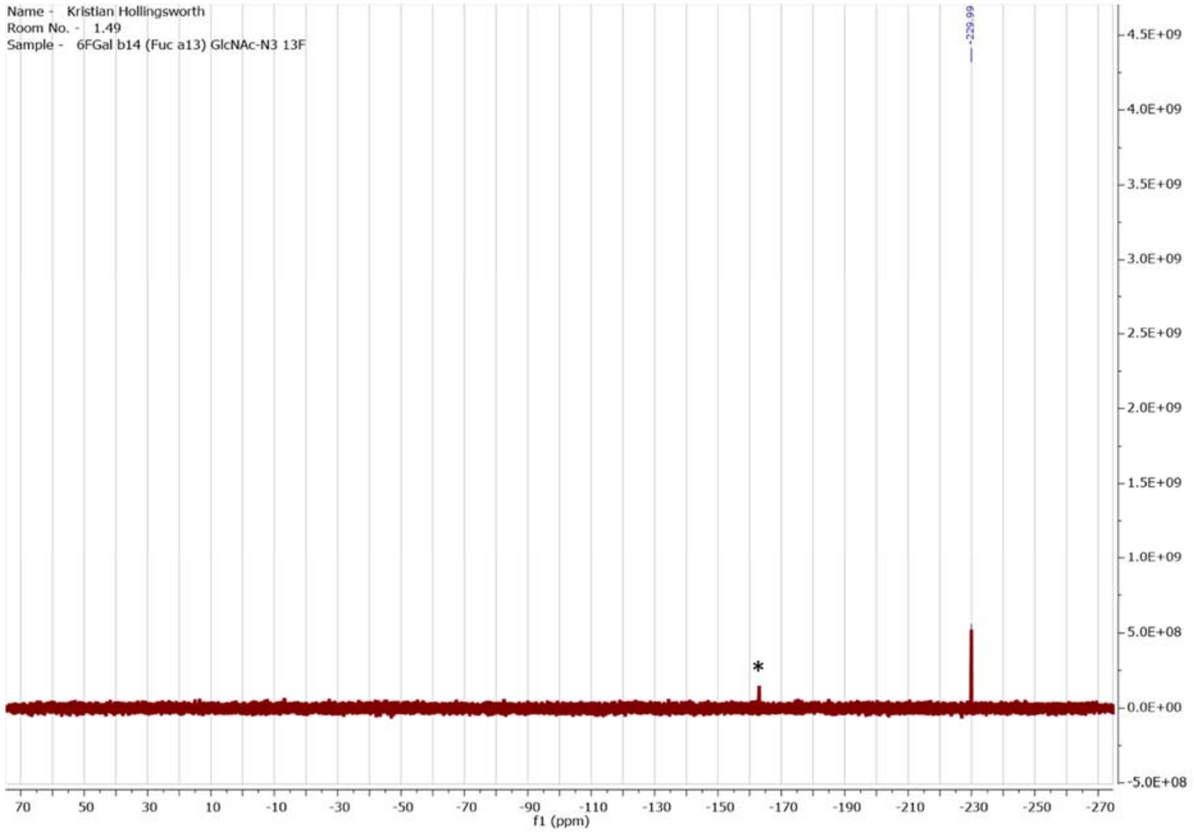

2.19.10 LeX10

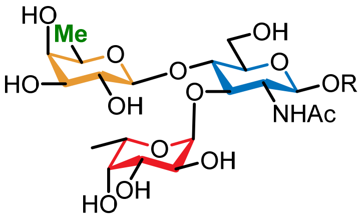

2.19.10.1 Supplementary Figure 224. ^{1}H NMR spectrum for compound **LeX10**

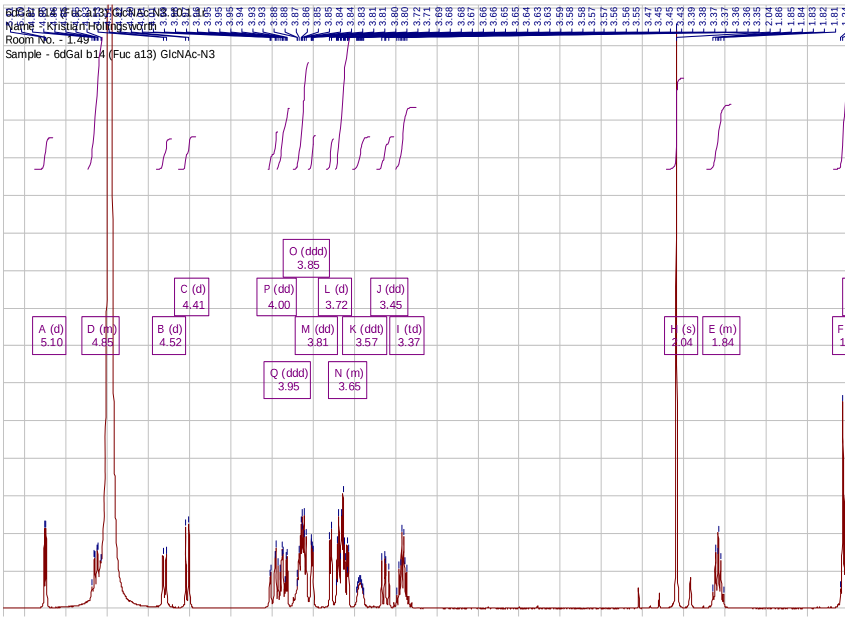

2.19.10.2 Supplementary Figure 225. ^{13}C NMR spectrum for compound **LeX10**

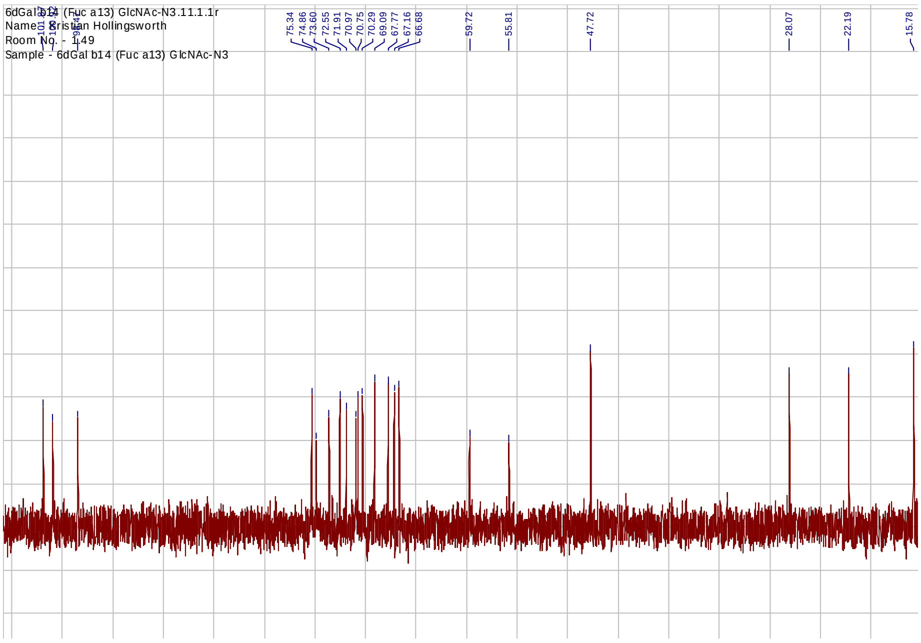

2.19.11 LeX11

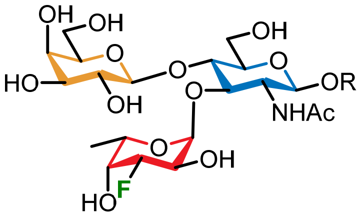

2.19.11.1 Supplementary Figure 226. ^{1}H NMR spectrum for compound **LeX11**

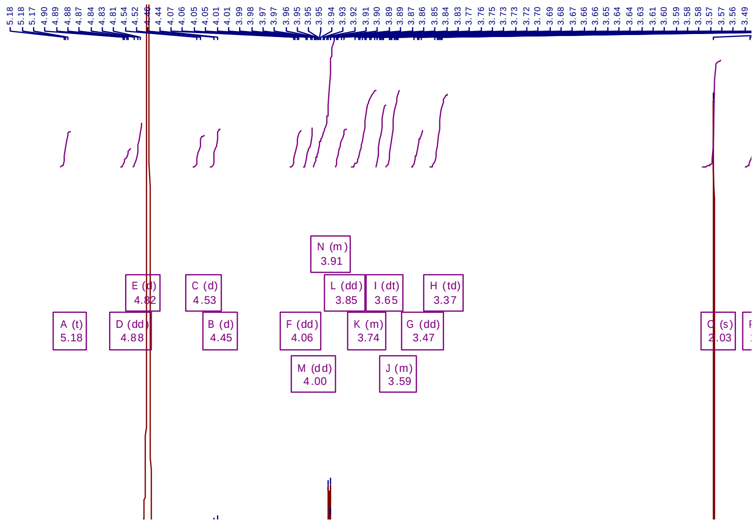

2.19.11.2 Supplementary Figure 227. ^{13}C NMR spectrum for compound **LeX11**

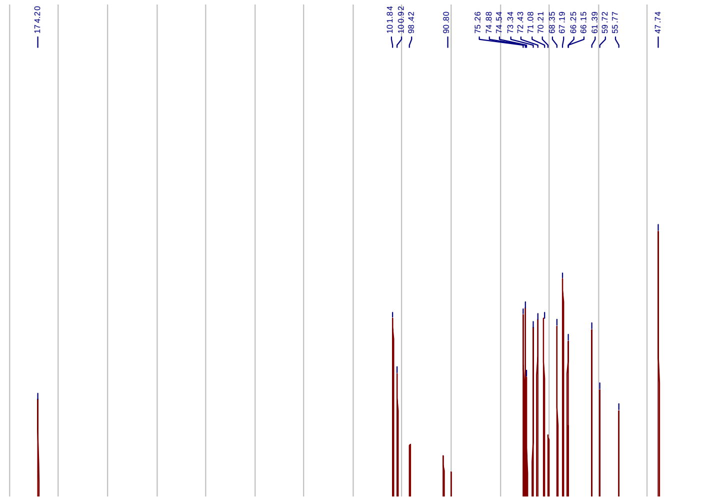

2.19.11.3 Supplementary Figure 228. $^{19}F\{1H\}$ NMR (Insert: ^{19}F NMR expansion) spectrum for compound LeX11

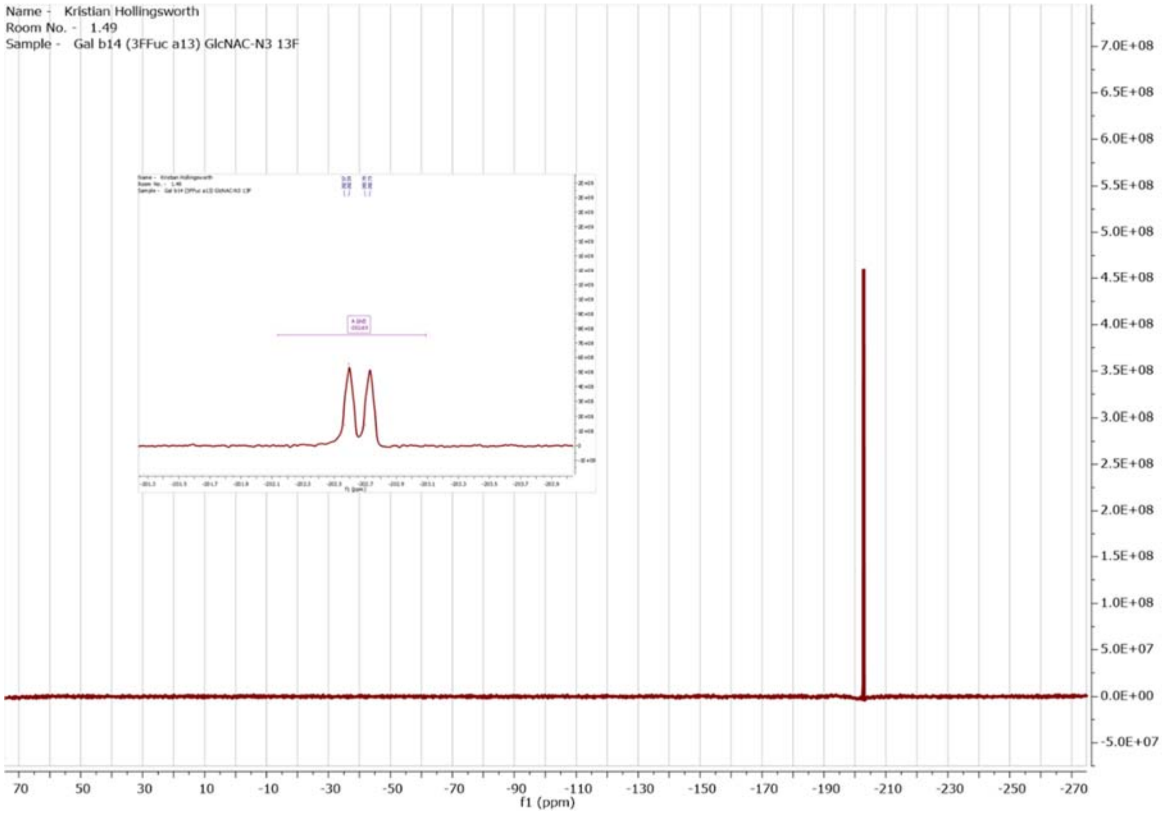

2.19.12 LeX12

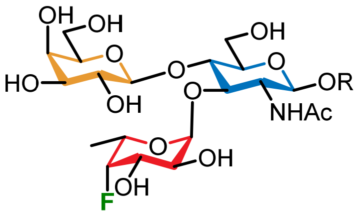

2.19.12.1 Supplementary Figure 229. ^{1}H NMR spectrum for compound **LeX12**

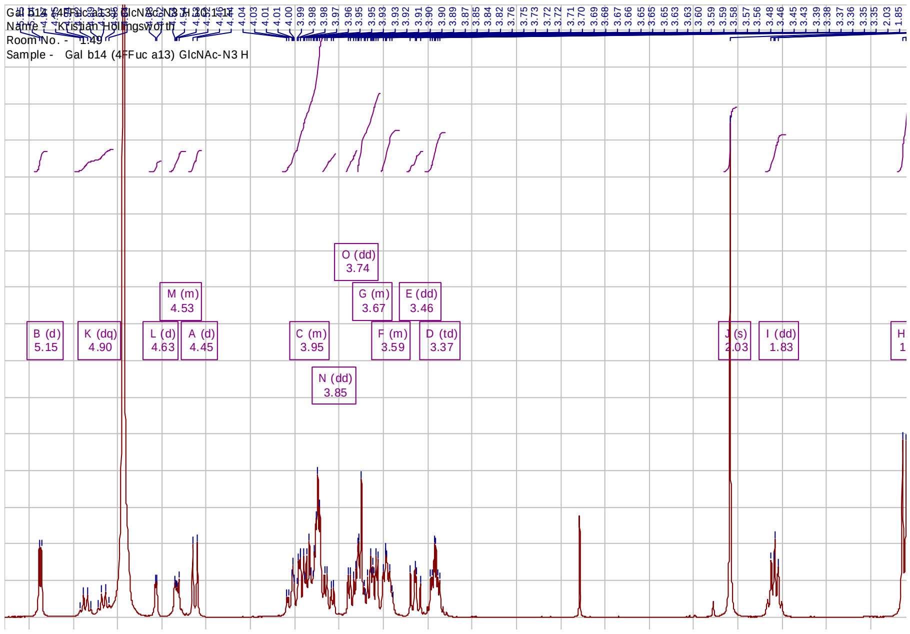

2.19.12.2 Supplementary Figure 230. ^{13}C NMR spectrum for compound **LeX12**

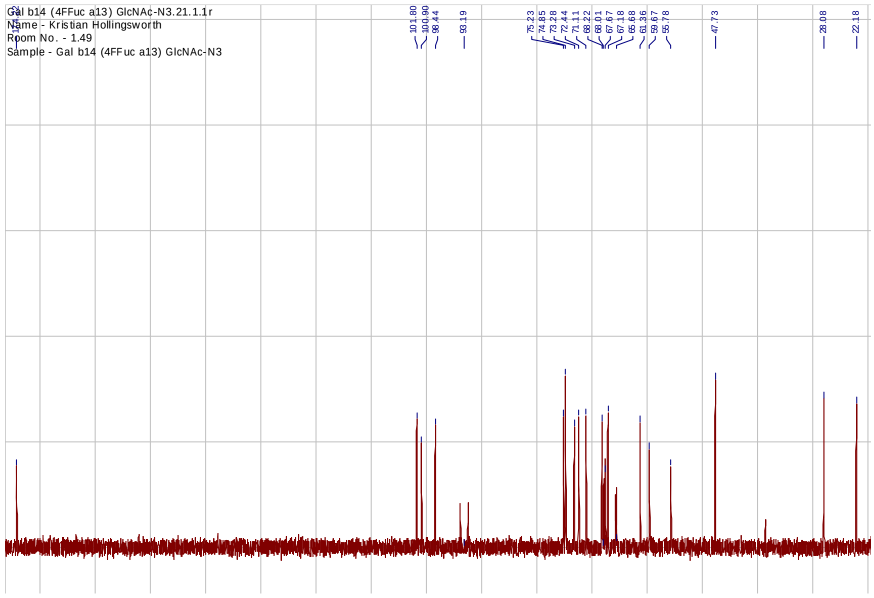

2.19.12.3 Supplementary Figure 231. $^{19}F\{1H\}$ NMR (Insert: ^{19}F NMR expansion) spectrum for compound LeX12

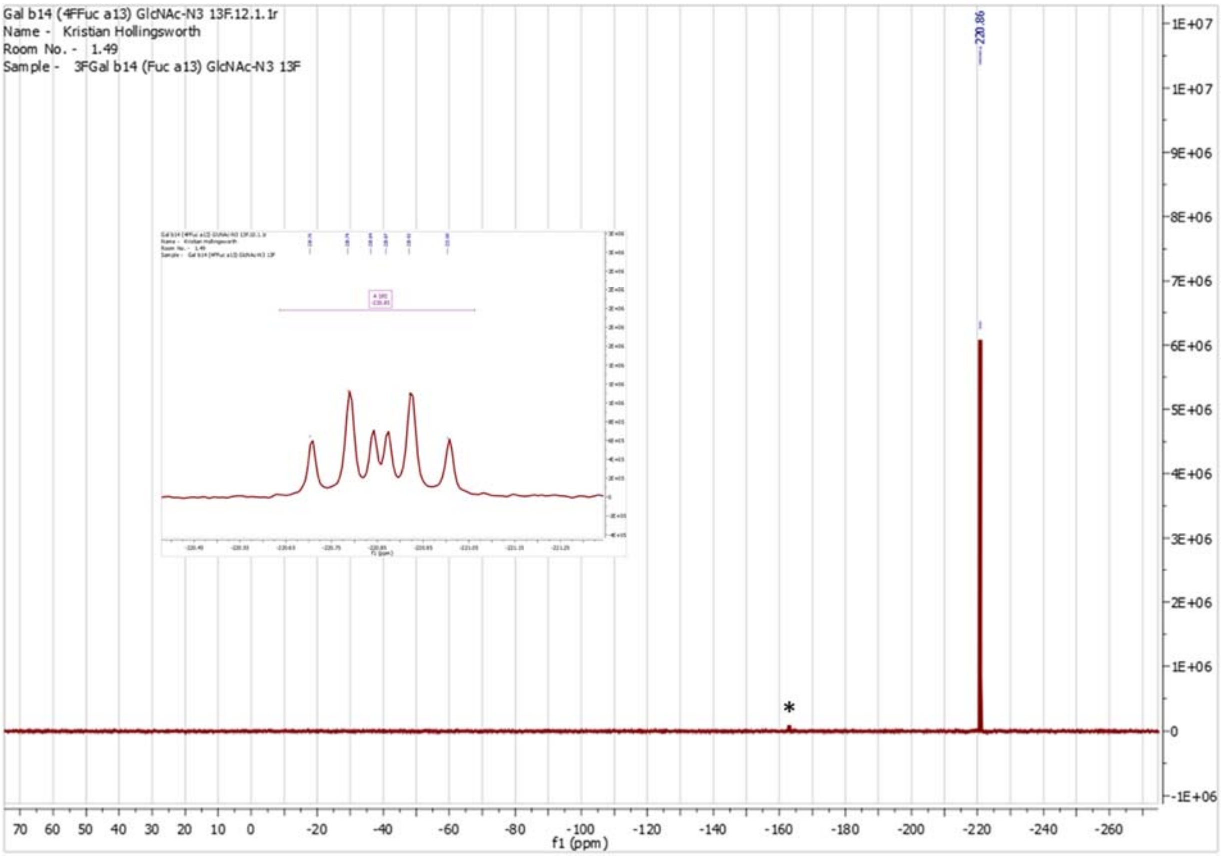

2.19.13 LeX13

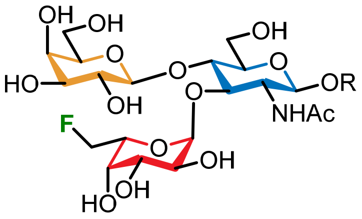

2.19.13.1 Supplementary Figure 232. ^{1}H NMR spectrum for compound **LeX13**

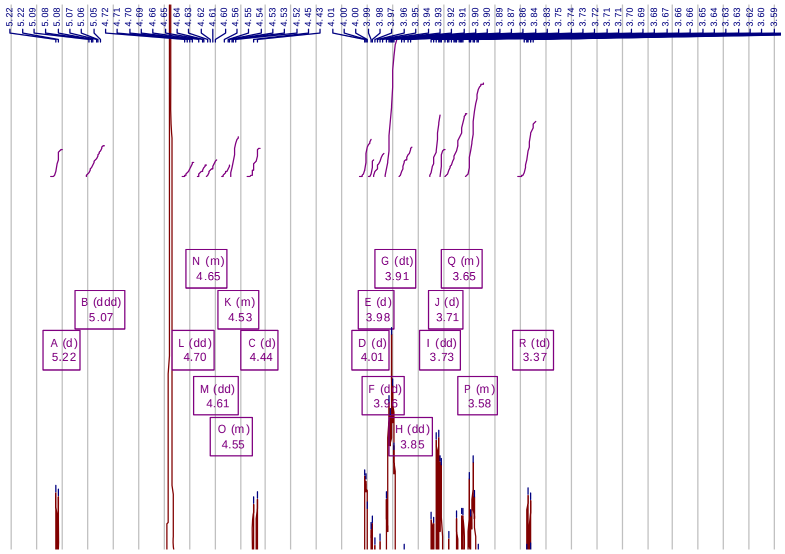

2.19.13.2 Supplementary Figure 233. ^{13}C NMR spectrum for compound **LeX13**

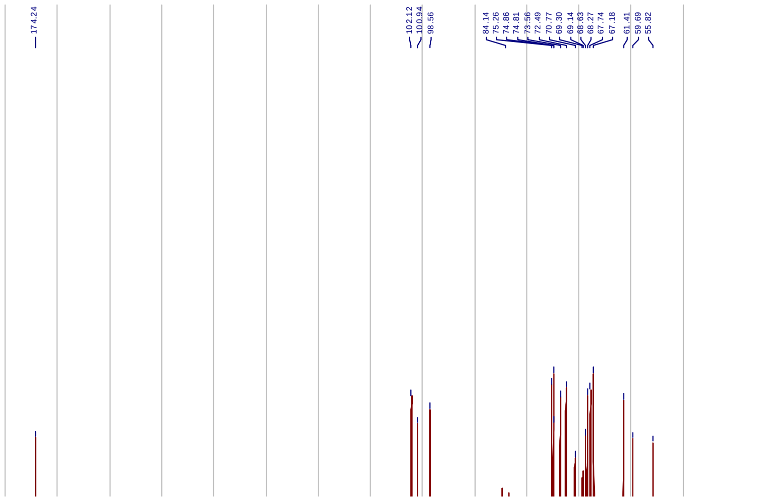

2.19.13.3 Supplementary Figure 234. $^{19}F\{1H\}$ NMR (Insert: ^{19}F NMR expansion) spectrum for compound LeX13

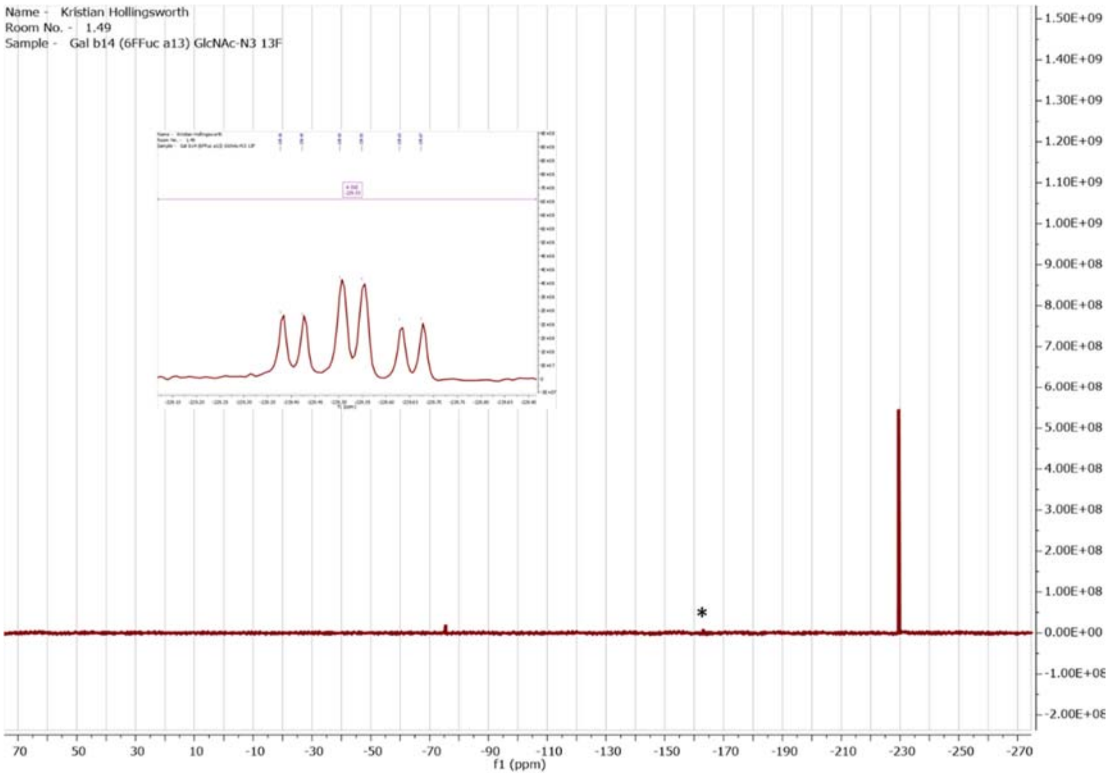

2.19.14 LeX14

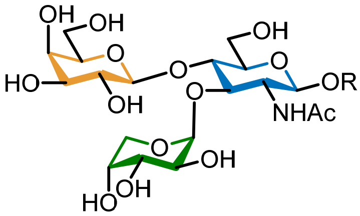

2.19.14.1 Supplementary Figure 235. ^{1}H NMR spectrum for compound **LeX14**

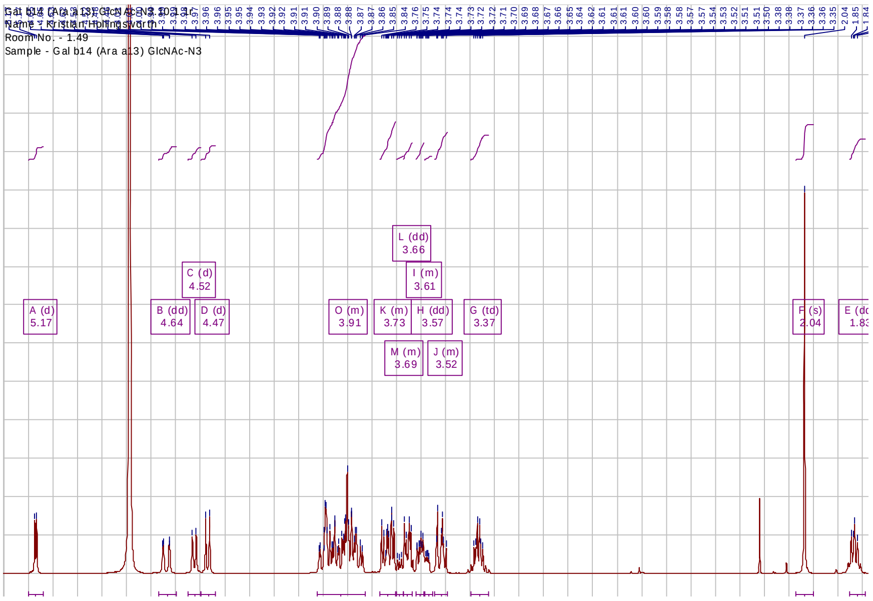

2.19.14.2 Supplementary Figure 236. ^{13}C NMR spectrum for compound **LeX14**

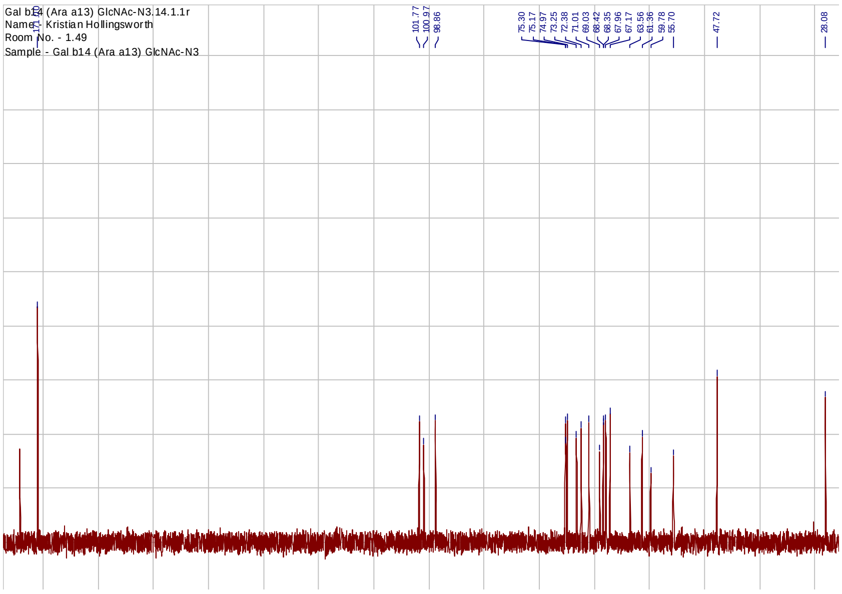

2.19.15 LeX15

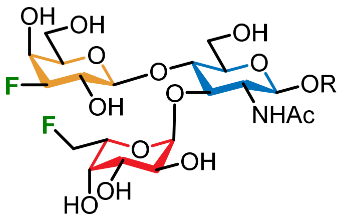

2.19.15.1 Supplementary Figure 237. ^{1}H NMR spectrum for compound **LeX15**

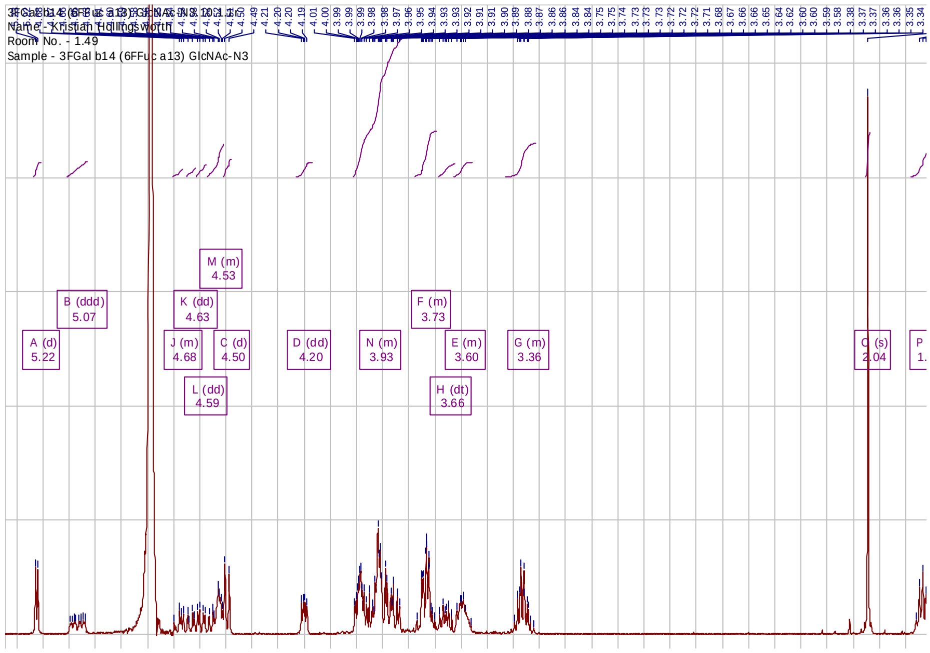

2.19.15.2 Supplementary Figure 238. ^{13}C NMR spectrum for compound **LeX15**

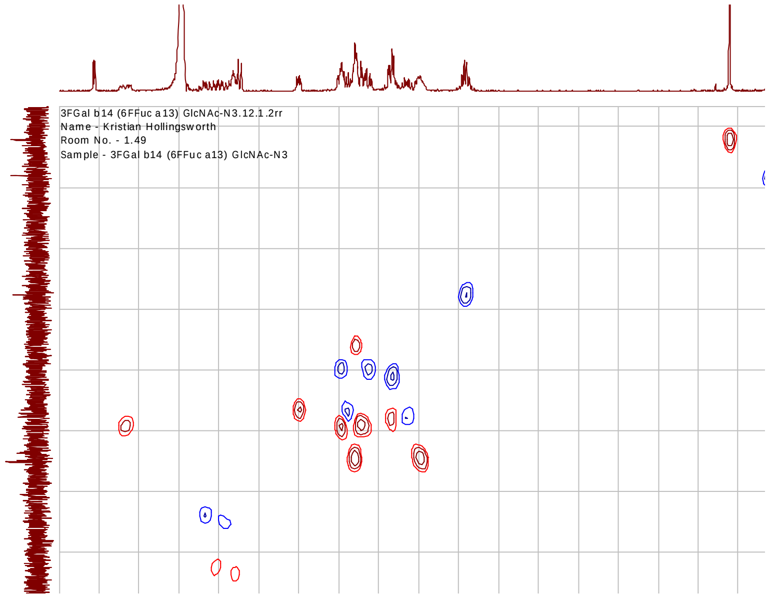

2.19.15.3 Supplementary Figure 239. $^{19}F\{1H\}$ NMR (Insert: ^{19}F NMR expansions) spectrum for compound LeX15

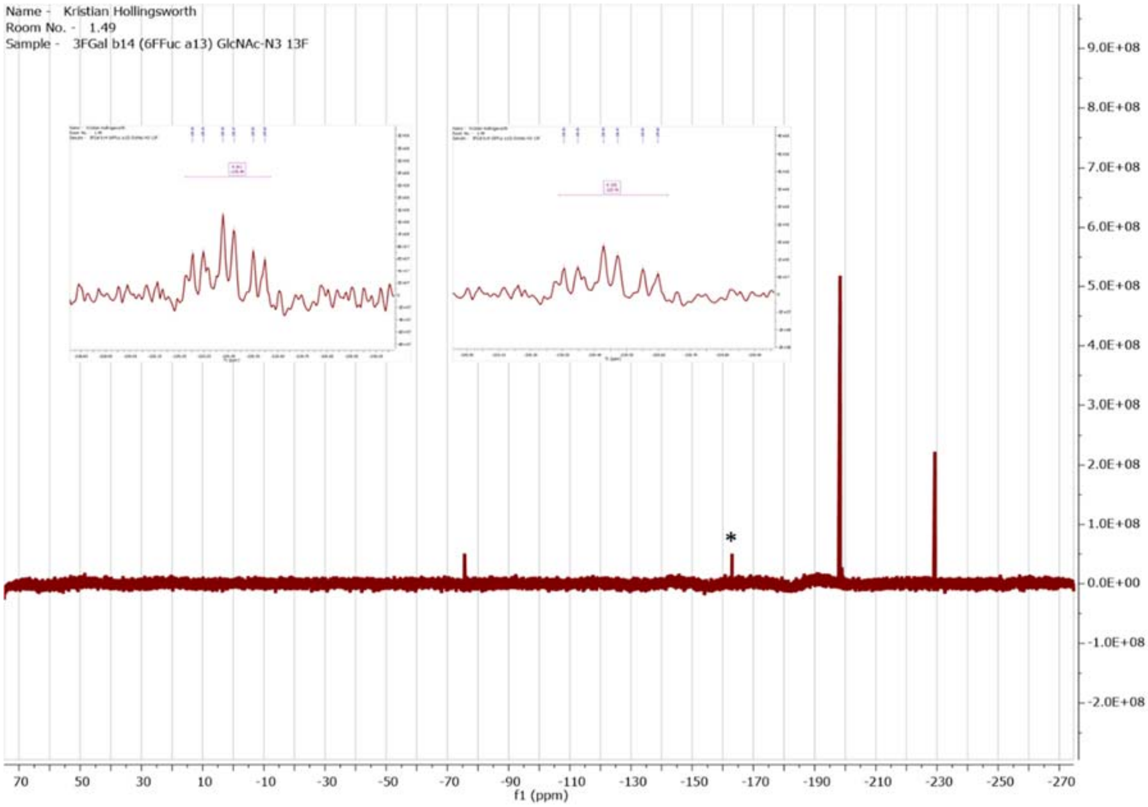

2.19.16 LeX16

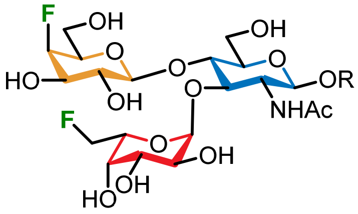

2.19.16.1 Supplementary Figure 240. ^{1}H NMR spectrum for compound **LeX16**

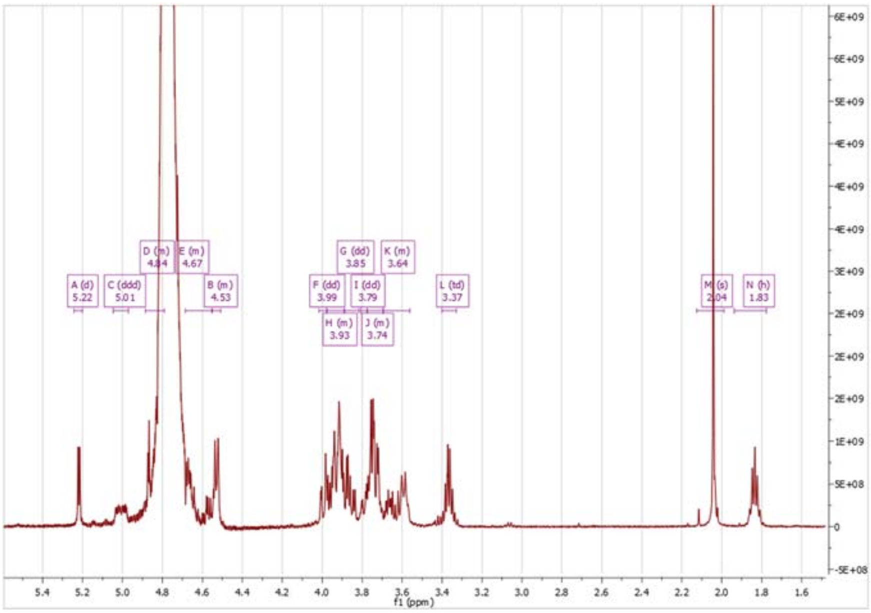

2.19.16.2 Supplementary Figure 241. ^{13}C NMR spectrum for compound **LeX16**

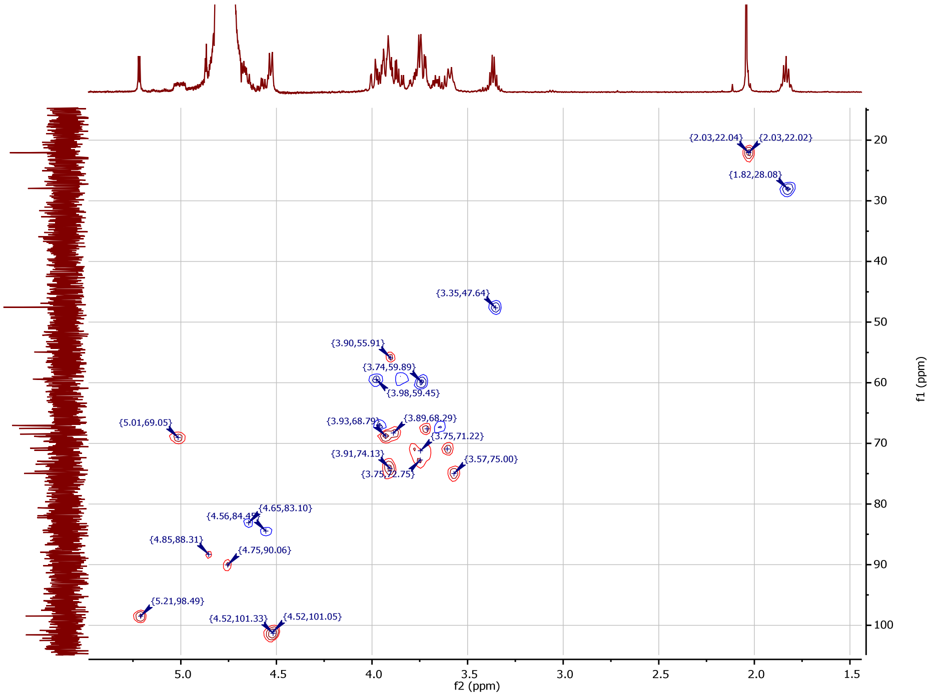

2.19.16.3 Supplementary Figure 242. $^{19}F\{1H\}$ NMR (Insert: ^{19}F NMR expansions) spectrum for compound LeX16

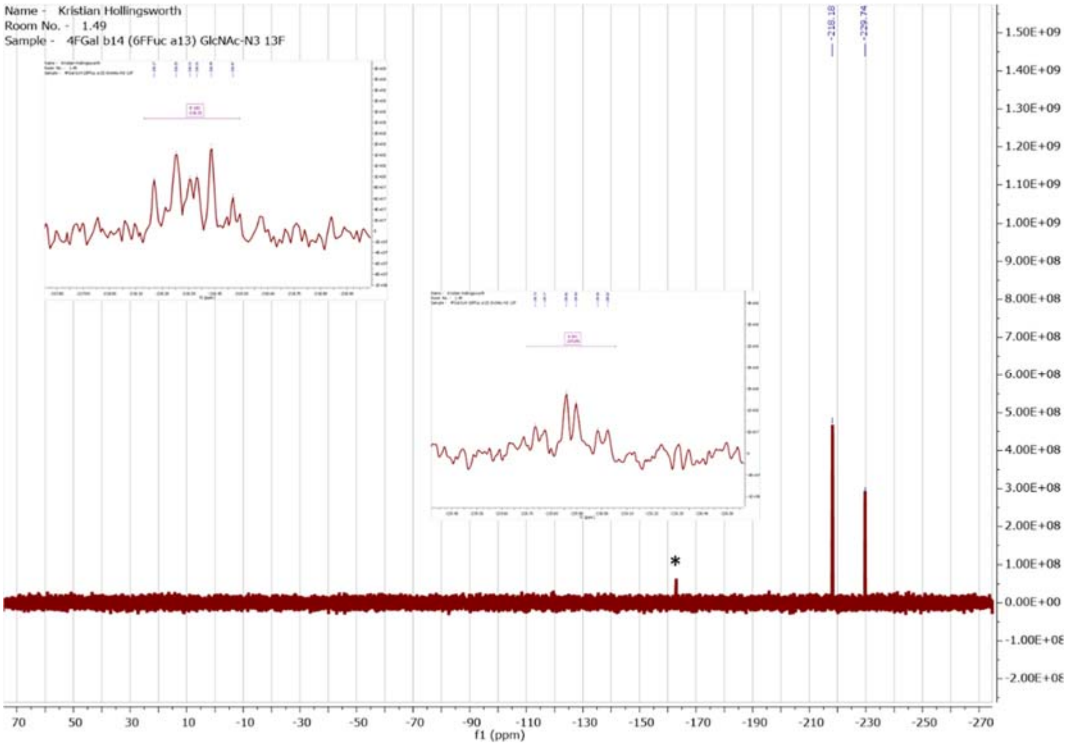

2.19.17 LeX17

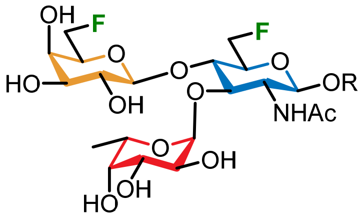

Note: contains traces of the corresponding LacNAc, which were removed after neoglycolipid formation.

2.19.17.1 Supplementary Figure 243. ^{1}H NMR spectrum for compound **LeX17**

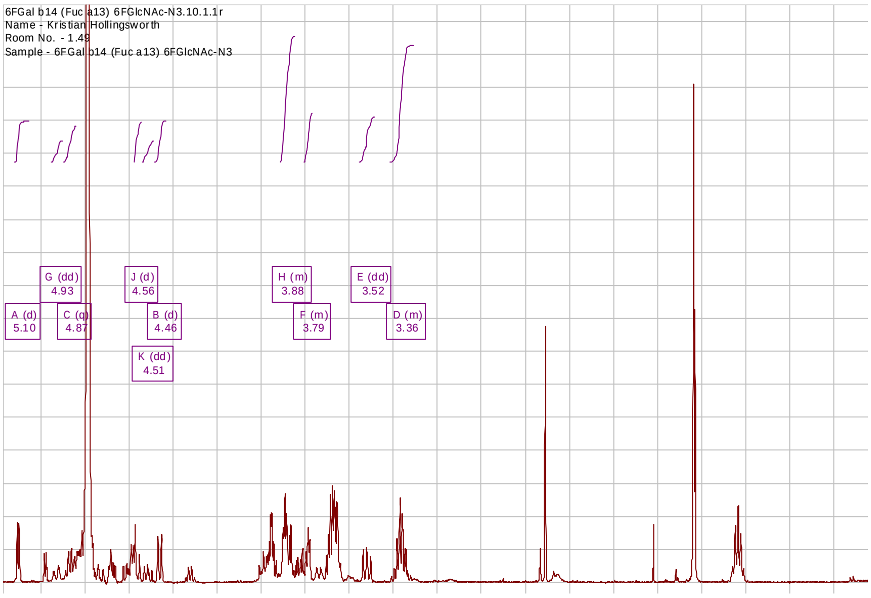

2.19.17.2 Supplementary Figure 244. ^{13}C NMR spectrum for compound **LeX17**

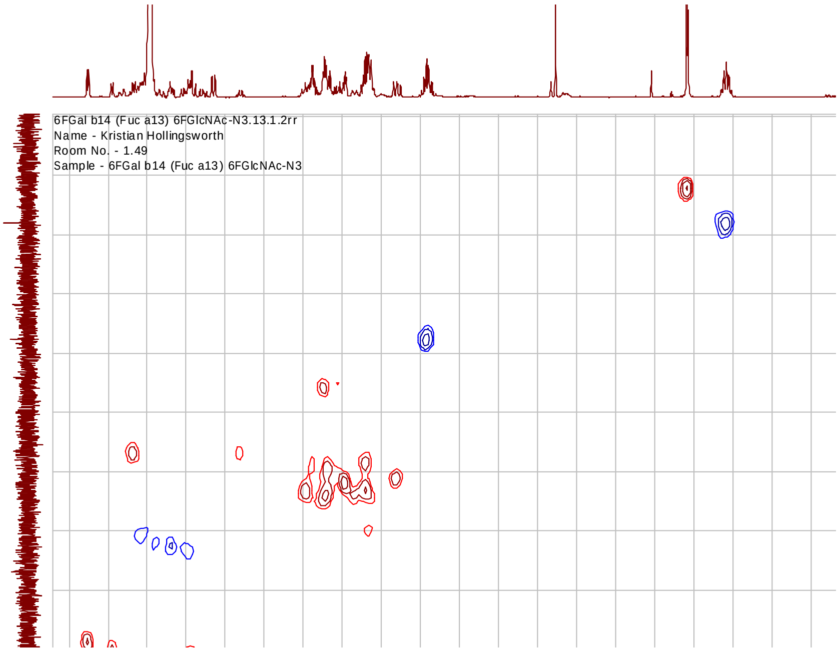

2.19.17.3 Supplementary Figure 245. $^{19}F\{1H\}$ NMR (Insert: ^{19}F NMR expansions) spectrum for compound **LeX17**

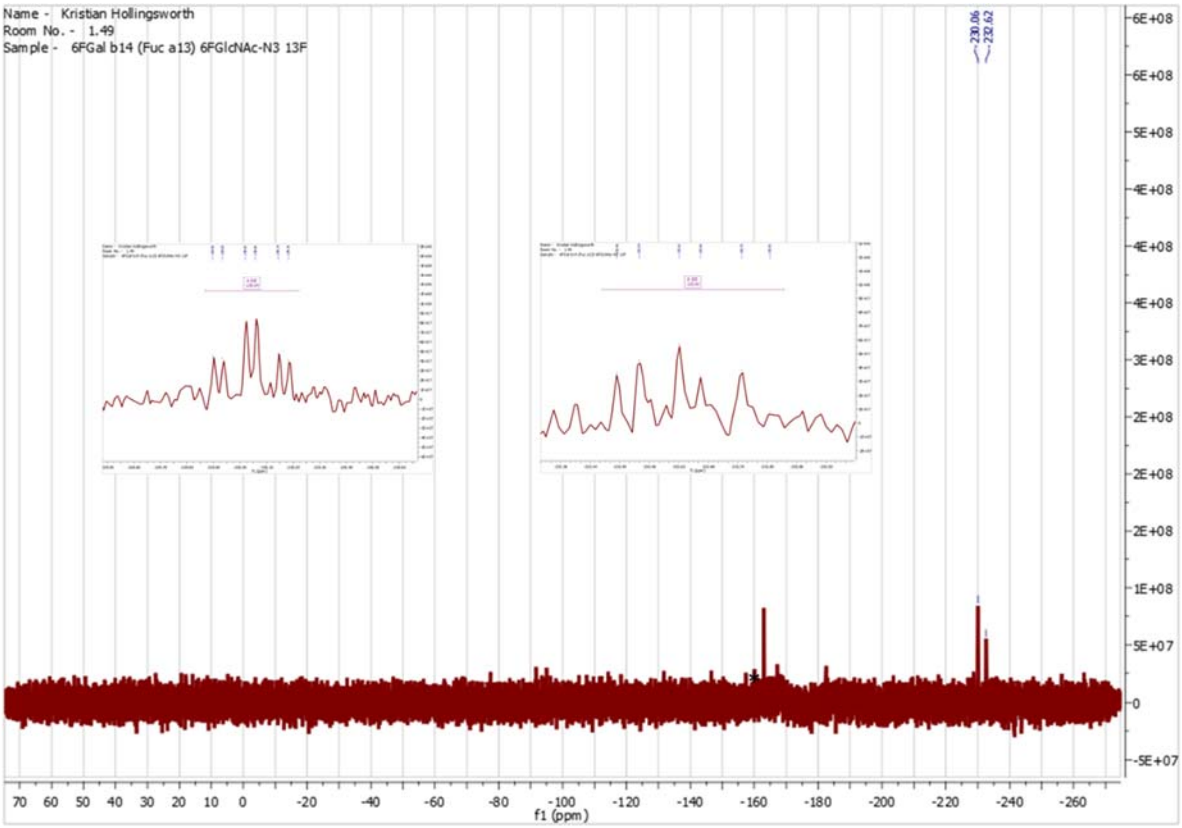

2.19.18 LeX18

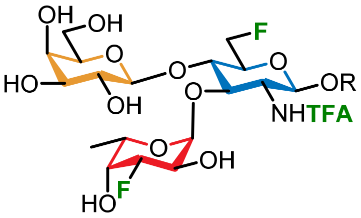

2.19.18.1 Supplementary Figure 246. ^{1}H NMR spectrum for compound **LeX18**

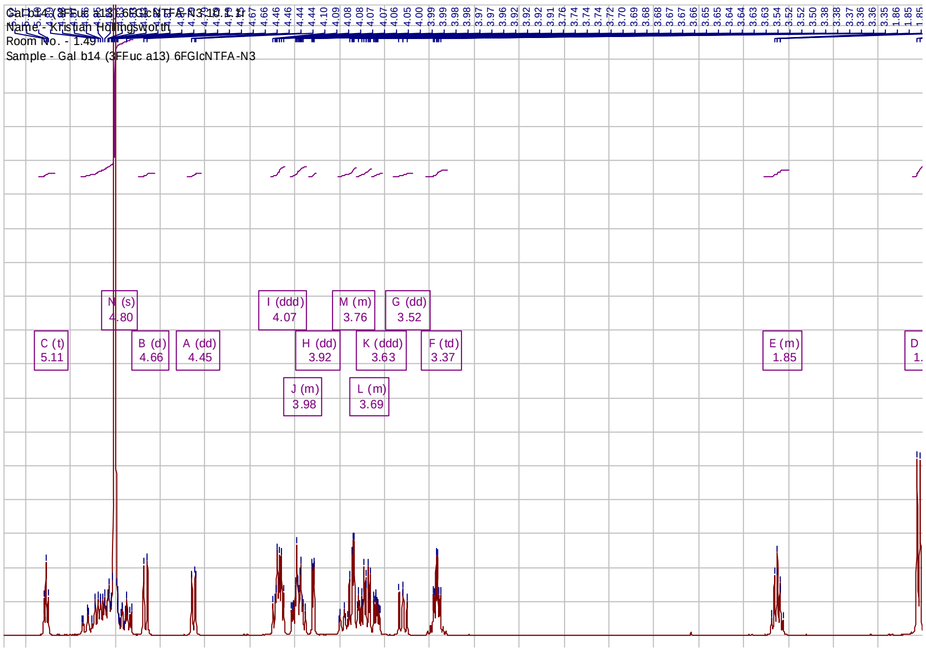

2.19.18.2 Supplementary Figure 247. ^{13}C NMR spectrum for compound **LeX18**

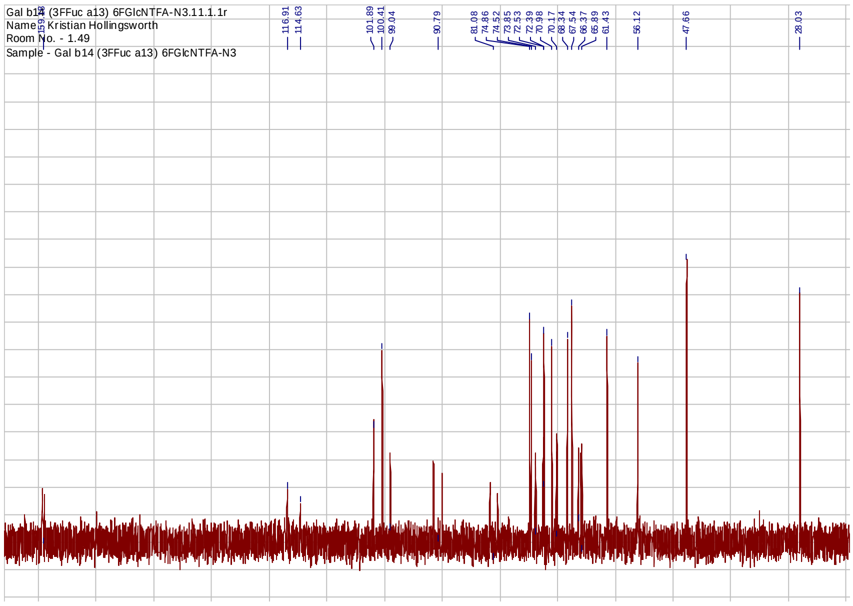

2.19.18.3 Supplementary Figure 248. $^{19}F\{^1H\}$ NMR (Insert: ^{19}F NMR expansions) spectrum for compound LeX18

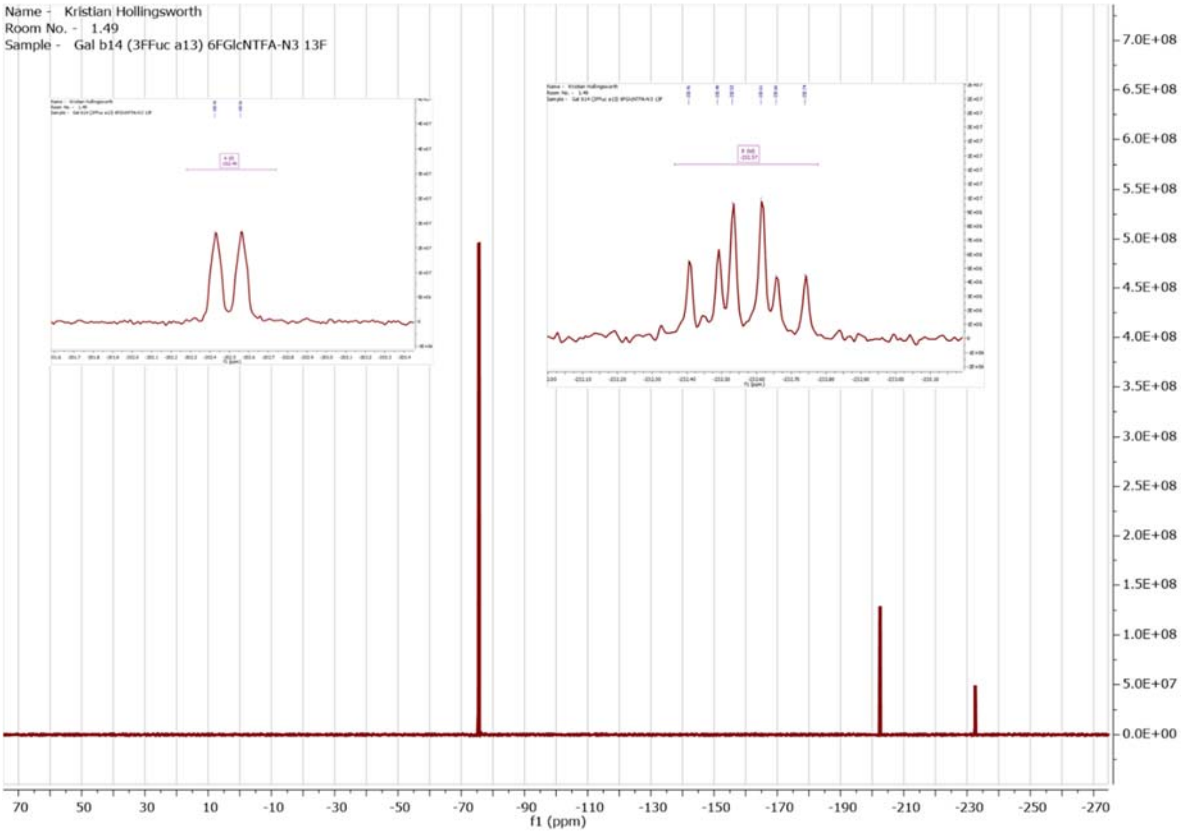

2.19.19 LeX19

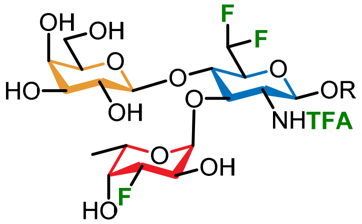

2.19.19.1 Supplementary Figure 249. ^{1}H NMR spectrum for compound **LeX19**

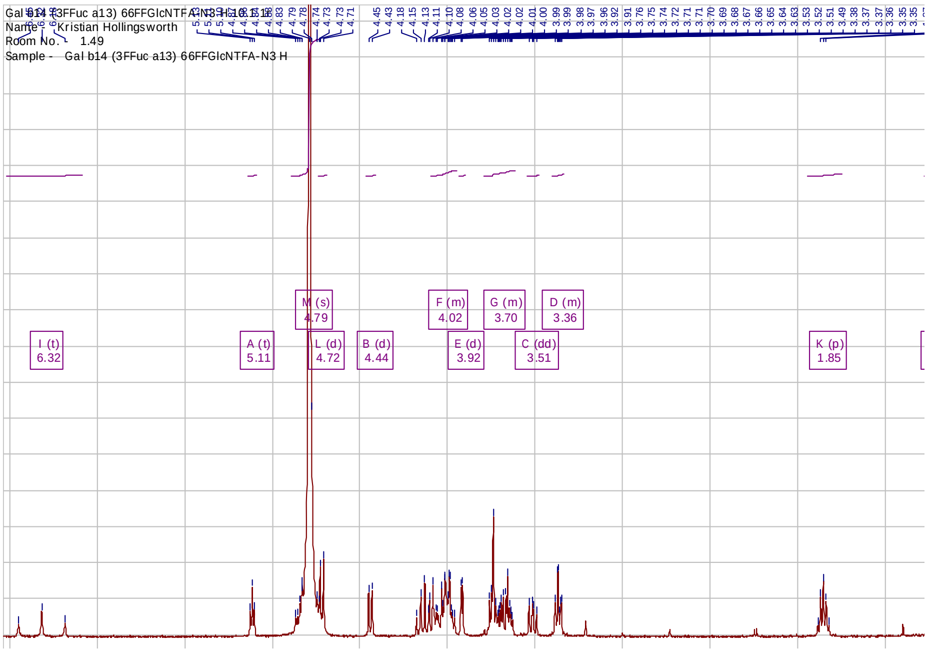

2.19.19.2 Supplementary Figure 250. ^{13}C NMR spectrum for compound **LeX19**

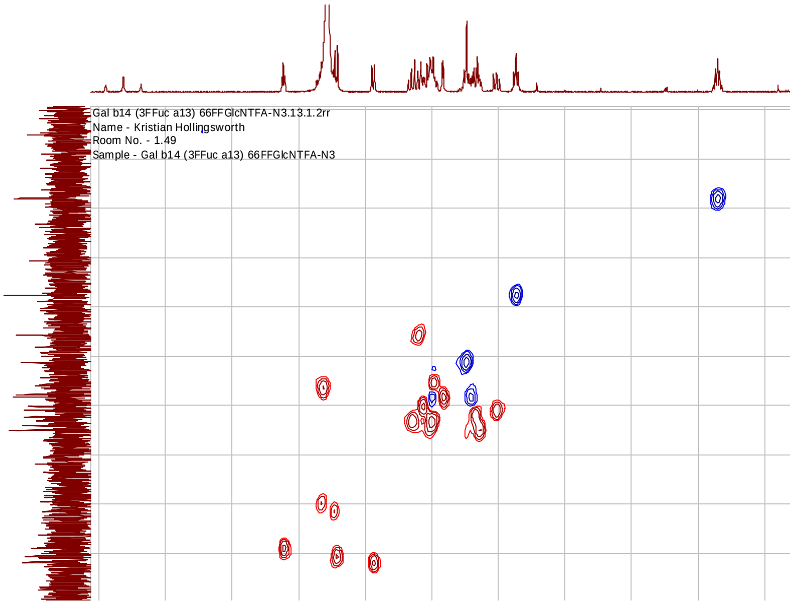

2.19.19.3 Supplementary Figure 251. $^{19}F\{1H\}$ NMR (Insert: ^{19}F NMR expansions) spectrum for compound LeX19

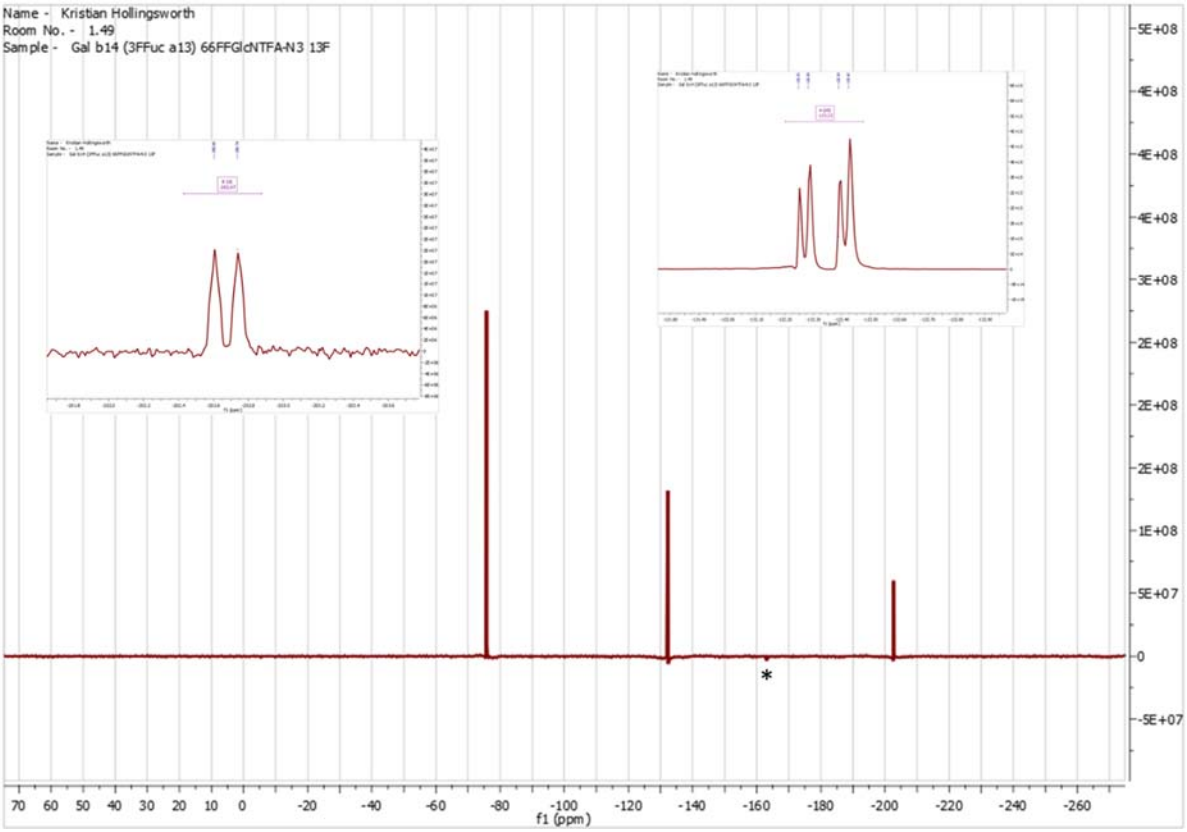

2.19.20 LeX20

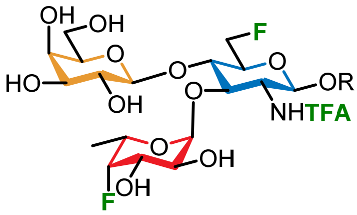

Note: contains traces of the corresponding LacNAc, which were removed after neoglycolipid formation.

2.19.20.1 Supplementary Figure 252. ^{1}H NMR spectrum for compound **LeX20**

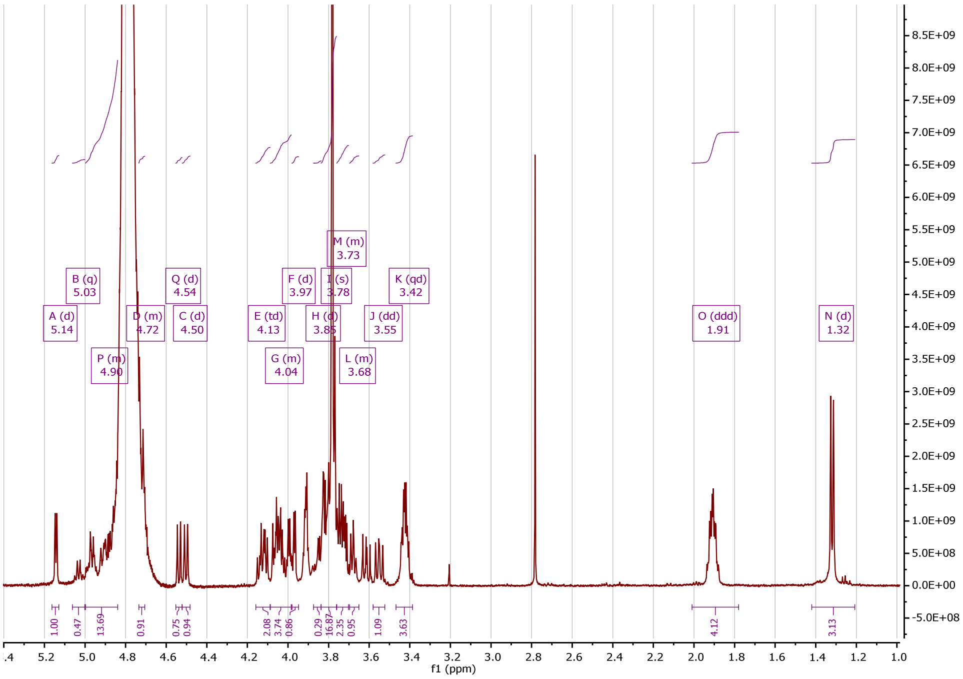

2.19.20.2 Supplementary Figure 253. ^{13}C NMR spectrum for compound **LeX20**

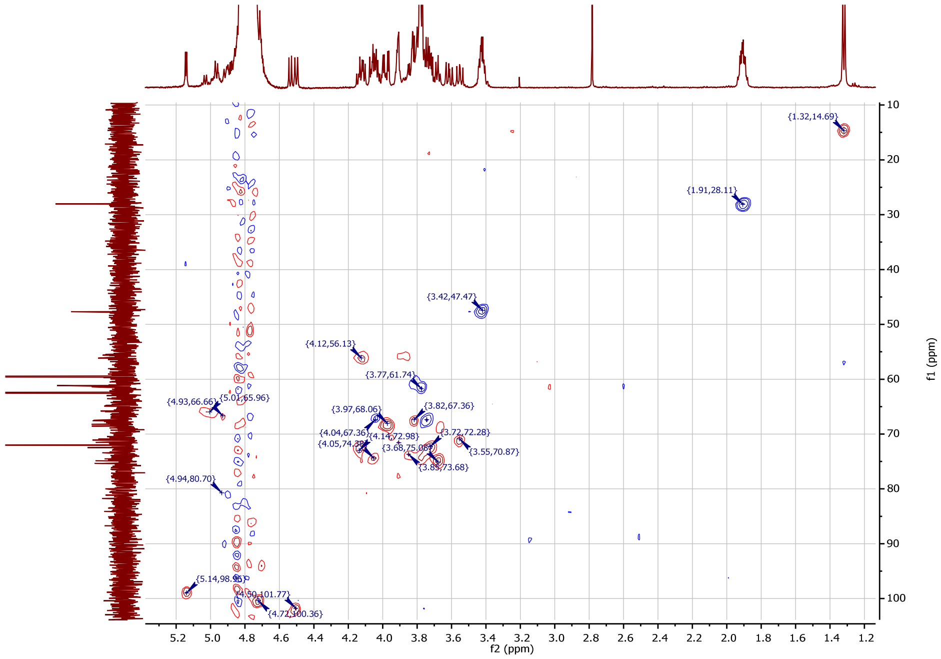

2.19.20.3 Supplementary Figure 254. $^{19}F\{1H\}$ NMR spectrum for compound **LeX20**

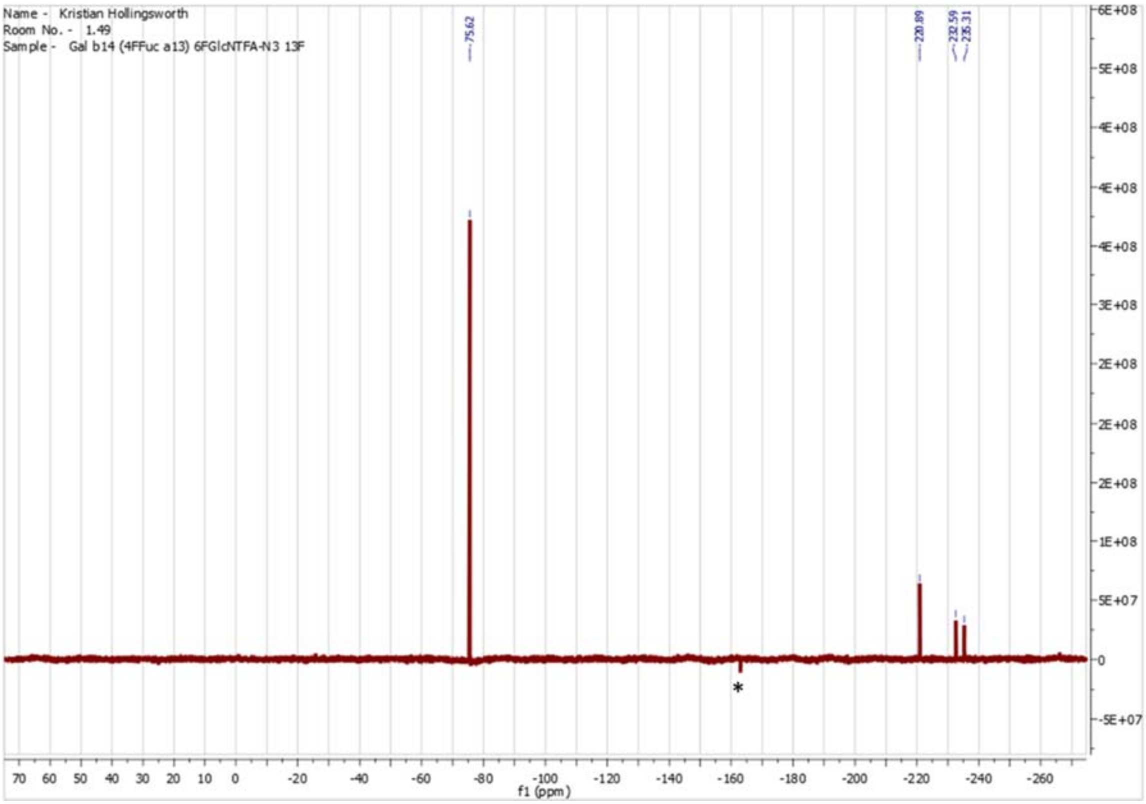

2.19.21 LeX21

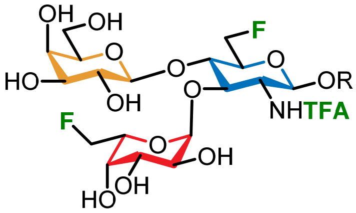

2.19.21.1 Supplementary Figure 255. ^{1}H NMR spectrum for compound **LeX21**

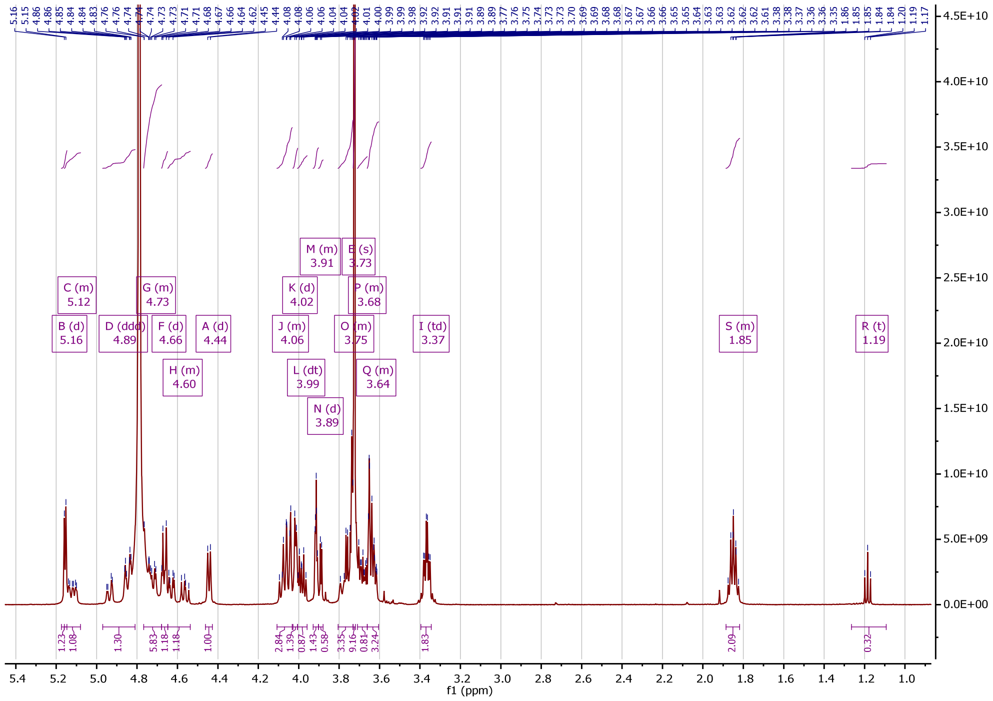

2.19.21.2 Supplementary Figure 256. ^{13}C NMR spectrum for compound **LeX21**

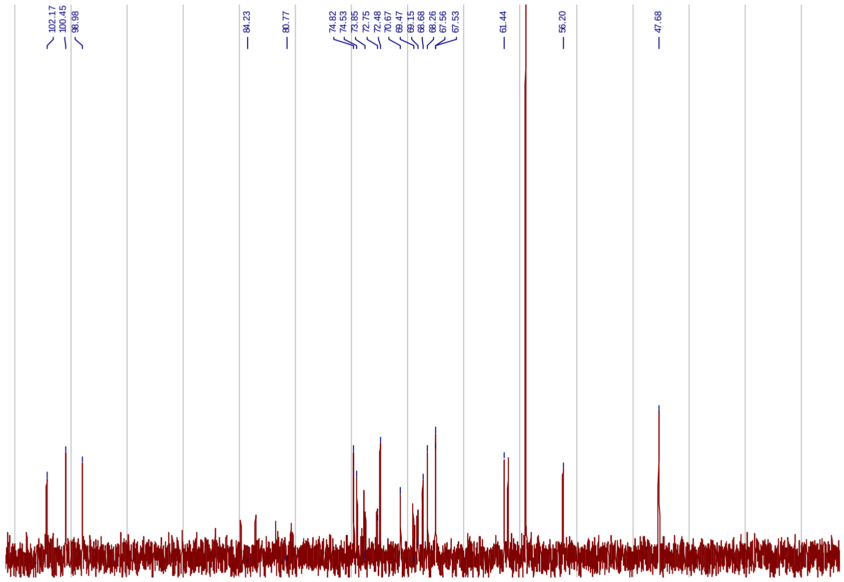

2.19.21.3 Supplementary Figure 257. $^{19}F\{^1H\}$ NMR (Insert: ^{19}F NMR expansions) spectrum for compound LeX21

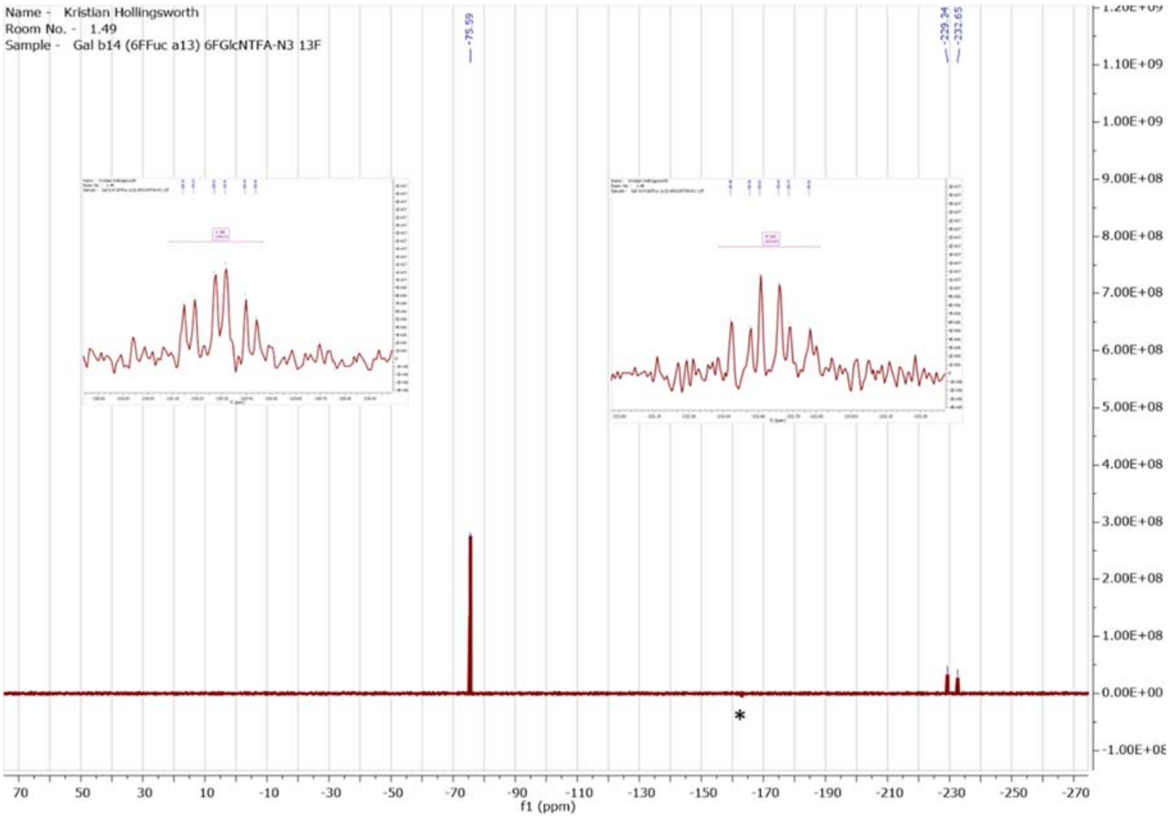

2.19.22 LeX22

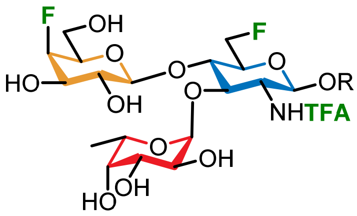

2.19.22.1 Supplementary Figure 258. ^{1}H NMR spectrum for compound **LeX22**

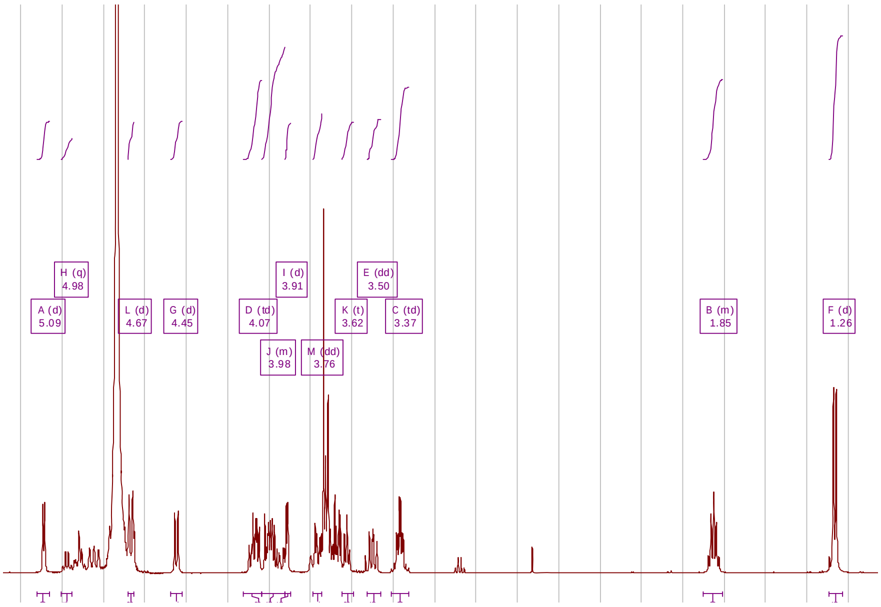

2.19.22.2 Supplementary Figure 259. ^{13}C NMR spectrum for compound **LeX22**

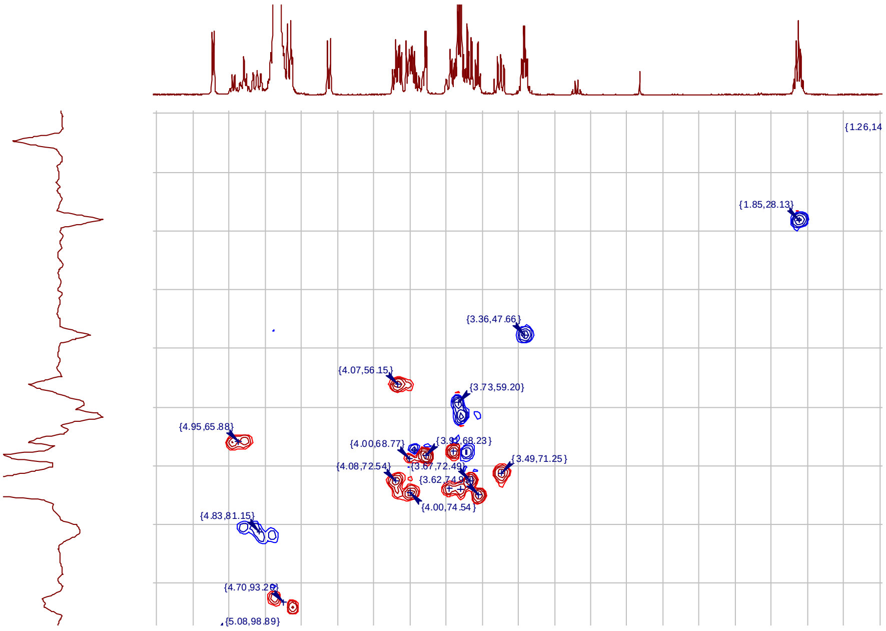

2.19.22.3 Supplementary Figure 260. $^{19}F\{1H\}$ NMR spectrum for compound LeX22

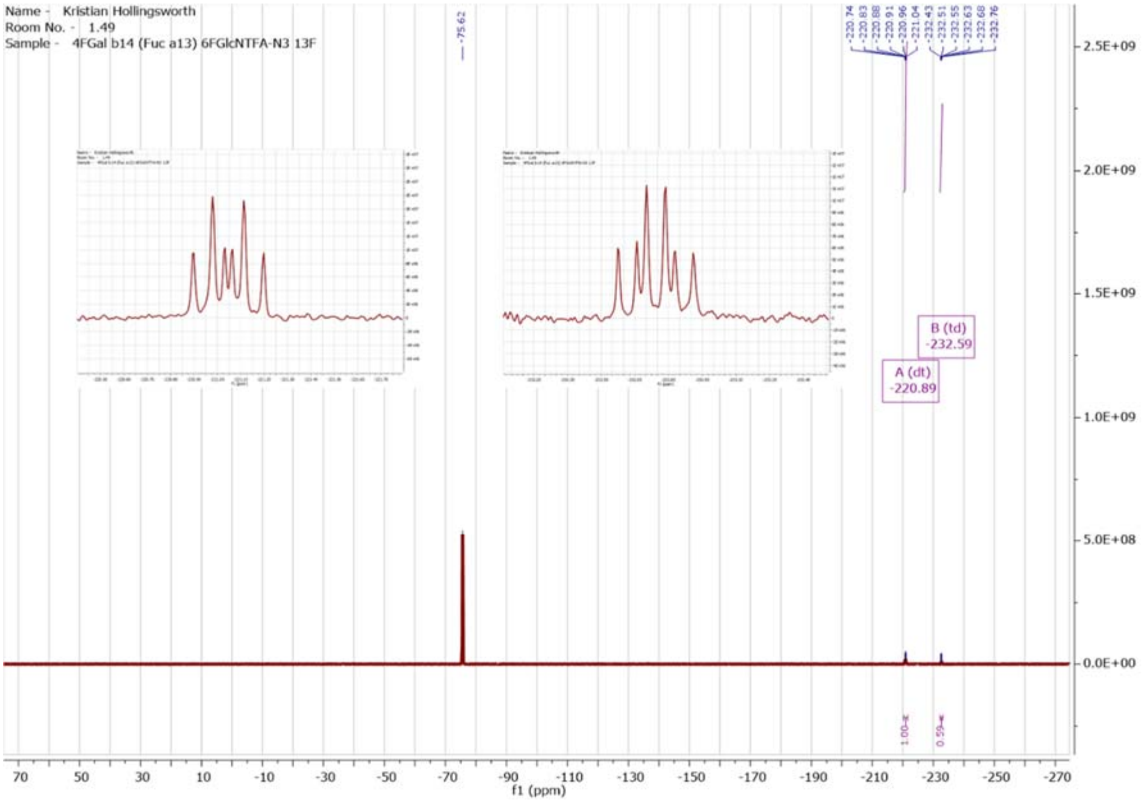

2.19.23 LeX23

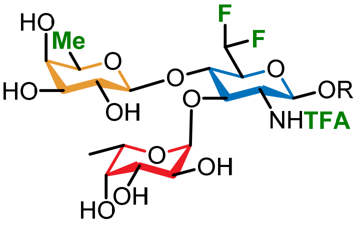

2.19.23.1 Supplementary Figure 261. ¹H NMR spectrum for compound **LeX23**

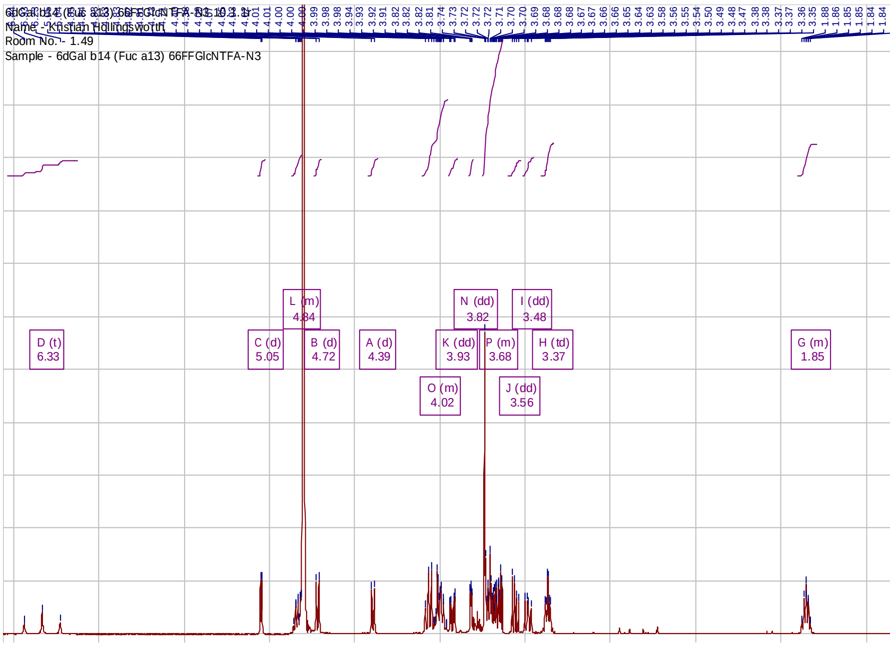

2.19.23.2 Supplementary Figure 262. ¹³C NMR spectrum for compound **LeX23**

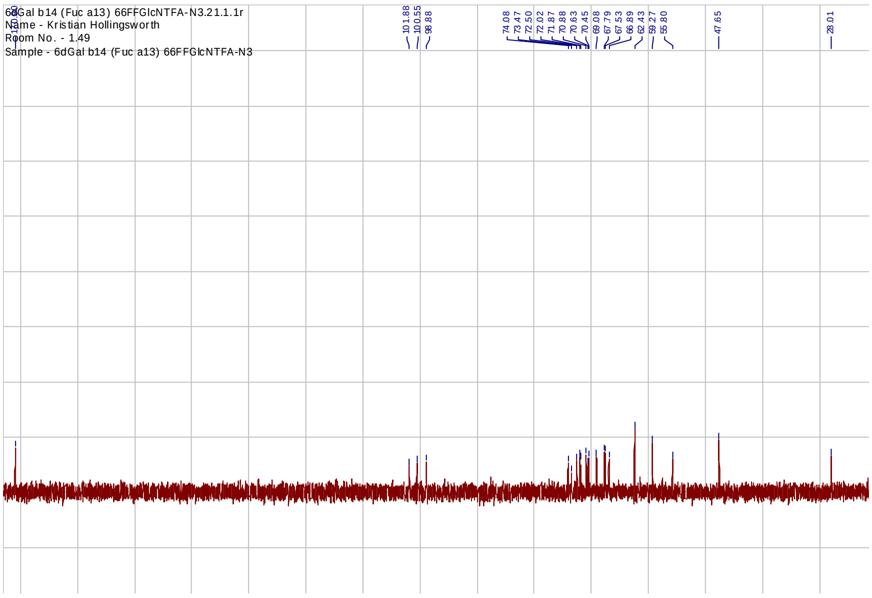

2.19.23.3 Supplementary Figure 263. $^{19}F\{1H\}$ NMR (Insert: ^{19}F NMR expansion) spectrum for compound LeX23

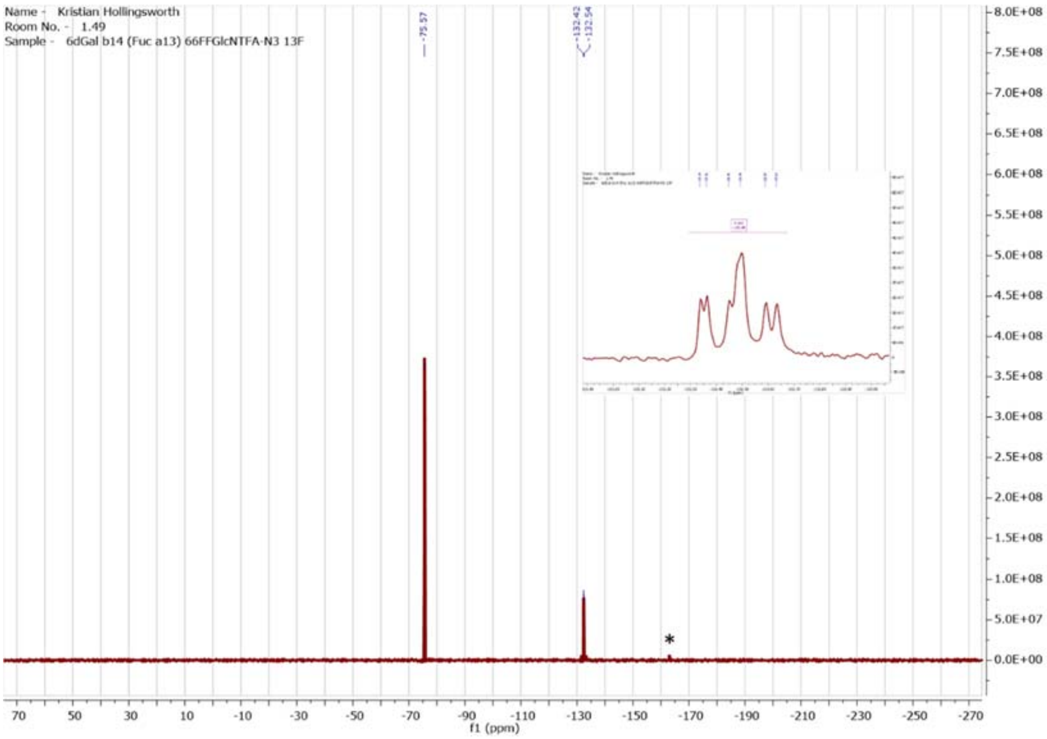

2.19.24 LeX24

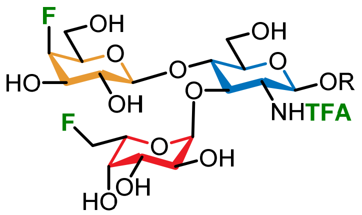

2.19.24.1 Supplementary Figure 264. ^{1}H NMR spectrum for compound **LeX24**

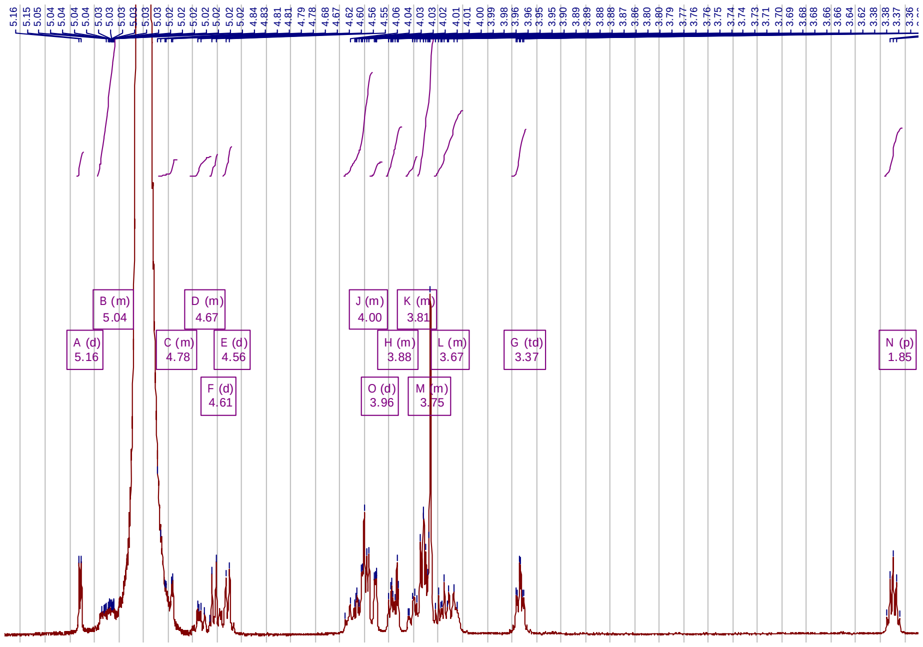

2.19.24.2 Supplementary Figure 265. ^{13}C NMR spectrum for compound **LeX24**

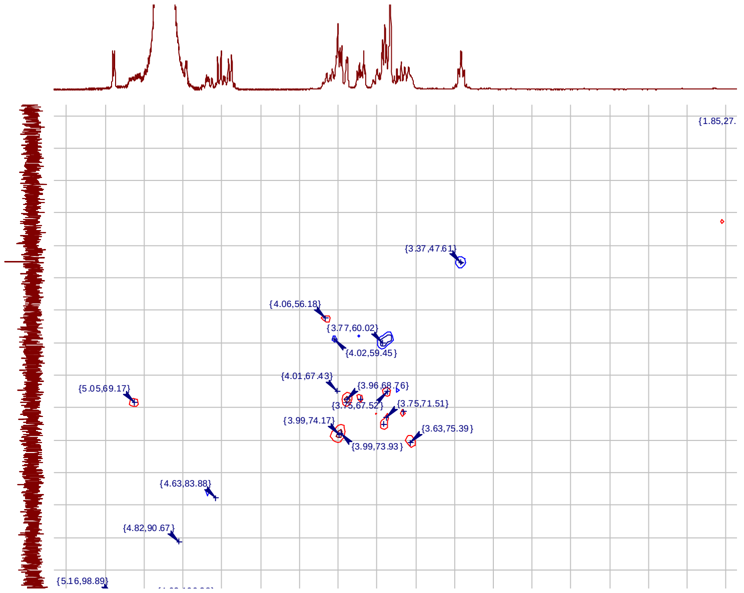

2.19.24.3 Supplementary Figure 266. $^{19}F\{1H\}$ NMR (Insert: ^{19}F NMR expansion) spectrum for compound **LeX24**

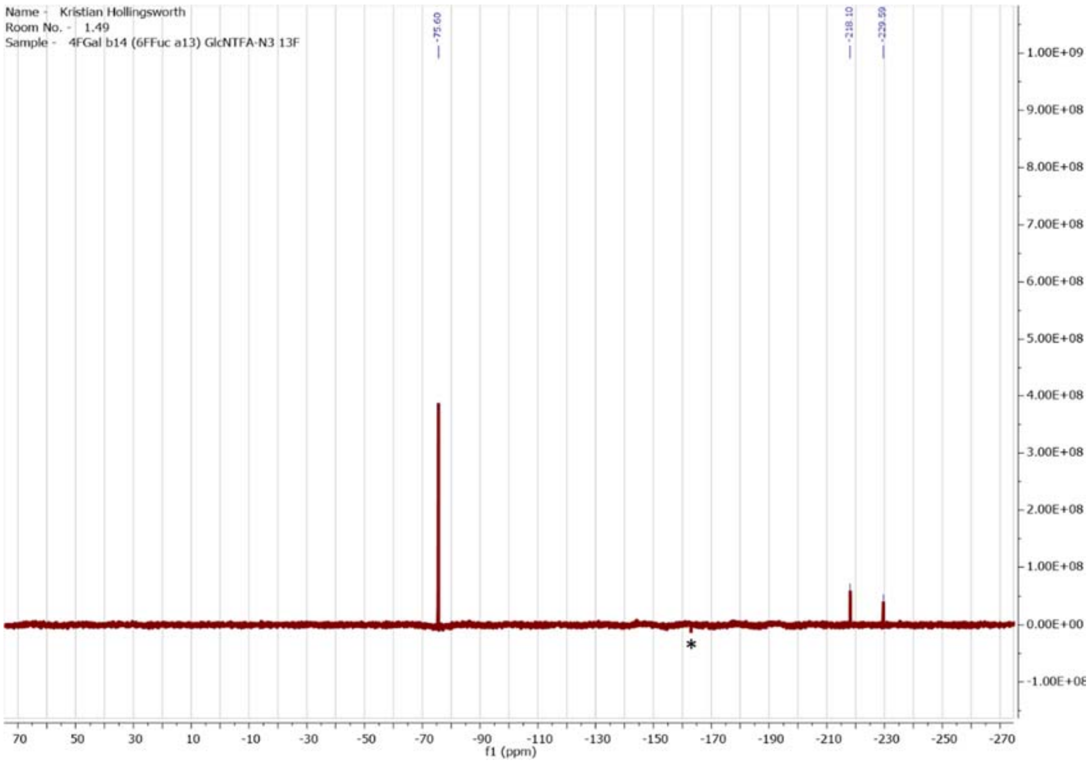

2.20 Supplementary Figures 267-268. NGL related

2.20.1 Supplementary Figure 267. Negative-ion MALDI-MS spectrum of the DBCO-DH lipid reagent.

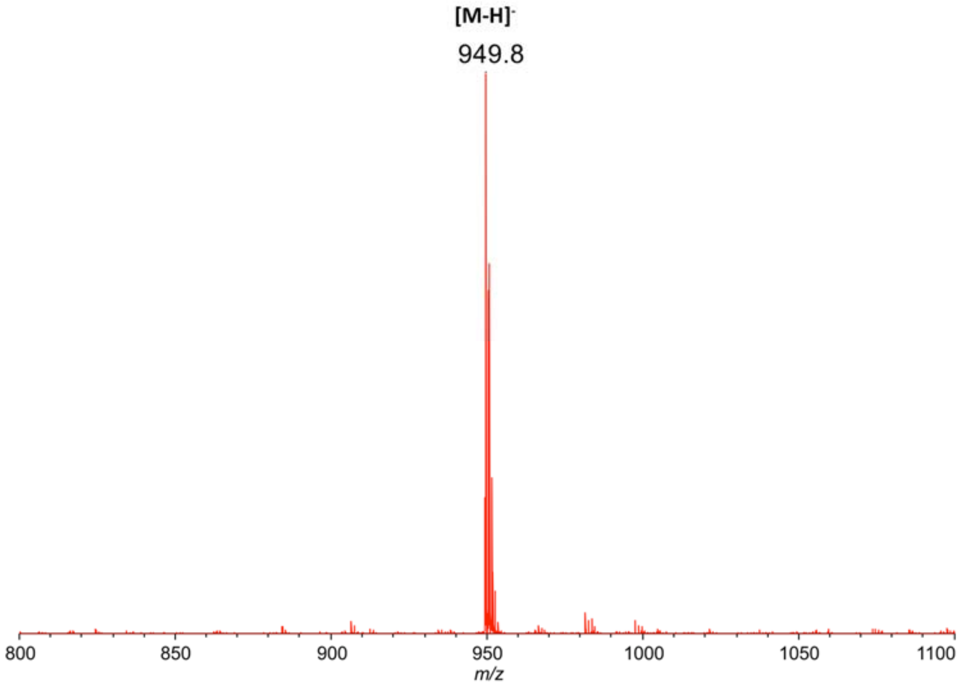

2.20.2 Supplementary Figure 268. Negative-ion MALDI-MS spectra of the 24 Lewis^X related NGLs. R_1= OCH2-CH2-CH2-N_3-DBCO-DH.

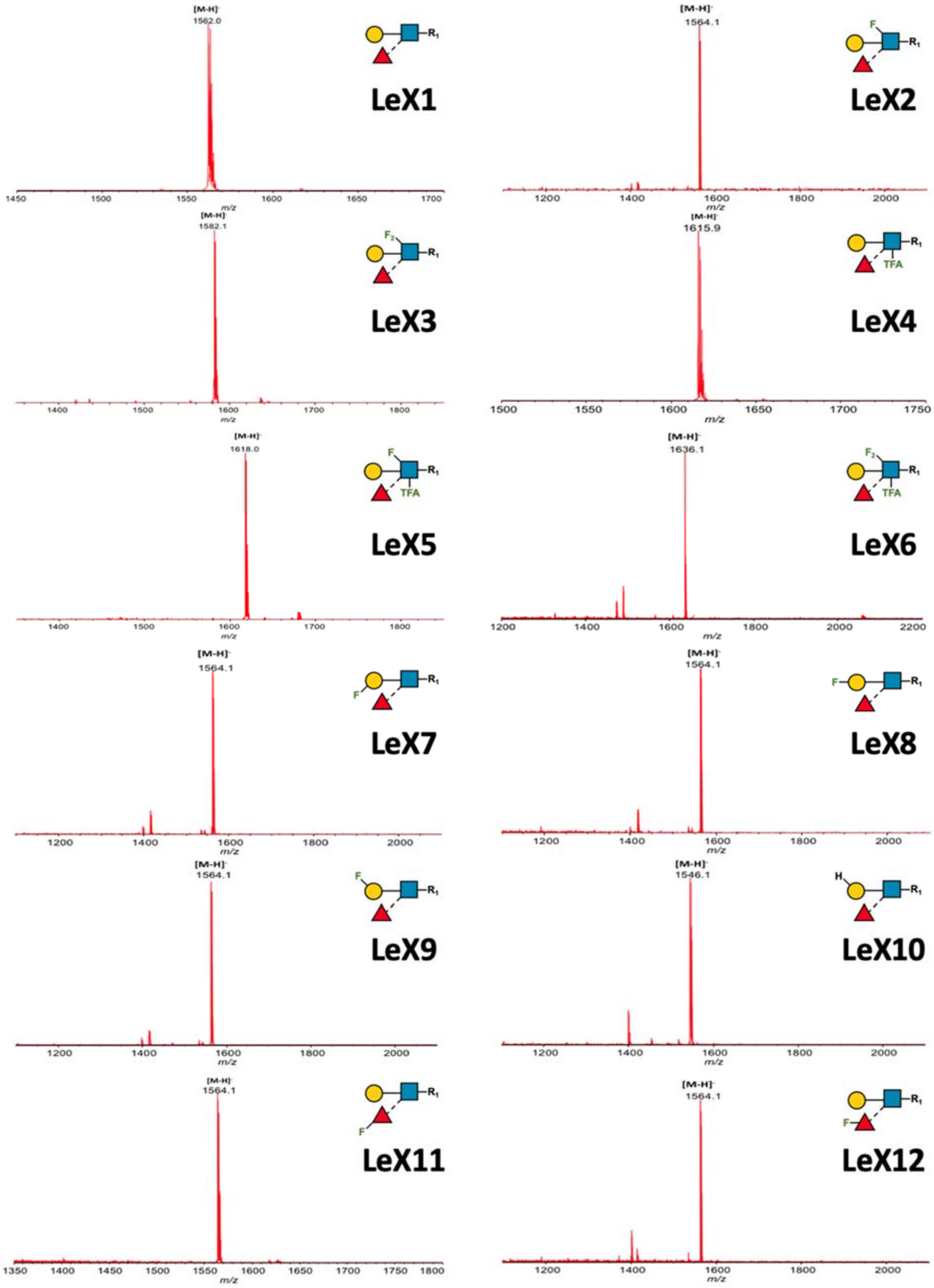

Continue overleaf

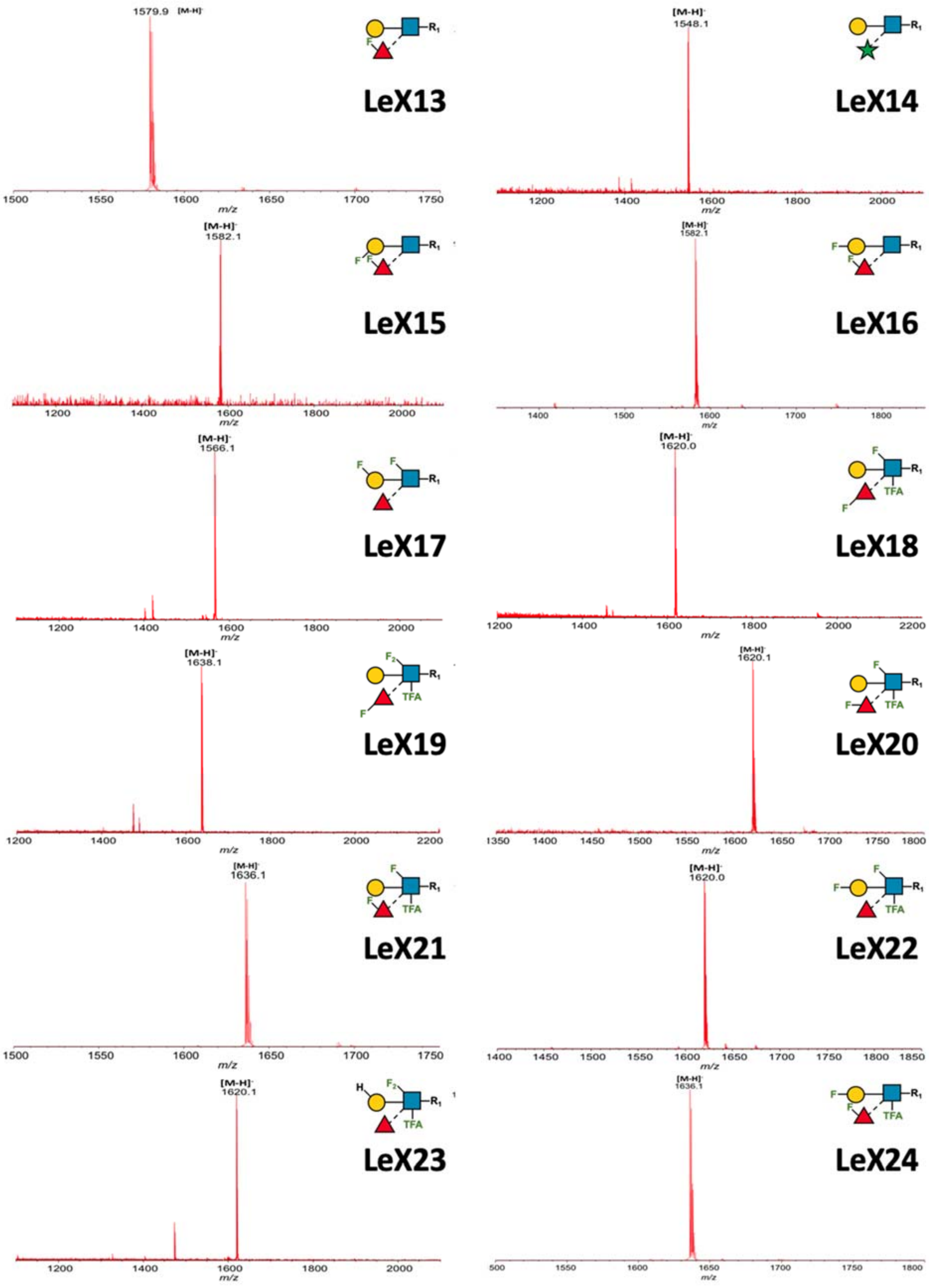
LeX13
1579.9 [M-H]-
LeX14
[M-H]- 1548.1
LeX15
[M-H]- 1582.1
LeX16
[M-H]- 1582.1
LeX17
[M-H]- 1566.1
LeX18
[M-H]- 1620.0
LeX19
[M-H]- 1638.1
LeX20
[M-H]- 1620.1
LeX21
[M-H]- 1636.1
LeX22
[M-H]- 1620.0
LeX23
[M-H]- 1620.1
LeX24
[M-H]- 1636.1
m/z
R1
TFA

3 Supplementary Tables

3.1 Supplementary Table 1. ESMS-derived conversion efficiencies of GlcNAc derivatives bearing ITags to LacNAc analogues, and from LacNAc analogues to Lewis^x analogues containing fucose, 3F-Fuc, 4F-Fuc, 6F-Fuc, and Ara

All tables display conversion as a percentage. All values are taken as a total conversion from GlcNAc-ITag analogue to LacNAc-ITag analogue, or *conversion from LacNAc-ITag analogue to LewisX-ITag analogue.*

GlcNAc-ITag to LacNAc-ITag						
**Disacch.**	GlcNAc	6F-GlcNAc	6,6-diF-GlcNAc	GlcNTFA	6F-GlcNTFA	6,6-diF-GlcNTFA
Gal	100	97	96	99	95	88
3F-Gal	38	18	13	23	10	7
4F-Gal	100	95	89	94	100	68
6F-Gal	71	35	22	54	18	6
6d-Gal	100	92	93	93	74	60

LacNAc-ITag to LewisX-ITag						
**Fuc**	GlcNAc	6F-GlcNAc	6,6-diF-GlcNAc	GlcNTFA	6F-GlcNTFA	6,6-diF-GlcNTFA
Gal	100	92	100	100	97	93
3F-Gal	99	100	100	100	86	66
4F-Gal	100	99	100	100	97	95
6F-Gal	100	100	100	100	93	76
6d-Gal	98	92	94	100	98	85

LacNAc-ITag to LewisX-ITag						
**3F-Fuc**	GlcNAc	6F-GlcNAc	6,6-diF-GlcNAc	GlcNTFA	6F-GlcNTFA	6,6-diF-GlcNTFA
Gal	100	100	100	100	98	100
3F-Gal	100	88	82	93	82	74
4F-Gal	100	100	100	100	98	97
6F-Gal	84	94	100	97	90	77
6d-Gal	31	96	96	97	100	93

LacNAc-ITag to LewisX-ITag						
**4F-Fuc**	GlcNAc	6F-GlcNAc	6,6-diF-GlcNAc	GlcNTFA	6F-GlcNTFA	6,6-diF-GlcNTFA
Gal	100	100	100	100	100	100
3F-Gal	100	100	100	100	91	100
4F-Gal	100	100	100	98	98	100
6F-Gal	57	100	100	96	72	100
6d-Gal	16	80	60	90	95	91

LacNAc-ITag to LewisX-ITag

**6F-Fuc**	GlcNAc	6F-GlcNAc	6,6-diF-GlcNAc	GlcNTFA	6F-GlcNTFA	6,6-diF-GlcNTFA
Gal	100	100	100	100	100	100
3F-Gal	89	63	73	82	83	74
4F-Gal	100	88	100	100	95	100
6F-Gal	94	58	74	85	70	100
6d-Gal	99	100	100	100	100	86

LacNAc-ITag to LewisX-ITag						
**Ara**	GlcNAc	6F-GlcNAc	6,6-diF-GlcNAc	GlcNTFA	6F-GlcNTFA	6,6-diF-GlcNTFA
Gal	100	100	79	81	92	68
3F-Gal	100	100	82	84	74	100
4F-Gal	100	100	79	80	92	70
6F-Gal	100	100	80	86	81	73
6d-Gal	97	100	80	84	94	72

3.2 Supplementary Table 2. List of the NGL probes included in the microarray study with their glycan sequences.

Name	Position in the array	Sequences	
		Textual[a]	Symbolic[b]
LeX1	1	Galβ1-4(Fucα1-3)GlcNAcβ1-R1	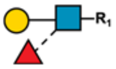
LeX2	2	Galβ1-4(Fucα1-3)6F-GlcNAcβ1-R1	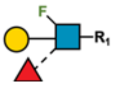
LeX3	3	Galβ1-4(Fucα1-3)6,6-diFGlcNAcβ1-R1	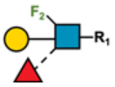
LeX4	4	Galβ1-4(Fucα1-3)GlcNTFAβ1-R1	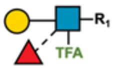
LeX5	5	Galβ1-4(Fucα1-3)6F-GlcNTFAβ1-R1	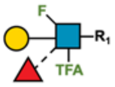
LeX6	6	Galβ1-4(Fucα1-3)6,6-diFGlcNTFAβ1-R1	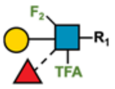
LeX7	7	3F-Galβ1-4(Fucα1-3)GlcNAcβ1-R1	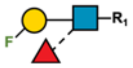
LeX8	8	4F-Galβ1-4(Fucα1-3)GlcNAcβ1-R1	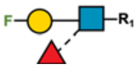

LeX9	9	6F-Galβ1-4(Fucα1-3)GlcNAcβ1-R1	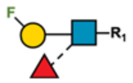
LeX10	10	6d-Galβ1-4(Fucα1-3)GlcNAcβ1-R1	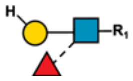
LeX11	11	Galβ1-4(3F-Fucα1-3)GlcNAcβ1-R1	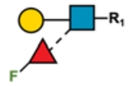
LeX12	12	Galβ1-4(4F-Fucα1-3)GlcNAcβ1-R1	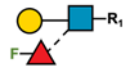
LeX13	13	Galβ1-4(6F-Fucα1-3)GlcNAcβ1-R1	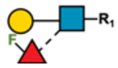
LeX14	14	Galβ14(Araα1-3)GlcNAcβ1-R1	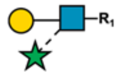
LeX15	15	3F-Galβ1-4(6F-Fucα1-3)GlcNAcβ1-R1	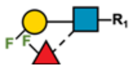
LeX16	16	4F-Galβ1-4(6F-Fucα1-3)GlcNAcβ1-R1	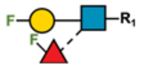
LeX17	17	6F-Galβ1-4(Fucα1-3)6F-GlcNAcβ1-R1	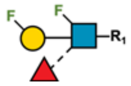
LeX18	18	Galβ1-4(3F-Fucα1-3)6F-GlcNTFAβ1-R1	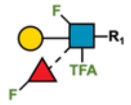
LeX19	19	Galβ1-4(3F-Fucα1-3)6,6-diFGlcNTFAβ1-R1	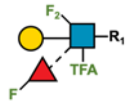
LeX20	20	Galβ1-4(4F-Fucα1-3)6F-GlcNTFAβ1-R1	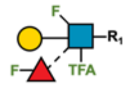
LeX21	21	Galβ1-4(6F-Fucα1-3)6F-GlcNTFAβ1-R1	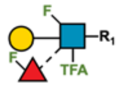
LeX22	22	4F-Galβ1-4(Fucα1-3)6F-GlcNTFAβ1-R1	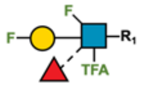
LeX23	23	6d-Galβ1-4(Fucα1-3)6,6-diFGlcNTFAβ1-R1	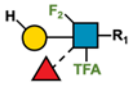
LeX24	24	4F-Galβ1-4(6F-Fucα1-3)GlcNTFAβ1-R1	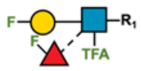
LNnT-DH	25	Galβ-4GlcNAcβ-3Galβ-4Glc-DH	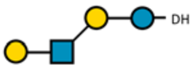

LNFP-III-DH	26	Galβ-4GlcNAcβ-3Galβ-4Glc-DH<br>| Fucα-3	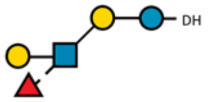
GM1-penta-DH	27	Galβ-3GalNAcβ-4Galβ-4Glc-DH<br>| NeuAcα-3	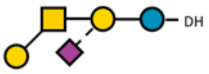
Man9GN2-AO	28	Manα-2Manα-6<br>| Manα-2Manα-3Manα-6<br>| Manβ-4GlcNAcβ-4GlcNAc-AO<br>| Manα-2Manα-2Manα-3	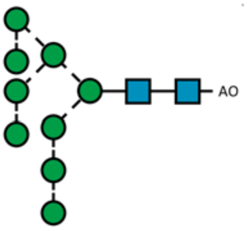

[a] Sequence of NGL probes in textual format. Definition of the lipid tags: R_1= OCH_2-CH_2-CH_2-N_3-DBCO-DH; DH, amino lipid 1,2-dihexadecyl-sn-glycero-3-phosphoethanolamine (DHPE); AO, aminooxy (AO) functionalised DHPE. Please see Table S3 for details.

[b] Glycan sequences presented using the Symbol Nomenclature for Glycans (SNFG) style. (https://www.ncbi.nlm.nih.gov/glycans/snfg.html)

3.3 Supplementary Table 3. Supplemental glycan microarray document based on MIRAGE Guidelines **(doi:10.3762/mirage.3)**[2]

<table>
<tr><th>Classification</th><th>Guidelines</th></tr>
<tr><td colspan="2">1. Sample: Glycan Binding Sample</td></tr>
<tr><td>Description of Sample</td><td>
The list of glycan binding proteins investigated summarised in the table below.
<table>
<tr><th></th><th>Source</th><th>Additional information</th></tr>
<tr><td>human DC SIGN / CD209</td><td>Sino Biological 10200-H01H</td><td rowspan="3">Recombinant protein expressed in HEK293 Cells</td></tr>
<tr><td>human DC SIGNR / CD299</td><td>Sino Biological 10559-H01H</td></tr>
<tr><td>Rhesus Langerin / CD207 Langerin</td><td>Sino Biological 90159-C01H</td></tr>
<tr><td>Anti-L5</td><td>Gift from Andrea Streit, King's College London</td><td>Monoclonal rat IgM[3]</td></tr>
<tr><td>Anti Lewis^X (BG-7)</td><td>Bio Legend 912901</td><td>Monoclonal mouse IgM</td></tr>
<tr><td>Anti Lewis^X (anti-SSEA-1)</td><td>DSHB MC-480 (SSEA-1) Concentrate 0.1 mL</td><td>Monoclonal mouse IgM</td></tr>
<tr><td>Classical CTB</td><td>Sigma-Aldrich Merck c9903</td><td></td></tr>
<tr><td>CTB El Tor biotype (El Tor CTB)</td><td rowspan="2">Prepared by Turnbull Lab</td><td rowspan="2">Expressed and purified from Vibrio sp60, for details please see the Supporting Information of Mandal et al.[4]</td></tr>
<tr><td>E. coli heat-labile toxin B-subunit (LTBh)</td></tr>
</table>
</td></tr>
<tr><td>Sample modifications</td><td>Not relevant.</td></tr>
<tr><td>Assay protocol</td><td>
Microarray analyses were performed essentially as described.[5] In brief, the subarrays after wetting with 10 mM Hepes buffer pH 7.4, 150 mM NaCl with 5 mM CaCl2 (HBS-Ca) for 1 min and blocked with 140 µL of the blocking solution, followed by the incubation steps with the proteins and antibodies. The Fc-tagged proteins (human DC-SIGN, human DC-SIGNR and rhesus Langerin) were analysed as protein-antibody complexes, which were prepared by incubation of the Fc-tagged lectins with biotinylated anti-human IgG at a ratio of 1:2 (by weight) for 1h at 4°C. The analyses of the anti-LewisX antibodies (anti-L5, anti-BG-7, and anti-SSEA-1) and the bacterial toxin proteins (Classical CTB, El Tor variant and LTBh) analysed by a stepwise procedure. For all the analyses, AlexaFluor-647-labeled streptavidin (overlaid at 1 µg/mL for 30 min) was used as the final fluorescence detection step.

The detection and other biological reagents used are summarised below.
<table>
<tr><th>Reagents used</th><th>Source and references</th></tr>
<tr><td colspan="2">Detection antibodies</td></tr>
<tr><td>Rabbit anti-Cholera Toxin antibody</td><td>Sigma-Aldrich Merck C3062</td></tr>
<tr><td>Biotinylated goat anti-human IgG</td><td>Vector Lab BA3000</td></tr>
<tr><td>Biotinylated goat anti-mouse IgM</td><td>Vector Lab BA2020</td></tr>
</table>
</td></tr>
</table>

Biotinylated rabbit anti-rat IgM	Rockland Immunochemicals 612-4607
Biotinylated goat anti-rabbit IgG	MERCK- B7389
Other reagents	
Alexa Fluor-647-labeled streptavidin	Thermo Fisher S21374
Bovine Serum Albumin (BSA) solution (30% in saline, protease free, aseptically filled)	Sigma-Aldrich Merck A8577
BSA solution (heat shock fraction, protease free, fatty acid free, essentially globulin free, pH 7, ≥98%)	Sigma-Aldrich Merck A7030
Blocker™ Casein in PBS	Thermo Fisher 37582
Hepes Buffer 1M solution	Fisher scientific BP299-1

Information on blocking conditions, overlay concentrations of the glycan binding proteins, and the detection antibodies used are summarised in the table below.

Blocker and diluent solutions used:

A. 1% (w/v) BSA (A8577), containing 0.02% (w/v) casein blocker 5 mM $CaCl_2$ in HBS
B. 3% (w/v) BSA (A8577), 5 mM $CaCl_2$ in HBS
C. 1% (w/v) BSA (A8577), 5 mM $CaCl_2$ in HBS
D. 1% (w/v) BSA (A7030), containing 0.02% (w/v) casein blocker 5 mM $CaCl_2$ in HBS

Glycan binding sample	Overlay Conc.	1st Detection antibody (Conc)	2nd Detection antibody (Conc)	Blocker and diluent used
Human DC-SIGN	5 µg/mL	Biotin (BI) anti-human IgG	NA	Solution A
Human DC-SIGNR	20 µg/mL			
Rhesus Langerin	20 µg/mL			
Anti Lewis^X (BG-7)	1/50	BI anti-mouse IgM (1/200)	NA	Solution B as blocker and C as diluent
Anti Lewis^X (anti-SSEA1)	1/200			
Anti-L5	1/200	BI anti-Rat IgM (1/200)		
Classical CTB	25 µg/mL	Rabbit anti-CTB (1/1000)	BI anti-rabbit IgG (1/200)	Solution D
El Tor CTB	25 µg/mL			
LTBh	25 µg/mL			

2. Glycan Library

Glycan description	The Focused Lewis^X NGL Array contained 28 NGL probes, the names and structures of which are in Supplementary Table S2. This is a sub-set of fluorinated glycan microarray (in-house designation “BBSRC Fluoro-glycan array Sets 5,6”); the full probe list of which will be published elsewhere.

Glycan modifications	NGLs derived from the 24 Lewis^X trisaccharide analogues in the Focused-Array were prepared by SPAAC coupling using DBCO-DHPE as described in Materials and Methods.<br><br>The four NGL standards were selected from the Glycosciences Laboratory probe collection (https://glycosciences.med.ic.ac.uk/glycanLibraryList.html). NGLs of LNnT, LNFP-III and GM1-penta were prepared from reducing oligosaccharides by reductive amination with the amino lipid, 1,2-dihexadecyl-*sn*-glycero-3-phosphoethanolamine (DHPE);[6] the NGL of Man9GN2 was prepared by oxime ligation with aminooxy functionalized DHPE (AOPE).[7]
**3. Printing Surface; e.g., Microarray Slide**	
Description of surface	Nitrocellulose-coated glass microarray slides.
Manufacturer	16-pad UniSart® 3D Microarray Slide from Sartorius (Goettingen, Germany)
Custom preparation of surface	Not relevant.
Non-covalent Immobilization	NGLs were formulated as liposomes by adding carrier lipids, phosphatidylcholine (or 1,2-dihexanoyl-sn-glycero-3-phosphocholine, designated as DHPC) and cholesterol[5] for robotically arraying and non-covalent immobilization on nitrocellulose-coated glass slides.
**4. Arrayer (Printer)**	
Description of Arrayer	Nano-Plotter 2.1 (GeSim, Radeberg, Germany)
Dispensing mechanism	Non-contact liquid delivery with four dispensing tips.
Glycan deposition	Approximately 0.33 nl was printed per spot.<br><br>NGL probes were printed at 2 and 5 fmol per spot in duplicate.
Printing conditions	The printing solutions were aqueous based. Printing was performed at ambient temperature and relative humidity of 50-54%.<br><br>The printing solutions contained 100 pmol/µl each of cholesterol and DHPC as lipid carriers in addition to the lipid-linked glycan probes in water (HPLC grade). The concentrations of the lipid-linked glycan probes were 5 and 15 pmol/µl for the 2 and 5 fmol per spot levels, respectively.<br><br>All printing solutions contained Cy3 NHS ester (GE Healthcare) at 20 ng/ml (26 fmol/µl) as a marker to monitor the printing process.
**5. Glycan Microarray with "Map"**	
Array layout	Each array slide contained 16 identical subarrays (pads). Each pad was set up for printing 64 probes maximum, each at 2 levels in duplicate (four spots for one probe in a row); up to 256 spots (16x16) in total in each pad.
Glycan identification and quality control	The five NGL probes derived from natural glycans LeX1 and the four NGL standards (Table S2) were well recognized as predicted by the C-type lectins, anti-Lewis^X antibodies and the classic

	CTB, consistent with a good quality array. The unnatural Lewis^X NGLs were well printed as visualised by the post printing Cy3 images of the array.
**6. Detector and Data Processing**	
Scanning hardware	GenePix 4300A (Molecular Devices, Berkshire, UK)
Scanner settings	Scanning resolution: 10 µm / pixel (this resolution is adequate for the sizes of sample spots)<br>Laser channel: Red (scan wavelength 635 nm)<br>PMT voltage: 350<br>Scan power was adjusted for each sample to achieve maximum signal without saturation of any single spot. The laser powers used are indicated in the Supplementary Dataset 1 (Excel file).
Image analysis software	GenePix® Pro 7 (Molecular Devices, Berkshire, UK) was used for quantitation of the signal intensities from the array images.
Data processing	The gpr files were entered into an in-house microarray database using software (designed by Mark Stoll, http://www.beilstein-institut.de/en/publications/proceedings/glyco-2009) for data processing. No particular normalization method, or statistical analysis was used.
**7. Glycan Microarray Data Presentation**	
Data presentation	The microarray binding results are in the **Source Data File**, as well as **Figures 4b and Supplementary figure S7**. Binding results are presented as means of fluorescence intensities of duplicate spots at 5 fmol/spot with error bars representing one-half of the difference between the two values.
**8. Interpretation and Conclusion from Microarray Data**	
Data interpretation	No software or algorithms were used to interpret processed data.
Conclusions	Distinct binding patterns were observed to the unnatural Lewis^X NGLs in the microarray with different glycan binding proteins investigated, indicating a protein specific influence of the fluorine modifications on Lewis^X trisaccharide recognition.

3.4 Supplementary Table 4. Summary of effects on glycan binding by proteins with the 11 individual modifications on the Lewis^X structure.

Modification on different monosaccharide residues is indicated in different colours: blue for GlcNAc, gold for Gal, and red for Fuc.

Effect on binding	hDC-SIGN	Anti-L5	Anti-SSEA-1	Anti-Lewis^X (BG-7)	CTB	LTBh
**Strong enhancement**	6,6-diFGlcNAc<br>GlcNTFA	6dH-Gal<br>3F-Fuc	6F-Gal	6F-Gal	3F-Gal<br>4F-Gal<br>3F-Fuc	3F-Fuc
**Modest enhancement**	3F-Gal	6,6-diFGlcNAc<br>6F-Gal	6dH-Gal	4F-Gal<br>6dH-Gal<br>6F-Fuc	6F-GlcNAc<br>6,6-diFGlcNAc<br>6F-Gal<br>6dH-Gal<br>6F-Fuc	6F-GlcNAc<br>6,6-diFGlcNAc<br>4F-Gal<br>6F-Gal<br>6F-Fuc
**Modest diminution**	6F-GlcNAc<br>4F-Gal<br>6F-Gal<br>6dH-Gal<br>6F-Fuc<br>Ara	6F-GlcNAc<br>GlcNTFA<br>4F-Gal<br>4F-Fuc<br>6F-Fuc<br>Ara	GlcNTFA<br>4F-Gal<br>3F-Fuc<br>6F-Fuc	GlcNTFA<br>3F-Fuc<br>4F-Fuc	GlcNTFA	3F-Gal<br>6dH-Gal
**Abolished binding**	3F-Fuc<br>4F-Fuc	3F-Gal	6F-GlcNAc<br>6,6-diFGlcNAc<br>3F-Gal<br>4F-Fuc<br>Ara	6F-GlcNAc<br>6,6-diFGlcNAc<br>3F-Gal<br>Ara	4F-Fuc<br>Ara	GlcNTFA<br>4F-Fuc<br>Ara

3.5 Supplementary Table 5 Summary of results of negative-ion MALDI-MS analysis of the 24 LewisX NGLs.

Glycan	Calculated NGL mass (monoiso)	Detected $[M-H]^-$	Glycan	Calculated NGL mass (monoiso)	Detected $[M-H]^-$
LeX1	1562.9	**1562.0**	LeX13	1580.8	**1579.9**
LeX2	1564.9	**1564.1**	LeX14	1548.9	**1548.1**
LeX3	1582.9	**1582.1**	LeX15	1582.9	**1582.1**
LeX4	1616.8	**1615.9**	LeX16	1582.9	**1582.1**
LeX5	1618.8	**1618.0**	LeX17	1566.9	**1566.1**
LeX6	1636.9	**1636.1**	LeX18	1620.9	**1620.0**
LeX7	1564.9	**1564.1**	LeX19	1638.9	**1638.1**
LeX8	1564.9	**1564.1**	LeX20	1620.8	**1620.1**
LeX9	1564.9	**1564.1**	LeX21	1636.8	**1636.1**
LeX10	1546.9	**1546.1**	LeX22	1620.8	**1620.0**
LeX11	1564.9	**1564.1**	LeX23	1620.9	**1620.1**
LeX12	1564.9	**1564.1**	LeX24	1636.8	**1636.1**

3.6 Supplementary Table 6: Characterisation of non-functionalised and functionalised AuNPs used in this study.

Code	Glycan	UV_{max}(a) (nm)	D_h(c) (nm)	$D_{h\ (DLS)}$(d) (nm)
$AuNP_{55}$	-	533	55	58.6 ± 3.2
**Le^{x}1**-$PHEA_{25}$@$AuNP_{55}$	Gal β1,4 (Fuc α1,3) GlcNAc	538	64	70.6 ± 2.5
**Le^{x}4**-$PHEA_{25}$@$AuNP_{55}$	Gal β1,4 (Fuc α1,3) GlcNTFA	537	62	67.7 ± 2.4
**Le^{x}5**-$PHEA_{25}$@$AuNP_{55}$	Gal β1,4 (Fuc α1,3) 6F-GlcNTFA	537	62	67.0 ± 2.5
**Le^{x}8**-$PHEA_{25}$@$AuNP_{55}$	4F-Gal β1,4 (Fuc α1,3) GlcNAc	537	62	71.9 ± 2.1
**Le^{x}11**-$PHEA_{25}$@$AuNP_{55}$	Gal β1,4 (3F-Fuc α1,3) GlcNAc	537	62	64.9 ± 2.6
**Le^{x}13**-$PHEA_{25}$@$AuNP_{55}$	Gal β1,4 (6F-Fuc α1,3) GlcNAc	538	64	74.9 ± 1.8
**Le^{x}16**-$PHEA_{25}$@$AuNP_{55}$	4F-Gal β1,4 (6F-Fuc α1,3) GlcNAc	538	64	69.4 ± 2.5
**Le^{x}21**-$PHEA_{25}$@$AuNP_{55}$	Gal β1,4 (6F-Fuc α1,3) 6F-GlcNTFA	537	62	79.5 ± 2.5
**Le^{x}22**-$PHEA_{25}$@$AuNP_{55}$	4F-Gal β1,4 (Fuc α1,3) 6F-GlcNTFA	537	62	68.0 ± 2.8
**Le^{x}24**-$PHEA_{25}$@$AuNP_{55}$	Gal β1,4 (6F-Fuc α1,3) GlcNTFA	536	60	65.6 ± 2.0

(a) SPR absorption maximum; (b) Absorbance ratio of SPR to 450 nm; (c) Estimated from UV-Vis[3]; (d) From dynamic light scattering.

4 Supplementary Notes

4.1 Structures of bacterial toxin-Lewis glycan complexes (Supplementary Figures 269–270)

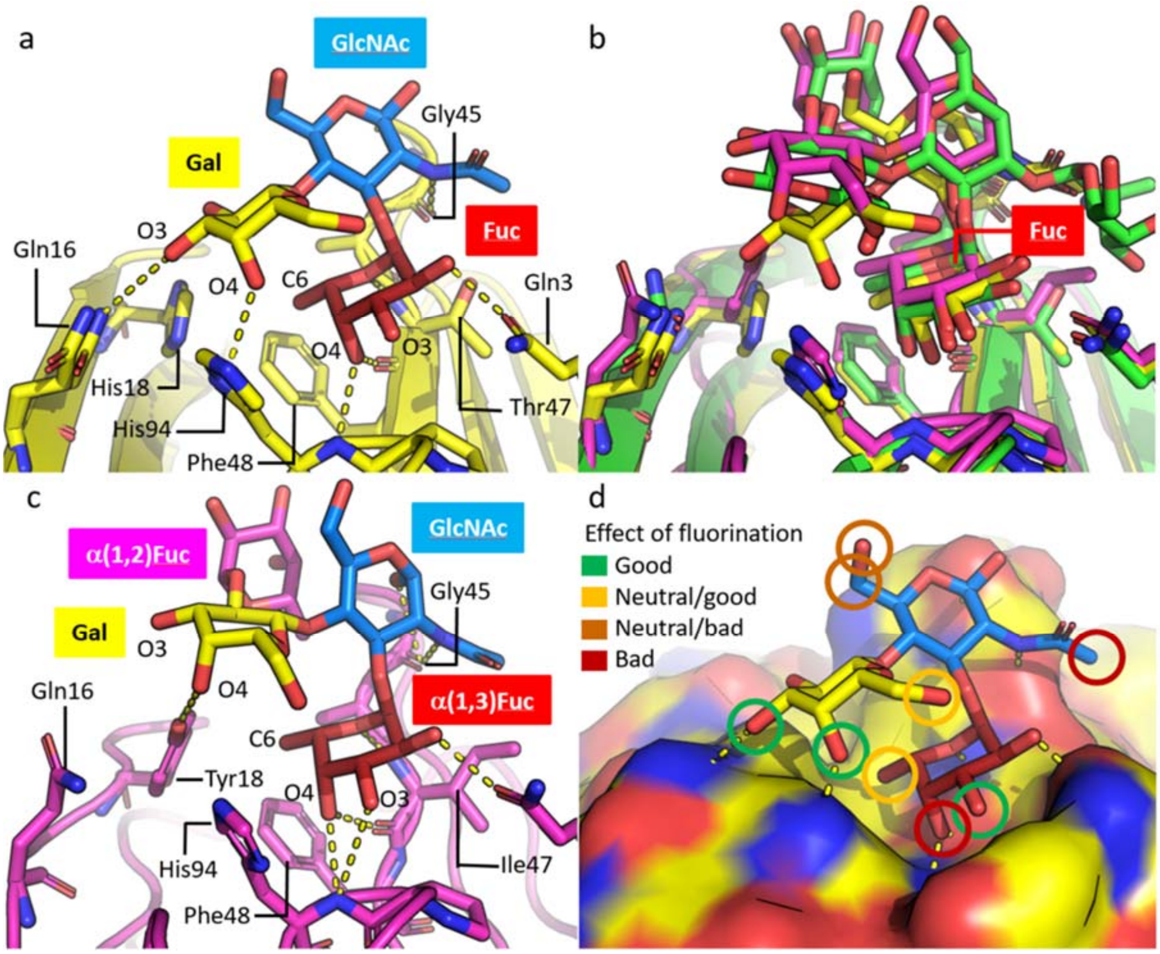

Supplementary figure 269. Structures of bacterial toxin-Lewis glycan complexes. a) Lewis^x binding to Classical CTB based on protein data bank file 6hjd; d) Summary of whether fluorination at different sites on Lewis^x is good or bad for binding to Classical CTB.

A crystal structure for Lewis^x bound to Classical CTB has been reported by Krengel and co-workers (Supplementary Figure 269a).[8] Structures for El Tor CTB and LTBh complexed to other members of the Lewis glycan family are also available and show only minor changes to the location of fucose in the binding pocket (Supplementary Figure 269b).[9] While Lewis^y can bind to El Tor CTB (Supplementary Figure 269c), the His18Tyr mutation (compared to Classical CTB) moves the galactose residue away from the normal closed conformation of Lewis^x.[10] The lower affinity of El Tor CTB for Lewis^x presumably originates from losing the additional stabilising interactions between the α-1,2-fucosyl residue of Lewis^y and the protein. The Lewis^x fucose methyl group sits in a hydrophobic pocket lined by Phe48 and Tyr/His18 (Supplementary Figure 269a). Removal of the methyl group or replacement with a hydroxymethyl group is reported to causes a loss of affinity.[11] In contrast, our results show that a fluoromethyl group at this position (Supplementary Figure 269d, Supplementary Figure 270) is either neutral in its effect or gives a small binding increase in some cases, possibly through an enhanced CH-π interaction with Phe48, or potential stabilisation of the closed conformation of Lewis^x based on the increased chemical shift for Gal-H2 as noted in the main manuscript. Fuc-O4 is placed deep in the binding pocket making potential H-bonds to both the backbone NH of His94 and the carbonyl of Thr47.

Replacing this hydroxy group with hydrogen,[11] or fluorine not surprisingly causes a reduction in affinity. More notable is the enhancing effect of 3F-Fuc on the interactions with all three bacterial toxins. While there are no direct interactions between Fuc-O3 and Classical CTB (Supplementary Figure 269a), deoxygenation at this position is known to be detrimental to binding,[11] and H-bonds are possible from Fuc-O3 to the backbone amide NH of His94 for El Tor CTB (Supplementary Figure 269c) and LTBh. However, such an interaction would presumably be mutually exclusive with the H-bond from Fuc-O4 to His94 or Thr47, so it is possible that 3F-Fuc may be exhibiting enhanced H-bonding through its O4 when position 3 is fluorinated. 4F-Gal provided enhanced affinity for both the El Tor and Classical CTB interactions. In the former, Gal-O4 is within hydrogen bonding distance to Tyr18-OH, and in the latter case to His18-N^{τ}. The reduction in binding associated with trifluorination of the GlcNAc acetamide likely arises from a steric clash with the hydroxy group of Thr47 and the carbonyl of Gly45, but it is more difficult to identify a reason for the smaller negative influence of fluorination at GlcNAc-C6. A summary of Lewis^{x} positions where fluorination enhances or reduces binding to Classical CTB is shown in Supplementary Figure 269d. While it is difficult to draw definitive conclusions in the absence of structural data for each fluorinated glycan, we conclude that the observed modulation of their binding affinities is consistent with the known structures of CTB/LTBh–Lewis glycan complexes.

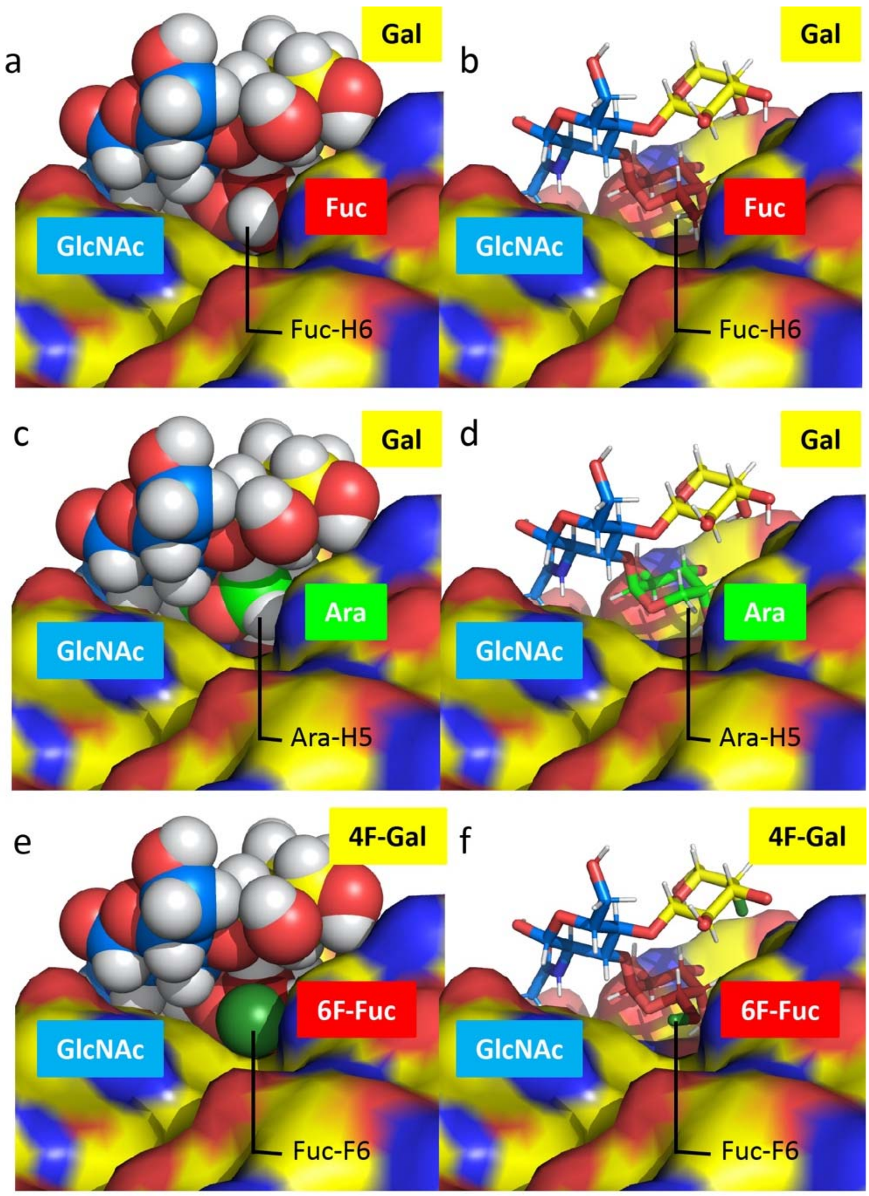

Supplementary figure 270. a) Space-filling and b) stick models of the crystal structure of Lewis^x (LeX1) binding to Classical CTB based on Protein Data Bank file 6hjd.pdb showing the location of the fucosyl methyl group, and models of Lewis^x: c) and d) LeX14; e) and f) Lex16.

4.2 Proof-of-concept: AuNP-based detection of CTB using **LeX4** and **LeX16** (Figures 271–272)

As an initial proof-of-concept for using this a diagnostic a lateral flow 'dipstick' was produced for the detection of CTB (cholera toxin B) using GM1 as the control line, with detection based on visualisation of the AuNP.

In such a test 1 uL of 10 mg.mL^{-1} GM1 is spotted directly onto the nitrocellulose as the test line. A solution containing CTB is flowed over the control line by dipping the stick into the AuNP solution. Through the action of capillary forces the solution moves in upwards direction, aided by the wick at the top-end of the stick. Then a solution containing functionalised AuNPs is applied (Supplementary Figure 269).

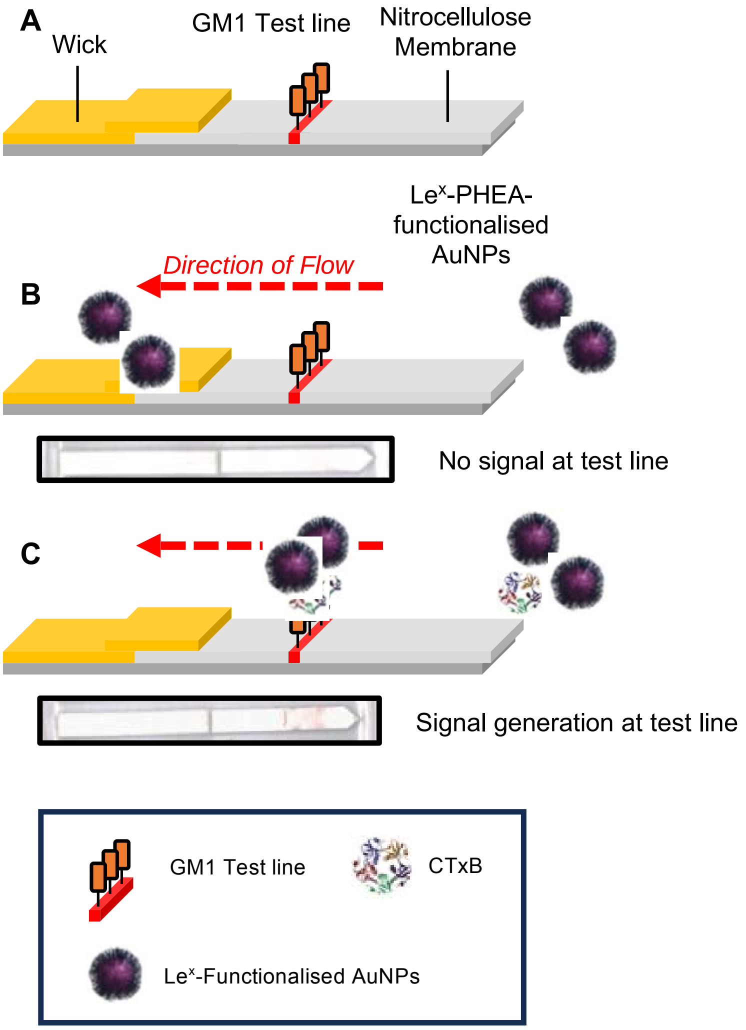

Supplementary figure 271. Schematic of dipstick lateral flow assay. A) Design of dipstick. B) Lateral flow with GM1 test line and no analyte; particles do not engage test line. C) Lateral flow with GM1 test line and CTxB (analyte) resulting in capture and signal generation.

LeX4-functionalised AuNPs and LeX16-functionalised AuNPs were chosen as a low-binding and high-binding example signal generators respectively, guided by the aggregation assays (Supplementary Figure 272). The assay works as follows: GM1 will bind to the CTB. The LeX4-functionalised NP, which do not bind to CTB, will not be captured and hence no signal is generated. In contrast, the LeX16 functionalised NP will bind to the CTB, and this generates a signal at the GM1 spotting line. This shows that the solution assay results translate well to a lateral flow assay with LeX4-functionalised AuNPs giving little to no binding in presence of CTB and LeX16-functionalised showing at least a 10-fold increase in response to CTB. In the presence of no analyte (CTB) there is no response, showing that the AuNPs do not interact with GM1 alone. AuNPs flowed up a black strip (no GM1) show no binding (and flow to the end of the strip).

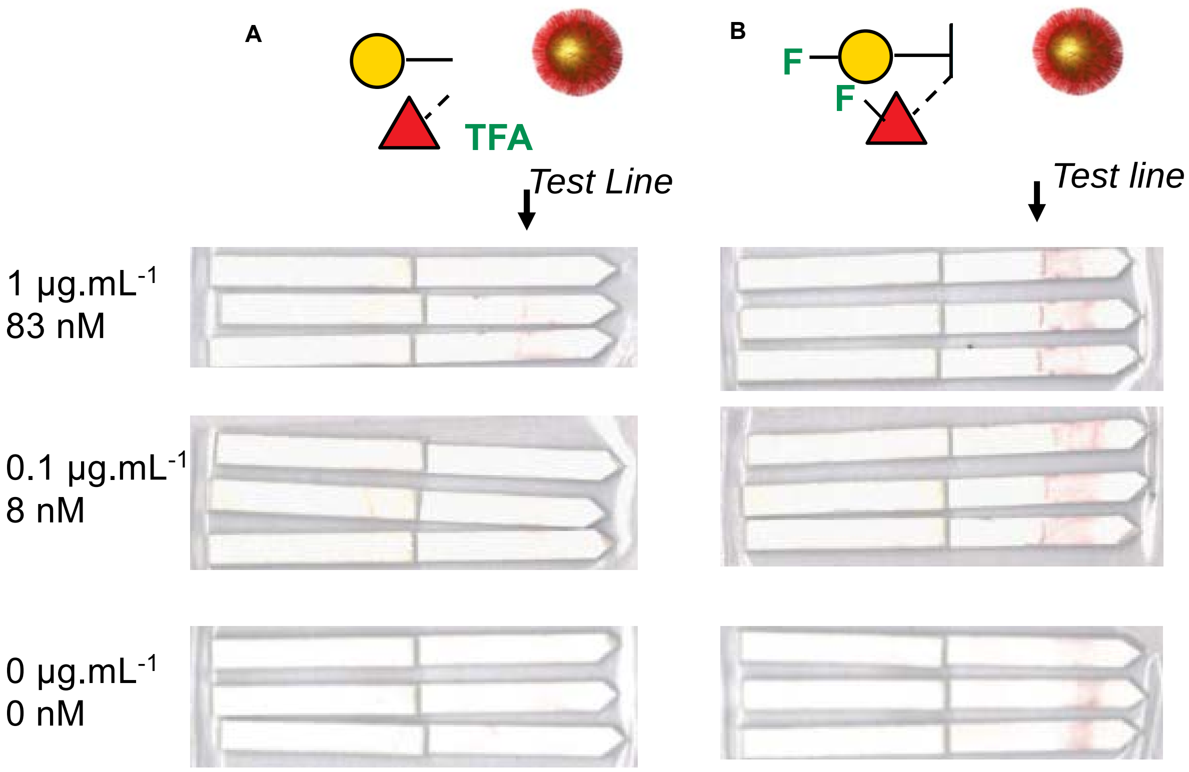

Supplementary figure 272. Scanned images of dipstick lateral flow assays. The wick is on the left hand side (the central line indicates the front of the wick. Flow from right to left. A) Dipsticks run with the low-binding Le^x4-functionalised AuNPs as the signal generator B) Dipsticks run with high-binding Le^x16-functionalised AuNPs as the signal generator. Only the high-binding Le^x16-functionalised AuNPs are captured by CTB bound to GM1.

5 Supplementary References

(1) Zierke, M.; Smieško, M.; Rabbani, S.; Aeschbacher, T.; Cutting, B.; Allain, F. H. T.; Schubert, M.; Ernst, B. Stabilization of Branched Oligosaccharides: Lewisx Benefits from a Nonconventional C–H···O Hydrogen Bond. *J. Am. Chem. Soc.* **2013**, *135* (36), 13464-13472. DOI: 10.1021/ja4054702. Imberty, A.; Pérez, S. Structure, Conformation, and Dynamics of Bioactive Oligosaccharides: Theoretical Approaches and Experimental Validations. *Chem. Rev.* **2000**, *100* (12), 4567-4588. DOI: 10.1021/cr990343j.

(2) Liu, Y.; McBride, R.; Stoll, M.; Palma, A. S.; Silva, L.; Agravat, S.; Aoki-Kinoshita, K. F.; Campbell, M. P.; Costello, C. E.; Dell, A.; et al. The minimum information required for a glycomics experiment (MIRAGE) project: improving the standards for reporting glycan microarray-based data. *Glycobiology* **2017**, *27* (4), 280-284. DOI: 10.1093/glycob/cww118 From NLM.

(3) Streit, A.; Yuen, C.-T.; Loveless, R. W.; Lawson, A. M.; Finne, J.; Schmitz, B.; Feizi, T.; Stern, C. D. The Lex Carbohydrate Sequence Is Recognized by Antibody to L5, a Functional Antigen in Early Neural Development. *J. Neurochem.* **1996**, *66* (2), 834-844, https://doi.org/10.1046/j.1471-4159.1996.66020834.x. DOI: https://doi.org/10.1046/j.1471-4159.1996.66020834.x (acccessed 2023/02/28).

(4) Mandal, P. K.; Branson, T. R.; Hayes, E. D.; Ross, J. F.; Gavín, J. A.; Daranas, A. H.; Turnbull, W. B. Towards a structural basis for the relationship between blood group and the severity of El Tor cholera. *Angew. Chem. Int. Ed. Engl.* **2012**, *51* (21), 5143-5146. DOI: 10.1002/anie.201109068 From NLM.
(5) Liu, Y.; Childs, R. A.; Palma, A. S.; Campanero-Rhodes, M. A.; Stoll, M. S.; Chai, W.; Feizi, T. Neoglycolipid-based oligosaccharide microarray system: preparation of NGLs and their noncovalent immobilization on nitrocellulose-coated glass slides for microarray analyses. *Methods Mol Biol* **2012**, *808*, 117-136. DOI: 10.1007/978-1-61779-373-8_8 From NLM.
(6) Chai, W.; Stoll, M. S.; Galustian, C.; Lawson, A. M.; Feizi, T. Neoglycolipid Technology: Deciphering Information Content of Glycome. In *Methods in Enzymology*, Vol. 362; Academic Press, 2003; pp 160-195.
(7) Liu, Y.; Feizi, T.; Carnpanero-Rhodes, M. A.; Childs, R. A.; Zhang, Y.; Muiioy, B.; Evans, P. G.; Osborn, H. M. I.; Otto, D.; Crocker, P. R.; Chai, W. Neoglycolipid probes prepared via oxime ligation for microarray analysis of oligosaccharide-protein interactions. *Chem. Biol.* **2007**, *14* (7), 847-859. DOI: 10.1016/j.chembiol.2007.06.009.
(8) Heim, J. B.; Hodnik, V.; Heggelund, J. E.; Anderluh, G.; Krengel, U. Crystal structures of cholera toxin in complex with fucosylated receptors point to importance of secondary binding site. *Sci Rep* **2019**, *9* (1), 12243. DOI: 10.1038/s41598-019-48579-2 From NLM.
(9) Heggelund, J. E.; Burschowsky, D.; Bjørnestad, V. A.; Hodnik, V.; Anderluh, G.; Krengel, U. High-Resolution Crystal Structures Elucidate the Molecular Basis of Cholera Blood Group Dependence. *PLOS Pathogens* **2016**, *12* (4), e1005567. DOI: 10.1371/journal.ppat.1005567. Holmner, Å.; Askarieh, G.; Ökvist, M.; Krengel, U. Blood Group Antigen Recognition by Escherichia coli Heat-labile Enterotoxin. *J. Mol. Biol.* **2007**, *371* (3), 754-764. DOI: https://doi.org/10.1016/j.jmb.2007.05.064.
(10) Zierke, M.; Smieško, M.; Rabbani, S.; Aeschbacher, T.; Cutting, B.; Allain, F. H. T.; Schubert, M.; Ernst, B. Stabilization of Branched Oligosaccharides: Lewisx Benefits from a Nonconventional C–H···O Hydrogen Bond. *J. Am. Chem. Soc.* **2013**, *135* (36), 13464-13472. DOI: 10.1021/ja4054702. Imberty, A.; Pérez, S. Structure, Conformation, and Dynamics of Bioactive Oligosaccharides: Theoretical Approaches and Experimental Validations. *Chem. Rev.* **2000**, *100* (12), 4567-4588. DOI: 10.1021/cr990343j.
(11) Wands, A. M.; Cervin, J.; Huang, H.; Zhang, Y.; Youn, G.; Brautigam, C. A.; Matson Dzebo, M.; Björklund, P.; Wallenius, V.; Bright, D. K.; et al. Fucosylated Molecules Competitively Interfere with Cholera Toxin Binding to Host Cells. *ACS Infect Dis* **2018**, *4* (5), 758-770. DOI: 10.1021/acsinfecdis.7b00085 From NLM.
