# Supplementary material for: Synthesis and screening of a library of Lewisx deoxyfluoro-analogues reveals differential recognition by glycan-binding partners
Source: Nat Commun. 2024 Sep 13;15:7925. doi: 10.1038/s41467-024-51081-7 (PMC11399408; doi:10.1038/s41467-024-51081-7)
Supplement: Supplementary file 3 — Reporting Summary [file 41467_2024_51081_MOESM3_ESM.pdf]

nature portfolio

Corresponding author(s): Ten Feizi, Yan Liu, Matthew Gibson, W. Bruce Turnbull, Bruno Linclau

Last updated by author(s): Jul 16, 2024

Reporting Summary

Nature Portfolio wishes to improve the reproducibility of the work that we publish. This form provides structure for consistency and transparency in reporting. For further information on Nature Portfolio policies, see our Editorial Policies and the Editorial Policy Checklist.

Statistics

For all statistical analyses, confirm that the following items are present in the figure legend, table legend, main text, or Methods section.

n/a	Confirmed	
☐	☒	The exact sample size (*n*) for each experimental group/condition, given as a discrete number and unit of measurement
☐	☒	A statement on whether measurements were taken from distinct samples or whether the same sample was measured repeatedly
☐	☒	The statistical test(s) used AND whether they are one- or two-sided<br>*Only common tests should be described solely by name; describe more complex techniques in the Methods section.*
☒	☐	A description of all covariates tested
☒	☐	A description of any assumptions or corrections, such as tests of normality and adjustment for multiple comparisons
☐	☒	A full description of the statistical parameters including central tendency (e.g. means) or other basic estimates (e.g. regression coefficient) AND variation (e.g. standard deviation) or associated estimates of uncertainty (e.g. confidence intervals)
☐	☒	For null hypothesis testing, the test statistic (e.g. *F*, *t*, *r*) with confidence intervals, effect sizes, degrees of freedom and *P* value noted<br>*Give P values as exact values whenever suitable.*
☒	☐	For Bayesian analysis, information on the choice of priors and Markov chain Monte Carlo settings
☒	☐	For hierarchical and complex designs, identification of the appropriate level for tests and full reporting of outcomes
☐	☒	Estimates of effect sizes (e.g. Cohen's *d*, Pearson's *r*), indicating how they were calculated

Our web collection on statistics for biologists contains articles on many of the points above.

Software and code

Policy information about availability of computer code

Data collection	For the synthetic glycans, NMR spectra were acquired using Bruker TopSpin version 3.6.3 for 400 MHz and TopSpin version 4.1.3 for 500 MHz experiments. Mass spectrometry experiments were acquired using Bruker Daltonics otofControl Version 3.2. For glycan microarrays, the fluorescence microarray images were recorded using GenePix 4300A scanner (Molecular Devices). Transmission Electron Microscopy data was acquired using Thermo Scientific Velox software supplied with the Thermo Talos F200X microscope. Data for protein-mediated aggregation study was collected on a Biotek Synergy HT using Gen 5 software version 1.11. Isothermal titration Calorimetry data was acquired using MicroCal iTC200 software version 1.25.5
Data analysis	Mass spectra were analysed in Bruker Compass DataAnalysis 4.3 software (Bruker Daltonik). NMR spectra were analysed in Mnova 14.3.3 (Mestrelab). Microarray data (image analysis) was performed using GenePix® Pro 7 (Molecular Devices). Binding data and Protein-mediated aggregation data was analysed, fitted and plotted using OriginPro 2022b Version 9.9.5.171 academic. Nanoparticle TEM data was analysed using Image J2 version 2.14.0/1.54f. Isothermal titration Calorimetry Data was processed using the MicroCal PEAQ-ITC analysis software version 1.0.0.1259

For manuscripts utilizing custom algorithms or software that are central to the research but not yet described in published literature, software must be made available to editors and reviewers. We strongly encourage code deposition in a community repository (e.g. GitHub). See the Nature Portfolio guidelines for submitting code & software for further information.

Data

Policy information about availability of data

All manuscripts must include a data availability statement. This statement should provide the following information, where applicable:

- Accession codes, unique identifiers, or web links for publicly available datasets
- A description of any restrictions on data availability
- For clinical datasets or third party data, please ensure that the statement adheres to our policy

All data associated with this report as contained within the manuscript, the supplemental information or source data files.
Source data files of NMR spectroscopy and mass spectrometry data associated with glycan synthesis, and isothermal titration calorimetry data will be made available through the Leeds Research Data Repository (DOI: https://doi.org/10.5518/1412).
The glycan array datasets will be deposited and shared via the GlyGen Glycan Array Repository currently under its final testing phase as part of the NIH-funded GlyGen initiative (https://www.glygen.org/). Currently there is no currently public glycan array data repository in use. Once published we will share our data via our Facility web portal. In the meantime, the raw fluorescence intensities of the quantified microarray data are provided as Source data _ Supplementary DataSet 1 (excel file). All the glycan array related metadata are in the the Supplementary Information (Supplementary Table 3) compliant with the glycan microarray guidelines of MIRAGE, analogous to the MIAME guidelines for gene microarrays.

Research involving human participants, their data, or biological material

Policy information about studies with human participants or human data. See also policy information about sex, gender (identity/presentation), and sexual orientation and race, ethnicity and racism.

Reporting on sex and gender	N/A
Reporting on race, ethnicity, or other socially relevant groupings	N/A
Population characteristics	N/A
Recruitment	N/A
Ethics oversight	N/A

Note that full information on the approval of the study protocol must also be provided in the manuscript.

Field-specific reporting

Please select the one below that is the best fit for your research. If you are not sure, read the appropriate sections before making your selection.

☒ Life sciences ☐ Behavioural & social sciences ☐ Ecological, evolutionary & environmental sciences

For a reference copy of the document with all sections, see nature.com/documents/nr-reporting-summary-flat.pdf

Life sciences study design

All studies must disclose on these points even when the disclosure is negative.

Sample size	No sample size calculations were performed beforehand. A subset of 24 of the 150 available synthetic glycans were made at scale and taken forward for the glycan array screening study. These were selected to ensure that each individual chemical modification was represented in the set. Of these 10 glycans were included in the gold nanoparticle-based aggregation testing - they were selected to represent examples across the full range of glycan array binding scores.
Data exclusions	No data were excluded.
Replication	All attempts at replication were successful. The binding data presented for the glycan microarray are representative of a minimum of two independent experiments. Aggregation measurements were replicated 3 times to evaluate the replication and high agreement was observed. ITC experiments were each repeated three times.
Randomization	Not relevant for this study - no experiments involve allocating samples into groups.
Blinding	Samples were allocated arbitrary codes when testing for ease and to minimise bias. Analysis of glycan microarray data and gold nanoparticle-based aggregation assays were performed independently and the correlation of the two sets of data was performed by a third party.

Reporting for specific materials, systems and methods

We require information from authors about some types of materials, experimental systems and methods used in many studies. Here, indicate whether each material, system or method listed is relevant to your study. If you are not sure if a list item applies to your research, read the appropriate section before selecting a response.

Materials & experimental systems

n/a	Involved in the study
☐	☒ Antibodies
☒	☐ Eukaryotic cell lines
☒	☐ Palaeontology and archaeology
☒	☐ Animals and other organisms
☒	☐ Clinical data
☒	☐ Dual use research of concern
☒	☐ Plants

Methods

n/a	Involved in the study
☒	☐ ChIP-seq
☒	☐ Flow cytometry
☒	☐ MRI-based neuroimaging

Antibodies

Antibodies used

Anti-L5 (gift from Andrea Streit, King's College London) monoclonal rat IgM;
Anti-SSEA-1 (deposited to the Developmental Studies Hybridoma Bank (DSHB) by Solter, D. / Knowles, B.B (DSHB Hybridoma Product MC-480 (SSEA-1)) SSEA-1 is a carbohydrate epitope on glycolipids and glycoproteins involving fucosylated [GalBeta1-4(FucAlpha1-3) GlcNAcBeta1-R] type 2 blood group chains;
Anti-Lewisx/Anti-CD15 (BG-7) also known as Blood Group Antigen BG-7 Lewis x (BioLegend 912901, Previously Covance catalog# SIG-3339) The antigen is a trisaccharide structure, 3-fucosyl-N-acetyllactosamine, also know as Xhapten. This antigen is also expressed by granulocytes and a number of normal tissue types.

Validation

Anti-L5 (Streit, A.; Yuen, C.-T.; Loveless, R. W.; Lawson, A. M.; Finne, J.; Schmitz, B.; Feizi, T.; Stern, C. D. The Lex Carbohydrate Sequence Is Recognized by Antibody to L5, a Functional Antigen in Early Neural Development. J. Neurochem. 1996, 66 (2), 834-844, https://doi.org/10.1046/j.1471-4159.1996.66020834.x.);
Anti-SSEA-1: MC-480 recognizes mouse embryos, mouse embryonic cells (EC), mouse embryonic stem cells (ES) and mouse & human embryonic germ cells (EG). MC-480 does not bind to human EC, ES or iPS cells. SSEA-1 is a carbohydrate epitope on glycolipids and glycoproteins involving fucosylated [GalBeta1-4(FucAlpha1-3)GlcNAcBeta1-R] type 2 blood group chains. Antibody Registry ID: AB_528475;
Anti-Lewisx/Anti-CD15 (BG-7): This antibody is effective in immunohistochemistry (IHC). Positive tissue (human): Hodgkin's lymphoma, Kidney The BG-7 antibody, also known as CD15, clone P12 is specific for Lewis x (Type 2 Chain). This antibody reacts with an antigen present on Reed Sternberg (RS) cells.
